# Organocatalytic cycloaddition–elimination cascade for atroposelective construction of heterobiaryls†

**DOI:** 10.1039/d1sc05161j

**Published:** 2021-10-30

**Authors:** Wen-Lei Xu, Wei-Ming Zhao, Ru-Xia Zhang, Jie Chen, Ling Zhou

**Affiliations:** Key Laboratory of Synthetic and Natural Functional Molecule of the Ministry of Education, College of Chemistry & Materials Science, Northwest University Xi'an 710102 P. R. China zhoul@nwu.edu.cn

## Abstract

The first chiral phosphoric acid (CPA) catalyzed cycloaddition–elimination cascade reaction of 2-naphthol- and phenol-derived enecarbamates with azonaphthalenes has been established, providing a highly atroposelective route to an array of axially chiral aryl-C3-benzoindoles in excellent yields with excellent enantioselectivities. The success of this strategy derives from the stepwise process involving CPA-catalyzed asymmetric formal [3 + 2] cycloaddition and subsequent central-to-axial chirality conversion by elimination of a carbamate. In addition, the practicality of this reaction had been verified by varieties of transformations towards functionalized atropisomers.

## Abstract 2

An organocatalytic asymmetric cycloaddition–elimination cascade reaction of aryl enecarbamates with azonaphthalenes has been developed to access axially chiral heterobiaryls in excellent yields and enantioselectivities.

## Graphical abstract

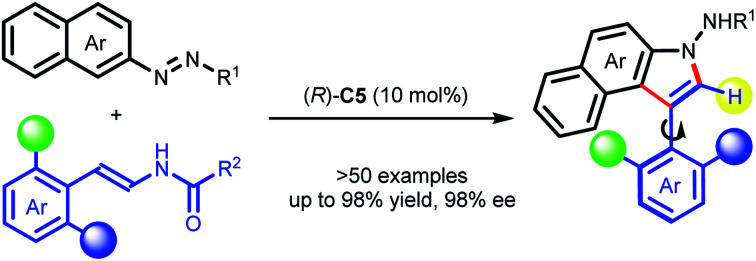

## Introduction

**Scheme 1:**
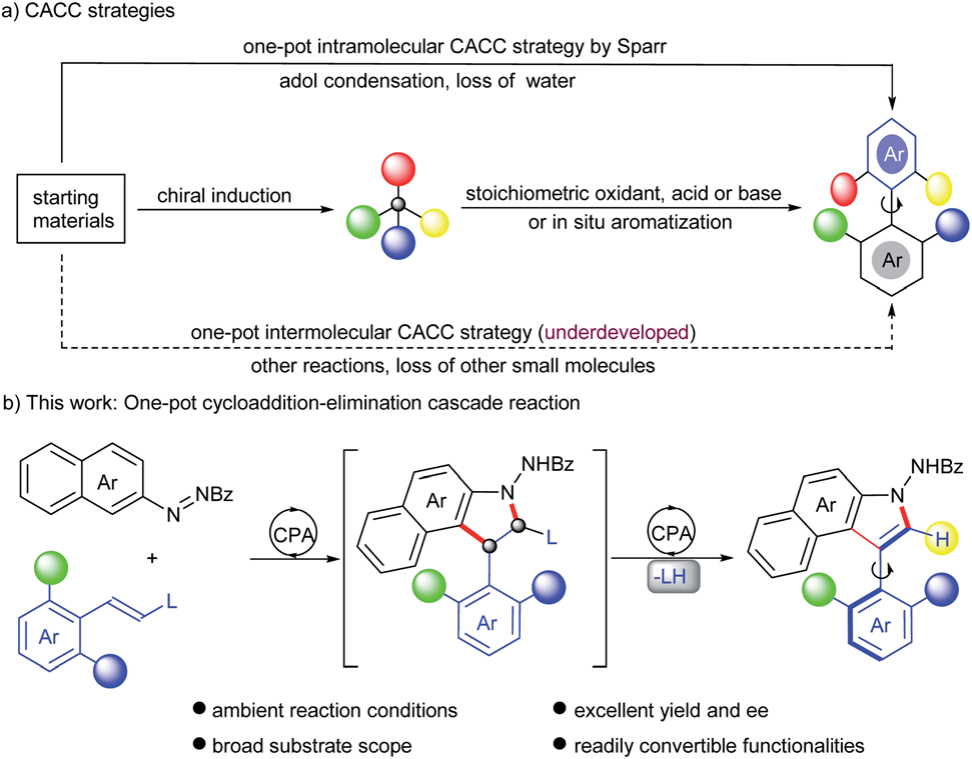
(a) CACC strategies; (b) this work: one-pot cycloaddition–elimination cascade reaction.

Axially chiral biaryls serve as the core motifs of abundant natural products, clinical drugs and functional materials.^1–3^ They also constitute privileged skeletons in lots of organocatalysts and ligands.^4^ Therefore, the development of novel and efficient methods to assemble structurally diverse axially chiral frameworks has attracted considerable attention from synthetic chemists.^1*a*,5^ As is well known, the central-to-axial chirality conversion (CACC) strategy has been developed into forceful tools to construct biaryl atropisomers and natural products that are not readily accessible otherwise.^6,7^ Despite the progress, most of these reactions require two steps including preparation of centrally chiral intermediates and then a chirality conversion step. Except for the driving force from *in situ* aromatization,^6*f*,*g*,8^ exogenous stoichiometric reagents, such as oxidants,^6*e*,*i*,*m*–*p*^ Lewis acids^6*c*,*j*^ and bases^6*h*,*k*^ are needed to trigger central-to-axial chirality conversion, which hampers the efficiency and is incongruous with the principle of step economy (Scheme 1a). In sharp contrast, only one example successively circumvents these drawbacks. Sparr and co-workers disclosed a secondary amine-catalyzed intramolecular asymmetric aldol condensation with concomitant dehydration for accessing various axially chiral skeletons (Scheme 1a).^7^ Given a lack of highly efficient routes in terms of the CACC strategy, the development of an alternative catalytic mode continues to be enthralling but challenging.

Indoles possessing significant bioactivity are diversely found in many natural alkaloids and pharmaceuticals.^9^ Particularly, indole-based axially chiral skeletons hold vast potential in bioactive molecules and asymmetric catalysis.^10^ Therefore, a suite of synthetic attempts were devoted to procuring such optically active compounds, including dynamic kinetic resolution,^11^ cyclization,^12^ atroposelective arylation,^8*c*,*i*,13^*de novo* construction of the indole ring^14^ and the CACC strategy.^6*m*,*n*^ Nevertheless, almost all generated atropisomeric indoles are installed with bulky substituents at the *ortho*-position around the axis, due to a lower rotation barrier and inferior conformational stability, which dramatically restricted the downstream functionalization as well as diversity-oriented synthesis of these chiral atropisomers. Exceptionally, Yan and co-workers devised an asymmetric annulation of *ortho*-alkynlanilines to produce axially chiral C3-unsubstituted naphthyl-indole scaffolds.^14*d*^ However, the enantioselective establishment of C2-unsubstituted indole-based biaryls remains underdeveloped. Inspired by conventional CACC strategies and asymmetric cycloadditon of alkenes with azonaphthalenes,^6*m*,*n*,8*c*,15^ we envisioned that axially chiral C2-unsubstituted aryl-indole skeletons might be obtained after sequential cycloaddition and elimination by introducing a leaving group into *ortho* disubstituted arylethylene substrates, *via* a catalytic CACC mode (Scheme 1b). However, several challenges were still imbedded in this strategy, including (1) finding a feasible leaving group with catalytic sites to activate arylethylene substrates; (2) selecting powerful catalysts to increase the reactivity and promote elimination of the leaving group as well; (3) efficiently inducing stereocontrol in the sequential cycloaddition and elimination steps. To address these challenges, the alkoxycarbonylamino group was selected as the leaving group because enecarbamates have long served as a reactive dipolarophile to deliver enantioenriched compounds bearing contiguous chiral centers,^16^ especially upon activation by chiral phosphoric acid (CPA).^16*c–i*^ In addition, the elimination of carbamate has proven to be feasible under strong acidic conditions;^17^ here CPA is expected to serve as an acid to realize the elimination. To the best of our knowledge, the construction of axially chiral biaryls by this designed CACC strategy has not been reported. Herein, we describe the first CPA-catalyzed cycloaddition–elimination cascade reaction of aryl enecarbamates with azonaphthalenes, providing a straightforward approach toward axially chiral aryl-C3-benzoindoles with excellent yields and enantioselectivities.

## Results and discussion

To probe the feasibility of this assumption, our reaction development commenced with 2-naphthol-derived benzyl enecarbamate **1A** and benzoyl azonaphthalene **2a** in the presence of chiral phosphoric acid (*R*)-**C1** (10 mol%) at room temperature. As expected, the desired product **3a** was isolated smoothly in 83% yield with 81% ee without any cycloaddition intermediate remained (Table S1,† entry 1).^18^ This proof-of-principle result demonstrated that the proposed CPA-catalyzed cycloaddition–elimination cascade reaction seemed to be feasible. Based on this promising result, reaction parameters such as temperatures and solvents were screened, and excellent yield and high ee values were observed in DCM at 0 °C for 30 h and then 30 °C for 6 h (Table S2,† entry 1).^18^ To further improve the enantioselectivity, a series of BINOL-, H8-BINOL- and SPINOL-derived CPA catalysts were evaluated (Table 1, entries 2–9), which indicated that the CPA (*R*)-**C5** could give rise to good enantiocontrol (Table 1, entry 5). In addition, the stereochemistry was also affected by different additives and O-protecting groups (Table 1, entries 10–15). An additive survey revealed Na_2_SO_4_ as the preferred choice with respect to enantioselectivity (Table 1, entry 10; Table S3†).^18^ Furthermore, the leaving group effect on enantioselectivity was tested in detail (Table 1, entries 16–19), and 2-naphthol-derived enecarbamate **1a** bearing the 2-furanylmethoxy group afforded axially chiral benzoindole derivative **3a** with 92% ee (Table 1, entry 19). Reduction of catalyst loading to 5 mol% resulted in retained ee but lower yield (Table 1, entry 20). The addition of H_2_O or EtOH caused significant decreases in the reaction stereoselectivity, which helps explain why hydrophilic additives produced slightly improved ee values (Table 1, entries 21–22). We finally identified the optimal conditions as follows: **1a** (0.12 mmol), **2a** (0.1 mmol), (*R*)-**C5** (10 mol%), Na_2_SO_4_ (40 mg), in DCM (4.0 mL) at 0 °C for 30 h and then 30 °C for 6 h, affording product **3a** in 94% yield with 92% ee. The structure of **3a** and its absolute configuration was confirmed by X-ray crystallography analysis of the 2-bromobenzoindole derivative (*vide infra*).

**Table 1:** Optimization of the reaction conditions^a^

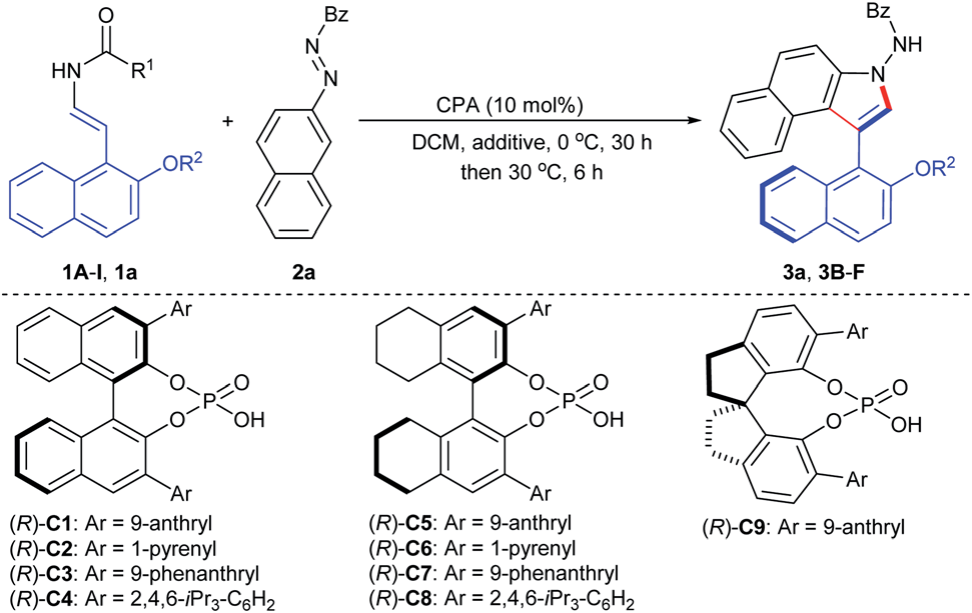					
Entry	**1** (R^1^/R^2^)	CPA	Additive	Yield^b^ (%)	ee^c^ (%)
1	**1A** (BnO/Me)	(*R*)-**C1**	—	**3a**, 90	86
2	**1A** (BnO/Me)	(*R*)-**C2**	—	**3a**, 78	55
3	**1A** (BnO/Me)	(*R*)-**C3**	—	**3a**, 91	71
4	**1A** (BnO/Me)	(*R*)-**C4**	—	**3a**, n.r.	—
5	**1A** (BnO/Me)	(*R*)-**C5**	—	**3a**, 93	88
6	**1A** (BnO/Me)	(*R*)-**C6**	—	**3a**, 90	64
7	**1A** (BnO/Me)	(*R*)-**C7**	—	**3a**, 69	70
8	**1A** (BnO/Me)	(*R*)-**C8**	—	**3a**, 8	11
9	**1A** (BnO/Me)	(*R*)-**C9**	—	**3a**, trace	—
10	**1A** (BnO/Me)	(*R*)-**C5**	Na_2_SO_4_	**3a**, 95	90
11	**1B** (BnO/Et)	(*R*)-**C5**	Na_2_SO_4_	**3B**, 95	84
12	**1C** (BnO/*n*-Pr)	(*R*)-**C5**	Na_2_SO_4_	**3C**, 87	83
13	**1D** (BnO/i-Pr)	(*R*)-**C5**	Na_2_SO_4_	**3D**, 93	89
14	**1E** (BnO/All)	(*R*)-**C5**	Na_2_SO_4_	**3E**, 92	78
15	**1F** (BnO/Bn)	(*R*)-**C5**	Na_2_SO_4_	**3F**, 90	67
16	**1G** (Pro/Me)	(*R*)-**C5**	Na_2_SO_4_	**3a**, 91	72
17	**1H** (Nmo/Me)	(*R*)-**C5**	Na_2_SO_4_	**3a**, 93	90
18	**1I** (BnS/Me)	(*R*)-**C5**	Na_2_SO_4_	**3a**, 95	90
19	**1a** (Fmo/Me)	(*R*)-**C5**	Na_2_SO_4_	**3a**, 94	92
20^d^	**1a** (Fmo/Me)	(*R*)-**C5**	Na_2_SO_4_	**3a**, 83	92
21^e^	**1a** (Fmo/Me)	(*R*)-**C5**	Na_2_SO_4_	**3a**, 87	81
22^f^	**1a** (Fmo/Me)	(*R*)-**C5**	Na_2_SO_4_	**3a**, 71	24

a Reactions were carried out with **1** (0.12 mmol), **2a** (0.10 mmol) and additive (40 mg) CPA (10 mol%) in DCM (4.0 mL) at 0 °C for 30 h and then 30 °C for further 6 h.

b Isolated yield.

c The ee value was determined by chiral HPLC analysis.

d 5 mol% of CPA was used.

e 10 mg H_2_O was added.

f 10 mg EtOH was added. Pro = *n*-propoxy. Nmo = 2-naphthylmethoxy. Fmo = 2-furanylmethoxy.

With the optimum conditions identified, we next explored the substrate generality of the asymmetric cycloaddition–elimination cascade reaction. Firstly, the 2-naphthol-derived enecarbamates **1a–t** were examined and the results are summarized in Table 2. Regardless of the type of functional groups, such as alkyl (Me, Et, i-Pr, *t*-Bu, and Bn), aryl (Ph), alkoxyl (OMe) and halogen (Br), at the C5- or C6- position on the naphthyl ring, the reaction proceeded smoothly with benzoyl azonaphthalene **2a** to generate the corresponding products (**3a–n**) in excellent yields with excellent enantioselectivities. However, the electronic properties of substituents at the C7-position on the naphthyl ring had an obvious effect on stereoselectivity. Compared with electron-withdrawing and -neutral groups, the electron-donating group at the C7-position returned product **3r** with outstanding results in both reactivity and enantiocontrol. 2-Naphthol-derived enecarbamates with C3-substituents on the naphthyl ring were also tolerated, furnishing the expected products **3s** and **3t** with good reaction outcomes by extending the reaction time, presumably due to the steric reason. Subsequently, the substrate scope in terms of the benzoyl group of azonaphthalenes was investigated. Phenyl rings with *para*- and *meta*-substituents were compatible, delivering products **3ab–ag** in remarkable results. Reduced enantiocontrol was gained by introducing substituents into the *ortho*-position of the phenyl ring, probably due to steric hindrance (**3ah**, **3ai**). To investigate the configurational stability of these prepared axially chiral aryl-C3-benzoindoles, a thermal racemization experiment was conducted. The rotation barrier of compound **3a** was calculated to be 31.1 kcal mol^−1^ at 100 °C, corresponding to a half-life of 107.0 years at 25 °C.

**Table 2:** Substrate generality for atroposelective synthesis of naphthyl-C3-benzoindoles^a^

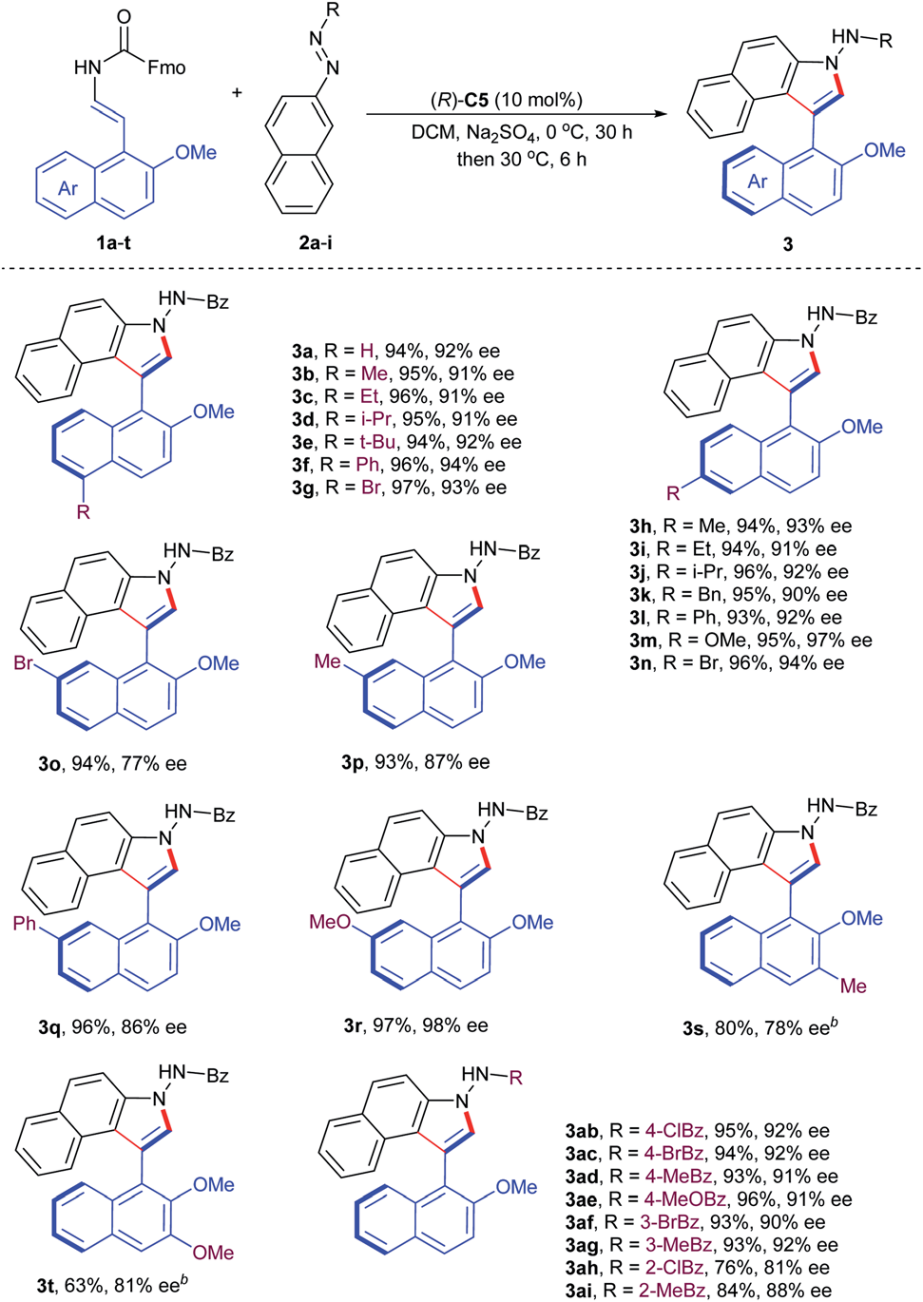

a Reactions were carried out with **1** (0.12 mmol), **2** (0.10 mmol), Na_2_SO_4_ (40 mg), and (*R*)-**C5** (10 mol%) in DCM (4.0 mL) at 0 °C for 30 h and then 30 °C for further 6 h. Isolated yield. The ee value was determined by chiral HPLC analysis.

b At 0 °C for 72 h and then 30 °C for further 12 h.

To further demonstrate the utility of this organocatalytic CACC methodology, we then turned our attention to the construction of axially chiral phenyl-benzoindoles (Table 3). The phenol-derived enecarbamates **1u–w** reacted effectively with benzoyl azonaphthalene **2a** under the optimal conditions, giving the desired products **3ua–wa** with outstanding enantioselectivities and yields. Then, the scope of benzoyl azonaphthalenes was examined. The positions and electron properties of substituents on benzoyl azonaphthalenes did not dramatically influence the results, since the corresponding products **3uj–us** were obtained in high yields with an enantiomeric excess range of 95–98%. Moreover, carbalkoxyl azonaphthalenes were further explored and generally provided the products **3ut–uz** with similar enantiocontrol. The absolute configuration of **3ut** was determined by an X-ray crystallographic study and other products were assigned analogously.^19^

**Table 3:** Substrate generality for atroposelective synthesis of phenyl-C3-benzoindoles^a^

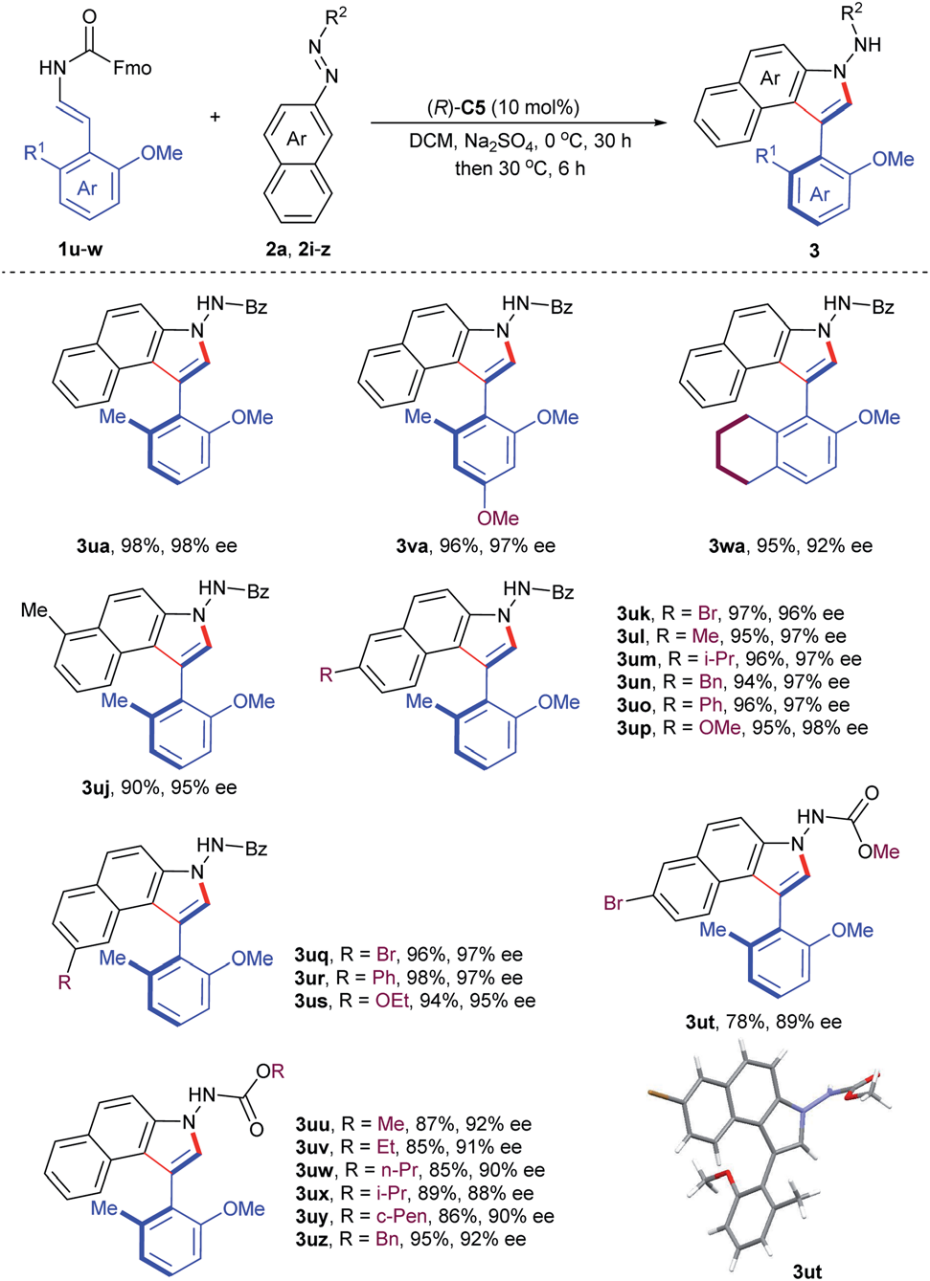

a Reactions were carried out with **1** (0.12 mmol), **2** (0.10 mmol), Na_2_SO_4_ (40 mg), and (*R*)-**C5** (10 mol%) in DCM (4.0 mL) at 0 °C for 30 h and then 30 °C for further 6 h. Isolated yield. The ee value was determined by chiral HPLC analysis.

**Scheme 2:**
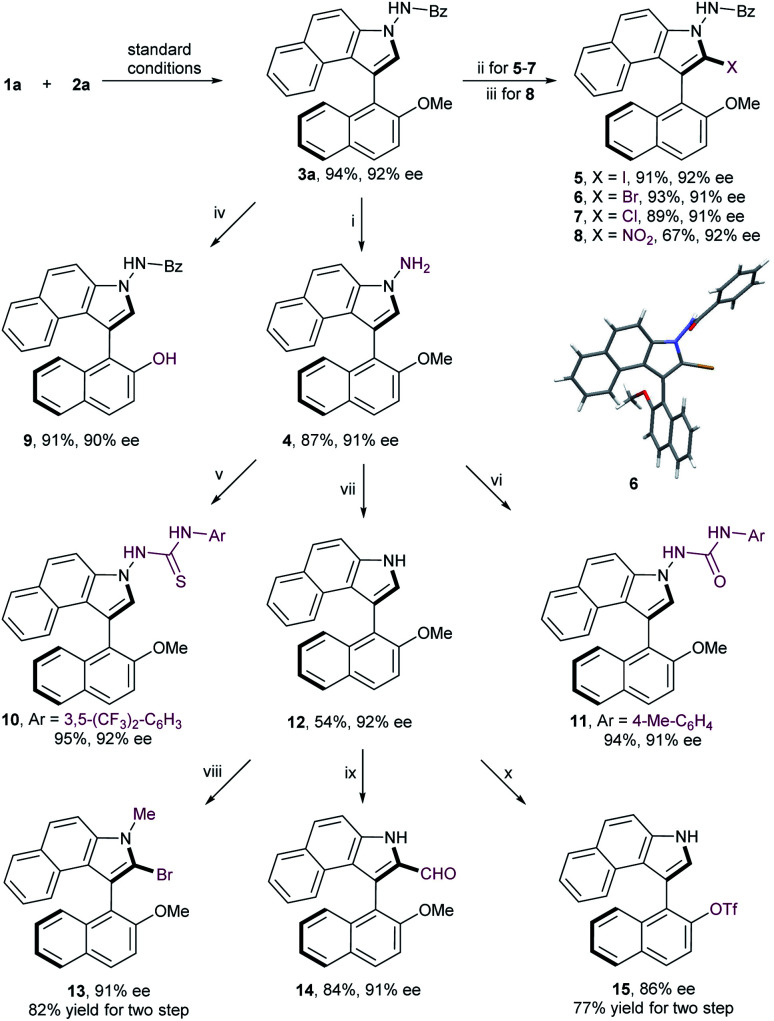
Gram-scale synthesis and further derivatizations. Reagents and conditions: (i) concentrated HCl/EtOH (1/2 v/v), 70 °C; (ii) NXS, TFA, MeCN, 0 °C; (iii) *t*-BuONO, MeCN, r.t.; (iv) NaSEt, DMF, 70 °C; (v) 1-isothiocyanato-3,5-bis(trifluoromethyl)benzene, MeCN, 0 °C; (vi) 1-isocyanato-4-methylbenzene, THF, r.t.; (vii) NaNO_2_, H_2_O/EtOH (1/2 v/v), 0 °C; (viii) NaH, MeI, THF, 0 °C; NBS, TFA, MeCN, 0 °C; (ix) DMF, POCl_3_, 0 °C; (x) BBr_3_, DCM, −78 °C; Tf_2_O, Et_3_N, DCM, 0 °C.

To substantiate the practicability of this new strategy, gram-scale synthesis of **3a** was performed under the optimal reaction conditions, which was obtained in comparable yield without deterioration of enantiomeric excess (95% yield, 92% ee; Scheme 2). Debenzoylation of compound **3a** gave the aminobiaryl **4** in 87% yield with 91% ee. Furthermore, a range of derivatizations of **3a** were conducted to expand the potential synthetic utility of this reaction. Halogenation of **3a** with corresponding halogen sources, such as NIS, NBS and NCS, afforded 2-iodobenzoindole **5**, 2-bromobenzoindole **6** and 2-chlorobenzoindole **7** in high yield with negligible erosion of optical purity, which serve as useful functional handles for further transformations. Atropisomeic 2-nitrobenzoindole **8** was also successfully synthesized directly from **3a** in moderate yield with retention of enantiopurity. More importantly, treatment of **3a** with sodium thioethylate at 70 °C led to a facile demethylation reaction, providing **9** in 91% yield with 90% ee. This finding enables the methyl moiety to play a temporary role, thus allowing downstream functionalization at the C2-position on the naphthyl ring. Further conversion of **4** with isocyanatoarene or isothiocyanatoarene proceeded smoothly to generate the axially chiral thiourea **10** and urea **11**, two potential organocatalysts, in good yields with no loss of enantiomeric purity. In addition, cleavage of the N–N bond was realized to produce N-unsubstituted naphthyl-C3-benzoindole **12** in moderate yield with the same enantiopurity. To further highlight the versatile reactivities of the C2-position on the benzoindolyl ring, the axially chiral product **12** was transformed into 1-methyl-2-bromobenzoindole **13** and 2-formylbenzoindole **14**, respectively. Moreover, the methoxy group of compound **12** could be readily converted to triflate, which is a crucial precursor to chiral monophosphorus ligands.^13^

**Scheme 3:**
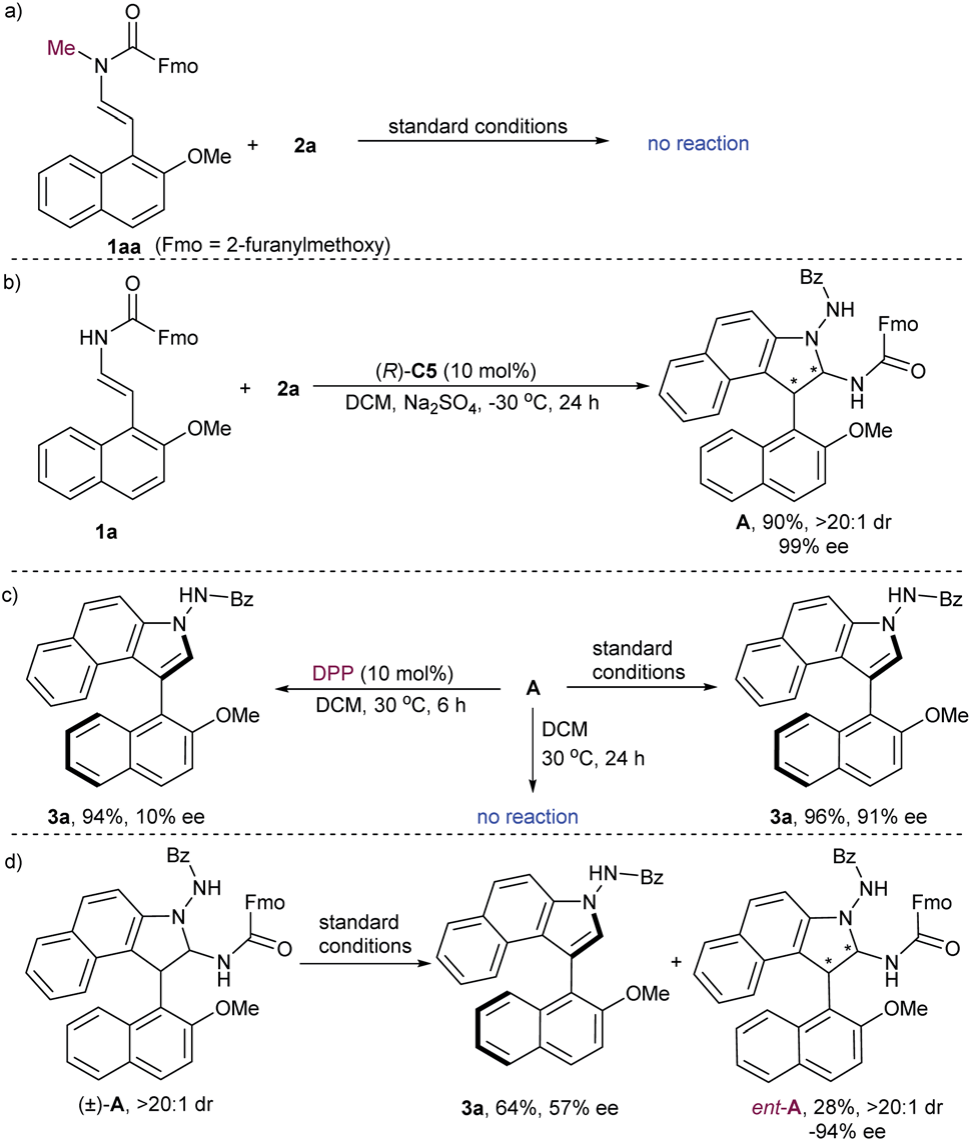
Mechanistic studies.

To gain some insight into the mechanism of this reaction, several control experiments were carried out (Scheme 3). No reaction proceeded when *N*-methyl-protected **1aa** was employed in the initial cycloaddition reaction, which suggested that the hydrogen-bonding between the N–H group and the P=O moiety of the catalyst is important for the reactivity (Scheme 3a). In addition, we performed a reaction between 2-naphthol-derived enecarbamate **1a** and benzoyl azonaphthalene **2a** under the catalysis of CPA (*R*)-**C5** at −30 °C for 24 h. The formal [3 + 2] cycloaddition intermediate **A** with diastereoisomeric conformers could be isolated in 90% yield with 99% ee and excellent diastereoselectivity (Scheme 3b).^18,20^ Unfortunately, these rotamers are inseparable, and the attempt to identify the absolute configuration of compound **A** failed. Nonetheless, some useful clues were observed from this centrally chiral intermediate. The desired axially chiral product **3a** was prepared from intermediate **A** with comparable yield and ee under the standard conditions, while poor chirality conversion was observed under the catalysis of diphenyl phosphate (DPP, 10% ee). On the other hand, treatment of **A** in the absence of CPA in DCM at 30 °C for 24 h afforded only the recovered **A**. These results implied that the CPA catalyst plays a crucial role in controlling the reactivity and enantioselectivity during the central-to-axial chirality conversion (Scheme 3c). When the racemic cycloaddition intermediate (±)-**A** was treated with CPA (*R*)-**C5** in DCM for 6 h, the product **3a** was obtained in 64% yield with 57% ee, and the starting material was recovered in 28% yield with opposite enantioselectivity (**ent-A**, −94% ee). These results suggested that the kinetic resolution of (±)-**A** through CPA-catalyzed elimination of a carbamate occurred (Scheme 3d). The preferential elimination of the fast-reacting enantiomer for racemic aminal intermediate (±)-**A** can then be rationalized through minimization of unfavourable steric hindrance between the aminal intermediate and the (*R*)-configurated CPA to form a steric configuration-matched iminium-phosphate ion pair.

**Fig. 1:**
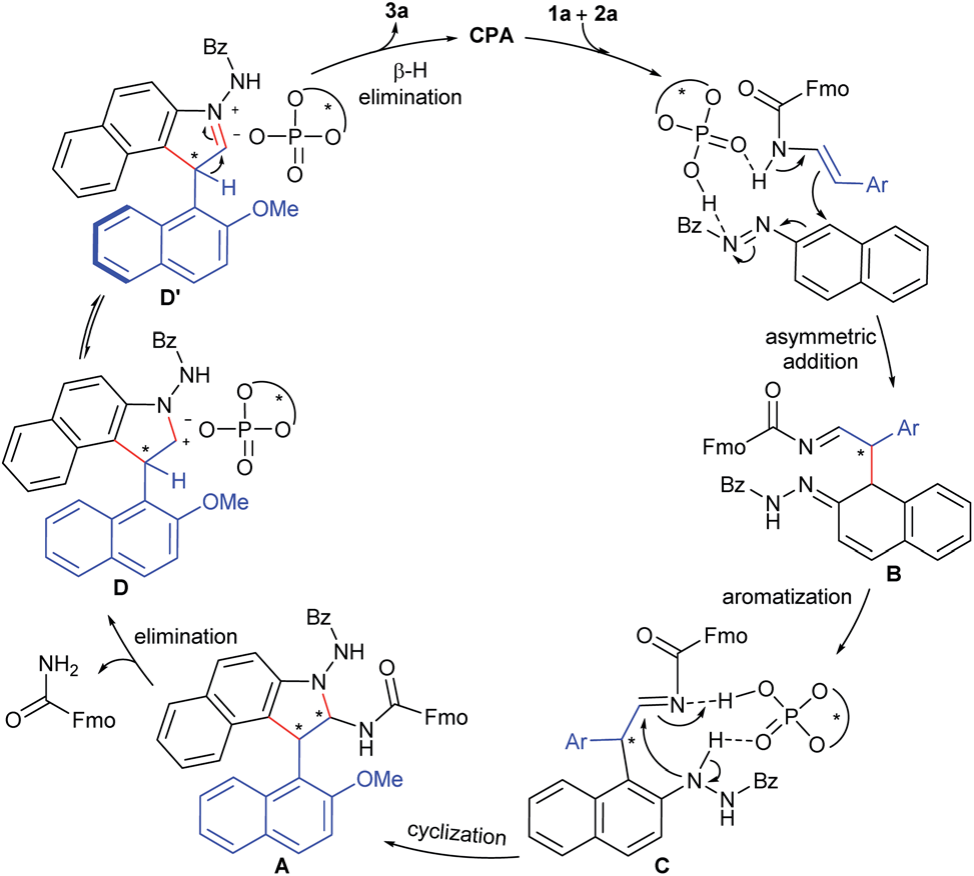
Proposed mechanism.

On the basis of these observations as well as previous literature,^4*f*,8*c*,16*i*,21^ a catalytic mechanism was proposed in Fig. 1. Firstly, the mechanistic studies suggest a stereochemical model for the enantioselective [3 + 2] cycloaddition, in which the activation of both 2-naphthol-derived enecarbamate **1a** and benzoyl azonaphthalene **2a** by a bifunctional catalyst through dual hydrogen-bonding facilitates nucleophilic attack at the α-position of **2a** to give the dearomatized intermediate **B**. Facile aromatization of intermediate **B** generates the arylhydrazine intermediate **C**. Then, cyclization occurs *via* intramolecular aminalization to afford cycloaddition aminal intermediate **A**. Subsequently, the CPA catalyst could accelerate elimination of a carbamate from intermediate **A** to form the identical steric configuration-matched iminium-phosphate ion pair **D** or **D′**. Finally, further release of CPA by β-H elimination leads to aromatization to accomplish the central-to-axial chirality conversion, which ultimately affords the axially chiral product **3a**.

In summary, we have developed the first well-designed CPA-catalyzed asymmetric cycloaddition–elimination cascade reactions of 2-naphthol- or phenol-derived enecarbamates with azonaphthalenes. A wide range of naphthyl-C3-benzoindoles and phenyl-C3-benzoindoles with functionality versatility could be obtained in high yields with good to excellent enantioselectivities. This reaction shuns the use of extra steps and exogenous stoichiometric reagents, and thus represents as a step- and atom-economical concept. Furthermore, the synthetic utility of this protocol was explored *via* convenient functional group transformation methods. Mechanistic studies disclosed that chiral phosphoric acid played significant roles in controlling both the reactivity as well as enantioselectivity to the cycloaddition and central-to-axial chirality conversion and showed efficient kinetic resolution performance on this type of aminal. This work will not only provide a straightforward alternative to access C2-unsubstituted axially chiral aryl-C3-benzoindoles, but will also open new avenues for conventional central-to-axial chirality conversion. Further investigation of the mechanism in detail and utilization of this strategy for the synthesis of substantial atropisomeric biaryl backbones are currently undergoing in this laboratory.

## Data availability

All experimental and crystallographic data is available in the ESI.†

## Author contributions

W.-L. X., W.-M. Z. and R.-X. Z. performed all the experiments. W.-L. X., J. C. and L. Z. contributed to the conception of the experiments, discussion of the results and preparation of the manuscript.

## Conflicts of interest

There are no conflicts to declare.

## Supplementary Material

SC-012-D1SC05161J-s001

SC-012-D1SC05161J-s002
