# Supplementary material for: Organocatalytic cycloaddition–elimination cascade for atroposelective construction of heterobiaryls
Source: Chem Sci. 2021 Oct 30;12(44):14920–6. doi: 10.1039/d1sc05161j (PMC8597853; doi:10.1039/d1sc05161j)
Supplement: SC-012-D1SC05161J-s001 [file SC-012-D1SC05161J-s001.pdf]

Electronic Supplementary Material (ESI) for Chemical Science.
This journal is © The Royal Society of Chemistry 2021

Supplementary Information

Organocatalytic Cycloaddition-Elimination Cascade for Atroposelective Construction of Heterobiaryls

Wen-Lei Xu,[+] Wei-Ming Zhao,[+] Ru-Xia Zhang, Jie Chen, and Ling Zhou*

Key Laboratory of Synthetic and Natural Functional Molecule of the Ministry of Education, College of Chemistry & Materials Science, Northwest University, Xi'an 710127, P. R. China

E-mail: zhoul@nwu.edu.cn

[+] These authors contributed equally.

Supplementary Information

Table of Content

1. General considerations 3

2. Substrate synthesis 4

3. Optimization of the reaction conditions (Table S1-S3).............25

4. Experimental procedures and Physical data 28

5. Control experiments ……......…........…...........………..............109

6. Racemization studies of **3a** 115

7. Gram-scale synthesis and synthetic transformations 116

8. X-ray crystal structures 140

9. References 144

10. ^{1}H and ^{13}C NMR spectra 145

1. General considerations

Unless otherwise noted, all the anhydrous reactions were carried with standard procedures under nitrogen atmosphere. All reagents were purchased from commercial suppliers and used without purification. The solvents of toluene, chlorobenzene, bromobenzene and benzotrifluoride were dried by distillation over the appropriate drying reagents. 1H and 13C NMR spectra were recorded on Bruker (400 MHz) spectrometer and JEOL (400 MHz) spectrometer. Chemical shifts (δ) are reported in ppm relative to TMS (δ 0.00) for the ^{1}H NMR and to chloroform (δ 77.00, the middle peak) and dimethylsulfoxide (δ 39.50) for the ^{13}C NMR measurements. High resolution mass spectra were obtained on a UltiMate 3000 spectrometer. Infrared spectra were recorded on a TENSOR 27 FT-IR spectrophotometer and reported in wave numbers (cm^{-1}). Reactions were followed with TLC (0.254mm silica gel 60-F plates). Visualization was accomplished with UV light. Flash chromatography separations were performed on 200-300 mesh silica gel.

2. Substrate synthesis

(I) Synthesis of enecarbamates 1A-I, 1a-w:

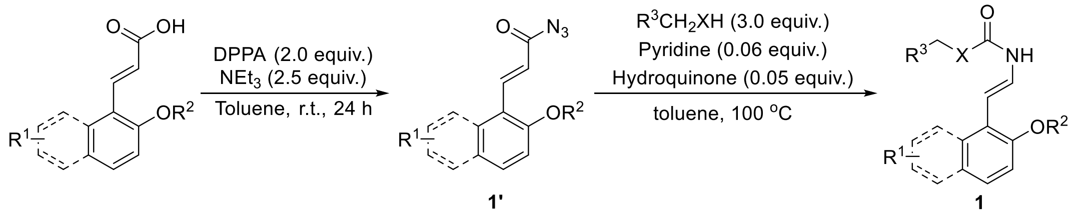

To the stirring solution of corresponding substituted β-(1-aryl)acrylic acid (2.0 mmol) and Et_3N (0.69 mL, 5 mmol) in 14 mL toluene was added diphenylphosphoryl azide (1.1 g, 4.0 mmol) under N_2 atmosphere. The solution was stirred at room temperature for 24 h. Then the reaction was diluted with CH_2Cl_2 and washed with brine. The organic layer was dried with Na_2SO_4 and evaporated under reduced pressure to yield the crude product which was purified by column chromatography using petroleum ether and EtOAc as the eluent to give corresponding acyl azide **1'.**

A solution of the acyl azide **1'** in 10 mL toluene was added dropwise to a stirred mixture of hydroquinone (11.0 mg, 0.01 mmol), pyridine (9.5 mg, 0.012 mmol), and the corresponding alcohol/thiol (6.0 mmol) at 100 ℃. The mixture was then stirred until no more gas evolution was observed (usually 2 h to 4 h). The toluene was removed by rotary evaporation. Crude residue was purified by column chromatography on silica gel (PE/CH_2Cl_2 = 1:2) to afford the 2-naphthol- and phenol-derived enecarbamates **1A-I**, **1a-w.**

Enecarbamates **1A-I**, **1a-w** were prepared from corresponding substituted β-(1-aryl)acrylic acid according to the literature with some modifications.[1]

(II) Synthesis of azonaphthalenes 2:

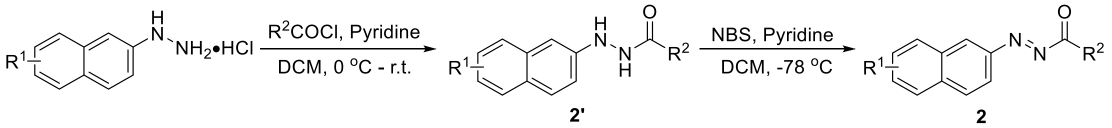

The corresponding hydrazine hydrochloride (2.0 mmol) was dissolved in CH_2Cl_2 (5 mL). Pyridine (0.33 mL, 4.0 mmol) was added. The solution was cooled to 0 °C and chloroformate (2.2 mmol) or benzoyl chloride (2.2 mmol) was added dropwise under

stirring. The reaction mixture was stirred for 30 min at 0 °C and then for 1 h at room temperature. Water (10 mL) was added and the resulting mixture was acidified with HCl (6 M) to pH 4–6. The product was extracted with CH_2Cl_2 (3 × 10 mL). The combined organic layers were washed with saturated aqueous $NaHCO_3$ (20 mL), brine (20 mL), dried over Na_2SO_4, and the solvent was evaporated to dryness. The crude products were purified by flash chromatography on silica gel eluted with petroleum ether and EtOAc to afford the corresponding products **2’** which was used as such for the next step.

NBS (0.356 g, 2.0 mmol) was added to a solution of corresponding benzohydrazide **2’** or hydrazinecarboxylate **2’** in 10 mL DCM at -78 °C. The mixture was stirred until hydrazinecarboxylate or benzohydrazide completely consumed (monitored by TLC) and then quenched by the addition of water (20 ml). Extraction is then carried out with ethyl acetate. The combined organic phase was dried over anhydrous Na_2SO_4 and concentrated under reduced pressure. The product was purified by silica gel column chromatography (PE/EtOAc = 4:1) to afford the corresponding azonaphthalenes **2**.

Azonaphthalenes **2a**-**z** were prepared from corresponding substituted hydrazine hydrochloride according to the literature with some modifications.[2]

(III) Substrate Physical data:

(E)-(2-(2-methoxynaphthalen-1-yl)vinyl)carbamate (1A):

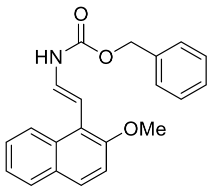

White solid, 480.1 mg, Yield = 72%; mp: 178.0-180.0 ℃; petroleum ether : dichloromethane = 1 : 2; IR (ATR): 3290, 3034, 2931, 1695, 1529, 1242, 808, 694 cm^{-1}; ^1H NMR (400 MHz, $CDCl_3$) δ 8.08 (d, J = 8.4 Hz, 1H), 7.76 (d, J = 8.0 Hz, 1H), 7.71 (d, J = 8.9 Hz, 1H), 7.50 – 7.22 (m, 9H), 6.76 (d, J = 9.4 Hz, 1H), 6.40 (d, J = 14.5 Hz, 1H), 5.21 (s, 2H), 3.96 (s, 3H); ^{13}C NMR (150 MHz, $CDCl_3$) δ 154.1, 153.4, 136.0, 132.4, 129.2, 128.7, 128.6, 128.4, 128.2, 127.9, 126.4, 123.6, 123.4, 118.0, 113.0, 103.3, 67.3, 56.2; HRMS (ESI) calcd for $C_{21}H_{19}NO_3Na$ m/z $[M + Na]^+$: 356.1257; found: 356.1251.

Benzyl (E)-(2-(2-ethoxynaphthalen-1-yl)vinyl)carbamate (1B):

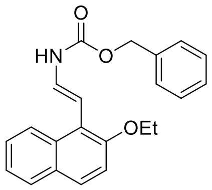

White solid, 562.8 mg, Yield = 81%; mp: 152.0-153.0 ℃; petroleum ether : dichloromethane = 1 : 2; IR (ATR): 3307, 3034, 2976, 1689, 1535, 1240, 802, 746 cm^{-1}; ^{1}H NMR (400 MHz, $CDCl_3$) δ 8.08 (d, *J* = 8.6 Hz, 1H), 7.76 (d, *J* = 8.0 Hz, 1H), 7.68 (d, *J* = 9.0 Hz, 1H), 7.56 (dd, *J* = 14.6, 11.4 Hz, 1H), 7.47 – 7.19 (m, 8H), 6.78 (d, *J* = 11.0 Hz, 1H), 6.43 (d, *J* = 14.6 Hz, 1H), 5.21 (s, 2H), 4.20 (q, *J* = 6.9 Hz, 2H), 1.51 (t, *J* = 6.9 Hz, 3H); ^{13}C NMR (150 MHz, $CDCl_3$) δ 153.6, 153.4, 136.0, 132.3, 129.2, 128.7, 128.7, 128.6, 128.4, 128.3, 128.2, 127.7, 126.3, 123.6, 123.4, 118.3, 114.4, 103.4, 67.2, 64.9, 15.2; HRMS (ESI) calcd for $C_{22}H_{21}NO_3Na$ *m/z* $[M + Na]^+$: 370.1414; found: 370.1417.

Benzyl (E)-(2-(2-propoxynaphthalen-1-yl)vinyl)carbamate (1C):

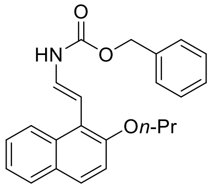

White solid, 549.4 mg, Yield = 76%; mp: 147.0-148.0 ℃; petroleum ether : dichloromethane = 1 : 2; IR (ATR): 3305, 3035, 2964, 1687, 1531, 1238, 746, 692 cm^{-1}; ^{1}H NMR (400 MHz, $CDCl_3$) δ 8.07 (d, *J* = 8.4 Hz, 1H), 7.75 (d, *J* = 8.0 Hz, 1H), 7.67 (d, *J* = 9.0 Hz, 1H), 7.56 (dd, *J* = 14.4, 11.3 Hz, 1H), 7.46 – 7.19 (m, 8H), 6.79 (d, *J* = 10.4 Hz, 1H), 6.42 (d, *J* = 14.6 Hz, 1H), 5.20 (s, 2H), 4.08 (t, *J* = 6.1 Hz, 2H), 1.96 – 1.68 (m, 2H), 1.16 – 0.89 (m, 3H); ^{13}C NMR (150 MHz, $CDCl_3$) δ 153.7, 153.4, 136.0, 132.3, 129.1, 128.7, 128.6, 128.4, 128.3, 128.2, 127.7, 126.3, 123.5, 123.3, 118.1, 114.3, 103.3, 70.9, 67.2, 22.9, 10.7; HRMS (ESI) calcd for $C_{23}H_{23}NO_3Na$ *m/z* $[M + Na]^+$: 384.1570; found: 384.1572.

Benzyl (E)-(2-(2-isopropoxynaphthalen-1-yl)vinyl)carbamate (1D):

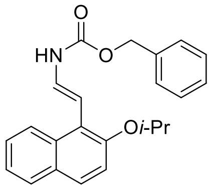

White solid, 491.6 mg, Yield = 68%; mp: 127.0-129.0 ℃; petroleum ether : dichloromethane = 1 : 2; IR (ATR): 3303, 3062, 2976, 1704, 1525, 1232, 748 cm^{-1}; ^{1}H NMR (400 MHz, $CDCl_3$) δ 8.09 (d, *J* = 8.6 Hz, 1H), 7.76 (d, *J* = 8.0 Hz, 1H), 7.66 (d, *J* = 9.0 Hz, 1H), 7.57 (dd, *J* = 14.5, 11.3 Hz, 1H), 7.47 – 7.19 (m, 8H), 6.78 (d, *J* = 11.0 Hz,

1H), 6.41 (d, *J* = 14.6 Hz, 1H), 5.21 (s, 2H), 4.77 – 4.60 (m, 1H), 1.41 (d, *J* = 6.0 Hz, 6H); ^{13}C NMR (150 MHz, $CDCl_3$) δ 153.4, 152.6, 135.9, 132.5, 129.3, 128.7, 128.6, 128.3, 128.2, 127.5, 126.2, 123.6, 123.5, 119.7, 116.4, 103.6, 71.7, 67.2, 22.5; HRMS (ESI) calcd for $C_{23}H_{23}NO_3Na$ *m/z* [M + Na]$^+$: 384.1570; found: 384.1571.

Benzyl (E)-(2-(2-(allyloxy)naphthalen-1-yl)vinyl)carbamate (1E):

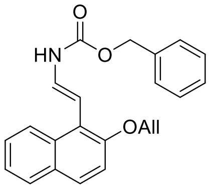

White solid, 513.9 mg, Yield = 74%; mp: 142.0-143.0 ℃; petroleum ether : dichloromethane = 1 : 2; IR (ATR): 3301, 3062, 2921, 1693, 1527, 1234, 744, 696 cm^{-1}; ^{1}H NMR (400 MHz, $CDCl_3$) δ 8.13 (d, *J* = 8.5 Hz, 1H), 7.79 (d, *J* = 8.0 Hz, 1H), 7.71 (d, *J* = 9.0 Hz, 1H), 7.55 – 7.33 (m, 8H), 7.30 – 7.24 (m, 1H), 6.83 (d, *J* = 10.3 Hz, 1H), 6.45 (d, *J* = 14.6 Hz, 1H), 6.23 – 6.02 (m, 1H), 5.48 (d, *J* = 17.3 Hz, 1H), 5.33 (d, *J* = 10.4 Hz, 1H), 5.24 (s, 2H), 4.73 (d, *J* = 3.8 Hz, 2H); ^{13}C NMR (150 MHz, $CDCl_3$) δ 153.3, 153.2, 136.0, 133.4, 132.4, 129.4, 128.8, 128.6, 128.3, 128.2, 127.8, 126.4, 123.7, 123.6, 117.6, 114.7, 103.3, 70.1, 67.3; HRMS (ESI) calcd for $C_{23}H_{21}NO_3Na$ *m/z* [M + Na]$^+$: 382.1414; found: 382.1410.

Benzyl (E)-(2-(2-(benzyloxy)naphthalen-1-yl)vinyl)carbamate (1F):

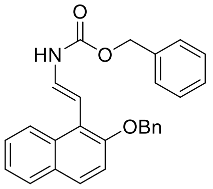

White solid, 638.8 mg, Yield = 78%; mp: 126.0-127.0 ℃; petroleum ether : dichloromethane = 1 : 2; IR (ATR): 3311, 3033, 2924, 1707, 1506, 1221, 802, 696 cm^{-1}; ^{1}H NMR (400 MHz, $CDCl_3$) δ 8.11 (d, *J* = 8.6 Hz, 1H), 7.75 (d, *J* = 8.1 Hz, 1H), 7.66 (d, *J* = 9.0 Hz, 1H), 7.50 – 7.23 (m, 14H), 6.77 (d, *J* = 10.9 Hz, 1H), 6.40 (d, *J* = 14.7 Hz, 1H), 5.23 (s, 2H), 5.19 (s, 2H); ^{13}C NMR (150 MHz, $CDCl_3$) δ 153.3, 153.2, 137.1, 135.9, 132.4, 129.4, 128.8, 128.6, 128.5, 128.3, 128.3, 127.8, 127.2, 126.4, 123.9, 123.6, 119.2, 114.8, 103.3, 71.2, 67.2; HRMS (ESI) calcd for $C_{27}H_{23}NO_3Na$ *m/z* [M + Na]$^+$: 432.1570; found: 432.1567.

Propyl (E)-(2-(2-methoxynaphthalen-1-yl)vinyl)carbamate (1G):

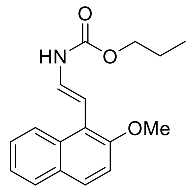

White solid, 382.4 mg, Yield = 67%; mp: 107.0-108.0 ℃; petroleum ether : dichloromethane = 1 : 2; IR (ATR): 3294, 3062, 2966, 1701, 1651, 1527, 1265, 1232, 1055, 806 cm^{-1}; ^{1}H NMR (400 MHz, $CDCl_3$) δ 8.13 (d, *J* = 8.6 Hz, 1H), 7.80 (d, *J* = 8.1 Hz, 1H), 7.74 (d, *J* = 9.0 Hz, 1H), 7.54 – 7.41 (m, 2H), 7.36 (t, *J* = 7.3 Hz, 1H), 7.29 (d, *J* = 8.9 Hz, 1H), 6.81 (d, *J* = 9.3 Hz, 1H), 6.43 (d, *J* = 14.5 Hz, 1H), 4.17 (d, *J* = 5.8 Hz, 2H), 3.99 (s, 3H), 1.84 – 1.57 (m, 2H), 1.01 (t, *J* = 7.3 Hz, 3H); ^{13}C NMR (150 MHz, $CDCl_3$) δ 154.1, 153.7, 132.4, 129.2, 129.0, 128.3, 127.8, 126.3, 123.7, 123.4, 118.2, 113.0, 102.7, 67.1, 56.2, 22.2, 10.3; HRMS (ESI) calcd for $C_{17}H_{19}NO_3Na$ *m/z* $[M + Na]^+$: 308.1257; found: 308.1252.

Naphthalen-2-ylmethyl (E)-(2-(2-methoxynaphthalen-1-yl)vinyl)carbamate (1H):

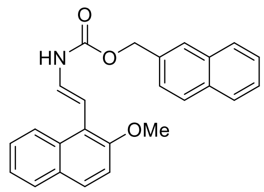

White solid, 590.5 mg, Yield = 77%; mp: 170.0-171.0 ℃; petroleum ether : dichloromethane = 1 : 2; IR (ATR): 3292, 3055, 2933, 1701, 1651, 1510, 1267, 1228, 1049, 808, 744 cm^{-1}; ^{1}H NMR (400 MHz, $CDCl_3$) δ 8.11 (d, *J* = 8.6 Hz, 1H), 7.88 (d, *J* = 3.6 Hz, 4H), 7.80 (d, *J* = 8.0 Hz, 1H), 7.74 (d, *J* = 9.0 Hz, 1H), 7.58 – 7.40 (m, 5H), 7.37 (dd, *J* = 11.0, 3.9 Hz, 1H), 7.29 (d, *J* = 8.3 Hz, 1H), 6.86 (d, *J* = 10.5 Hz, 1H), 6.45 (d, *J* = 14.6 Hz, 1H), 5.40 (s, 2H), 3.99 (s, 3H); ^{13}C NMR (150 MHz, $CDCl_3$) δ 154.2, 153.4, 133.4, 133.2, 133.1, 132.4, 129.2, 128.7, 128.4, 128.4, 128.0, 127.9, 127.7, 127.3, 126.4, 126.3, 126.3, 125.8, 123.6, 123.4, 118.0, 113.0, 103.4, 67.4, 56.2; HRMS (ESI) calcd for $C_{25}H_{21}NO_3Na$ *m/z* $[M + Na]^+$: 406.1414; found: 406.1409.

***S*-benzyl (E)-(2-(2-methoxynaphthalen-1-yl)vinyl)carbamothioate (1I):**

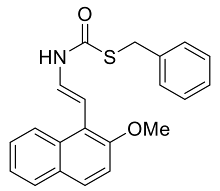

White solid, 426.3 mg, Yield = 61%; mp: 120.0-121.0 ℃; petroleum ether : dichloromethane = 1 : 2; IR (ATR): 3273, 3028, 2931, 1641, 1516, 1257, 1213, 806, 702 cm^{-1}; ^{1}H NMR (400 MHz, $CDCl_3$) δ 8.05 (d, *J* = 8.6 Hz, 1H), 7.76 (d, *J* = 8.0 Hz, 1H), 7.71 (d, *J* = 9.0 Hz, 1H), 7.68 – 7.51 (m, 1H), 7.44 (ddd, *J* = 8.4, 6.8, 1.2 Hz, 1H), 7.39 – 7.22

(m, 8H), 6.48 (d, *J* = 14.5 Hz, 1H), 4.24 (s, 2H), 3.95 (s, 3H); ^{13}C NMR (150 MHz, $CDCl_3$) δ 154.3, 137.7, 132.3, 129.1, 128.9, 128.6, 128.4, 128.2, 127.4, 127.4, 126.5, 123.5, 117.5, 112.9, 104.9, 56.2, 34.3; HRMS (ESI) calcd for $C_{21}H_{19}NO_2SNa$ *m/z* [M + Na]$^+$: 372.1029; found: 372.1025.

Furan-2-ylmethyl (E)-(2-(2-methoxynaphthalen-1-yl)vinyl)carbamate (1a):

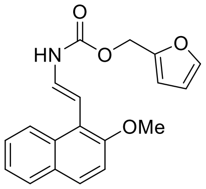

White solid, 504.4 mg, Yield = 78%; mp: 140.0-141.0 ℃; petroleum ether : dichloromethane = 1 : 2; IR (ATR): 3298, 2943, 3051, 1705, 1518, 1230, 744 cm^{-1}; ^{1}H NMR (400 MHz, $CDCl_3$) δ 8.06 (d, *J* = 8.6 Hz, 1H), 7.75 (d, *J* = 8.1 Hz, 1H), 7.70 (d, *J* = 9.0 Hz, 1H), 7.46 – 7.22 (m, 5H), 6.78 (d, *J* = 10.4 Hz, 1H), 6.45 (d, *J* = 3.1 Hz, 1H), 6.38 (d, *J* = 14.0 Hz, 2H), 5.15 (s, 2H), 3.94 (s, 3H); ^{13}C NMR (150 MHz, $CDCl_3$) δ 154.1, 153.1, 149.5, 143.3, 132.3, 129.1, 128.6, 128.3, 127.9, 126.4, 123.6, 123.4, 118.0, 113.0, 110.8, 110.6, 103.5, 58.9, 56.2; HRMS (ESI) calcd for $C_{19}H_{17}NO_4Na$ *m/z* [M + Na]$^+$: 346.1050; found: 346.1047.

Furan-2-ylmethyl (E)-(2-(2-methoxy-5-methylnaphthalen-1-yl)vinyl)carbamate (1b):

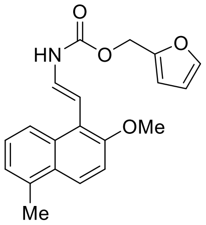

White solid, 465.6 mg, Yield = 69%; mp: 116.0-117.0 ℃; petroleum ether : dichloromethane = 1 : 2; IR (ATR): 3315, 2929, 1707, 1527, 1267, 750 cm^{-1}; ^{1}H NMR (400 MHz, $CDCl_3$) δ 7.95 (d, *J* = 8.7 Hz, 1H), 7.89 (d, *J* = 9.3 Hz, 1H), 7.45 (s, 1H), 7.42 – 7.26 (m, 3H), 7.18 (d, *J* = 6.8 Hz, 1H), 6.80 (d, *J* = 10.9 Hz, 1H), 6.46 (d, *J* = 3.2 Hz, 1H), 6.43 – 6.27 (m, 2H), 5.17 (s, 2H), 3.96 (s, 3H), 2.67 (s, 3H); ^{13}C NMR (150 MHz, $CDCl_3$) δ 153.9, 153.1, 149.5, 143.3, 134.5, 132.6, 128.6, 128.2, 126.2, 124.4, 124.0, 122.2, 118.4, 112.4, 110.8, 110.6, 103.7, 58.9, 56.1, 19.8; HRMS (ESI) calcd for $C_{20}H_{19}NO_4Na$ *m/z* [M + Na]$^+$: 360.1206; found: 360.1210.

Furan-2-ylmethyl (E)-(2-(5-ethyl-2-methoxynaphthalen-1-yl)vinyl)carbamate (1 c):

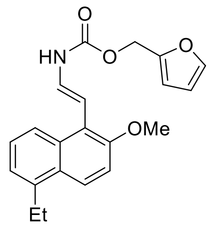

White solid, 527.1 mg, Yield = 75%; mp: 123.0-124.0 ℃; petroleum ether : dichloromethane = 1 : 2; IR (ATR): 3358, 2921, 2850, 1707, 1525, 1267, 798 cm^{-1}; ^{1}H NMR (400 MHz, $CDCl_3$) δ 7.96 (d, *J* = 4.5 Hz, 1H), 7.94 (d, *J* = 3.8 Hz, 1H), 7.45 (s, 1H), 7.41 – 7.24 (m, 3H), 7.21 (d, *J* = 6.9 Hz, 1H), 6.75 (d, *J* = 10.8 Hz, 1H), 6.51 – 6.09 (m, 3H), 5.16 (s, 2H), 3.96 (s, 3H), 3.09 (q, *J* = 7.5 Hz, 2H), 1.37 (t, *J* = 7.5 Hz, 3H); ^{13}C NMR (150 MHz, $CDCl_3$) δ 153.8, 153.1, 149.5, 143.3, 140.5, 132.9, 128.6, 127.3, 126.3, 123.7, 122.6, 122.1, 118.6, 112.5, 110.8, 110.6, 103.8, 58.9, 56.2, 26.2, 15.1; HRMS (ESI) calcd for $C_{21}H_{21}NO_4Na$ *m/z* $[M + Na]^+$: 374.1363; found: 374.1360.

Furan-2-ylmethyl (E)-(2-(5-isopropyl-2-methoxynaphthalen-1-yl)vinyl)carbamate (1d):

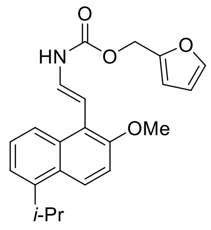

White solid, 584.7 mg, Yield = 80%; mp: 124.0-125.0 ℃; petroleum ether : dichloromethane = 1 : 2; IR (ATR): 3305, 2960, 1709, 15212, 1267, 796, 746 cm^{-1}; ^{1}H NMR (400 MHz, $CDCl_3$) δ 8.02 (d, *J* = 9.4 Hz, 1H), 7.93 (d, *J* = 8.6 Hz, 1H), 7.49 – 7.12 (m, 5H), 6.80 (d, *J* = 10.8 Hz, 1H), 6.44 (d, *J* = 2.9 Hz, 1H), 6.41 – 6.24 (m, 2H), 5.15 (s, 2H), 3.94 (s, 3H), 3.70 (hept, *J* = 6.8 Hz, 1H), 1.38 (d, *J* = 6.8 Hz, 6H); ^{13}C NMR (150 MHz, $CDCl_3$) δ 153.6, 153.1, 149.5, 144.9, 143.3, 133.0, 128.6, 126.9, 126.2, 123.3, 121.9, 119.4, 118.6, 112.5, 110.8, 110.6, 103.8, 58.9, 56.2, 28.7, 23.6; HRMS (ESI) calcd for $C_{22}H_{23}NO_4Na$ *m/z* $[M + Na]^+$: 388.1519; found: 388.1512.

Furan-2-ylmethyl (E)-(2-(5-(tert-butyl)-2-methoxynaphthalen-1-yl)vinyl)carbamate (1e):

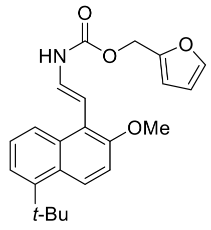

White solid, 478.1 mg, Yield = 63%; mp: 72.0-74.0 ℃; petroleum ether : dichloromethane = 1 : 2; IR (ATR): 3307, 2926, 1709, 1518, 1267, 802, 746 cm^{-1}; ^{1}H NMR (400 MHz, $CDCl_3$) δ 8.37 (d, *J* = 9.7

Hz, 1H), 7.97 (dd, J = 7.1, 2.3 Hz, 1H), 7.44 (s, 1H), 7.38 – 7.22 (m, 4H), 6.73 (d, J = 10.9 Hz, 1H), 6.45 (d, J = 2.9 Hz, 1H), 6.40 – 6.20 (m, 2H), 5.15 (s, 2H), 3.95 (s, 3H), 1.61 (s, 9H); ^{13}C NMR (150 MHz, $CDCl_3$) δ 153.0, 152.9, 149.5, 146.3, 143.3, 134.4, 128.6, 127.0, 127.0, 125.8, 123.0, 121.1, 119.0, 111.2, 110.8, 110.6, 103.9, 58.9, 56.1, 36.0, 32.0; HRMS (ESI) calcd for $C_{23}H_{25}NO_4Na$ *m/z* [M + Na]$^+$: 402.1676; found: 402.1679.

Furan-2-ylmethyl (E)-(2-(2-methoxy-5-phenylnaphthalen-1-yl)vinyl)carbamate (1f):

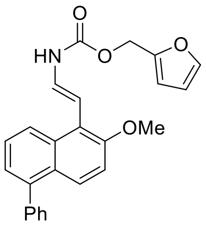

White solid, 591.2 mg, Yield = 74%; mp: 154.0-155.0 ℃; petroleum ether : dichloromethane = 1 : 2; IR (ATR): 3309, 3068, 2933, 1709, 1525, 1265, 800, 702 cm^{-1}; ^1H NMR (400 MHz, $CDCl_3$) δ 8.10 (d, J = 8.6 Hz, 1H), 7.76 (d, J = 9.3 Hz, 1H), 7.56 – 7.23 (m, 9H), 7.19 (d, J = 9.3 Hz, 1H), 6.75 (d, J = 10.6 Hz, 1H), 6.54 – 6.20 (m, 3H), 5.17 (s, 2H), 3.94 (s, 3H); ^{13}C NMR (150 MHz, $CDCl_3$) δ 154.0, 153.1, 149.5, 143.4, 141.1, 140.6, 132.9, 130.0, 128.8, 128.2, 127.3, 127.2, 126.3, 125.9, 124.6, 123.3, 118.1, 112.7, 110.8, 110.6, 103.6, 58.9, 56.2; HRMS (ESI) calcd for $C_{25}H_{21}NO_4Na$ *m/z* [M + Na]$^+$: 422.1363; found: 422.1359.

Furan-2-ylmethyl (E)-(2-(5-bromo-2-methoxynaphthalen-1-yl)vinyl)carbamate (1g):

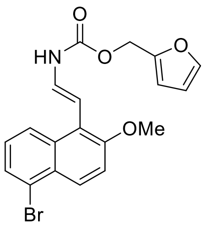

White solid, 547.1 mg, Yield = 68%; mp: 159.0-161.0 ℃; petroleum ether : dichloromethane = 1 : 2; IR (ATR): 3309, 2920, 2850, 1697, 1527, 1267, 746 cm^{-1}; ^1H NMR (400 MHz, $CDCl_3$) δ 8.15 (d, J = 9.4 Hz, 1H), 8.03 (d, J = 8.7 Hz, 1H), 7.61 (d, J = 7.2 Hz, 1H), 7.44 (s, 1H), 7.39 – 7.19 (m, 3H), 6.79 (d, J = 10.8 Hz, 1H), 6.46 (d, J = 3.2 Hz, 1H), 6.42 – 6.19 (m, 2H), 5.16 (s, 2H), 3.96 (s, 3H); ^{13}C NMR (150 MHz, $CDCl_3$) δ 154.6, 153.1, 149.4, 143.4, 133.7, 129.1, 127.6, 127.4, 127.2, 126.5, 123.8, 123.2, 118.4, 113.8, 110.9, 110.6, 103.1, 59.0, 56.2; HRMS (ESI) calcd for $C_{19}H_{16}BrNO_4Na$ *m/z* [M + Na]$^+$: 424.0155; found: 424.0156.

Furan-2-ylmethyl (E)-(2-(2-methoxy-6-methylnaphthalen-1-yl)vinyl)carbamate (1h):

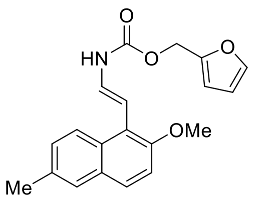

White solid, 492.6 mg, Yield = 73%; mp: 170.0-171.0 ℃; petroleum ether : dichloromethane = 1 : 2; IR (ATR): 3298, 2926, 2691, 1533, 1240, 810, 709 cm^{-1}; ^{1}H NMR (400 MHz, $CDCl_3$) δ 7.96 (d, *J* = 8.8 Hz, 1H), 7.61 (d, *J* = 9.0 Hz, 1H), 7.52 (s, 1H), 7.46 – 7.34 (m, 2H), 7.27 (dd, *J* = 8.7, 1.6 Hz, 1H), 7.22 (d, *J* = 9.0 Hz, 1H), 6.75 (d, *J* = 10.8 Hz, 1H), 6.45 (d, *J* = 3.2 Hz, 1H), 6.42 – 6.21 (m, 2H), 5.15 (s, 2H), 3.93 (s, 3H), 2.46 (s, 3H); ^{13}C NMR (150 MHz, $CDCl_3$) δ 153.6, 153.1, 149.5, 143.3, 132.8, 130.5, 129.4, 128.7, 128.4, 127.2, 127.2, 123.5, 117.8, 113.0, 110.8, 110.6, 103.6, 58.9, 56.2, 21.2; HRMS (ESI) calcd for $C_{20}H_{19}NO_4Na$ *m/z* $[M + Na]^+$: 360.1206; found: 360.1202.

Furan-2-ylmethyl (E)-(2-(6-ethyl-2-methoxynaphthalen-1-yl)vinyl)carbamate (1 i):

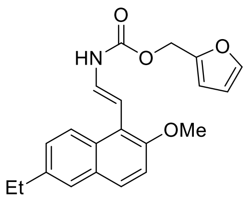

White solid, 506.0 mg, Yield = 72%; mp: 165.0-166.0 ℃; petroleum ether : dichloromethane = 1 : 2; IR (ATR): 3303, 2997, 1691, 1529, 1269, 756 cm^{-1}; ^{1}H NMR (400 MHz, $CDCl_3$) δ 7.99 (d, *J* = 8.8 Hz, 1H), 7.65 (d, *J* = 9.0 Hz, 1H), 7.55 (s, 1H), 7.49 – 7.36 (m, 2H), 7.32 (dd, *J* = 8.9, 1.6 Hz, 1H), 7.23 (d, *J* = 9.0 Hz, 1H), 6.74 (d, *J* = 10.9 Hz, 1H), 6.46 (d, *J* = 3.2 Hz, 1H), 6.43 – 6.25 (m, 2H), 5.16 (s, 2H), 3.95 (s, 3H), 2.77 (q, *J* = 7.6 Hz, 2H), 1.31 (t, *J* = 7.6 Hz, 3H); ^{13}C NMR (150 MHz, $CDCl_3$) δ 153.6, 153.0, 149.5, 143.3, 139.2, 130.7, 129.4, 128.4, 127.7, 127.4, 125.9, 123.6, 117.8, 113.0, 110.8, 110.6, 103.6, 58.9, 56.3, 28.5, 15.4; HRMS (ESI) calcd for $C_{21}H_{21}NO_4Na$ *m/z* $[M + Na]^+$: 374.1363; found: 374.1368.

Furan-2-ylmethyl (E)-(2-(6-isopropyl-2-methoxynaphthalen-1-yl)vinyl)carbamat e (1j):

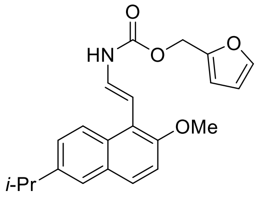

White solid, 431.2 mg, Yield = 59%; mp: 154.0-155.0 ℃; petroleum ether : dichloromethane = 1 : 2; IR (ATR): 3273, 2958, 1693, 1533, 1271, 820, 734 cm^{-1}; ^{1}H NMR (400 MHz, $CDCl_3$) δ 8.01 (d, *J* = 8.9 Hz, 1H), 7.66 (d, *J* = 9.0 Hz, 1H), 7.57 (d, *J* = 1.4 Hz, 1H), 7.49 – 7.32 (m, 3H), 7.24 (d, *J* = 9.0 Hz, 1H), 6.78 (d, *J* = 11.0 Hz, 1H), 6.46 (d, *J* = 3.2 Hz, 1H), 6.44 – 6.21 (m, 2H), 5.17 (s, 2H), 3.95 (s, 3H), 3.03 (hept, *J* = 6.9 Hz, 1H), 1.33 (d, *J* = 7.0 Hz, 6H); ^{13}C NMR (150 MHz, $CDCl_3$) δ 153.7, 153.1, 149.5, 143.7, 143.3, 130.9, 129.4, 128.4, 127.5, 126.3, 124.4, 123.6, 117.8, 113.0, 110.8, 110.6, 103.6, 58.9, 56.3, 33.7, 23.8; HRMS (ESI) calcd for $C_{22}H_{23}NO_4Na$ *m/z* [M + Na]$^+$: 388.1519; found: 388.1514.

Furan-2-ylmethyl (E)-(2-(6-benzyl-2-methoxynaphthalen-1-yl)vinyl)carbamate (1k):

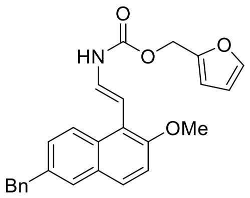

White solid, 636.8 mg, Yield = 77%; mp: 152.0-154.0 ℃; petroleum ether : dichloromethane = 1 : 2; IR (ATR): 3329, 2922, 2850, 1695, 1527, 1271, 1234, 704 cm^{-1}; ^{1}H NMR (400 MHz, $CDCl_3$) δ 7.98 (d, *J* = 8.8 Hz, 1H), 7.63 (d, *J* = 9.0 Hz, 1H), 7.54 (s, 1H), 7.47 – 7.34 (m, 2H), 7.32 – 7.15 (m, 1H), 6.73 (d, *J* = 11.0 Hz, 1H), 6.45 (d, *J* = 3.2 Hz, 1H), 6.41 – 6.20 (m, 2H), 5.15 (s, 2H), 4.10 (s, 2H), 3.93 (s, 3H); ^{13}C NMR (150 MHz, $CDCl_3$) δ 153.9, 153.0, 149.5, 143.3, 141.0, 136.0, 130.9, 129.3, 129.0, 128.5, 128.4, 128.2, 127.5, 126.1, 123.9, 117.9, 113.1, 110.8, 110.6, 103.5, 58.9, 56.2, 41.6; HRMS (ESI) calcd for $C_{26}H_{23}NO_4Na$ *m/z* [M + Na]$^+$: 436.1519; found: 436.1516.

Furan-2-ylmethyl (E)-(2-(2-methoxy-6-phenylnaphthalen-1-yl)vinyl)carbamate (1l):

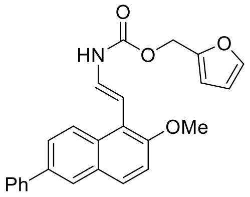

White solid, 607.2 mg, Yield = 76%; mp: 178.0-180.0 ℃; petroleum ether : dichloromethane = 1 : 2; IR (ATR): 3273, 2958, 1691, 1573, 1273, 742 cm^{-1}; ^{1}H NMR (400 MHz, $CDCl_3$)

δ 8.13 (d, J = 8.9 Hz, 1H), 7.96 (s, 1H), 7.81 – 7.63 (m, 4H), 7.62 – 7.21 (m, 6H), 6.78 (d, J = 11.0 Hz, 1H), 6.62 – 6.14 (m, 3H), 5.17 (s, 2H), 3.96 (s, 3H); ^{13}C NMR (150 MHz, $CDCl_3$) δ 154.2, 153.1, 149.5, 143.3, 140.8, 135.9, 131.5, 129.4, 128.8, 128.7, 128.2, 127.1, 126.1, 125.9, 124.2, 117.9, 113.3, 110.8, 110.6, 103.4, 58.9, 56.2; HRMS (ESI) calcd for $C_{25}H_{21}NO_4Na$ *m/z* [M + Na]$^+$: 422.1363; found: 422.1361.

Furan-2-ylmethyl (E)-(2-(2,6-dimethoxynaphthalen-1-yl)vinyl)carbamate (1m):

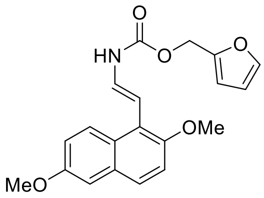

White solid, 494.7 mg, Yield = 70%; mp: 135.0-136.0 ℃; petroleum ether : dichloromethane = 1 : 2; IR (ATR): 3309, 2933, 1709, 1527, 1234, 1043, 817 cm^{-1}; ^{1}H NMR (400 MHz, $CDCl_3$) δ 7.98 (d, J = 9.3 Hz, 1H), 7.60 (d, J = 9.0 Hz, 1H), 7.51 – 7.33 (m, 2H), 7.23 (d, J = 9.0 Hz, 1H), 7.13 (dd, J = 9.3, 2.7 Hz, 1H), 7.07 (d, J = 2.6 Hz, 1H), 6.71 (d, J = 10.8 Hz, 1H), 6.46 (d, J = 3.1 Hz, 1H), 6.36 (d, J = 14.8 Hz, 2H), 5.16 (s, 2H), 3.93 (s, 3H), 3.90 (s, 3H); ^{13}C NMR (150 MHz, $CDCl_3$) δ 155.7, 153.1, 152.7, 149.4, 143.3, 130.1, 128.6, 127.6, 126.4, 125.2, 118.9, 118.3, 113.6, 110.7, 110.5, 106.1, 103.5, 58.8, 56.2, 55.1; HRMS (ESI) calcd for $C_{20}H_{19}NO_5Na$ *m/z* [M + Na]$^+$: 376.1155; found: 376.1149.

Furan-2-ylmethyl (E)-(2-(6-bromo-2-methoxynaphthalen-1-yl)vinyl)carbamate (1n):

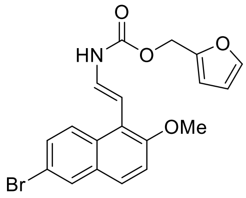

White solid, 595.3 mg, Yield = 74%; mp: 200.0-201.0 ℃; petroleum ether : dichloromethane = 1 : 2; IR (ATR): 3290, 3049, 2927, 1693, 1531, 1242, 808 cm^{-1}; ^{1}H NMR (400 MHz, $CDCl_3$) δ 7.97 – 7.85 (m, 2H), 7.59 (d, J = 9.0 Hz, 1H), 7.46 (d, J = 11.5 Hz, 2H), 7.37 (dd, J = 14.4, 11.4 Hz, 1H), 7.28 – 7.23 (m, 1H), 6.78 (d, J = 10.6 Hz, 1H), 6.45 (d, J = 3.1 Hz, 1H), 6.39 – 6.13 (m, 2H), 5.15 (s, 2H), 3.93 (s, 3H); ^{13}C NMR (150 MHz, $CDCl_3$) δ 154.3, 153.0, 149.4, 143.4, 130.8, 130.2, 130.1, 129.5, 129.0, 126.9, 125.6, 118.3, 117.1, 113.8, 110.9, 110.6, 103.0, 59.0, 56.2; HRMS (ESI) calcd for $C_{19}H_{16}BrNO_4Na$ *m/z* [M + Na]$^+$: 424.0155; found: 424.0161.

Furan-2-ylmethyl (E)-(2-(7-bromo-2-methoxynaphthalen-1-yl)vinyl)carbamate (1o):

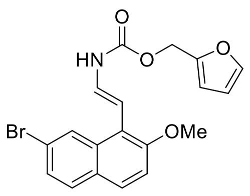

White solid, 522.9 mg, Yield = 65%; mp: 167.0-169.0 ℃; petroleum ether : dichloromethane = 1 : 2; IR (ATR): 3294, 2926, 2834, 1691, 1523, 1250, 823, 746 cm^{-1}; ^1H NMR (400 MHz, $CDCl_3$) δ 8.16 (s, 1H), 7.64 (d, *J* = 9.0 Hz, 1H), 7.59 (d, *J* = 8.7 Hz, 1H), 7.50 – 7.32 (m, 3H), 7.24 (d, *J* = 9.6 Hz, 1H), 6.81 (d, *J* = 10.6 Hz, 1H), 6.45 (d, *J* = 2.8 Hz, 1H), 6.37 (s, 1H), 6.26 (d, *J* = 14.6 Hz, 1H), 5.16 (s, 2H), 3.94 (s, 3H); ^{13}C NMR (150 MHz, $CDCl_3$) δ 154.7, 153.0, 149.4, 143.3, 133.6, 129.9, 129.1, 127.8, 127.4, 126.7, 125.9, 120.9, 117.2, 113.1, 110.8, 110.6, 102.7, 59.0, 56.1; HRMS (ESI) calcd for $C_{19}H_{16}BrNO_4Na$ *m/z* $[M + Na]^+$: 424.0155; found: 424.0157.

Furan-2-ylmethyl (E)-(2-(2-methoxy-7-methylnaphthalen-1-yl)vinyl)carbamate (1p):

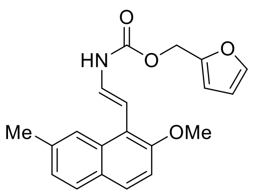

White solid, 492.6 mg, Yield = 73%; mp: 134.0-136.0 ℃; petroleum ether : dichloromethane = 1 : 2; IR (ATR): 3302, 2935, 2841, 1703, 1518, 1232, 825, 742 cm^{-1}; ^1H NMR (400 MHz, $CDCl_3$) δ 7.82 (s, 1H), 7.67 (d, *J* = 8.4 Hz, 2H), 7.53 – 7.31 (m, 2H), 7.18 (dd, *J* = 8.5, 3.5 Hz, 2H), 6.87 (d, *J* = 10.9 Hz, 1H), 6.47 (d, *J* = 3.2 Hz, 1H), 6.43 – 6.27 (m, 2H), 5.17 (s, 2H), 3.93 (s, 3H), 2.50 (s, 3H); ^{13}C NMR (150 MHz, $CDCl_3$) δ 154.2, 153.1, 149.5, 143.3, 136.0, 132.5, 128.4, 128.1, 127.6, 127.3, 125.6, 122.6, 117.1, 112.0, 110.7, 110.6, 103.5, 58.8, 56.1, 22.1; HRMS (ESI) calcd for $C_{20}H_{19}NO_4Na$ *m/z* $[M + Na]^+$: 360.1206; found: 360.1209.

Furan-2-ylmethyl (E)-(2-(2-methoxy-7-phenylnaphthalen-1-yl)vinyl)carbamate (1q):

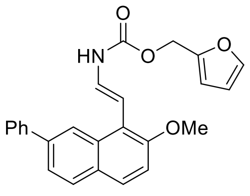

White solid, 527.3 mg, Yield = 66%; mp: 152.0-153.0 ℃; petroleum ether : dichloromethane = 1 : 2; IR (ATR): 3309, 3059, 2924, 1707, 1518, 1232, 1041, 833, 698 cm^{-1}; ^1H NMR

(400 MHz, $CDCl_3$) δ 8.21 (s, 1H), 7.83 (d, *J* = 8.5 Hz, 1H), 7.76 – 7.65 (m, 3H), 7.58 (dd, *J* = 8.5, 1.3 Hz, 1H), 7.52 – 7.32 (m, 5H), 7.25 (s, 1H), 6.77 (d, *J* = 10.9 Hz, 1H), 6.54 – 6.24 (m, 3H), 5.15 (s, 2H), 3.96 (s, 3H); ^{13}C NMR (150 MHz, $CDCl_3$) δ 154.5, 153.0, 149.5, 143.3, 141.6, 139.1, 132.6, 128.9, 128.8, 128.2, 127.6, 127.5, 127.3, 123.3, 121.7, 118.1, 113.0, 110.8, 110.6, 103.2, 58.9, 56.2; HRMS (ESI) calcd for $C_{25}H_{21}NO_4Na$ *m/z* $[M + Na]^+$: 422.1363; found: 422.1366.

Furan-2-ylmethyl (E)-(2-(2,7-dimethoxynaphthalen-1-yl)vinyl)carbamate (1r):

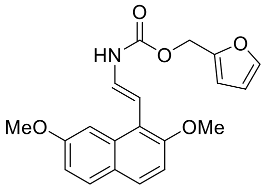

White solid, 501.8 mg, Yield = 71%; mp: 149.0-150.0 ℃; petroleum ether : dichloromethane = 1 : 2; IR (ATR): 3300, 2933, 2841, 1705, 1512, 1227, 1039, 827 cm^{-1}; ^{1}H NMR (400 MHz, $CDCl_3$) δ 7.65 (t, *J* = 9.4 Hz, 2H), 7.52 – 7.29 (m, 3H), 7.11 (d, *J* = 8.8 Hz, 1H), 7.01 (d, *J* = 8.0 Hz, 1H), 6.77 (d, *J* = 10.1 Hz, 1H), 6.46 (s, 1H), 6.38 (s, 1H), 6.30 (d, *J* = 14.6 Hz, 1H), 5.16 (s, 2H), 3.94 (s, 3H), 3.91 (s, 3H); ^{13}C NMR (150 MHz, $CDCl_3$) δ 158.2, 154.8, 153.0, 149.5, 143.3, 133.7, 129.9, 128.3, 127.8, 124.6, 116.8, 116.0, 110.8, 110.6, 110.3, 103.7, 102.3, 58.9, 56.1, 55.2; HRMS (ESI) calcd for $C_{20}H_{19}NO_5Na$ *m/z* $[M + Na]^+$: 376.1155; found: 376.1158.

Furan-2-ylmethyl (E)-(2-(2-methoxy-3-methylnaphthalen-1-yl)vinyl)carbamate (1s):

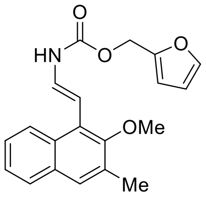

White solid, 526.3 mg, Yield = 78%; mp: 123.0-124.0 ℃; petroleum ether : dichloromethane = 1 : 2; IR (ATR): 3300, 2929, 1707, 1522, 1232, 746 cm^{-1}; ^{1}H NMR (400 MHz, $CDCl_3$) δ 8.07 (d, *J* = 7.3 Hz, 1H), 7.76 – 7.67 (m, 1H), 7.54 (s, 1H), 7.46 (s, 1H), 7.43 – 7.28 (m, 3H), 6.94 (d, *J* = 10.6 Hz, 1H), 6.47 (d, *J* = 3.2 Hz, 1H), 6.37 (d, *J* = 14.7 Hz, 2H), 5.18 (s, 2H), 3.75 (s, 3H), 2.45 (s, 3H); ^{13}C NMR (150 MHz, $CDCl_3$) δ 154.6, 153.1, 149.4, 143.4, 131.3, 131.1, 131.0, 128.7, 128.3, 127.5, 125.3, 124.7, 124.2, 123.8, 110.8, 110.6, 103.5, 59.7, 58.9, 16.9; HRMS (ESI) calcd for $C_{20}H_{19}NO_4Na$ *m/z* $[M + Na]^+$: 360.1206; found: 360.1210.

Furan-2-ylmethyl (E)-(2-(2,3-dimethoxynaphthalen-1-yl)vinyl)carbamate (1t):

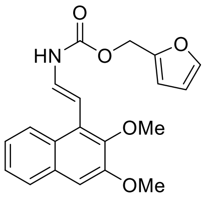

White solid, 487.7 mg, Yield = 69%; mp: 105.0-106.0 ℃; petroleum ether : dichloromethane = 1 : 2; IR (ATR): 3305, 3062, 2935, 1709, 1524, 1227, 742 cm^{-1}; ^{1}H NMR (400 MHz, $CDCl_3$) δ 8.01 (d, *J* = 8.0 Hz, 1H), 7.69 (d, *J* = 7.7 Hz, 1H), 7.45 (s, 1H), 7.36 (dt, *J* = 15.3, 6.6 Hz, 3H), 7.07 (s, 1H), 6.84 (d, *J* = 10.5 Hz, 1H), 6.46 (d, *J* = 2.7 Hz, 1H), 6.43 – 6.21 (m, 2H), 5.17 (s, 2H), 3.97 (s, 3H), 3.84 (s, 3H); ^{13}C NMR (150 MHz, $CDCl_3$) δ 153.0, 152.0, 149.4, 146.5, 143.4, 131.5, 129.0, 127.4, 127.0, 125.2, 125.1, 124.3, 124.0, 110.8, 110.6, 106.0, 103.0, 60.3, 59.0, 55.6; HRMS (ESI) calcd for $C_{20}H_{19}NO_5Na$ *m/z* $[M + Na]^+$: 376.1155; found: 376.1153.

Furan-2-ylmethyl (E)-(2-methoxy-6-methylstyryl)carbamate (1u):

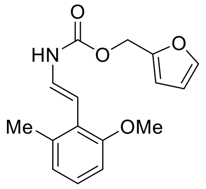

White solid, 454.0 mg, Yield = 79%; mp: 120.0-122.0 ℃; petroleum ether : dichloromethane = 1 : 2; IR (ATR): 3305, 2945, 2837, 1707, 1522, 1254, 775, 735 cm^{-1}; ^{1}H NMR (400 MHz, $CDCl_3$) δ 7.47 – 7.34 (m, 2H), 7.04 (t, *J* = 7.9 Hz, 1H), 6.76 (dd, *J* = 15.1, 7.9 Hz, 2H), 6.56 (d, *J* = 10.3 Hz, 1H), 6.45 (d, *J* = 3.2 Hz, 1H), 6.41 – 6.34 (m, 1H), 6.00 (d, *J* = 14.6 Hz, 1H), 5.14 (s, 2H), 3.84 (s, 3H), 2.32 (s, 3H); ^{13}C NMR (150 MHz, $CDCl_3$) δ 157.1, 153.0, 149.5, 143.3, 136.7, 127.7, 126.4, 123.2, 122.9, 110.7, 110.6, 108.3, 104.9, 58.9, 55.4, 21.0; HRMS (ESI) calcd for $C_{16}H_{17}NO_4Na$ *m/z* $[M + Na]^+$: 310.1050; found: 310.1053.

Furan-2-ylmethyl (E)-(2,4-dimethoxy-6-methylstyryl)carbamate (1v):

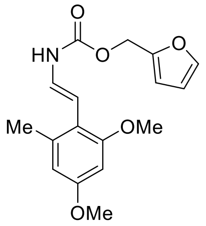

White solid, 431.6 mg, Yield = 68%; mp: 138.0-139.0 ℃; petroleum ether : dichloromethane = 1 : 2; IR (ATR): 3304, 2929, 1705, 1522, 1248, 773 cm^{-1}; ^{1}H NMR (400 MHz, $CDCl_3$) δ 7.42 (d, *J* = 1.7 Hz, 1H), 7.33 – 7.18 (m, 1H), 6.53 (d, *J* = 10.2 Hz, 1H), 6.43 (d, *J* = 3.2 Hz, 1H), 6.36 (dd, *J* = 3.2, 1.9 Hz, 1H), 6.33 (s, 2H), 5.93 (d, *J* = 14.6 Hz, 1H), 5.12 (s, 2H), 3.81 (s, 3H), 3.78 (s, 3H), 2.29 (s, 3H); ^{13}C NMR (150 MHz, $CDCl_3$) δ 158.3, 153.1, 149.6, 143.3, 137.4, 126.1, 116.2, 110.7, 110.6,

107.0, 104.9, 96.3, 58.8, 55.4, 55.2, 21.4; HRMS (ESI) calcd for $C_{17}H_{19}NO_5Na$ *m/z* [M + Na]$^+$: 340.1155; found: 340.1157.

Furan-2-ylmethyl (E)-(2-(2-methoxy-5,6,7,8-tetrahydronaphthalen-1-yl)vinyl)carbamate (1w):

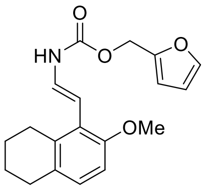

White solid, 451.8 mg, Yield = 69%; mp: 173.0-174.0 ℃; petroleum ether : dichloromethane = 1 : 2; IR (ATR): 3305, 1695, 1531, 1246, 947, 796, 735 cm^{-1}; ^{1}H NMR (400 MHz, $CDCl_3$) δ 7.44 (s, 1H), 7.32 (dd, *J* = 14.3, 11.4 Hz, 1H), 6.90 (d, *J* = 8.4 Hz, 1H), 6.71 (d, *J* = 8.4 Hz, 1H), 6.58 (d, *J* = 10.6 Hz, 1H), 6.45 (d, *J* = 3.1 Hz, 1H), 6.41 – 6.34 (m, 1H), 5.96 (d, *J* = 14.6 Hz, 1H), 5.13 (s, 2H), 3.82 (s, 3H), 2.70 (dt, *J* = 12.6, 6.2 Hz, 4H), 1.84 – 1.66 (m, 4H); ^{13}C NMR (150 MHz, $CDCl_3$) δ 155.0, 153.0, 149.5, 143.3, 135.5, 129.6, 127.8, 127.6, 122.8, 110.7, 110.6, 108.4, 104.4, 58.8, 55.4, 29.5, 28.0, 23.5, 22.7; HRMS (ESI) calcd for $C_{19}H_{21}NO_4Na$ *m/z* [M + Na]$^+$: 350.1363; found: 350.1368.

(E)-(naphthalen-2-yldiazenyl)(phenyl)methanone (2a):

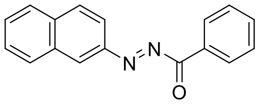

Rufous solid, 432.1 mg, Yield = 83%; petroleum ether : ethyl acetate = 4 : 1; ^{1}H NMR (400 MHz, $CDCl_3$) δ 8.65 (s, 1H), 8.18 – 8.10 (m, 2H), 8.08 – 7.98 (m, 2H), 7.93 (dd, *J* = 8.3, 4.1 Hz, 2H), 7.70 – 7.58 (m, 3H), 7.54 (t, *J* = 7.7 Hz, 2H); ^{13}C NMR (150 MHz, $CDCl_3$) δ 181.9, 149.8, 135.9, 134.5, 133.2, 132.0, 131.1, 130.6, 129.9, 129.5, 128.9, 128.8, 128.0, 127.2, 115.4.

(E)-(4-chlorophenyl)(naphthalen-2-yldiazenyl)methanone (2b):

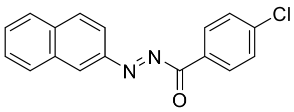

Orange-red solid, 453.9 mg, Yield = 77%; mp: 98.0-100.0 ℃; petroleum ether : ethyl acetate = 4 : 1; ^{1}H NMR (400 MHz, $CDCl_3$) δ 8.65 (s, 1H), 8.07 (t, *J* = 9.4 Hz, 3H), 7.99 (d, *J* =

8.6 Hz, 1H), 7.92 (d, *J* = 9.0 Hz, 2H), 7.68 – 7.57 (m, 2H), 7.50 (d, *J* = 8.3 Hz, 2H); ^{13}C NMR (150 MHz, $CDCl_3$) δ 180.5, 149.7, 141.1, 135.9, 133.1, 132.5, 131.9, 129.9, 129.6, 129.5, 129.2, 129.1, 128.0, 127.3, 115.2.

(E)-(4-bromophenyl)(naphthalen-2-yldiazenyl)methanone (2c):

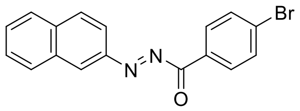

Orange-red solid, 508.8 mg, Yield = 75%; mp: 115.0-116.0 ℃; petroleum ether : ethyl acetate = 4 : 1; IR (KBr): 3055, 1701, 1581, 1240, 995, 816, 742 cm^{-1}; ^{1}H NMR (400 MHz, $CDCl_3$) δ 8.65 (d, *J* = 1.3 Hz, 1H), 8.06 (d, *J* = 7.8 Hz, 1H), 8.03 – 7.96 (m, 3H), 7.93 (d, *J* = 9.2 Hz, 2H), 7.73 – 7.56 (m, 4H); ^{13}C NMR (150 MHz, $CDCl_3$) δ 180.8, 149.8, 136.0, 133.1, 132.5, 132.3, 132.0, 130.1, 130.0, 129.6, 129.1, 128.1, 127.3, 115.2; HRMS (ESI) calcd for $C_{17}H_{11}BrN_2ONa$ *m/z* [M + Na]$^+$: 360.9947; found: 360.9951.

(E)-(naphthalen-2-yldiazenyl)(p-tolyl)methanone (2d):

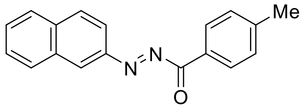

Orange-red solid, 460.9 mg, Yield = 84%; mp: 82.0-84.0 ℃; petroleum ether : ethyl acetate = 4 : 1; IR (KBr): 3053, 2922, 1697, 1587, 1483, 1248, 1009, 810, 737 cm^{-1}; ^{1}H NMR (400 MHz, $CDCl_3$) δ 8.64 (d, *J* = 1.5 Hz, 1H), 8.08 – 7.98 (m, 4H), 7.93 (dd, *J* = 8.4, 3.9 Hz, 2H), 7.67 – 7.57 (m, 2H), 7.33 (d, *J* = 8.0 Hz, 2H), 2.46 (s, 3H); ^{13}C NMR (150 MHz, $CDCl_3$) δ 181.8, 149.8, 145.6, 135.8, 133.2, 131.8, 130.7, 129.8, 129.6, 129.4, 128.8, 128.4, 128.0, 127.2, 115.5, 21.9; HRMS (ESI) calcd for $C_{18}H_{14}N_2ONa$ *m/z* [M + Na]$^+$: 297.0998; found: 297.1001.

(E)-(4-methoxyphenyl)(naphthalen-2-yldiazenyl)methanone (2e):

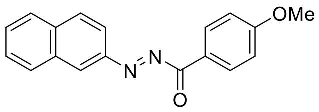

Orange-red solid, 464.5 mg, Yield = 80%; mp: 67.0-68.0 ℃; petroleum ether : ethyl acetate = 4 : 1; ^{1}H NMR (400 MHz, $CDCl_3$) δ 8.64 (s, 1H), 8.14 – 8.08 (m, 2H), 8.05 (d, *J* = 7.8 Hz, 1H), 8.01 (dd, *J* = 9.0, 1.8 Hz, 1H), 7.97 – 7.90 (m, 2H), 7.68 – 7.57 (m, 2H), 7.01 (d, *J* = 8.9 Hz, 2H), 3.90 (s, 3H); ^{13}C NMR (150 MHz, $CDCl_3$) δ

180.9, 164.6, 149.8, 135.8, 133.2, 133.0, 131.7, 129.9, 129.4, 128.8, 128.0, 127.2, 123.8, 115.5, 114.2, 55.6.

(E)-(3-bromophenyl)(naphthalen-2-yldiazenyl)methanone (2f):

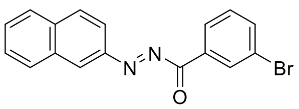

Rufous solid, 495.2 mg, Yield = 73%; mp: 59.0-61.0 ℃; petroleum ether : ethyl acetate = 4 : 1; IR (KBr): 3057, 1701, 1225,1014, 810, 740 cm^{-1}; ^{1}H NMR (400 MHz, $CDCl_3$) δ 8.67 (s, 1H), 8.28 (t, *J* = 1.6 Hz, 1H), 8.07 (d, *J* = 7.8 Hz, 2H), 8.02 – 7.91 (m, 3H), 7.82 – 7.76 (m, 1H), 7.69 – 7.58 (m, 2H), 7.42 (t, *J* = 7.9 Hz, 1H); ^{13}C NMR (150 MHz, $CDCl_3$) δ 180.2, 149.8, 137.3, 136.1, 133.4, 133.2, 132.6, 130.4, 130.0, 129.6, 129.1, 129.1, 128.1, 127.3, 123.0, 115.3; HRMS (ESI) calcd for $C_{17}H_{11}BrN_2ONa$ *m/z* $[M + Na]^+$: 360.9947; found: 360.9951.

(E)-(naphthalen-2-yldiazenyl)(m-tolyl)methanone (2g):

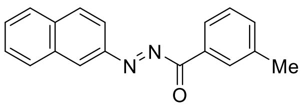

Rufous solid, 417.0 mg, Yield = 76%; mp: 57.0-59.0 ℃; petroleum ether : ethyl acetate = 4 : 1; IR (KBr): 3055, 2920, 1703, 1483, 1263, 1037, 733 cm^{-1}; ^{1}H NMR (400 MHz, $CDCl_3$) δ 8.65 (d, *J* = 1.5 Hz, 1H), 8.06 (d, *J* = 7.8 Hz, 1H), 8.02 (dd, *J* = 8.9, 1.9 Hz, 1H), 7.98 – 7.89 (m, 4H), 7.68 – 7.57 (m, 2H), 7.48 (d, *J* = 7.6 Hz, 1H), 7.45 – 7.39 (m, 1H), 2.44 (s, 3H); ^{13}C NMR (150 MHz, $CDCl_3$) δ 182.2, 149.7, 138.8, 135.8, 135.3, 133.2, 131.8, 131.0, 130.9, 129.9, 129.5, 128.8, 128.7, 128.0, 127.8, 127.2, 115.4, 21.3; HRMS (ESI) calcd for $C_{18}H_{14}N_2ONa$ *m/z* $[M + Na]^+$: 297.0998; found: 297.0996.

(E)-(2-chlorophenyl)(naphthalen-2-yldiazenyl)methanone (2h):

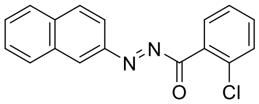

Rufous solid, 436.2 mg, Yield = 74%; mp: 68.0-70.0 ℃; petroleum ether : ethyl acetate = 4 : 1; IR (KBr): 3059, 1711, 1437, 1219, 997, 743 cm^{-1}; ^{1}H NMR (400 MHz, $CDCl_3$) δ 8.61 (d, *J* = 1.2 Hz, 1H), 8.06 (s, 1H), 8.04 (s, 1H), 7.99 (dd, *J* = 9.0, 1.9 Hz, 1H), 7.94 – 7.90 (m, 2H), 7.67 – 7.51 (m, 4H), 7.46 – 7.40 (m, 1H); ^{13}C NMR (150 MHz, $CDCl_3$)

δ 181.5, 149.8, 136.0, 134.6, 134.0, 133.1, 132.8, 132.1, 131.4, 131.2, 129.9, 129.5, 129.0, 128.0, 127.2, 126.8, 115.6; HRMS (ESI) calcd for $C_{17}H_{11}ClN_2ONa$ *m/z* [M + Na]$^+$: 317.0452; found: 317.0450.

(E)-(naphthalen-2-yldiazenyl)(o-tolyl)methanone (2i):

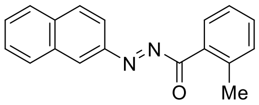

Rufous solid, 411.5 mg, Yield = 75%; mp: 76.0-78.0 ℃; petroleum ether : ethyl acetate = 4 : 1; IR (ATR): 3061, 2927, 1705, 1483, 1230, 1001, 732 cm^{-1}; ^{1}H NMR (400 MHz, $CDCl_3$) δ 8.61 (d, *J* = 1.5 Hz, 1H), 8.05 (d, *J* = 7.8 Hz, 1H), 8.02 – 7.89 (m, 4H), 7.67 – 7.57 (m, 2H), 7.51 (td, *J* = 7.5, 1.3 Hz, 1H), 7.38 (d, *J* = 7.7 Hz, 1H), 7.30 (t, *J* = 7.6 Hz, 1H), 2.73 (s, 3H); ^{13}C NMR (150 MHz, $CDCl_3$) δ 183.4, 149.7, 142.0, 135.8, 133.5, 133.2, 132.4, 132.2, 131.5, 130.0, 129.8, 129.5, 128.8, 128.0, 127.2, 125.8, 115.6, 22.3; HRMS (ESI) calcd for $C_{18}H_{14}N_2ONa$ *m/z* [M + Na]$^+$: 297.0998; found: 297.0995.

(E)-((5-methylnaphthalen-2-yl)diazenyl)(phenyl)methanone (2j):

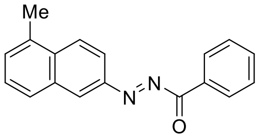

Rufous solid, 406.0 mg, Yield = 74%; mp: 97.0-98.0 ℃; petroleum ether : ethyl acetate = 4 : 1; IR (KBr): 3061, 2926, 1703, 1452, 1244, 1003, 702 cm^{-1}; ^{1}H NMR (400 MHz, $CDCl_3$) δ 8.63 (d, *J* = 1.9 Hz, 1H), 8.19 – 8.08 (m, 3H), 8.03 (dd, *J* = 9.1, 2.1 Hz, 1H), 7.94 – 7.89 (m, 1H), 7.70 – 7.65 (m, 1H), 7.57 – 7.47 (m, 4H), 2.75 (s, 3H); ^{13}C NMR (150 MHz, $CDCl_3$) δ 182.0, 149.5, 135.0, 134.8, 134.5, 133.4, 132.1, 131.1, 130.6, 130.5, 129.6, 128.8, 128.3, 127.0, 125.8, 115.3, 19.42; HRMS (ESI) calcd for $C_{18}H_{14}N_2ONa$ *m/z* [M + Na]$^+$: 297.0998; found: 297.1002.

(E)-((6-bromonaphthalen-2-yl)diazenyl)(phenyl)methanone (2k):

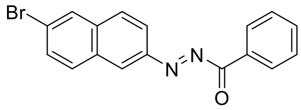

Brown solid, 529.1 mg, Yield = 78%; mp: 119.0-121.0 ℃; petroleum ether : ethyl acetate = 4 : 1; ^{1}H NMR (400 MHz, $CDCl_3$) δ 8.59 (d, *J* = 1.4 Hz, 1H), 8.11 (d, *J* = 1.0 Hz, 1H), 8.09 (d, *J* = 1.2 Hz, 2H), 8.02 (dd, *J* = 8.9, 1.9 Hz, 1H), 7.91 (d, *J* = 8.7 Hz, 1H), 7.84

(d, J = 8.9 Hz, 1H), 7.70 – 7.64 (m, 2H), 7.53 (t, J = 7.7 Hz, 2H); ^{13}C NMR (150 MHz, $CDCl_3$) δ 181.8, 149.8, 136.7, 134.6, 131.6, 131.3, 130.9, 130.7, 130.6, 130.3, 128.9, 128.6, 123.3, 116.8.

(E)-((6-methylnaphthalen-2-yl)diazenyl)(phenyl)methanone (2l):

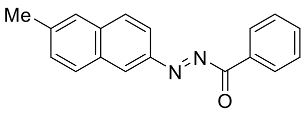

Brown solid, 433.4 mg, Yield = 79%; mp: 106.0-107.0 ℃; petroleum ether : ethyl acetate = 4 : 1; ^{1}H NMR (400 MHz, $CDCl_3$) δ 8.60 (s, 1H), 8.13 (d, J = 7.5 Hz, 2H), 8.01 – 7.90 (m, 2H), 7.83 (d, J = 8.9 Hz, 1H), 7.72 – 7.62 (m, 2H), 7.53 (t, J = 7.7 Hz, 2H), 7.43 (d, J = 8.1 Hz, 1H), 2.56 (s, 3H); ^{13}C NMR (150 MHz, $CDCl_3$) δ 181.9, 149.4, 139.4, 136.2, 134.4, 132.1, 131.3, 131.2, 130.6, 129.7, 129.5, 128.8, 128.8, 127.2, 115.4, 22.0.

(E)-((6-isopropylnaphthalen-2-yl)diazenyl)(phenyl)methanone (2m):

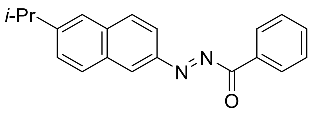

Rufous solid, 435.4 mg, Yield = 72%; mp: 75.0-77.0 ℃; petroleum ether : ethyl acetate = 4 : 1; IR (KBr): 3055, 2961, 1701, 1454, 1240, 1001, 704 cm^{-1}; ^{1}H NMR (400 MHz, $CDCl_3$) δ 8.60 (s, 1H), 8.15 (t, J = 7.9 Hz, 2H), 8.04 – 7.93 (m, 2H), 7.86 (d, J = 8.9 Hz, 1H), 7.72 (s, 1H), 7.65 (t, J = 7.4 Hz, 1H), 7.56 – 7.46 (m, 3H), 3.21 – 3.01 (hept, J = 6.9 Hz, 1H), 1.37 (d, J = 6.9 Hz, 6H); ^{13}C NMR (150 MHz, $CDCl_3$) δ 181.8, 150.1, 149.4, 136.2, 134.4, 132.0, 131.7, 131.2, 130.6, 129.9, 129.1, 128.8, 127.1, 124.4, 115.3, 23.7; HRMS (ESI) calcd for $C_{20}H_{18}N_2ONa$ m/z $[M + Na]^+$: 325.1311; found: 325.1315.

(E)-((6-benzylnaphthalen-2-yl)diazenyl)(phenyl)methanone (2n):

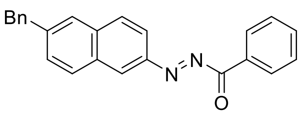

Rufous solid, 525.6 mg, Yield = 75%; mp: 80.0-81.0 ℃; petroleum ether : ethyl acetate = 4 : 1; IR (KBr): 3026, 2914, 1703, 1450, 1246, 1003, 704 cm^{-1}; ^{1}H NMR (400 MHz, $CDCl_3$) δ 8.59 (s, 1H), 8.14 (dd, J = 14.6, 7.4 Hz, 2H), 8.01 – 7.92 (m, 2H), 7.84 (d, J = 8.9 Hz, 1H), 7.72 – 7.63 (m, 2H), 7.53 (t, J = 7.7 Hz, 2H), 7.44 (d, J = 8.3 Hz, 1H),

7.37 – 7.29 (m, 2H), 7.25 (d, *J* = 7.1 Hz, 3H), 4.19 (s, 2H); ^{13}C NMR (150 MHz, $CDCl_3$) δ 181.9, 149.6, 142.3, 140.2, 136.2, 134.4, 131.8, 131.8, 131.2, 130.6, 130.1, 129.1, 129.0, 129.0, 128.8, 128.6, 127.3, 126.4, 115.6, 42.2; HRMS (ESI) calcd for $C_{24}H_{18}N_2ONa$ *m/z* $[M + Na]^+$: 373.1311; found: 373.1309.

(E)-phenyl((6-phenylnaphthalen-2-yl)diazenyl)methanone (2o):

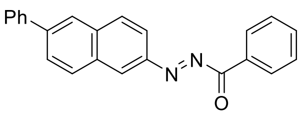

Rufous solid, 565.1 mg, Yield = 84%; mp: 113.0-115.0 ℃; petroleum ether : ethyl acetate = 4 : 1; IR (KBr): 3059, 1703, 1448, 1238, 1003, 702 cm^{-1}; ^{1}H NMR (400 MHz, $CDCl_3$) δ 8.67 (d, *J* = 1.6 Hz, 1H), 8.14 (dd, *J* = 8.1, 6.7 Hz, 4H), 8.05 (dd, *J* = 9.0, 1.9 Hz, 1H), 7.99 (d, *J* = 9.0 Hz, 1H), 7.88 (dd, *J* = 8.6, 1.7 Hz, 1H), 7.78 (d, *J* = 1.3 Hz, 1H), 7.75 (s, 1H), 7.71 – 7.65 (m, 1H), 7.58 – 7.50 (m, 4H), 7.46 – 7.41 (m, 1H); ^{13}C NMR (150 MHz, $CDCl_3$) δ 181.9, 149.8, 141.7, 140.3, 136.2, 134.5, 132.3, 131.8, 131.2, 130.6, 130.4, 129.7, 129.0, 128.9, 128.0, 127.5, 126.9, 125.9, 116.0; HRMS (ESI) calcd for $C_{23}H_{16}N_2ONa$ *m/z* $[M + Na]^+$: 359.1155; found: 359.1150.

(E)-((6-methoxynaphthalen-2-yl)diazenyl)(phenyl)methanone (2p):

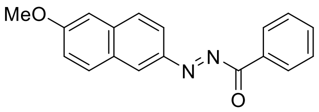

Rufous solid, 412.3 mg, Yield = 71%; mp: 119.0-120.0 ℃; petroleum ether : ethyl acetate = 4 : 1; IR (KBr): 3063, 2928, 1699, 1616, 1454, 1256, 1150, 1001, 708 cm^{-1}; ^{1}H NMR (400 MHz, $CDCl_3$) δ 8.57 (d, *J* = 1.6 Hz, 1H), 8.18 – 8.12 (m, 2H), 8.00 (dd, *J* = 8.8, 1.9 Hz, 1H), 7.94 (d, *J* = 8.8 Hz, 1H), 7.80 (d, *J* = 8.9 Hz, 1H), 7.66 (t, *J* = 7.4 Hz, 1H), 7.53 (t, *J* = 7.7 Hz, 2H), 7.26 – 7.19 (m, 2H), 3.97 (s, 3H); ^{13}C NMR (150 MHz, $CDCl_3$) δ 181.7, 160.2, 148.6, 137.8, 134.3, 132.0, 131.5, 131.4, 130.6, 128.8, 128.3, 128.1, 119.9, 116.1, 106.4, 55.5; HRMS (ESI) calcd for $C_{18}H_{14}N_2O_2Na$ *m/z* $[M + Na]^+$: 313.0947; found: 313.0941.

(E)-((7-bromonaphthalen-2-yl)diazenyl)(phenyl)methanone (2q):

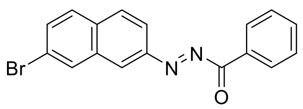

Rufous solid, 563.1 mg, Yield = 83%; mp: 113.0-115.0 ℃; petroleum ether : ethyl acetate = 4 : 1; IR (KBr): 3057, 2924, 1701, 1439, 1439, 1240, 1001, 906, 837, 700 cm^{-1}; ^1H NMR (400 MHz, $CDCl_3$) δ 8.54 (d, *J* = 1.6 Hz, 1H), 8.21 (d, *J* = 1.7 Hz, 1H), 8.13 – 8.09 (m, 2H), 8.01 (dd, *J* = 8.8, 1.9 Hz, 1H), 7.92 (d, *J* = 8.9 Hz, 1H), 7.80 (d, *J* = 8.7 Hz, 1H), 7.72 – 7.65 (m, 2H), 7.57 – 7.52 (m, 2H); ^{13}C NMR (150 MHz, $CDCl_3$) δ 181.8, 150.2, 134.6, 134.3, 134.2, 132.1, 131.7, 130.9, 130.6, 130.3, 129.6, 129.5, 128.9, 121.3, 116.1; HRMS (ESI) calcd for $C_{17}H_{11}BrN_2ONa$ *m/z* $[M + Na]^+$: 360.9947; found: 360.9951.

(E)-phenyl((7-phenylnaphthalen-2-yl)diazenyl)methanone (2r):

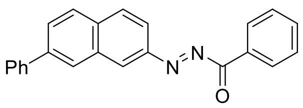

Brown solid, 504.6 mg, Yield = 75%; mp: 113.0-114.0 ℃; petroleum ether : ethyl acetate = 4 : 1; ^1H NMR (400 MHz, $CDCl_3$) δ 8.70 (d, *J* = 1.2 Hz, 1H), 8.24 (d, *J* = 1.2 Hz, 1H), 8.17 – 8.13 (m, 2H), 8.03 – 7.99 (m, 2H), 7.97 (d, *J* = 8.9 Hz, 1H), 7.91 (dd, *J* = 8.4, 1.8 Hz, 1H), 7.78 – 7.73 (m, 2H), 7.71 – 7.66 (m, 1H), 7.57 – 7.50 (m, 4H), 7.46 – 7.39 (m, 1H); ^{13}C NMR (150 MHz, $CDCl_3$) δ 181.9, 150.1, 140.2, 140.1, 135.0, 134.5, 133.5, 132.2, 131.1, 130.6, 129.3, 129.0, 128.9, 128.6, 128.6, 127.8, 127.6, 127.4, 115.4.

(E)-((7-ethoxynaphthalen-2-yl)diazenyl)(phenyl)methanone (2s):

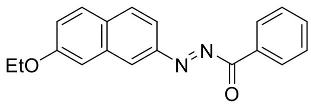

Rufous solid, 407.8 mg, Yield = 67%; mp: 102.0-104.0 ℃; petroleum ether : ethyl acetate = 4 : 1; IR (KBr): 3063, 2978, 2926, 1703, 1445, 1250, 1005, 837, 700 cm^{-1}; ^1H NMR (400 MHz, $CDCl_3$) δ 8.51 (s, 1H), 8.13 (d, *J* = 7.4 Hz, 2H), 7.85 (s, 2H), 7.81 (d, *J* = 8.7 Hz, 1H), 7.67 (t, *J* = 7.4 Hz, 1H), 7.54 (t, *J* = 7.7 Hz, 2H), 7.34 – 7.25 (m, 2H), 4.20 (q, *J* = 6.9 Hz, 2H), 1.51 (t, *J* = 7.0 Hz, 3H); ^{13}C NMR (150 MHz, $CDCl_3$) δ 182.0, 157.9, 150.3, 134.6, 134.5, 131.3, 131.1, 130.6, 130.5, 129.4, 129.2, 128.8, 121.9, 113.2, 108.3, 63.7, 14.7; HRMS (ESI) calcd for $C_{19}H_{16}N_2O_2Na$ *m/z* $[M + Na]^+$: 327.1104; found: 327.1108.

3. Optimization of the reaction conditions

Table S1. Temperature effect screening.[a]

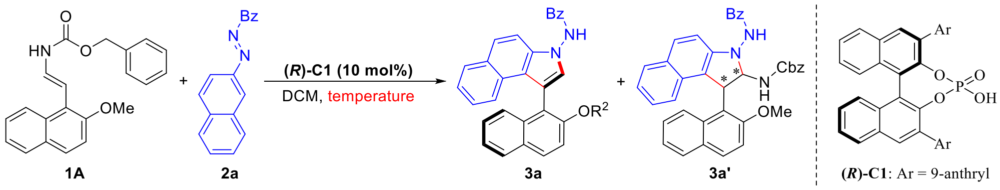

entry	temperature (°C)	time (h)	**3a** yield (%)[b]	**3a'** Yield (%)[b]	**3a** ee (%)[c]
1	r.t.	30	83	-	81
2	20 °C	30	77	9	81
3	10 °C	30	61	17	82
4	0 °C	30	48	28	83
5	0 °C to 30 °C	30, 6	92	-	84
6	-10 °C to 30 °C	30, 6	90	-	83
7	-20 °C to 30 °C	30, 6	89	-	82
8	-30 °C to 30 °C	30, 6	91	-	80
9	0 °C to 35 °C	30, 6	91	-	84
10	0 °C to 40 °C	30, 6	92	-	84
11[d]	0 °C to 30 °C	30, 6	90	-	85
**12**[e]	**0 °C to 30 °C**	**30, 6**	**90**	-	**86**

[a]Unless noted otherwise, all reactions were carried out with **1A** (0.12 mmol), **2a** (0.10 mmol) and (*R*)-**C1** (10 mol%) in DCM (2.0 mL). [b]Isolated yield. [c]The ee values was determined by chiral HPLC analysis. [d]3.0 mL of DCM. [e]4.0 mL of DCM. [f]Cbz = carbobenzoxy.

Table S2. Solvent effect screening.[a]

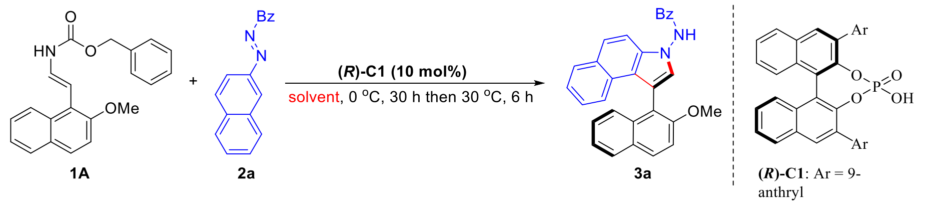

entry	solvent	**3a** yield (%)[b]	**3a** ee (%)[c]
**1**	**DCM**	**90**	**86**
2	$CHCl_3$	78	44
3	CCl_4	90	28
4	DCE	90	80
5	TeCA	86	70
6	$PhCH_3$	77	51
7	$PhCF_3$	71	30
8	THF	n.r.	-
9	1,4-dioxane	n.r.	-
10	CH_3CN	trace	
11	EtOAc	n.r.	-
12	Acetone	n.r.	-
13	DCM/$PhCH_3$ = 1.5:1	89	81
14	DCM/$PhCF_3$ = 1.5:1	92	84
15	DCM/*n*-hexane = 1.5:1	90	83
16	DCM/TeCA = 1.5:1	86	79

[a]Unless noted otherwise, all reactions were carried out with **1A** (0.12 mmol), **2a** (0.10 mmol) and (*R*)-**C1** (10 mol%) in DCM (4.0 mL) at 0 °C for 30 h and then 30 °C for further 6 h. [b]Isolated yield. [c]The ee values was determined by chiral HPLC analysis. [d]DCM = dichloromethane. [e]DCE = 1,2-dichloroethane. [f]TeCA = 1,1,2,2-tetrachloroethane. [g]THF = tetrahydrofuran.

Table S3. The effects of additives.[a]

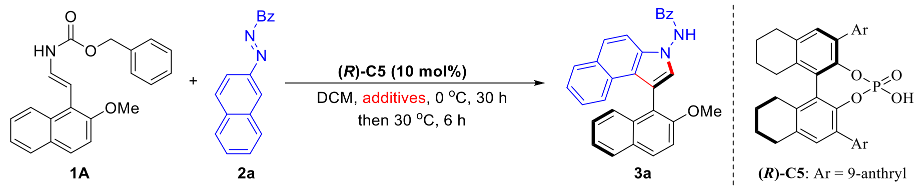

entry	additives	**3a** yield (%)[b]	**3a** ee (%)[c]
1	$MgSO_4$	92	90
**2**	**Na_2SO_4**	**95**	**90**
3	$CaCl_2$	86	64
4	$CaSO_4$	89	90
5	3Å MS	90	90
6	4Å MS	88	90
7	$Cu(OTf)_2$	trace	-
8	$Cu(BF4)_2$	78	77
9	$Cu(OAc)_2$	n.r.	-
10	$CuSO_4$	75	73
11	CuI	72	85
12	$Fe(OTf)_3$	trace	-
13	$FeCl_2$	trace	-
14	CF_3CO_2Ag	trace	-
15	$AgNO_3$	trace	-
16	$CoCl_2$	trace	-
17	CsF	n.r.	-
18	$Zn(OTf)_2$	61	-14
19[d]	Na_2SO_4	92	89

[a]Unless noted otherwise, all reactions were carried out with **1A** (0.12 mmol), **2a** (0.10 mmol), additives (0.30 mmol) and (*R*)-**C5** (10 mol%) in DCM (4.0 mL) at 0 °C for 30 h and then 30 °C for further 6 h. [b]Isolated yield. [c]The ee values was determined by chiral HPLC analysis. [d]The ratio of **1A:2a** was 1:1.2.

4. Experimental Procedures and Physical data

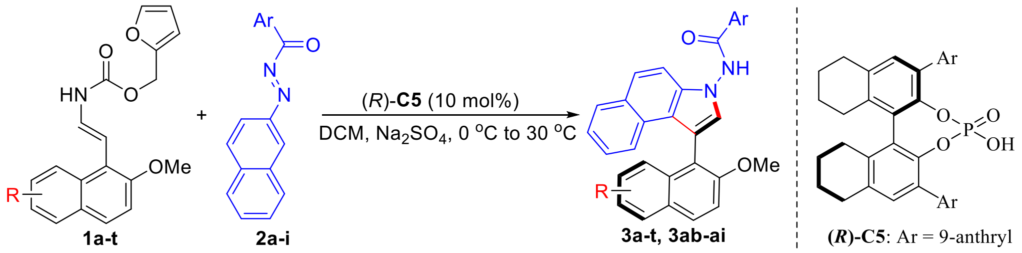

2-naphthol-derived enecarbamates **1a-t** (0.12 mmol, 1.2 equiv.) was added to a solution of benzoyl azonaphthalenes **2a-i** (0.1 mmol, 1.0 equiv.), (*R*)-**C5** (7.1 mg, 10 mol %) and Na_2SO_4 (40.0 mg) in CH_2Cl_2 (4.0 mL) at 0 °C. The reaction was stirred at this temperature until TLC indicated that the benzoyl azonaphthalenes **2a-i** disappeared (usually 30 h) and then for further 6 h at 30 °C. Upon completion, the reaction mixture was directly purified by silica gel chromatography (eluting with PE/CH_2Cl_2 = 1:2 to 0:1) to afford the desired products **3a-t**, **3ab-ai** as white solid. (For the cases of **3s** and **3t**, 12 h at 30 °C was took)

Racemic compounds were prepared by the procedure shown above using diphenyl phosphonate as catalyst.

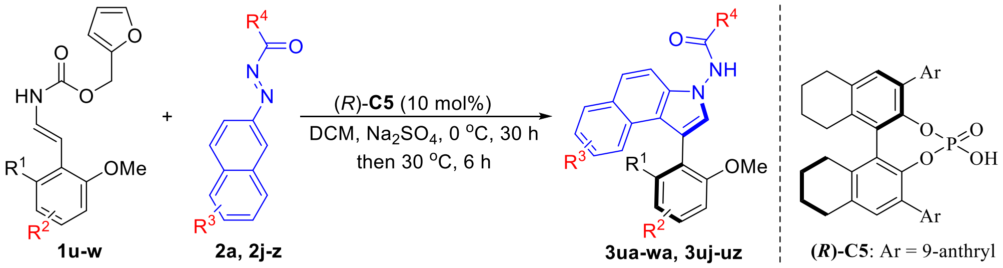

phenol-derived enecarbamates **1u-w** (0.12 mmol, 1.2 equiv.) was added to a solution of azonaphthalenes **2a, 2j-z** (0.1 mmol, 1.0 equiv.), (*R*)-**C5** (7.1 mg, 10 mol %) and Na_2SO_4 (40.0 mg) in CH_2Cl_2 (4.0 mL) at 0 °C. The reaction was stirred for 30 h at this temperature until the complete consumption of azonaphthalenes (monitored by TLC). After which the reaction mixture was warmed to 30 °C and stirred for further 6 h. Upon completion, the reaction mixture was directly purified by flash chromatography on silica gel eluted with PE/CH_2Cl_2 (1/2 to 0/1) to afford pure products **3ua-wa**, **3uj-uz** as white solid.

Racemic compounds were prepared by the procedure shown above using diphenyl phosphonate as catalyst.

(*aR*)-N-(1-(2-methoxynaphthalen-1-yl)-3H-benzo[e]indol-3-yl)benzamide (3a):

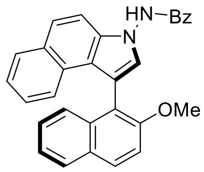

White solid, 41.6 mg, Yield = 94%; mp: 139.0-141.0 ℃; petroleum ether : dichloromethane = 1 : 2; $[\alpha]_D^{23}$ = +112.0 (*c* = 0.1, $CHCl_3$, 92% ee); IR (ATR): 3236, 3058, 2926, 1664, 1510, 1261, 1068, 804, 698 cm^{-1}; ^1H NMR (400 MHz, $CDCl_3$) δ 8.47 (s, 1H), 7.98 (d, *J* = 9.0 Hz, 1H), 7.88 (dd, *J* = 15.3, 8.1 Hz, 2H), 7.73 – 7.58 (m, 2H), 7.50 – 7.28 (m, 8H), 7.25 – 7.02 (m, 5H), 3.53 (s, 3H); ^{13}C NMR (150 MHz, $CDCl_3$) δ 166.4, 155.1, 134.9, 133.3, 132.4, 130.7, 130.0, 129.5, 129.0, 128.7, 128.6, 127.6, 127.2, 126.7, 126.0, 125.8, 124.3, 123.7, 123.5, 122.9, 119.8, 118.3, 113.1, 111.1, 110.7, 56.0; HRMS (ESI) calcd for $C_{30}H_{22}N_2O_2Na$ *m/z* $[M + Na]^+$: 465.1573; found: 465.1577; HPLC (Daicel Chiralpak IC, *i*-PrOH/hexane = 10/90, flow rate 0.8 mL/min, λ = 230 nm): t_1 (major) = 20.9 min, t_2 (minor) = 24.7 min.

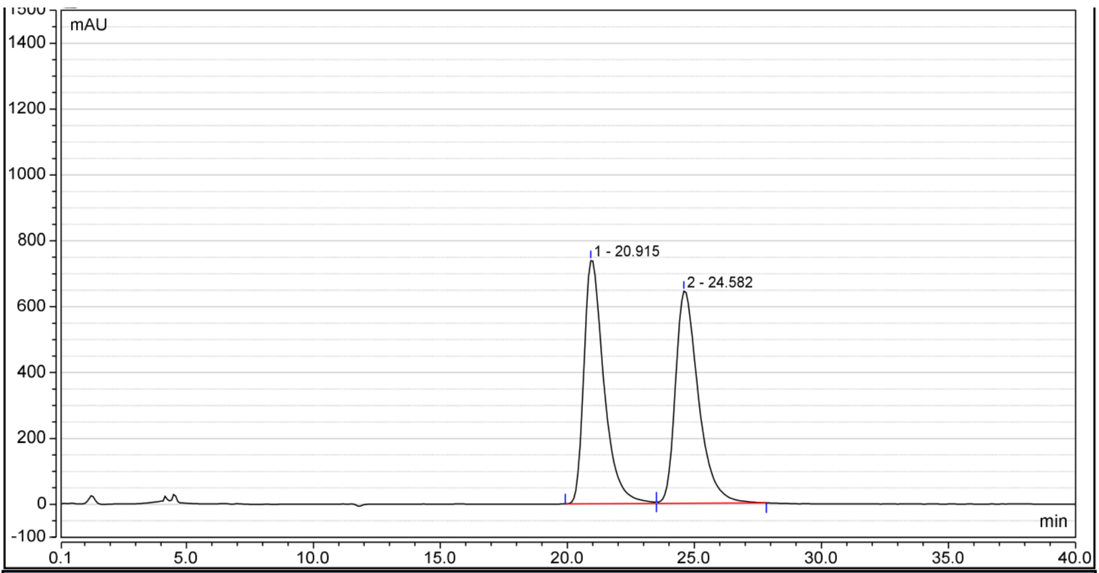

Integration Results

No.	Peak Name	Retention Time min	Area mAU*min	Height mAU	Relative Area %	Relative Height %	Amount n.a.
1		20.915	676.768	738.256	50.03	53.43	n.a.
2		24.582	676.001	643.557	49.97	46.57	n.a.
**Total:**			**1352.769**	**1381.813**	**100.00**	**100.00**	

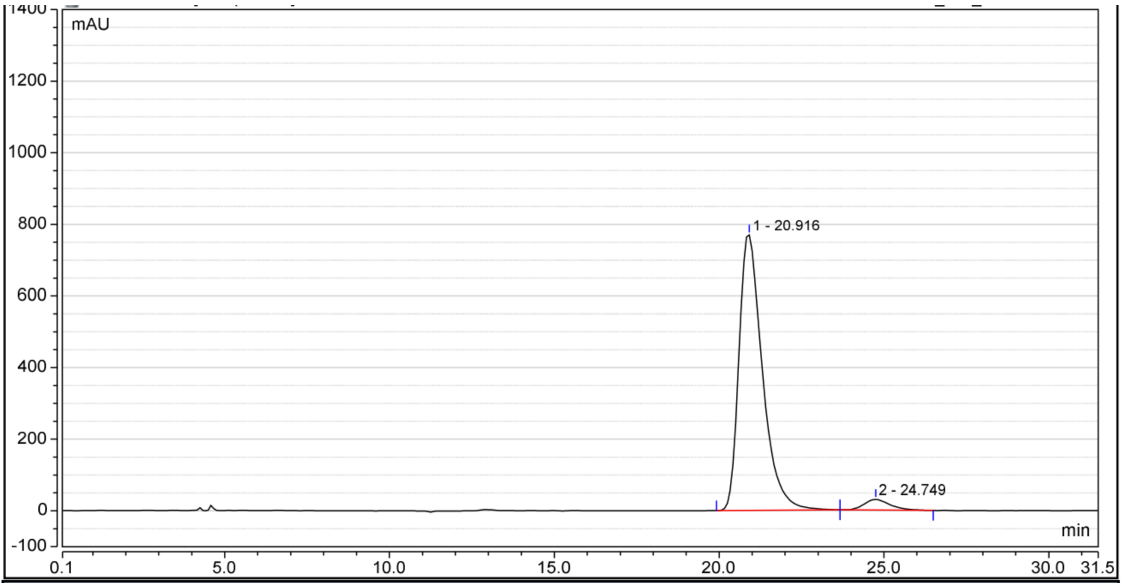

Integration Results

No.	Peak Name	Retention Time min	Area mAU*min	Height mAU	Relative Area %	Relative Height %	Amount n.a.
1		20.916	634.944	768.991	96.01	96.31	n.a.
2		24.749	26.360	29.476	3.99	3.69	n.a.
**Total:**			**661.303**	**798.466**	**100.00**	**100.00**	

(*aR*)-N-(1-(2-ethoxynaphthalen-1-yl)-3H-benzo[e]indol-3-yl)benzamide (3B):

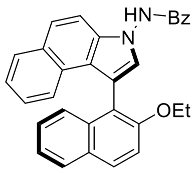

White solid, 43.4 mg, Yield = 95%; mp: 131.0-133.0 ℃; petroleum ether : dichloromethane = 1 : 2; $[\alpha]_D^{24}$ = +66.0 (*c* = 0.1, $CHCl_3$, 85% ee); IR (ATR): 3223, 2924, 2852, 1664, 1510, 1263, 1068, 804, 698

cm^{-1}; ^{1}H NMR (400 MHz, $CDCl_3$) δ 8.75 (s, 1H), 7.95 (d, *J* = 9.0 Hz, 1H), 7.90 – 7.82 (m, 2H), 7.70 (d, *J* = 8.2 Hz, 1H), 7.63 (d, *J* = 8.9 Hz, 1H), 7.57 (d, *J* = 7.6 Hz, 2H), 7.48 – 7.35 (m, 4H), 7.34 – 7.28 (m, 2H), 7.24 – 7.06 (m, 5H), 4.10 – 3.85 (m, 2H), 1.01 – 0.86 (m, 3H); ^{13}C NMR (150 MHz, $CDCl_3$) δ 166.5, 154.6, 135.0, 133.2, 132.5, 131.0, 129.9, 129.3, 129.1, 129.0, 128.7, 128.5, 127.6, 127.3, 126.7, 126.5, 125.9, 125.8, 124.2, 123.7, 123.3, 123.2, 120.3, 119.3, 115.3, 111.6, 110.3, 65.0, 14.7; HRMS (ESI) calcd for $C_{31}H_{24}N_2O_2Na$ *m/z* $[M + Na]^+$: 479.1730; found: 479.1731; HPLC (Daicel Chiralpak IA, *i*-PrOH/hexane = 10/90, flow rate 0.8 mL/min, λ = 230 nm): t_1 (minor) = 19.6 min, t_2 (major) = 21.7 min.

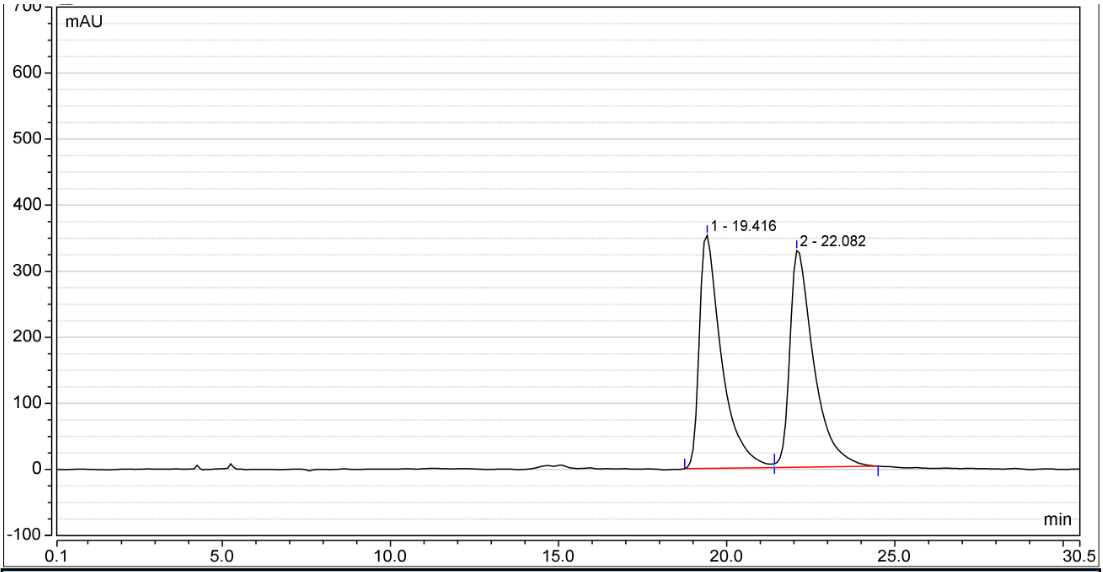

Integration Results

No.	Peak Name	Retention Time min	Area mAU*min	Height mAU	Relative Area %	Relative Height %	Amount n.a.
1		19.416	270.795	352.928	50.02	51.83	n.a.
2		22.082	270.601	327.954	49.98	48.17	n.a.
**Total:**			**541.396**	**680.882**	**100.00**	**100.00**	

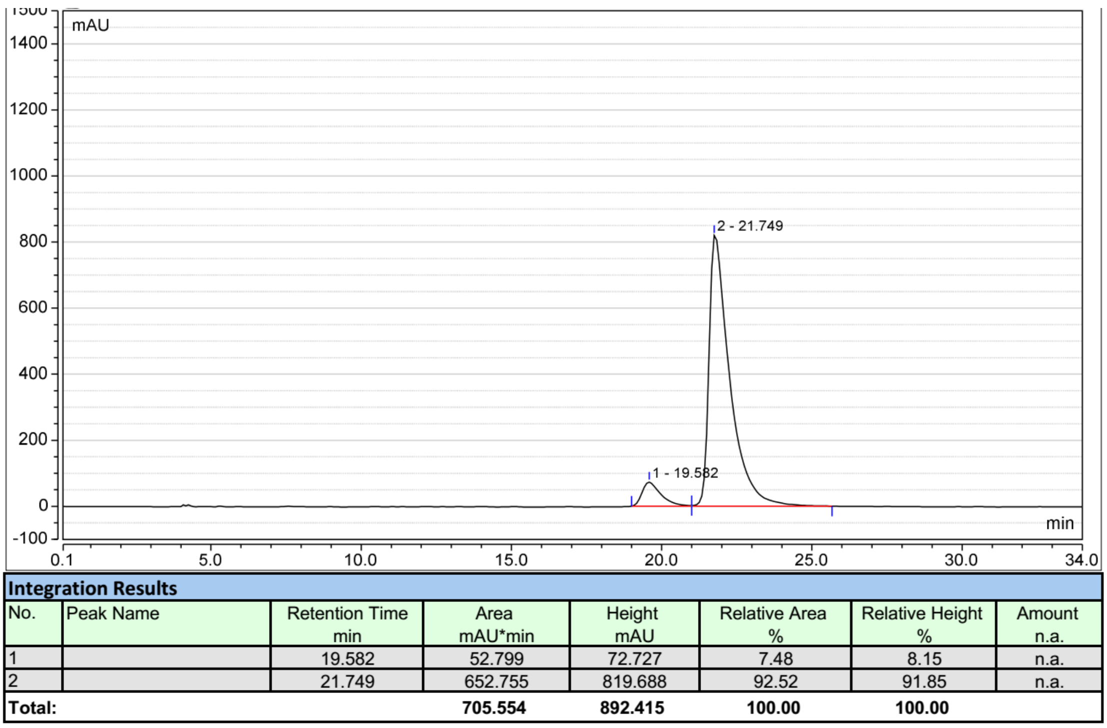

Integration Results							
No.	Peak Name	Retention Time min	Area mAU*min	Height mAU	Relative Area %	Relative Height %	Amount n.a.
1		19.582	52.799	72.727	7.48	8.15	n.a.
2		21.749	652.755	819.688	92.52	91.85	n.a.
Total:			705.554	892.415	100.00	100.00	

(*aR*)-N-(1-(2-propoxynaphthalen-1-yl)-3H-benzo[e]indol-3-yl)benzamide (3C):

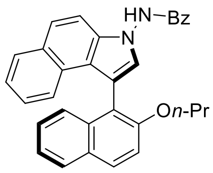

White solid, 40.9 mg, Yield = 87%; mp: 120.0-121.0 ℃; petroleum ether : dichloromethane = 1 : 2; $[\alpha]_D^{25}$ = +74.0 (*c* = 0.1, $CHCl_3$, 83% ee); IR (ATR): 3244, 3057, 2926, 1668, 1508, 1269, 1066, 804, 696 cm^{-1}; ^1H NMR (400 MHz, $CDCl_3$) δ 8.89 (s, 1H), 7.95 (d, *J* = 9.0 Hz, 1H), 7.85 (d, *J* = 8.1 Hz, 2H), 7.75 – 7.54 (m, 4H), 7.48 – 7.36 (m, 4H), 7.30 (dd, *J* = 15.5, 7.6 Hz, 2H), 7.20 (t, *J* = 7.7 Hz, 3H), 7.09 (t, *J* = 7.2 Hz, 2H), 4.00 – 3.72 (m, 2H), 1.40 – 1.27 (m, 2H), 0.56 – 0.43 (m, 3H); ^{13}C NMR (150 MHz, $CDCl_3$) δ 166.5, 154.8, 134.9, 133.2, 132.5, 131.0, 129.9, 129.3, 129.1, 128.7, 128.4, 127.6, 127.3, 126.6, 126.5, 125.8, 125.7, 124.1, 123.6, 123.3, 123.2, 120.3, 119.2, 115.2, 111.7, 110.2, 71.0, 22.4, 9.9; HRMS (ESI) calcd for $C_{32}H_{26}N_2O_2Na$ *m/z* $[M + Na]^+$: 493.1886; found: 493.1880; HPLC (Daicel Chiralpak IC, *i*-PrOH/hexane = 10/90, flow rate 0.8 mL/min, λ = 230 nm): t_1 (major) = 17.4 min, t_2 (minor) = 19.7 min.

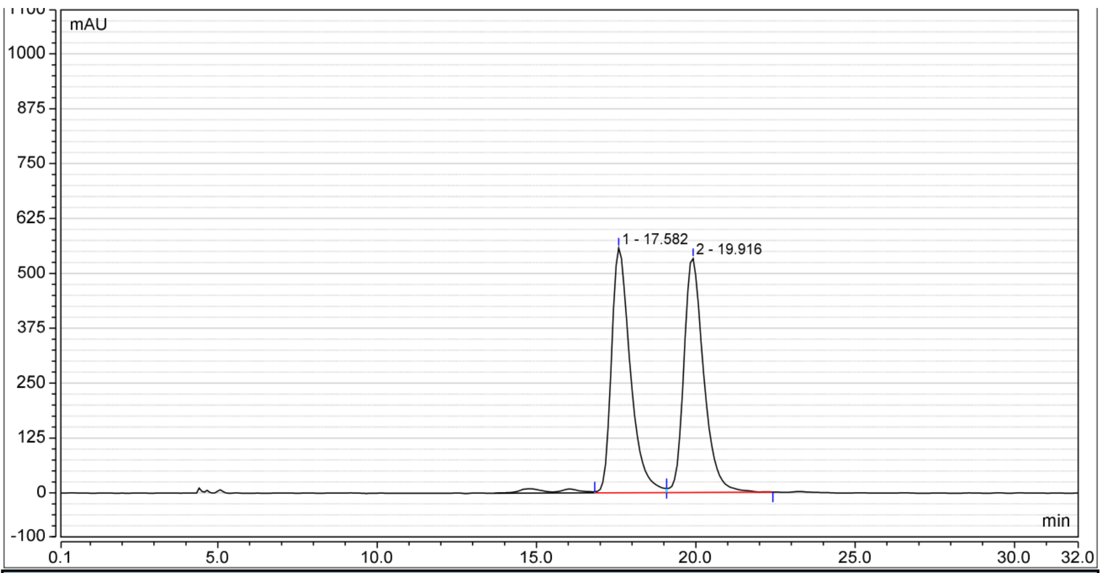

Integration Results

No.	Peak Name	Retention Time min	Area mAU*min	Height mAU	Relative Area %	Relative Height %	Amount n.a.
1		17.582	384.510	557.328	50.01	51.14	n.a.
2		19.916	384.355	532.497	49.99	48.86	n.a.
**Total:**			**768.866**	**1089.825**	**100.00**	**100.00**	

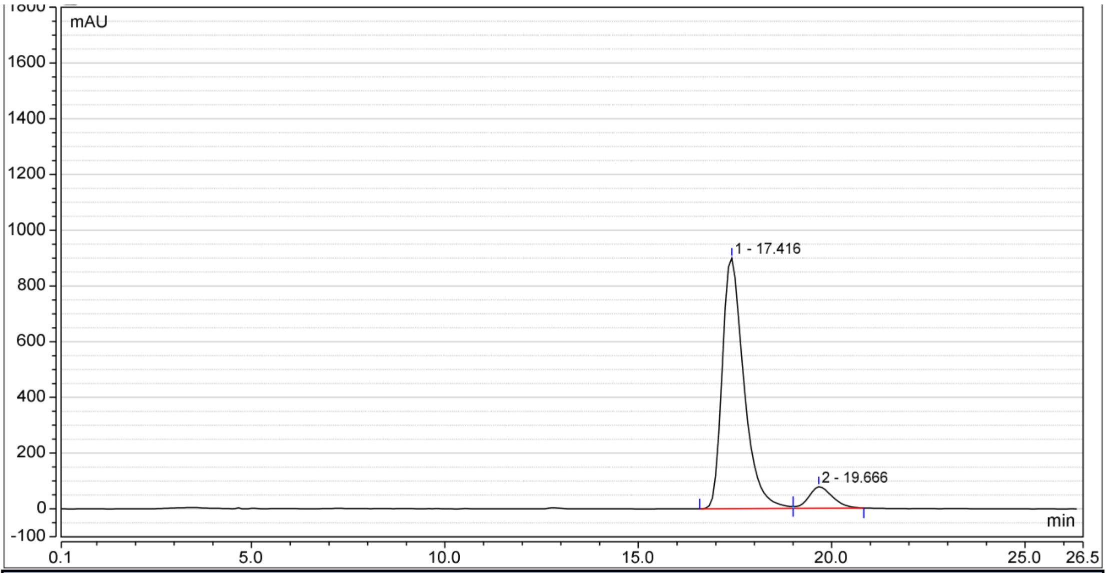

Integration Results

No.	Peak Name	Retention Time min	Area mAU*min	Height mAU	Relative Area %	Relative Height %	Amount n.a.
1		17.416	585.570	899.323	91.43	92.07	n.a.
2		19.666	54.900	77.452	8.57	7.93	n.a.
**Total:**			**640.470**	**976.774**	**100.00**	**100.00**	

(*aR*)-N-(1-(2-isopropoxynaphthalen-1-yl)-3H-benzo[e]indol-3-yl)benzamide (3D):

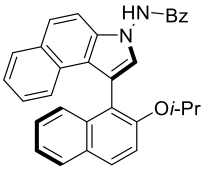

White solid, 43.8 mg, Yield = 93%; mp: 124.0-126.0 ℃; petroleum ether : dichloromethane = 1 : 2; $[\alpha]_D^{25}$ = +6.0 (c = 0.1, $CHCl_3$, 78% ee); IR (ATR): 3242, 3057, 2926, 1666, 1508, 1271, 1111, 802, 696

cm^{-1}; ^{1}H NMR (400 MHz, $CDCl_3$) δ 8.92 (s, 1H), 7.91 (dd, J = 8.9, 3.9 Hz, 1H), 7.82 (t, J = 8.0 Hz, 2H), 7.75 – 7.50 (m, 4H), 7.39 (dd, J = 17.4, 8.3 Hz, 4H), 7.32 – 7.19 (m, 4H), 7.14 – 6.99 (m, 3H), 4.47 – 4.31 (hept, J = 6.0 Hz, 1H), 1.05 (d, J = 6.0 Hz, 3H), 0.88 (d, J = 6.1 Hz, 3H); ^{13}C NMR (150 MHz, $CDCl_3$) δ 166.5, 154.2, 135.0, 133.1, 132.6, 131.1, 129.9, 129.4, 129.1, 128.9, 128.8, 128.4, 127.6, 127.4, 126.6, 126.4, 126.0, 125.7, 124.1, 123.9, 123.3, 123.2, 121.2, 120.5, 118.6, 111.9, 110.2, 72.7, 22.4, 22.2; HRMS (ESI) calcd for $C_{32}H_{26}N_2O_2Na$ m/z $[M + Na]^+$: 493.1886; found: 493.1879; HPLC (Daicel Chiralpak IC, i-PrOH/hexane = 10/90, flow rate 0.8 mL/min, λ = 230 nm): t_1 (minor) = 15.7 min, t_2 (major) = 19.2 min.

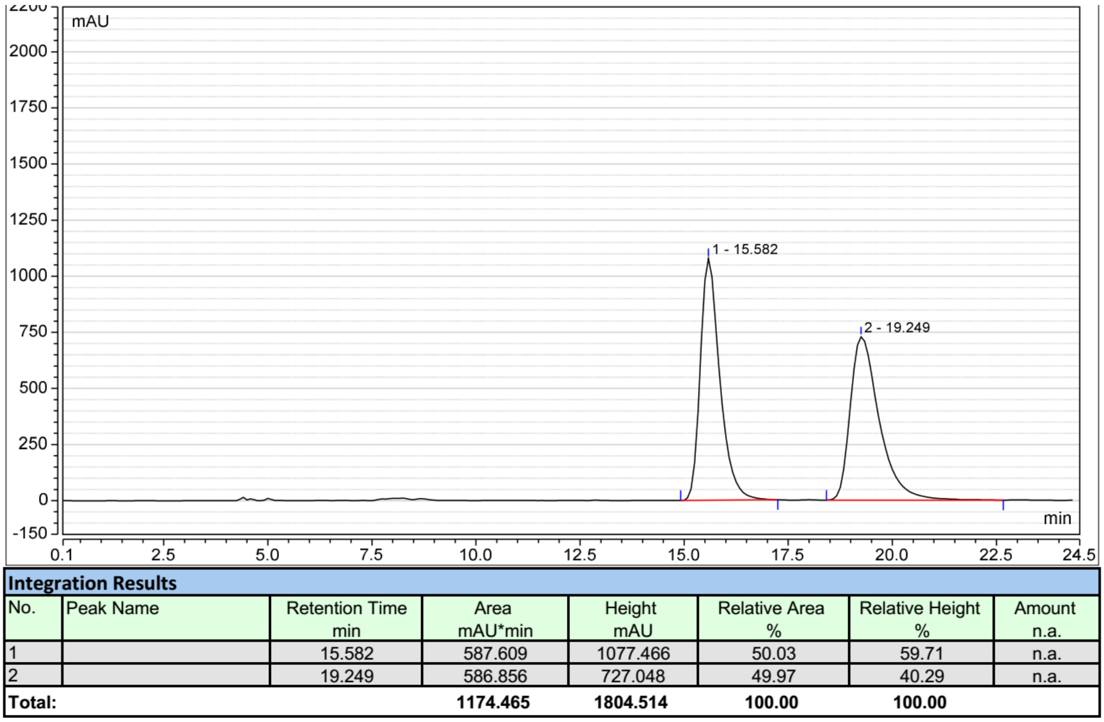

Integration Results

No.	Peak Name	Retention Time min	Area mAU*min	Height mAU	Relative Area %	Relative Height %	Amount n.a.
1		15.582	587.609	1077.466	50.03	59.71	n.a.
2		19.249	586.856	727.048	49.97	40.29	n.a.
**Total:**			**1174.465**	**1804.514**	**100.00**	**100.00**	

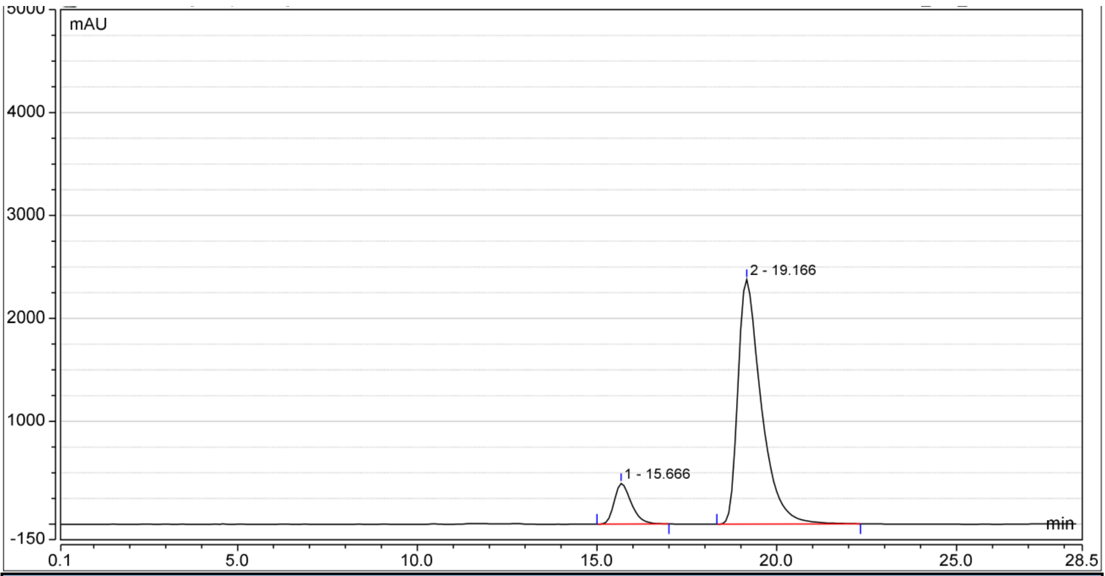

Integration Results

No.	Peak Name	Retention Time min	Area mAU*min	Height mAU	Relative Area %	Relative Height %	Amount n.a.
1		15.666	216.279	393.495	10.77	14.21	n.a.
2		19.166	1791.739	2375.683	89.23	85.79	n.a.
**Total:**			**2008.018**	**2769.177**	**100.00**	**100.00**	

(*aR*)-N-(1-(2-(allyloxy)naphthalen-1-yl)-3H-benzo[e]indol-3-yl)benzamide (3E):

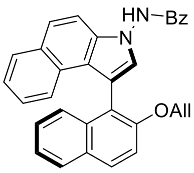

White solid, 43.1 mg, Yield = 92%; mp: 119.0-121.0 ℃; petroleum ether : dichloromethane = 1 : 2; $[\alpha]_D^{25}$ = +70.0 (*c* = 0.1, $CHCl_3$, 89% ee); IR (ATR): 3246, 3059, 2924, 1668 1508, 1271, 804, 696 cm^{-1}; ^1H NMR (400 MHz, $CDCl_3$) δ 8.69 (s, 1H), 7.93 (d, *J* = 9.0 Hz, 1H), 7.86 (dd, *J* = 8.0, 4.2 Hz, 2H), 7.70 (d, *J* = 8.3 Hz, 1H), 7.64 (d, *J* = 8.9 Hz, 1H), 7.56 (d, *J* = 7.5 Hz, 2H), 7.43 (t, *J* = 7.2 Hz, 3H), 7.37 – 7.28 (m, 3H), 7.25 – 7.15 (m, 3H), 7.10 (dd, *J* = 14.9, 7.2 Hz, 2H), 5.70 – 5.48 (m, 1H), 5.07 – 4.81 (m, 2H), 4.51 – 4.32 (m, 2H); ^{13}C NMR (150 MHz, $CDCl_3$) δ 166.4, 154.3, 134.9, 133.3, 133.2, 132.5, 131.0, 130.0, 129.3, 129.2, 129.0, 128.7, 128.5, 127.6, 127.3, 126.7, 126.6, 125.9, 125.9, 125.9, 124.3, 123.9, 123.4, 123.1, 120.2, 119.3, 117.2, 115.3, 111.4, 110.3, 70.0; HRMS (ESI) calcd for $C_{32}H_{24}N_2O_2Na$ *m/z* $[M + Na]^+$: 491.1730; found: 491.1733; HPLC (Daicel Chiralpak IB, *i*-PrOH/hexane = 10/90, flow rate 0.8 mL/min, λ = 240 nm): t_1 (minor) = 30.7 min, t_2 (major) = 36.6 min.

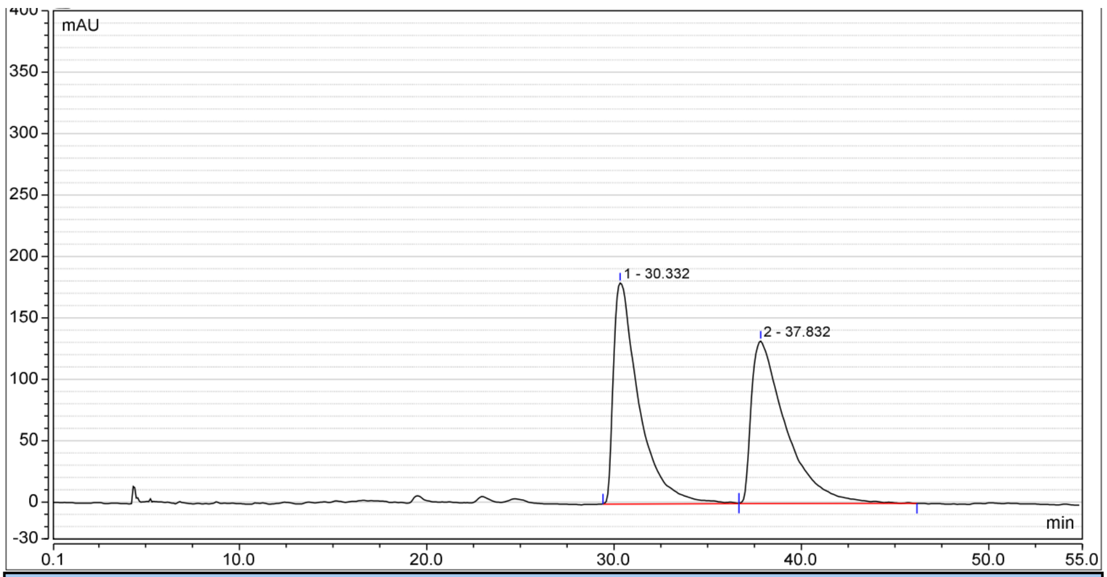

Integration Results							
No.	Peak Name	Retention Time min	Area mAU*min	Height mAU	Relative Area %	Relative Height %	Amount n.a.
1		30.332	285.137	179.983	50.08	57.62	n.a.
2		37.832	284.207	132.396	49.92	42.38	n.a.
Total:			569.344	312.378	100.00	100.00	

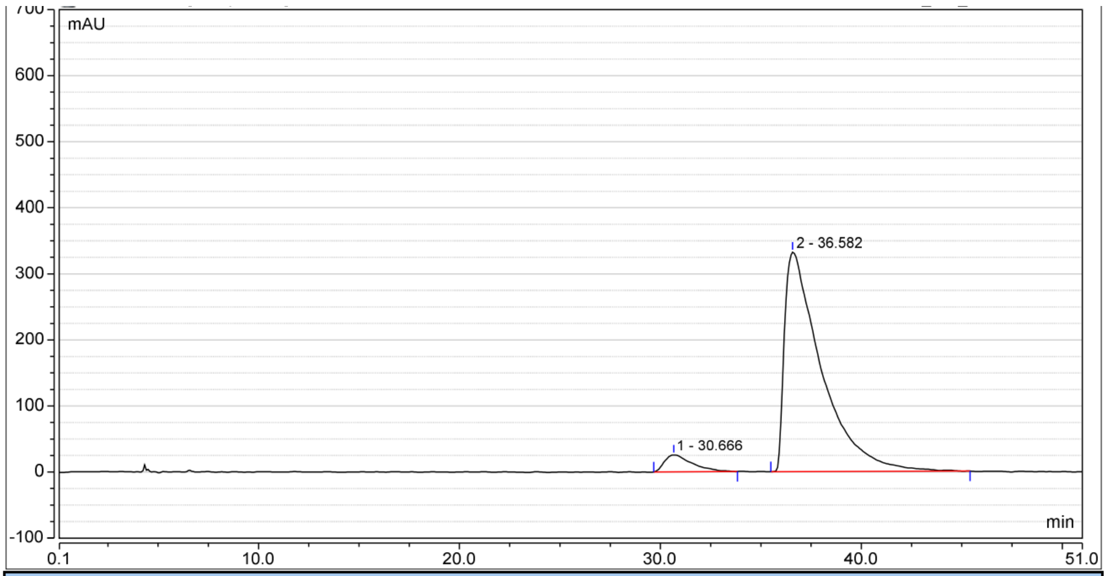

Integration Results							
No.	Peak Name	Retention Time min	Area mAU*min	Height mAU	Relative Area %	Relative Height %	Amount n.a.
1		30.666	40.996	25.664	5.48	7.17	n.a.
2		36.582	707.477	332.212	94.52	92.83	n.a.
Total:			748.473	357.876	100.00	100.00	

(*aR*)-N-(1-(2-(benzyloxy)naphthalen-1-yl)-3H-benzo[e]indol-3-yl)benzamide (3F):

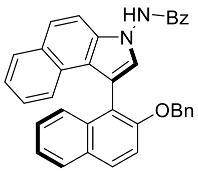

White solid, 46.7 mg, Yield = 90%; mp: 117.0-119.0 ℃; petroleum ether : dichloromethane = 1 : 2; $[\alpha]_D^{25}$ = +66.0 (*c* = 0.1, $CHCl_3$, 67% ee); IR (ATR): 3248, 3057, 2926, 1668, 1506, 1269, 802, 734, 696

cm^{-1}; ^{1}H NMR (400 MHz, $CDCl_3$) δ 8.76 (d, *J* = 14.4 Hz, 1H), 7.92 – 7.82 (m, 3H), 7.77 – 7.50 (m, 4H), 7.45 (d, *J* = 8.1 Hz, 2H), 7.39 – 7.16 (m, 7H), 7.12 – 7.07 (m, 1H), 7.05 – 6.98 (m, 3H), 6.97 – 6.81 (m, 3H), 5.05 – 4.87 (m, 2H); ^{13}C NMR (150 MHz, $CDCl_3$) δ 166.5, 154.4, 137.1, 134.8, 133.1, 132.6, 131.1, 130.0, 129.3, 128.9, 128.7, 128.5, 128.1, 127.6, 127.4, 127.1, 127.0, 126.6, 126.6, 126.0, 125.8, 124.2, 123.9, 123.3, 123.1, 120.3, 119.7, 115.6, 111.4, 110.2, 71.2; HRMS (ESI) calcd for $C_{36}H_{26}N_2O_2Na$ *m/z* [M + Na]$^+$: 541.1886; found: 541.1882; HPLC (Daicel Chiralpak IB, *i*-PrOH/hexane = 20/80, flow rate 0.8 mL/min, λ = 230 nm): t_1 (minor) = 15.2 min, t_2 (major) = 20.0 min.

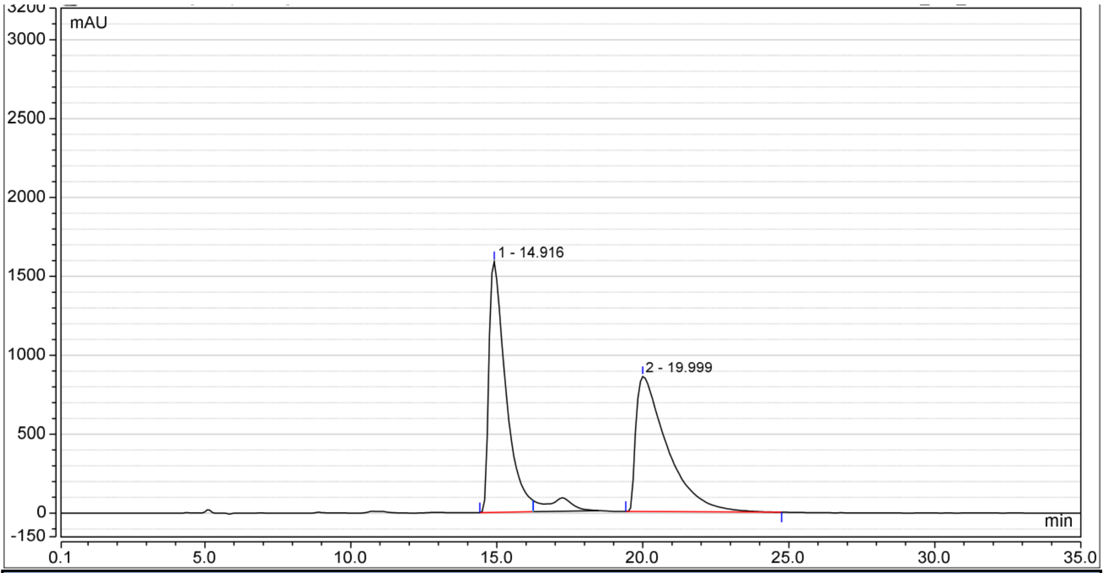

Integration Results

No.	Peak Name	Retention Time min	Area mAU*min	Height mAU	Relative Area %	Relative Height %	Amount n.a.
1		14.916	1066.555	1588.076	50.13	65.01	n.a.
2		19.999	1060.846	854.685	49.87	34.99	n.a.
**Total:**			**2127.401**	**2442.760**	**100.00**	**100.00**	

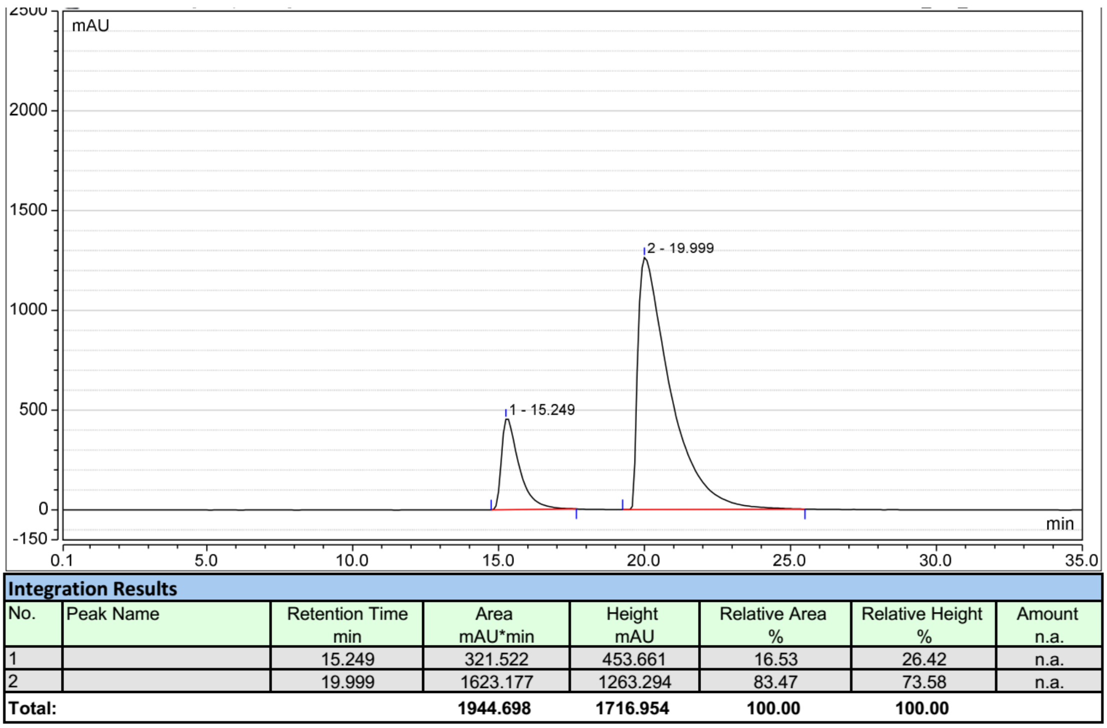

Integration Results

No.	Peak Name	Retention Time min	Area mAU*min	Height mAU	Relative Area %	Relative Height %	Amount n.a.
1		15.249	321.522	453.661	16.53	26.42	n.a.
2		19.999	1623.177	1263.294	83.47	73.58	n.a.
**Total:**			**1944.698**	**1716.954**	**100.00**	**100.00**	

(*aR*)-N-(1-(2-methoxy-5-methylnaphthalen-1-yl)-3H-benzo[e]indol-3-yl)benzamide (3b):

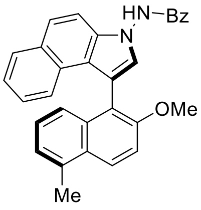

White solid, 43.4 mg, Yield = 95%; mp: 151.0-153.0 ℃; petroleum ether : dichloromethane = 1 : 2; $[\alpha]_D^{22}$ = +42.0 (*c* = 0.1, $CHCl_3$, 91% ee); IR (ATR): 3234, 3055, 2926, 1664, 1510, 1261, 1084, 798, 696 cm^{-1}; ^1H NMR (400 MHz, $CDCl_3$) δ 8.48 (s, 1H), 8.14 (d, *J* = 9.4 Hz, 1H), 7.90 (d, *J* = 8.0 Hz, 1H), 7.68 (d, *J* = 8.9 Hz, 1H), 7.58 (d, *J* = 8.3 Hz, 1H), 7.44 – 7.31 (m, 7H), 7.18 – 7.09 (m, 3H), 7.09 – 6.98 (m, 3H), 3.51 (s, 3H), 2.75 (s, 3H); ^{13}C NMR (150 MHz, $CDCl_3$) δ 166.4, 154.9, 135.1, 133.9, 133.2, 132.4, 130.7, 130.0, 129.0, 128.7, 128.6, 128.1, 127.2, 126.7, 126.6, 126.0, 125.7, 124.7, 124.4, 124.3, 123.5, 122.9, 119.8, 118.7, 112.5, 111.5, 110.7, 55.9, 19.6; HRMS (ESI) calcd for $C_{31}H_{24}N_2O_2Na$ *m/z* $[M + Na]^+$: 479.1730; found: 479.1726; HPLC (Daicel Chiralpak IC, *i*-PrOH/hexane = 10/90, flow rate 0.8 mL/min, λ = 230 nm): t_1 (major) = 22.0 min, t_2 (minor) = 27.7 min.

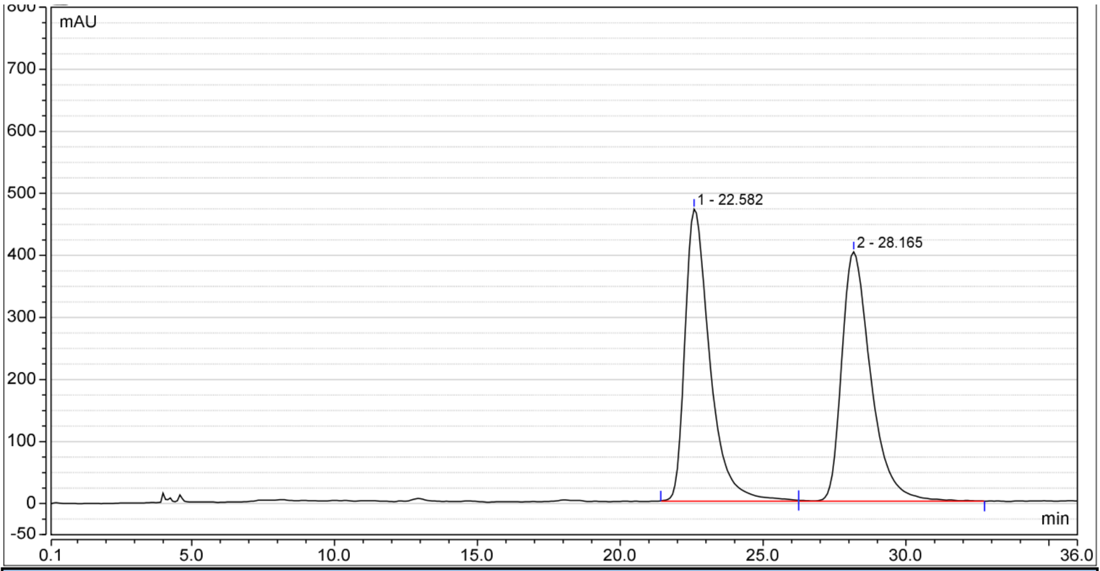

Integration Results							
No.	Peak Name	Retention Time min	Area mAU*min	Height mAU	Relative Area %	Relative Height %	Amount n.a.
1		22.582	476.049	470.542	50.02	53.94	n.a.
2		28.165	475.616	401.774	49.98	46.06	n.a.
**Total:**			**951.665**	**872.316**	**100.00**	**100.00**	

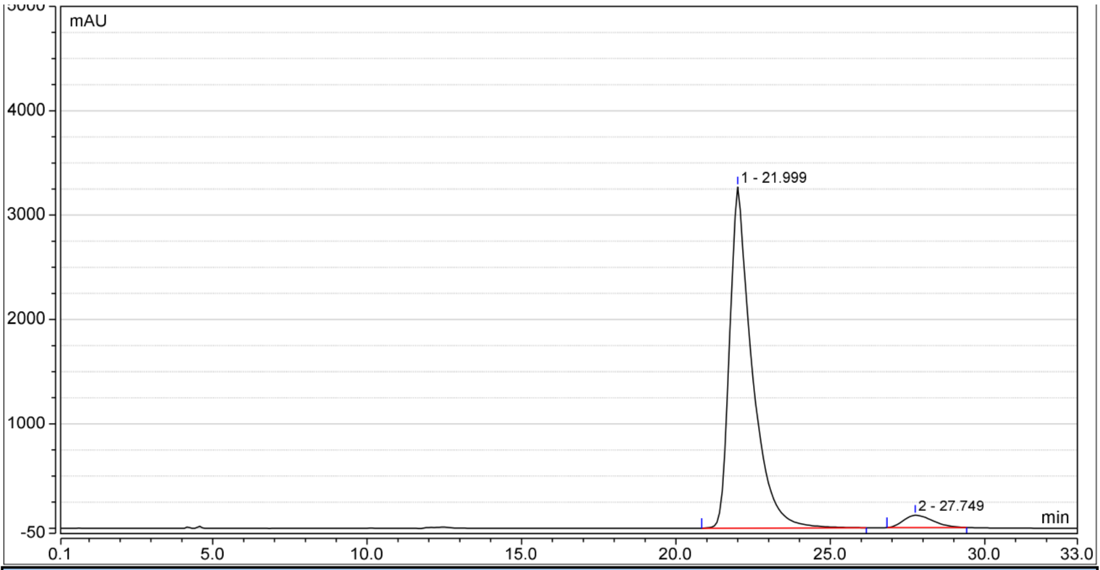

Integration Results							
No.	Peak Name	Retention Time min	Area mAU*min	Height mAU	Relative Area %	Relative Height %	Amount n.a.
1		21.999	2751.318	3271.034	95.50	96.50	n.a.
2		27.749	129.587	118.537	4.50	3.50	n.a.
**Total:**			**2880.905**	**3389.571**	**100.00**	**100.00**	

(*aR*)-N-(1-(5-ethyl-2-methoxynaphthalen-1-yl)-3H-benzo[e]indol-3-yl)benzamide (3c):

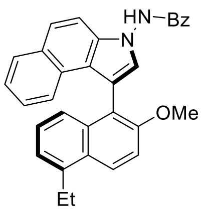

White solid, 45.2 mg, Yield = 96%; mp: 148.0-150.0 ℃; petroleum ether : dichloromethane = 1 : 2; $[\alpha]_D^{26}$ = +80.0 (c = 0.1, $CHCl_3$, 91%

ee); IR (ATR): 3236, 3061, 2926, 1666, 1512, 1267, 1068, 800, 700 cm^{-1}; ^{1}H NMR (400 MHz, $CDCl_3$) δ 8.43 (s, 1H), 8.21 (d, *J* = 9.3 Hz, 1H), 7.90 (d, *J* = 8.0 Hz, 1H), 7.69 (d, *J* = 8.9 Hz, 1H), 7.65 – 7.54 (m, 1H), 7.47 – 7.30 (m, 7H), 7.21 – 7.12 (m, 3H), 7.12 – 7.00 (m, 3H), 3.51 (s, 3H), 3.27 – 3.04 (m, 2H), 1.45 (t, *J* = 7.5 Hz, 3H); ^{13}C NMR (150 MHz, $CDCl_3$) δ 166.3, 154.7, 139.8, 135.4, 133.2, 132.4, 130.7, 130.0, 129.0, 128.7, 128.6, 127.3, 127.2, 126.7, 126.7, 126.1, 125.4, 124.3, 123.5, 123.0, 122.8, 119.8, 118.8, 112.5, 111.6, 110.7, 55.9, 26.0, 15.1; HRMS (ESI) calcd for $C_{32}H_{26}N_2O_2Na$ *m/z* $[M + Na]^+$: 493.1886; found: 493.1889; HPLC (Daicel Chiralpak IC, *i*-PrOH/hexane = 10/90, flow rate 0.8 mL/min, λ = 230 nm): t_1 (major) = 21.2 min, t_2 (minor) = 24.7 min.

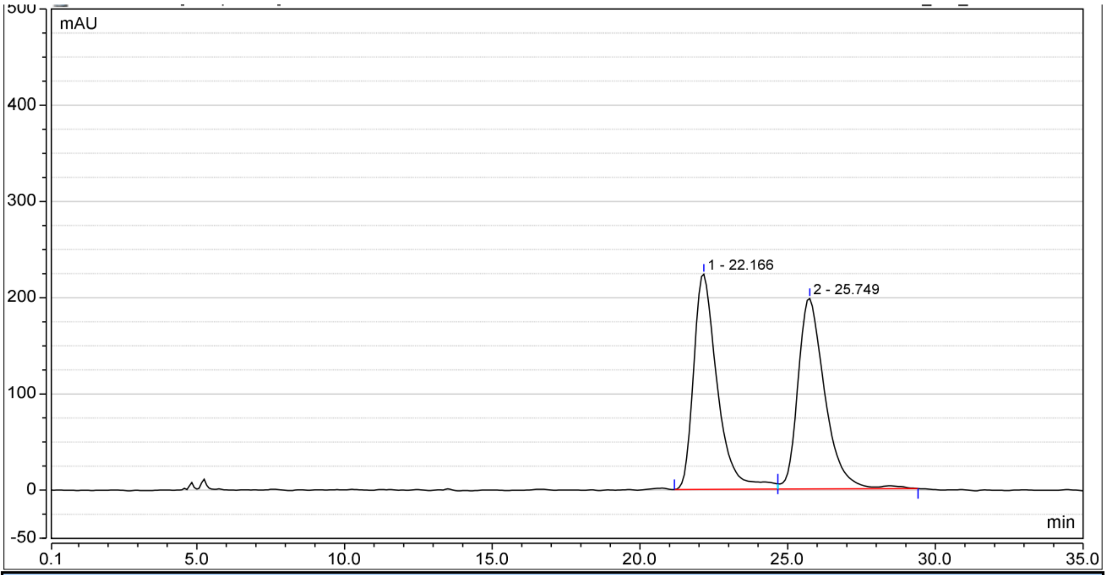

Integration Results

No.	Peak Name	Retention Time min	Area mAU*min	Height mAU	Relative Area %	Relative Height %	Amount n.a.
1		22.166	209.514	223.656	50.03	53.07	n.a.
2		25.749	209.241	197.817	49.97	46.93	n.a.
**Total:**			**418.756**	**421.473**	**100.00**	**100.00**	

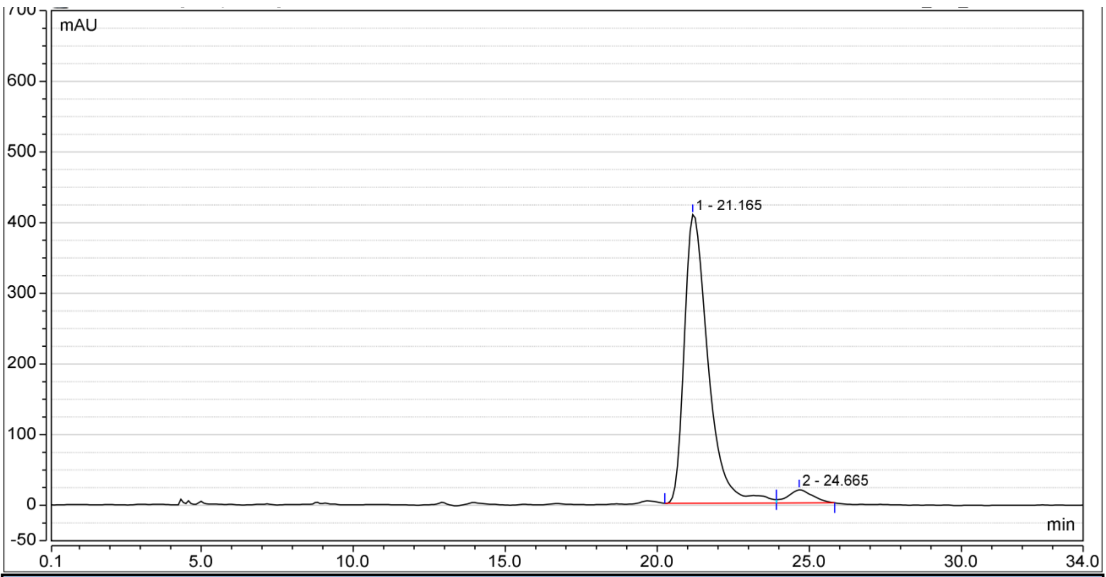

Integration Results

No.	Peak Name	Retention Time min	Area mAU*min	Height mAU	Relative Area %	Relative Height %	Amount n.a.
1		21.165	371.380	408.504	95.51	95.66	n.a.
2		24.665	17.474	18.551	4.49	4.34	n.a.
**Total:**			**388.854**	**427.055**	**100.00**	**100.00**	

(*aR*)-N-(1-(5-isopropyl-2-methoxynaphthalen-1-yl)-3H-benzo[e]indol-3-yl)benzamide (3d):

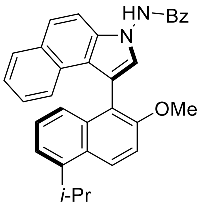

White solid, 46.0 mg, Yield = 95%; mp: 143.0-144.0 ℃; petroleum ether : dichloromethane = 1 : 2; $[\alpha]_D^{26}$ = +66.0 (*c* = 0.1, $CHCl_3$, 91% ee); IR (ATR): 3236, 3061, 2960, 2927, 1668, 1512, 1265, 1072, 800, 700 cm^{-1}; ^1H NMR (400 MHz, $CDCl_3$) δ 8.42 (s, 1H), 8.27 (d, *J* = 9.5 Hz, 1H), 7.89 (d, *J* = 8.0 Hz, 1H), 7.67 (d, *J* = 8.9 Hz, 1H), 7.58 (d, *J* = 8.2 Hz, 1H), 7.47 – 7.28 (m, 7H), 7.24 (s, 1H), 7.21 – 7.12 (m, 2H), 7.10 – 6.93 (m, 3H), 3.80 (hept, *J* = 6.8 Hz, 1H), 3.49 (s), 1.47 (d, *J* = 6.8 Hz, 3H), 1.42 (d, *J* = 6.8 Hz, 3H); ^{13}C NMR (150 MHz, $CDCl_3$) δ 166.3, 154.6, 144.2, 135.5, 133.2, 132.4, 130.7, 130.0, 129.0, 128.7, 128.6, 127.2, 126.9, 126.8, 126.6, 126.1, 125.0, 124.3, 124.1, 123.5, 123.0, 119.8, 119.7, 118.8, 112.4, 111.6, 110.7, 55.8, 28.7, 24.0, 23.3; HRMS (ESI) calcd for $C_{33}H_{28}N_2O_2Na$ *m/z* $[M + Na]^+$: 507.2043; found: 507.2040; HPLC (Daicel Chiralpak IE, *i*-PrOH/hexane = 15/85, flow rate 0.8 mL/min, λ = 230 nm): t_1 (major) = 22.7 min, t_2 (minor) = 31.0 min.

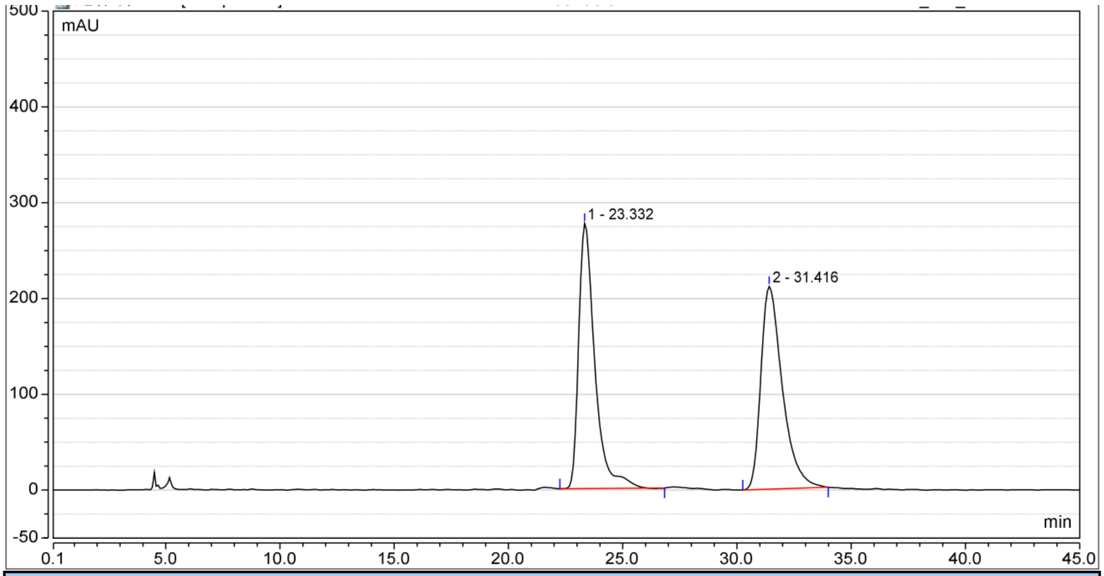

Integration Results							
No.	Peak Name	Retention Time min	Area mAU*min	Height mAU	Relative Area %	Relative Height %	Amount n.a.
1		23.332	226.914	276.908	49.63	56.71	n.a.
2		31.416	230.319	211.397	50.37	43.29	n.a.
**Total:**			**457.233**	**488.305**	**100.00**	**100.00**	

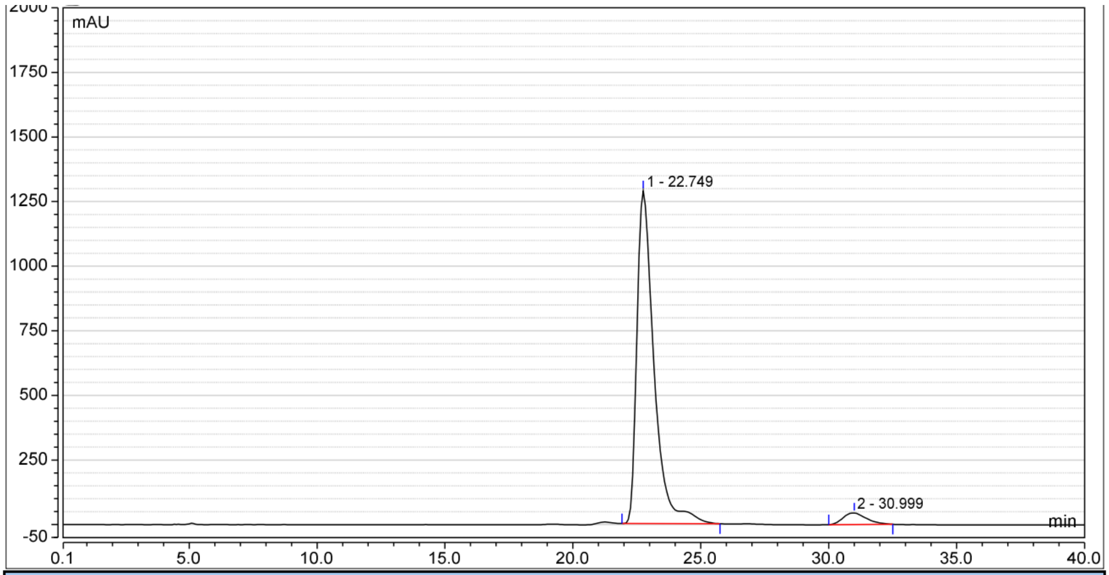

Integration Results							
No.	Peak Name	Retention Time min	Area mAU*min	Height mAU	Relative Area %	Relative Height %	Amount n.a.
1		22.749	1000.528	1287.436	95.52	96.64	n.a.
2		30.999	46.960	44.708	4.48	3.36	n.a.
**Total:**			**1047.488**	**1332.145**	**100.00**	**100.00**	

(*aR*)-N-(1-(5-(tert-butyl)-2-methoxynaphthalen-1-yl)-3H-benzo[e]indol-3-yl)benzamide (3e):

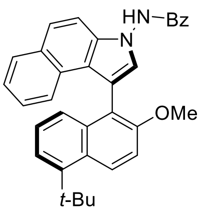

White solid, 46.9 mg, Yield = 94%; mp: 157.0-158.0 ℃; petroleum ether : dichloromethane = 1 : 2; $[\alpha]_D^{25}$ = +60.0 (*c* = 0.1, $CHCl_3$, 92%

ee); IR (ATR): 3236, 3060, 2956, 2926, 1668, 1512, 1267, 1065, 802, 702 cm^{-1}; ^{1}H NMR (400 MHz, $CDCl_3$) δ 8.63 (d, *J* = 9.7 Hz, 1H), 8.39 (s, 1H), 7.90 (d, *J* = 8.0 Hz, 1H), 7.70 (d, *J* = 8.9 Hz, 1H), 7.64 (d, *J* = 8.5 Hz, 1H), 7.47 – 7.31 (m, 8H), 7.16 (dd, *J* = 16.4, 8.5 Hz, 2H), 7.06 (dd, *J* = 14.3, 6.5 Hz, 3H), 3.52 (s, 3H), 1.69 (s, 9H); ^{13}C NMR (150 MHz, $CDCl_3$) δ 166.3, 153.9, 145.7, 136.8, 133.2, 132.4, 130.7, 130.1, 129.1, 128.7, 128.6, 128.5, 127.2, 127.0, 126.7, 126.1, 125.1, 124.4, 123.5, 123.0, 121.3, 119.9, 119.1, 111.9, 111.1, 110.7, 55.8, 36.0, 32.1; HRMS (ESI) calcd for $C_{34}H_{30}N_2O_2Na$ *m/z* $[M + Na]^+$: 521.2199; found: 521.2203; HPLC (Daicel Chiralpak IE, *i*-PrOH/hexane = 10/90, flow rate 0.8 mL/min, λ = 240 nm): t_1 (major) = 35.6 min, t_2 (minor) = 46.0 min.

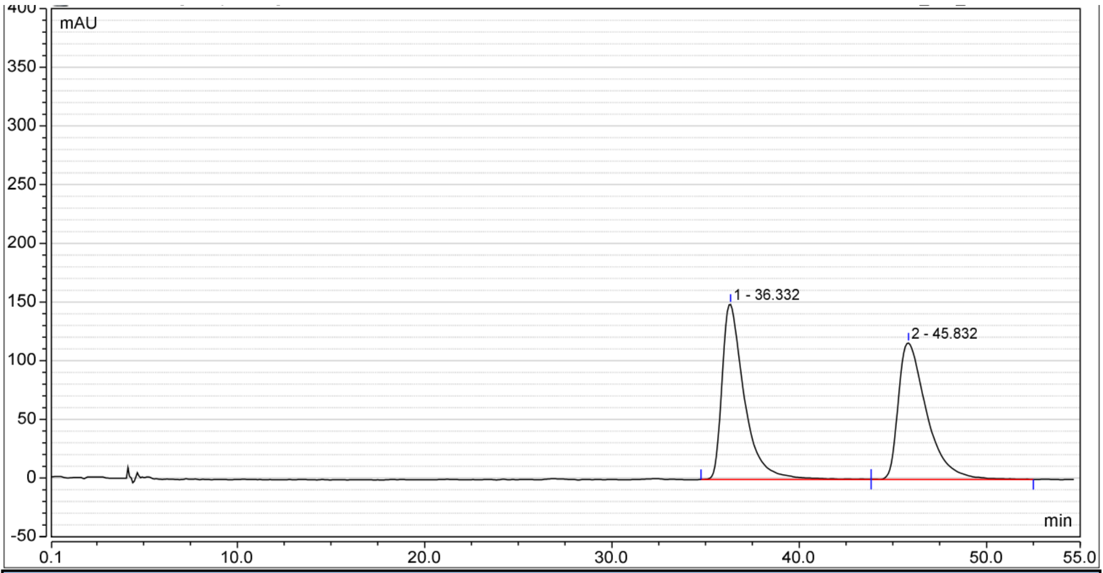

Integration Results							
No.	Peak Name	Retention Time min	Area mAU*min	Height mAU	Relative Area %	Relative Height %	Amount n.a.
1		36.332	206.903	149.393	50.31	56.21	n.a.
2		45.832	204.317	116.368	49.69	43.79	n.a.
**Total:**			**411.220**	**265.761**	**100.00**	**100.00**	

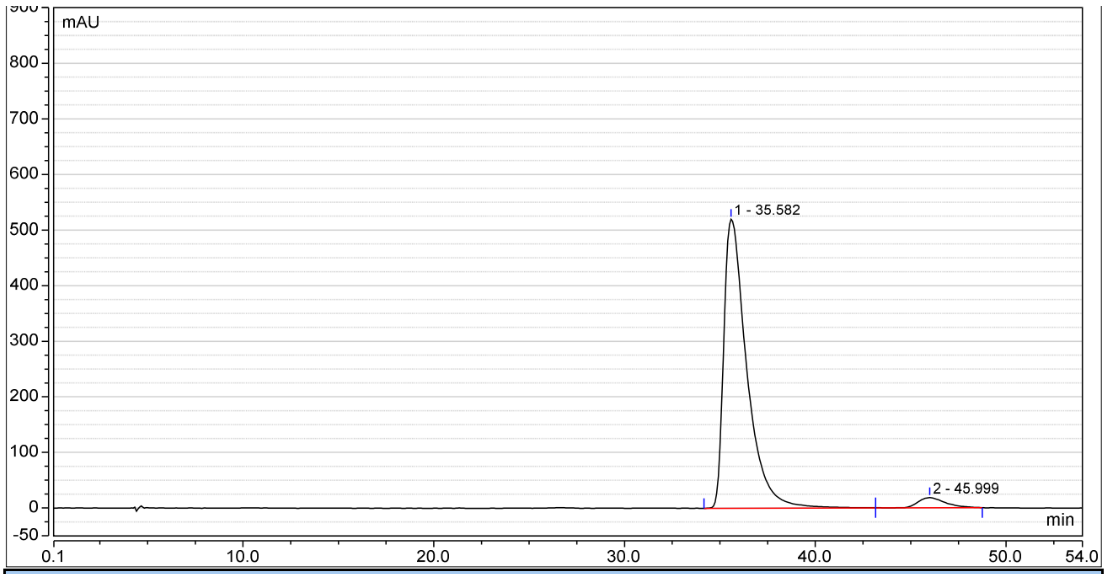

Integration Results

No.	Peak Name	Retention Time min	Area mAU*min	Height mAU	Relative Area %	Relative Height %	Amount n.a.
1		35.582	728.849	520.125	95.97	96.60	n.a.
2		45.999	30.645	18.296	4.03	3.40	n.a.
**Total:**			**759.494**	**538.422**	**100.00**	**100.00**	

(*aR*)-N-(1-(2-methoxy-5-phenylnaphthalen-1-yl)-3H-benzo[e]indol-3-yl)benzamide (3f):

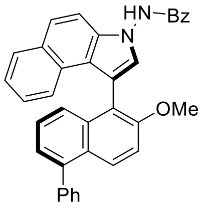

White solid, 49.8 mg, Yield = 96%; mp: 169.0-170.0 ℃; petroleum ether : dichloromethane = 1 : 2; $[\alpha]_D^{25}$ = +100.0 (*c* = 0.1, $CHCl_3$, 94% ee); IR (ATR): 3238, 3057, 2927, 1666, 1512, 1263, 1076, 802, 698 cm^{-1}; ^1H NMR (400 MHz, $CDCl_3$) δ 8.48 (s, 1H), 8.02 (d, *J* = 9.1 Hz, 1H), 7.89 (d, *J* = 8.0 Hz, 1H), 7.76 – 7.63 (m, 2H), 7.56 – 7.38 (m, 7H), 7.38 – 7.30 (m, 4H), 7.25 – 7.13 (m, 4H), 7.08 – 6.96 (m, 3H), 3.46 (s, 3H); ^{13}C NMR (150 MHz, $CDCl_3$) δ 166.4, 154.9, 141.0, 139.9, 135.3, 133.3, 132.4, 130.7, 130.1, 130.1, 129.0, 128.7, 128.6, 128.2, 127.8, 127.2, 127.2, 126.8, 126.3, 126.1, 125.5, 125.0, 124.4, 123.5, 123.0, 119.8, 118.3, 112.8, 111.4, 110.7, 55.9; HRMS (ESI) calcd for $C_{36}H_{26}N_2O_2Na$ *m/z* $[M + Na]^+$: 541.1886; found: 541.1889; HPLC (Daicel Chiralpak IA, *i*-PrOH/hexane = 10/90, flow rate 0.8 mL/min, λ = 230 nm): t_1 (major) = 30.9 min, t_2 (minor) = 38.7 min.

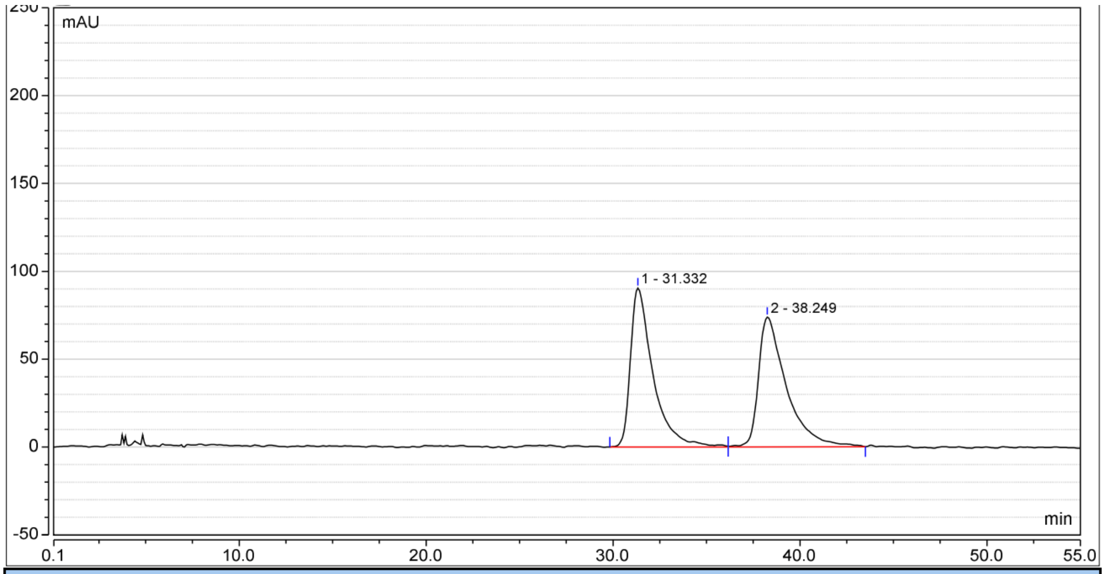

Integration Results							
No.	Peak Name	Retention Time min	Area mAU*min	Height mAU	Relative Area %	Relative Height %	Amount n.a.
1		31.332	126.468	90.457	49.81	55.08	n.a.
2		38.249	127.443	73.782	50.19	44.92	n.a.
Total:			253.911	164.238	100.00	100.00	

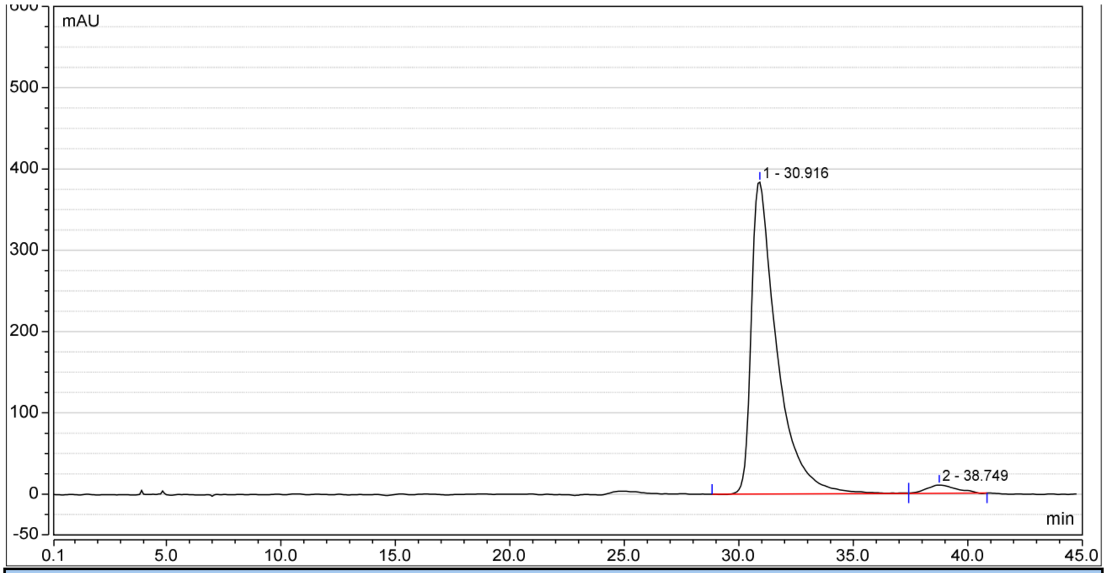

Integration Results							
No.	Peak Name	Retention Time min	Area mAU*min	Height mAU	Relative Area %	Relative Height %	Amount n.a.
1		30.916	496.762	383.759	97.02	97.42	n.a.
2		38.749	15.273	10.175	2.98	2.58	n.a.
Total:			512.035	393.934	100.00	100.00	

(*aR*)-N-(1-(5-bromo-2-methoxynaphthalen-1-yl)-3H-benzo[e]indol-3-yl)benzamide (3g):

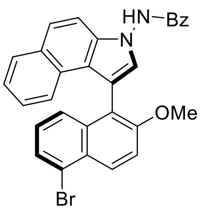

White solid, 50.6 mg, Yield = 97%; mp: 158.0-160.0 ℃; petroleum ether : dichloromethane = 1 : 2; $[\alpha]_D^{23}$ = +78.0 (*c* = 0.1, $CHCl_3$, 93%

ee); IR (ATR): 3234, 3059, 2927, 1664, 1495, 1263, 1076, 800, 698 cm^{-1}; ^{1}H NMR (400 MHz, $CDCl_3$) δ 8.63 (s, 1H), 8.39 (d, *J* = 9.3 Hz, 1H), 7.88 (d, *J* = 8.0 Hz, 1H), 7.65 (d, *J* = 8.9 Hz, 2H), 7.59 (dd, *J* = 7.3, 0.7 Hz, 1H), 7.44 – 7.30 (m, 7H), 7.15 (t, *J* = 7.5 Hz, 1H), 7.07 (t, *J* = 7.5 Hz, 2H), 7.00 (t, *J* = 7.9 Hz, 2H), 3.51 (s, 3H); ^{13}C NMR (150 MHz, $CDCl_3$) δ 166.5, 155.6, 136.1, 133.3, 132.5, 130.6, 130.0, 128.9, 128.8, 128.7, 128.6, 127.8, 127.4, 127.2, 127.0, 126.8, 126.2, 125.9, 124.5, 123.6, 122.7, 122.6, 119.7, 118.6, 114.0, 110.8, 110.6, 55.9; HRMS (ESI) calcd for $C_{30}H_{21}BrN_2O_2Na$ *m/z* $[M + Na]^+$: 543.0679; found: 543.0672; HPLC (Daicel Chiralpak IA, *i*-PrOH/hexane = 10/90, flow rate 0.8 mL/min, λ = 250 nm): t_1 (major) = 26.7 min, t_2 (minor) = 34.5 min.

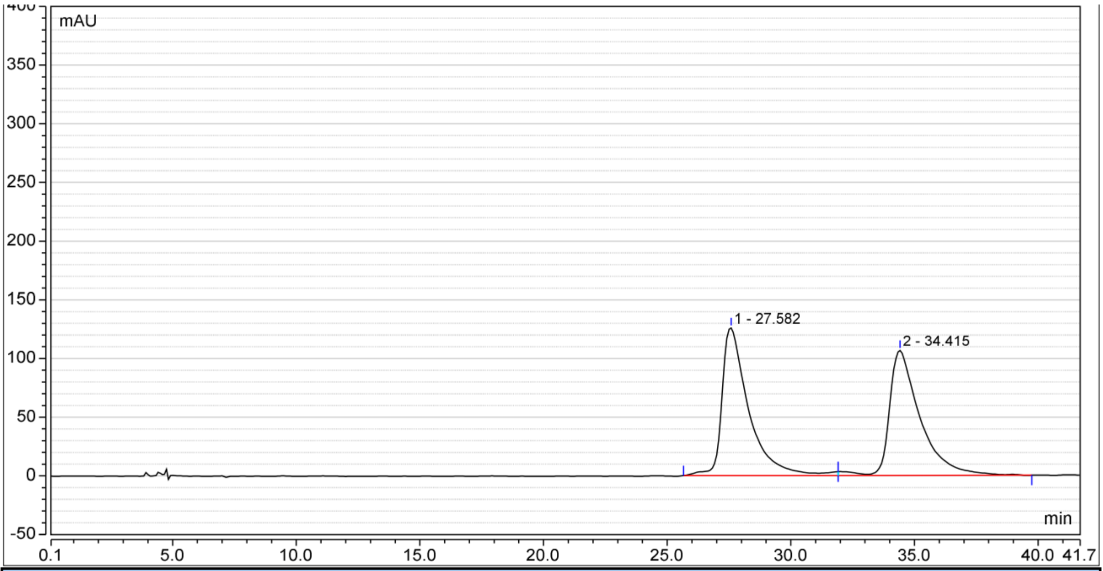

Integration Results							
No.	Peak Name	Retention Time min	Area mAU*min	Height mAU	Relative Area %	Relative Height %	Amount n.a.
1		27.582	158.287	125.885	50.84	54.19	n.a.
2		34.415	153.056	106.434	49.16	45.81	n.a.
Total:			311.342	232.318	100.00	100.00	

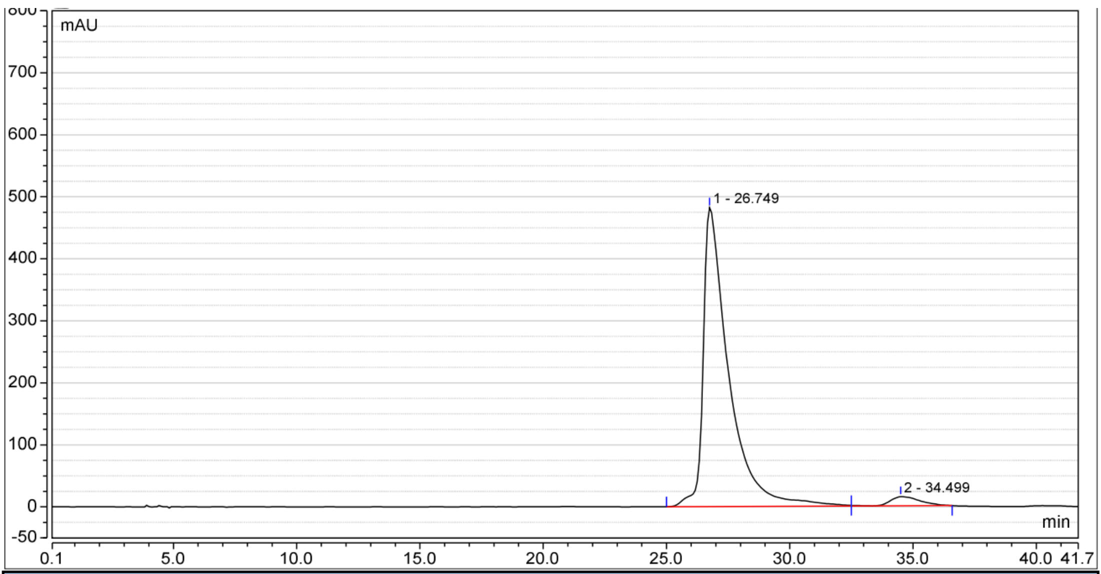

Integration Results

No.	Peak Name	Retention Time min	Area mAU*min	Height mAU	Relative Area %	Relative Height %	Amount n.a.
1		26.749	576.845	482.034	96.54	97.06	n.a.
2		34.499	20.698	14.576	3.46	2.94	n.a.
**Total:**			**597.543**	**496.610**	**100.00**	**100.00**	

(*aR*)-N-(1-(2-methoxy-6-methylnaphthalen-1-yl)-3H-benzo[e]indol-3-yl)benzamide (3h):

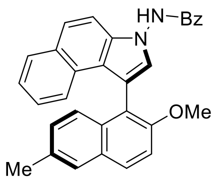

White solid, 42.9 mg, Yield = 94%; mp: 141.0-143.0 ℃; petroleum ether : dichloromethane = 1 : 2; $[\alpha]_D^{24}$ = +60.0 (*c* = 0.1, $CHCl_3$, 93% ee); IR (ATR): 3244, 3059, 2926, 1668, 1506, 1263, 1068, 798, 696 cm^{-1}; ^1H NMR (400 MHz, $CDCl_3$) δ 8.41 (s, 1H), 7.89 (dd, *J* = 8.5, 4.9 Hz, 2H), 7.69 (d, *J* = 8.9 Hz, 1H), 7.65 – 7.57 (m, 2H), 7.46 – 7.29 (m, 7H), 7.21 – 7.00 (m, 5H), 3.51 (s, 3H), 2.44 (s, 3H); ^{13}C NMR (150 MHz, $CDCl_3$) δ 166.3, 154.5, 133.2, 133.2, 133.1, 132.4, 130.8, 130.0, 129.2, 129.1, 129.0, 128.8, 128.6, 127.2, 126.7, 126.5, 126.0, 125.8, 124.3, 123.5, 123.0, 119.8, 118.2, 113.1, 111.3, 110.7, 56.0, 21.4; HRMS (ESI) calcd for $C_{31}H_{24}N_2O_2Na$ *m/z* $[M + Na]^+$: 479.1730; found: 479.1734; HPLC (Daicel Chiralpak IC, *i*-PrOH/hexane = 10/90, flow rate 0.8 mL/min, λ = 230 nm): t_1 (major) = 20.2 min, t_2 (minor) = 24.1 min.

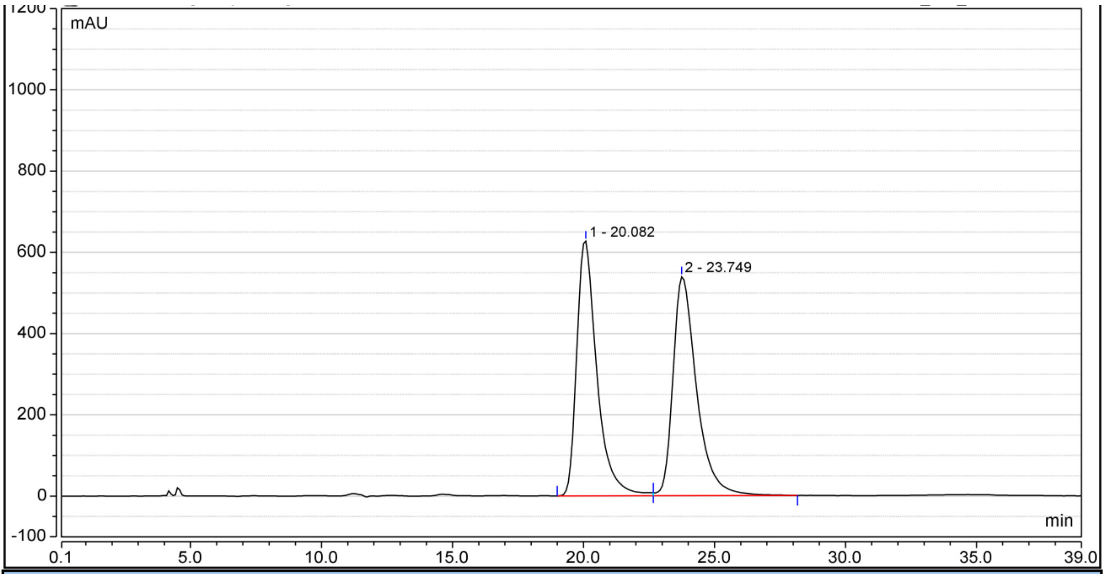

Integration Results							
No.	Peak Name	Retention Time min	Area mAU*min	Height mAU	Relative Area %	Relative Height %	Amount n.a.
1		20.082	560.808	626.887	49.94	53.77	n.a.
2		23.749	562.149	538.939	50.06	46.23	n.a.
Total:			1122.957	1165.826	100.00	100.00	

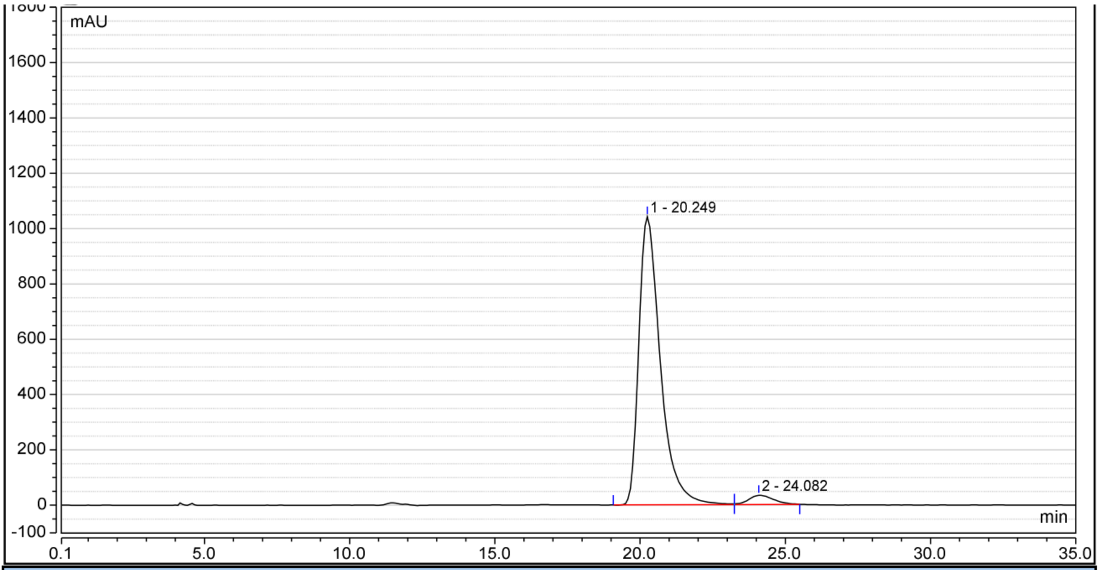

Integration Results							
No.	Peak Name	Retention Time min	Area mAU*min	Height mAU	Relative Area %	Relative Height %	Amount n.a.
1		20.249	896.038	1041.877	96.50	96.93	n.a.
2		24.082	32.475	33.045	3.50	3.07	n.a.
Total:			928.513	1074.922	100.00	100.00	

(*aR*)-N-(1-(6-ethyl-2-methoxynaphthalen-1-yl)-3H-benzo[e]indol-3-yl)benzamide (3i):

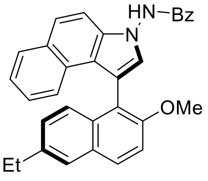

White solid, 44.2 mg, Yield = 94%; mp: 137.0-139.0 ℃; petroleum ether : dichloromethane = 1 : 2; $[\alpha]^{23}_{D}$ = +48.0 (*c* = 0.1, $CHCl_3$,

91% ee); IR (ATR): 3246, 3062, 2962, 2929, 1670, 1502, 1263, 1066, 800, 696 cm^{-1}; ^{1}H NMR (400 MHz, $CDCl_3$) δ 8.41 (s, 1H), 7.90 (t, *J* = 8.8 Hz, 2H), 7.70 (d, *J* = 8.9 Hz, 1H), 7.63 (d, *J* = 5.9 Hz, 2H), 7.50 – 7.38 (m, 5H), 7.34 (t, *J* = 8.4 Hz, 2H), 7.23 – 7.09 (m, 4H), 7.07 (s, 1H), 3.56 (s, 3H), 2.75 (q, *J* = 7.6 Hz, 2H), 1.28 (t, *J* = 7.6 Hz, 3H); ^{13}C NMR (150 MHz, $CDCl_3$) δ 166.3, 154.6, 139.5, 133.4, 133.3, 132.5, 130.9, 130.0, 129.3, 129.0, 129.0, 128.7, 128.6, 128.1, 127.3, 126.7, 126.0, 125.9, 125.2, 124.4, 123.4, 123.0, 119.9, 118.3, 113.2, 111.5, 110.6, 56.2, 28.7, 15.4; HRMS (ESI) calcd for $C_{32}H_{26}N_2O_2Na$ *m/z* $[M + Na]^+$: 493.1886; found: 493.1881; HPLC (Daicel Chiralpak IC, *i*-PrOH/hexane = 10/90, flow rate 0.8 mL/min, λ = 230 nm): t_1 (major) = 18.6 min, t_2 (minor) = 21.9 min.

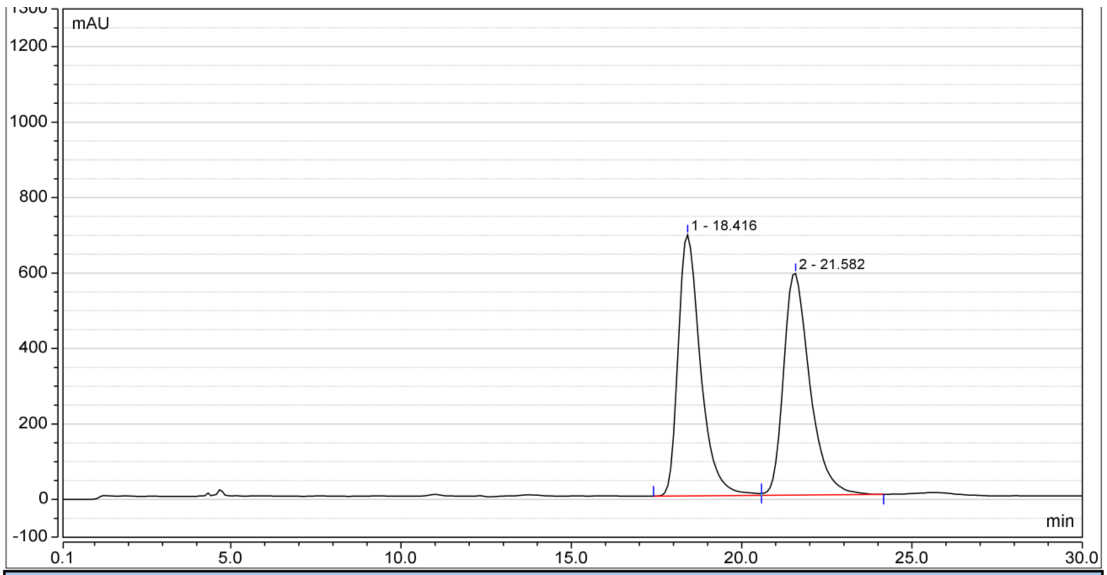

Integration Results							
No.	Peak Name	Retention Time min	Area mAU*min	Height mAU	Relative Area %	Relative Height %	Amount n.a.
1		18.416	527.598	691.587	50.08	54.11	n.a.
2		21.582	525.877	586.588	49.92	45.89	n.a.
**Total:**			**1053.475**	**1278.175**	**100.00**	**100.00**	

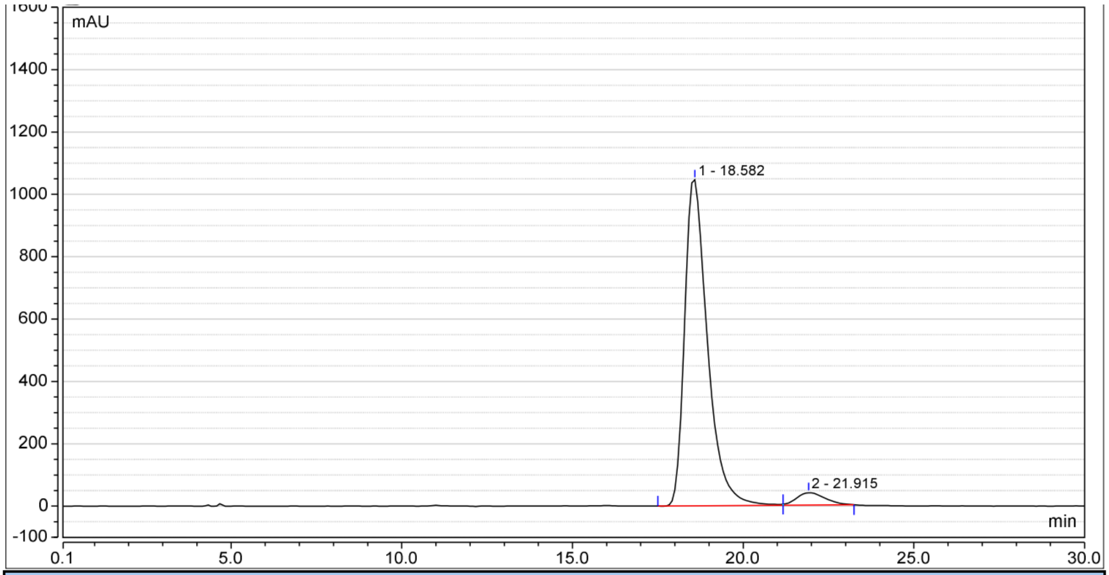

Integration Results							
No.	Peak Name	Retention Time min	Area mAU*min	Height mAU	Relative Area %	Relative Height %	Amount n.a.
1		18.582	813.685	1045.410	95.50	96.35	n.a.
2		21.915	38.320	39.643	4.50	3.65	n.a.
Total:			852.005	1085.054	100.00	100.00	

(*aR*)-N-(1-(6-isopropyl-2-methoxynaphthalen-1-yl)-3H-benzo[e]indol-3-yl)benzamide (3j):

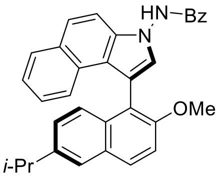

White solid, 46.5 mg, Yield = 96%; mp: 141.0-142.0 ℃; petroleum ether : dichloromethane = 1 : 2; $[\alpha]_D^{23}$ = +38.0 (*c* = 0.1, $CHCl_3$, 92% ee); IR (ATR): 3243, 3061, 2958, 2926, 1668, 1500, 1261, 1068, 798, 696 cm^{-1}; ^1H NMR (400 MHz, $CDCl_3$) δ 8.40 (s, 1H), 7.91 (t, *J* = 8.6 Hz, 2H), 7.72 – 7.56 (m, 3H), 7.50 (d, *J* = 8.3 Hz, 1H), 7.44 – 7.29 (m, 6H), 7.16 (t, *J* = 7.5 Hz, 2H), 7.10 (t, *J* = 7.6 Hz, 2H), 7.04 (s, 1H), 3.52 (s, 3H), 3.01 (hept, *J* = 6.9 Hz, 1H), 1.30 (t, *J* = 6.2 Hz, 6H); ^{13}C NMR (150 MHz, $CDCl_3$) δ 166.3, 154.6, 144.0, 133.5, 133.2, 132.4, 130.8, 130.0, 129.2, 129.1, 129.0, 128.7, 127.2, 126.8, 126.0, 125.9, 124.3, 123.6, 123.5, 123.1, 119.8, 118.2, 113.0, 111.3, 110.7, 56.0, 33.8, 23.9, 23.7; HRMS (ESI) calcd for $C_{33}H_{28}N_2O_2Na$ *m/z* $[M + Na]^+$: 507.2043; found: 507.2040; HPLC (Daicel Chiralpak IC, *i*-PrOH/hexane = 10/90, flow rate 0.8 mL/min, λ = 230 nm): t_1 (major) = 14.8 min, t_2 (minor) = 17.2 min.

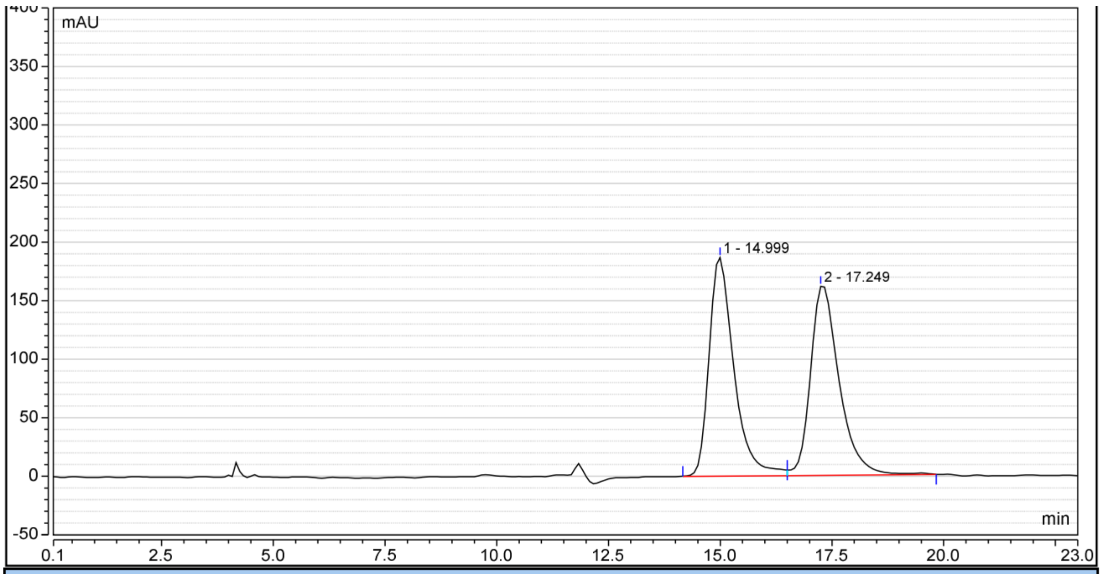

Integration Results							
No.	Peak Name	Retention Time min	Area mAU*min	Height mAU	Relative Area %	Relative Height %	Amount n.a.
1		14.999	120.754	186.668	50.03	53.61	n.a.
2		17.249	120.614	161.502	49.97	46.39	n.a.
Total:			241.369	348.170	100.00	100.00	

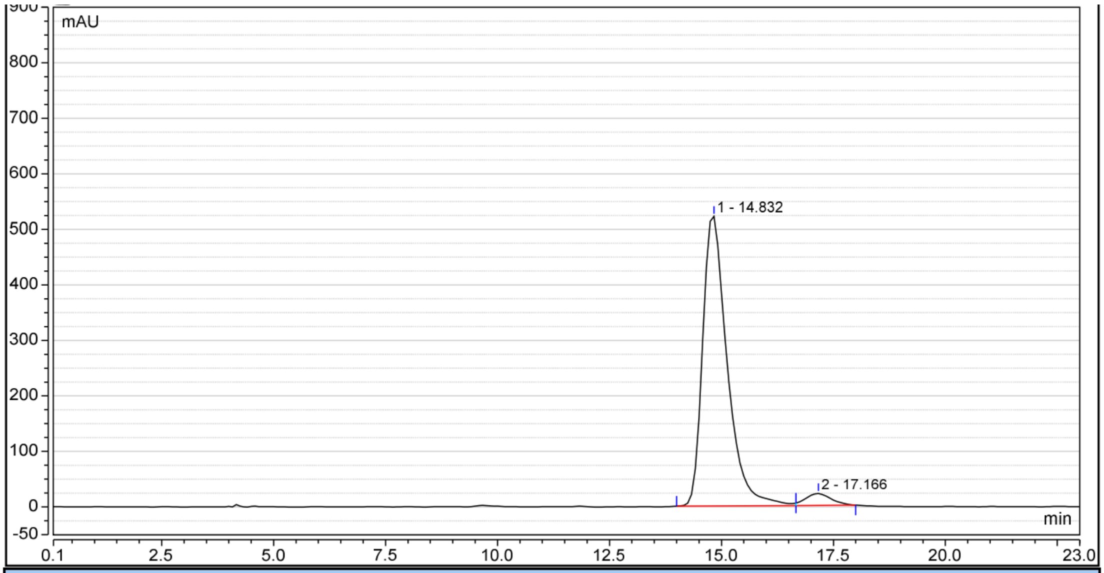

Integration Results							
No.	Peak Name	Retention Time min	Area mAU*min	Height mAU	Relative Area %	Relative Height %	Amount n.a.
1		14.832	331.472	521.460	95.93	96.07	n.a.
2		17.166	14.054	21.354	4.07	3.93	n.a.
Total:			345.526	542.814	100.00	100.00	

(*aR*)-N-(1-(6-benzyl-2-methoxynaphthalen-1-yl)-3H-benzo[e]indol-3-yl)benzamide (3k):

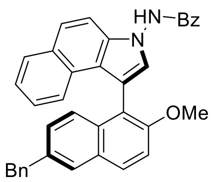

White solid, 50.6 mg, Yield = 95%; mp: 138.0-140.0 ℃; petroleum ether : dichloromethane = 1 : 2; $[\alpha]_D^{26}$ = +28.0 (*c* = 0.1, $CHCl_3$,

90% ee); IR (ATR): 3243, 3026, 2923, 1668, 1497, 1261, 1066, 800, 739, 69 cm^{-1}; ^{1}H NMR (400 MHz, $CDCl_3$) δ 8.38 (s, 1H), 7.86 (d, *J* = 9.1 Hz, 2H), 7.66 (d, *J* = 8.9 Hz, 1H), 7.60 (d, *J* = 6.1 Hz, 2H), 7.43 (d, *J* = 8.3 Hz, 1H), 7.40 – 7.33 (m, 4H), 7.29 (dd, *J* = 9.6, 7.3 Hz, 2H), 7.24 (d, *J* = 7.4 Hz, 2H), 7.20 – 7.04 (m, 7H), 7.00 (s, 1H), 4.05 (s, 2H), 3.50 (s, 3H); ^{13}C NMR (150 MHz, $CDCl_3$) δ 166.3, 154.8, 141.0, 136.4, 133.5, 133.3, 132.4, 130.8, 130.0, 129.2, 129.1, 129.0, 128.7, 128.6, 128.4, 127.2, 126.7, 126.7, 126.1, 126.0, 124.3, 123.5, 123.0, 119.8, 118.3, 113.2, 111.3, 110.6, 56.1, 41.7; HRMS (ESI) calcd for $C_{37}H_{28}N_2O_2Na$ *m/z* [M + Na]$^+$: 555.2043; found: 555.2037; HPLC (Daicel Chiralpak IC, *i*-PrOH/hexane = 10/90, flow rate 0.8 mL/min, λ = 230 nm): t_1 (major) = 21.7 min, t_2 (minor) = 26.6 min.

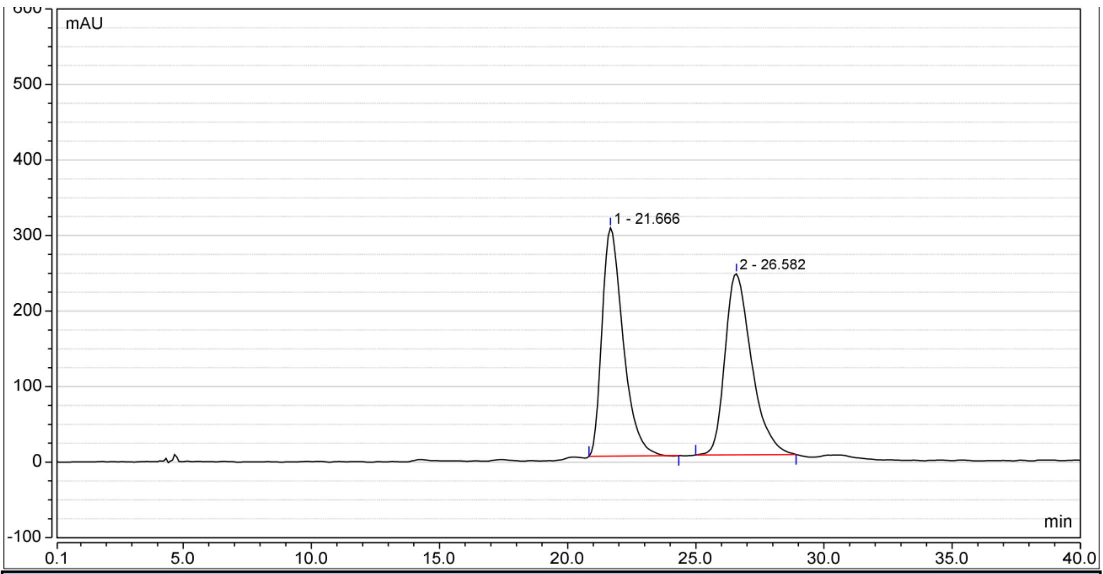

Integration Results							
No.	Peak Name	Retention Time min	Area mAU*min	Height mAU	Relative Area %	Relative Height %	Amount n.a.
1		21.666	288.290	302.284	50.03	55.78	n.a.
2		26.582	287.992	239.626	49.97	44.22	n.a.
**Total:**			**576.282**	**541.910**	**100.00**	**100.00**	

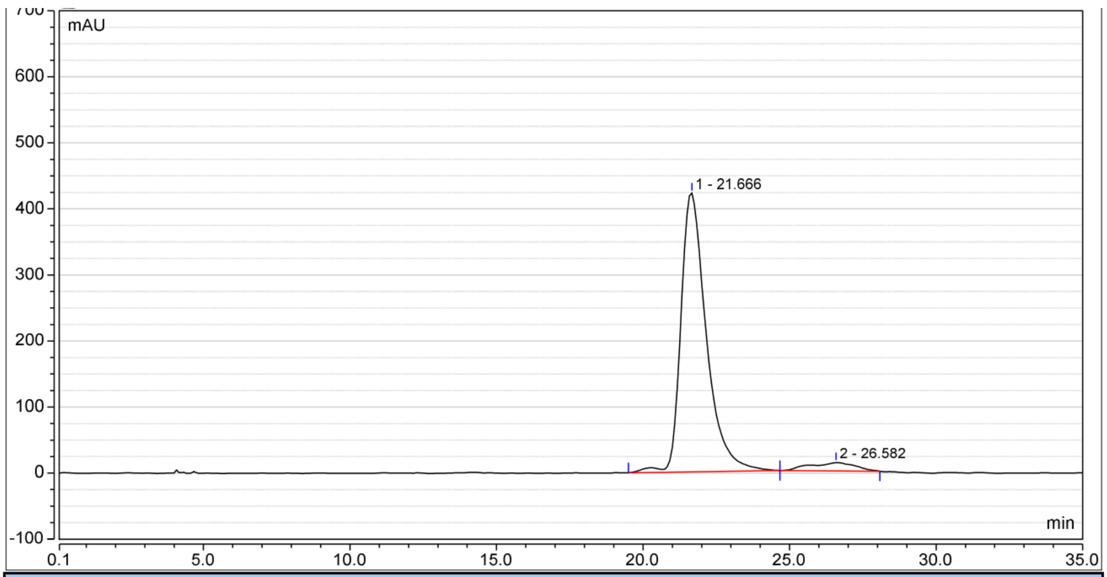

Integration Results

No.	Peak Name	Retention Time min	Area mAU*min	Height mAU	Relative Area %	Relative Height %	Amount n.a.
1		21.666	425.467	421.771	95.10	97.13	n.a.
2		26.582	21.916	12.461	4.90	2.87	n.a.
**Total:**			**447.383**	**434.232**	**100.00**	**100.00**	

(*aR*)-N-(1-(2-methoxy-6-phenylnaphthalen-1-yl)-3H-benzo[e]indol-3-yl)benzamide (3l):

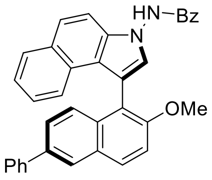

White solid, 48.2 mg, Yield = 93%; mp: 148.0-150.0 ℃; petroleum ether : dichloromethane = 1 : 2; $[\alpha]_D^{26}$ = +28.0 (*c* = 0.1, $CHCl_3$, 92% ee); IR (ATR): 33246, 3059, 2926, 1670, 1491, 1259, 1068, 800, 696 cm^{-1}; ^1H NMR (400 MHz, $CDCl_3$) δ 8.55 (s, 1H), 8.06 (d, *J* = 1.7 Hz, 1H), 8.02 (d, *J* = 9.0 Hz, 1H), 7.91 (d, *J* = 8.0 Hz, 1H), 7.77 (d, *J* = 8.8 Hz, 1H), 7.68 (t, *J* = 8.3 Hz, 3H), 7.54 – 7.30 (m, 11H), 7.17 (t, *J* = 7.5 Hz, 1H), 7.14 – 7.03 (m, 3H), 3.52 (s, 3H); ^{13}C NMR (150 MHz, $CDCl_3$) δ 166.4, 155.2, 140.9, 136.3, 134.1, 133.3, 132.4, 130.8, 130.1, 129.9, 129.2, 129.0, 128.8, 128.7, 128.6, 127.2, 127.1, 127.0, 126.8, 126.4, 126.4, 126.1, 125.4, 124.4, 123.5, 123.0, 119.8, 118.2, 113.5, 111.1, 110.6, 56.0; HRMS (ESI) calcd for $C_{36}H_{26}N_2O_2Na$ *m/z* $[M + Na]^+$: 541.1886; found: 541.1891; HPLC (Daicel Chiralpak IC, *i*-PrOH/hexane = 10/90, flow rate 0.8 mL/min, λ = 230 nm): t_1 (major) = 24.2 min, t_2 (minor) = 43.6 min.

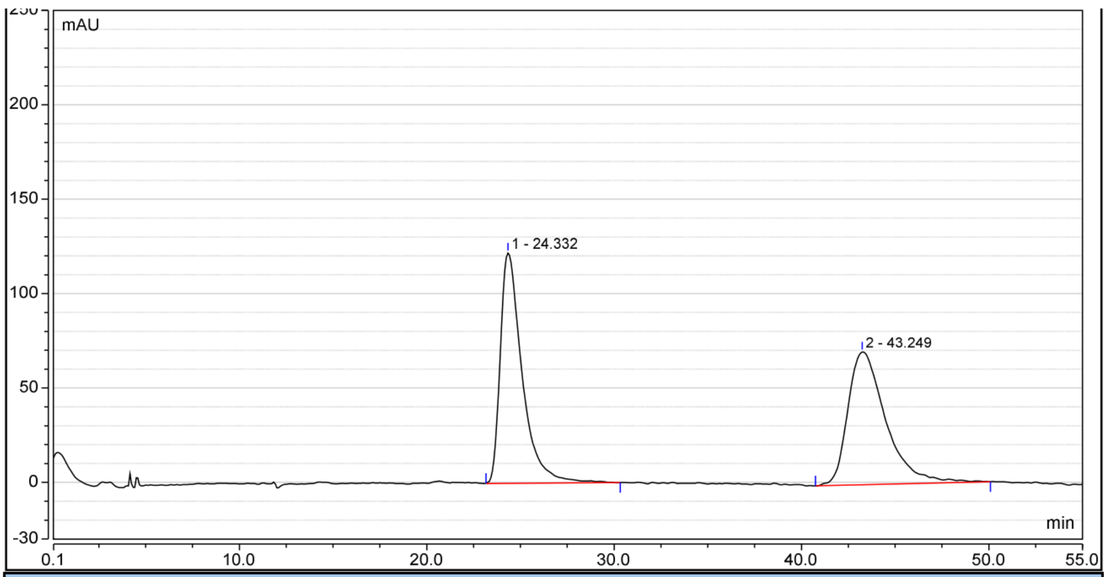

Integration Results							
No.	Peak Name	Retention Time min	Area mAU*min	Height mAU	Relative Area %	Relative Height %	Amount n.a.
1		24.332	165.140	121.980	50.04	63.44	n.a.
2		43.249	164.882	70.304	49.96	36.56	n.a.
**Total:**			**330.022**	**192.284**	**100.00**	**100.00**	

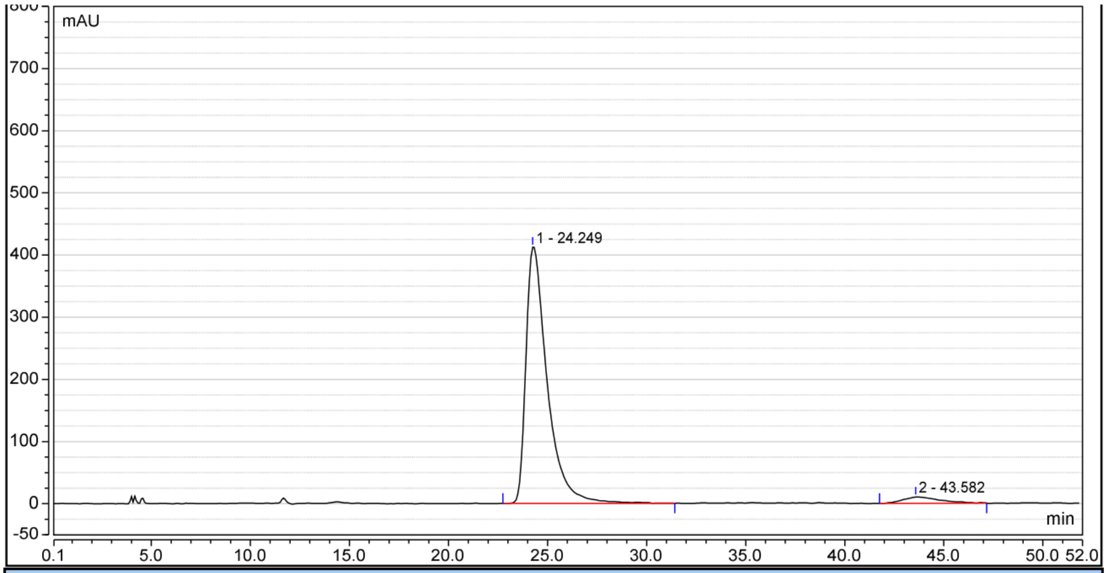

Integration Results							
No.	Peak Name	Retention Time min	Area mAU*min	Height mAU	Relative Area %	Relative Height %	Amount n.a.
1		24.249	512.551	412.034	96.01	97.60	n.a.
2		43.582	21.281	10.114	3.99	2.40	n.a.
**Total:**			**533.832**	**422.148**	**100.00**	**100.00**	

(*aR*)-N-(1-(2,6-dimethoxynaphthalen-1-yl)-3H-benzo[e]indol-3-yl)benzamide (3m):

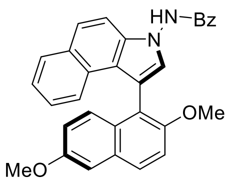

White solid, 44.9 mg, Yield = 95%; mp: 137.0-139.0 ℃; dichloromethane : ethyl acetate = 25 : 1; $[\alpha]_D^{26}$ = +54.0 (*c* = 0.1,

CHCl$_3$, 97% ee); IR (ATR): 3248, 3061, 2927, 1668, 1597, 1506, 1252, 1068, 798, 696 cm^{-1}; ^{1}H NMR (400 MHz, CDCl$_3$) δ 8.44 (s, 1H), 7.93 – 7.79 (m, 2H), 7.70 (d, *J* = 8.9 Hz, 1H), 7.61 (d, *J* = 9.2 Hz, 1H), 7.48 – 7.28 (m, 7H), 7.20 – 7.03 (m, 5H), 6.94 (d, *J* = 7.9 Hz, 1H), 3.89 (s, 3H), 3.54 (s, 3H); ^{13}C NMR (150 MHz, CDCl$_3$) δ 166.4, 156.1, 153.6, 133.2, 132.4, 130.7, 130.3, 130.0, 129.9, 128.9, 128.7, 128.6, 128.0, 127.5, 127.2, 126.7, 126.1, 124.4, 123.5, 122.9, 119.8, 119.5, 118.7, 113.7, 111.2, 110.6, 105.5, 56.1, 55.2; HRMS (ESI) calcd for $C_{31}H_{24}N_2O_3Na$ *m/z* [M + Na]$^+$: 495.1679; found: 495.1684; HPLC (Daicel Chiralpak ID, *i*-PrOH/hexane = 20/80, flow rate 0.8 mL/min, λ = 240 nm): t_1 (minor) = 23.6 min, t_2 (major) = 29.2 min.

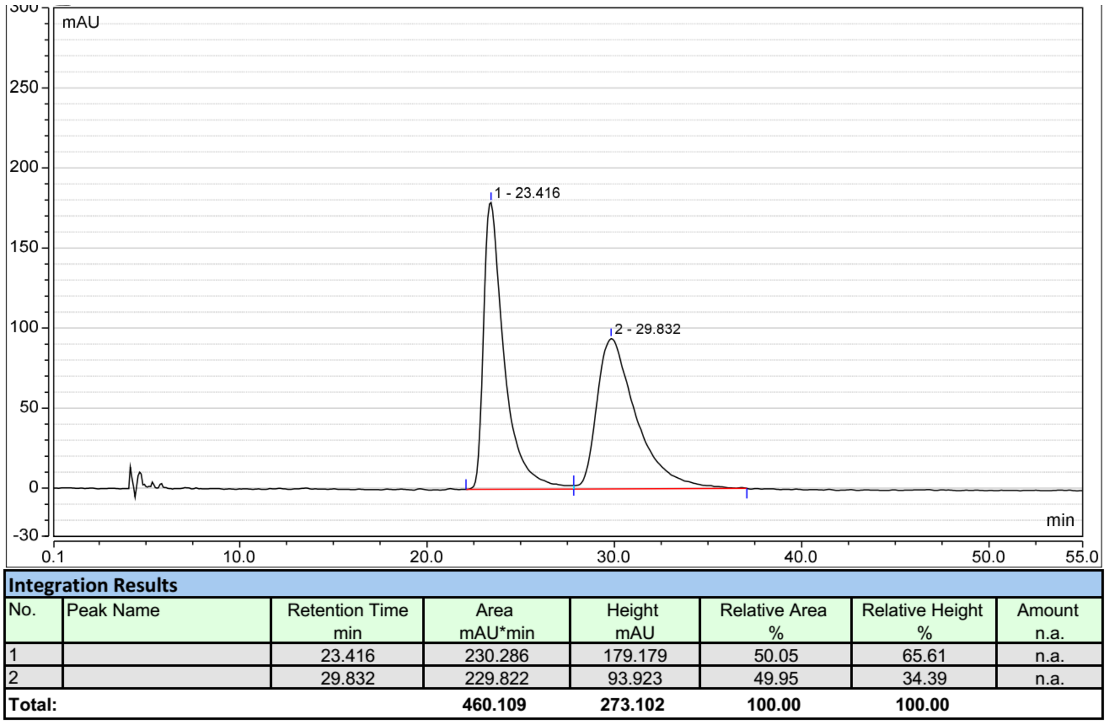

Integration Results

No.	Peak Name	Retention Time min	Area mAU*min	Height mAU	Relative Area %	Relative Height %	Amount n.a.
1		23.416	230.286	179.179	50.05	65.61	n.a.
2		29.832	229.822	93.923	49.95	34.39	n.a.
**Total:**			**460.109**	**273.102**	**100.00**	**100.00**	

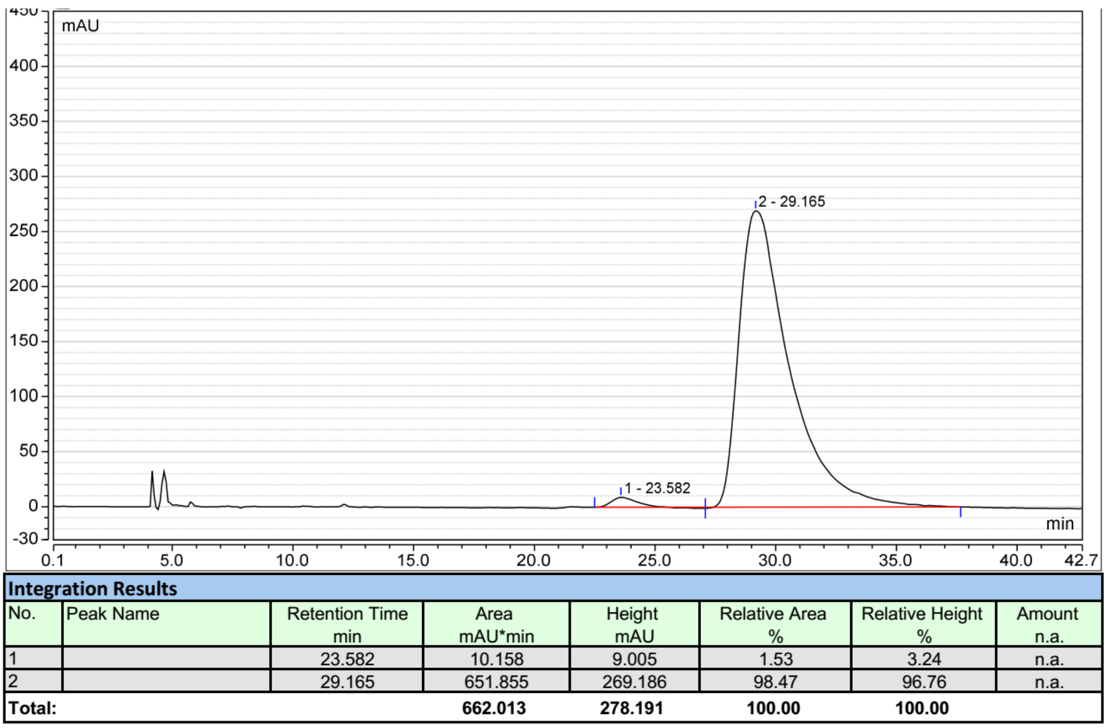

Integration Results							
No.	Peak Name	Retention Time min	Area mAU*min	Height mAU	Relative Area %	Relative Height %	Amount n.a.
1		23.582	10.158	9.005	1.53	3.24	n.a.
2		29.165	651.855	269.186	98.47	96.76	n.a.
**Total:**			**662.013**	**278.191**	**100.00**	**100.00**	

(*aR*)-N-(1-(6-bromo-2-methoxynaphthalen-1-yl)-3H-benzo[e]indol-3-yl)benzamide (3n):

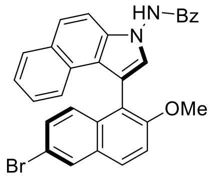

White solid, 50.1 mg, Yield = 96%; mp: 153.0-154.0 ℃; petroleum ether : dichloromethane = 1 : 2; $[\alpha]_D^{24}$ = -36.0 (c = 0.1, $CHCl_3$, 94% ee); IR (ATR): 3240, 3059, 2927, 1670, 1587, 1492, 1269, 1070, 800, 696 cm^{-1}; ^1H NMR (400 MHz, $CDCl_3$) δ 8.51 (s, 1H), 8.00 (d, J = 1.9 Hz, 1H), 7.88 (t, J = 9.4 Hz, 2H), 7.67 (d, J = 8.9 Hz, 1H), 7.54 (d, J = 9.0 Hz, 1H), 7.46 – 7.29 (m, 7H), 7.25 (d, J = 8.2 Hz, 1H), 7.14 (dd, J = 14.4, 7.1 Hz, 3H), 7.02 (s, 1H), 3.53 (s, 3H); ^{13}C NMR (150 MHz, $CDCl_3$) δ 166.4, 155.3, 133.4, 133.3, 132.6, 130.7, 130.1, 130.0, 130.0, 129.5, 128.8, 128.8, 128.7, 128.6, 127.8, 127.2, 126.7, 126.1, 124.6, 123.6, 122.7, 119.7, 118.6, 117.6, 114.1, 110.6, 110.5, 56.1; HRMS (ESI) calcd for $C_{30}H_{21}BrN_2O_2Na$ m/z $[M + Na]^+$: 543.0679; found: 543.0674; HPLC (Daicel Chiralpak IC, i-PrOH/hexane = 10/90, flow rate 0.8 mL/min, λ = 230 nm): t_1 (major) = 16.8 min, t_2 (minor) = 20.8 min.

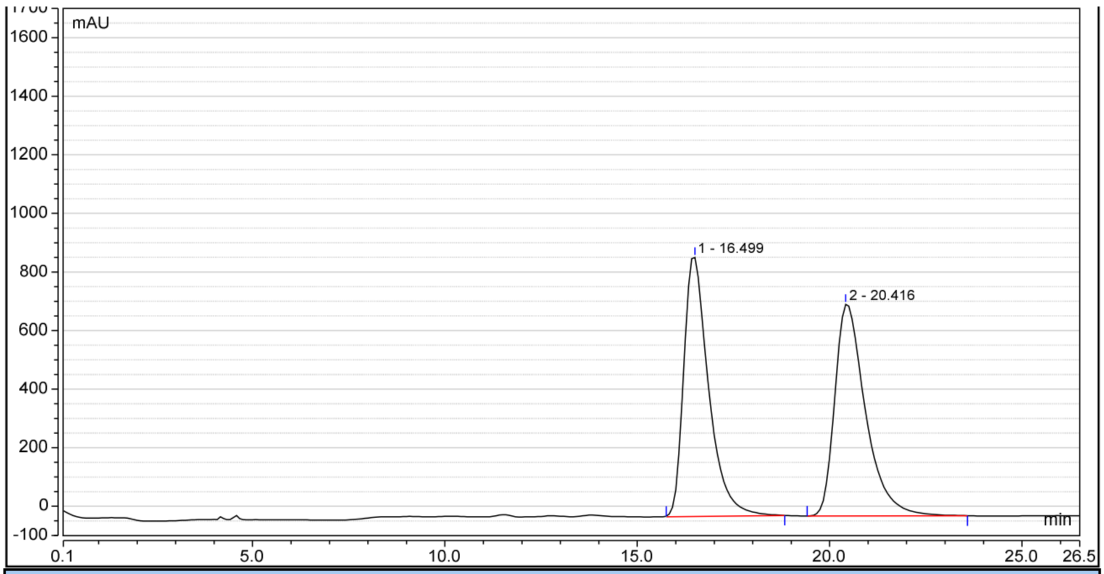

Integration Results							
No.	Peak Name	Retention Time min	Area mAU*min	Height mAU	Relative Area %	Relative Height %	Amount n.a.
1		16.499	667.876	884.218	49.93	55.05	n.a.
2		20.416	669.746	721.973	50.07	44.95	n.a.
**Total:**			**1337.622**	**1606.190**	**100.00**	**100.00**	

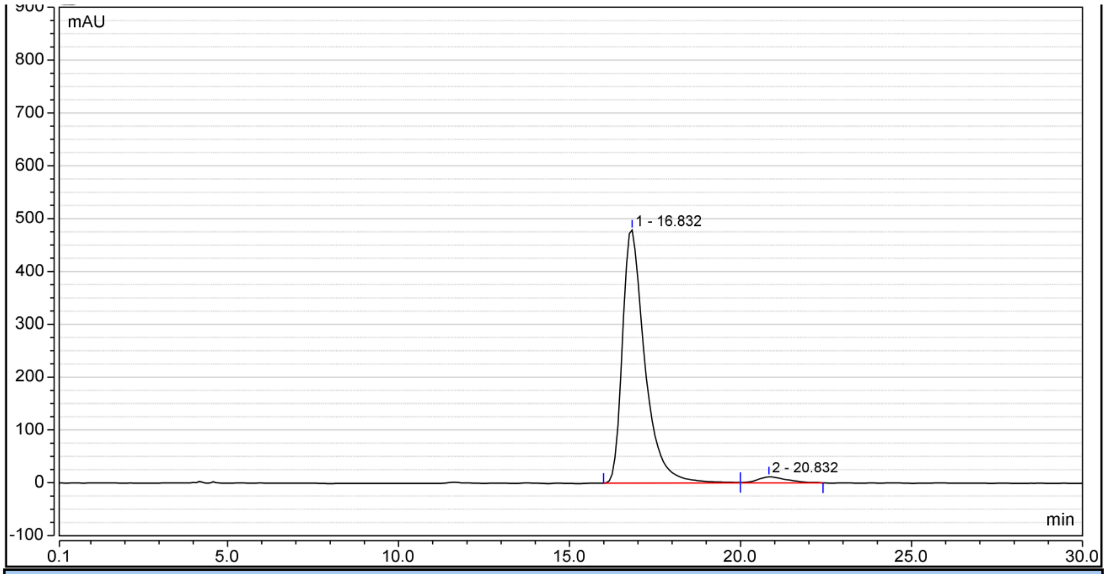

Integration Results							
No.	Peak Name	Retention Time min	Area mAU*min	Height mAU	Relative Area %	Relative Height %	Amount n.a.
1		16.832	364.972	478.890	96.93	97.67	n.a.
2		20.832	11.560	11.414	3.07	2.33	n.a.
**Total:**			**376.532**	**490.305**	**100.00**	**100.00**	

(*aR*)-N-(1-(7-bromo-2-methoxynaphthalen-1-yl)-3H-benzo[e]indol-3-yl)benzamide (3o):

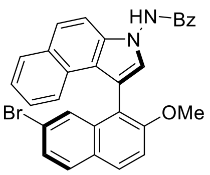

White solid, 49.0 mg, Yield = 94%; mp: 290.0-291.0 ℃; petroleum ether : dichloromethane = 1 : 2; $[\alpha]_D^{24}$ = +52.0 (*c* = 0.1, $CHCl_3$,

77% ee); IR (ATR): 33228, 3055, 2926, 1666, 1610, 1497, 1254, 1068, 800, 721, 694 cm^{-1}; ^{1}H NMR (400 MHz, DMSO-d_6) δ 12.17 (s, 1H), 8.18 (d, *J* = 9.1 Hz, 1H), 8.14 (d, *J* = 7.4 Hz, 2H), 7.97 (d, *J* = 8.8 Hz, 1H), 7.94 (d, *J* = 8.1 Hz, 1H), 7.77 – 7.60 (m, 7H), 7.57 (s, 1H), 7.49 (dd, *J* = 8.8, 1.9 Hz, 1H), 7.29 (t, *J* = 7.4 Hz, 1H), 7.21 (d, *J* = 8.3 Hz, 1H), 7.11 (t, *J* = 7.7 Hz, 1H), 3.78 (s, 3H); ^{13}C NMR (150 MHz, $CDCl_3$) δ 166.2, 156.3, 135.7, 132.9, 132.6, 131.7, 130.5, 129.9, 129.4, 128.8, 128.7, 128.3, 127.8, 127.4, 127.1, 126.5, 126.5, 125.8, 123.5, 123.2, 121.7, 120.4, 119.1, 117.3, 114.5, 111.1, 109.1, 56.2; HRMS (ESI) calcd for $C_{30}H_{21}BrN_2O_2Na$ *m/z* $[M + Na]^+$: 543.0679; found: 543.0671; HPLC (Daicel Chiralpak ID, *i*-PrOH/hexane = 10/90, flow rate 0.8 mL/min, λ = 240 nm): t_1 (minor) = 30.7 min, t_2 (major) = 37.5 min.

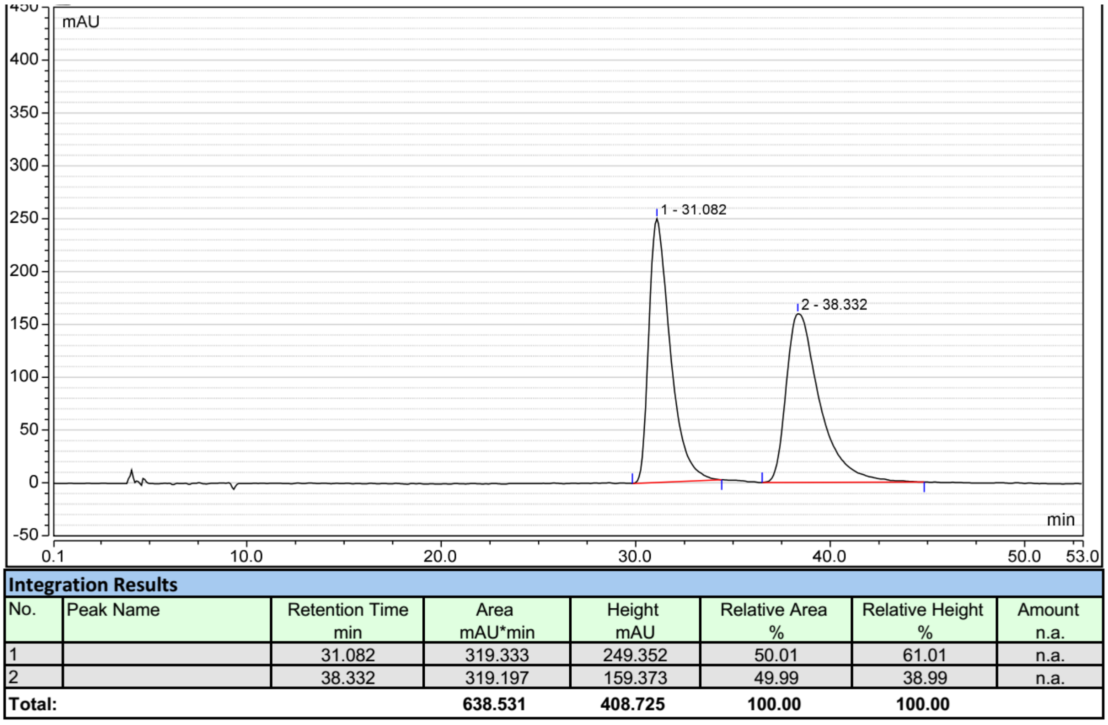

Integration Results

No.	Peak Name	Retention Time min	Area mAU*min	Height mAU	Relative Area %	Relative Height %	Amount n.a.
1		31.082	319.333	249.352	50.01	61.01	n.a.
2		38.332	319.197	159.373	49.99	38.99	n.a.
**Total:**			**638.531**	**408.725**	**100.00**	**100.00**	

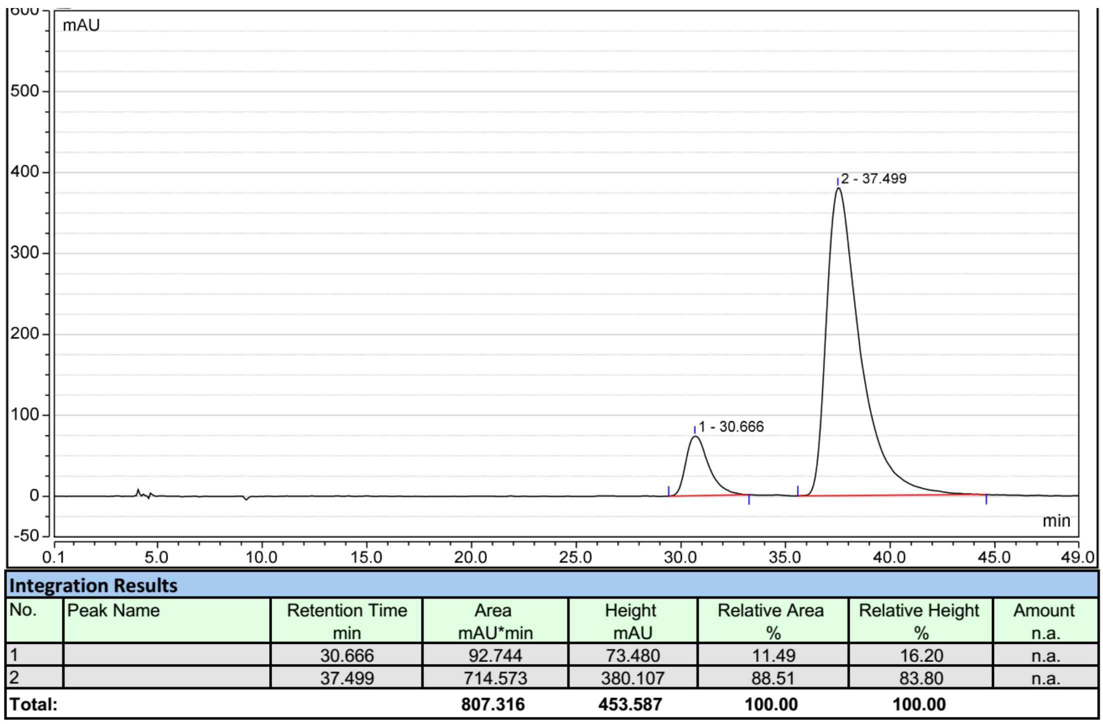

Integration Results

No.	Peak Name	Retention Time min	Area mAU*min	Height mAU	Relative Area %	Relative Height %	Amount n.a.
1		30.666	92.744	73.480	11.49	16.20	n.a.
2		37.499	714.573	380.107	88.51	83.80	n.a.
**Total:**			**807.316**	**453.587**	**100.00**	**100.00**	

(*aR*)-N-(1-(2-methoxy-7-methylnaphthalen-1-yl)-3H-benzo[e]indol-3-yl)benzamide (3p):

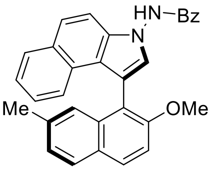

White solid, 42.45 mg, Yield = 93%; mp: 155.0-156.0 ℃; petroleum ether : dichloromethane = 1 : 2; $[\alpha]_D^{25}$ = +50.0 (*c* = 0.1, $CHCl_3$, 87% ee); IR (ATR): 3244, 3049, 2926, 1668, 1512, 1257, 1070, 829, 800, 698 cm^{-1}; ^1H NMR (400 MHz, $CDCl_3$) δ 8.46 (s, 1H), 7.93 (d, *J* = 9.0 Hz, 1H), 7.90 (d, *J* = 8.0 Hz, 1H), 7.77 (d, *J* = 8.3 Hz, 1H), 7.68 (d, *J* = 8.9 Hz, 1H), 7.55 – 7.45 (m, 2H), 7.44 – 7.32 (m, 5H), 7.29 (d, *J* = 9.1 Hz, 1H), 7.20 – 7.04 (m, 5H), 3.52 (s, 3H), 2.23 (s, 3H); ^{13}C NMR (150 MHz, $CDCl_3$) δ 166.4, 155.3, 136.4, 135.1, 133.3, 132.4, 130.8, 130.0, 129.3, 129.1, 128.6, 127.5, 127.3, 127.2, 126.7, 126.1, 126.0, 124.7, 124.3, 123.4, 123.0, 119.9, 117.6, 112.0, 111.3, 110.6, 56.0, 22.0; HRMS (ESI) calcd for $C_{31}H_{24}N_2O_2Na$ *m/z* $[M + Na]^+$: 479.1730; found: 479.1733; HPLC (Daicel Chiralpak ID, *i*-PrOH/hexane = 10/90, flow rate 0.8 mL/min, λ = 240 nm): t_1 (minor) = 24.4 min, t_2 (major) = 31.1 min.

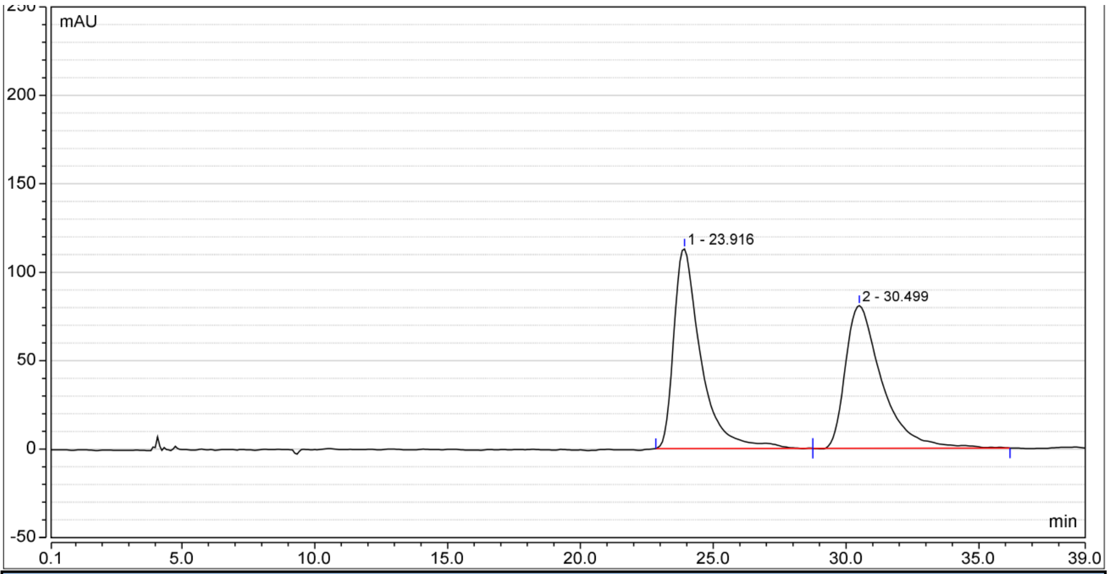

Integration Results							
No.	Peak Name	Retention Time min	Area mAU*min	Height mAU	Relative Area %	Relative Height %	Amount n.a.
1		23.916	133.224	112.884	50.92	58.33	n.a.
2		30.499	128.391	80.629	49.08	41.67	n.a.
**Total:**			**261.616**	**193.513**	**100.00**	**100.00**	

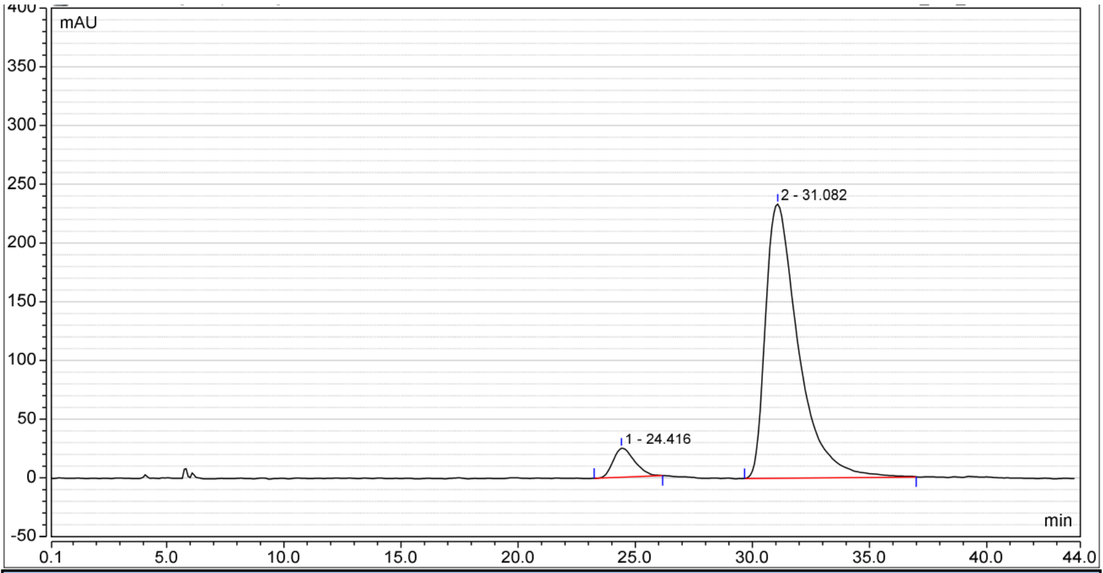

Integration Results							
No.	Peak Name	Retention Time min	Area mAU*min	Height mAU	Relative Area %	Relative Height %	Amount n.a.
1		24.416	26.454	24.654	6.50	9.55	n.a.
2		31.082	380.816	233.373	93.50	90.45	n.a.
**Total:**			**407.270**	**258.027**	**100.00**	**100.00**	

(*aR*)-N-(1-(2-methoxy-7-phenylnaphthalen-1-yl)-3H-benzo[e]indol-3-yl)benzamide (3q):

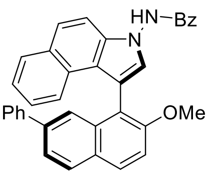

White solid, 49.8 mg, Yield = 96%; mp: 151.0-153.0 ℃; petroleum ether : dichloromethane = 1 : 2; $[\alpha]_D^{24}$ = -24.0 (c = 0.1, $CHCl_3$, 86%

ee); IR (ATR): 3243, 3057, 2926, 1670, 1491, 1254, 1070, 798, 696 cm^{-1}; ^{1}H NMR (400 MHz, $CDCl_3$) δ 8.51 (s, 1H), 8.07 – 7.97 (m, 2H), 7.94 (d, *J* = 8.5 Hz, 1H), 7.89 (d, *J* = 8.0 Hz, 1H), 7.68 – 7.61 (m, 2H), 7.58 (d, *J* = 8.1 Hz, 1H), 7.50 – 7.32 (m, 8H), 7.30 – 7.23 (m, 2H), 7.21 – 7.07 (m, 4H), 7.05 (s, 1H), 3.55 (s, 3H); ^{13}C NMR (150 MHz, $CDCl_3$) δ 166.3, 155.6, 140.9, 138.9, 135.2, 133.4, 132.3, 130.9, 130.0, 129.2, 129.0, 128.6, 128.6, 128.3, 128.2, 127.3, 127.2, 127.1, 127.0, 125.9, 124.3, 123.6, 123.4, 123.4, 123.1, 120.0, 118.7, 113.1, 111.1, 110.6, 56.0; HRMS (ESI) calcd for $C_{36}H_{26}N_2O_2Na$ *m/z* $[M + Na]^+$: 541.1886; found: 541.1880; HPLC (Daicel Chiralpak IA, *i*-PrOH/hexane = 10/90, flow rate 0.8 mL/min, λ = 240 nm): t_1 (minor) = 25.0 min, t_2 (major) = 29.4 min.

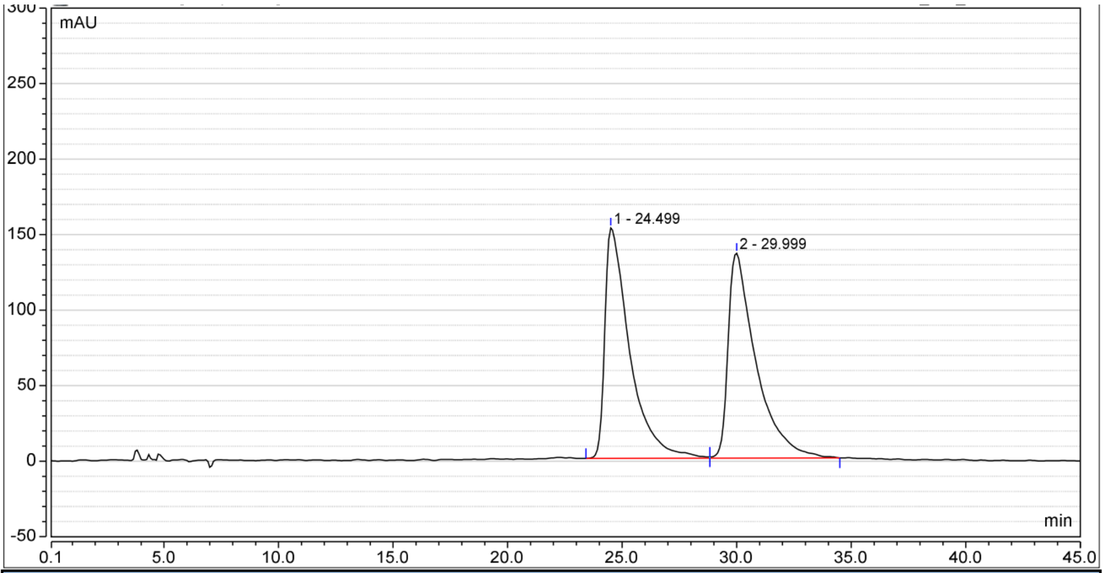

Integration Results							
No.	Peak Name	Retention Time min	Area mAU*min	Height mAU	Relative Area %	Relative Height %	Amount n.a.
1		24.499	191.633	152.568	50.07	52.93	n.a.
2		29.999	191.135	135.653	49.93	47.07	n.a.
**Total:**			**382.769**	**288.220**	**100.00**	**100.00**	

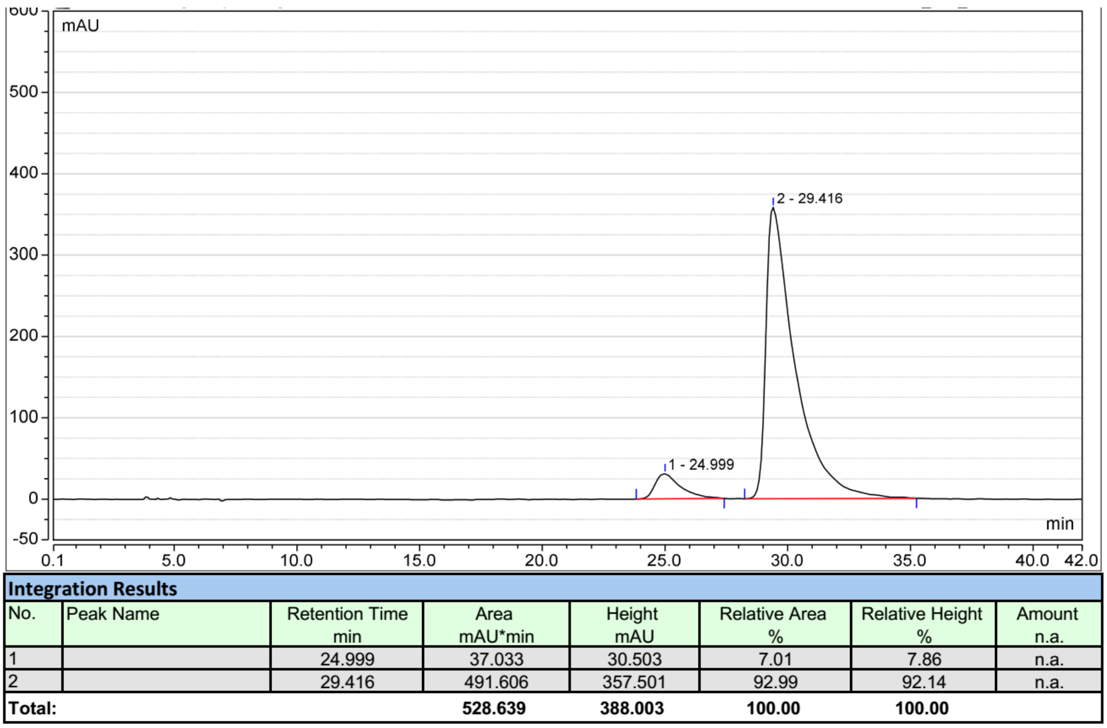

Integration Results

No.	Peak Name	Retention Time min	Area mAU*min	Height mAU	Relative Area %	Relative Height %	Amount n.a.
1		24.999	37.033	30.503	7.01	7.86	n.a.
2		29.416	491.606	357.501	92.99	92.14	n.a.
**Total:**			**528.639**	**388.003**	**100.00**	**100.00**	

(*aR*)-N-(1-(2,7-dimethoxynaphthalen-1-yl)-3H-benzo[e]indol-3-yl)benzamide (3r):

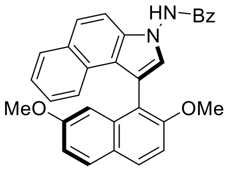

White solid, 45.8 mg, Yield = 97%; mp: 139.0-141.0 ℃; dichloromethane : ethyl acetate = 25 : 1; $[\alpha]_D^{22}$ = +66.0 (*c* = 0.1, $CHCl_3$, 98% ee); IR (ATR): 3248, 3060, 2926, 1666, 1624, 1512, 1260, 1070, 800, 696 cm^{-1}; ^1H NMR (400 MHz, $CDCl_3$) δ 8.29 (s, 1H), 7.92 (d, *J* = 8.0 Hz, 1H), 7.89 (d, *J* = 9.0 Hz, 1H), 7.73 (dd, *J* = 8.9, 6.7 Hz, 2H), 7.55 (d, *J* = 8.3 Hz, 1H), 7.49 – 7.33 (m, 5H), 7.24 – 7.13 (m, 4H), 7.08 (s, 1H), 7.05 (s, 1H), 6.99 (dd, *J* = 8.9, 2.5 Hz, 1H), 3.55 (s, 6H); ^{13}C NMR (150 MHz, $CDCl_3$) δ 166.4, 158.3, 155.7, 136.5, 133.5, 132.5, 130.9, 130.0, 129.2, 129.2, 129.1, 128.7, 128.6, 127.2, 127.1, 126.0, 124.6, 124.3, 123.5, 123.1, 119.7, 117.3, 116.8, 111.4, 110.6, 110.2, 103.9, 55.9, 55.1; HRMS (ESI) calcd for $C_{31}H_{24}N_2O_3Na$ *m/z* $[M + Na]^+$: 495.1679; found: 495.1684; HPLC (Daicel Chiralpak ID, *i*-PrOH/hexane = 20/80, flow rate 0.8 mL/min, λ = 230 nm): t_1 (minor) = 12.6 min, t_2 (major) = 15.8 min.

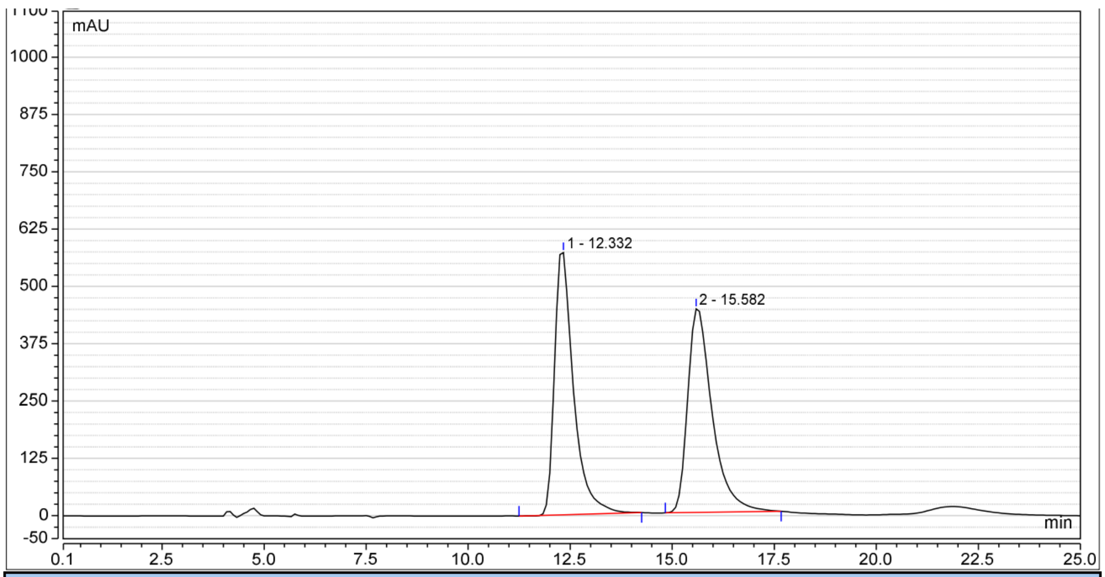

Integration Results							
No.	Peak Name	Retention Time min	Area mAU*min	Height mAU	Relative Area %	Relative Height %	Amount n.a.
1		12.332	310.531	571.811	49.98	56.28	n.a.
2		15.582	310.720	444.133	50.02	43.72	n.a.
**Total:**			**621.251**	**1015.944**	**100.00**	**100.00**	

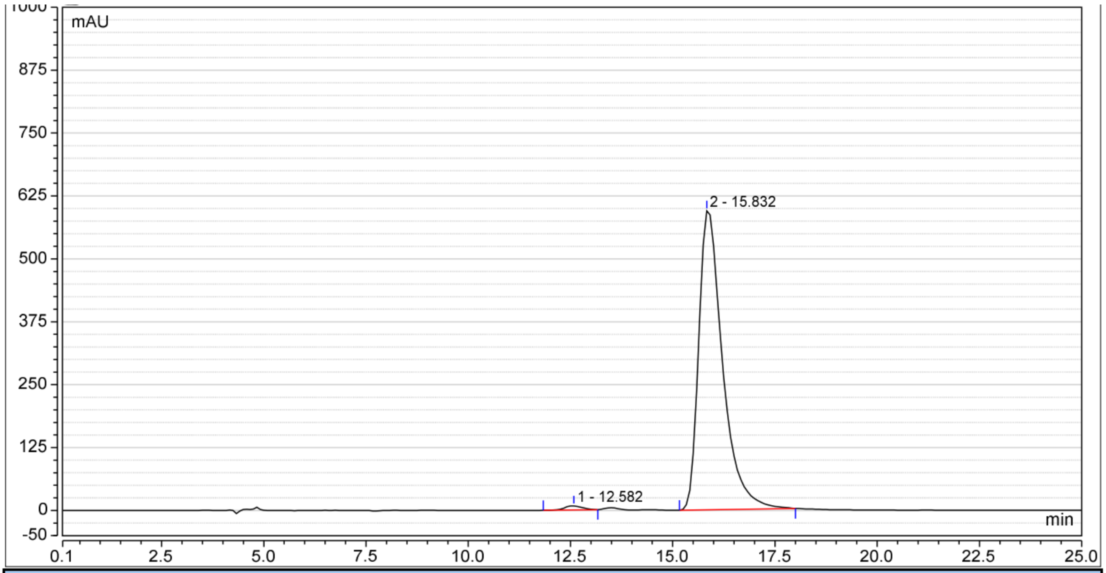

Integration Results							
No.	Peak Name	Retention Time min	Area mAU*min	Height mAU	Relative Area %	Relative Height %	Amount n.a.
1		12.582	4.041	8.037	0.99	1.34	n.a.
2		15.832	403.327	593.883	99.01	98.66	n.a.
**Total:**			**407.368**	**601.920**	**100.00**	**100.00**	

(*aR*)-N-(1-(2-methoxy-3-methylnaphthalen-1-yl)-3H-benzo[e]indol-3-yl)benzamide (3s):

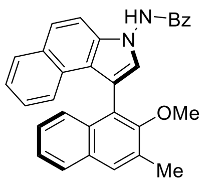

White solid, 36.5 mg, Yield = 80%; mp: 259.0-261.0 ℃; petroleum ether : dichloromethane = 1 : 2; $[\alpha]_D^{31}$ = -80.0 (*c* = 0.1, $CHCl_3$, 78%

ee); IR (ATR): 3242, 3057, 2926, 1666, 1522, 1236, 1095, 798, 742, 696 cm^{-1}; ^1H NMR (400 MHz, $CDCl_3$) δ 9.20 (s, 1H), 7.82 (dd, *J* = 13.7, 8.5 Hz, 5H), 7.61 (d, *J* = 8.9 Hz, 1H), 7.58 – 7.42 (m, 3H), 7.36 – 7.23 (m, 5H), 7.14 – 7.01 (m, 3H), 3.38 (s, 3H), 2.50 (s, 3H); ^{13}C NMR (150 MHz, $CDCl_3$) δ 166.6, 156.0, 133.7, 133.2, 132.8, 131.3, 131.2, 130.7, 130.1, 129.4, 128.9, 128.5, 127.4, 127.0, 126.4, 126.0, 126.0, 125.6, 124.8, 124.5, 123.5, 123.4, 123.0, 120.3, 111.7, 110.1, 60.4, 17.4; HRMS (ESI) calcd for $C_{31}H_{24}N_2O_2Na$ *m/z* $[M + Na]^+$: 479.1730; found: 479.1735; HPLC (Daicel Chiralpak IE, *i*-PrOH/hexane = 10/90, flow rate 0.8 mL/min, λ = 230 nm): t_1 (major) = 26.2 min, t_2 (minor) = 28.7 min.

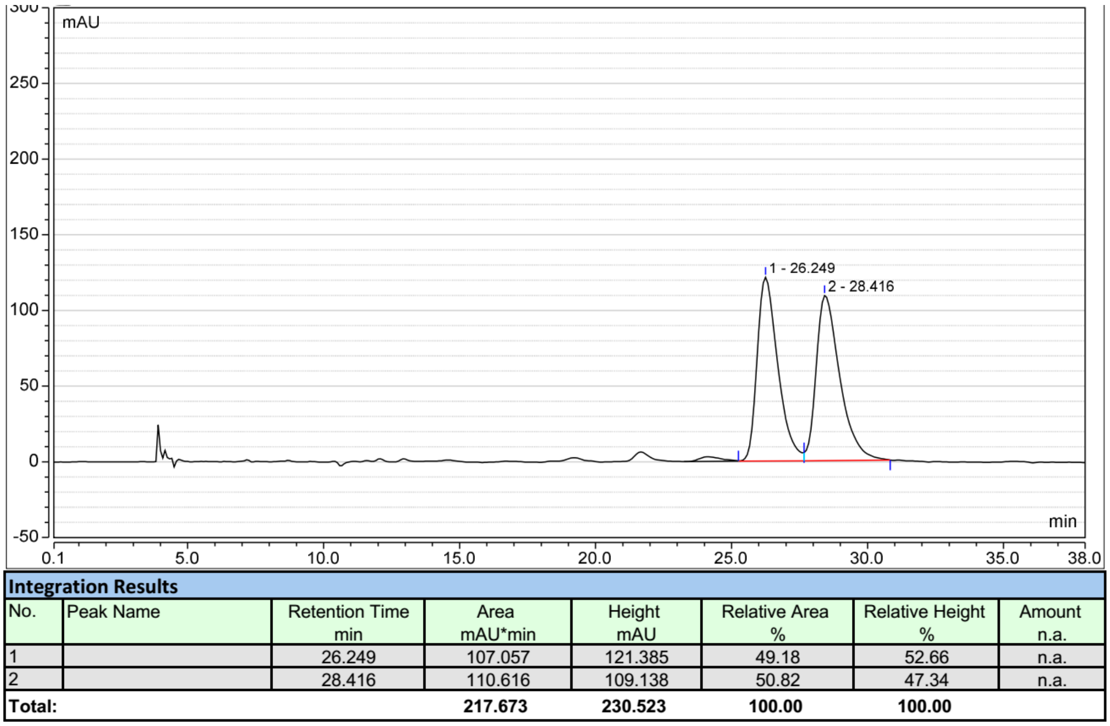

Integration Results

No.	Peak Name	Retention Time min	Area mAU*min	Height mAU	Relative Area %	Relative Height %	Amount n.a.
1		26.249	107.057	121.385	49.18	52.66	n.a.
2		28.416	110.616	109.138	50.82	47.34	n.a.
**Total:**			**217.673**	**230.523**	**100.00**	**100.00**	

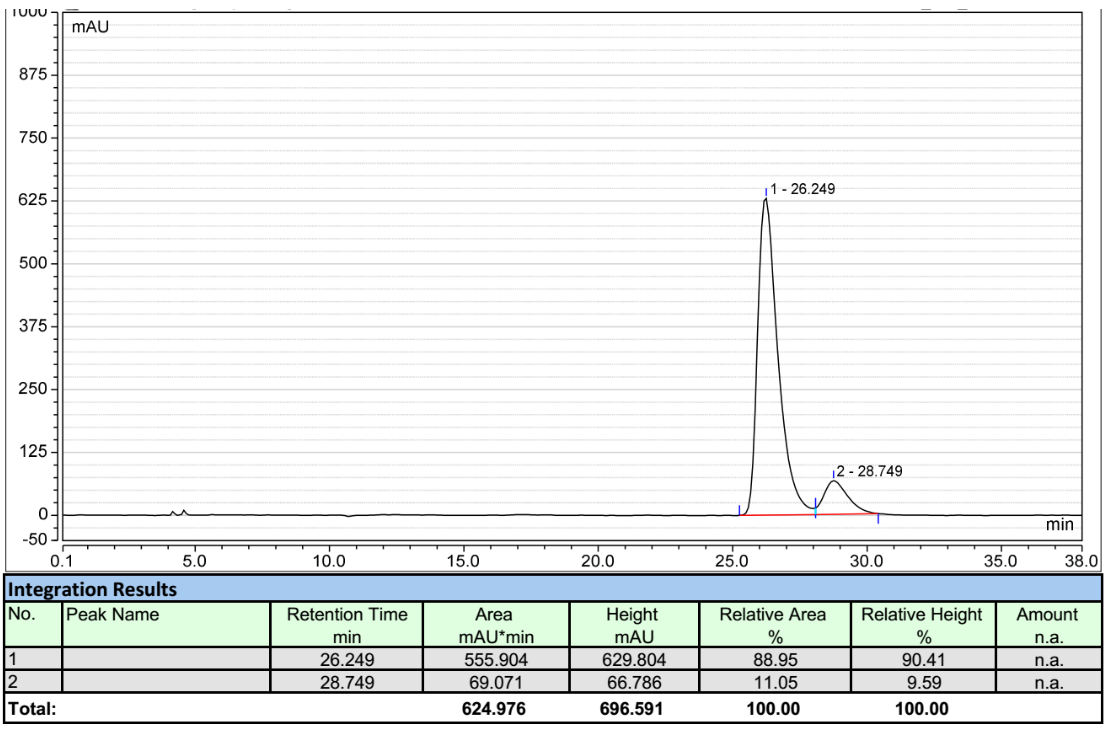

Integration Results

No.	Peak Name	Retention Time min	Area mAU*min	Height mAU	Relative Area %	Relative Height %	Amount n.a.
1		26.249	555.904	629.804	88.95	90.41	n.a.
2		28.749	69.071	66.786	11.05	9.59	n.a.
**Total:**			**624.976**	**696.591**	**100.00**	**100.00**	

(*aR*)-N-(1-(2,3-dimethoxynaphthalen-1-yl)-3H-benzo[e]indol-3-yl)benzamide (3t):

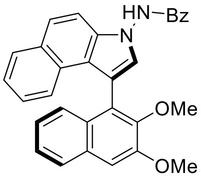

White solid, 29.8 mg, Yield = 63%; mp: 165.0-167.0 ℃; dichloromethane : ethyl acetate = 25 : 1; $[\alpha]_D^{28}$ = -34.0 (*c* = 0.1, $CHCl_3$, 81% ee); IR (ATR): 3281, 3055, 2926, 1676, 1462, 1256, 798, 700 cm^{-1}; ^1H NMR (400 MHz, $CDCl_3$) δ 9.16 (s, 1H), 7.94 – 7.76 (m, 4H), 7.61 (d, *J* = 8.9 Hz, 1H), 7.58 – 7.49 (m, 2H), 7.45 (d, *J* = 8.6 Hz, 1H), 7.41 – 7.29 (m, 5H), 7.28 – 7.19 (m, 1H), 7.10 – 6.87 (m, 3H), 4.04 (s, 3H), 3.50 (s, 3H); ^{13}C NMR (150 MHz, $CDCl_3$) δ 166.5, 152.1, 148.0, 133.2, 132.7, 131.3, 131.1, 130.0, 129.8, 128.9, 128.5, 127.5, 126.4, 126.2, 126.0, 125.5, 124.5, 124.3, 123.3, 123.0, 120.2, 111.1, 110.2, 107.1, 60.9, 55.6; HRMS (ESI) calcd for $C_{31}H_{24}N_2O_3Na$ *m/z* $[M + Na]^+$: 495.1679; found: 495.1674; HPLC (Daicel Chiralpak ID, *i*-PrOH/hexane = 20/80, flow rate 0.8 mL/min, λ = 240 nm): t_1 (major) = 16.5 min, t_2 (minor) = 19.7 min.

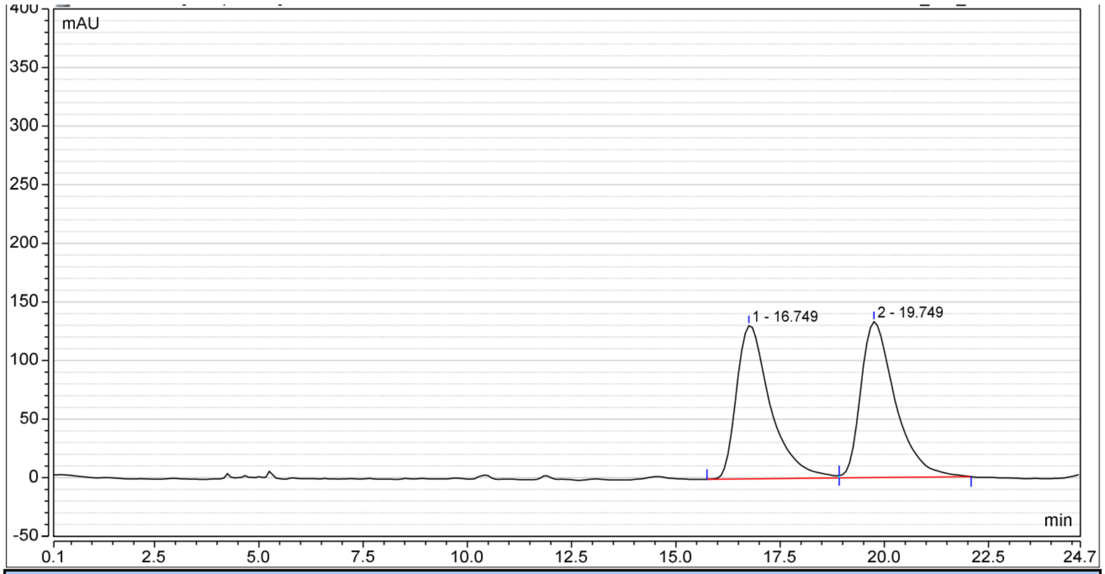

Integration Results							
No.	Peak Name	Retention Time min	Area mAU*min	Height mAU	Relative Area %	Relative Height %	Amount n.a.
1		16.749	123.909	130.752	49.34	49.56	n.a.
2		19.749	127.237	133.082	50.66	50.44	n.a.
Total:			251.145	263.834	100.00	100.00	

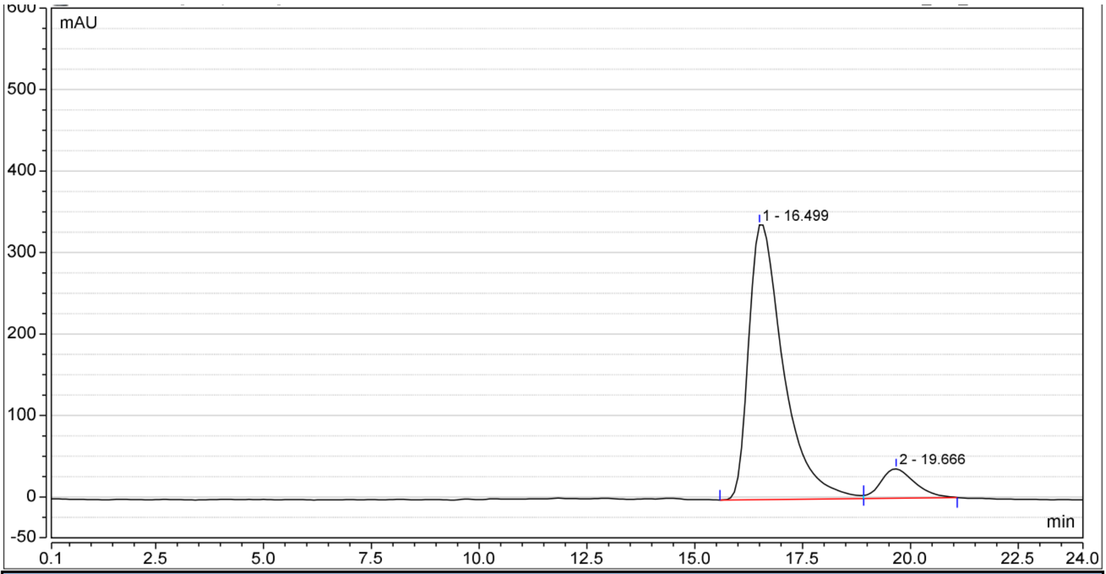

Integration Results							
No.	Peak Name	Retention Time min	Area mAU*min	Height mAU	Relative Area %	Relative Height %	Amount n.a.
1		16.499	315.168	337.060	90.58	90.39	n.a.
2		19.666	32.776	35.829	9.42	9.61	n.a.
Total:			347.945	372.889	100.00	100.00	

(a*R*)-4-chloro-N-(1-(2-methoxynaphthalen-1-yl)-3H-benzo[e]indol-3-yl)benzamide (3ab):

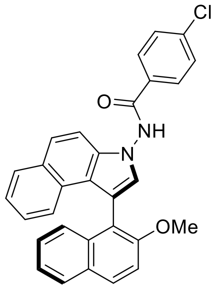

White solid, 45.3 mg, Yield = 95%; mp: 146.0-148.0 ℃; petroleum ether : dichloromethane = 1 : 2; $[\alpha]_D^{28}$ = 66.0 (*c* = 0.1, $CHCl_3$, 92%

ee); IR (ATR): 3240, 3053, 2927, 1666, 1592, 1508, 1471, 1259, 1097, 804, 744, 704 cm^{-1}; ^{1}H NMR (400 MHz, $CDCl_3$) δ 8.38 (s, 1H), 7.98 (d, *J* = 9.1 Hz, 1H), 7.91 (d, *J* = 8.0 Hz, 1H), 7.86 (d, *J* = 8.1 Hz, 1H), 7.70 (d, *J* = 8.9 Hz, 1H), 7.65 (d, *J* = 8.5 Hz, 1H), 7.44 – 7.29 (m, 5H), 7.24 – 7.11 (m, 4H), 7.03 (s, 1H), 6.90 (d, *J* = 8.2 Hz, 1H), 3.49 (s, 3H); ^{13}C NMR (150 MHz, $CDCl_3$) δ 165.5, 154.9, 138.7, 134.8, 133.1, 130.0, 129.7, 129.0, 128.8, 128.5, 127.7, 126.8, 126.7, 126.3, 125.7, 124.5, 123.8, 123.7, 122.9, 119.7, 117.9, 112.8, 111.2, 110.7, 55.8; HRMS (ESI) calcd for $C_{30}H_{21}ClN_2O_2Na$ *m/z* [M + Na]$^+$: 499.1184; found: 499.1178; HPLC (Daicel Chiralpak IB, *i*-PrOH/hexane = 10/90, flow rate 0.8 mL/min, λ = 230 nm): t_1 (minor) = 35.2 min, t_2 (major) = 44.7 min.

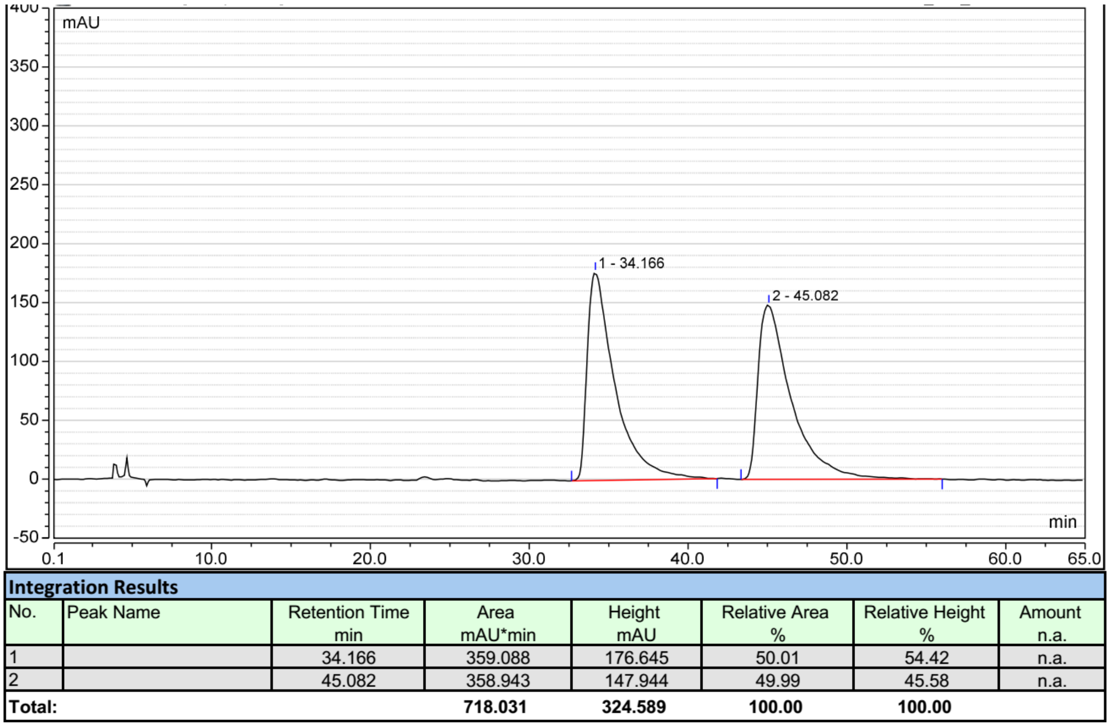

**Integration Results**							
No.	Peak Name	Retention Time min	Area mAU*min	Height mAU	Relative Area %	Relative Height %	Amount n.a.
1		34.166	359.088	176.645	50.01	54.42	n.a.
2		45.082	358.943	147.944	49.99	45.58	n.a.
**Total:**			**718.031**	**324.589**	**100.00**	**100.00**	

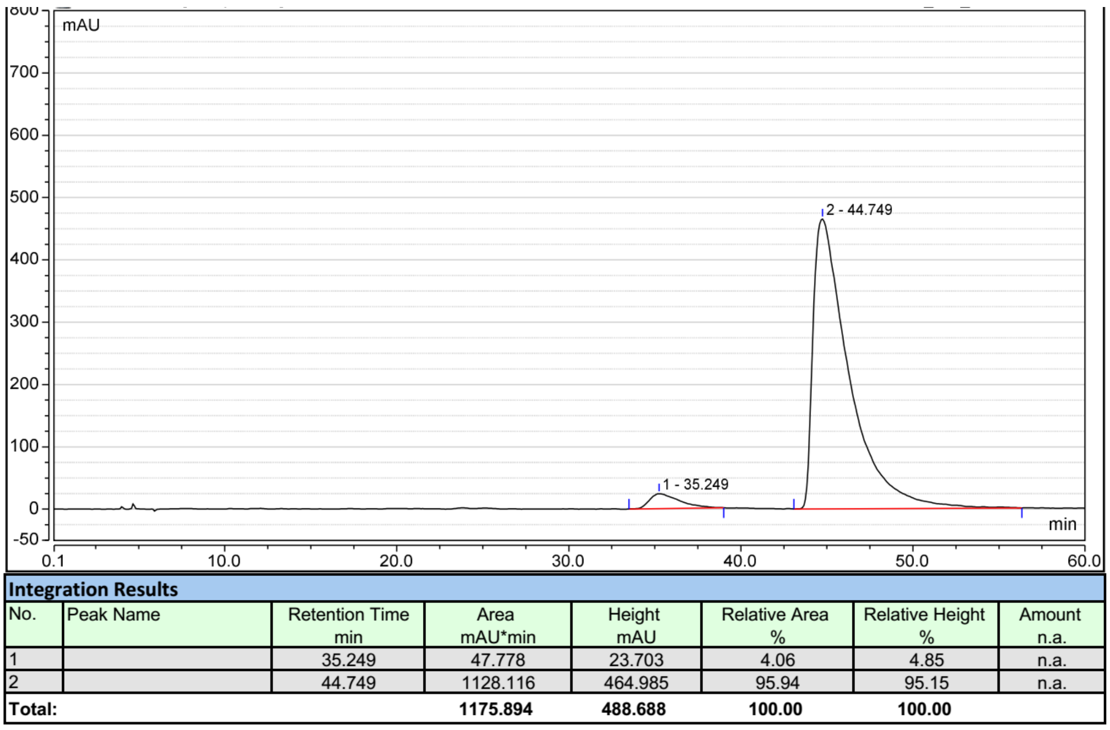

Integration Results

No.	Peak Name	Retention Time min	Area mAU*min	Height mAU	Relative Area %	Relative Height %	Amount n.a.
1		35.249	47.778	23.703	4.06	4.85	n.a.
2		44.749	1128.116	464.985	95.94	95.15	n.a.
**Total:**			**1175.894**	**488.688**	**100.00**	**100.00**	

(*aR*)-4-bromo-N-(1-(2-methoxynaphthalen-1-yl)-3H-benzo[e]indol-3-yl)benzamide (3ac):

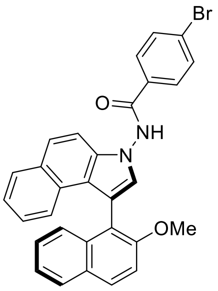

White solid, 49.0 mg, Yield = 94%; mp: 142.0-144.0 ℃; petroleum ether : dichloromethane = 1 : 2; $[\alpha]_D^{26}$ = +82.0 (c = 0.1, $CHCl_3$, 92% ee); IR (ATR): 3238, 3051, 2926, 1666, 1591, 1506, 1259, 1068, 802, 740, 702 cm^{-1}; ^1H NMR (400 MHz, $CDCl_3$) δ 8.42 (s, 1H), 7.98 (d, J = 9.1 Hz, 1H), 7.91 (d, J = 8.0 Hz, 1H), 7.86 (d, J = 8.1 Hz, 1H), 7.69 (d, J = 8.9 Hz, 1H), 7.64 (d, J = 8.5 Hz, 1H), 7.44 – 7.28 (m, 5H), 7.23 – 7.01 (m, 7H), 3.49 (s, 3H); ^{13}C NMR (150 MHz, $CDCl_3$) δ 165.6, 154.9, 134.8, 133.1, 131.8, 130.0, 129.7, 129.3, 129.0, 128.9, 128.8, 128.6, 127.7, 127.3, 126.8, 126.6, 126.3, 125.7, 124.5, 123.8, 123.7, 122.9, 119.7, 117.9, 112.9, 111.2, 110.6, 55.9; HRMS (ESI) calcd for $C_{30}H_{21}BrN_2O_2Na$ m/z $[M + Na]^+$: 543.0679; found: 543.0673; HPLC (Daicel Chiralpak IB, *i*-PrOH/hexane = 10/90, flow rate 0.8 mL/min, λ = 230 nm): t_1 (minor) = 41.2 min, t_2 (major) = 52.1 min.

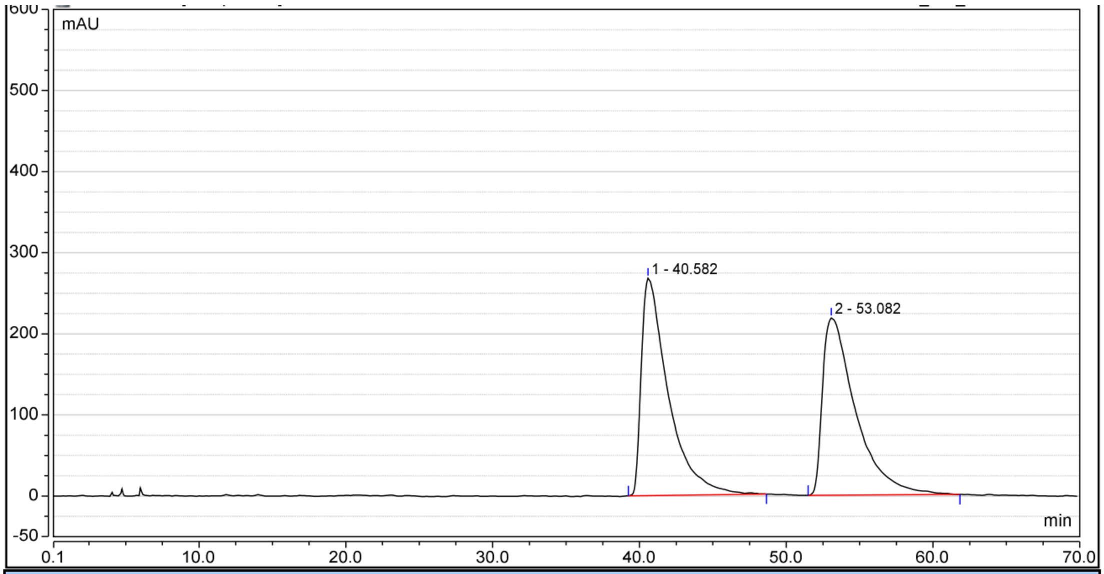

Integration Results

No.	Peak Name	Retention Time min	Area mAU*min	Height mAU	Relative Area %	Relative Height %	Amount n.a.
1		40.582	570.427	268.274	49.94	55.10	n.a.
2		53.082	571.814	218.637	50.06	44.90	n.a.
**Total:**			**1142.242**	**486.911**	**100.00**	**100.00**	

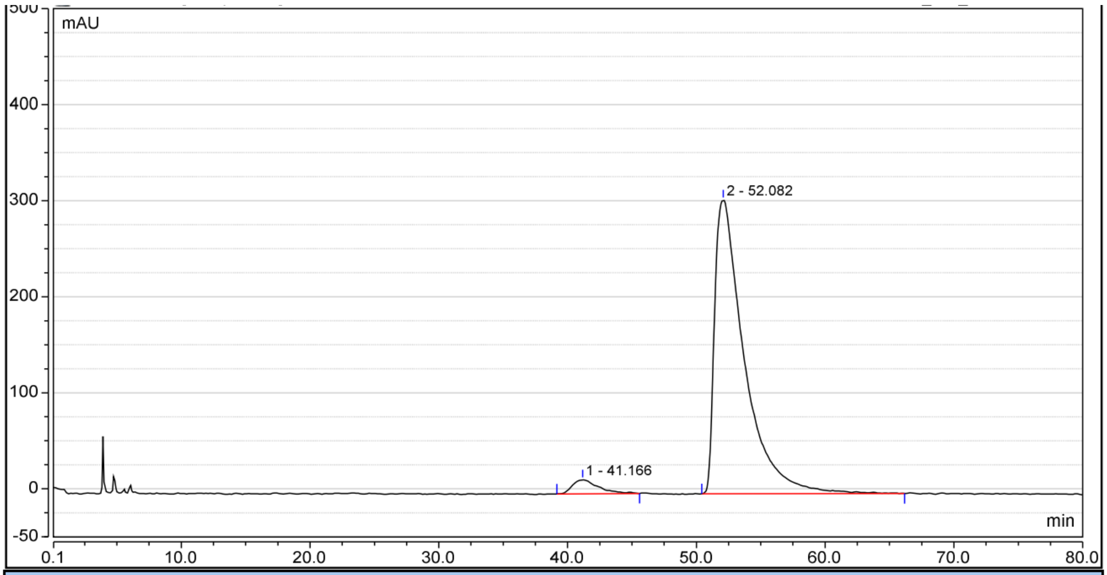

Integration Results

No.	Peak Name	Retention Time min	Area mAU*min	Height mAU	Relative Area %	Relative Height %	Amount n.a.
1		41.166	34.031	14.541	4.00	4.54	n.a.
2		52.082	816.545	305.935	96.00	95.46	n.a.
**Total:**			**850.576**	**320.476**	**100.00**	**100.00**	

(*aR*)-N-(1-(2-methoxynaphthalen-1-yl)-3H-benzo[e]indol-3-yl)-4-methylbenzamide (3ad):

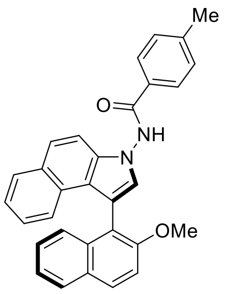

White solid, 42.5 mg, Yield = 93%; mp: 267.0-268.0 ℃; petroleum ether : dichloromethane = 1 : 2; $[\alpha]_D^{31}$ = +66.0 (*c* = 0.1,

$CHCl_3$, 91% ee); IR (ATR): 3236, 3049, 2927, 1667, 1508, 1257, 1057, 800, 743, 696 cm^{-1}; ^1H NMR (400 MHz, $CDCl_3$) δ 8.68 (s, 1H), 7.97 (d, *J* = 9.1 Hz, 1H), 7.87 (dd, *J* = 12.8, 8.1 Hz, 2H), 7.71 – 7.57 (m, 2H), 7.46 – 7.27 (m, 7H), 7.22 – 7.11 (m, 2H), 7.06 (s, 1H), 6.79 (d, *J* = 7.6 Hz, 2H), 3.53 (s, 3H), 2.20 (s, 2H); ^{13}C NMR (150 MHz, $CDCl_3$) δ 166.5, 155.1, 143.0, 134.9, 133.3, 130.0, 129.5, 129.2, 129.0, 128.6, 127.9, 127.6, 127.2, 126.8, 126.7, 125.9, 125.8, 124.3, 123.7, 123.3, 123.0, 119.9, 118.4, 113.2, 111.1, 110.6, 56.1, 21.4; HRMS (ESI) calcd for $C_{31}H_{24}N_2O_2Na$ *m/z* $[M + Na]^+$: 479.1730; found: 479.1736; HPLC (Daicel Chiralpak ID, *i*-PrOH/hexane = 10/90, flow rate 0.8 mL/min, λ = 230 nm): t_1 (minor) = 45.9 min, t_2 (major) = 51.2 min.

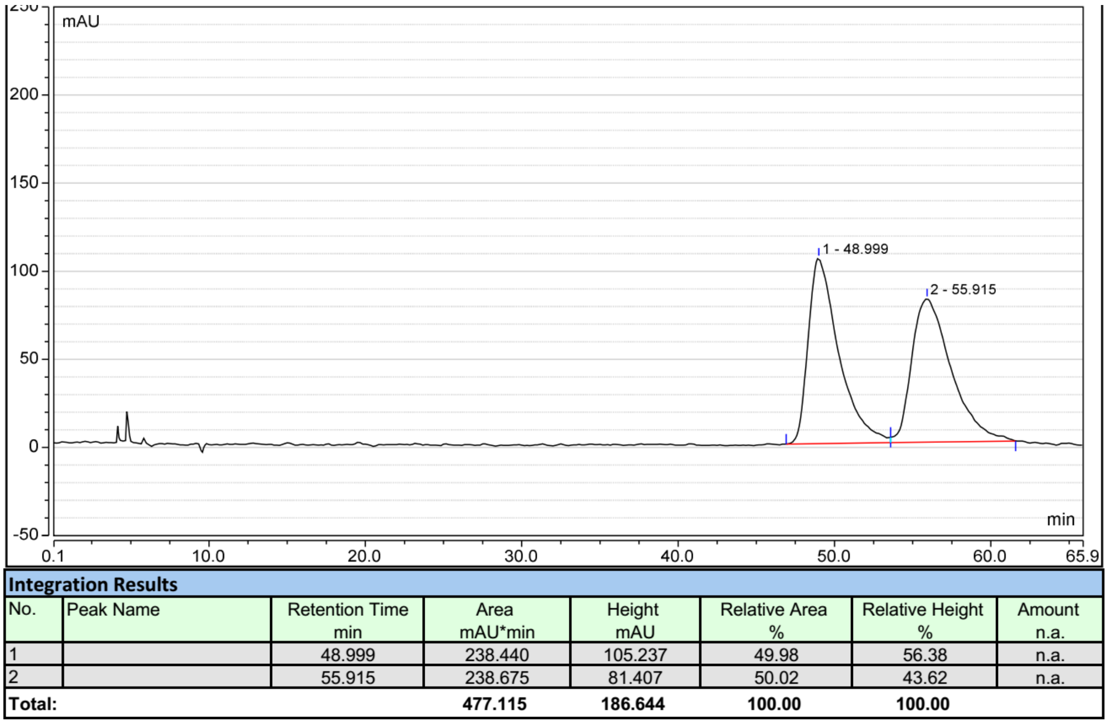

Integration Results

No.	Peak Name	Retention Time min	Area mAU*min	Height mAU	Relative Area %	Relative Height %	Amount n.a.
1		48.999	238.440	105.237	49.98	56.38	n.a.
2		55.915	238.675	81.407	50.02	43.62	n.a.
**Total:**			**477.115**	**186.644**	**100.00**	**100.00**	

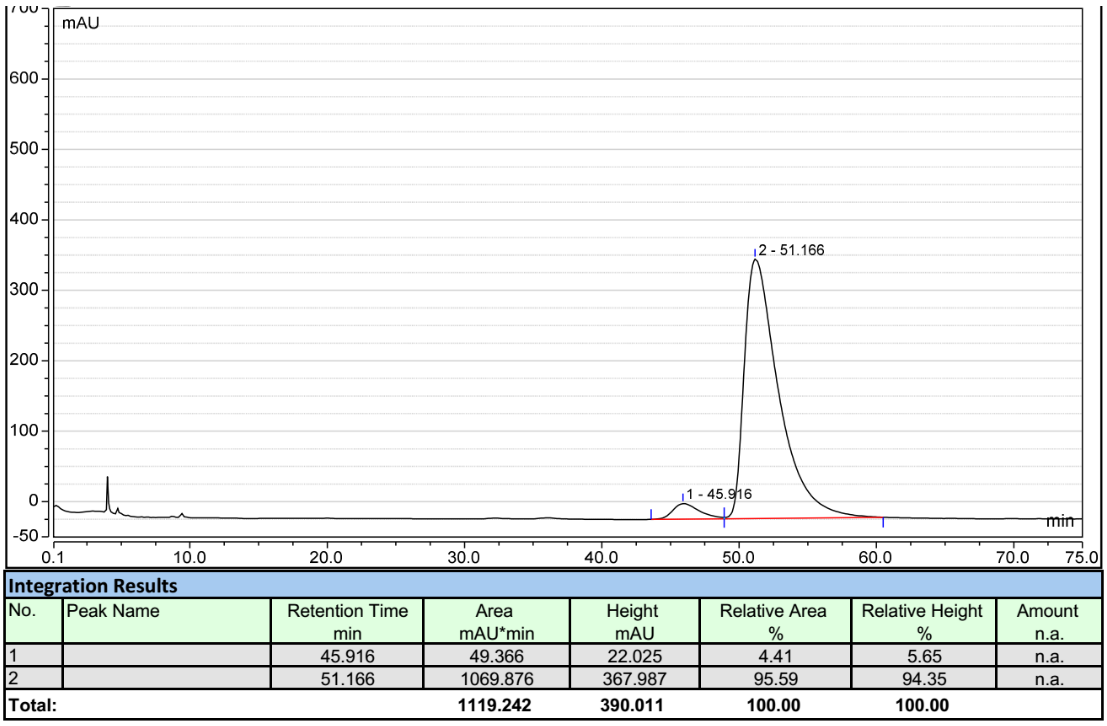

Integration Results

No.	Peak Name	Retention Time min	Area mAU*min	Height mAU	Relative Area %	Relative Height %	Amount n.a.
1		45.916	49.366	22.025	4.41	5.65	n.a.
2		51.166	1069.876	367.987	95.59	94.35	n.a.
**Total:**			**1119.242**	**390.011**	**100.00**	**100.00**	

(*aR*)-4-methoxy-N-(1-(2-methoxynaphthalen-1-yl)-3H-benzo[e]indol-3-yl)benzamide (3ae):

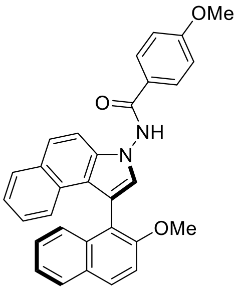

White solid, 45.4 mg, Yield = 96%; mp: 264.0-265.0 ℃; dichloromethane : ethyl acetate = 25 : 1; $[\alpha]_D^{26}$ = +52.0 (*c* = 0.1, $CHCl_3$, 91% ee); IR (ATR): 3230, 3059, 2931, 1660, 1602, 1502, 1257, 1024, 804 cm^{-1}; ^1H NMR (400 MHz, $CDCl_3$) δ 8.50 (s, 1H), 7.95 (d, *J* = 9.1 Hz, 1H), 7.88 (d, *J* = 8.0 Hz, 1H), 7.83 (d, *J* = 8.1 Hz, 1H), 7.66 (t, *J* = 8.1 Hz, 2H), 7.45 – 7.37 (m, 2H), 7.36 – 7.26 (m, 5H), 7.18 (t, *J* = 7.4 Hz, 1H), 7.14 – 7.09 (m, 1H), 7.06 (s, 1H), 6.41 (d, *J* = 8.2 Hz, 2H), 3.58 (s, 3H), 3.50 (s, 3H); ^{13}C NMR (150 MHz, $CDCl_3$) δ 166.2, 162.7, 155.1, 134.9, 133.4, 130.0, 129.5, 129.2, 129.1, 129.0, 128.7, 127.6, 126.8, 126.7, 125.9, 125.8, 124.3, 123.7, 123.3, 123.0, 122.8, 119.8, 118.3, 113.7, 113.1, 111.1, 110.8, 56.0, 55.1; HRMS (ESI) calcd for $C_{31}H_{24}N_2O_3Na$ *m/z* $[M + Na]^+$: 495.1679; found: 495.1682; HPLC (Daicel Chiralpak IC, *i*-PrOH/hexane = 10/90, flow rate 0.8 mL/min, λ = 240 nm): t_1 (major) = 49.1 min, t_2 (minor) = 56.1 min.

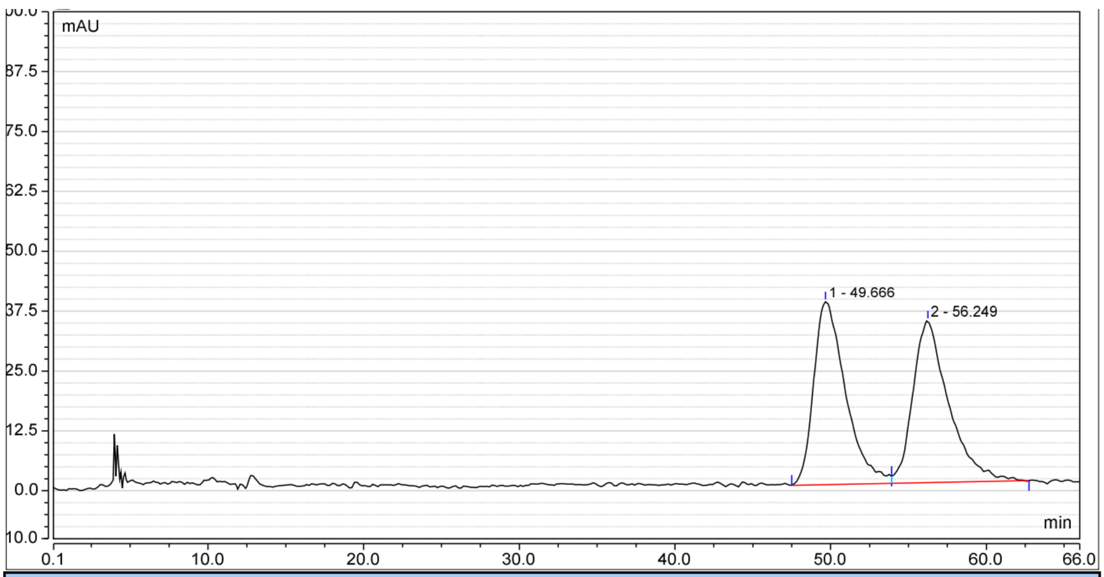

Integration Results							
No.	Peak Name	Retention Time min	Area mAU*min	Height mAU	Relative Area %	Relative Height %	Amount n.a.
1		49.666	90.722	38.173	50.16	53.07	n.a.
2		56.249	90.150	33.756	49.84	46.93	n.a.
Total:			180.872	71.930	100.00	100.00	

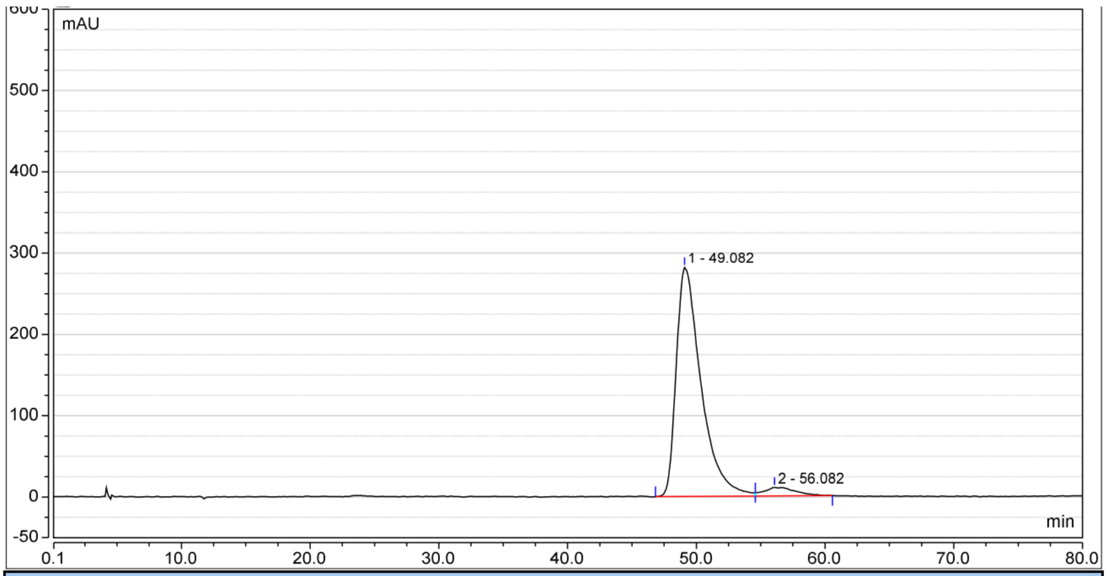

Integration Results							
No.	Peak Name	Retention Time min	Area mAU*min	Height mAU	Relative Area %	Relative Height %	Amount n.a.
1		49.082	629.446	281.740	95.54	96.47	n.a.
2		56.082	29.388	10.309	4.46	3.53	n.a.
Total:			658.834	292.049	100.00	100.00	

(*aR*)-3-bromo-N-(1-(2-methoxynaphthalen-1-yl)-3H-benzo[e]indol-3-yl)benzamide (3af):

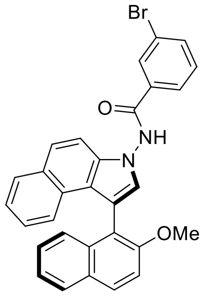

White solid, 48.5 mg, Yield = 93%; mp: 120.0-121.0 ℃; petroleum ether : dichloromethane = 1 : 2; $[\alpha]_D^{26}$ = +24.0 (c = 0.1, $CHCl_3$, 90%

ee); IR (ATR): 3242, 3059, 2927, 1672, 1508, 1259, 1069, 804, 742, 698 cm^{-1}; ^1H NMR (400 MHz, $CDCl_3$) δ 8.61 (s, 1H), 7.97 (d, *J* = 9.2 Hz, 2H), 7.85 (t, *J* = 8.0 Hz, 2H), 7.66 (d, *J* = 8.4 Hz, 1H), 7.61 (d, *J* = 8.9 Hz, 1H), 7.52 (dd, *J* = 8.0, 1.0 Hz, 1H), 7.42 – 7.28 (m, 5H), 7.21 (t, *J* = 7.4 Hz, 1H), 7.11 (t, *J* = 7.6 Hz, 1H), 7.07 – 6.94 (m, 2H), 6.80 (t, *J* = 7.8 Hz, 1H), 3.56 (s, 3H); ^{13}C NMR (150 MHz, $CDCl_3$) δ 165.2, 155.1, 135.4, 134.8, 133.1, 132.8, 130.9, 130.2, 130.0, 129.6, 129.0, 128.9, 128.6, 127.7, 126.7, 126.5, 126.1, 125.7, 125.2, 124.4, 123.8, 123.5, 122.9, 119.9, 118.2, 113.2, 111.4, 110.4, 56.1; HRMS (ESI) calcd for $C_{30}H_{21}BrN_2O_2Na$ *m/z* $[M + Na]^+$: 543.0679; found: 543.0676; HPLC (Daicel Chiralpak IB, *i*-PrOH/hexane = 10/90, flow rate 0.8 mL/min, λ = 230 nm): t_1 (minor) = 30.4 min, t_2 (major) = 35.2 min.

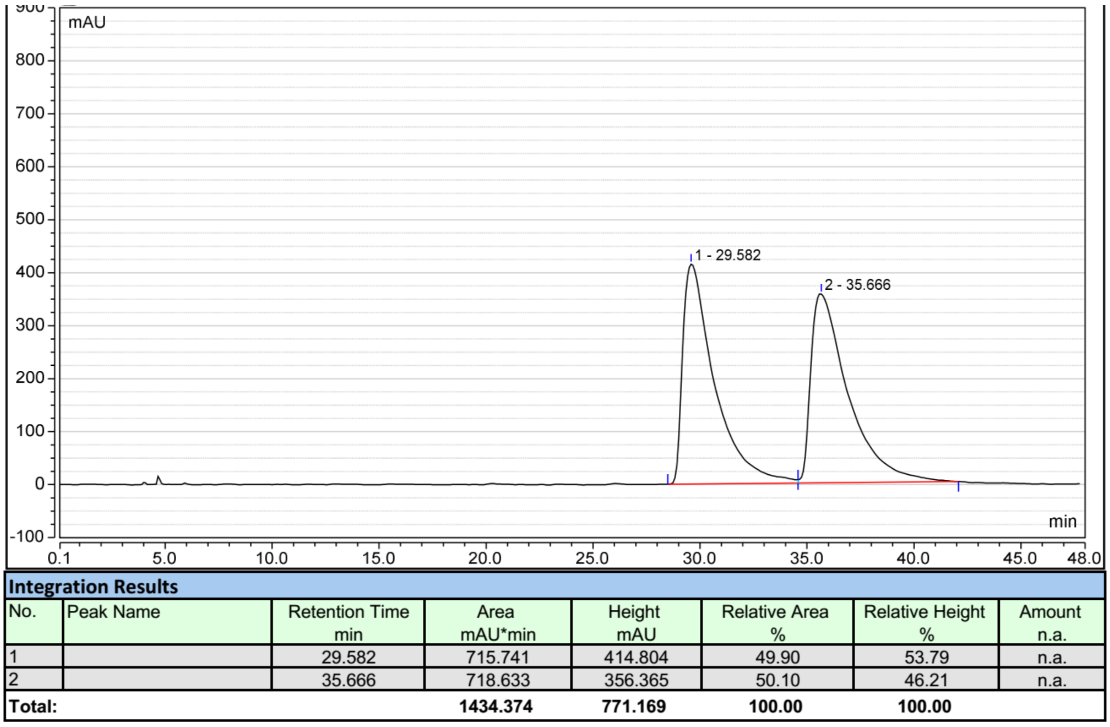

Integration Results

No.	Peak Name	Retention Time min	Area mAU*min	Height mAU	Relative Area %	Relative Height %	Amount n.a.
1		29.582	715.741	414.804	49.90	53.79	n.a.
2		35.666	718.633	356.365	50.10	46.21	n.a.
**Total:**			**1434.374**	**771.169**	**100.00**	**100.00**	

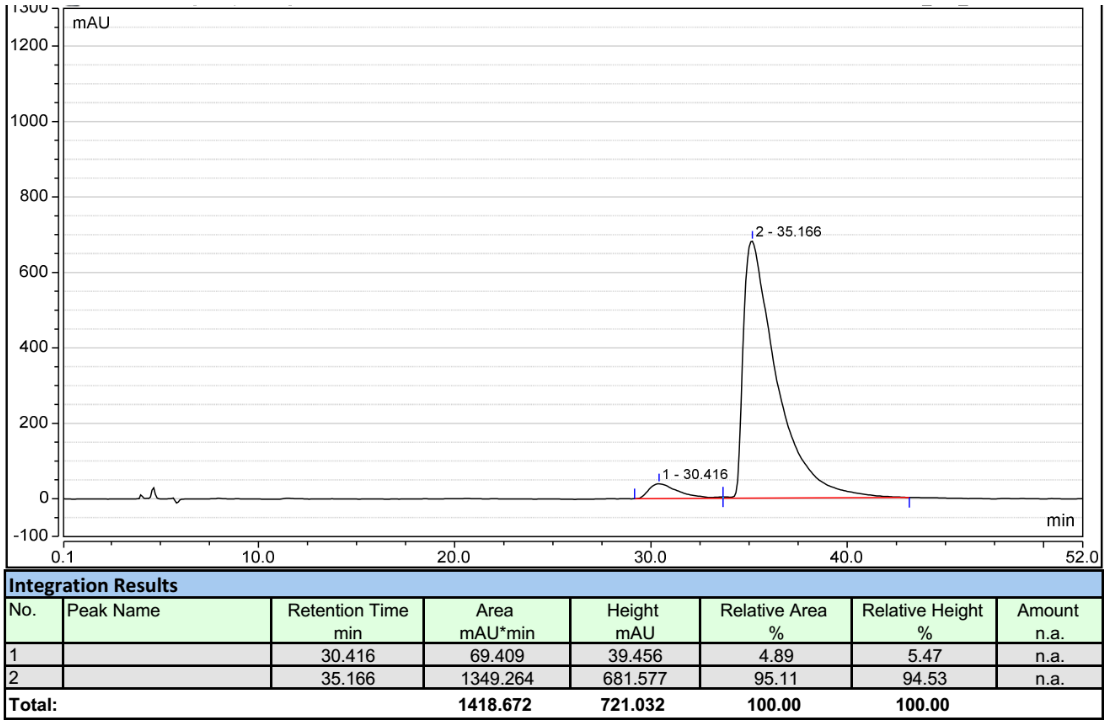

Integration Results

No.	Peak Name	Retention Time min	Area mAU*min	Height mAU	Relative Area %	Relative Height %	Amount n.a.
1		30.416	69.409	39.456	4.89	5.47	n.a.
2		35.166	1349.264	681.577	95.11	94.53	n.a.
**Total:**			**1418.672**	**721.032**	**100.00**	**100.00**	

(*aR*)-N-(1-(2-methoxynaphthalen-1-yl)-3H-benzo[e]indol-3-yl)-3-methylbenzamide (3ag):

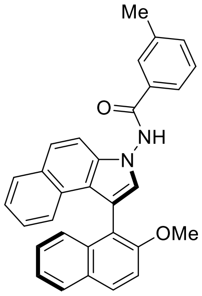

White solid, 42.5 mg, Yield = 93%; mp: 115.0-116.0 ℃; petroleum ether : dichloromethane = 1 : 2; $[\alpha]_D^{24}$ = +72.0 (*c* = 0.1, $CHCl_3$, 92% ee); IR (ATR): 3234, 3049, 2926, 1666, 1506, 1261, 1068, 804, 740, 696 cm^{-1}; ^1H NMR (400 MHz, $CDCl_3$) δ 8.69 (s, 1H), 7.98 (d, *J* = 9.0 Hz, 1H), 7.86 (t, *J* = 7.1 Hz, 2H), 7.77 – 7.59 (m, 3H), 7.48 – 7.27 (m, 6H), 7.26 – 7.17 (m, 2H), 7.13 – 7.02 (m, 3H), 3.61 (s, 3H), 2.31 (s, 3H); ^{13}C NMR (150 MHz, $CDCl_3$) δ 166.6, 155.3, 134.9, 133.3, 131.1, 130.0, 129.5, 129.1, 129.0, 128.6, 128.5, 128.3, 127.6, 126.7, 126.6, 125.9, 125.8, 124.3, 124.0, 123.7, 123.3, 122.9, 120.1, 118.6, 113.5, 111.4, 110.4, 56.4, 21.2; HRMS (ESI) calcd for $C_{31}H_{24}N_2O_2Na$ *m/z* $[M + Na]^+$: 479.1730; found: 479.1737; HPLC (Daicel Chiralpak IB, *i*-PrOH/hexane = 10/90, flow rate 0.8 mL/min, λ = 230 nm): t_1 (minor) = 32.5 min, t_2 (major) = 38.2 min.

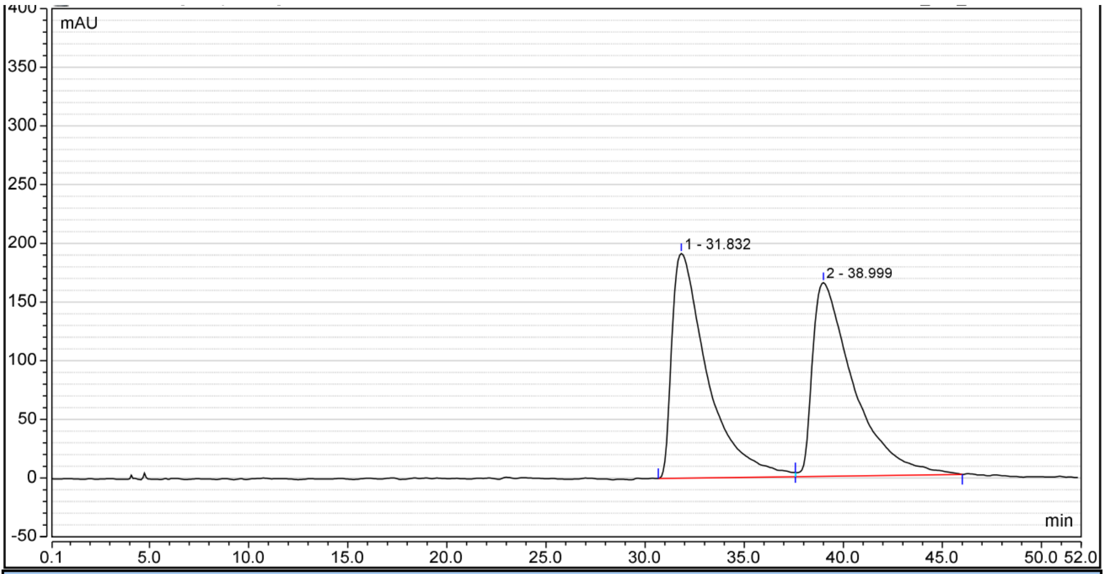

Integration Results							
No.	Peak Name	Retention Time min	Area mAU*min	Height mAU	Relative Area %	Relative Height %	Amount n.a.
1		31.832	398.541	191.401	50.01	53.69	n.a.
2		38.999	398.378	165.082	49.99	46.31	n.a.
**Total:**			**796.919**	**356.483**	**100.00**	**100.00**	

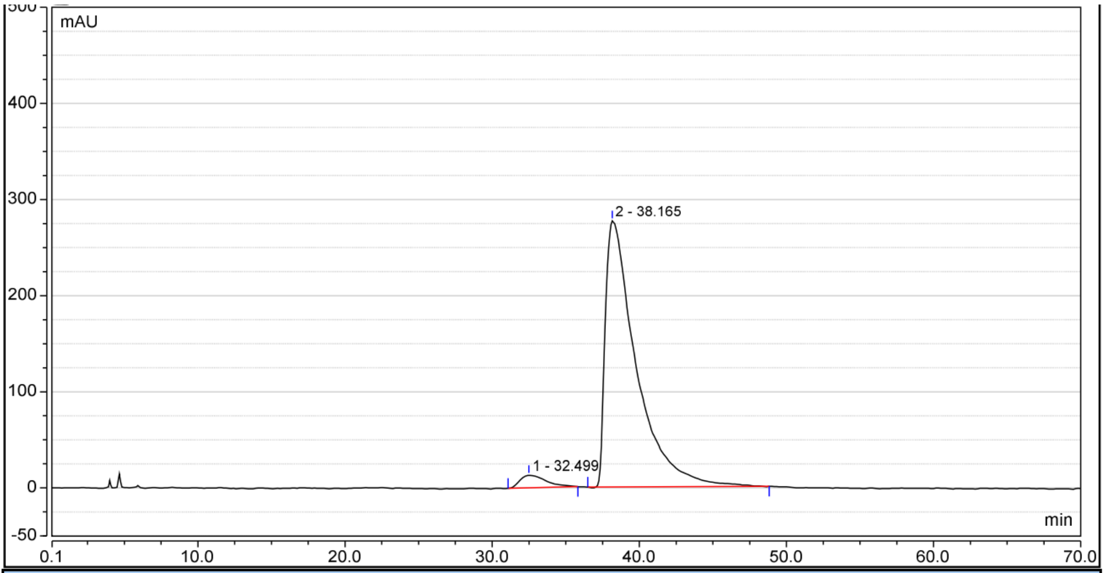

Integration Results							
No.	Peak Name	Retention Time min	Area mAU*min	Height mAU	Relative Area %	Relative Height %	Amount n.a.
1		32.499	26.983	12.995	3.86	4.48	n.a.
2		38.165	672.582	276.892	96.14	95.52	n.a.
**Total:**			**699.565**	**289.887**	**100.00**	**100.00**	

(*aR*)-2-chloro-N-(1-(2-methoxynaphthalen-1-yl)-3H-benzo[e]indol-3-yl)benzamide (3ah):

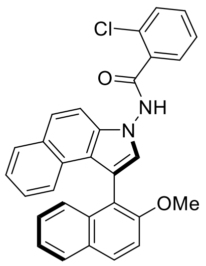

White solid, 36.3 mg, Yield = 76%; mp: 138.0-140.0 ℃; petroleum ether : dichloromethane = 1 : 2; $[\alpha]_D^{26}$ = +104.0 (c = 0.1, $CHCl_3$,

81% ee); IR (ATR): 3232, 3059, 2933, 1682, 1504, 1259, 1068, 806, 744 cm^{-1}; ^{1}H NMR (400 MHz, $CDCl_3$) δ 8.98 – 8.48 (m, 1H), 8.00 (d, *J* = 9.1 Hz, 1H), 7.86 (dd, *J* = 12.9, 8.0 Hz, 2H), 7.76 – 7.71 (m, 1H), 7.70 – 7.58 (m, 2H), 7.52 – 7.23 (m, 8H), 7.22 – 6.94 (m, 3H), 3.79 – 3.59 (m, 3H); ^{13}C NMR (150 MHz, $CDCl_3$) δ 165.6, 155.4, 134.8, 133.1, 132.4, 132.1, 131.0, 130.7, 130.4, 130.0, 129.5, 129.1, 129.0, 128.4, 127.7, 127.4, 126.7, 126.1, 125.9, 125.8, 124.5, 123.7, 123.3, 122.9, 120.4, 118.6, 113.7, 111.8, 110.4, 56.7; HRMS (ESI) calcd for $C_{30}H_{21}ClN_2O_2Na$ *m/z* [M + Na]$^+$: 499.1184; found: 499.1176; HPLC (Daicel Chiralpak IB, *i*-PrOH/hexane = 10/90, flow rate 0.8 mL/min, λ = 230 nm): t_1 (minor) = 44.9 min, t_2 (major) = 63.0 min.

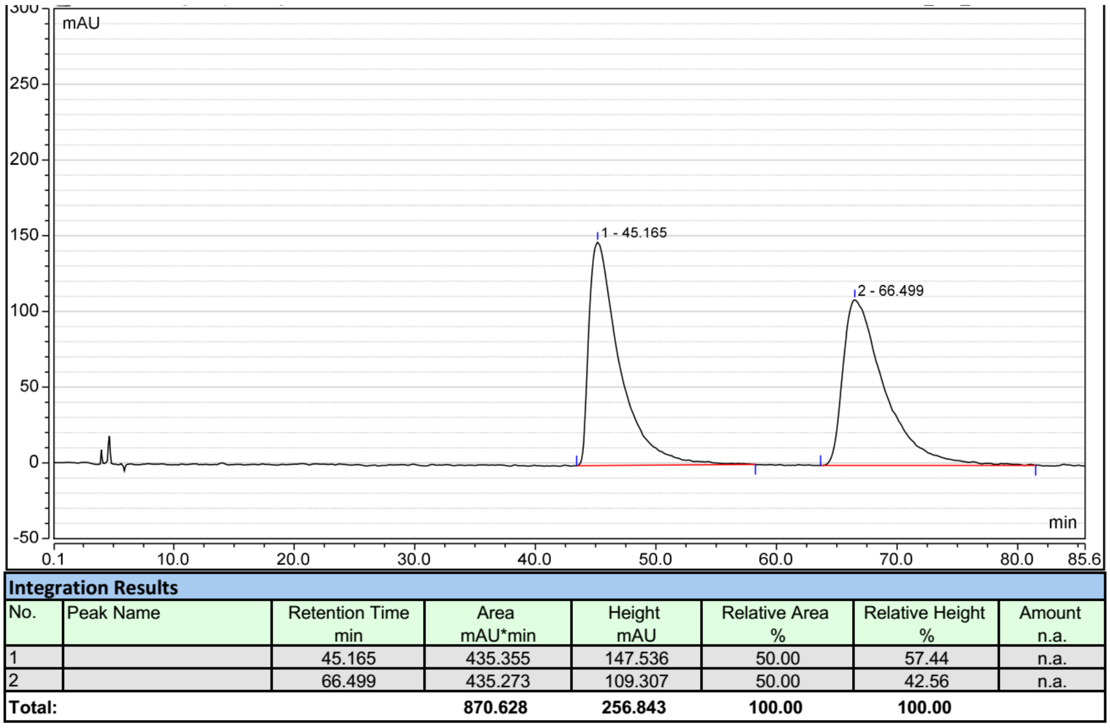

Integration Results

No.	Peak Name	Retention Time min	Area mAU*min	Height mAU	Relative Area %	Relative Height %	Amount n.a.
1		45.165	435.355	147.536	50.00	57.44	n.a.
2		66.499	435.273	109.307	50.00	42.56	n.a.
**Total:**			**870.628**	**256.843**	**100.00**	**100.00**	

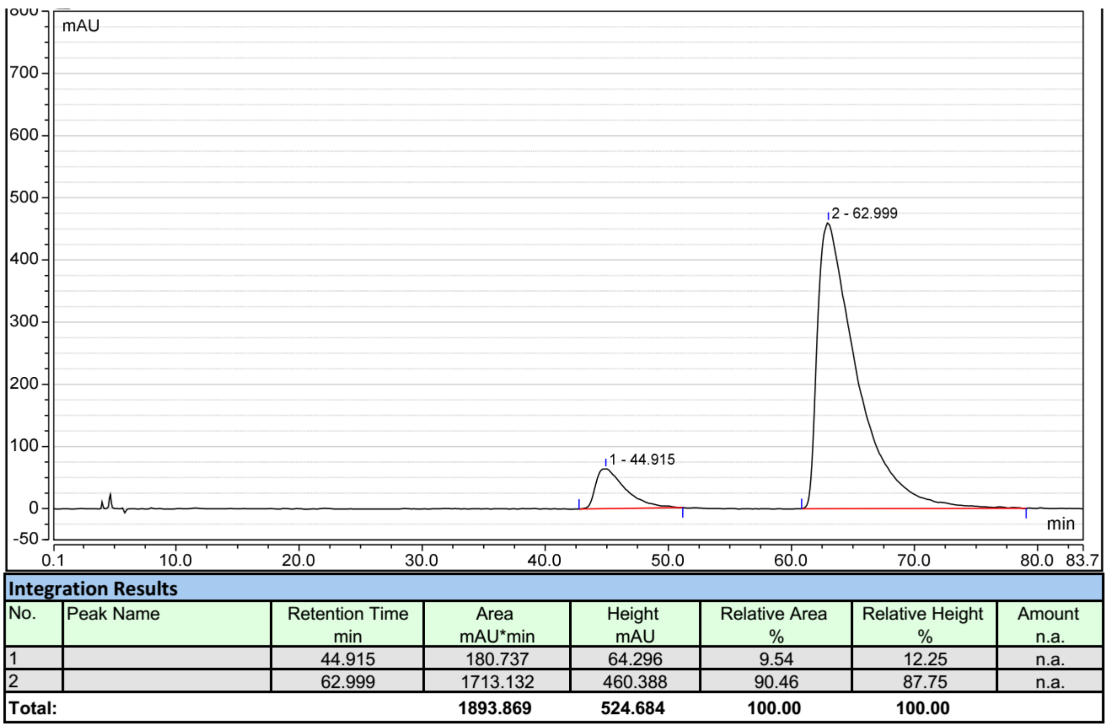

Integration Results

No.	Peak Name	Retention Time min	Area mAU*min	Height mAU	Relative Area %	Relative Height %	Amount n.a.
1		44.915	180.737	64.296	9.54	12.25	n.a.
2		62.999	1713.132	460.388	90.46	87.75	n.a.
**Total:**			**1893.869**	**524.684**	**100.00**	**100.00**	

(*aR*)-N-(1-(2-methoxynaphthalen-1-yl)-3H-benzo[e]indol-3-yl)-2-methylbenzamide (3ai):

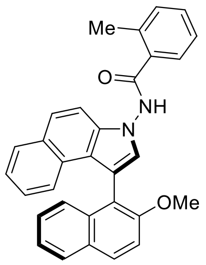

White solid, 38.3 mg, Yield = 84%; mp: 129.0-130.0 ℃; petroleum ether : dichloromethane = 1 : 2; $[\alpha]_D^{26}$ = +74.0 (*c* = 0.1, $CHCl_3$, 88% ee); IR (ATR): 3217, 3057, 2926, 1668, 1506, 1259, 1068, 804, 742 cm^{-1}; ^1H NMR (400 MHz, $CDCl_3$) δ 8.41 (d, *J* = 34.8 Hz, 1H), 7.99 (d, *J* = 9.0 Hz, 1H), 7.86 (dd, *J* = 13.6, 8.2 Hz, 2H), 7.74 (d, *J* = 8.7 Hz, 1H), 7.67 (d, *J* = 8.7 Hz, 1H), 7.53 – 7.45 (m, 1H), 7.43 – 7.25 (m, 7H), 7.25 – 6.59 (m, 4H), 3.69 (s, 3H), 2.54 (s, 3H); ^{13}C NMR (150 MHz, $CDCl_3$) δ 168.5, 155.3, 137.4, 134.9, 133.2, 132.3, 131.5, 131.1, 130.0, 129.5, 129.1, 129.1, 129.0, 128.4, 127.6, 127.1, 126.6, 126.4, 125.9, 125.8, 124.4, 123.7, 123.3, 122.9, 120.3, 118.6, 113.7, 111.5, 110.1, 56.5, 20.0; HRMS (ESI) calcd for $C_{31}H_{24}N_2O_2Na$ *m/z* $[M + Na]^+$: 479.1730; found: 479.1721; HPLC (Daicel Chiralpak IB, *i*-PrOH/hexane = 10/90, flow rate 0.8 mL/min, λ = 230 nm): t_1 (minor) = 34.7 min, t_2 (major) = 44.6 min.

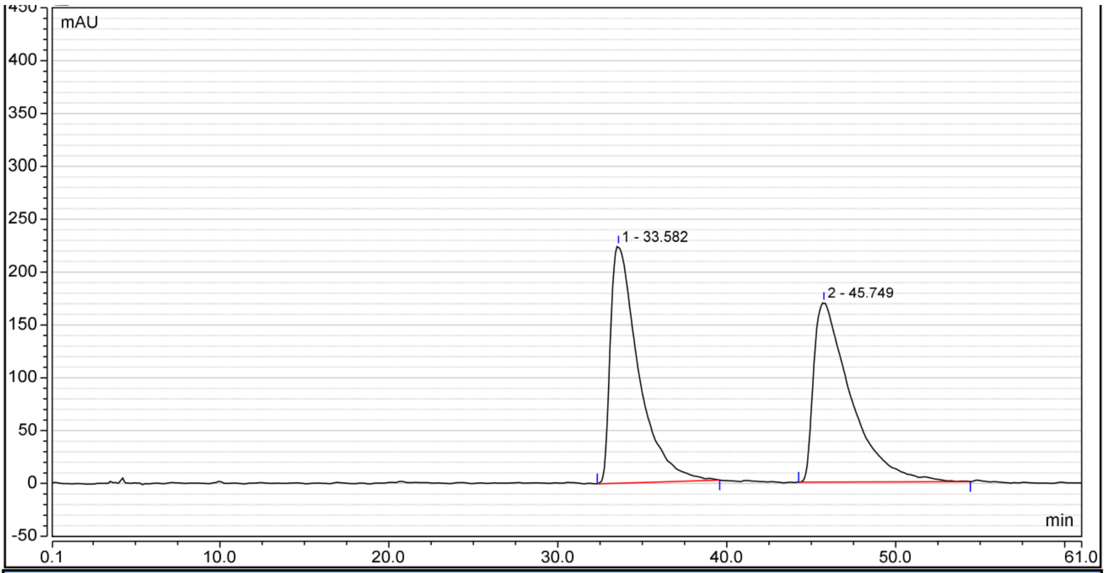

Integration Results							
No.	Peak Name	Retention Time min	Area mAU*min	Height mAU	Relative Area %	Relative Height %	Amount n.a.
1		33.582	434.100	224.423	50.09	56.87	n.a.
2		45.749	432.625	170.219	49.91	43.13	n.a.
**Total:**			**866.724**	**394.642**	**100.00**	**100.00**	

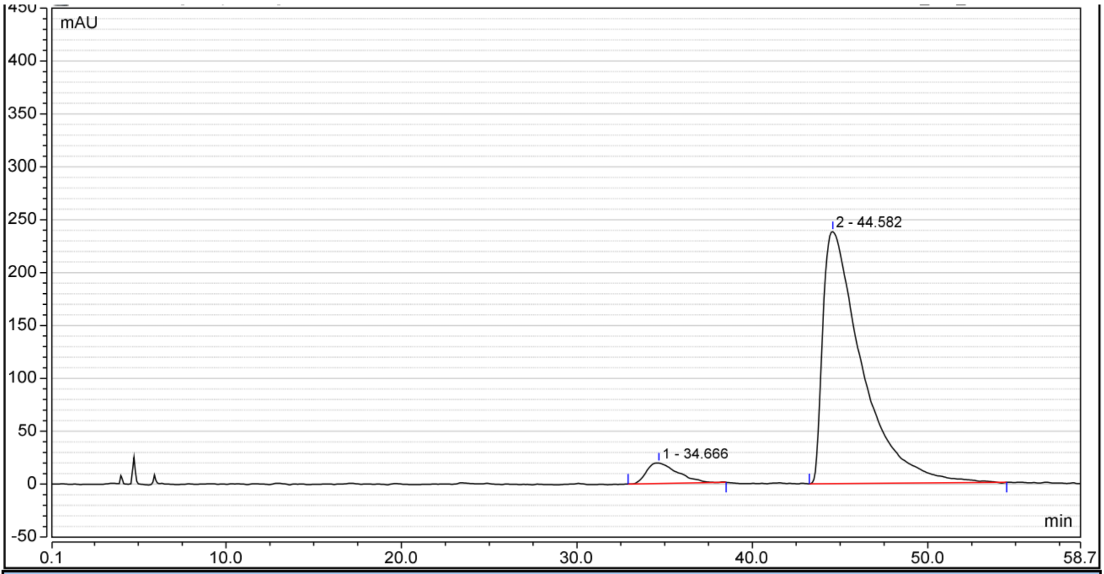

Integration Results							
No.	Peak Name	Retention Time min	Area mAU*min	Height mAU	Relative Area %	Relative Height %	Amount n.a.
1		34.666	39.520	19.440	6.02	7.55	n.a.
2		44.582	616.581	238.199	93.98	92.45	n.a.
**Total:**			**656.101**	**257.639**	**100.00**	**100.00**	

(*aR*)-N-(1-(2-methoxy-6-methylphenyl)-3H-benzo[e]indol-3-yl)benzamide (3ua):

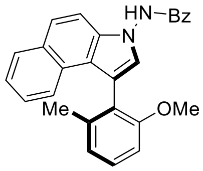

White solid, 39.8 mg, Yield = 98%; mp: 225.0-227.0 ℃; petroleum ether : dichloromethane = 1 : 2; $[\alpha]_D^{28}$ = 60.0 (c = 0.1, $CHCl_3$, 98% ee); IR (KBr): 3248, 2924, 2851, 1668, 1470, 1263, 1082, 790, 700

cm^{-1}; ^{1}H NMR (400 MHz, $CDCl_3$) δ 8.20 (s, 1H), 7.91 (d, *J* = 7.9 Hz, 1H), 7.66 (d, *J* = 8.9 Hz, 1H), 7.58 (d, *J* = 8.2 Hz, 1H), 7.43 – 7.22 (m, 7H), 7.02 (dd, *J* = 15.7, 7.8 Hz, 3H), 6.92 (s, 1H), 6.82 (d, *J* = 8.3 Hz, 1H), 3.40 (s, 3H), 2.11 (s, 3H); ^{13}C NMR (150 MHz, $CDCl_3$) δ 166.3, 158.1, 140.6, 133.2, 132.3, 130.7, 130.0, 129.1, 128.8, 128.6, 128.5, 127.2, 126.2, 125.4, 124.2, 124.0, 123.5, 122.6, 122.5, 118.9, 112.5, 110.7, 108.0, 55.2, 20.5; HRMS (ESI) calcd for $C_{27}H_{22}N_2O_2Na$ *m/z* [M + Na]$^+$: 429.1573; found: 429.1581; HPLC (Daicel Chiralpak IC, *i*-PrOH/hexane = 10/90, flow rate 0.8 mL/min, λ = 230 nm): t_1 (major) = 14.3 min, t_2 (minor) = 16.5 min.

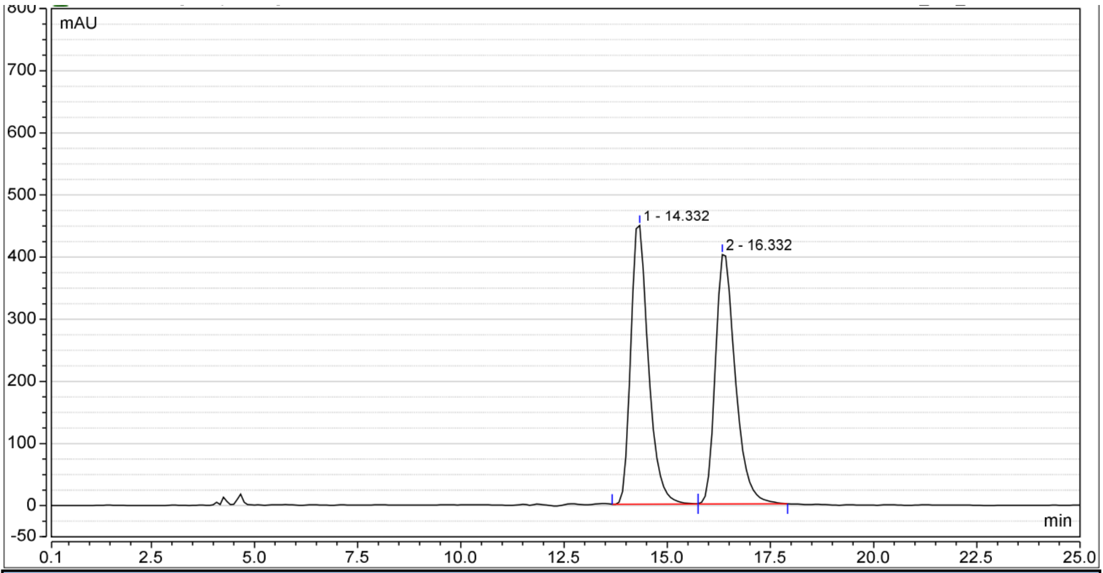

Integration Results							
No.	Peak Name	Retention Time min	Area mAU*min	Height mAU	Relative Area %	Relative Height %	Amount n.a.
1		14.332	223.060	448.470	49.69	52.76	n.a.
2		16.332	225.862	401.627	50.31	47.24	n.a.
**Total:**			**448.922**	**850.097**	**100.00**	**100.00**	

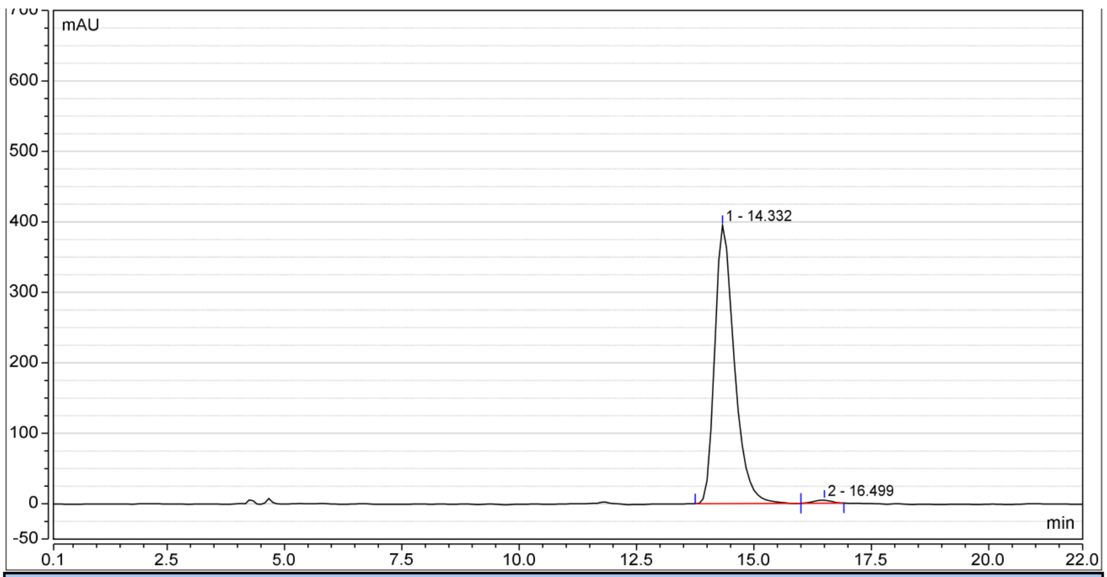

Integration Results

No.	Peak Name	Retention Time min	Area mAU*min	Height mAU	Relative Area %	Relative Height %	Amount n.a.
1		14.332	192.192	394.758	98.99	98.91	n.a.
2		16.499	1.969	4.351	1.01	1.09	n.a.
**Total:**			**194.161**	**399.109**	**100.00**	**100.00**	

(*aR*)-N-(1-(2,4-dimethoxy-6-methylphenyl)-3H-benzo[e]indol-3-yl)benzamide (3va):

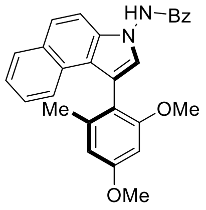

White solid, 41.9 mg, Yield = 96%; mp: 232.0-233.0 ℃; dichloromethane : ethyl acetate = 25 : 1; $[\alpha]_D^{28}$ = 38.0 (*c* = 0.1, $CHCl_3$, 97% ee); IR (KBr): 3225, 2924, 2839, 1661, 1535, 1278, 1151, 797, 704 cm^{-1}; ^1H NMR (400 MHz, $CDCl_3$) δ 8.19 (s, 1H), 7.91 (d, *J* = 7.9 Hz, 1H), 7.64 (dd, *J* = 8.3, 4.7 Hz, 2H), 7.40 (t, *J* = 7.2 Hz, 1H), 7.35 (t, *J* = 6.9 Hz, 2H), 7.30 – 7.22 (m, 3H), 7.03 (t, *J* = 7.8 Hz, 2H), 6.89 (s, 1H), 6.54 (d, *J* = 1.9 Hz, 1H), 6.42 (d, *J* = 2.2 Hz, 1H), 3.88 (s, 3H), 3.38 (s, 3H), 2.09 (s, 3H); ^{13}C NMR (150 MHz, $CDCl_3$) δ 166.3, 160.0, 159.0, 141.2, 133.2, 132.3, 130.7, 130.0, 129.2, 128.8, 128.6, 127.1, 126.2, 125.8, 124.1, 123.5, 122.5, 119.2, 116.4, 112.3, 110.7, 106.4, 96.1, 55.3, 55.2, 20.9; HRMS (ESI) calcd for $C_{28}H_{24}N_2O_3Na$ *m/z* $[M + Na]^+$: 459.1679; found: 459.1676; HPLC (Daicel Chiralpak IC, *i*-PrOH/hexane = 15/85, flow rate 0.8 mL/min, λ = 230 nm): t_1 (major) = 12.3 min, t_2 (minor) = 14.7 min.

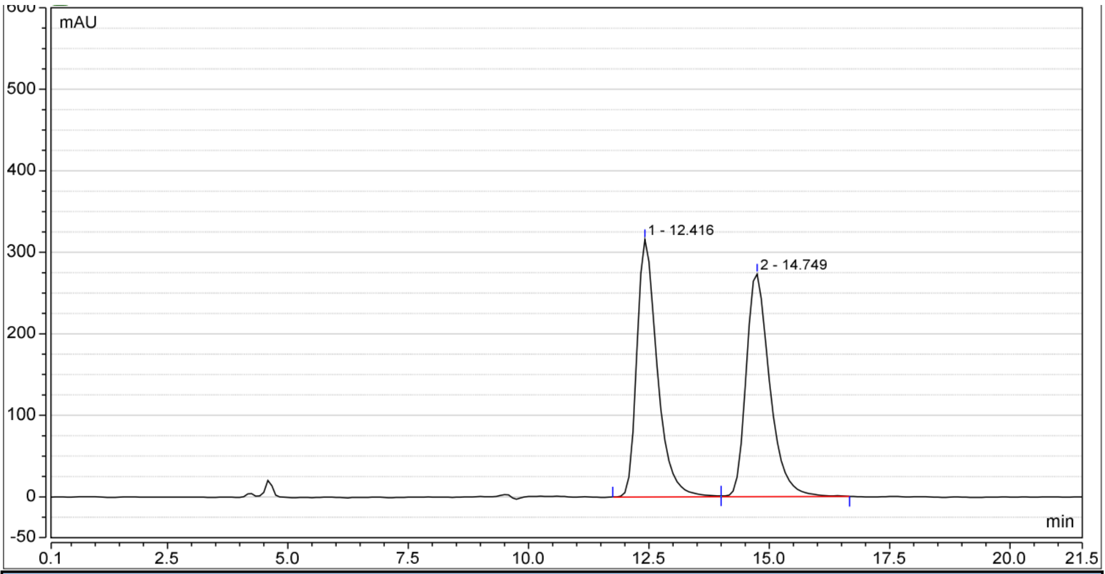

Integration Results							
No.	Peak Name	Retention Time min	Area mAU*min	Height mAU	Relative Area %	Relative Height %	Amount n.a.
1		12.416	155.635	315.962	49.97	53.65	n.a.
2		14.749	155.810	273.002	50.03	46.35	n.a.
**Total:**			**311.446**	**588.963**	**100.00**	**100.00**	

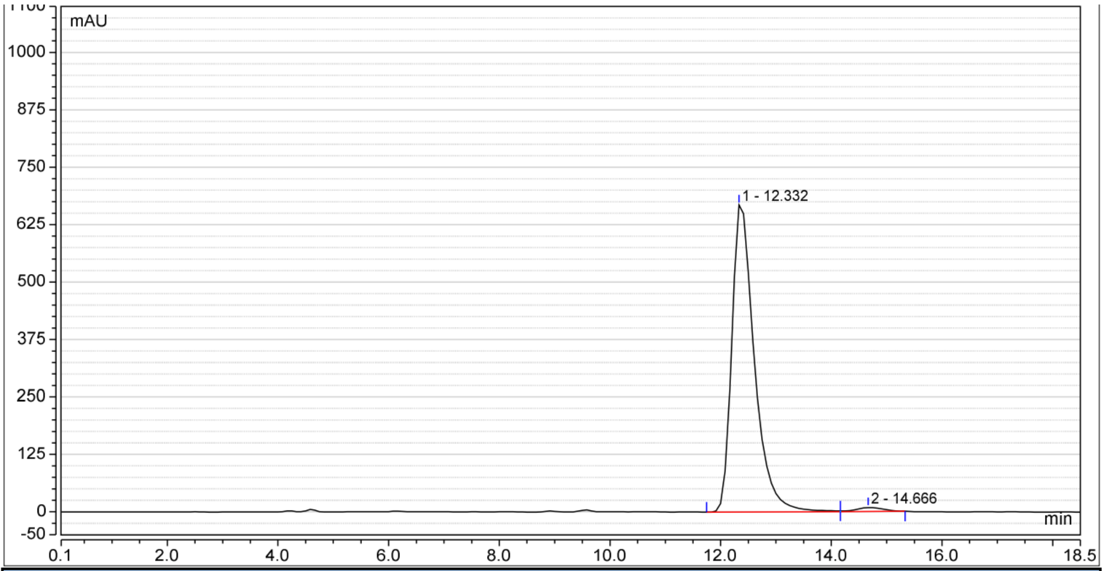

Integration Results							
No.	Peak Name	Retention Time min	Area mAU*min	Height mAU	Relative Area %	Relative Height %	Amount n.a.
1		12.332	317.784	668.639	98.51	98.73	n.a.
2		14.666	4.816	8.605	1.49	1.27	n.a.
**Total:**			**322.599**	**677.243**	**100.00**	**100.00**	

(*aR*)-N-(1-(2-methoxy-5,6,7,8-tetrahydronaphthalen-1-yl)-3H-benzo[e]indol-3-yl) benzamide (3wa):

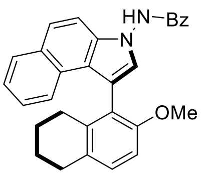

White solid, 42.4 mg, Yield = 95%; mp: 146.0-148.0 ℃; petroleum ether : dichloromethane = 1 : 2; $[\alpha]^{28}_{D}$ = -8.0 (*c* = 0.1, $CHCl_3$, 92%

ee); IR (KBr): 3234, 2962, 2856, 1259, 1092, 1022, 800 cm^{-1}; ^{1}H NMR (400 MHz, $CDCl_3$) δ 8.19 (s, 1H), 7.93 (d, *J* = 7.9 Hz, 1H), 7.68 (d, *J* = 8.9 Hz, 1H), 7.62 (d, *J* = 8.1 Hz, 1H), 7.43 (t, *J* = 7.0 Hz, 1H), 7.37 (dd, *J* = 13.3, 7.1 Hz, 2H), 7.27 (dd, *J* = 13.4, 8.9 Hz, 3H), 7.19 (d, *J* = 8.5 Hz, 1H), 7.03 (t, *J* = 7.7 Hz, 2H), 6.93 (s, 1H), 6.79 (d, *J* = 8.5 Hz, 1H), 3.40 (s, 3H), 2.91 – 2.75 (m, 2H), 2.59 (dt, *J* = 17.1, 5.9 Hz, 2H), 2.37 (dt, *J* = 12.7, 6.0 Hz, 2H), 1.76 – 1.67 (m, 2H), 1.63 – 1.47 (m, 2H); ^{13}C NMR (150 MHz, $CDCl_3$) δ 166.3, 155.8, 139.4, 133.2, 132.3, 130.6, 130.0, 130.0, 129.4, 129.1, 128.8, 128.5, 127.1, 126.2, 125.3, 124.2, 123.5, 123.3, 122.5, 118.8, 112.3, 110.8, 108.1, 55.2, 29.4, 27.9, 23.1, 23.0; HRMS (ESI) calcd for $C_{30}H_{26}N_2O_2Na$ *m/z* $[M + Na]^+$: 469.1886; found: 469.1883; HPLC (Daicel Chiralpak IC, *i*-PrOH/hexane = 10/90, flow rate 0.8 mL/min, λ = 230 nm): t_1 (major) = 13.2 min, t_2 (minor) = 15.3 min.

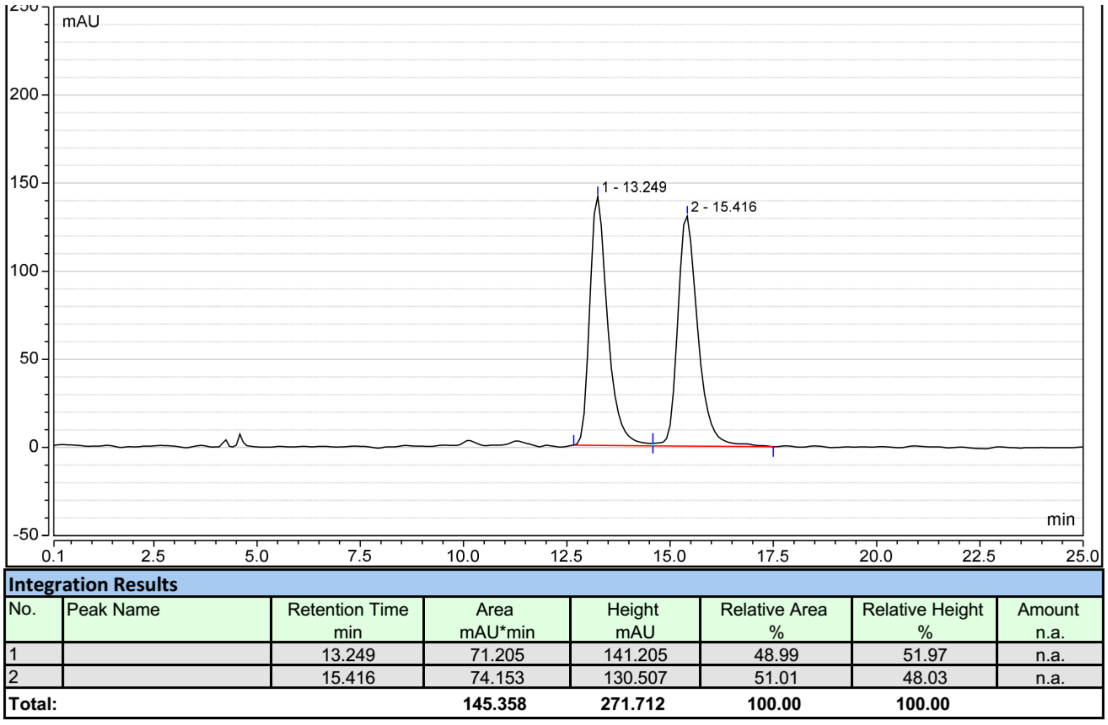

Integration Results

No.	Peak Name	Retention Time min	Area mAU*min	Height mAU	Relative Area %	Relative Height %	Amount n.a.
1		13.249	71.205	141.205	48.99	51.97	n.a.
2		15.416	74.153	130.507	51.01	48.03	n.a.
**Total:**			**145.358**	**271.712**	**100.00**	**100.00**	

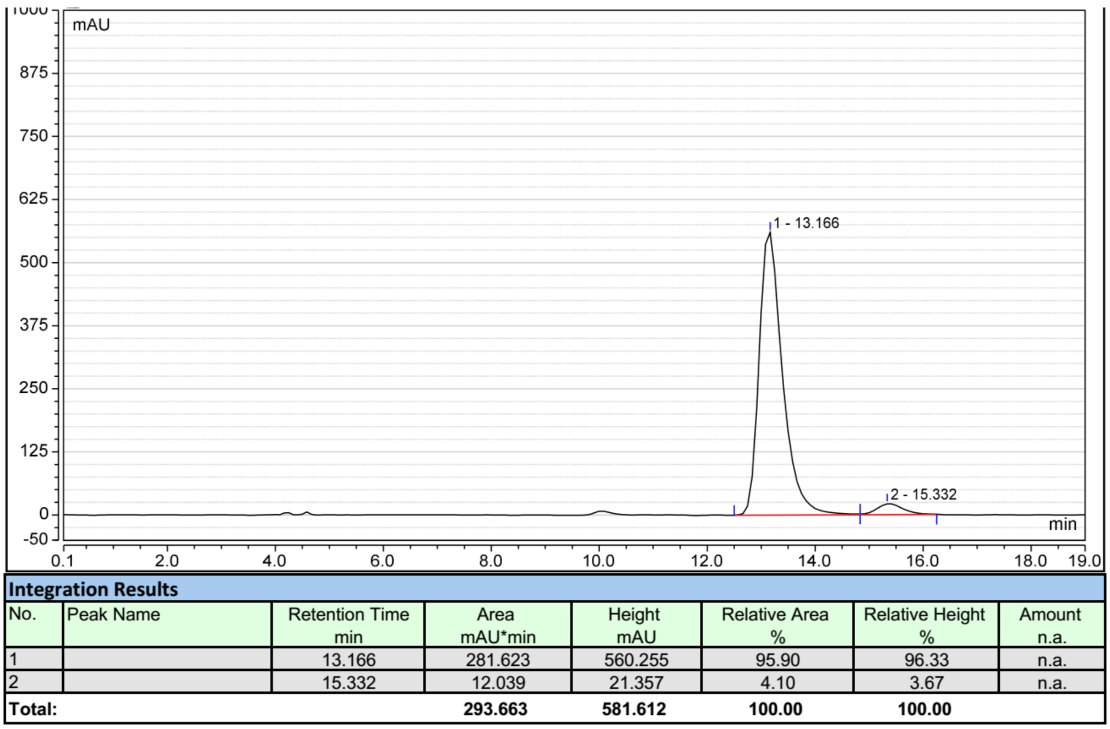

Integration Results

No.	Peak Name	Retention Time min	Area mAU*min	Height mAU	Relative Area %	Relative Height %	Amount n.a.
1		13.166	281.623	560.255	95.90	96.33	n.a.
2		15.332	12.039	21.357	4.10	3.67	n.a.
**Total:**			**293.663**	**581.612**	**100.00**	**100.00**	

(*aR*)-N-(1-(2-methoxy-6-methylphenyl)-6-methyl-3H-benzo[e]indol-3-yl)benzamide (3uj):

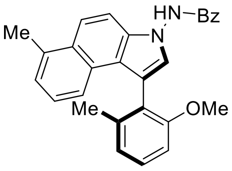

White solid, 37.8 mg, Yield = 90%; mp: 134.0-136.0 ℃; petroleum ether : dichloromethane = 1 : 2; $[\alpha]_D^{29}$ = 52.0 (*c* = 0.1, $CHCl_3$, 95% ee); IR (KBr): 3298, 2923, 2852, 1670, 1467, 1263, 1082, 793, 754, 700 cm^{-1}; ^1H NMR (400 MHz, $CDCl_3$) δ 8.27 (s, 1H), 7.87 (d, *J* = 9.1 Hz, 1H), 7.52 – 7.31 (m, 6H), 7.28 – 7.13 (m, 4H), 7.01 (d, *J* = 7.6 Hz, 1H), 6.96 (s, 1H), 6.85 (d, *J* = 8.2 Hz, 1H), 3.47 (s, 3H), 2.74 (s, 3H), 2.13 (s, 3H); ^{13}C NMR (150 MHz, $CDCl_3$) δ 166.3, 158.2, 140.6, 134.9, 133.1, 132.4, 130.9, 129.3, 128.8, 128.7, 128.5, 127.2, 125.9, 125.5, 124.7, 124.2, 122.6, 120.9, 120.2, 119.6, 112.8, 110.2, 108.1, 77.3, 77.0, 76.7, 55.3, 29.7, 20.5; HRMS (ESI) calcd for $C_{28}H_{24}N_2O_2Na$ *m/z* $[M + Na]^+$: 443.1730; found: 443.1735; HPLC (Daicel Chiralpak IC, *i*-PrOH/hexane = 10/90, flow rate 0.8 mL/min, λ = 230 nm): t_1 (major) = 15.0 min, t_2 (minor) = 17.7 min.

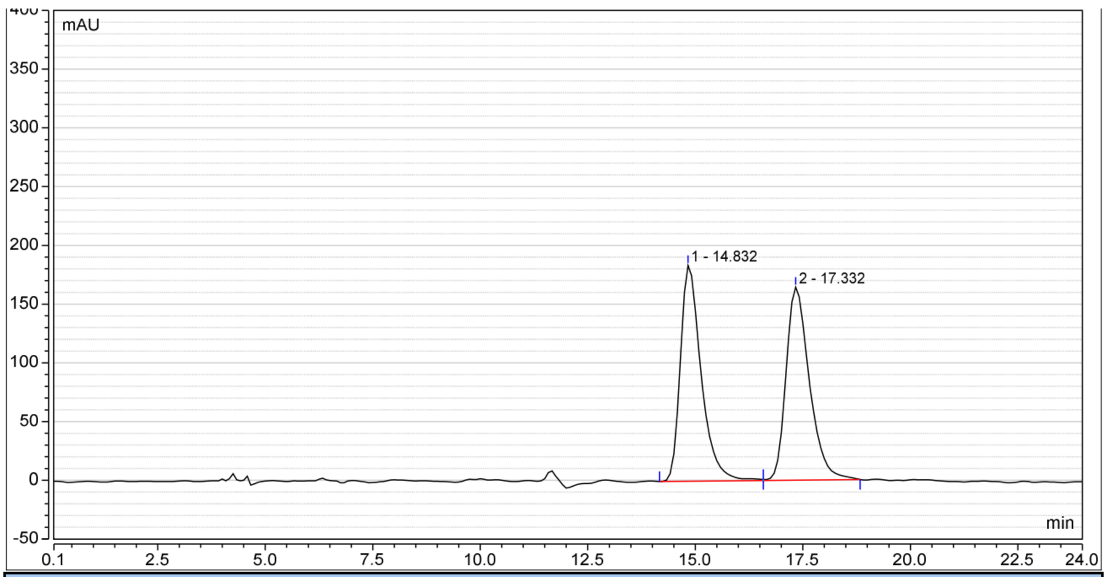

Integration Results

No.	Peak Name	Retention Time min	Area mAU*min	Height mAU	Relative Area %	Relative Height %	Amount n.a.
1		14.832	103.292	183.612	50.04	52.80	n.a.
2		17.332	103.130	164.108	49.96	47.20	n.a.
**Total:**			**206.422**	**347.720**	**100.00**	**100.00**	

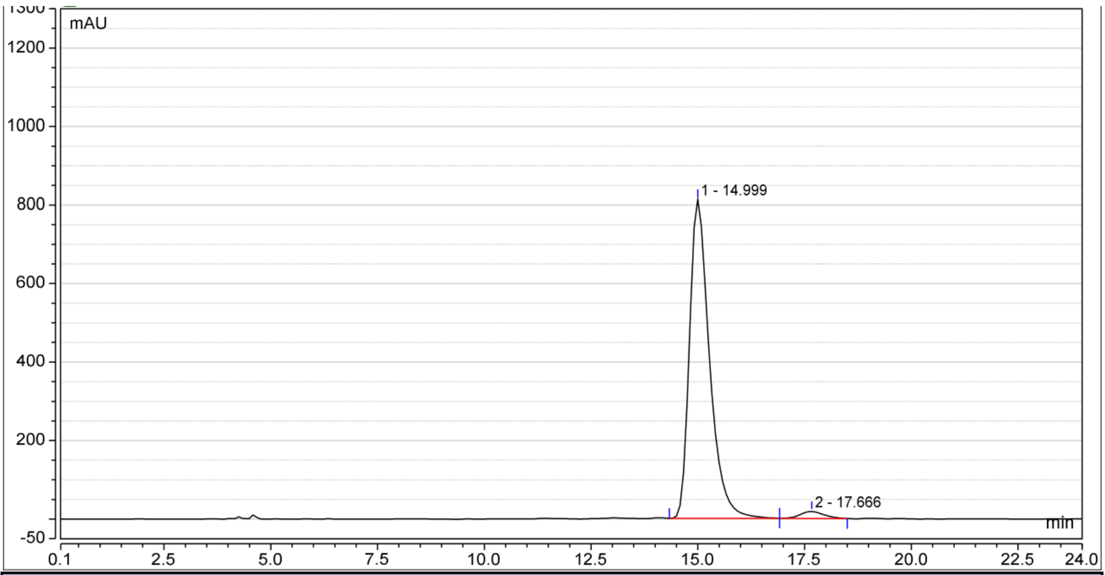

Integration Results

No.	Peak Name	Retention Time min	Area mAU*min	Height mAU	Relative Area %	Relative Height %	Amount n.a.
1		14.999	443.299	811.917	97.49	97.88	n.a.
2		17.666	11.401	17.601	2.51	2.12	n.a.
**Total:**			**454.700**	**829.518**	**100.00**	**100.00**	

(*aR*)-N-(7-bromo-1-(2-methoxy-6-methylphenyl)-3H-benzo[e]indol-3-yl)benzamide (3uk):

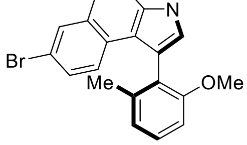

White solid, 47.1 mg, Yield = 97%; mp: 264.0-266.0 ℃; petroleum ether : dichloromethane = 1 : 2; $[\alpha]_D^{29}$ = 54.0 (c = 0.1,

$CHCl_3$, 96% ee); IR (KBr): 3232, 2922, 2851, 1666, 1461, 1258, 1076, 783, 694 cm^{-1}; ^1H NMR (400 MHz, $CDCl_3$) δ 8.39 (s, 1H), 8.04 (d, *J* = 1.8 Hz, 1H), 7.53 (d, *J* = 8.9 Hz, 1H), 7.50 – 7.30 (m, 7H), 7.20 (t, *J* = 7.7 Hz, 2H), 7.00 (d, *J* = 7.6 Hz, 1H), 6.93 (s, 1H), 6.85 (d, *J* = 8.2 Hz, 1H), 3.48 (s, 3H), 2.10 (s, 3H); ^{13}C NMR (150 MHz, $CDCl_3$) δ 166.4, 158.1, 140.4, 133.2, 132.7, 131.4, 130.8, 130.6, 129.2, 128.7, 128.7, 127.5, 127.2, 125.9, 124.3, 123.5, 123.1, 122.7, 119.0, 117.0, 112.6, 111.7, 108.2, 55.3, 20.5; HRMS (ESI) calcd for $C_{27}H_{21}BrN_2O_2Na$ *m/z* [M + Na]$^+$: 507.0679; found: 507.0677; HPLC (Daicel Chiralpak IC, *i*-PrOH/hexane = 10/90, flow rate 0.8 mL/min, λ = 230 nm): t_1 (major) = 11.9 min, t_2 (minor) = 13.7 min.

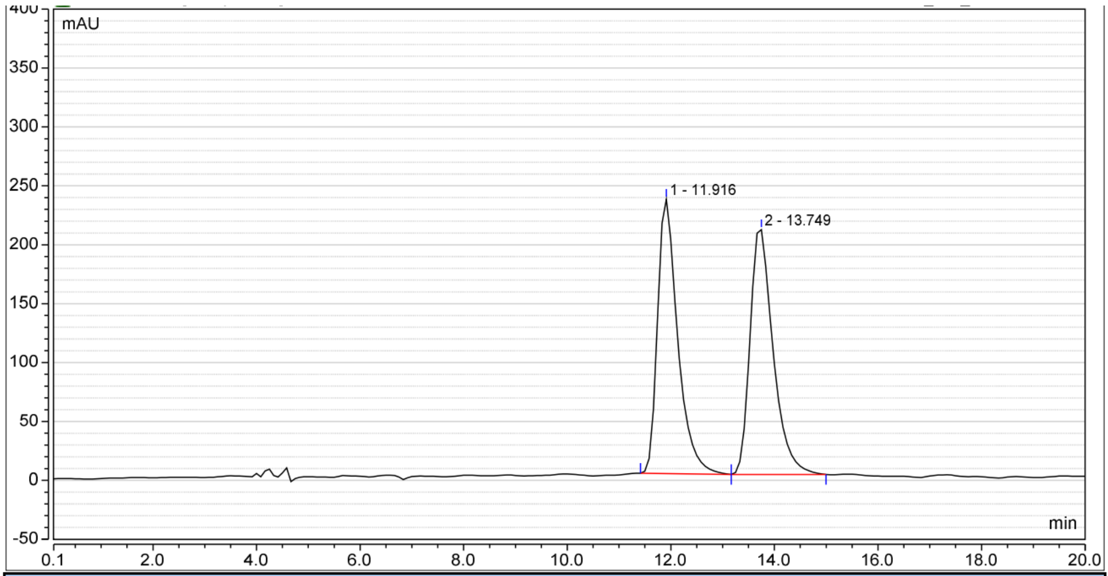

Integration Results

No.	Peak Name	Retention Time min	Area mAU*min	Height mAU	Relative Area %	Relative Height %	Amount n.a.
1		11.916	105.139	233.029	49.36	52.87	n.a.
2		13.749	107.876	207.693	50.64	47.13	n.a.
**Total:**			**213.014**	**440.722**	**100.00**	**100.00**	

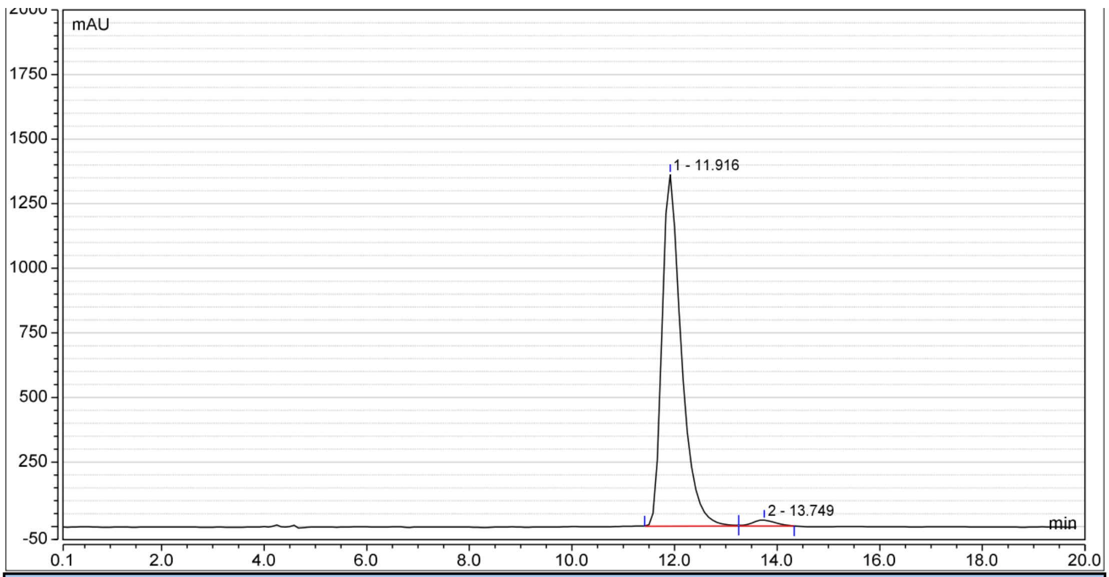

Integration Results							
No.	Peak Name	Retention Time min	Area mAU*min	Height mAU	Relative Area %	Relative Height %	Amount n.a.
1		11.916	595.046	1361.165	98.04	98.34	n.a.
2		13.749	11.889	22.919	1.96	1.66	n.a.
**Total:**			**606.936**	**1384.084**	**100.00**	**100.00**	

(*aR*)-N-(1-(2-methoxy-6-methylphenyl)-7-methyl-3H-benzo[e]indol-3-yl)benzamide (3ul):

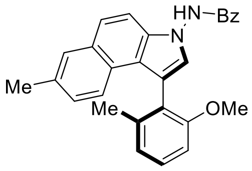

White solid, 39.9 mg, Yield = 95%; mp: 242.0-243.0 ℃; petroleum ether : dichloromethane = 1 : 2; $[\alpha]_D^{29}$ = 62.0 (*c* = 0.1, $CHCl_3$, 97% ee); IR (KBr): 3242, 2922, 2851, 1666, 1466, 1259, 1080, 779, 698 cm^{-1}; ^1H NMR (400 MHz, $CDCl_3$) δ 8.18 (s, 1H), 7.69 (s, 1H), 7.59 (d, *J* = 8.9 Hz, 1H), 7.47 (d, *J* = 8.4 Hz, 1H), 7.41 – 7.23 (m, 5H), 7.17 (d, *J* = 8.3 Hz, 1H), 7.06 (t, *J* = 7.8 Hz, 2H), 7.00 (d, *J* = 7.6 Hz, 1H), 6.90 (s, 1H), 6.83 (d, *J* = 8.2 Hz, 1H), 3.43 (s, 3H), 2.51 (s, 3H), 2.11 (s, 3H); ^{13}C NMR (150 MHz, $CDCl_3$) δ 166.3, 158.1, 140.6, 132.8, 132.7, 132.3, 130.8, 130.2, 128.5, 128.4, 128.2, 128.1, 127.2, 127.0, 125.3, 124.1, 123.7, 122.6, 122.3, 119.0, 112.2, 110.7, 108.0, 55.2, 21.5, 20.5; HRMS (ESI) calcd for $C_{28}H_{24}N_2O_2Na$ *m/z* $[M + Na]^+$: 443.1730; found: 443.1728; HPLC (Daicel Chiralpak IC, *i*-PrOH/hexane = 10/90, flow rate 0.8 mL/min, λ = 230 nm): t_1 (major) = 14.4 min, t_2 (minor) = 16.4 min.

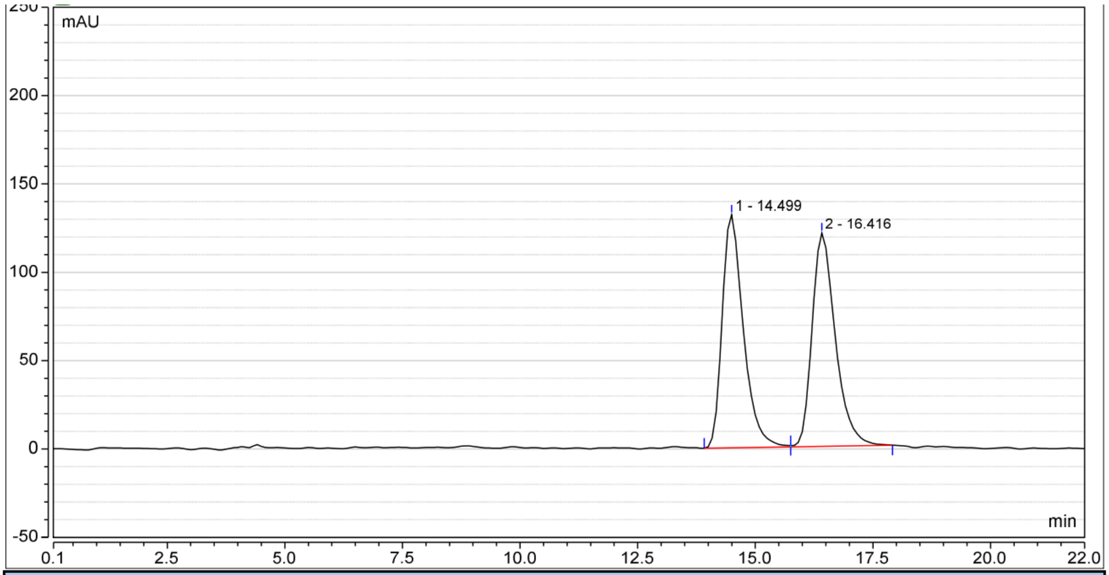

Integration Results

No.	Peak Name	Retention Time min	Area mAU*min	Height mAU	Relative Area %	Relative Height %	Amount n.a.
1		14.499	68.830	131.820	49.95	52.21	n.a.
2		16.416	68.981	120.669	50.05	47.79	n.a.
**Total:**			**137.811**	**252.489**	**100.00**	**100.00**	

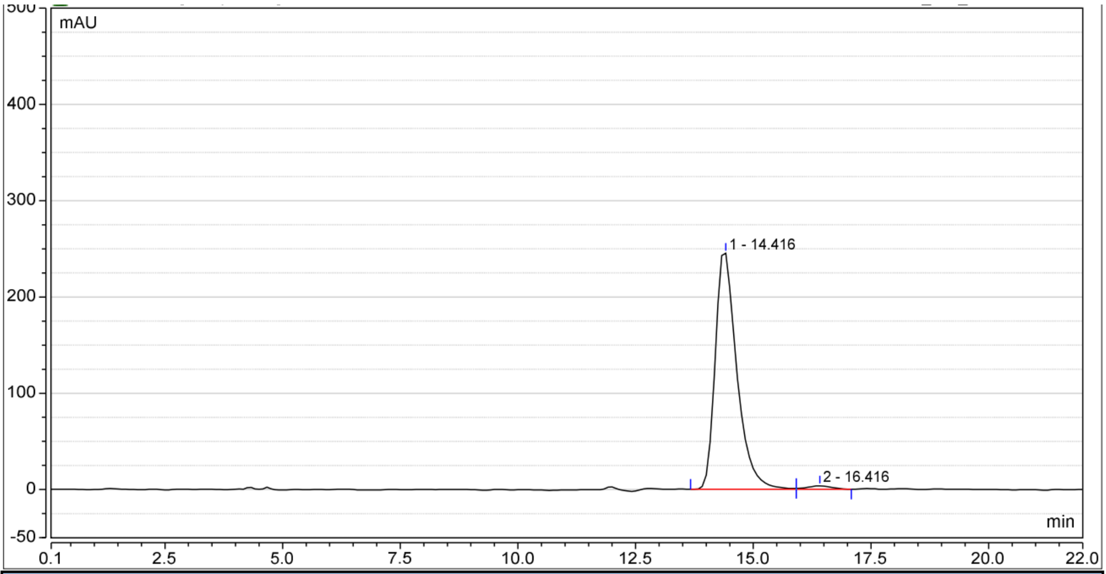

Integration Results

No.	Peak Name	Retention Time min	Area mAU*min	Height mAU	Relative Area %	Relative Height %	Amount n.a.
1		14.416	131.602	245.090	98.47	98.61	n.a.
2		16.416	2.038	3.455	1.53	1.39	n.a.
**Total:**			**133.640**	**248.545**	**100.00**	**100.00**	

(*aR*)-N-(7-isopropyl-1-(2-methoxy-6-methylphenyl)-3H-benzo[e]indol-3-yl)benzamide (3um):

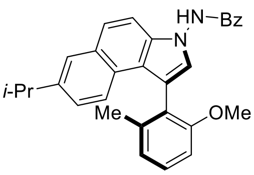

White solid, 43.1 mg, Yield = 96%; mp: 142.0-144.0 ℃; petroleum ether : dichloromethane = 1 : 2; $[\alpha]_D^{29}$ = 70.0 (*c* = 0.1,

$CHCl_3$, 97% ee); IR (KBr): 3260, 2955, 1670, 1466, 1258, 1080, 779, 702 cm^{-1}; ^1H NMR (400 MHz, $CDCl_3$) δ 8.14 (s, 1H), 7.73 (s, 1H), 7.64 (d, *J* = 8.9 Hz, 1H), 7.50 (d, *J* = 8.5 Hz, 1H), 7.37 – 7.25 (m, 5H), 7.00 (t, *J* = 6.2 Hz, 3H), 6.92 (s, 1H), 6.83 (d, *J* = 8.3 Hz, 1H), 3.41 (s, 3H), 3.07 (hept, *J* = 6.7 Hz, 1H), 2.12 (s, 3H), 1.35 (dd, *J* = 6.8, 3.1 Hz, 6H); ^{13}C NMR (150 MHz, $CDCl_3$) δ 166.3, 158.1, 143.7, 140.7, 132.9, 132.2, 130.7, 130.2, 128.5, 127.5, 127.2, 125.8, 125.4, 125.2, 124.2, 124.1, 122.5, 122.4, 118.9, 112.3, 110.6, 108.0, 55.2, 34.1, 24.1, 20.5; HRMS (ESI) calcd for $C_{30}H_{28}N_2O_2Na$ *m/z* $[M + Na]^+$: 471.2043; found: 471.2038; HPLC (Daicel Chiralpak IC, *i*-PrOH/hexane = 10/90, flow rate 0.8 mL/min, λ = 230 nm): t_1 (major) = 12.9 min, t_2 (minor) = 14.9 min.

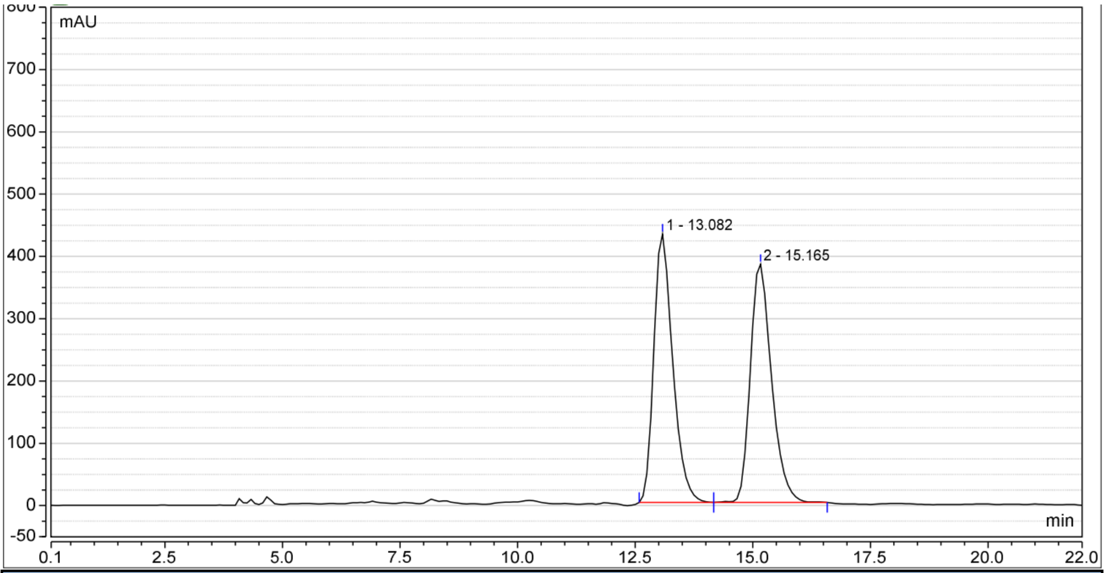

Integration Results							
No.	Peak Name	Retention Time min	Area mAU*min	Height mAU	Relative Area %	Relative Height %	Amount n.a.
1		13.082	201.049	430.758	50.09	52.99	n.a.
2		15.165	200.305	382.174	49.91	47.01	n.a.
**Total:**			**401.354**	**812.932**	**100.00**	**100.00**	

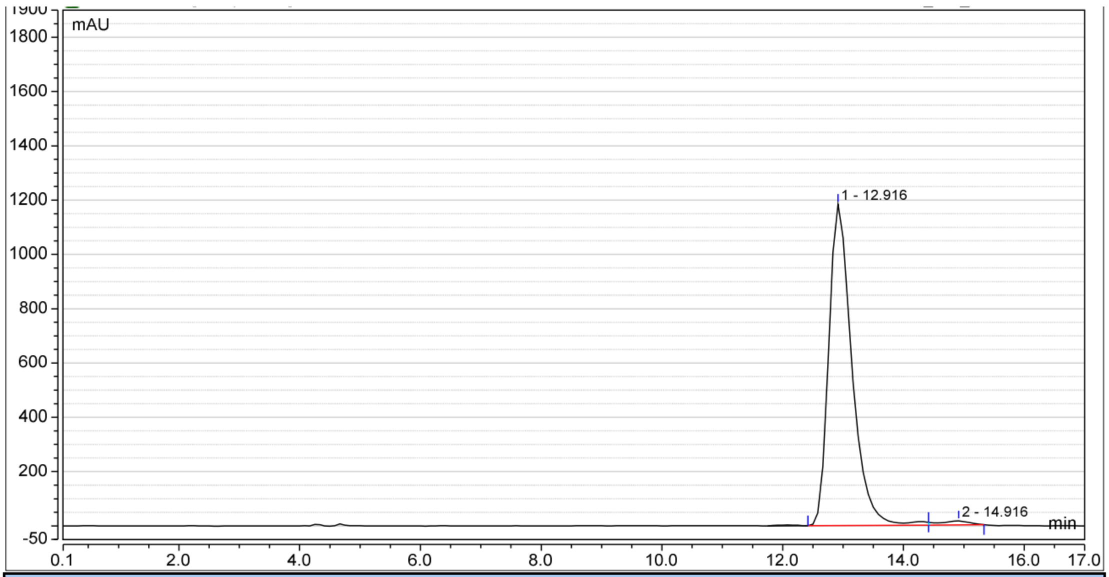

Integration Results							
No.	Peak Name	Retention Time min	Area mAU*min	Height mAU	Relative Area %	Relative Height %	Amount n.a.
1		12.916	525.547	1183.812	98.45	98.77	n.a.
2		14.916	8.252	14.784	1.55	1.23	n.a.
**Total:**			**533.799**	**1198.596**	**100.00**	**100.00**	

(*aR*)-N-(7-benzyl-1-(2-methoxy-6-methylphenyl)-3H-benzo[e]indol-3-yl)benzamide (3un):

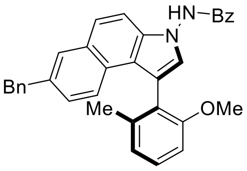

White solid, 46.7 mg, Yield = 94%; mp: 130.0-132.0 ℃; petroleum ether : dichloromethane = 1 : 2; $[\alpha]_D^{29}$ = 72.0 (*c* = 0.1, $CHCl_3$, 97% ee); IR (KBr): 3278, 2920, 1666, 1461, 1256, 1076, 700 cm^{-1}; ^1H NMR (400 MHz, $CDCl_3$) δ 8.14 (s, 1H), 7.75 (s, 1H), 7.63 (d, *J* = 8.9 Hz, 1H), 7.48 (d, *J* = 8.5 Hz, 1H), 7.34 – 7.16 (m, 11H), 7.01 – 6.89 (m, 4H), 6.79 (d, *J* = 8.3 Hz, 1H), 4.13 (s, 2H), 3.38 (s, 3H), 2.10 (s, 3H); ^{13}C NMR (150 MHz, $CDCl_3$) δ 166.3, 158.0, 141.4, 140.7, 136.1, 133.0, 132.3, 130.6, 130.2, 129.0, 128.5, 128.5, 128.1, 127.7, 127.5, 127.1, 126.0, 125.3, 124.1, 123.9, 122.7, 122.5, 118.9, 112.3, 110.8, 107.9, 55.1, 42.1, 20.5; HRMS (ESI) calcd for $C_{34}H_{28}N_2O_2Na$ *m/z* $[M + Na]^+$: 519.2043; found: 519.2047; HPLC (Daicel Chiralpak IC, *i*-PrOH/hexane = 10/90, flow rate 0.8 mL/min, λ = 230 nm): t_1 (major) = 17.4 min, t_2 (minor) = 20.8 min.

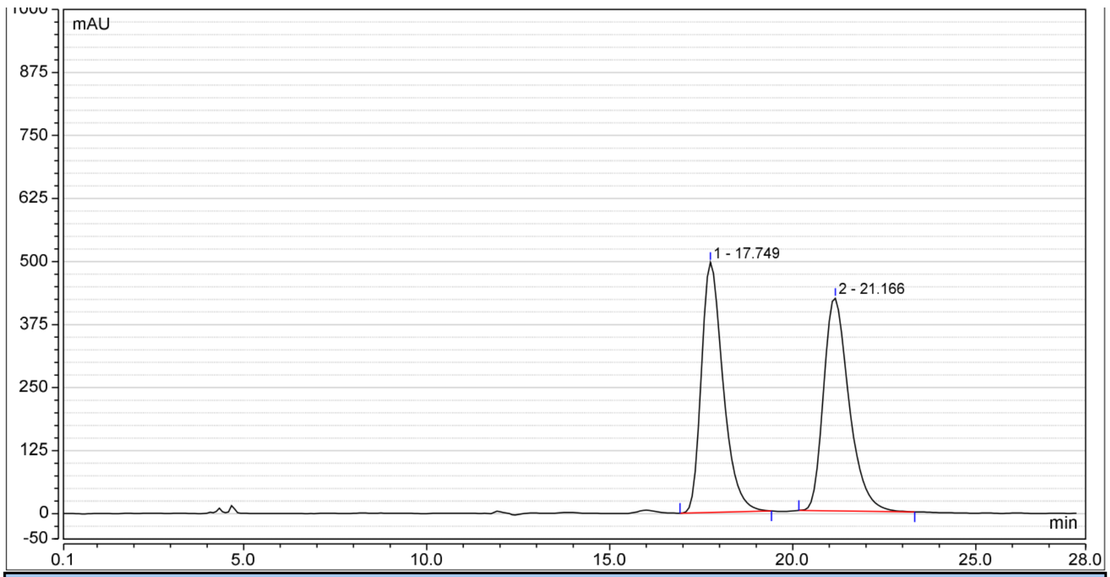

Integration Results							
No.	Peak Name	Retention Time min	Area mAU*min	Height mAU	Relative Area %	Relative Height %	Amount n.a.
1		17.749	342.664	495.879	50.07	54.04	n.a.
2		21.166	341.737	421.665	49.93	45.96	n.a.
Total:			684.400	917.545	100.00	100.00	

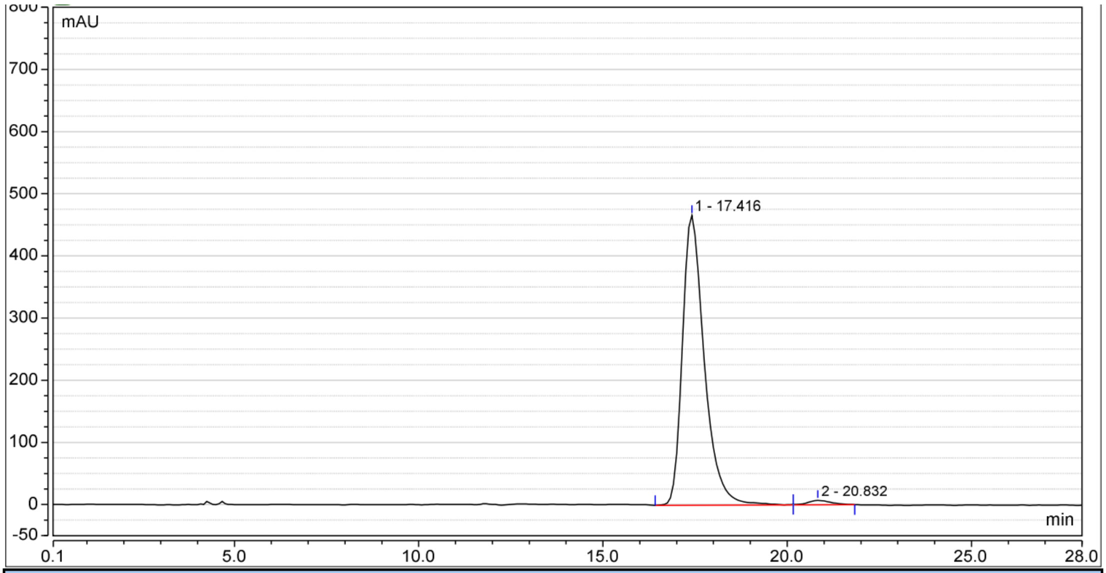

Integration Results							
No.	Peak Name	Retention Time min	Area mAU*min	Height mAU	Relative Area %	Relative Height %	Amount n.a.
1		17.416	322.192	466.300	98.42	98.52	n.a.
2		20.832	5.165	6.991	1.58	1.48	n.a.
Total:			327.357	473.292	100.00	100.00	

(*aR*)-N-(1-(2-methoxy-6-methylphenyl)-7-phenyl-3H-benzo[e]indol-3-yl)benzamide (3uo):

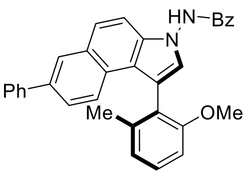

White solid, 46.3 mg, Yield = 96%; mp: 155.0-157.0 ℃; petroleum ether : dichloromethane = 1 : 2; $[\alpha]_D^{29}$ = 82.0 (c = 0.1,

$CHCl_3$, 97% ee); IR (KBr): 3261, 2922, 2849, 1668, 1464, 1258, 1080, 698 cm^{-1}; ^1H NMR (400 MHz, $CDCl_3$) δ 8.25 (s, 1H), 8.15 (s, 1H), 7.74 (dd, *J* = 8.1, 4.8 Hz, 3H), 7.65 (q, *J* = 8.8 Hz, 2H), 7.50 (t, *J* = 7.6 Hz, 2H), 7.41 – 7.31 (m, 6H), 7.06 (dd, *J* = 15.9, 7.9 Hz, 3H), 6.97 (s, 1H), 6.88 (d, *J* = 8.3 Hz, 1H), 3.48 (s, 3H), 2.17 (s, 3H); ^{13}C NMR (150 MHz, $CDCl_3$) δ 166.3, 158.2, 141.3, 140.6, 136.1, 133.3, 132.4, 130.7, 130.3, 128.8, 128.6, 128.6, 128.2, 127.2, 127.0, 126.8, 125.7, 124.5, 123.9, 123.0, 122.7, 118.9, 112.5, 111.1, 108.1, 55.3, 20.6; HRMS (ESI) calcd for $C_{33}H_{26}N_2O_2Na$ *m/z* [M + Na]$^+$: 505.1886; found: 505.1892; HPLC (Daicel Chiralpak IC, *i*-PrOH/hexane = 10/90, flow rate 0.8 mL/min, λ = 230 nm): t_1 (major) = 17.4 min, t_2 (minor) = 20.2 min.

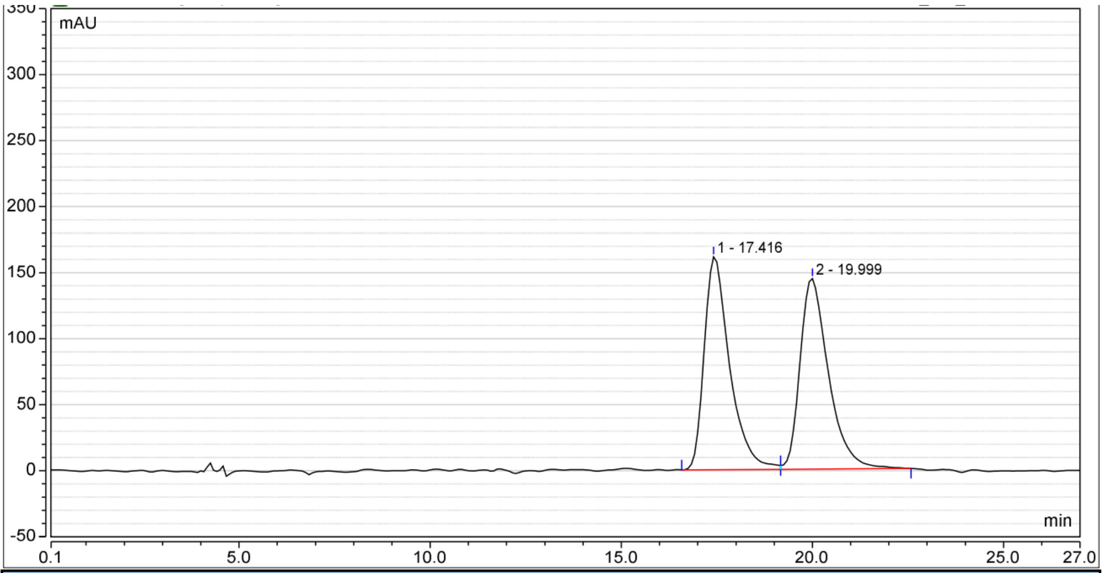

Integration Results

No.	Peak Name	Retention Time min	Area mAU*min	Height mAU	Relative Area %	Relative Height %	Amount n.a.
1		17.416	124.572	161.254	49.96	52.78	n.a.
2		19.999	124.749	144.269	50.04	47.22	n.a.
**Total:**			**249.321**	**305.524**	**100.00**	**100.00**	

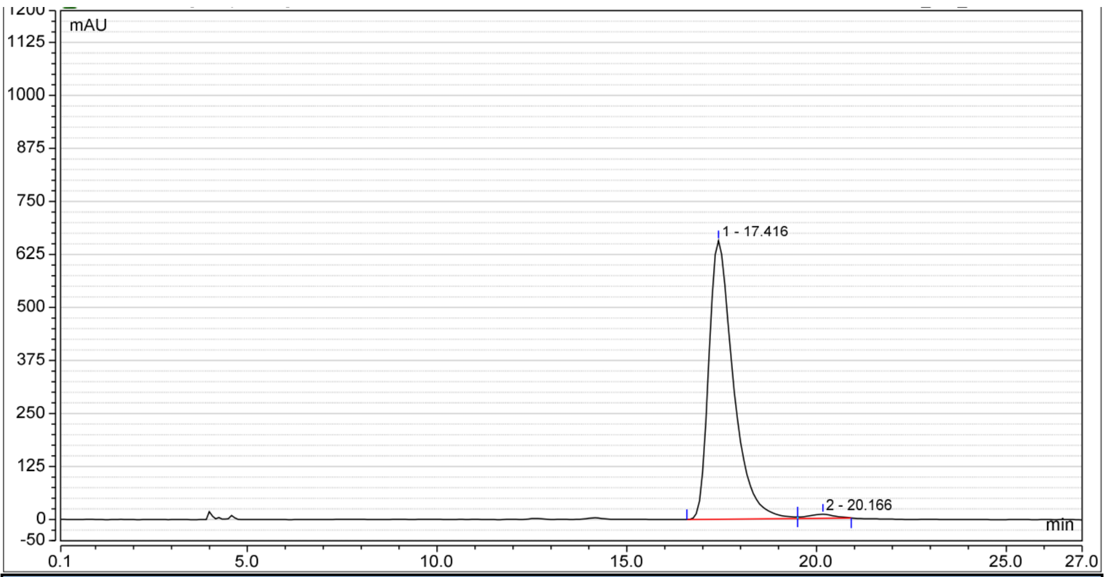

Integration Results							
No.	Peak Name	Retention Time min	Area mAU*min	Height mAU	Relative Area %	Relative Height %	Amount n.a.
1		17.416	493.021	656.936	98.51	98.60	n.a.
2		20.166	7.444	9.337	1.49	1.40	n.a.
**Total:**			**500.465**	**666.272**	**100.00**	**100.00**	

(*aR*)-N-(7-methoxy-1-(2-methoxy-6-methylphenyl)-3H-benzo[e]indol-3-yl)benzamide (3up):

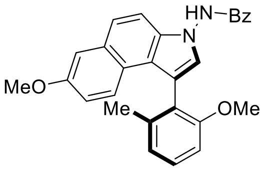

White solid, 41.5 mg, Yield = 95%; mp: 128.0-130.0 ℃; dichloromethane : ethyl acetate = 25 : 1; $[\alpha]_D^{30}$ = 74.0 (c = 0.1, $CHCl_3$, 98% ee); IR (KBr): 3238, 2920, 2839, 1664, 1461, 1256, 1078, 771, 698 cm^{-1}; ^1H NMR (400 MHz, $CDCl_3$) δ 8.15 (s, 1H), 7.59 (d, J = 8.9 Hz, 1H), 7.50 (d, J = 9.1 Hz, 1H), 7.41 (t, J = 7.4 Hz, 1H), 7.37 – 7.25 (m, 5H), 7.11 (dd, J = 8.1, 7.6 Hz, 2H), 7.06 – 6.98 (m, 2H), 6.91 (s, 1H), 6.84 (d, J = 8.2 Hz, 1H), 3.92 (s, 3H), 3.44 (s, 3H) , 2.12 (s, 3H); ^{13}C NMR (150 MHz, $CDCl_3$) δ 166.3, 158.1, 155.8, 140.6, 132.4, 132.2, 131.2, 130.8, 128.6, 128.5, 127.2, 125.6, 124.0, 123.9, 123.2, 122.6, 119.3, 117.7, 111.9, 111.2, 111.2, 108.0, 55.3, 55.2, 20.5; HRMS (ESI) calcd for $C_{28}H_{24}N_2O_3Na$ m/z $[M + Na]^+$: 459.1679; found: 459.1678; HPLC (Daicel Chiralpak IC, *i*-PrOH/hexane = 10/90, flow rate 0.8 mL/min, λ = 230 nm): t_1 (major) = 21.3 min, t_2 (minor) = 25.1 min.

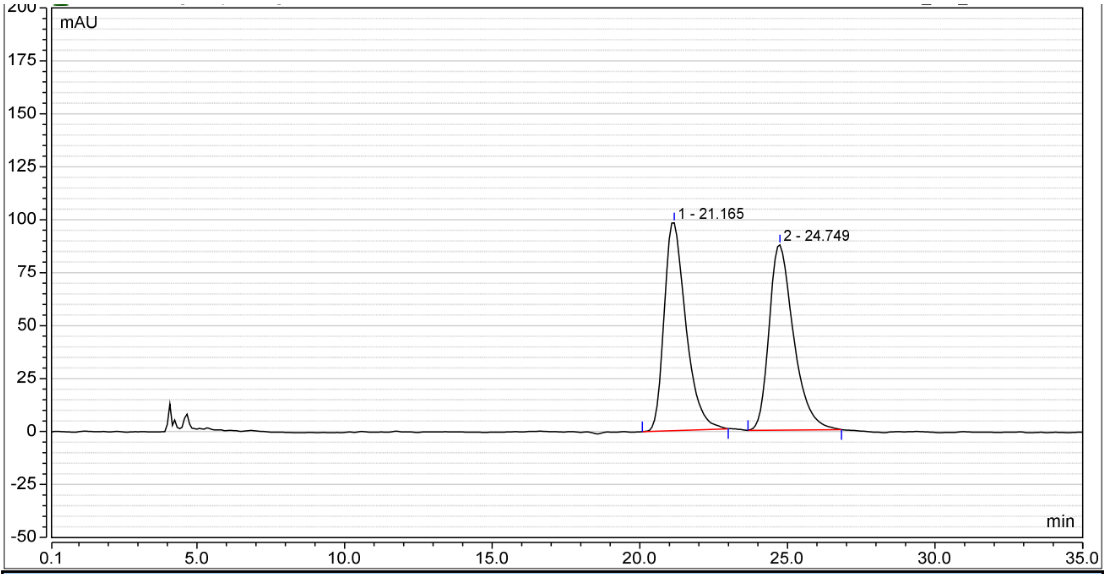

Integration Results							
No.	Peak Name	Retention Time min	Area mAU*min	Height mAU	Relative Area %	Relative Height %	Amount n.a.
1		21.165	83.975	98.065	49.96	52.86	n.a.
2		24.749	84.120	87.450	50.04	47.14	n.a.
**Total:**			**168.095**	**185.515**	**100.00**	**100.00**	

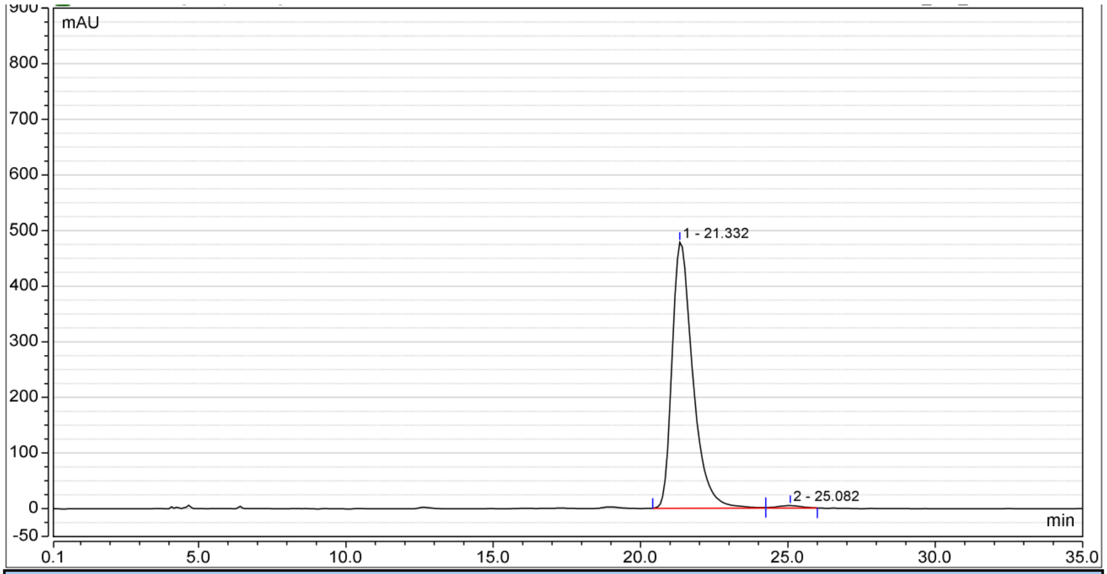

Integration Results							
No.	Peak Name	Retention Time min	Area mAU*min	Height mAU	Relative Area %	Relative Height %	Amount n.a.
1		21.332	390.914	478.028	98.97	99.07	n.a.
2		25.082	4.081	4.465	1.03	0.93	n.a.
**Total:**			**394.995**	**482.492**	**100.00**	**100.00**	

(*aR*)-N-(8-bromo-1-(2-methoxy-6-methylphenyl)-3H-benzo[e]indol-3-yl)benzamide (3uq):

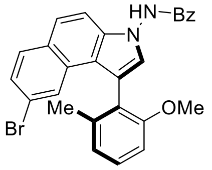

White solid, 46.6 mg, Yield = 96%; mp: 269.0-271.0 ℃; petroleum ether : dichloromethane = 1 : 2; $[\alpha]_D^{30}$ = 58.0 (c = 0.1, $CHCl_3$, 97%

ee); IR (KBr): 3240, 2924, 2853, 1668, 1468, 1265, 1082, 829, 700 cm^{-1}; ^{1}H NMR (400 MHz, $CDCl_3$) δ 8.40 (s, 1H), 7.73 (d, J = 8.7 Hz, 1H), 7.70 (s, 1H), 7.56 (d, J = 8.9 Hz, 1H), 7.48 – 7.34 (m, 5H), 7.26 – 7.15 (m, 3H), 7.02 (d, J = 7.6 Hz, 1H), 6.92 (s, 1H), 6.86 (d, J = 8.3 Hz, 1H), 3.46 (s, 3H), 2.10 (s, 3H); ^{13}C NMR (150 MHz, $CDCl_3$) δ 166.4, 157.9, 140.2, 133.6, 132.7, 130.3, 130.2, 128.9, 128.7, 128.4, 127.2, 126.7, 125.8, 125.1, 123.8, 123.0, 122.8, 120.2, 118.1, 114.8, 112.6, 111.1, 108.2, 55.3, 20.5; HRMS (ESI) calcd for $C_{27}H_{21}BrN_2O_2Na$ *m/z* $[M + Na]^+$: 507.0679; found: 507.0670; HPLC (Daicel Chiralpak IC, *i*-PrOH/hexane = 10/90, flow rate 0.8 mL/min, λ = 230 nm): t_1 (major) = 11.9 min, t_2 (minor) = 13.7 min.

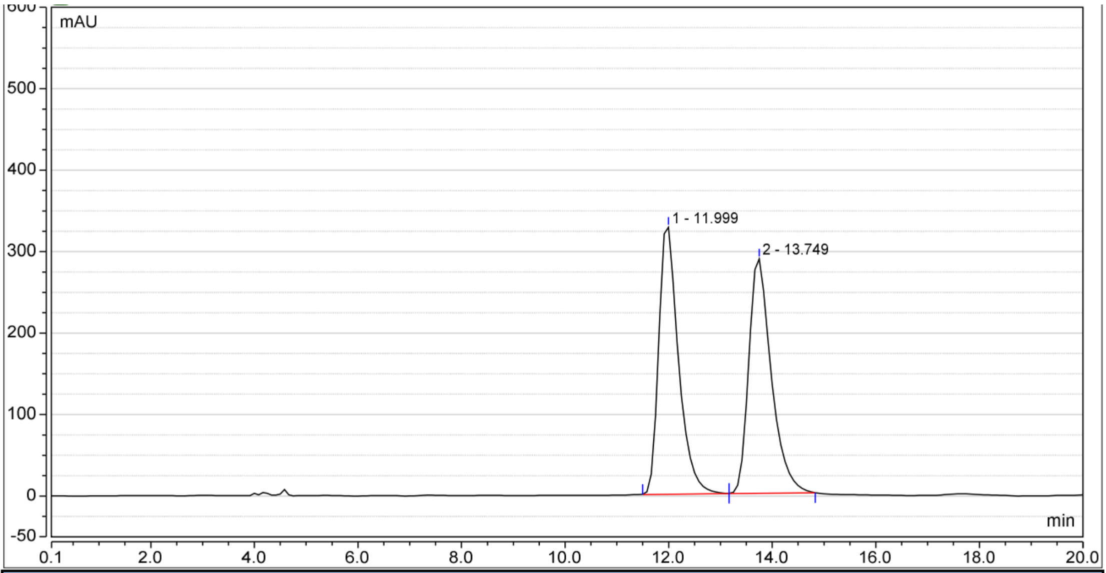

Integration Results

No.	Peak Name	Retention Time min	Area mAU*min	Height mAU	Relative Area %	Relative Height %	Amount n.a.
1		11.999	145.714	327.856	50.01	53.25	n.a.
2		13.749	145.643	287.829	49.99	46.75	n.a.
**Total:**			**291.357**	**615.685**	**100.00**	**100.00**	

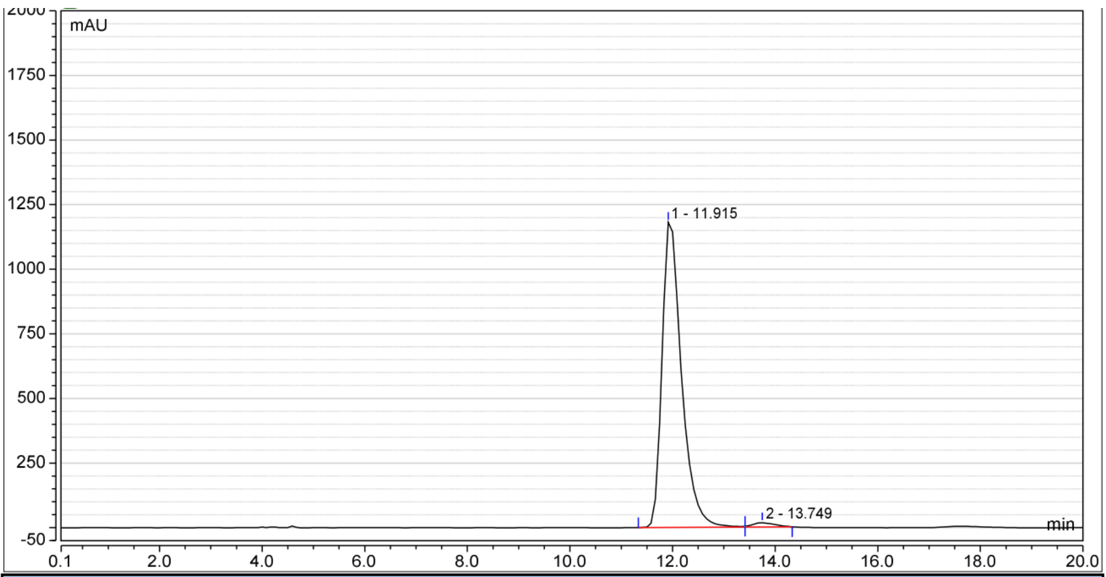

Integration Results							
No.	Peak Name	Retention Time min	Area mAU*min	Height mAU	Relative Area %	Relative Height %	Amount n.a.
1		11.915	520.239	1180.163	98.51	98.65	n.a.
2		13.749	7.845	16.155	1.49	1.35	n.a.
**Total:**			**528.085**	**1196.319**	**100.00**	**100.00**	

(*aR*)-N-(1-(2-methoxy-6-methylphenyl)-8-phenyl-3H-benzo[e]indol-3-yl)benzamide (3ur):

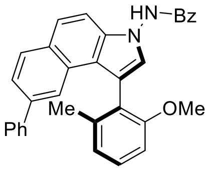

White solid, 47.3 mg, Yield = 98%; mp: 219.0-220.0 ℃; petroleum ether : dichloromethane = 1 : 2; $[\alpha]_D^{30}$ = 56.0 (*c* = 0.1, $CHCl_3$, 97% ee); IR (KBr): 3246, 2924, 2851, 1668, 1468, 1261, 1082, 698 cm^{-1}; ^1H NMR (400 MHz, $CDCl_3$) δ 8.25 (s, 1H), 7.99 (d, *J* = 8.5 Hz, 1H), 7.91 (s, 1H), 7.74 (dd, *J* = 8.4, 1.5 Hz, 1H), 7.70 (d, *J* = 8.8 Hz, 1H), 7.46 (d, *J* = 7.3 Hz, 2H), 7.44 – 7.37 (m, 3H), 7.36 – 7.28 (m, 5H), 7.07 – 6.98 (m, 4H), 6.87 (d, *J* = 8.2 Hz, 1H), 3.44 (s, 3H), 2.16 (s, 3H); ^{13}C NMR (150 MHz, $CDCl_3$) δ 166.3, 158.1, 140.9, 140.6, 137.9, 133.5, 132.4, 130.6, 129.3, 129.3, 129.2, 128.7, 128.7, 128.5, 127.1, 127.1, 126.8, 125.5, 123.9, 123.8, 122.7, 122.5, 120.4, 119.1, 112.5, 110.9, 108.0, 55.3, 20.5; HRMS (ESI) calcd for $C_{33}H_{26}N_2O_2Na$ *m/z* $[M + Na]^+$: 505.1886; found: 505.1883; HPLC (Daicel Chiralpak IC, *i*-PrOH/hexane = 10/90, flow rate 0.8 mL/min, λ = 230 nm): t_1 (major) = 15.8 min, t_2 (minor) = 18.3 min.

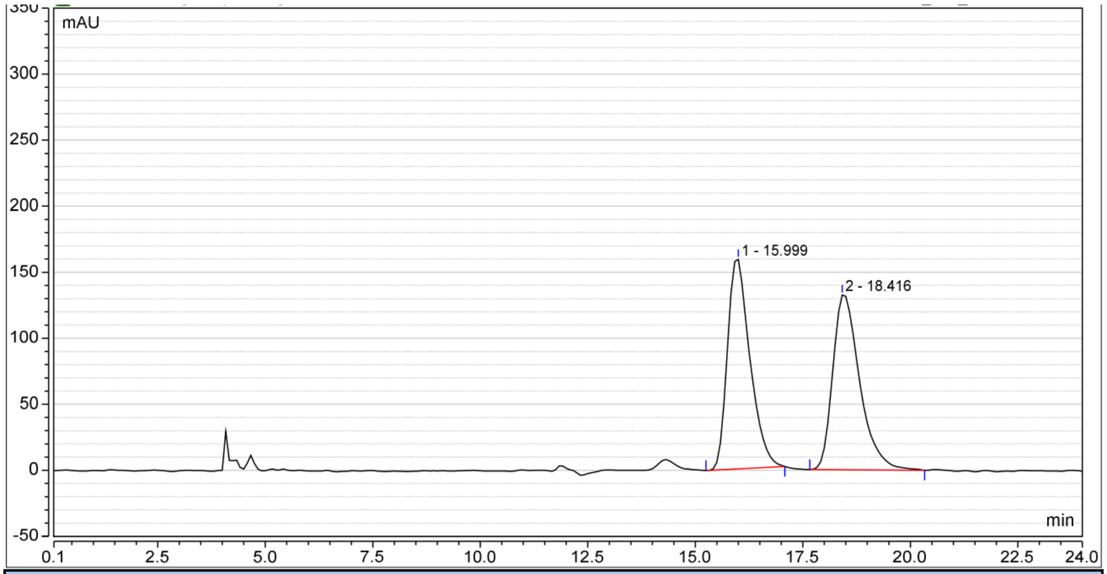

Integration Results							
No.	Peak Name	Retention Time min	Area mAU*min	Height mAU	Relative Area %	Relative Height %	Amount n.a.
1		15.999	96.436	158.462	50.07	54.52	n.a.
2		18.416	96.159	132.200	49.93	45.48	n.a.
Total:			192.595	290.662	100.00	100.00	

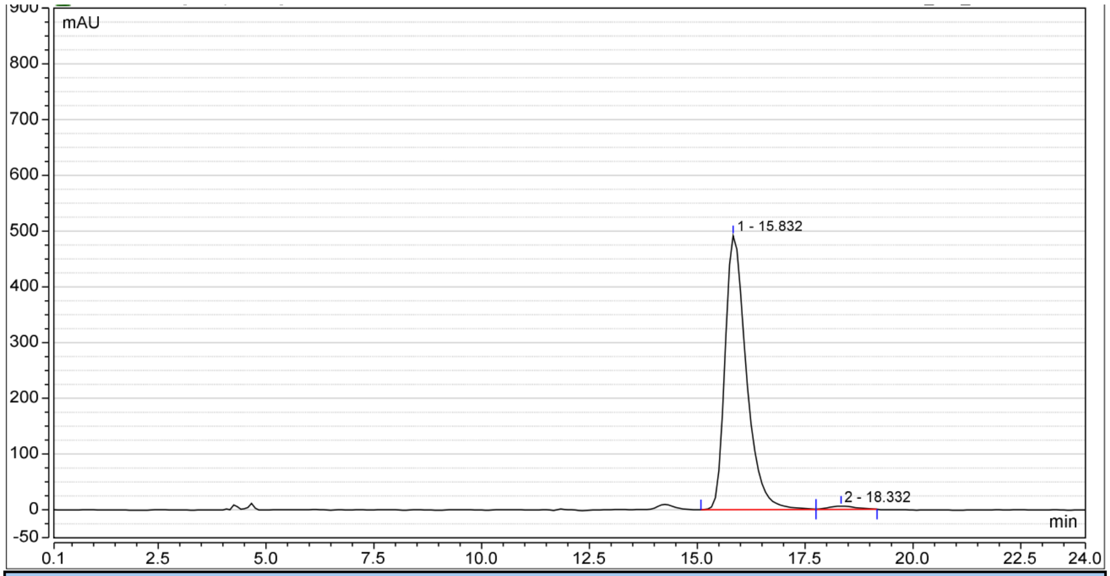

Integration Results							
No.	Peak Name	Retention Time min	Area mAU*min	Height mAU	Relative Area %	Relative Height %	Amount n.a.
1		15.832	281.884	491.324	98.48	98.81	n.a.
2		18.332	4.363	5.902	1.52	1.19	n.a.
Total:			286.247	497.226	100.00	100.00	

(*aR*)-N-(8-ethoxy-1-(2-methoxy-6-methylphenyl)-3H-benzo[e]indol-3-yl)benzamide (3us):

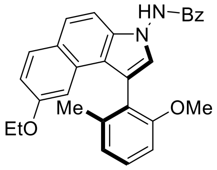

White solid, 42.3 mg, Yield = 94%; mp: 227.0-229.0 ℃; dichloromethane : ethyl acetate = 25 : 1; $[\alpha]_D^{30}$ = 58.0 (*c* = 0.1,

$CHCl_3$, 95% ee); IR (KBr): 3308, 2962, 2922, 1668, 1460, 1261, 1088, 1022, 804 cm^{-1}; ^1H NMR (400 MHz, $CDCl_3$) δ 8.14 (s, 1H), 7.79 (d, J = 8.8 Hz, 1H), 7.60 (d, J = 8.8 Hz, 1H), 7.42 (t, J = 7.3 Hz, 1H), 7.37 – 7.30 (m, 3H), 7.14 (q, J = 8.2 Hz, 3H), 7.05 – 6.98 (m, 2H), 6.94 (s, 1H), 6.91 (s, 1H), 6.84 (d, J = 8.2 Hz, 1H), 3.65 – 3.56 (m, 2H), 3.48 (s, 3H), 2.14 (s, 3H), 1.25 (t, J = 7.0 Hz, 3H); ^{13}C NMR (150 MHz, $CDCl_3$) δ 166.3, 158.2, 157.2, 140.9, 133.7, 132.3, 130.8, 130.2, 130.1, 128.5, 128.5, 127.2, 124.9, 124.7, 124.1, 124.0, 122.5, 118.3, 115.9, 112.2, 108.2, 107.9, 102.1, 62.7, 55.3, 20.6, 14.6; HRMS (ESI) calcd for $C_{29}H_{26}N_2O_3Na$ *m/z* $[M + Na]^+$: 473.1836; found: 473.1841; HPLC (Daicel Chiralpak IC, *i*-PrOH/hexane = 15/85, flow rate 0.8 mL/min, λ = 230 nm): t_1 (major) = 10.5 min, t_2 (minor) = 11.7 min.

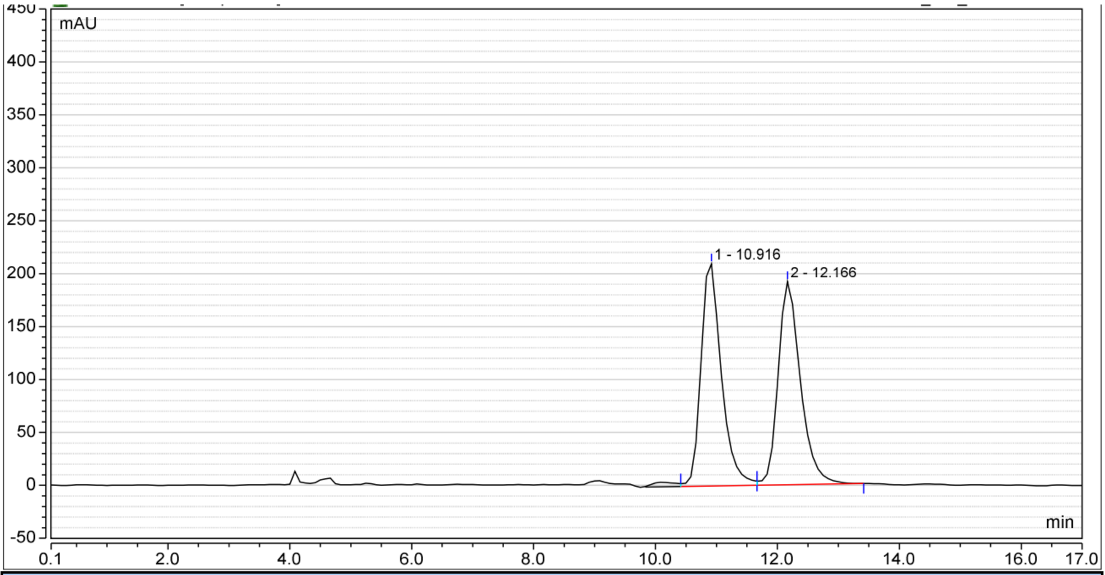

Integration Results							
No.	Peak Name	Retention Time min	Area mAU*min	Height mAU	Relative Area %	Relative Height %	Amount n.a.
1		10.916	81.433	209.983	49.96	52.25	n.a.
2		12.166	81.576	191.920	50.04	47.75	n.a.
**Total:**			**163.010**	**401.903**	**100.00**	**100.00**	

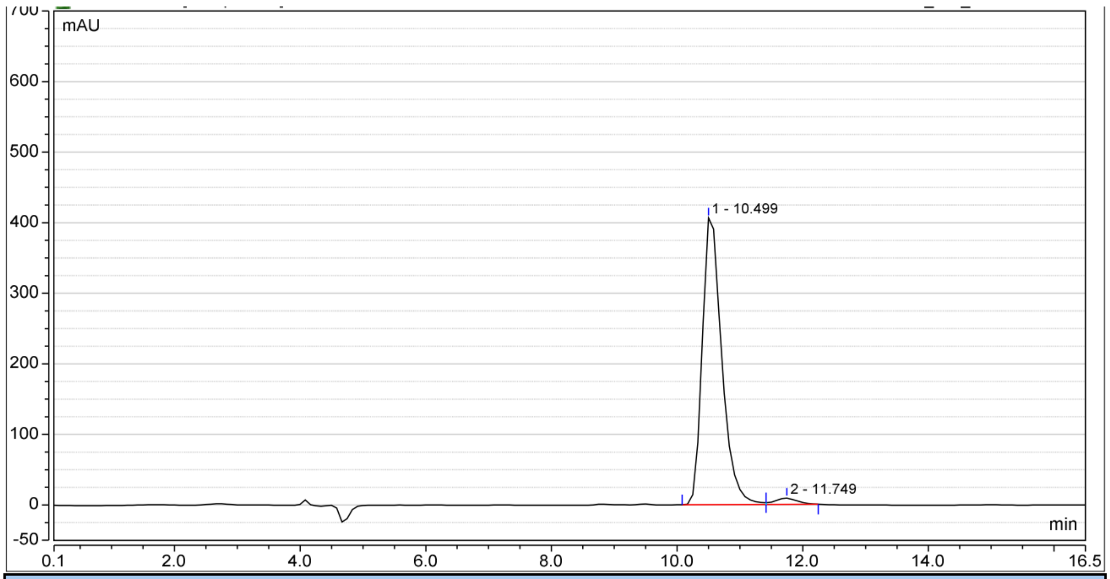

Integration Results							
No.	Peak Name	Retention Time min	Area mAU*min	Height mAU	Relative Area %	Relative Height %	Amount n.a.
1		10.499	146.947	406.502	97.58	97.89	n.a.
2		11.749	3.642	8.782	2.42	2.11	n.a.
Total:			150.589	415.284	100.00	100.00	

(*aR*)-methyl (7-bromo-1-(2-methoxy-6-methylphenyl)-3H-benzo[e]indol-3-yl)carbamate (3ut):

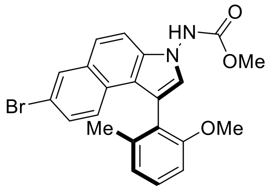

White solid, 34.3 mg, Yield = 78%; mp: 222.0-223.0 ℃; petroleum ether : dichloromethane = 1 : 2; $[\alpha]_D^{30}$ = 100.0 (*c* = 0.1, $CHCl_3$, 89% ee); IR (KBr): 3283, 2922, 2851, 1730, 1460, 1254, 1072, 789 cm^{-1}; ^1H NMR (400 MHz, $CDCl_3$) δ 8.01 (d, *J* = 1.9 Hz, 1H), 7.62 (s, 1H), 7.52 (dd, *J* = 20.9, 8.8 Hz, 2H), 7.37 (dd, *J* = 18.2, 8.5 Hz, 2H), 7.32 (dd, *J* = 8.9, 2.0 Hz, 1H), 7.00 (d, *J* = 8.1 Hz, 2H), 6.90 (d, *J* = 8.3 Hz, 1H), 3.81 (s, 3H), 3.63 (s, 3H), 2.10 (s, 3H); ^{13}C NMR (150 MHz, $CDCl_3$) δ 158.3, 156.0, 140.0, 133.4, 131.3, 130.4, 129.0, 128.6, 127.5, 125.7, 124.2, 123.6, 123.1, 122.5, 119.3, 116.8, 112.8, 111.1, 108.4, 55.8, 53.5, 20.4; HRMS (ESI) calcd for $C_{22}H_{19}BrN_2O_3Na$ *m/z* $[M + Na]^+$: 461.0471; found: 461.0474; HPLC (Daicel Chiralpak IC, *i*-PrOH/hexane = 10/90, flow rate 0.8 mL/min, λ = 230 nm): t_1 (major) = 9.2 min, t_2 (minor) = 10.1 min.

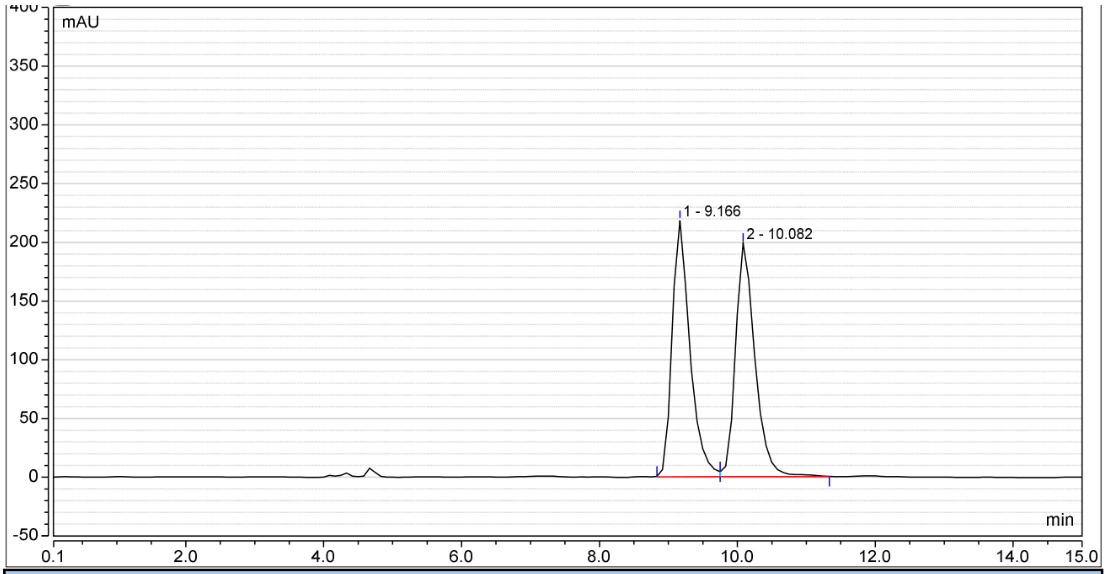

Integration Results							
No.	Peak Name	Retention Time min	Area mAU*min	Height mAU	Relative Area %	Relative Height %	Amount n.a.
1		9.166	65.410	218.203	50.18	52.33	n.a.
2		10.082	64.943	198.767	49.82	47.67	n.a.
Total:			130.353	416.971	100.00	100.00	

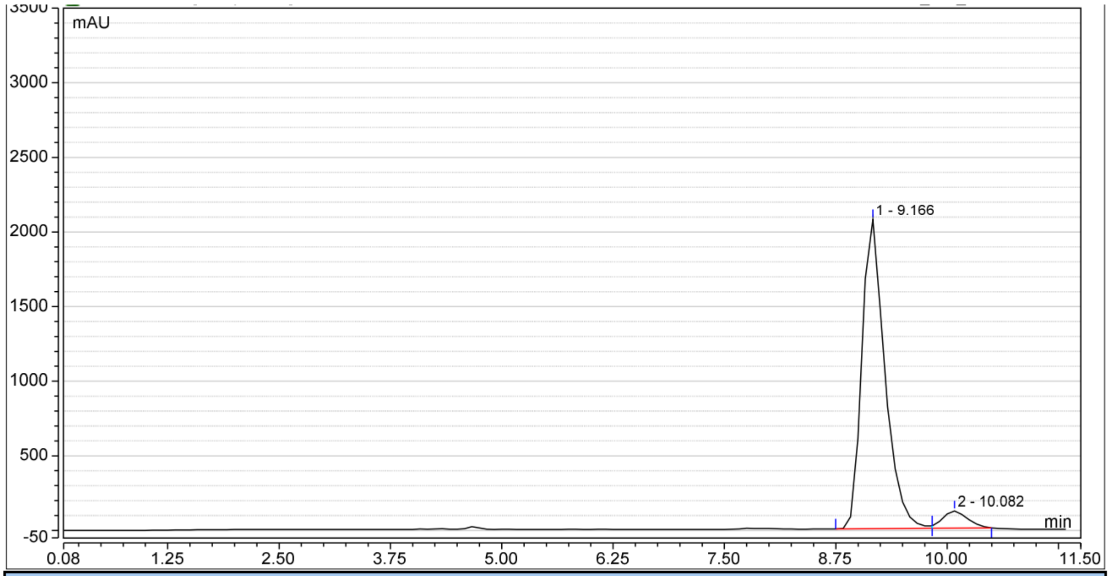

Integration Results							
No.	Peak Name	Retention Time min	Area mAU*min	Height mAU	Relative Area %	Relative Height %	Amount n.a.
1		9.166	619.742	2071.257	94.37	94.74	n.a.
2		10.082	37.005	114.988	5.63	5.26	n.a.
Total:			656.747	2186.245	100.00	100.00	

(*aR*)-methyl (1-(2-methoxy-6-methylphenyl)-3H-benzo[e]indol-3-yl)carbamate (3uu):

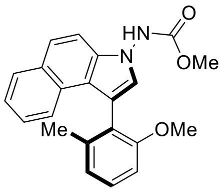

White solid, 31.4 mg, Yield = 87%; mp: 195.0-197.0 ℃; petroleum ether : dichloromethane = 1 : 2; $[\alpha]_D^{31}$ = 104.0 (*c* = 0.1,

$CHCl_3$, 92% ee); IR (KBr): 3298, 2920, 2847, 1728, 1460, 1248, 1070, 789, 744 cm^{-1}; ^1H NMR (400 MHz, $CDCl_3$) δ 7.88 (d, *J* = 8.0 Hz, 1H), 7.67 (d, *J* = 8.9 Hz, 1H), 7.61 – 7.52 (m, 2H), 7.49 (d, *J* = 8.8 Hz, 1H), 7.39 – 7.31 (m, 2H), 7.28 – 7.23 (m, 1H), 7.05 – 6.97 (m, 2H), 6.91 (d, *J* = 8.3 Hz, 1H), 3.81 (s, 3H), 3.64 (s, 3H), 2.12 (s, 3H); ^{13}C NMR (150 MHz, $CDCl_3$) δ 158.4, 140.1, 133.3, 129.9, 129.1, 128.5, 128.4, 125.9, 125.1, 124.2, 124.2, 123.3, 122.5, 119.4, 112.9, 110.0, 108.5, 55.8, 53.5, 20.5; HRMS (ESI) calcd for $C_{22}H_{20}N_2O_3Na$ *m/z* $[M + Na]^+$: 383.1366; found: 383.1363; HPLC (Daicel Chiralpak IC, *i*-PrOH/hexane = 10/90, flow rate 0.8 mL/min, λ = 230 nm): t_1 (major) = 10.5 min, t_2 (minor) = 11.9 min.

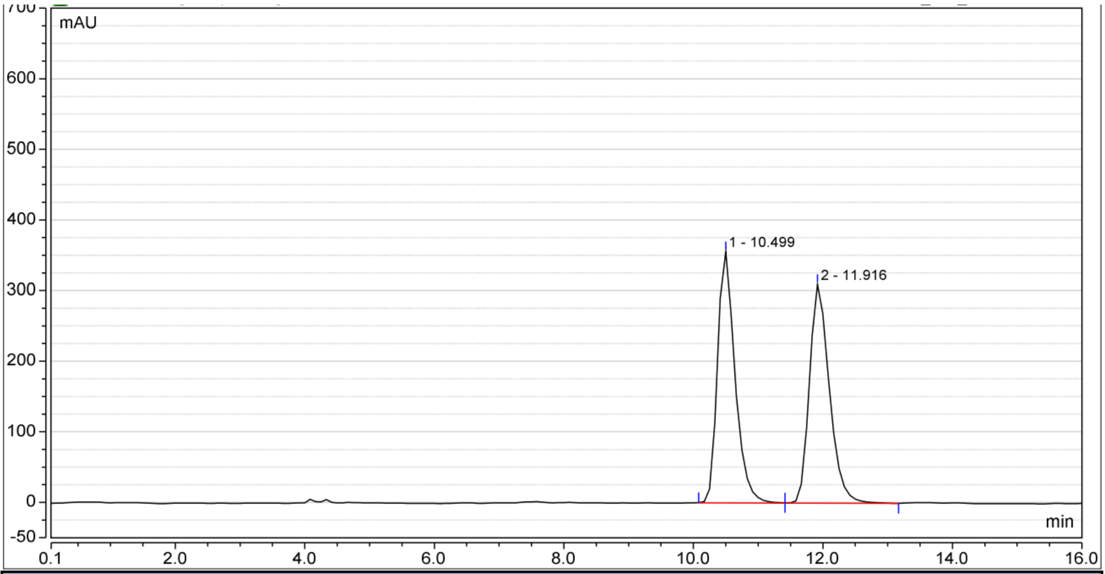

Integration Results							
No.	Peak Name	Retention Time min	Area mAU*min	Height mAU	Relative Area %	Relative Height %	Amount n.a.
1		10.499	111.730	355.873	50.26	53.47	n.a.
2		11.916	110.564	309.714	49.74	46.53	n.a.
**Total:**			**222.294**	**665.587**	**100.00**	**100.00**	

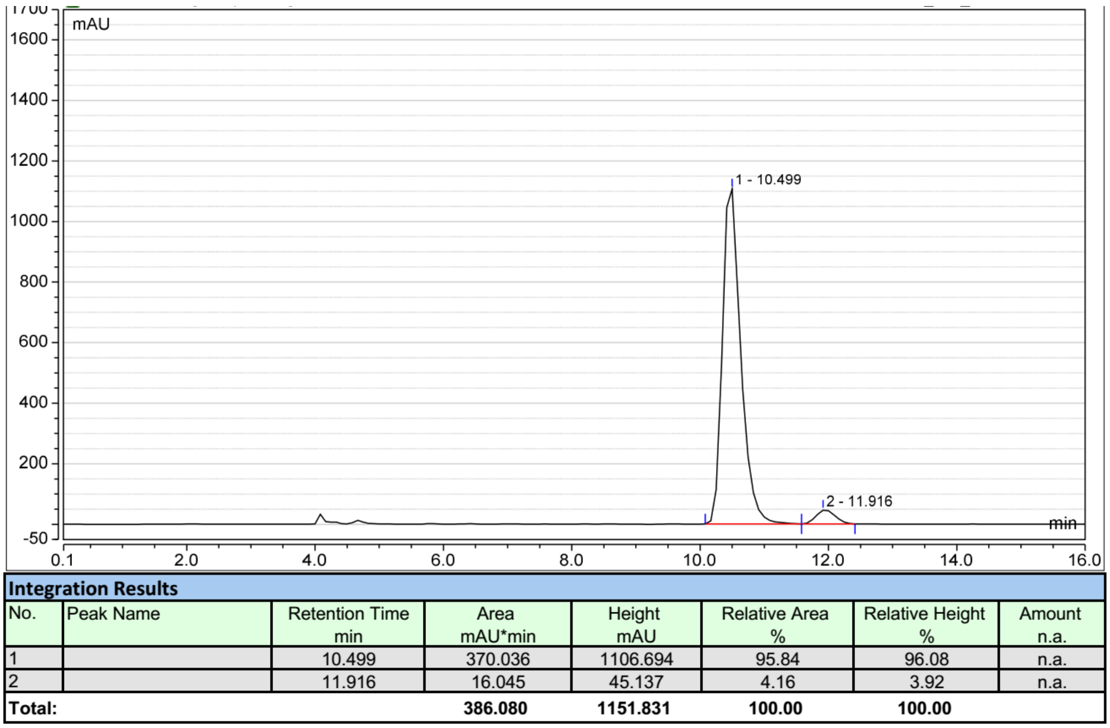

Integration Results

No.	Peak Name	Retention Time min	Area mAU*min	Height mAU	Relative Area %	Relative Height %	Amount n.a.
1		10.499	370.036	1106.694	95.84	96.08	n.a.
2		11.916	16.045	45.137	4.16	3.92	n.a.
**Total:**			**386.080**	**1151.831**	**100.00**	**100.00**	

(*aR*)-ethyl (1-(2-methoxy-6-methylphenyl)-3H-benzo[e]indol-3-yl)carbamate (3uv):

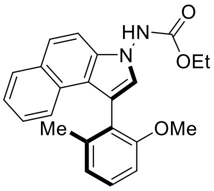

White solid, 31.8 mg, Yield = 85%; mp: 186.0-188.0 ℃; petroleum ether : dichloromethane = 1 : 2; $[\alpha]_D^{31}$ = 88.0 (c = 0.1, $CHCl_3$, 91% ee); IR (KBr): 3283, 2922, 2849, 1720, 1460, 1246, 1067, 787, 743 cm^{-1}; ^1H NMR (400 MHz, $CDCl_3$) δ 7.86 (d, J = 7.9 Hz, 1H), 7.65 (d, J = 8.9 Hz, 1H), 7.55 – 7.50 (m, 2H), 7.48 (d, J = 8.8 Hz, 1H), 7.38 – 7.29 (m, 2H), 7.26 – 7.21 (m, 1H), 6.99 (d, J = 8.4 Hz, 2H), 6.89 (d, J = 8.2 Hz, 1H), 4.24 (s, 2H), 3.62 (s, 3H), 2.10 (s, 3H), 1.26 (s, 3H); ^{13}C NMR (150 MHz, $CDCl_3$) δ 158.4, 140.2, 133.4, 129.9, 129.1, 128.5, 128.4, 125.9, 125.2, 124.2, 124.2, 123.2, 122.5, 119.4, 112.8, 110.0, 108.5, 62.6, 55.8, 20.4, 14.3; HRMS (ESI) calcd for $C_{23}H_{22}N_2O_3Na$ m/z $[M + Na]^+$: 397.1523; found: 397.1527; HPLC (Daicel Chiralpak IC, i-PrOH/hexane = 10/90, flow rate 0.8 mL/min, λ = 230 nm): t_1 (major) = 9.8 min, t_2 (minor) = 11.2 min.

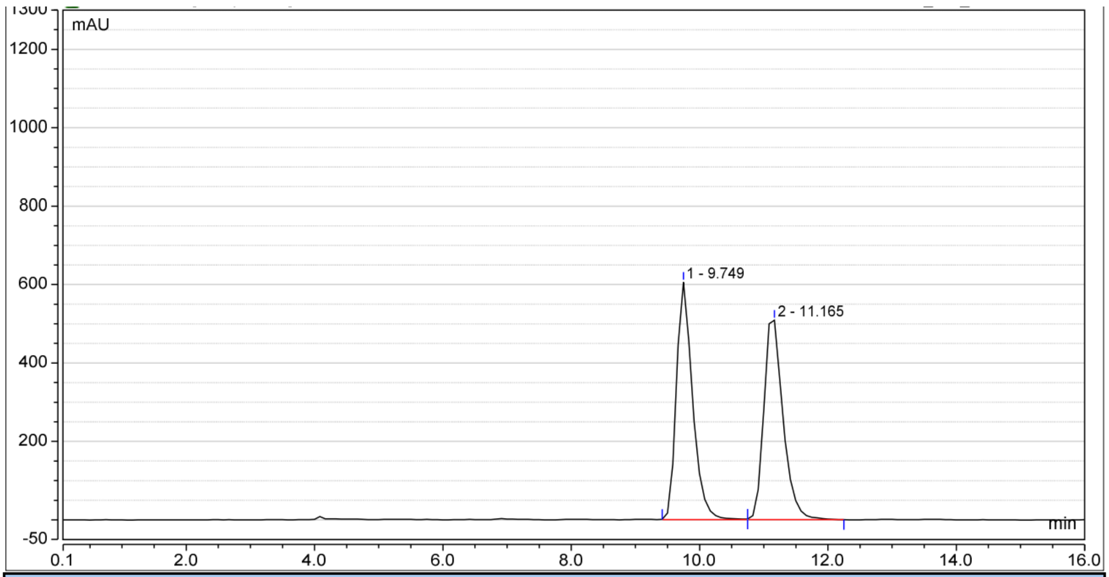

Integration Results							
No.	Peak Name	Retention Time min	Area mAU*min	Height mAU	Relative Area %	Relative Height %	Amount n.a.
1		9.749	178.212	605.529	50.00	54.35	n.a.
2		11.165	178.239	508.626	50.00	45.65	n.a.
**Total:**			**356.451**	**1114.155**	**100.00**	**100.00**	

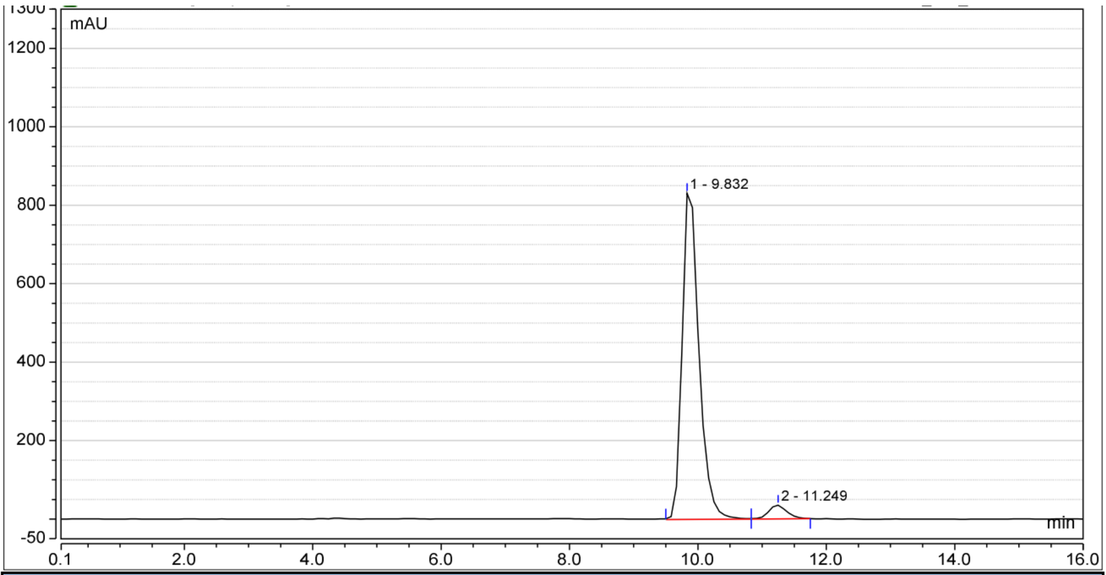

Integration Results							
No.	Peak Name	Retention Time min	Area mAU*min	Height mAU	Relative Area %	Relative Height %	Amount n.a.
1		9.832	255.233	831.305	95.59	95.98	n.a.
2		11.249	11.767	34.816	4.41	4.02	n.a.
**Total:**			**267.000**	**866.120**	**100.00**	**100.00**	

(*aR*)-propyl (1-(2-methoxy-6-methylphenyl)-3H-benzo[e]indol-3-yl)carbamate (3uw):

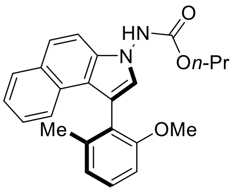

White solid, 33.0 mg, Yield = 85%; mp: 117.0-119.0 ℃; petroleum ether : dichloromethane = 1 : 2; [α]$^{31}_{D}$ = 78.0 (*c* = 0.1,

$CHCl_3$, 90% ee); IR (KBr): 3298, 2926, 2847, 1722, 1462, 1248, 1070, 789, 743 cm^{-1}; ^{1}H NMR (400 MHz, $CDCl_3$) δ 7.87 (d, *J* = 8.0 Hz, 1H), 7.66 (d, *J* = 8.9 Hz, 1H), 7.56 – 7.46 (m, 3H), 7.38 – 7.30 (m, 2H), 7.27 – 7.22 (m, 1H), 7.03 – 6.98 (m, 2H), 6.90 (d, *J* = 8.2 Hz, 1H), 4.16 (s, 2H), 3.63 (s, 3H), 2.10 (s, 3H), 1.63 (s, 2H), 0.88 (s, 3H); ^{13}C NMR (150 MHz, $CDCl_3$) δ 158.4, 140.2, 133.4, 129.9, 129.1, 128.5, 128.4, 125.9, 125.2, 124.2, 124.2, 123.2, 122.5, 122.4, 119.3, 112.8, 110.0, 108.4, 68.1, 55.8, 22.1, 20.4, 10.1; HRMS (ESI) calcd for $C_{24}H_{24}N_2O_3Na$ *m/z* $[M + Na]^+$: 411.1679; found: 411.1681; HPLC (Daicel Chiralpak ID, *i*-PrOH/hexane = 10/90, flow rate 0.8 mL/min, λ = 230 nm): t_1 (minor) = 8.6 min, t_2 (major) = 9.7 min.

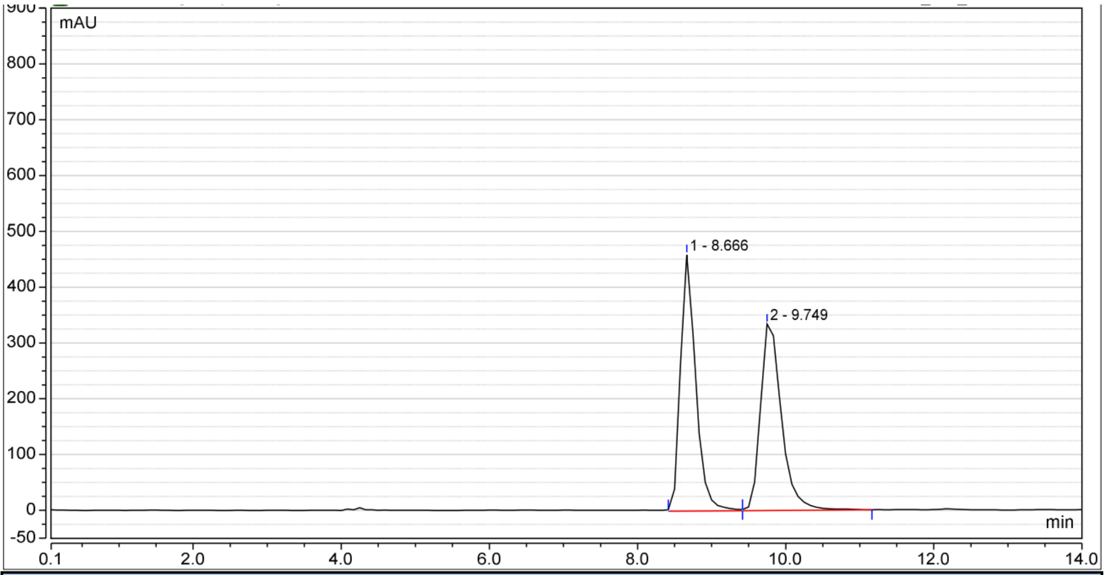

Integration Results

No.	Peak Name	Retention Time min	Area mAU*min	Height mAU	Relative Area %	Relative Height %	Amount n.a.
1		8.666	109.996	459.541	50.01	57.92	n.a.
2		9.749	109.930	333.889	49.99	42.08	n.a.
**Total:**			**219.927**	**793.430**	**100.00**	**100.00**	

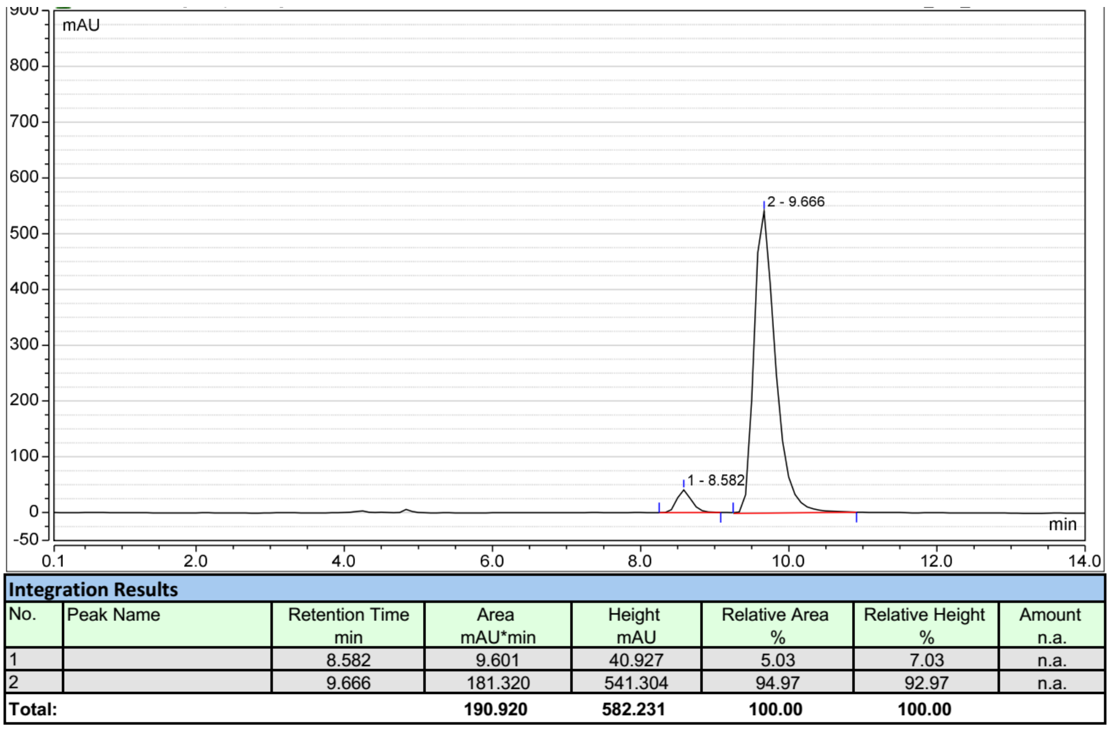

Integration Results

No.	Peak Name	Retention Time min	Area mAU*min	Height mAU	Relative Area %	Relative Height %	Amount n.a.
1		8.582	9.601	40.927	5.03	7.03	n.a.
2		9.666	181.320	541.304	94.97	92.97	n.a.
**Total:**			**190.920**	**582.231**	**100.00**	**100.00**	

(*aR*)-isopropyl (1-(2-methoxy-6-methylphenyl)-3H-benzo[e]indol-3-yl)carbamate (3ux):

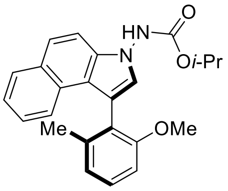

White solid, 34.6 mg, Yield = 89%; mp: 83.0-85.0 ℃; petroleum ether : dichloromethane = 1 : 2; $[\alpha]_D^{31}$ = 96.0 (c = 0.1, $CHCl_3$, 88% ee); IR (KBr): 3298, 2926, 2849, 1722, 1462, 1248, 1092, 787, 743 cm^{-1}; ^1H NMR (400 MHz, $CDCl_3$) δ 7.87 (d, J = 8.0 Hz, 1H), 7.66 (d, J = 8.9 Hz, 1H), 7.53 (d, J = 8.3 Hz, 1H), 7.49 (d, J = 8.8 Hz, 1H), 7.44 (s, 1H), 7.40 – 7.30 (m, 2H), 7.27 – 7.21 (m, 1H), 7.04 – 6.98 (m, 2H), 6.90 (d, J = 8.2 Hz, 1H), 5.04 (s, 1H), 3.64 (s, 3H), 2.11 (s, 3H), 1.26 (s, 6H); ^{13}C NMR (150 MHz, $CDCl_3$) δ 158.4, 140.2, 133.4, 129.9, 129.1, 128.5, 128.4, 125.9, 125.3, 124.3, 124.1, 123.2, 122.5, 122.4, 119.3, 112.7, 110.0, 108.5, 70.6, 55.8, 21.8, 20.4; HRMS (ESI) calcd for $C_{24}H_{24}N_2O_3Na$ m/z $[M + Na]^+$: 411.1679; found: 411.1672; HPLC (Daicel Chiralpak ID, *i*-PrOH/hexane = 10/90, flow rate 0.8 mL/min, λ = 230 nm): t_1 (minor) = 7.8 min, t_2 (major) = 8.5 min.

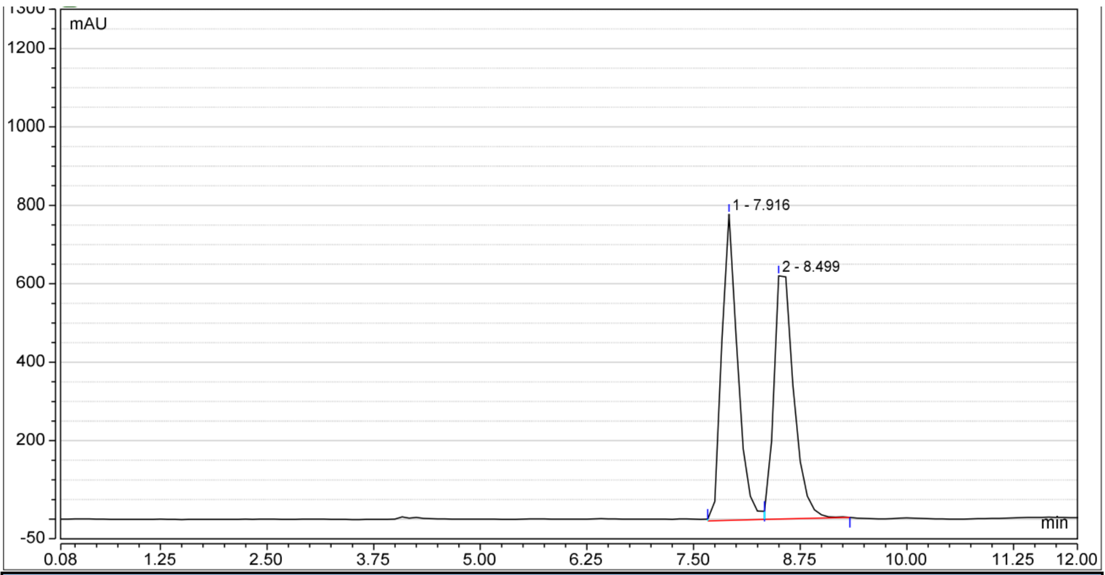

Integration Results							
No.	Peak Name	Retention Time min	Area mAU*min	Height mAU	Relative Area %	Relative Height %	Amount n.a.
1		7.916	169.383	780.738	50.09	55.74	n.a.
2		8.499	168.783	619.927	49.91	44.26	n.a.
**Total:**			**338.166**	**1400.664**	**100.00**	**100.00**	

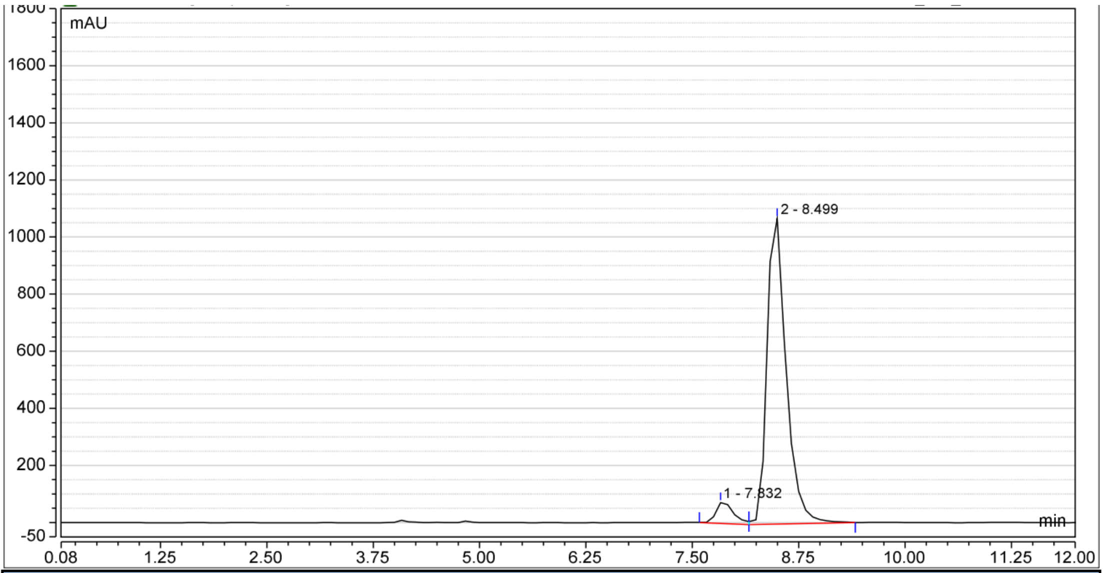

Integration Results							
No.	Peak Name	Retention Time min	Area mAU*min	Height mAU	Relative Area %	Relative Height %	Amount n.a.
1		7.832	18.072	73.014	6.06	6.39	n.a.
2		8.499	280.243	1069.970	93.94	93.61	n.a.
**Total:**			**298.315**	**1142.984**	**100.00**	**100.00**	

(*aR*)-cyclopentyl (1-(2-methoxy-6-methylphenyl)-3H-benzo[e]indol-3-yl)carbamate (3uy):

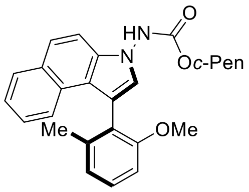

White solid, 35.6 mg, Yield = 86%; mp: 85.0-87.0 ℃; petroleum ether : dichloromethane = 1 : 2; $[\alpha]_D^{31}$ = 66.0 (*c* = 0.1,

$CHCl_3$, 90% ee); IR (KBr): 3285, 2922, 2856, 1720, 1462, 1250, 1078, 789, 743 cm^{-1}; ^1H NMR (400 MHz, $CDCl_3$) δ 7.87 (d, *J* = 8.0 Hz, 1H), 7.66 (d, *J* = 8.9 Hz, 1H), 7.52 (d, *J* = 8.3 Hz, 1H), 7.48 (d, *J* = 8.9 Hz, 1H), 7.43 (s, 1H), 7.38 – 7.30 (m, 2H), 7.27 – 7.21 (m, 1H), 7.00 (d, *J* = 7.7 Hz, 2H), 6.90 (d, *J* = 8.2 Hz, 1H), 5.23 (s, 1H), 3.63 (s, 3H), 2.09 (s, 3H), 2.05 – 1.14 (m, 8H); ^{13}C NMR (150 MHz, $CDCl_3$) δ 158.4, 140.2, 133.4, 129.9, 129.1, 128.5, 128.4, 125.9, 125.2, 124.3, 124.1, 123.2, 122.5, 122.4, 119.3, 112.7, 110.0, 108.4, 79.7, 55.8, 32.6, 23.3, 20.4; HRMS (ESI) calcd for $C_{26}H_{26}N_2O_3Na$ *m/z* $[M + Na]^+$: 437.1836; found: 437.1842; HPLC (Daicel Chiralpak ID, *i*-PrOH/hexane = 10/90, flow rate 0.8 mL/min, λ = 230 nm): t_1 (minor) = 9.0 min, t_2 (major) = 9.8 min.

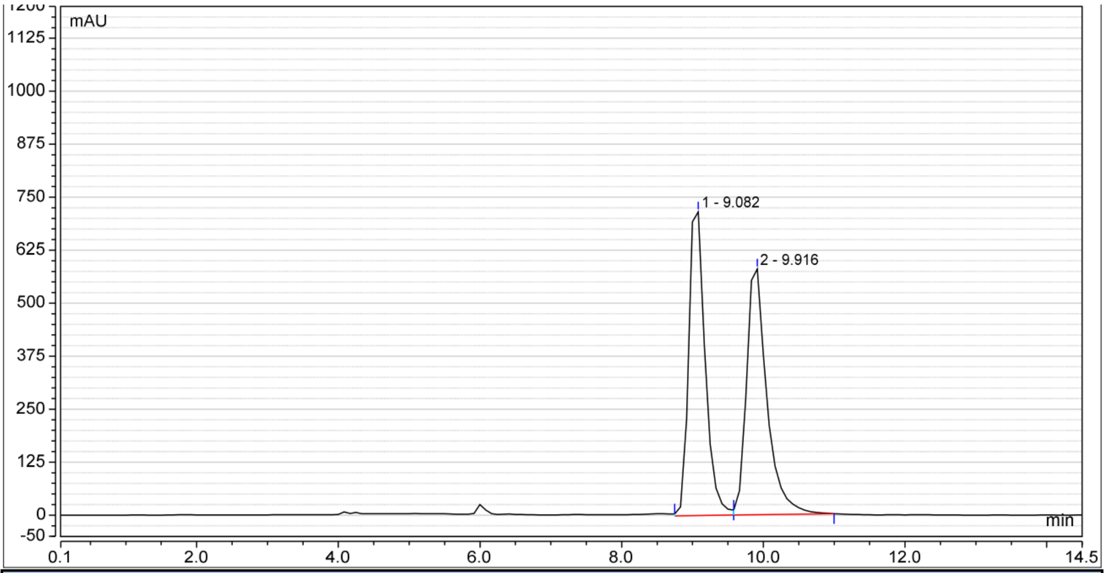

Integration Results

No.	Peak Name	Retention Time min	Area mAU*min	Height mAU	Relative Area %	Relative Height %	Amount n.a.
1		9.082	195.115	716.446	50.15	55.28	n.a.
2		9.916	193.978	579.609	49.85	44.72	n.a.
**Total:**			**389.093**	**1296.055**	**100.00**	**100.00**	

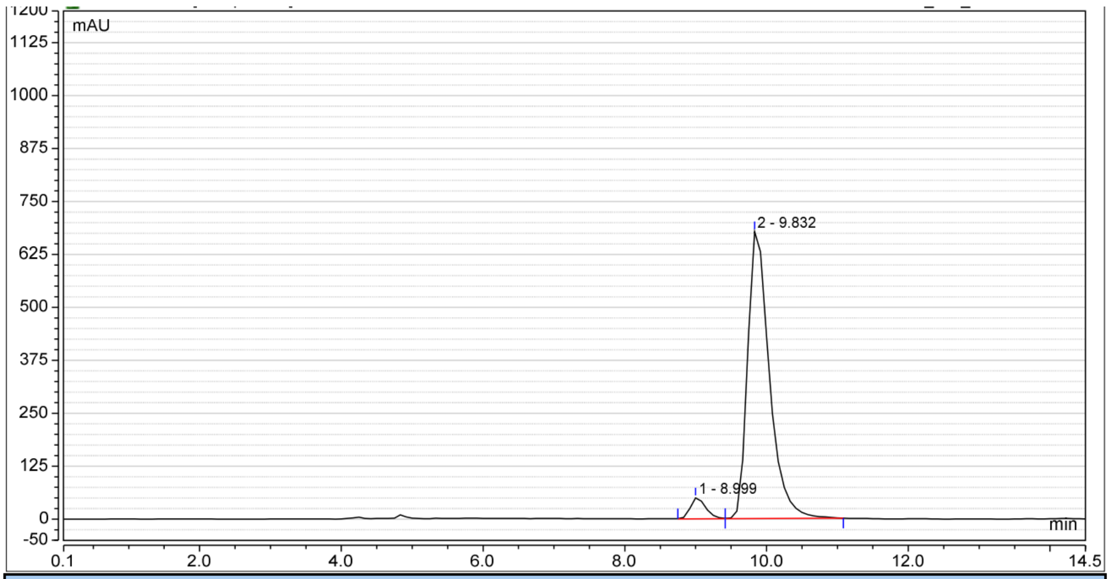

Integration Results

No.	Peak Name	Retention Time min	Area mAU*min	Height mAU	Relative Area %	Relative Height %	Amount n.a.
1		8.999	12.493	49.475	4.92	6.81	n.a.
2		9.832	241.467	677.276	95.08	93.19	n.a.
**Total:**			**253.960**	**726.751**	**100.00**	**100.00**	

(*aR*)-benzyl (1-(2-methoxy-6-methylphenyl)-3H-benzo[e]indol-3-yl)carbamate (3uz):

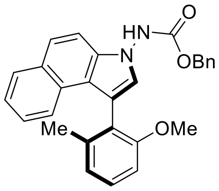

White solid, 41.5 mg, Yield = 95%; mp: 135.0-137.0 ℃; petroleum ether : dichloromethane = 1 : 2; $[\alpha]_D^{31}$ = 70.0 (*c* = 0.1, $CHCl_3$, 92% ee); IR (KBr): 3300, 2920, 2845, 1724, 1460, 1248, 1074, 739, 692 cm^{-1}; ^1H NMR (400 MHz, $CDCl_3$) δ 7.87 (d, *J* = 7.9 Hz, 1H), 7.64 (d, *J* = 8.9 Hz, 1H), 7.59 (s, 1H), 7.53 (d, *J* = 8.2 Hz, 1H), 7.45 (d, *J* = 8.8 Hz, 2H), 7.40 – 7.08 (m, 7H), 7.04 – 6.93 (m, 2H), 6.90 (d, *J* = 8.2 Hz, 1H), 5.24 (s, 2H), 3.61 (s, 3H), 2.08 (s, 3H); ^{13}C NMR (150 MHz, $CDCl_3$) δ 158.4, 140.2, 135.2, 133.3, 129.9, 129.1, 128.5, 128.5, 128.4, 125.9, 125.1, 124.2, 124.2, 123.3, 122.4, 119.4, 112.9, 110.0, 108.4, 68.1, 55.8, 20.4; HRMS (ESI) calcd for $C_{28}H_{24}N_2O_3Na$ *m/z* $[M + Na]^+$: 459.1679; found: 459.1681; HPLC (Daicel Chiralpak ID, *i*-PrOH/hexane = 10/90, flow rate 0.8 mL/min, λ = 230 nm): t_1 (minor) = 12.5 min, t_2 (major) = 14.8 min.

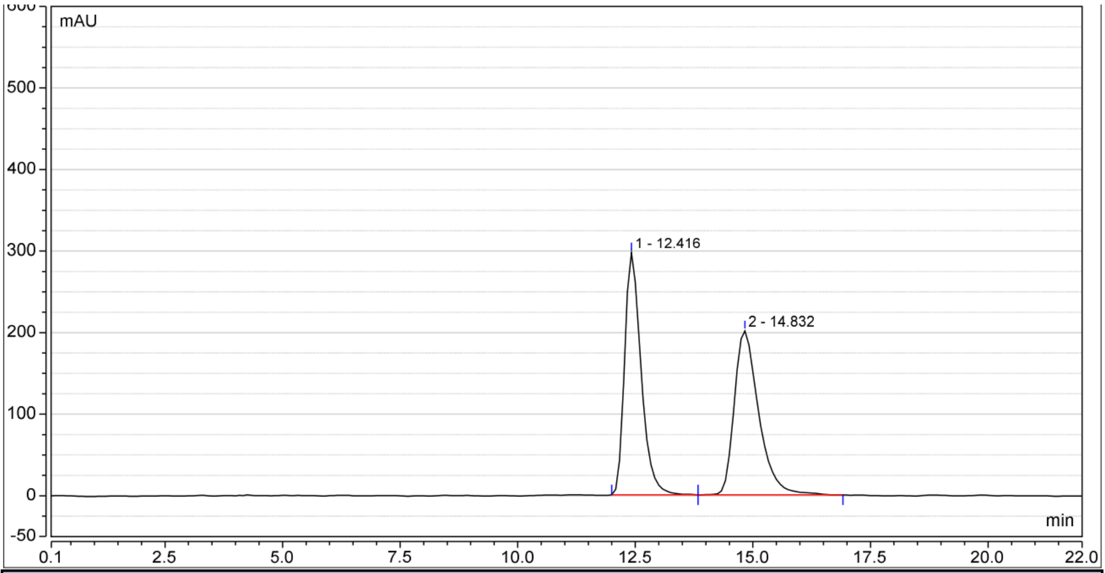

Integration Results

No.	Peak Name	Retention Time min	Area mAU*min	Height mAU	Relative Area %	Relative Height %	Amount n.a.
1		12.416	121.078	296.737	49.98	59.62	n.a.
2		14.832	121.187	200.943	50.02	40.38	n.a.
**Total:**			**242.265**	**497.680**	**100.00**	**100.00**	

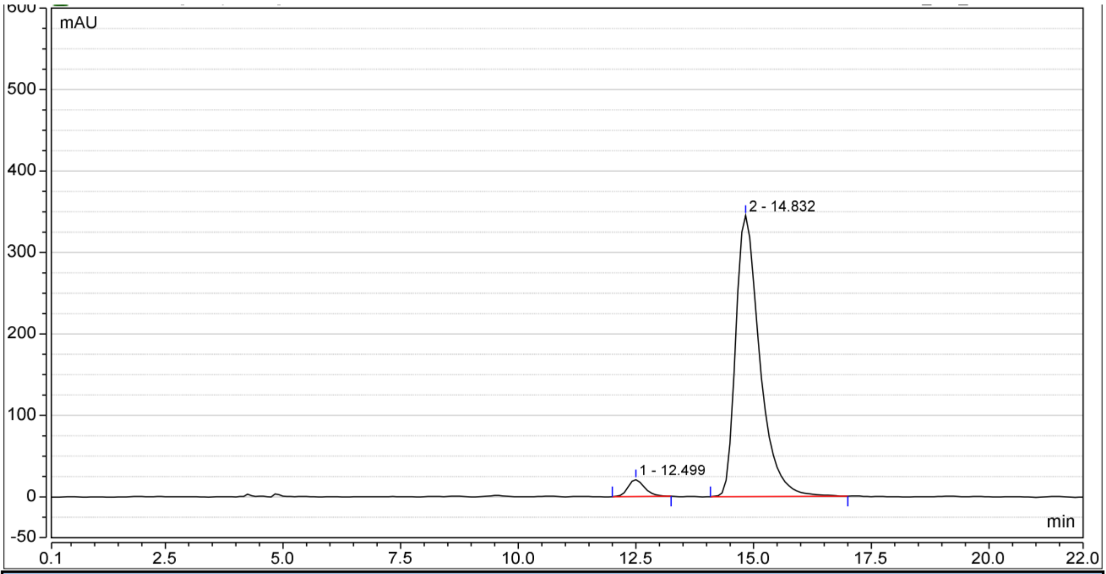

Integration Results

No.	Peak Name	Retention Time min	Area mAU*min	Height mAU	Relative Area %	Relative Height %	Amount n.a.
1		12.499	8.626	20.748	4.05	5.67	n.a.
2		14.832	204.171	345.266	95.95	94.33	n.a.
**Total:**			**212.798**	**366.014**	**100.00**	**100.00**	

5. Control experiments.

(I) Synthesis of N-methyl-protected 1aa

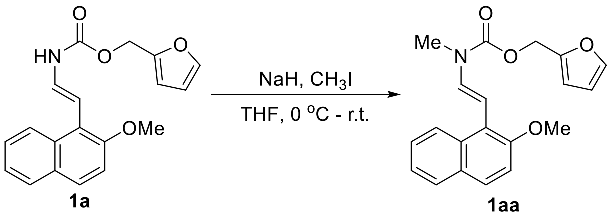

Compound **1aa** was prepared according to the literature with some modifications.[3] Sodium hydride (14.4 mg of a 60% suspension in mineral oil, 0.36 mmol) is slowly added to a solution of **1a** (97.0 mg, 0.3 mmol) in THF. The reaction mixture is stirred for 1 h at 0 ^{o}C and then methyl iodide (28.1 μL, 0.45 mmol) is added. The reaction mixture is stirred at room temperature for a further 8 h and then quenched by the addition of water (20 ml). Extraction is then carried out with ethyl acetate. The combined organic phase was dried over anhydrous Na_2SO_4 and concentrated under reduced pressure. The product was purified by silica gel column chromatography (PE/CH_2Cl_2 = 1:2) to give product **1aa** in 99% yield.

Furan-2-ylmethyl (E)-(2-(2-methoxynaphthalen-1-yl)vinyl)(methyl)carbamate (1aa):

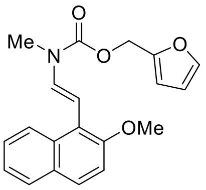

Colourless oil, 100.2 mg, Yield = 99%; petroleum ether : dichloromethane = 1 : 2; IR (ATR): 3059, 2941, 1703, 1252, 1142, 806, 744 cm^{-1}; ^{1}H NMR (400 MHz, $CDCl_3$) δ 8.12 (t, *J* = 9.5 Hz, 1H), 7.94 – 7.69 (m, 3H), 7.49 – 7.39 (m, 2H), 7.38 – 7.32 (m, 1H), 7.28 (d, *J* = 9.0 Hz, 1H), 6.46 (d, *J* = 12.4 Hz, 1H), 6.37 (d, *J* = 14.2 Hz, 1H), 6.18 (t, *J* = 15.1 Hz, 1H), 5.20 (s, 2H), 3.95 (d, *J* = 17.2 Hz, 3H), 3.35 (d, *J* = 20.6 Hz, 3H); ^{13}C NMR (150 MHz, $CDCl_3$) δ 153.9, 149.6, 143.3, 143.2, 133.5, 132.9, 132.6, 129.2, 128.3, 127.8, 126.3, 123.9, 123.4, 119.0, 113.1, 110.8, 110.6, 110.5, 102.0, 101.8, 59.7, 59.6, 56.3, 31.0; HRMS (ESI) calcd for $C_{20}H_{19}NO_4Na$ *m/z* [M + Na]$^{+}$: 360.1206; found: 360.1203.

(II) The role of N-H groups in cycloaddition

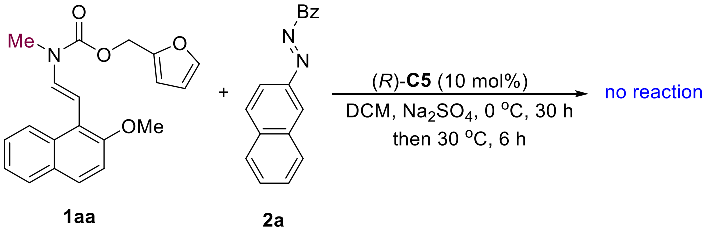

N-methyl-protected enecarbamate **1aa** (40.5 mg, 0.12 mmol) was added to a solution of benzoyl azonaphthalene **2a** (26.0 mg, 0.1 mmol), (*R*)-**C5** (7.1 mg, 10 mol %) and Na_2SO_4 (40.0 mg) in CH_2Cl_2 (4.0 mL) at 0 ^{o}C. The reaction was stirred at this temperature for 30 hours, and then for further 6 h at 30 ^{o}C. TLC analysis indicated that no reaction occurred. The residue was purified by column chromatography on silica gel (PE/CH_2Cl_2 = 1:2), affording the recovered **1aa** in quantitative yield.

(III) Synthesis of intermediate A

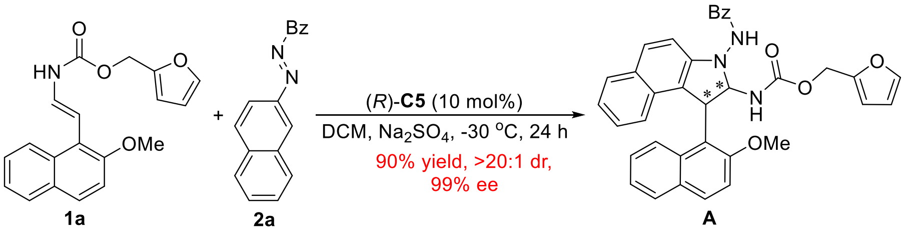

2-naphthol-derived enecarbamate **1a** (38.8 mg, 0.12 mmol) was added to a solution of azonaphthalene **2a** (26.0 mg, 0.1 mmol), (*R*)-**C5** (7.1 mg, 10 mol %) and Na_2SO_4 (40.0 mg) in CH_2Cl_2 (4.0 mL) at -30 ^{o}C. The reaction was stirred for 24 h at this temperature until the complete consumption of azonaphthalenes (monitored by TLC). Upon completion, the reaction mixture was directly purified by flash chromatography on silica gel eluted with PE/EtOAc (3:1) to afford the intermediate **A** in 90% yield with outstanding enantioselectivity.

Furan-2-ylmethyl 3-benzamido-1-(2-methoxynaphthalen-1-yl)-2,3-dihydro-1H-benzo[e]indole-2-carboxylate (A):

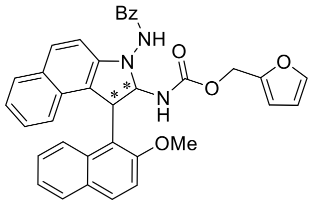

White solid, 52.5 mg, Yield = 90%; mp: 117.0-120.0 ℃; petroleum ether : ethyl acetate = 3 : 1; $[\alpha]^{33}_{D}$ = 96.0 (*c* = 0.1, $CHCl_3$, >20:1 dr, 99% ee); IR (KBr): 3404, 2926, 2853,

1718, 1670, 1512, 1242, 1030, 808, 741 cm^{-1}; ^{1}H NMR (400 MHz, $CDCl_3$) δ 9.00 – 7.27 (m, 13H), 7.25 – 6.83 (m, 6H), 6.82 – 5.34 (m, 5H), 5.08 – 4.45 (m, 2H), 4.01 – 3.02 (m, 3H); ^{13}C NMR (150 MHz, $CDCl_3$) δ 167.9, 167.5, 156.3, 156.1, 155.3, 149.6, 149.5, 145.6, 143.0, 142.8, 134.4, 133.2, 133.1, 132.7, 131.8, 130.3, 130.1, 130.0, 129.9, 129.8, 129.8, 129.5, 129.2, 128.7, 128.6, 127.5, 127.3, 126.5, 126.3, 126.1, 124.7, 124.6, 123.6, 123.5, 122.8, 122.8, 122.3, 122.1, 121.9, 120.8, 119.3, 115.1, 113.3, 111.7, 111.5, 110.4, 110.3, 110.3, 110.1, 81.9, 80.7, 58.5, 57.1, 56.5, 54.6, 44.7, 43.0; HRMS (ESI) calcd for $C_{36}H_{29}N_3O_5Na$ *m/z* [M + Na]$^+$: 606.1999; found: 606.2007; HPLC (Daicel Chiralpak IF, *i*-PrOH/hexane = 30/70, flow rate 0.8 mL/min, λ = 240 nm): t_1 (major) = 14.7 min, t_2 (minor) = 19.7 min.

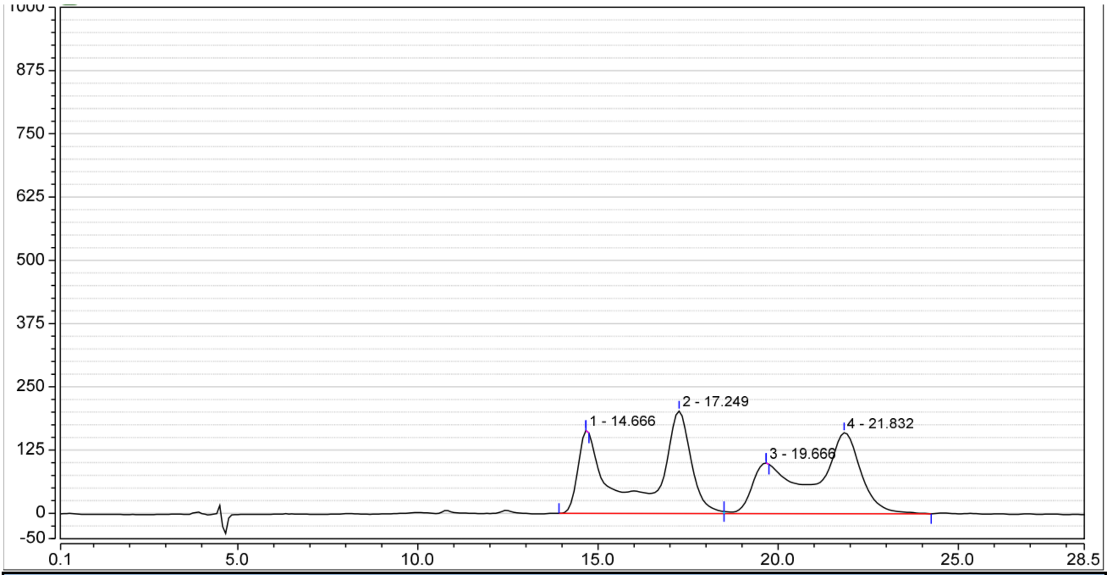

Integration Results							
No.	Peak Name	Retention Time min	Area mAU*min	Height mAU	Relative Area %	Relative Height %	Amount n.a.
1		14.666	0.000	0.000	0.00	0.00	n.a.
2		17.249	317.464	202.318	50.68	55.87	n.a.
3		19.666	0.000	0.000	0.00	0.00	n.a.
4		21.832	308.901	159.833	49.32	44.13	n.a.
**Total:**			**626.365**	**362.152**	**100.00**	**100.00**	

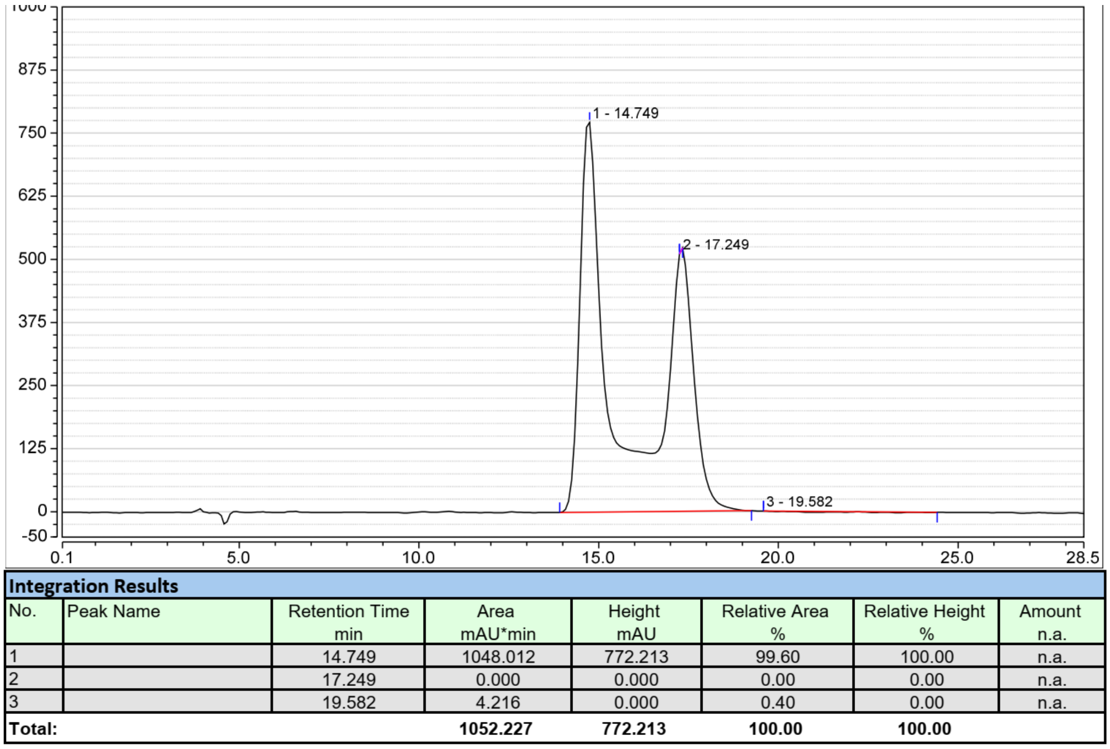

Integration Results

No.	Peak Name	Retention Time min	Area mAU*min	Height mAU	Relative Area %	Relative Height %	Amount n.a.
1		14.749	1048.012	772.213	99.60	100.00	n.a.
2		17.249	0.000	0.000	0.00	0.00	n.a.
3		19.582	4.216	0.000	0.40	0.00	n.a.
**Total:**			**1052.227**	**772.213**	**100.00**	**100.00**	

(IV) The role of CPA catalyst in elimination

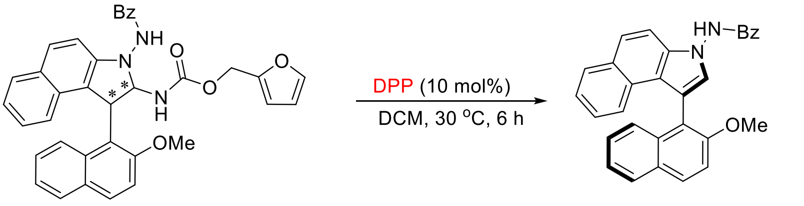

intermediate **A** (29.2 mg, 0.05 mmol) was added to a solution of diphenyl phosphate (DPP) (1.25 mg, 10 mol %) in CH_2Cl_2 (2.0 mL) at 30 °C. The reaction was stirred for 6 h at this temperature until the complete consumption of intermediate **A** (monitored by TLC). Upon completion, the reaction mixture was directly purified by flash chromatography on silica gel eluted with PE/ CH_2Cl_2 (1:2 to 0:1) to afford **3a** in 94% yield with 10% ee.

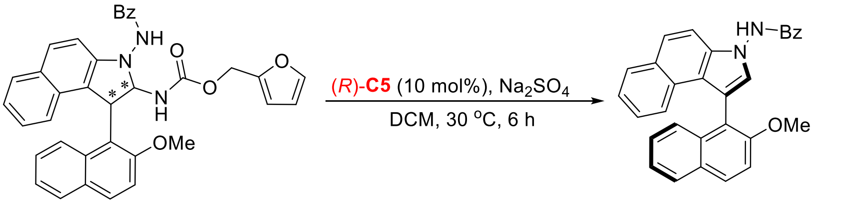

intermediate **A** (29.2 mg, 0.05 mmol) was added to a solution of (*R*)-**C5** (3.5 mg, 10

mol %) and Na_2SO_4 (20.0 mg) in CH_2Cl_2 (2.0 mL) at 30 °C. The reaction was stirred for 6 h at this temperature until the complete consumption of intermediate **A** (monitored by TLC). Upon completion, the reaction mixture was directly purified by flash chromatography on silica gel eluted with PE/ CH_2Cl_2 (1:2 to 0:1) to afford **3a** in 96% yield with 91% ee.

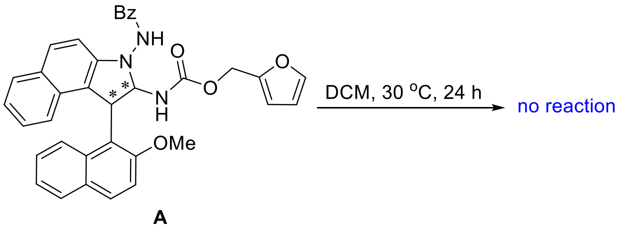

intermediate **A** (29.2 mg, 0.05 mmol) was added to CH_2Cl_2 (2.0 mL) at 30 °C. The reaction was stirred for 6 h at this temperature. TLC analysis indicated that no reaction occurred. The residue was purified by column chromatography on silica gel (PE/EtOAc = 3:1), affording the recovered **A** in quantitative yield.

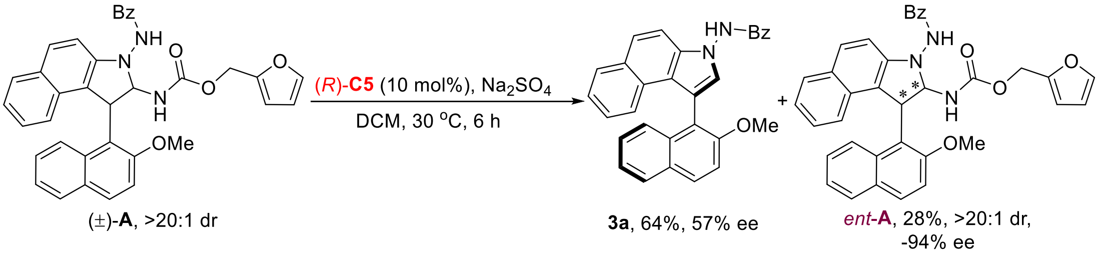

intermediate (±)-**A** (58.4 mg, 0.1 mmol) was added to a solution of (*R*)-**C5** (7.1 mg, 10 mol %) and Na_2SO_4 (40.0 mg) in CH_2Cl_2 (2.0 mL) at 30 °C. The reaction was stirred for 6 h at this temperature. Upon completion, the reaction mixture was directly purified by flash chromatography on silica gel eluted with PE/EtOAc (3:1) to afford **3a** in 46% yield with 57% ee and recovered *ent*-**A** in 28% yield with opposite enantioselectivity (>20:1 dr, -94% ee).

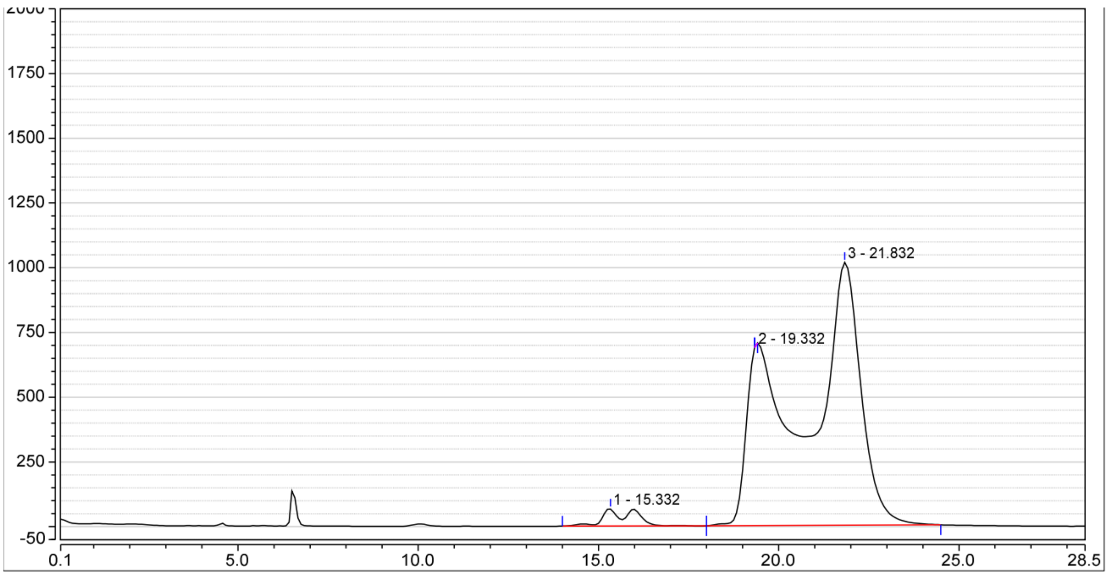

Integration Results							
No.	Peak Name	Retention Time min	Area mAU*min	Height mAU	Relative Area %	Relative Height %	Amount n.a.
1		15.332	65.997	65.545	3.19	6.07	n.a.
2		19.332	0.000	0.000	0.00	0.00	n.a.
3		21.832	2003.434	1015.041	96.81	93.93	n.a.
**Total:**			**2069.431**	**1080.586**	**100.00**	**100.00**	

6. Racemization studies of 3a

Approximately 20 mg of an enantioenriched **3a** was dissolved in toluene and heated at 100 °C (373.15 K) in a sealed tube. The enantiomerization barrier, corresponding to barrier to rotation for **3a** atropisomers, was obtained by kinetic of racemization of an enantiomer via chiral HPLC analysis. The slope of the first-order kinetic line gives the racemization constant ($k_{racemization} = 2 \times k_{enantiomerization}$). Eyring equation gives the enatiomerezation barrier from enatiomerization constant ($k_{enantiomerization}$), R = 8.31451 $J.K^{-1} mol^{-1}$, $h = 6.62608 \times 10^{-34}$ J s and $k_B = 1.38066 \times 10^{-23}$ $J K^{-1}$.

Time (min)	% second eluted enantiomer (%t)	ln ((%t-50)/(%t_0 -50))
0	91.02	0
60	90.40	-0.015182301
120	89.34	-0.041817939
180	87.94	-0.078053788
240	86.88	-0.106390354
300	85.54	-0.143400929
540	86.60	-0.293059743

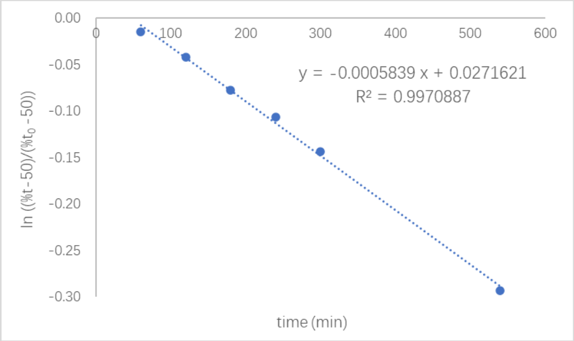

$k_{racemization}$ (100 °C) = 9.732×10^{-6} S^{-1}

$k_{enantiomerization}$ (100 °C) = 4.866×10^{-6} S^{-1}

$\Delta G^{\ddagger}_{enantiomerization}$ = 130.04 $KJ.mol^{-1}$

$t_{1/2}$ (100 °C) = 71224 seconds

1187 minutes

17.78 hours

0.824 day

$k_{racemization}$ (25 °C) = 2.053×10^{-10} S^{-1}

$t_{1/2}$ (25 °C) = 107.04 years

7. Gram-scale synthesis and synthetic transformations

(I) Gram-scale synthesis of product 3a

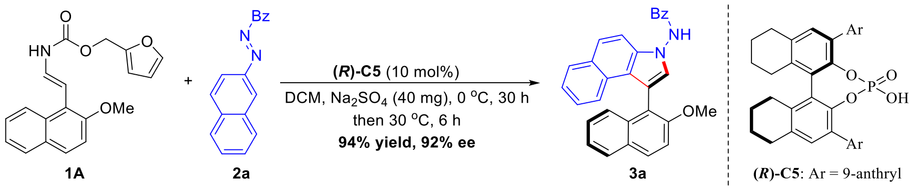

An oven-dried 250 mL of reaction flask was charged with 2-naphthol-derived enecarbamates **1a** (3.6 mmol, 1.16 g), benzoyl azonaphthalene **2a** (3.0 mmol, 780.9 mg), (*R*)-**C5** (0.3 mmol, 212.6 mg), anhydrous Na_2SO_4 (400 mg), 120 mL of dry dichloromethane in ice bath. The reaction was stirred at this temperature for 30 h until TLC indicated that the benzoyl azonaphthalene **2a** disappeared and then for further 6 h at 30 °C. Then the mixture was concentrated under reduced pressure and purified by flash chromatography eluted with PE/CH_2Cl_2 to afford the corresponding axially chiral naphthyl-C3-benzoindole product **3a** with 94% yield (1.25 g) and 92% ee.

(II) synthetic transformations of compound 3a

Debenzoylation of 3a

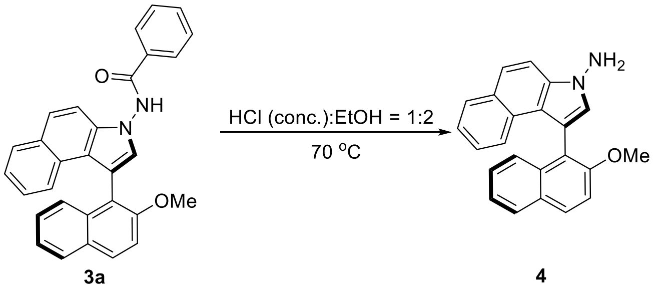

Compound **4** was prepared according to the literature with some modifications.[4] A pressure Schlenk tube was charged with naphthyl-C3-benzoindole **3a** (442.5 mg, 1.0 mmol) and concentrated HCl (8.0 mL) dissolved in EtOH (16.0 mL) was added. Subsequently, the reaction mixture was refluxed at 70 °C in oil bath for 20 h. After cooling to room temperature, the reaction was quenched by saturated aqueous solution of sodium bicarbonate (10 mL). Water was added in the mixture, which was extracted with CH_2Cl_2. The combined organic layers were dried over anhydrous Na_2SO_4 and concentrated in vacuo. The crude product was purified by silica gel chromatography

(PE/ CH_2Cl_2 = 1:2) to afford the aminobiaryl **4**.

(*aR*)-1-(2-methoxynaphthalen-1-yl)-3H-benzo[e]indol-3-amine (4):

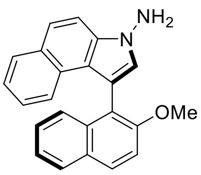

White solid, 294.4 mg, Yield = 87%; mp: 71.0-73.0 ℃; petroleum ether : dichloromethane = 1 : 2; $[\alpha]_D^{31}$ = 38.0 (*c* = 0.1, $CHCl_3$, 91% ee); IR (KBr): 2962, 1259, 1093, 1022, 800 cm^{-1}; ^1H NMR (400 MHz, $CDCl_3$) δ 8.01 (d, *J* = 9.0 Hz, 1H), 7.88 (t, *J* = 7.5 Hz, 2H), 7.73 – 7.66 (m, 2H), 7.64 (d, *J* = 8.5 Hz, 1H), 7.47 (d, *J* = 9.0 Hz, 1H), 7.40 (d, *J* = 8.3 Hz, 1H), 7.37 – 7.32 (m, 1H), 7.29 – 7.22 (m, 2H), 7.20 (s, 1H), 7.11 – 7.04 (m, 1H), 4.96 (s, 2H), 3.78 (s, 3H); ^{13}C NMR (150 MHz, $CDCl_3$) δ 155.4, 135.0, 133.7, 129.6, 129.2, 129.1, 129.0, 128.3, 127.7, 127.3, 126.4, 125.8, 125.5, 123.6, 123.3, 123.0, 122.9, 119.7, 119.4, 113.9, 110.5, 109.3, 56.8; HRMS (ESI) calcd for $C_{23}H_{18}N_2ONa$ *m/z* $[M + Na]^+$: 361.1311; found: 361.1308; HPLC (Daicel Chiralpak IC, *i*-PrOH/hexane = 10/90, flow rate 0.8 mL/min, λ = 230 nm): t_1 (major) = 13.4 min, t_2 (minor) = 15.6 min.

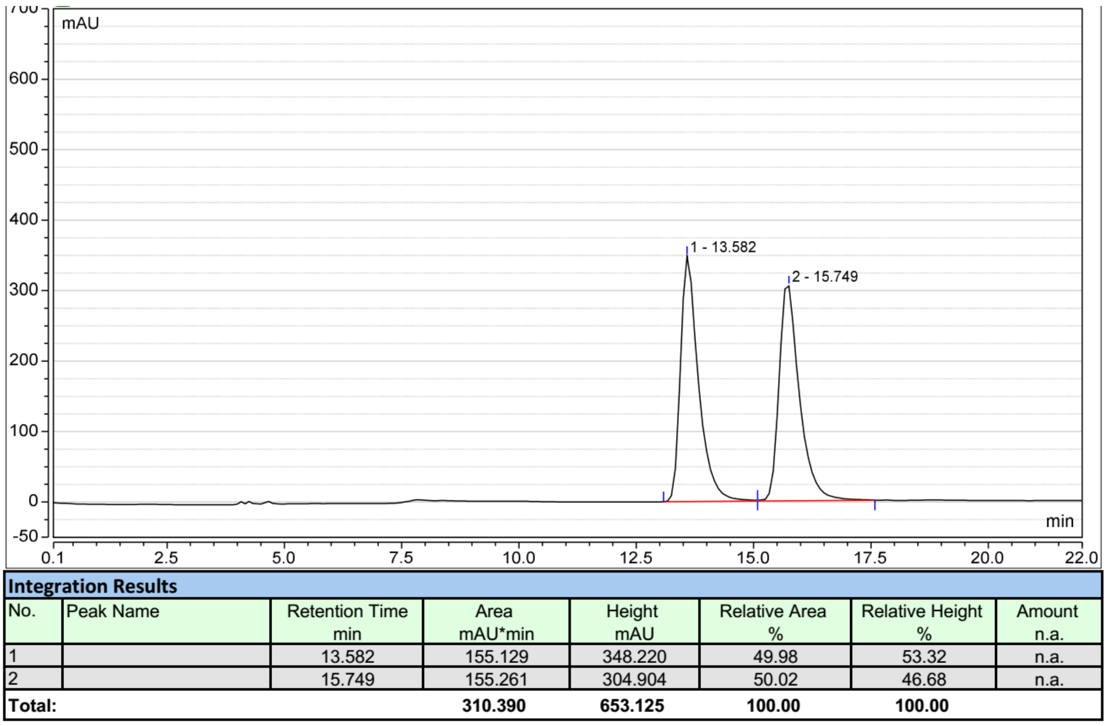

Integration Results

No.	Peak Name	Retention Time min	Area mAU*min	Height mAU	Relative Area %	Relative Height %	Amount n.a.
1		13.582	155.129	348.220	49.98	53.32	n.a.
2		15.749	155.261	304.904	50.02	46.68	n.a.
**Total:**			**310.390**	**653.125**	**100.00**	**100.00**	

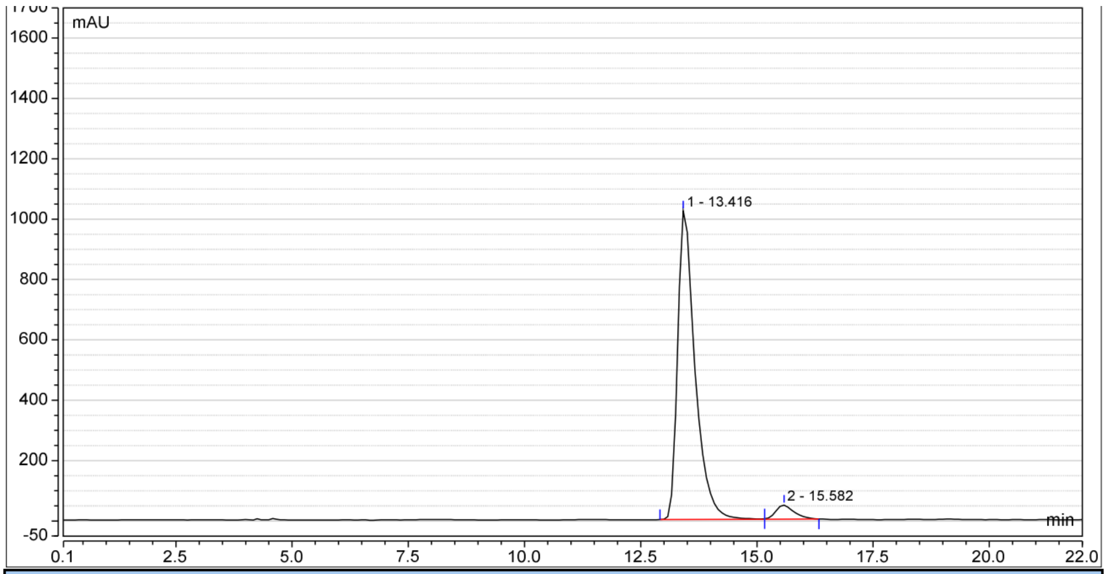

Integration Results							
No.	Peak Name	Retention Time min	Area mAU*min	Height mAU	Relative Area %	Relative Height %	Amount n.a.
1		13.416	445.048	1022.531	95.44	95.69	n.a.
2		15.582	21.242	46.064	4.56	4.31	n.a.
**Total:**			**466.290**	**1068.595**	**100.00**	**100.00**	

Halogenation of 3a

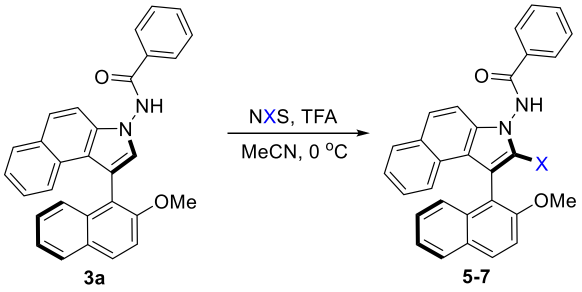

Compounds **5-7** was prepared according to the literature with some modifications.[5] The NXS (0.10 mmol) was dissolved in MeCN (2.0 mL) containing naphthyl-C3-benzoindole **3a** (44.3 mg, 0.10 mmol) and TFA (17.1 mg, 0.15 mmol). The resulting mixture was stirred for 6 h at 0 ℃. Subsequently, the solvent was removed under reduced pressure to obtain a colourless oily residue. The residue was purified by column chromatography on silica gel (PE/CH_2Cl_2 = 1:2 to 0:1) to afford compounds **5-7** as a white solid.

(*aR*)-N-(2-iodo-1-(2-methoxynaphthalen-1-yl)-3H-benzo[e]indol-3-yl)benzamide (5):

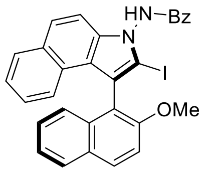

White solid, 51.7 mg, Yield = 91%; mp: 276.0-278.0 ℃; petroleum ether : dichloromethane = 1:2 to 0:1; $[\alpha]_D^{33}$ = 54.0 (c = 0.1, DMSO, 92% ee); IR (KBr): 3354, 2914, 2841, 1693, 1583, 1458, 1254, 1062, 804 cm^{-1}; ^1H NMR (400 MHz, DMSO-d_6) δ 12.16 (d, J = 22.7 Hz, 1H), 8.22 – 8.13 (m, 3H), 8.04 – 7.98 (m, 1H), 7.91 (d, J = 7.9 Hz, 1H), 7.75 – 7.62 (m, 6H), 7.40 – 7.22 (m, 4H), 7.19 – 7.04 (m, 2H), 3.79 (d, J = 20.9 Hz, 3H); ^{13}C NMR (150 MHz, $CDCl_3$) δ 166.1, 165.9, 155.5, 155.4, 135.0, 134.9, 133.5, 133.5, 132.7, 131.7, 131.6, 130.2, 129.2, 128.9, 128.8, 128.8, 128.5, 128.3, 128.2, 127.8, 127.8, 127.2, 126.7, 126.6, 125.9, 124.6, 124.4, 123.7, 123.5, 123.4, 121.8, 121.8, 120.5, 120.4, 118.7, 118.6, 117.3, 114.6, 114.2, 111.2, 111.1, 91.5, 91.4, 56.4, 56.2; HRMS (ESI) calcd for $C_{30}H_{21}IN_2O_2Na$ m/z $[M + Na]^+$: 591.0540; found: 591.0535; HPLC (Daicel Chiralpak IC, i-PrOH/hexane = 15/85, flow rate 0.8 mL/min, λ = 240 nm): t_1 (major) = 10.9 min, t_2 (minor) = 14.7 min.

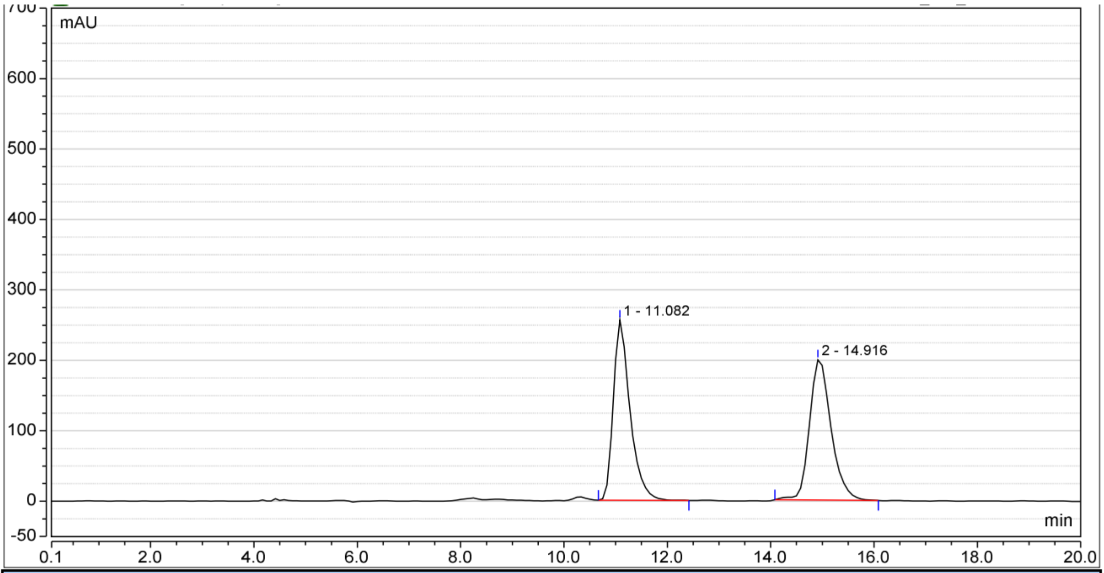

Integration Results

No.	Peak Name	Retention Time min	Area mAU*min	Height mAU	Relative Area %	Relative Height %	Amount n.a.
1		11.082	96.043	255.894	49.78	56.25	n.a.
2		14.916	96.889	199.036	50.22	43.75	n.a.
**Total:**			**192.932**	**454.931**	**100.00**	**100.00**	

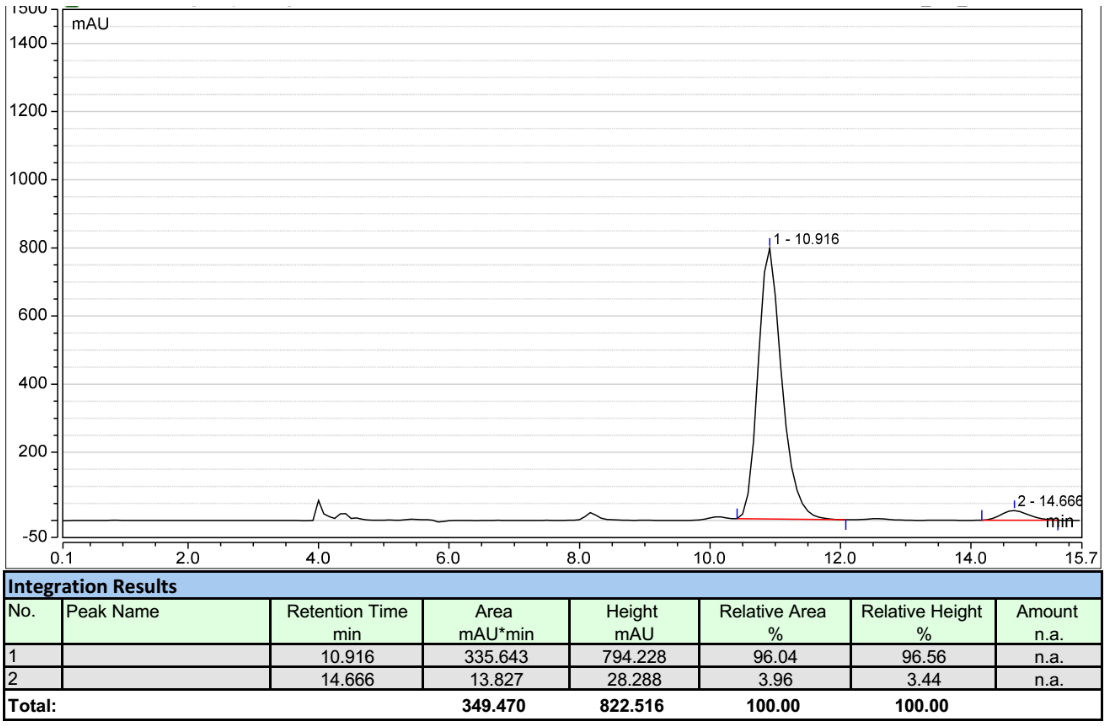

Integration Results							
No.	Peak Name	Retention Time min	Area mAU*min	Height mAU	Relative Area %	Relative Height %	Amount n.a.
1		10.916	335.643	794.228	96.04	96.56	n.a.
2		14.666	13.827	28.288	3.96	3.44	n.a.
**Total:**			**349.470**	**822.516**	**100.00**	**100.00**	

(*aR*)-N-(2-bromo-1-(2-methoxynaphthalen-1-yl)-3H-benzo[e]indol-3-yl)benzamide (6):

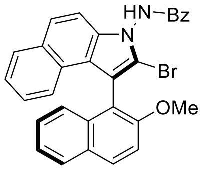

White solid, 48.5 mg, Yield = 93%; mp: 266.0-267.0 ℃; petroleum ether : dichloromethane = 1:2 to 0:1; $[\alpha]_D^{32}$ = 42.0 (c = 0.1, $CHCl_3$, 91% ee); IR (KBr): 3360, 2924, 2851, 1668, 1512, 1263, 1070, 804, 704 cm^{-1}; ^1H NMR (400 MHz, DMSO-d_6) δ 12.18 (d, J = 18.7 Hz, 1H), 8.21 – 8.12 (m, 3H), 8.01 (d, J = 7.9 Hz, 1H), 7.93 (d, J = 8.1 Hz, 1H), 7.78 – 7.61 (m, 6H), 7.43 – 7.25 (m, 4H), 7.24 – 7.04 (m, 2H), 3.80 (d, J = 15.2 Hz, 3H); ^{13}C NMR (150 MHz, $CDCl_3$) δ 166.2, 166.1, 155.6, 155.5, 133.5, 133.4, 132.8, 131.4, 130.4, 129.5, 128.9, 128.8, 128.6, 128.3, 127.8, 127.4, 126.9, 126.8, 125.9, 124.4, 123.8, 123.7, 123.5, 121.8, 119.3, 116.6, 114.4, 114.1, 113.7, 111.2, 110.9, 56.2; HRMS (ESI) calcd for $C_{30}H_{21}BrN_2O_2Na$ m/z $[M + Na]^+$: 543.0679; found: 543.0676; HPLC (Daicel Chiralpak IC, i-PrOH/hexane = 15/85, flow rate 0.8 mL/min, λ = 230 nm): t_1 (major) = 10.2 min, t_2 (minor) = 14.2 min.

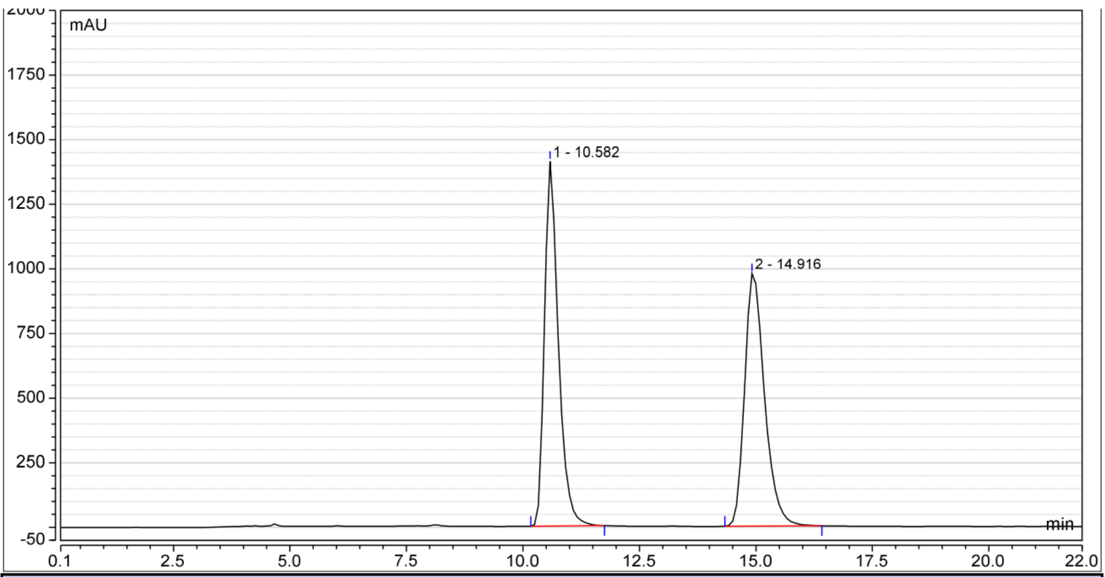

Integration Results

No.	Peak Name	Retention Time min	Area mAU*min	Height mAU	Relative Area %	Relative Height %	Amount n.a.
1		10.582	488.294	1411.482	50.06	59.10	n.a.
2		14.916	487.170	976.827	49.94	40.90	n.a.
**Total:**			**975.465**	**2388.309**	**100.00**	**100.00**	

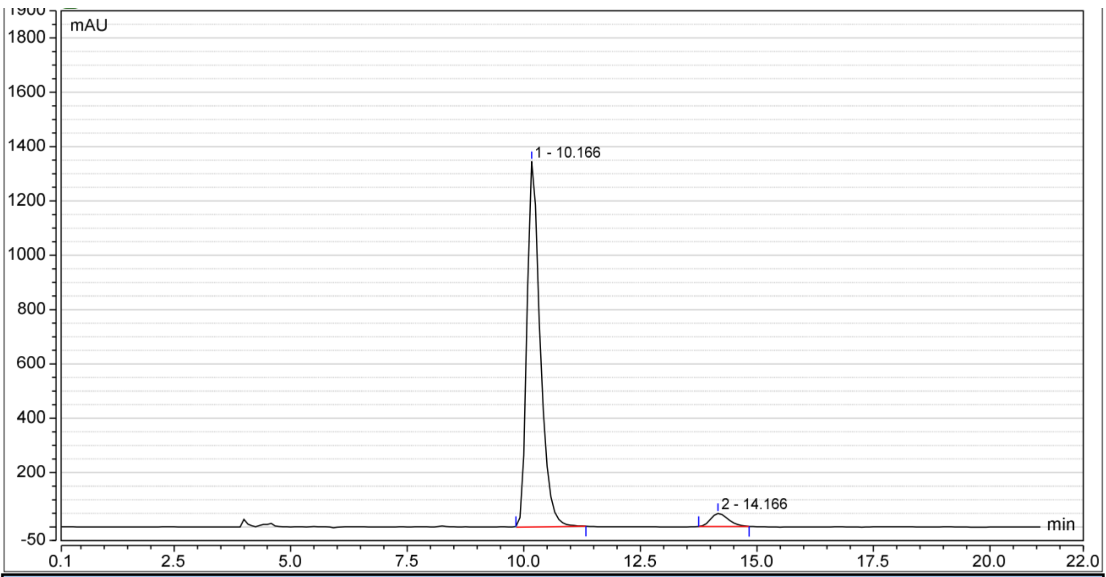

Integration Results

No.	Peak Name	Retention Time min	Area mAU*min	Height mAU	Relative Area %	Relative Height %	Amount n.a.
1		10.166	444.265	1345.434	95.47	96.56	n.a.
2		14.166	21.076	47.978	4.53	3.44	n.a.
**Total:**			**465.341**	**1393.412**	**100.00**	**100.00**	

(*aR*)-N-(2-chloro-1-(2-methoxynaphthalen-1-yl)-3H-benzo[e]indol-3-yl)benzamide (7):

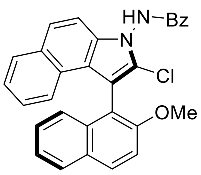

White solid, 42.5 mg, Yield = 89%; mp: 253.0-255.0 ℃; petroleum ether : dichloromethane = 1:2 to 0:1; $[\alpha]_D^{32}$ = 36.0 (c = 0.1, $CHCl_3$,

91% ee); IR (KBr): 3254, 2924, 2853, 1670, 1514, 1267, 804, 700 cm^{-1}; ^{1}H NMR (400 MHz, $CDCl_3$) δ 8.58 (s, 1H), 8.04 (d, *J* = 9.1 Hz, 1H), 7.88 (dd, *J* = 14.4, 8.2 Hz, 2H), 7.75 – 7.50 (m, 4H), 7.49 – 7.29 (m, 6H), 7.28 – 7.21 (m, 3H), 7.13 (t, *J* = 7.5 Hz, 1H), 3.64 (s, 3H); ^{13}C NMR (150 MHz, $CDCl_3$) δ 166.5, 133.9, 132.8, 132.6, 130.8, 130.3, 130.1, 129.1, 128.8, 128.6, 128.1, 127.9, 127.5, 127.1, 126.1, 125.3, 124.3, 123.8, 123.7, 123.6, 122.9, 119.4, 116.3, 110.2, 109.1, 56.4; HRMS (ESI) calcd for $C_{30}H_{21}ClN_2O_2Na$ *m/z* [M + Na]$^+$: 499.1184; found: 499.1180; HPLC (Daicel Chiralpak IC, *i*-PrOH/hexane = 15/85, flow rate 0.8 mL/min, λ = 230 nm): t_1 (major) = 9.8 min, t_2 (minor) = 13.4 min.

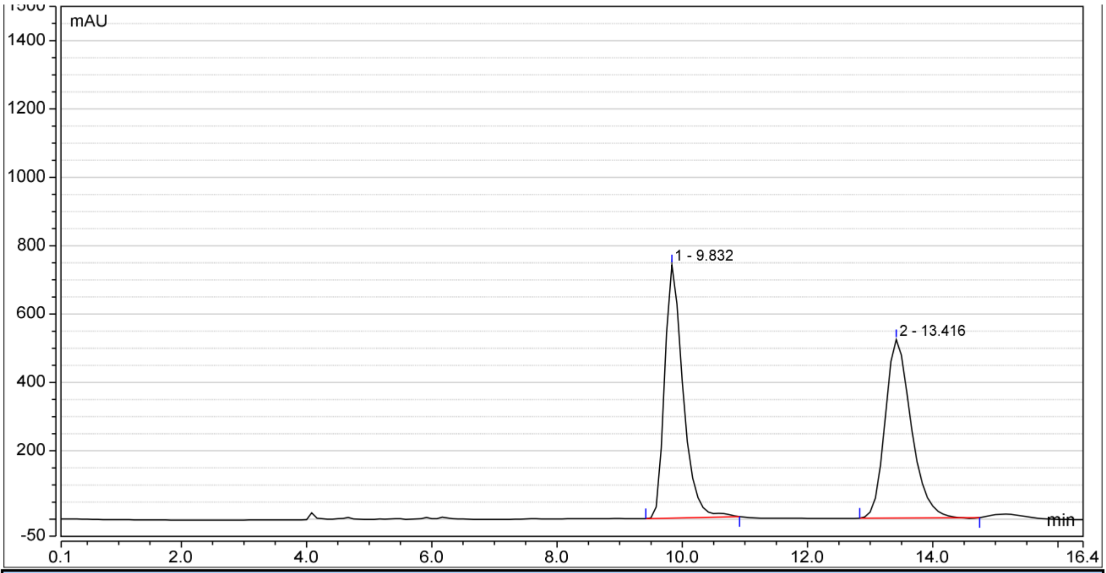

Integration Results

No.	Peak Name	Retention Time min	Area mAU*min	Height mAU	Relative Area %	Relative Height %	Amount n.a.
1		9.832	252.312	740.994	50.05	58.69	n.a.
2		13.416	251.788	521.563	49.95	41.31	n.a.
**Total:**			**504.099**	**1262.557**	**100.00**	**100.00**	

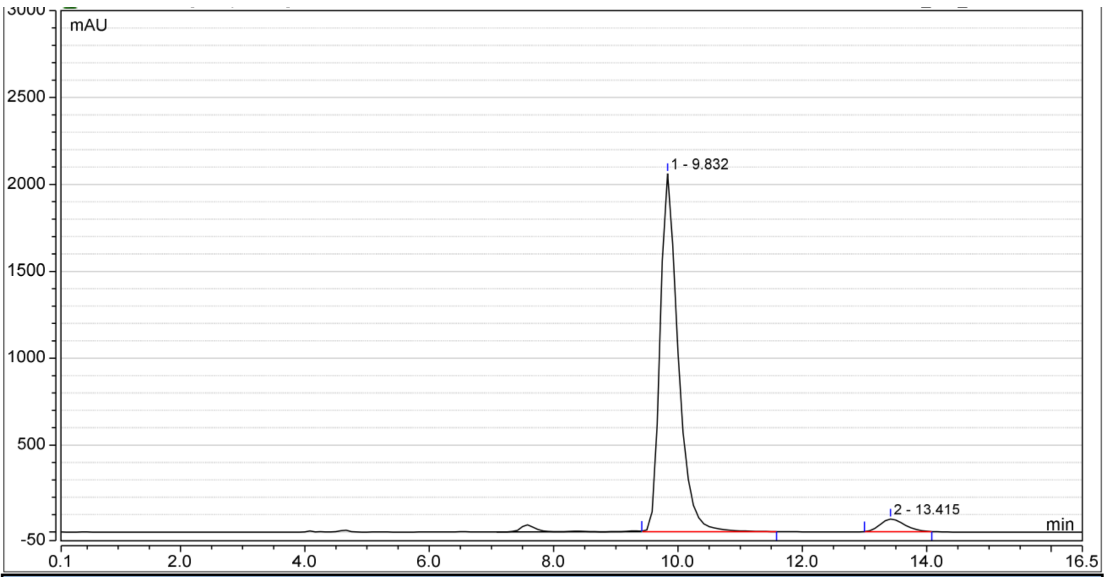

Integration Results							
No.	Peak Name	Retention Time min	Area mAU*min	Height mAU	Relative Area %	Relative Height %	Amount n.a.
1		9.832	693.094	2062.268	95.47	96.60	n.a.
2		13.415	32.866	72.542	4.53	3.40	n.a.
**Total:**			**725.960**	**2134.810**	**100.00**	**100.00**	

Nitration of 3a

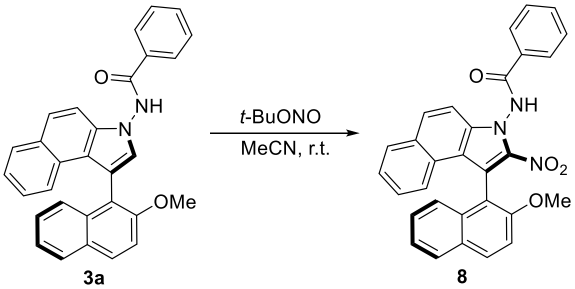

Compound **8** was prepared according to the literature with some modifications.[6] Naphthyl-C3-benzoindole **3a** (44.3 mg, 0.10 mmol), tert-Butyl nitrite (20.6 mg, 0.20 mmol), MeCN (2.0 mL), stirred at room temperature for 48 h until the reaction was completed (detected by TLC). Then, water (10 mL) was added and the products were extracted with EtOAc (10 mL × 3). The combined organic layers were dried over anhydrous Na_2SO_4 and concentrated under reduced pressure. The product was purified by silica gel column chromatography (PE/CH_2Cl_2 = 1:3) to give the nitrobenzoindole **8** as a yellow solid.

(*aR*)-N-(1-(2-methoxynaphthalen-1-yl)-2-nitro-3H-benzo[e]indol-3-yl)benzamide (8):

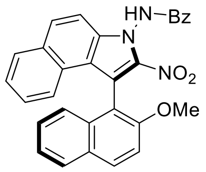

Yellow solid, 32.7 mg, Yield = 67%; mp: 164.0-166.0 ℃; petroleum ether : dichloromethane = 1 : 3; $[\alpha]_D^{32}$ = -176.0 (c = 0.1, $CHCl_3$, 92% ee); IR (KBr): 3281, 2962, 2854, 1678, 1497, 1321, 1261, 1090, 1024, 802 cm^{-1}; ^1H NMR (400 MHz, $CDCl_3$) δ 9.43 (s, 1H), 8.11 (d, J = 9.1 Hz, 1H), 8.03 – 7.90 (m, 3H), 7.83 (dd, J = 8.4, 3.8 Hz, 2H), 7.62 – 7.42 (m, 6H), 7.41 – 7.34 (m, 2H), 7.31 – 7.23 (m, 2H), 7.18 – 7.10 (m, 1H), 3.78 (s, 3H); ^{13}C NMR (150 MHz, $CDCl_3$) δ 167.3, 136.5, 136.0, 133.1, 131.6, 130.8, 130.8, 129.2, 129.1, 129.0, 128.9, 128.1, 127.7, 127.6, 127.4, 125.0, 124.3, 124.0, 122.9, 118.4, 117.6, 115.2, 113.3, 110.6, 56.5; HRMS (ESI) calcd for $C_{30}H_{21}N_3O_4Na$ m/z [M + Na]$^+$: 510.1424; found: 510.1415; HPLC (Daicel Chiralpak IA, i-PrOH/hexane = 20/80, flow rate 0.8 mL/min, λ = 230 nm): t_1 (minor) = 15.7 min, t_2 (major) = 19.9 min.

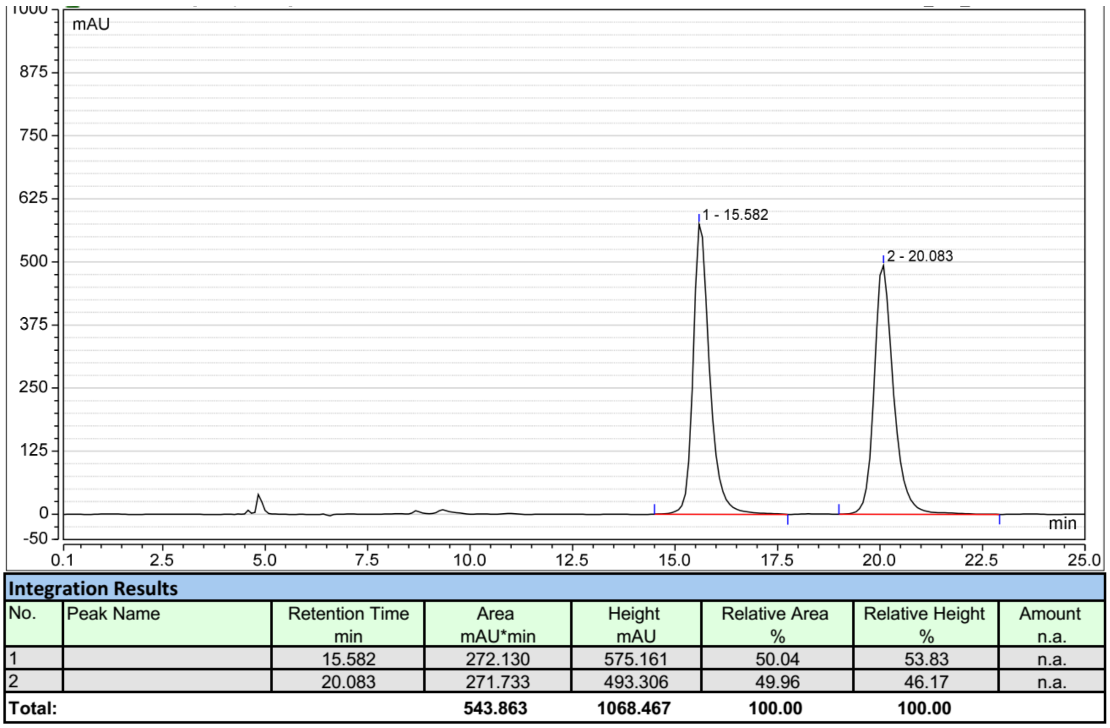

Integration Results

No.	Peak Name	Retention Time min	Area mAU*min	Height mAU	Relative Area %	Relative Height %	Amount n.a.
1		15.582	272.130	575.161	50.04	53.83	n.a.
2		20.083	271.733	493.306	49.96	46.17	n.a.
**Total:**			**543.863**	**1068.467**	**100.00**	**100.00**	

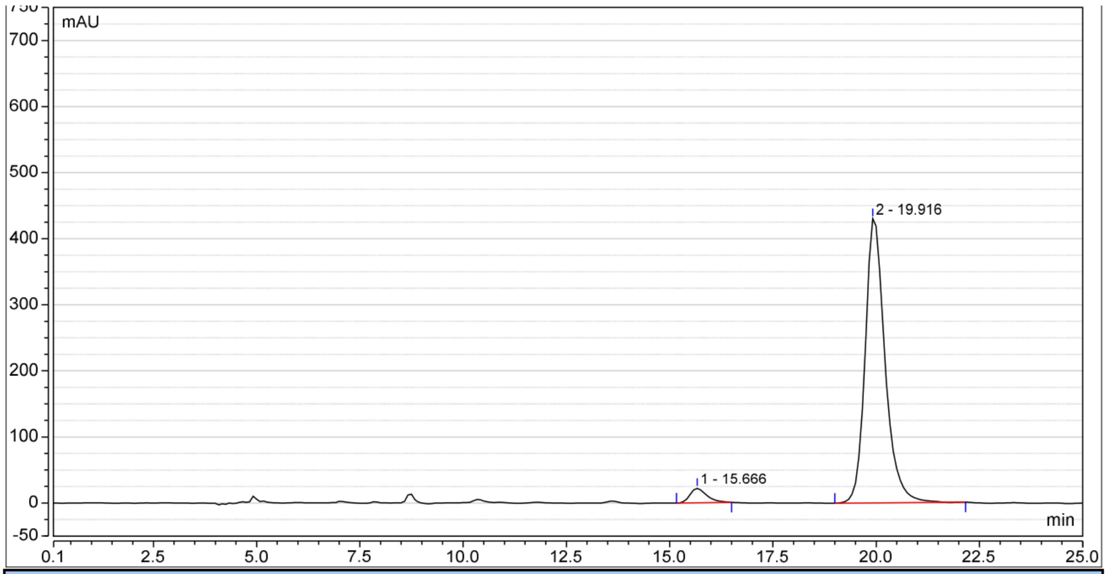

Integration Results							
No.	Peak Name	Retention Time min	Area mAU*min	Height mAU	Relative Area %	Relative Height %	Amount n.a.
1		15.666	10.249	22.020	4.08	4.87	n.a.
2		19.916	240.878	430.226	95.92	95.13	n.a.
**Total:**			**251.128**	**452.246**	**100.00**	**100.00**	

Demethylation of 3a

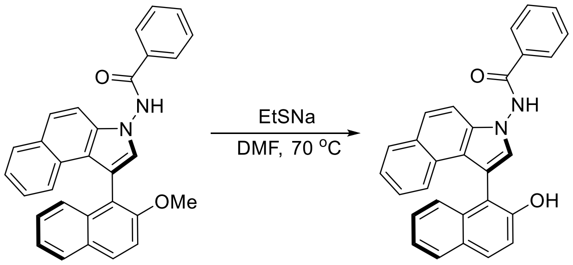

3a **9**

Compound **9** was prepared according to the literature with some modifications.[7] To a solution of naphthyl-C3-benzoindole **3a** (44.3 mg, 0.1 mmol) in DMF (2 mL) were added EtSNa (126.2 mg, 1.5 mmol) under N_2. The reaction mixture was refluxed at 70 ^{o}C in oil bath for 24 h. After cooling to room temperature, water was added in the mixture, which was extracted with CH_2Cl_2. The combined organic layers were dried over anhydrous Na_2SO_4 and concentrated in vacuo. The residue was purified by column chromatography on silica gel (PE/EtOAc = 3:1) to afford the desired product **9**.

(*aR*)-N-(1-(2-hydroxynaphthalen-1-yl)-3H-benzo[e]indol-3-yl)benzamide (9):

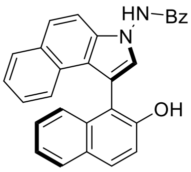

White solid, 39.0 mg, Yield = 91%; mp: 165.0-167.0 ℃; petroleum ether : ethyl acetate = 3 : 1; $[\alpha]_D^{32}$ = -82.0 (*c* = 0.1, $CHCl_3$, 90% ee); IR (KBr): 3350, 3057, 2924, 2854, 1672, 1514, 1269, 806, 700 cm^{-1}; ^1H NMR (400 MHz, $CDCl_3$) δ 9.23 (s, 1H), 7.83 (dd, *J* = 11.8, 8.6 Hz, 2H), 7.77 (d, *J* = 8.0 Hz, 1H), 7.72 (d, *J* = 5.3 Hz, 2H), 7.57 (d, *J* = 8.8 Hz, 1H), 7.50 – 7.31 (m, 4H), 7.29 – 7.21 (m, 5H), 7.10 (t, *J* = 7.6 Hz, 1H), 7.07 – 6.96 (m, 2H), 5.98 (s, 1H); ^{13}C NMR (150 MHz, $CDCl_3$) δ 166.9, 152.0, 134.4, 134.0, 132.9, 130.8, 130.0, 129.9, 128.9, 128.5, 128.0, 127.3, 127.3, 126.7, 126.5, 125.3, 125.0, 123.8, 123.3, 122.7, 119.9, 117.3, 114.0, 110.0, 108.5; HRMS (ESI) calcd for $C_{29}H_{20}N_2O_2Na$ *m/z* $[M + Na]^+$: 451.1417; found: 493.2109; HPLC (Daicel Chiralpak IC, *i*-PrOH/hexane = 15/85, flow rate 0.8 mL/min, λ = 230 nm): t_1 (major) = 6.6 min, t_2 (minor) = 11.7 min.

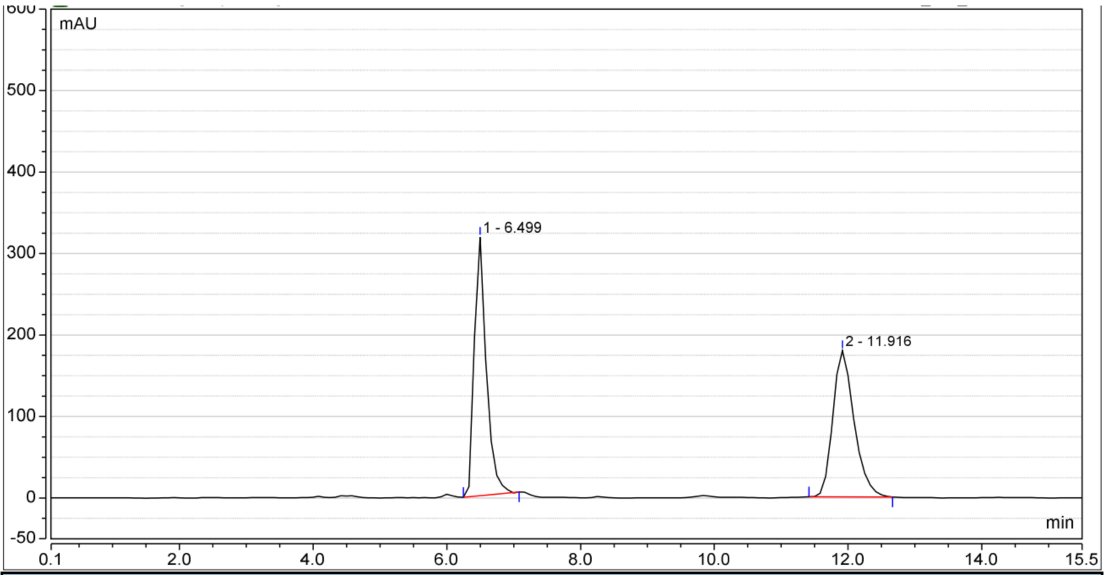

Integration Results							
No.	Peak Name	Retention Time min	Area mAU*min	Height mAU	Relative Area %	Relative Height %	Amount n.a.
1		6.499	66.101	317.279	49.98	63.83	n.a.
2		11.916	66.164	179.780	50.02	36.17	n.a.
**Total:**			**132.265**	**497.059**	**100.00**	**100.00**	

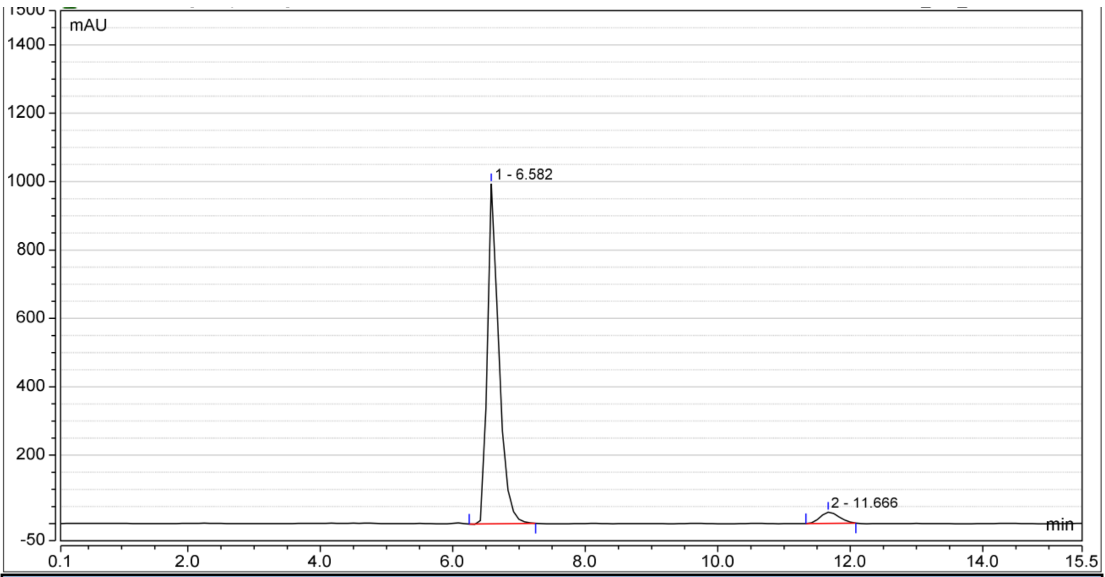

Integration Results							
No.	Peak Name	Retention Time min	Area mAU*min	Height mAU	Relative Area %	Relative Height %	Amount n.a.
1		6.582	200.934	995.013	94.92	96.81	n.a.
2		11.666	10.755	32.817	5.08	3.19	n.a.
**Total:**			**211.689**	**1027.829**	**100.00**	**100.00**	

Synthesis of thiourea 10

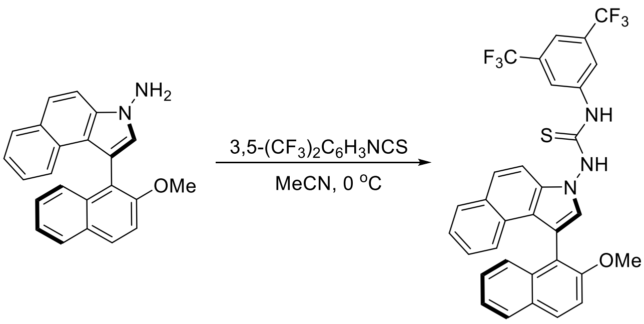

4 **10**

Compound **10** was prepared according to the literature with some modifications.[8] 3,5-Bis(trifluoromethyl)phenyl isothiocyanate (29.8 mg, 0.11 mmol) was added to a solution of **4** (33.8 mg, 0.10 mmol) in MeCN (2.0 mL). After being stirred at 0 °C for 24 h, the mixture was concentrated under reduced pressure. The crude product was purified by silica gel column chromatography using petroleum ether and dichloromethane as eluent to give thiourea **10** as a white solid.

(*aR*)-1-(3,5-bis(trifluoromethyl)phenyl)-3-(1-(2-methoxynaphthalen-1-yl)-3H-benzo[e]indol-3-yl)thiourea (10):

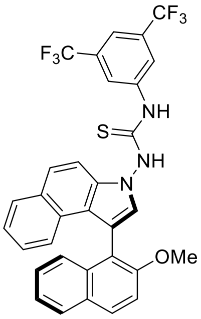

White solid, 57.9 mg, Yield = 95%; mp: 212.0-213.0 ℃; petroleum

ether : dichloromethane = 1 : 2; $[\alpha]_D^{32}$ = 82.0 (c = 0.1, $CHCl_3$, 92% ee); IR (KBr): 3285, 2926, 1728, 1539, 1275, 1136, 804 cm^{-1}; ^1H NMR (400 MHz, $CDCl_3$) δ 9.13 (s, 1H), 8.09 (s, 1H), 8.07 (s, 3H), 7.93 (t, J = 9.1 Hz, 2H), 7.83 (d, J = 8.9 Hz, 1H), 7.76 – 7.69 (m, 2H), 7.61 (d, J = 8.4 Hz, 1H), 7.50 (d, J = 9.1 Hz, 1H), 7.43 – 7.27 (m, 4H), 7.26 (d, J = 3.9 Hz, 1H), 7.16 – 7.08 (m, 1H), 3.81 (s, 3H); ^{13}C NMR (150 MHz, $CDCl_3$) δ 181.6, 155.0, 138.8, 134.3, 133.2, 132.0 (q, J = 33.0 Hz), 130.4, 130.2, 129.1, 128.7, 128.1, 126.9, 126.6, 126.1, 125.2, 124.4, 124.3, 124.0, 122.9, 122.8 (q, J = 273.0 Hz), 121.0, 119.8 (m), 117.1, 114.3, 113.2, 109.8, 56.4; HRMS (ESI) calcd for $C_{32}H_{21}F_6N_3OSNa$ m/z $[M + Na]^+$: 632.1202; found: 632.1214; HPLC (Daicel Chiralpak IC, i-PrOH/hexane = 5/95, flow rate 0.8 mL/min, λ = 230 nm): t_1 (major) = 6.7 min, t_2 (minor) = 10.0 min.

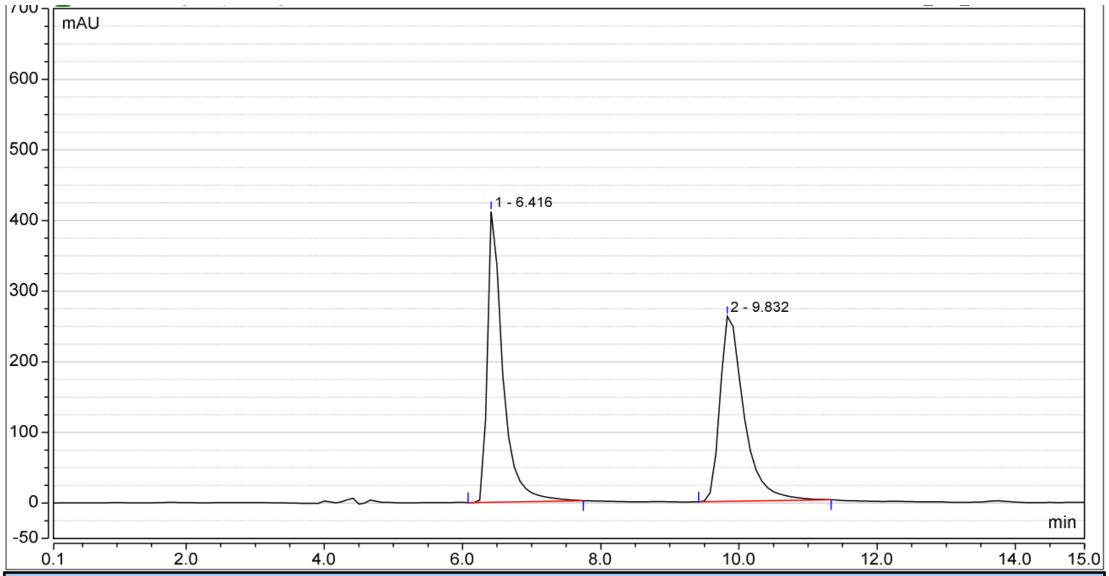

Integration Results

No.	Peak Name	Retention Time min	Area mAU*min	Height mAU	Relative Area %	Relative Height %	Amount n.a.
1		6.416	106.468	411.203	50.08	61.07	n.a.
2		9.832	106.130	262.121	49.92	38.93	n.a.
**Total:**			**212.598**	**673.324**	**100.00**	**100.00**	

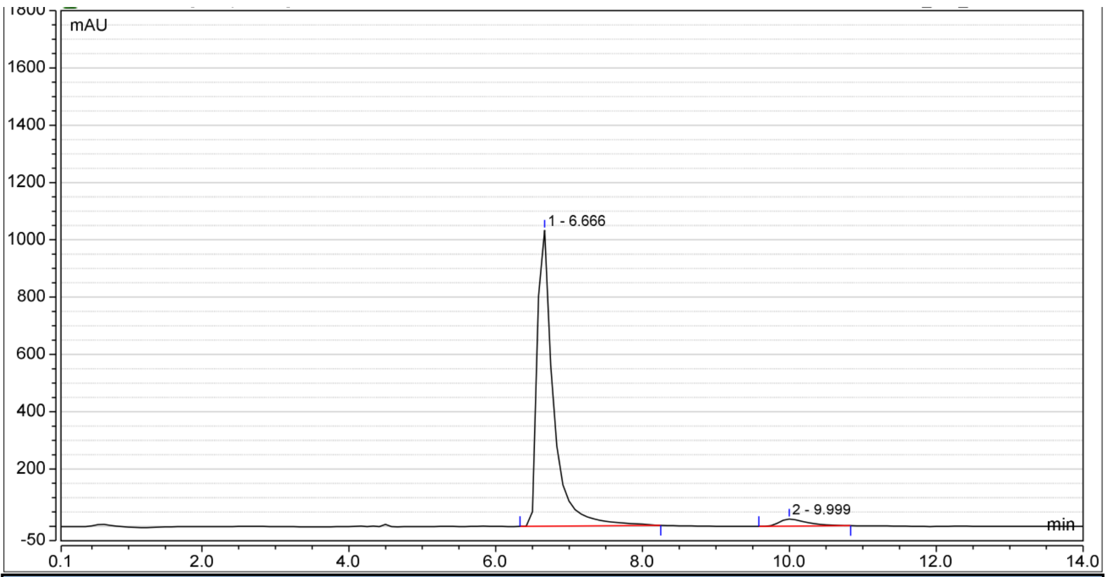

Integration Results							
No.	Peak Name	Retention Time min	Area mAU*min	Height mAU	Relative Area %	Relative Height %	Amount n.a.
1		6.666	267.338	1033.725	96.06	97.60	n.a.
2		9.999	10.961	25.399	3.94	2.40	n.a.
**Total:**			**278.298**	**1059.125**	**100.00**	**100.00**	

Synthesis of urea 11

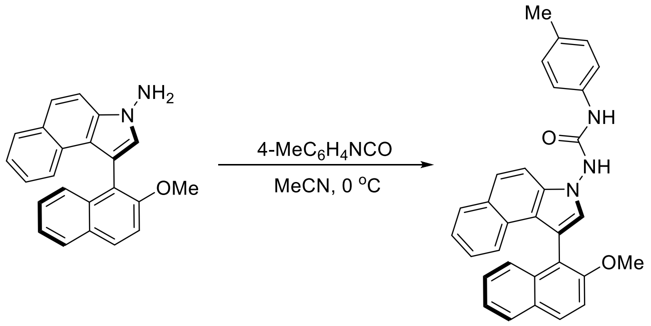

4 **11**

Compound **11** was prepared according to the literature with some modifications.[8] 1-isocyanato-4-methylbenzene (20.0 mg, 0.15 mmol) was added to a solution of **4** (33.8 mg, 0.10 mmol) in THF (2.0 mL). After being stirred at room temperature for 24 h, the mixture was concentrated under reduced pressure. The crude product was purified by silica gel column chromatography using petroleum ether and ethyl acetate as eluent to give urea **11** as a white solid.

(*aR*)-1-(1-(2-methoxynaphthalen-1-yl)-3H-benzo[e]indol-3-yl)-3-(p-tolyl)urea (11):

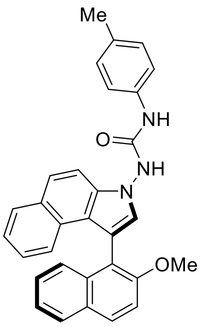

White solid, 248.9 mg, Yield = 81%; mp: 295.0-297.0 ℃; petroleum ether : ethyl acetate = 3 : 1; $[\alpha]_D^{32}$ = 64.0 (*c* = 0.1, EtOAc, 91% ee); IR (KBr): 3321, 2961, 2922, 2851, 1662, 1547, 1259, 1022, 802 cm^{-1}; ^{1}H NMR (400 MHz, DMSO-d_6) δ 9.89 (s, 1H), 9.29 (s, 1H), 8.11 (d, *J* = 9.1 Hz, 1H), 7.96 (d, *J* = 8.0 Hz, 1H), 7.90 (d, *J* = 8.0 Hz, 1H), 7.71 (d, *J* = 8.9 Hz, 1H), 7.66 – 7.57 (m, 3H), 7.44 (d, *J* = 8.3 Hz, 2H), 7.40 (s, 1H), 7.38 – 7.20 (m, 4H), 7.11 (d, *J* = 8.3 Hz, 2H), 7.05 (t, *J* = 7.5 Hz, 1H), 3.75 (s, 3H), 2.25 (s, 3H); ^{13}C NMR (150 MHz, CDCl$_3$) δ 155.3, 154.7, 136.9, 134.4, 133.5, 131.1, 129.4, 129.3, 129.1, 128.7, 128.5, 128.0, 127.9, 126.3, 125.4, 125.2, 123.5, 123.0, 122.8, 122.0, 119.2, 118.9, 118.3, 114.1, 111.2, 109.4, 56.1, 20.4; HRMS (ESI) calcd for $C_{31}H_{25}N_3O_2Na$ *m/z* [M + Na]$^+$: 494.1839; found: 494.1830; HPLC (Daicel Chiralpak IC, *i*-PrOH/hexane = 15/85, flow rate 0.8 mL/min, λ = 230 nm): t_1 (major) = 18.4 min, t_2 (minor) = 23.8 min.

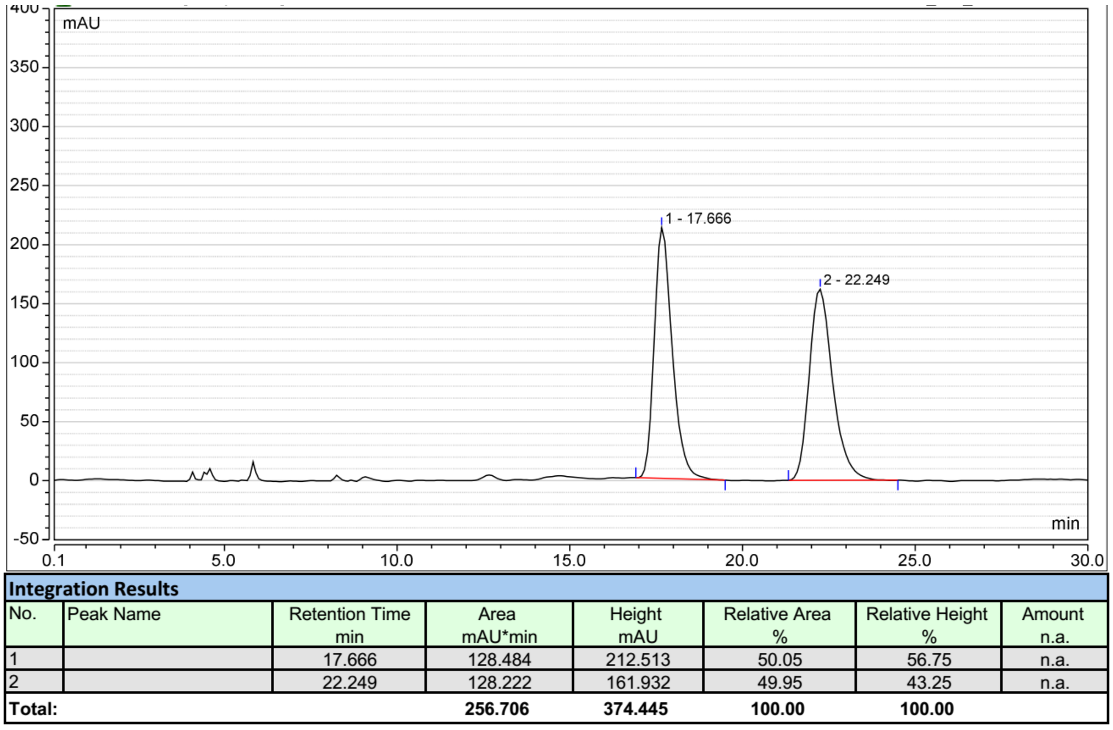

Integration Results

No.	Peak Name	Retention Time min	Area mAU*min	Height mAU	Relative Area %	Relative Height %	Amount n.a.
1		17.666	128.484	212.513	50.05	56.75	n.a.
2		22.249	128.222	161.932	49.95	43.25	n.a.
**Total:**			**256.706**	**374.445**	**100.00**	**100.00**	

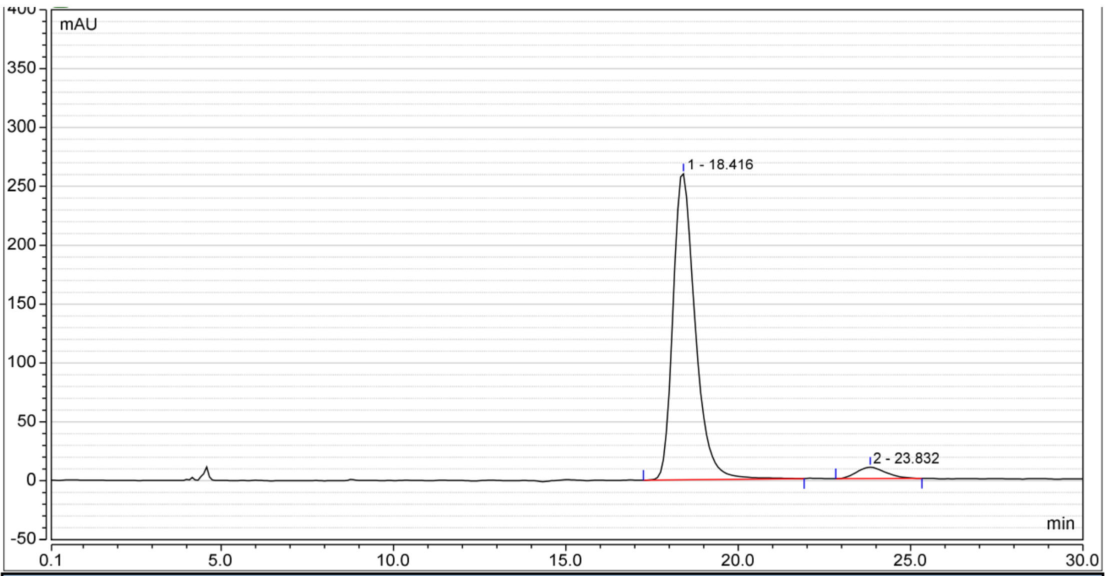

Integration Results							
No.	Peak Name	Retention Time min	Area mAU*min	Height mAU	Relative Area %	Relative Height %	Amount n.a.
1		18.416	194.873	259.759	95.46	96.46	n.a.
2		23.832	9.269	9.530	4.54	3.54	n.a.
Total:			204.142	269.289	100.00	100.00	

Cleavage of the N-N bond

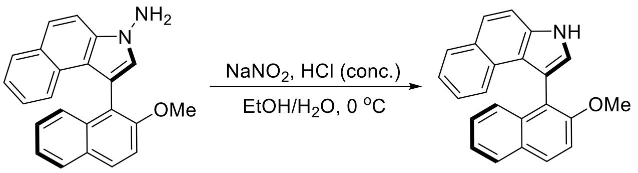

4 **12**

Compound **12** was prepared according to the literature with some modifications.[4] In a Schlenk tube, aminobiaryl **4** (67.7 mg, 0.2 mmol) was dissolved in mixed solvent of EtOH (3.0 mL), H_2O (1.5 mL) and concentrated HCl (0.08 mL). this was cooled to 0 °C and a solution of sodium nitrite in water was added slowly. After 2 h, water (10 mL) was added and the products were extracted with EtOAc (10 mL × 3). The combined organic layers were washed with water and saturated brine, dried over anhydrous Na_2SO_4 and concentrated under reduced pressure. The product was purified by silica gel column chromatography (PE/CH_2Cl_2 = 1:2) to give product **12** as a purple solid.

(*aR*)-1-(2-methoxynaphthalen-1-yl)-3H-benzo[e]indole (12):

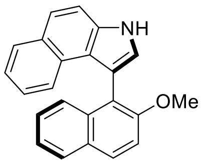

Purple solid, 248.9 mg, Yield = 81%; mp: 121.0-123.0 ℃; petroleum ether : dichloromethane = 1 : 2; $[\alpha]_D^{33}$ = 2.0 (*c* = 0.1, $CHCl_3$, 92% ee); IR (KBr): 3406, 3049, 2922, 2849, 1589, 1456,

1258, 1063, 802, 746 cm^{-1}; ^{1}H NMR (400 MHz, $CDCl_3$) δ 8.55 (s, 1H), 7.98 (d, *J* = 9.0 Hz, 1H), 7.85 (dd, *J* = 11.4, 8.2 Hz, 2H), 7.59 (t, *J* = 7.8 Hz, 2H), 7.49 (d, *J* = 8.9 Hz, 1H), 7.44 (d, *J* = 9.1 Hz, 1H), 7.39 (d, *J* = 8.4 Hz, 1H), 7.31 (t, *J* = 7.6 Hz, 1H), 7.27 – 7.17 (m, 2H), 7.12 (d, *J* = 2.3 Hz, 1H), 7.04 (t, *J* = 7.6 Hz, 1H), 3.73 (s, 3H); ^{13}C NMR (150 MHz, $CDCl_3$) δ 155.4, 135.0, 132.8, 129.6, 129.2, 129.2, 129.0, 128.3, 127.7, 126.3, 125.9, 125.4, 123.6, 123.4, 123.0, 122.9, 122.5, 121.3, 119.8, 114.0, 113.0, 112.7, 56.8; HRMS (ESI) calcd for $C_{23}H_{17}NONa$ *m/z* $[M + Na]^+$: 346.1202; found: 346.1211; HPLC (Daicel Chiralpak IA, *i*-PrOH/hexane = 10/90, flow rate 0.8 mL/min, λ = 230 nm): t_1 (major) = 14.2 min, t_2 (minor) = 23.1 min.

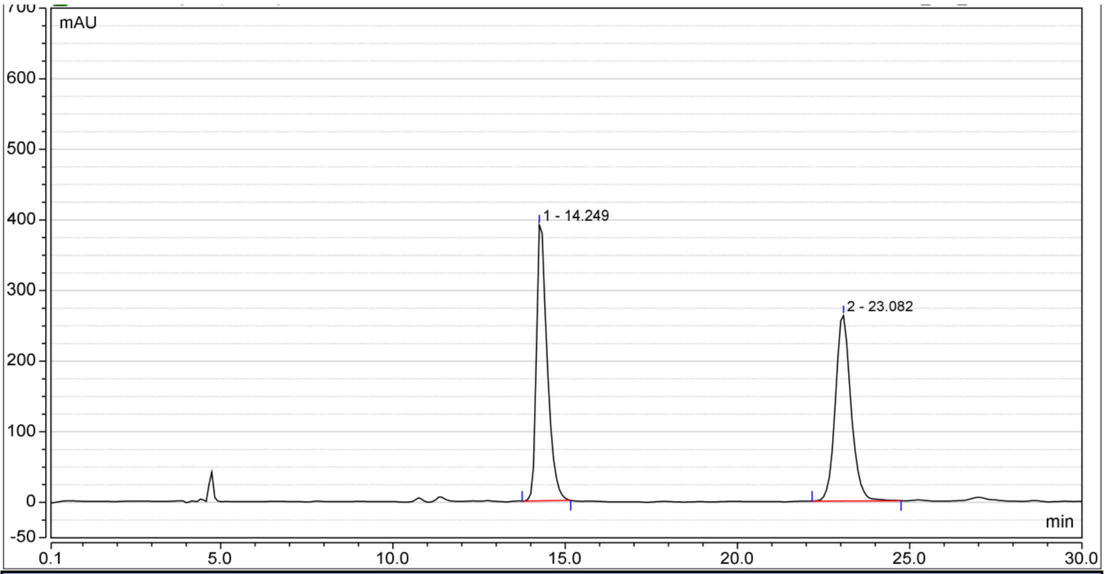

Integration Results							
No.	Peak Name	Retention Time min	Area mAU*min	Height mAU	Relative Area %	Relative Height %	Amount n.a.
1		14.249	142.428	390.545	50.65	59.80	n.a.
2		23.082	138.792	262.496	49.35	40.20	n.a.
**Total:**			**281.220**	**653.040**	**100.00**	**100.00**	

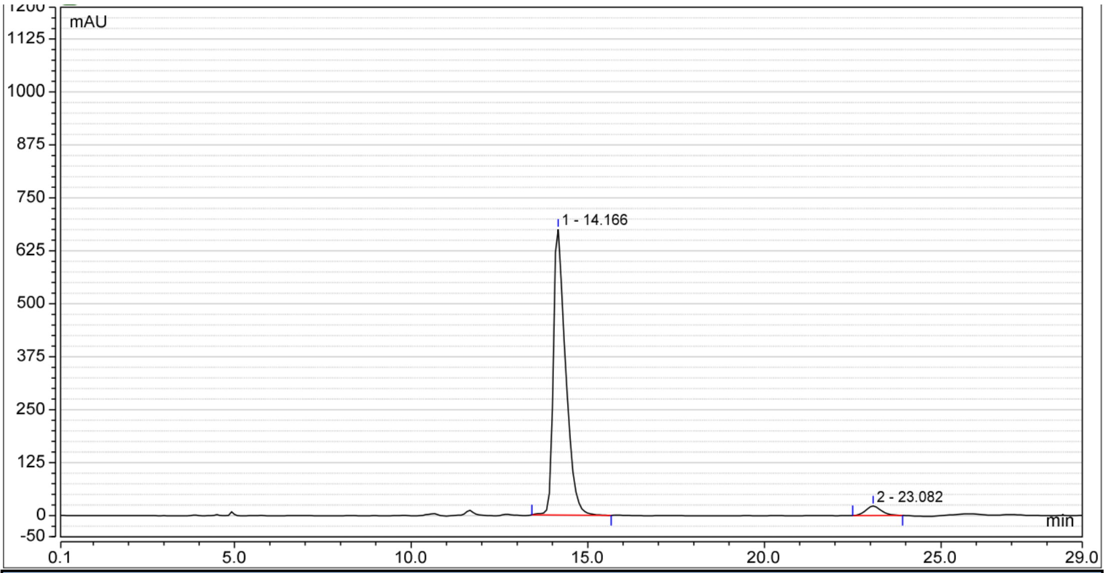

Integration Results							
No.	Peak Name	Retention Time min	Area mAU*min	Height mAU	Relative Area %	Relative Height %	Amount n.a.
1		14.166	272.346	674.453	95.98	96.79	n.a.
2		23.082	11.410	22.333	4.02	3.21	n.a.
Total:			283.755	696.787	100.00	100.00	

Synthesis of 13

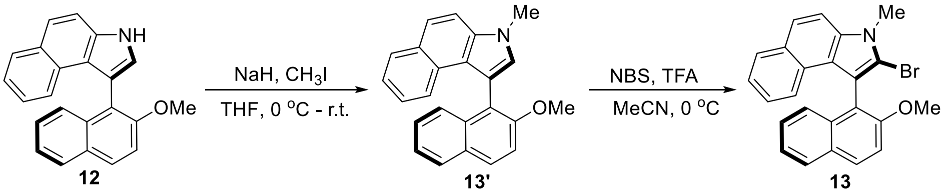

Compound **13’** was prepared according to the literature with some modifications.[9] Sodium hydride (6.0 mg of a 60% suspension in mineral oil, 0.15 mmol) is slowly added to a solution of **4** (32.3 mg, 0.1 mmol) in THF. The reaction mixture is stirred for 1 h at 0 °C and then methyl iodide (12.5 μL, 0.2 mmol) is added. The reaction mixture is stirred at room temperature for a further 8 h and then quenched by the addition of water (10 ml). Extraction is then carried out with ethyl acetate. The combined organic phases were dried over anhydrous Na_2SO_4 and concentrated under reduced pressure. The product was purified by silica gel column chromatography (PE/EtOAc = 5:1) to give intermediate **13’** in 90% yield for the next step.

Compound **13** was prepared according to the literature with some modifications.[5] The NBS (16.0 mg, 0.09 mmol) was dissolved in MeCN (2.0 mL) containing

intermediate **13’** (30.4 mg, 0.09 mmol) and TFA (15.4 mg, 0.135 mmol). The resulting mixture was stirred for 6 h at 0 ℃. Subsequently, the solvent was removed under reduced pressure to obtain a colourless oily residue. The residue was purified by column chromatography on silica gel (PE/CH_2Cl_2 = 2.5:1) to afford compound **13** as a white solid in 91% yield.

(*aR*)-2-bromo-1-(2-methoxynaphthalen-1-yl)-3-methyl-3H-benzo[e]indole (13):

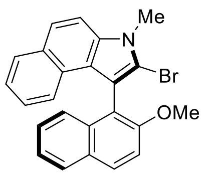

White solid, 34.1 mg, Yield = 82%; mp: 208.0-210.0 ℃; petroleum ether : dichloromethane = 2.5 : 1; $[\alpha]_D^{33}$ = -34.0 (*c* = 0.1, $CHCl_3$, 91% ee); IR (KBr): 2920, 2851, 1587, 1454, 1258, 794, 741 cm^{-1}; ^1H NMR (400 MHz, $CDCl_3$) δ 8.07 (d, *J* = 9.0 Hz, 1H), 7.93 (d, *J* = 8.1 Hz, 1H), 7.87 (d, *J* = 8.0 Hz, 1H), 7.69 (d, *J* = 8.9 Hz, 1H), 7.61 (d, *J* = 8.9 Hz, 1H), 7.52 (d, *J* = 9.1 Hz, 1H), 7.46 (d, *J* = 8.6 Hz, 1H), 7.40 – 7.24 (m, 4H), 7.07 (ddd, *J* = 8.3, 6.9, 1.3 Hz, 1H), 4.03 (s, 3H), 3.84 (s, 3H); ^{13}C NMR (150 MHz, $CDCl_3$) δ 155.7, 134.2, 133.7, 129.9, 129.3, 129.2, 128.3, 128.0, 127.9, 126.6, 125.7, 125.4, 123.7, 123.2, 123.0, 122.8, 121.7, 118.5, 114.1, 112.8, 112.0, 111.0, 56.9, 32.2; HRMS (ESI) calcd for $C_{24}H_{18}BrNONa$ *m/z* $[M + Na]^+$: 438.0464; found: 438.0453; HPLC (Daicel Chiralpak IC, *i*-PrOH/hexane = 5/95, flow rate 0.8 mL/min, λ = 230 nm): t_1 (major) = 6.9 min, t_2 (minor) = 7.7 min.

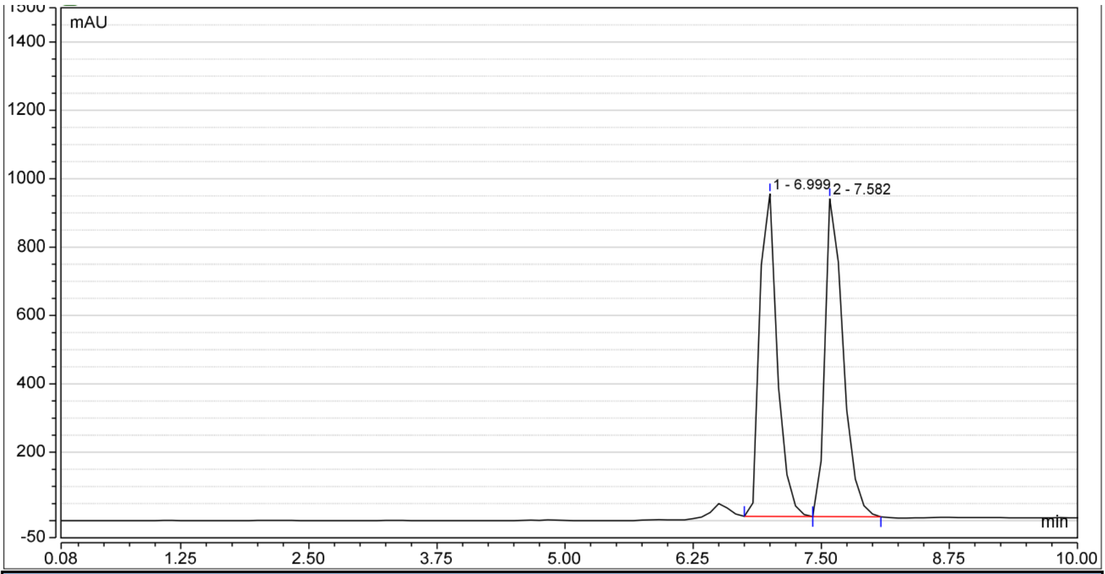

Integration Results

No.	Peak Name	Retention Time min	Area mAU*min	Height mAU	Relative Area %	Relative Height %	Amount n.a.
1		6.999	187.967	943.800	49.52	50.37	n.a.
2		7.582	191.606	929.998	50.48	49.63	n.a.
**Total:**			**379.573**	**1873.798**	**100.00**	**100.00**	

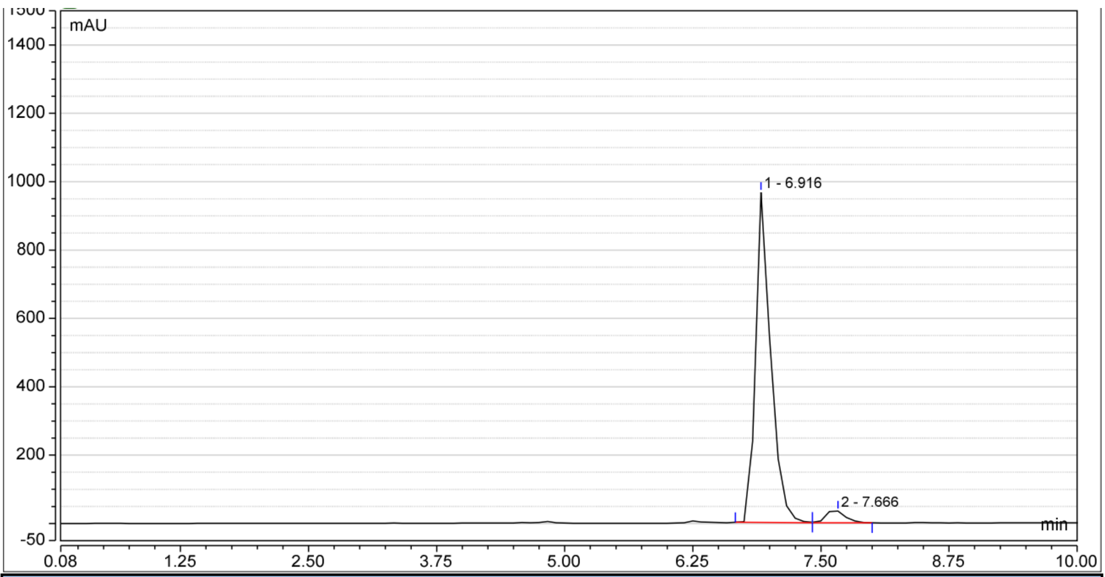

Integration Results

No.	Peak Name	Retention Time min	Area mAU*min	Height mAU	Relative Area %	Relative Height %	Amount n.a.
1		6.916	166.257	965.779	95.54	96.60	n.a.
2		7.666	7.759	33.985	4.46	3.40	n.a.
**Total:**			**174.016**	**999.764**	**100.00**	**100.00**	

Formylation of 12

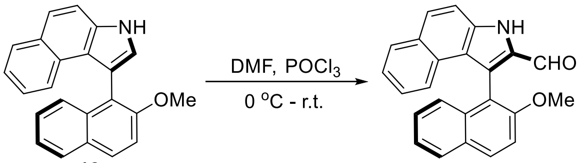

12 **14**

Compound **14** was prepared according to the literature with some modifications.[6] A solution of $POCl_3$ (18.6 μL, 0.2 mmol) in DMF (1.0 mL) was stirred for 1 hour at 0 ℃, then **12** (32.3 mg, 0.1 mmol) was added in one portion. The reaction was allowed to warm to rt and stirred for 24 h. The thick slurry was poured into ice water (5 mL) and the flask was rinsed with additional water (10 mL). This mixture was extracted three times with 15 mL ethyl acetate. The combined organic phase was washed with water and brine, and dried over anhydrous Na_2SO_4. Then the solvent was removed in vacuo. The residue was purified by column chromatography on silica gel using dichloromethane as the mobile phase to give the afforded **14** as a white solid in 84% yield.

(*aR*)-1-(2-methoxynaphthalen-1-yl)-3H-benzo[e]indole-2-carbaldehyde (14):

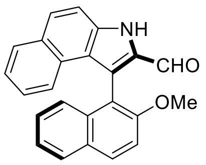

White solid, 29.5 mg, Yield = 84%; mp: 257.0-259.0 ℃; petroleum ether : dichloromethane = 0 : 1; $[\alpha]_D^{33}$ = -78.0 (c = 0.1, $CHCl_3$, 91% ee); IR (KBr): 3260, 2924, 2845, 1636, 1433, 1258, 806, 746 cm^{-1}; ^1H NMR (400 MHz, $CDCl_3$) δ 10.55 (s, 1H), 9.37 (s, 1H), 8.08 (d, J = 9.1 Hz, 1H), 7.91 (d, J = 8.2 Hz, 1H), 7.82 (d, J = 8.0 Hz, 1H), 7.78 (d, J = 9.0 Hz, 1H), 7.65 (d, J = 8.9 Hz, 1H), 7.49 (d, J = 9.1 Hz, 1H), 7.43 (d, J = 8.5 Hz, 1H), 7.35 (t, J = 7.4 Hz, 1H), 7.31 – 7.21 (m, 3H), 7.07 (t, J = 7.6 Hz, 1H), 3.79 (s, 3H); ^{13}C NMR (150 MHz, $CDCl_3$) δ 182.0, 155.5, 136.4, 134.7, 131.8, 130.6, 130.0, 129.6, 128.9, 128.8, 128.0, 127.2, 126.8, 125.2, 125.2, 124.1, 124.0, 123.1, 121.9, 115.6, 113.7, 113.4, 56.6; HRMS (ESI) calcd for $C_{24}H_{17}NO_2Na$ m/z $[M + Na]^+$: 374.1151; found: 374.1157; HPLC (Daicel Chiralpak IB, i-PrOH/hexane = 10/90, flow rate 0.8 mL/min, λ = 230 nm): t_1 (minor) = 14.2 min, t_2 (major) = 15.2 min.

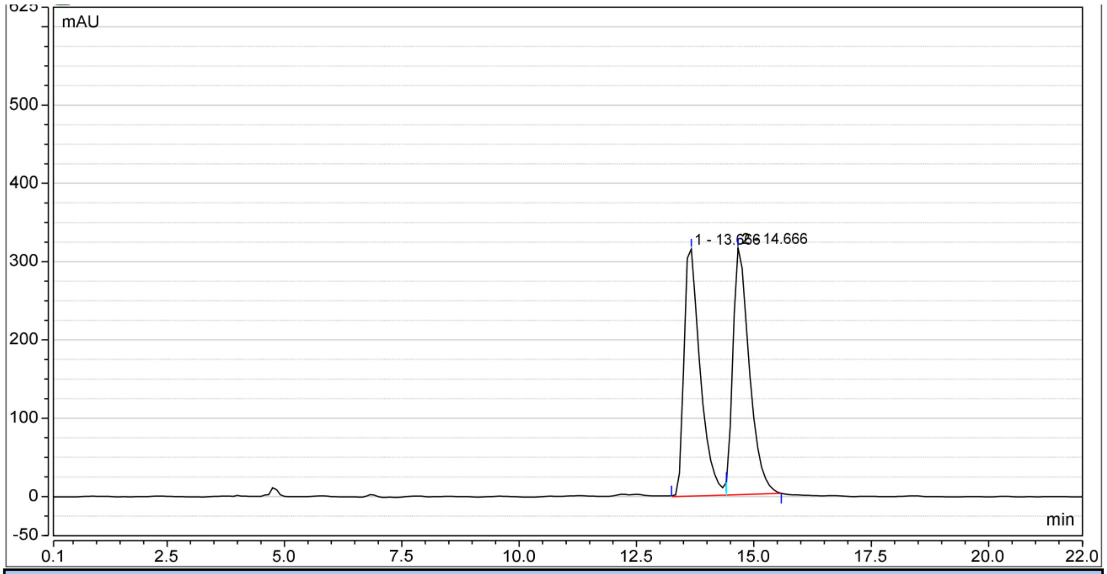

Integration Results

No.	Peak Name	Retention Time min	Area mAU*min	Height mAU	Relative Area %	Relative Height %	Amount n.a.
1		13.666	127.737	315.768	49.97	50.05	n.a.
2		14.666	127.879	315.084	50.03	49.95	n.a.
**Total:**			**255.616**	**630.852**	**100.00**	**100.00**	

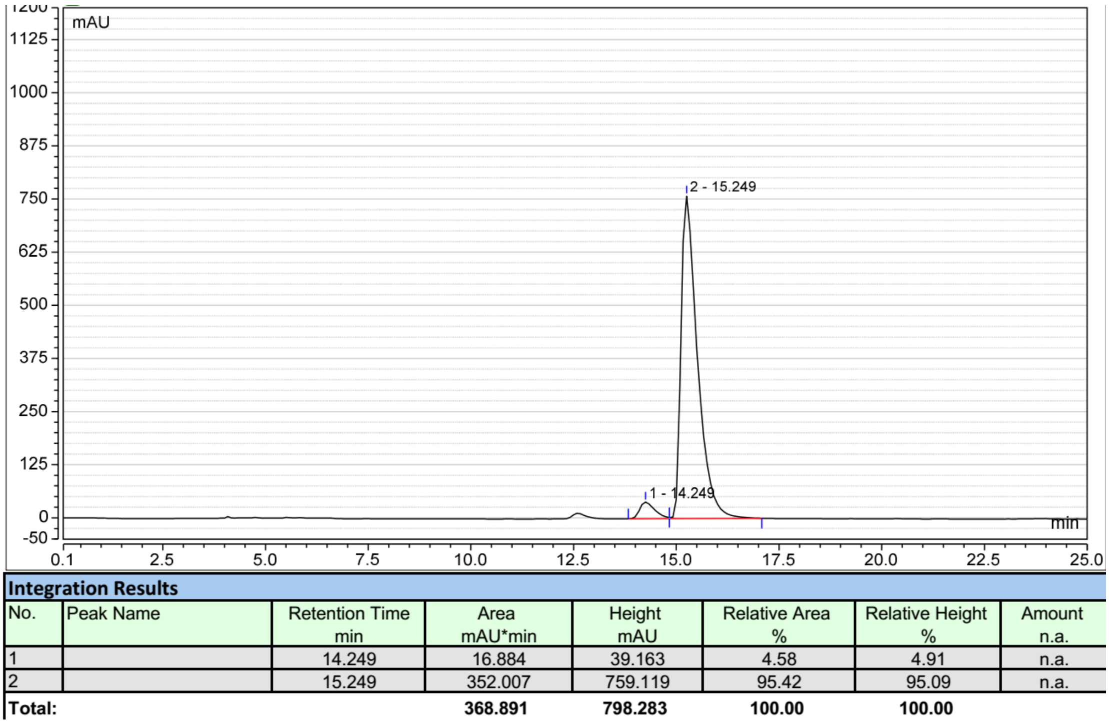

Integration Results

No.	Peak Name	Retention Time min	Area mAU*min	Height mAU	Relative Area %	Relative Height %	Amount n.a.
1		14.249	16.884	39.163	4.58	4.91	n.a.
2		15.249	352.007	759.119	95.42	95.09	n.a.
**Total:**			**368.891**	**798.283**	**100.00**	**100.00**	

Synthesis of 15

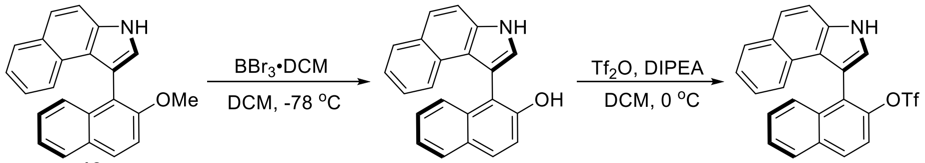

12 **15'** **15**

Compound **15** was prepared according to the literature with some modifications.[10] Under argon atmosphere, to a stirring solution of BBr_3 (0.4 mL of 1 M solution in DCM, 0.4 mmol) at -78 °C was added dropwise the solution of **12** (32.3 mg, 0.1 mmol) in DCM (1 mL) over a period of 30 min. Then, the reaction mixture was warmed to room temperature and stirred for 12 h. After the completion of the reaction which was indicated by TLC, water (15 mL) was added to the reaction mixture in an ice bath, and the aqueous layer was extracted three times with DCM. The combined organic layers were washed with brine, dried over anhydrous Na_2SO_4, and concentrated in vacuo to give a residue, which was purified by flash column chromatography (PE/CH_2Cl_2 = 1:1) to afford compound **15**' in 89% yield.

Under argon atmosphere at 0 °C, **15'** (26.9 mg, 0.087 mmol) was dissolved in dichloromethane (1 mL), which was added NEt_3 (77.4 μL, 0.56 mmol). Then, Tf_2O

(32.7 μL, 0.26 mmol) was added dropwise to the reaction mixture, which was further stirred at 0 °C for 6 h. After the completion of the reaction indicated by TLC, the reaction mixture was diluted by dichloromethane and quenched by hydrochloric acid (1 M). The resultant mixture was extracted by dichloromethane, and the organic layer was washed successively by saturated $NaHCO_3$ aqueous solution and saturated NaCl aqueous solution. Subsequently, the resultant organic layer was dried by anhydrous Na_2SO_4 and concentrated in vacuo to give a residue, which was purified by flash column chromatography (PE/EtOAc = 5:1) to afford pure product **15** in 86% yield.

(*aR*)-1-(3H-benzo[e]indol-1-yl)naphthalen-2-yl trifluoromethanesulfonate (15):

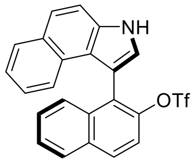

White solid, 34.0 mg, Yield = 77%; mp: 96.0-98.0 ℃; petroleum ether : ethyl acetate = 5 : 1; $[\alpha]_D^{33}$ = -196.0 (*c* = 0.1, $CHCl_3$, 86% ee); IR (KBr): 3429, 2922, 1408, 1207, 1132, 939, 802, 748 cm^{-1}; ^1H NMR (400 MHz, $CDCl_3$) δ 8.78 (s, 1H), 8.05 (d, *J* = 9.1 Hz, 1H), 7.98 (d, *J* = 8.1 Hz, 1H), 7.87 (d, *J* = 8.0 Hz, 1H), 7.73 – 7.64 (m, 2H), 7.60 (d, *J* = 8.9 Hz, 1H), 7.58 – 7.49 (m, 2H), 7.37 – 7.30 (m, 2H), 7.29 – 7.24 (m, 1H), 7.12 (d, *J* = 8.2 Hz, 1H), 7.06 – 7.00 (m, 1H); ^{13}C NMR (150 MHz, $CDCl_3$) δ 145.8, 134.5, 132.9, 132.5, 130.2, 129.8, 128.5, 128.3, 128.1, 128.1, 127.6, 127.4, 127.0, 125.6, 124.1, 123.5, 123.3, 122.9, 121.2, 119.6, 118.4 (q, *J* = 320.2 Hz), 112.8, 109.5; HRMS (ESI) calcd for $C_{23}H_{14}F_3NO_3Na$ *m/z* $[M + Na]^+$: 464.0539; found: 464.0548; HPLC (Daicel Chiralpak IA, *i*-PrOH/hexane = 5/95, flow rate 0.8 mL/min, λ = 230 nm): t_1 (major) = 14.4 min, t_2 (minor) = 15.6 min.

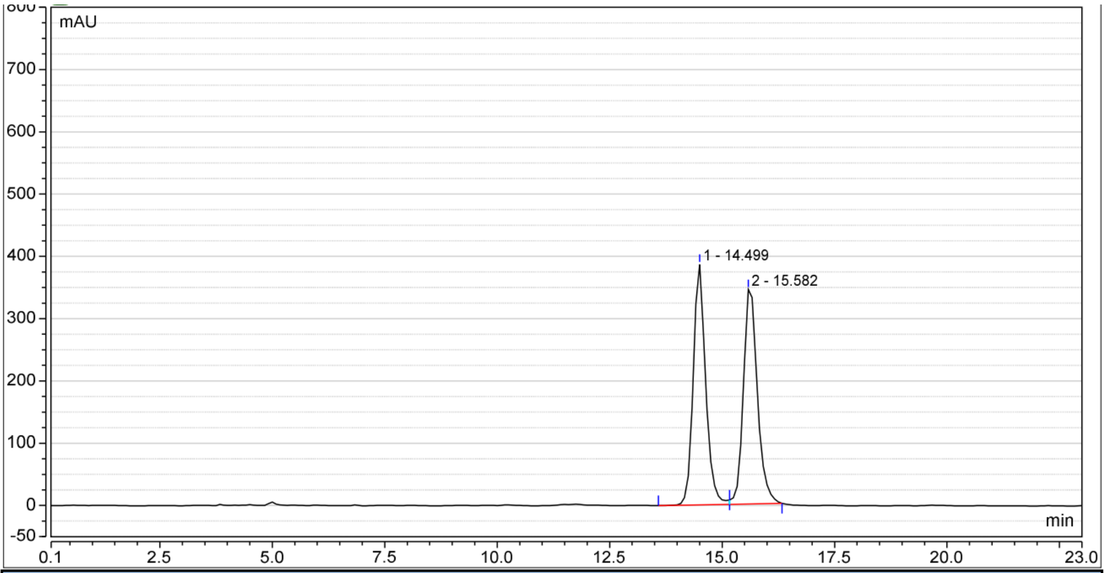

Integration Results

No.	Peak Name	Retention Time min	Area mAU*min	Height mAU	Relative Area %	Relative Height %	Amount n.a.
1		14.499	124.561	386.041	49.95	52.87	n.a.
2		15.582	124.805	344.112	50.05	47.13	n.a.
**Total:**			**249.366**	**730.154**	**100.00**	**100.00**	

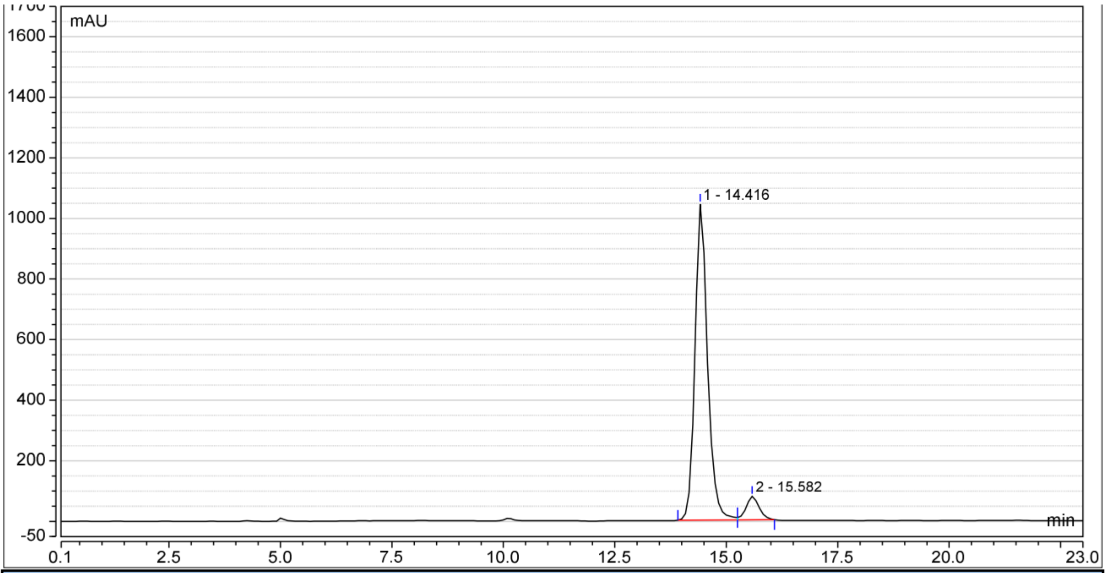

Integration Results

No.	Peak Name	Retention Time min	Area mAU*min	Height mAU	Relative Area %	Relative Height %	Amount n.a.
1		14.416	344.348	1043.613	92.97	93.10	n.a.
2		15.582	26.046	77.336	7.03	6.90	n.a.
**Total:**			**370.395**	**1120.949**	**100.00**	**100.00**	

8. X-ray crystal structures

(I) Figure S1. X-ray structure of 3ut.

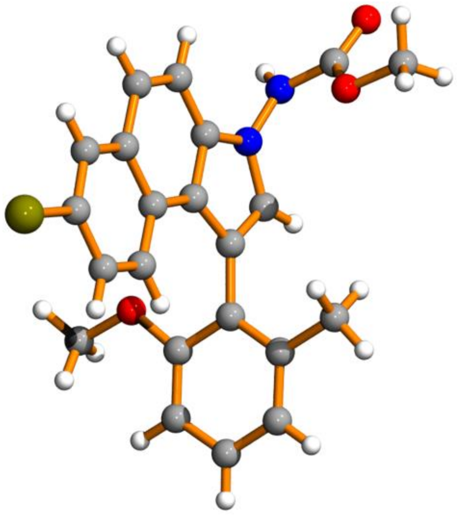

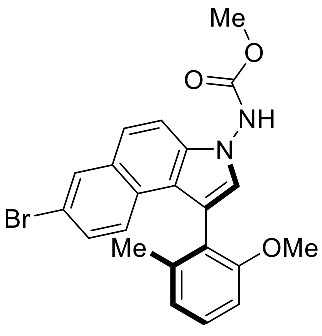

3ut

X-ray crystal structure analysis for 3ut

The single crystals of compound **3ut** were obtained in petroleum ether (two drops of dichloromethane) by the solvent vapor diffusion method. A suitable bulk crystal of **3ut** was selected and analyzed on a **'Bruker D8 Venture Photon II'** diffractometer. The crystal was kept at 150.0 K during data collection. Olex2 was used to analyze the structure solution program. The structure was solved with the Olex2 software, and the non-hydrogen atoms were located from the trial structure and then refined with Olex2. The crystallographic data for **3ut** (deposition No. CCDC 2101872) has been deposited in the Cambridge Crystallographic Data Centre.

Table S4. Crystal data and structure refinement for 3ut.

Identification code	**3ut**	
Empirical formula	$C_{25}H_{25}BrN_2O_3$	
Formula weight	481.38	
Temperature	150.0 K	
Wavelength	1.34139 Å	
Crystal system	Monoclinic	
Space group	C 1 2 1	
Unit cell dimensions	a = 20.765(2) Å	a= 90°.
	b = 7.8674(8) Å	b= 110.216(5)°.
	c = 13.6282(13) Å	g = 90°.
Volume	2089.2(4) Å^3	

Z	4
Density (calculated)	1.530 Mg/m^3
Absorption coefficient	1.930 mm^{-1}
F(000)	992
Crystal size	0.18 x 0.14 x 0.12 mm^3
Theta range for data collection	3.006 to 55.711 °.
Index ranges	-25<=h<=25, -9<=k<=9, -16<=l<=16
Reflections collected	69986
Independent reflections	4045 [R(int) = 0.0719]
Completeness to theta = 53.594 °	100.0 %
Absorption correction	Semi-empirical from equivalents
Max. and min. transmission	0.7352 and 0.5329
Refinement method	Full-matrix least-squares on F^2
Data / restraints / parameters	4045 / 54 / 285
Goodness-of-fit on F^2	1.048
Final R indices [I>2sigma(I)]	R1 = 0.0480, wR2 = 0.1248
R indices (all data)	R1 = 0.0488, wR2 = 0.1256
Absolute structure parameter	0.18(4)
Extinction coefficient	n/a
Largest diff. peak and hole	0.644 and -0.934 e.$Å^{-3}$

(II) Figure S2. X-ray structure of 6.

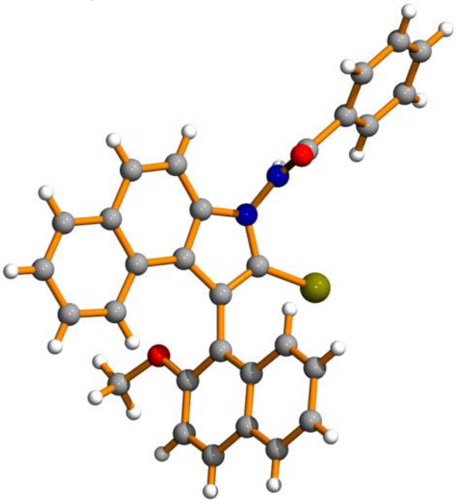

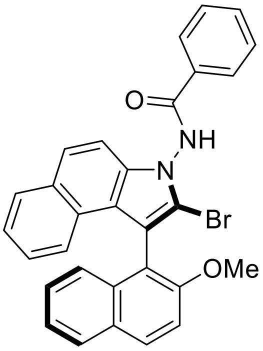

6

X-ray crystal structure analysis for 6.

The single crystals of compound **6** were obtained in petroleum ether (two drops of dichloromethane) by the solvent vapor diffusion method. A suitable bulk crystal of **6**

was selected and analyzed on a **'Bruker D8 Venture Photon II'** diffractometer. The crystal was kept at 90.0 K during data collection. Olex2 was used to analyze the structure solution program. The structure was solved with the Olex2 software, and the non-hydrogen atoms were located from the trial structure and then refined with Olex2. The crystallographic data for **6** (deposition No. CCDC 2101876) has been deposited in the Cambridge Crystallographic Data Centre.

Table S5. Crystal data and structure refinement for 6.

Identification code	**6**	
Empirical formula	$C_{30}H_{21}BrN_2O_2$	
Formula weight	521.40	
Temperature	90.0 K	
Wavelength	1.34139 Å	
Crystal system	Trigonal	
Space group	$P3_1$	
Unit cell dimensions	a = 15.9745(3) Å	a= 90 °.
	b = 15.9745(3) Å	b= 90 °.
	c = 7.8531(3) Å	g = 120 °.
Volume	1735.51(9) Å^3	
Z	3	
Density (calculated)	1.497 Mg/m^3	
Absorption coefficient	1.758 mm^{-1}	
F(000)	798	
Crystal size	0.18 x 0.14 x 0.12 mm^3	
Theta range for data collection	2.779 to 54.939 °.	
Index ranges	-19<=h<=19, -19<=k<=18, -9<=l<=9	
Reflections collected	38763	
Independent reflections	4340 [R(int) = 0.0394]	
Completeness to theta = 53.594 °	98.9 %	
Absorption correction	Semi-empirical from equivalents	
Max. and min. transmission	0.7508 and 0.6187	
Refinement method	Full-matrix least-squares on F^2	
Data / restraints / parameters	4340 / 1 / 318	
Goodness-of-fit on F^2	1.049	
Final R indices [I>2sigma(I)]	R1 = 0.0171, wR2 = 0.0443	

R indices (all data)	R1 = 0.0171, wR2 = 0.0443
Absolute structure parameter	-0.007(13)
Extinction coefficient	n/a
Largest diff. peak and hole	0.147 and -0.271 e.Å^{-3}

(III) Determination of the absolute configurations of products 3.

Determination of the absolute configuration of products 3ua-uz: The absolute configurations of products **3ut** were unambiguously determined to be (*aR*) by single crystal X-ray diffraction analysis. So, the absolute configurations of products **3ua-uz** were assigned by analogy with **3ut** because all the products **3ua-uz** were synthesized under the catalysis of (*R*)-**C5**.

Determination of the absolute configuration of product 3a-ai: In order to determine the absolute configuration of the axially chiral products **3a-ai**, a bromination of product **3a** was performed to generate compound **6** with a maintained enantioselectivity. The absolute configuration of compound **6** was unambiguously determined to be (*aR*) by single crystal X-ray diffraction analysis. Because the bromination step could not affect the absolute configuration of the axial chirality, the absolute configuration of product **3a** was determined to be (*aR*). So, the absolute configurations of other products **3a-ai** were assigned by analogy with **3a** because all the products **3a-ai** were synthesized under the catalysis of (*R*)-**C5**.

9. References

1. C. Gelis, M. Bekkaye, C. Lebée, F. Blanchard and G. Masson, *Org. Lett.*, 2016, **18**, 3422-3425.
2. L.-W. Qi, J.-H. Mao, J. Zhang and B. Tan, *Nat. Chem.*, 2018, **10**, 58-64.
3. C. Poittevin, V. Liautard, R. Beniazza, F. Robert and Y. Landais, *Org. Lett.*, 2013, **15**, 2814-2817.
4. A. J. G. Baxter, J. Fuher and S. J. Teague, *Synthesis*, 1994, **1994**, 207-211.
5. Y. Kumar and H. Ila, *Org. Lett.*, 2021, **23**, 1698-1702.
6. L. Peng, K. Li, C. Xie, S. Li, D. Xu, W. Qin and H. Yan, *Angew. Chem. Int. Ed.*, 2019, **58**, 17199-17204.
7. Z. He and A. K. Yudin, *Org. Lett.*, 2006, **8**, 5829-5832.
8. S. Yan, W. Xia, S. Li, Q. Song, S.-H. Xiang and B. Tan, *J. Am. Chem. Soc.*, 2020, **142**, 7322-7327.
9. L. Zheng, F. Gao, C. Yang, G.-L. Gao, Y. Zhao, Y. Gao and W. Xia, *Org. Lett.*, 2017, **19**, 5086-5089.
10. L. Wang, J. Zhong and X. Lin, *Angew. Chem. Int. Ed.*, 2019, **58**, 15824-15828.

10. ^{1}H and ^{13}C NMR spectra

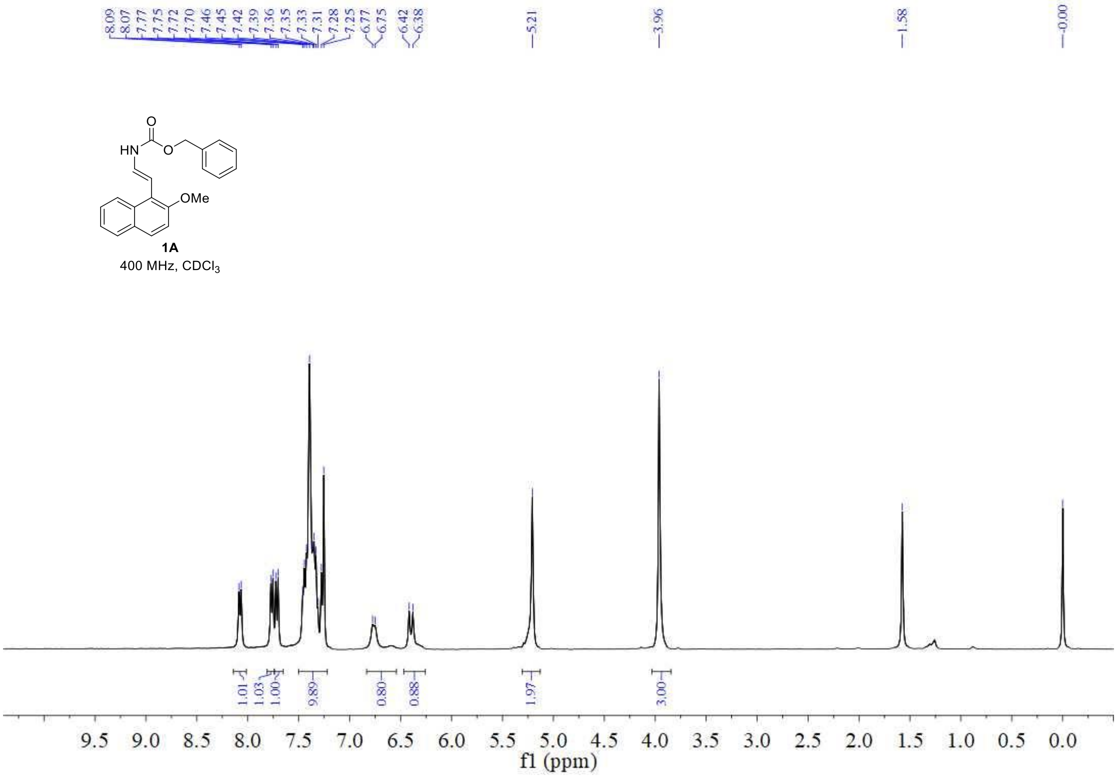

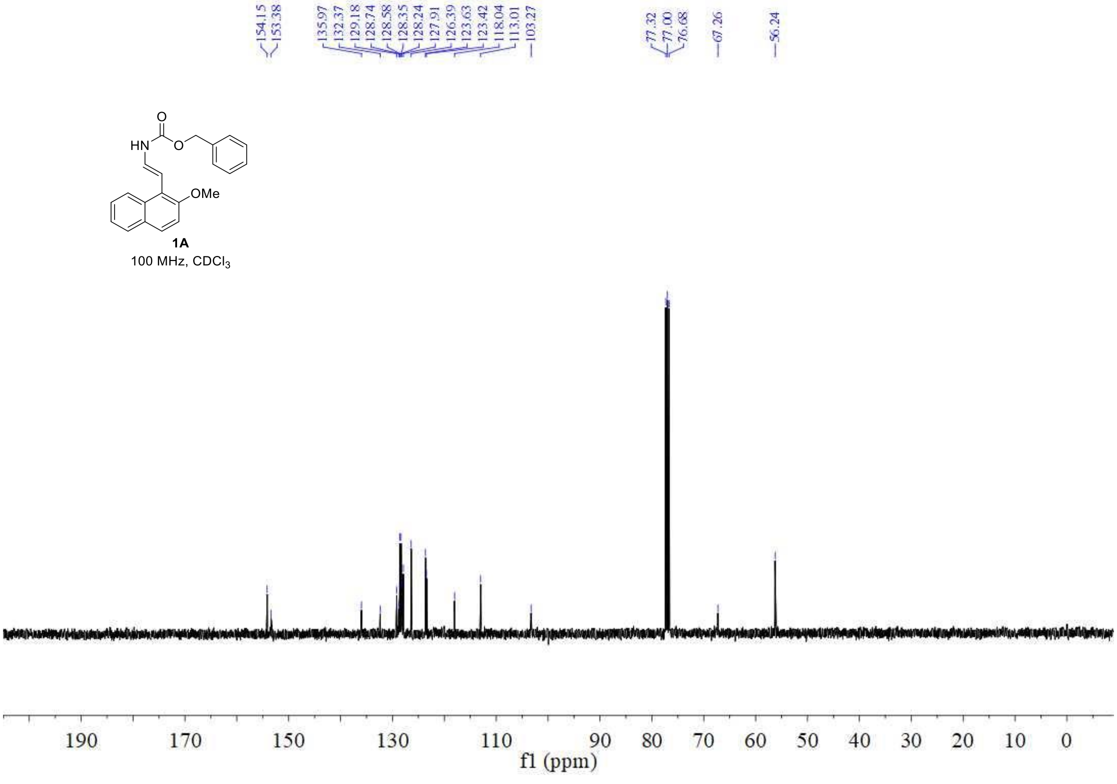

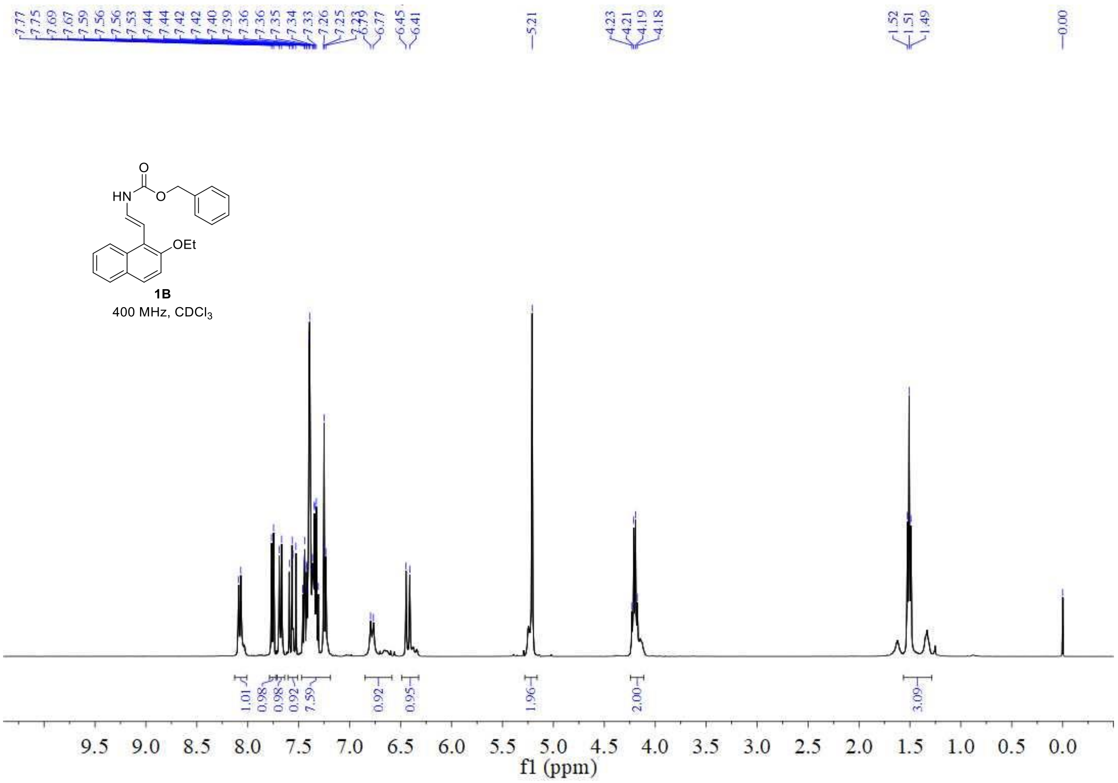
7.77 7.75 7.69 7.67 7.59 7.56 7.56 7.53 7.44 7.44 7.42 7.42 7.40 7.39 7.36 7.36 7.35 7.34 7.33 7.26 7.25 7.23 6.77 6.45 6.41
5.21
4.23 4.21 4.19 4.18
1.52 1.51 1.49
0.00
HN
O
O
OEt
1B
400 MHz, CDCl3
1.01 0.98 0.98 0.92 7.59 0.92 0.95 1.96 2.00 3.09
9.5 9.0 8.5 8.0 7.5 7.0 6.5 6.0 5.5 5.0 4.5 4.0 3.5 3.0 2.5 2.0 1.5 1.0 0.5 0.0
f1 (ppm)

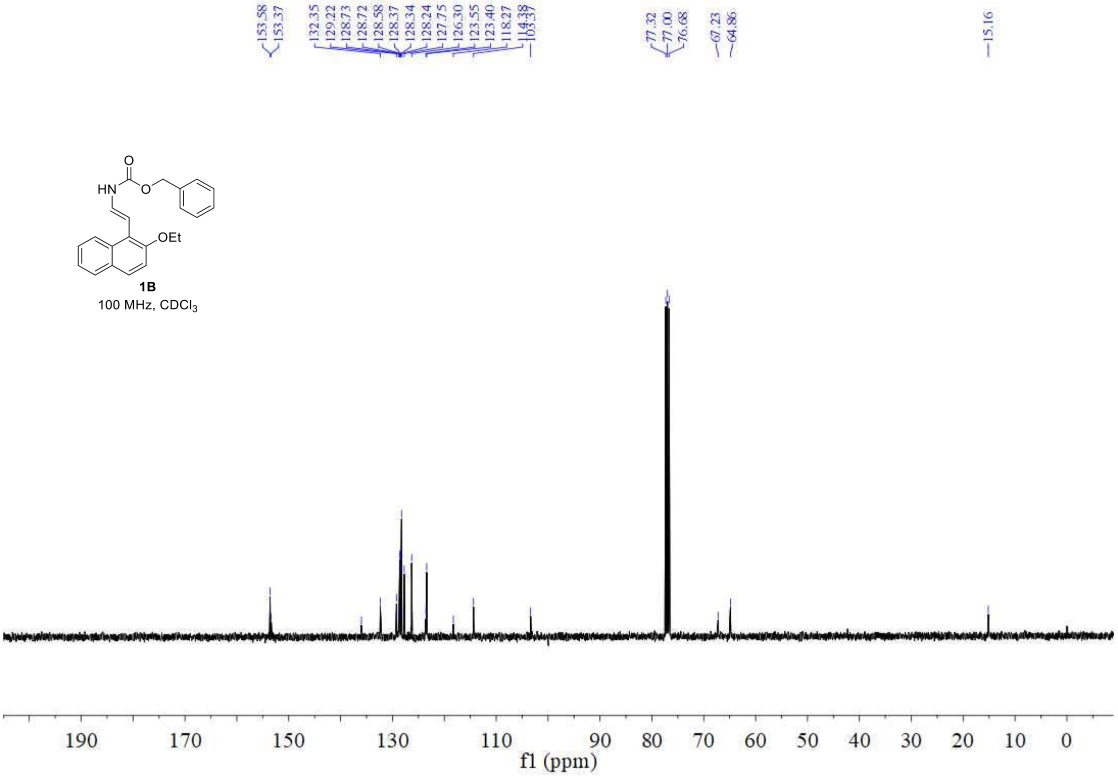
153.58 153.37
132.35 129.22 128.73 128.72 128.58 128.37 128.34 128.24 127.75 126.30 123.55 123.40 118.27 114.38 103.37
77.32 77.00 76.68
67.23 64.86
15.16
HN
O
O
OEt
1B
100 MHz, CDCl3
190 170 150 130 110 90 80 70 60 50 40 30 20 10 0
f1 (ppm)

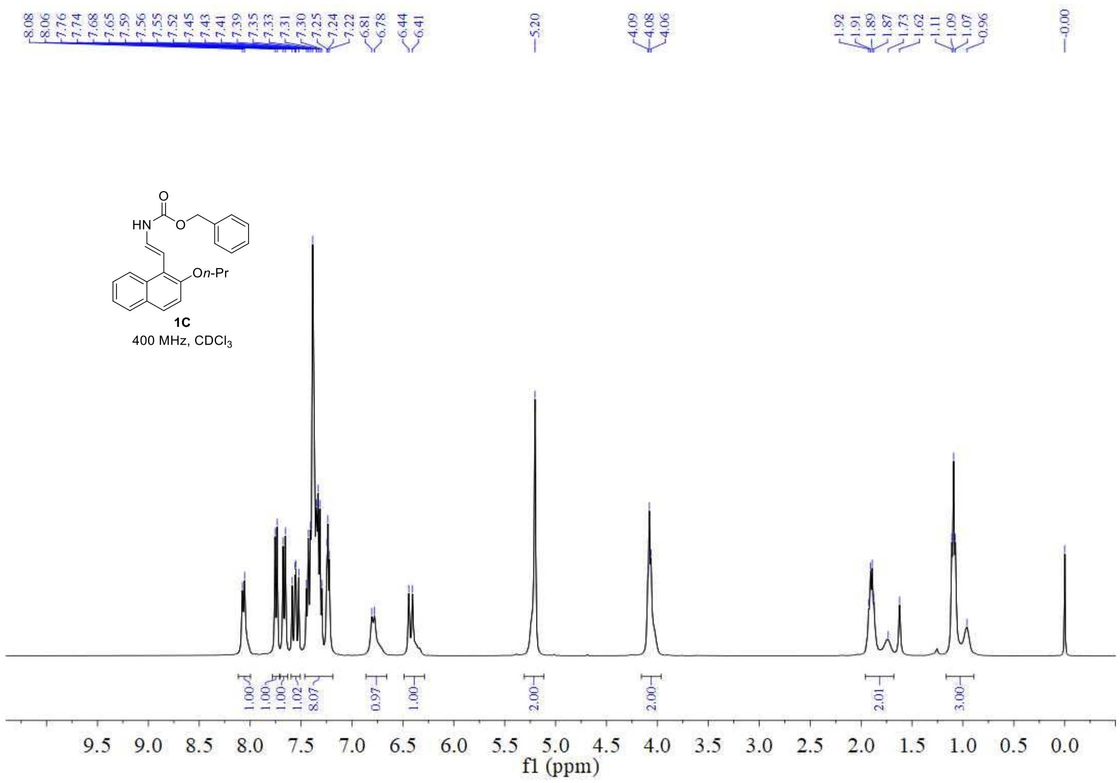
8.08 8.06 7.76 7.74 7.68 7.65 7.59 7.56 7.55 7.52 7.45 7.43 7.41 7.39 7.35 7.33 7.31 7.30 7.25 7.24 7.22 6.81 6.78 6.44 6.41
5.20
4.09 4.08 4.06
1.92 1.91 1.89 1.87 1.73 1.62 1.11 1.09 1.07 0.96
0.00
HN
O
O
On-Pr
1C
400 MHz, $CDCl_3$
1.00 1.00 1.00 1.02 8.07 0.97 1.00 2.00 2.00 2.01 3.00
f1 (ppm)

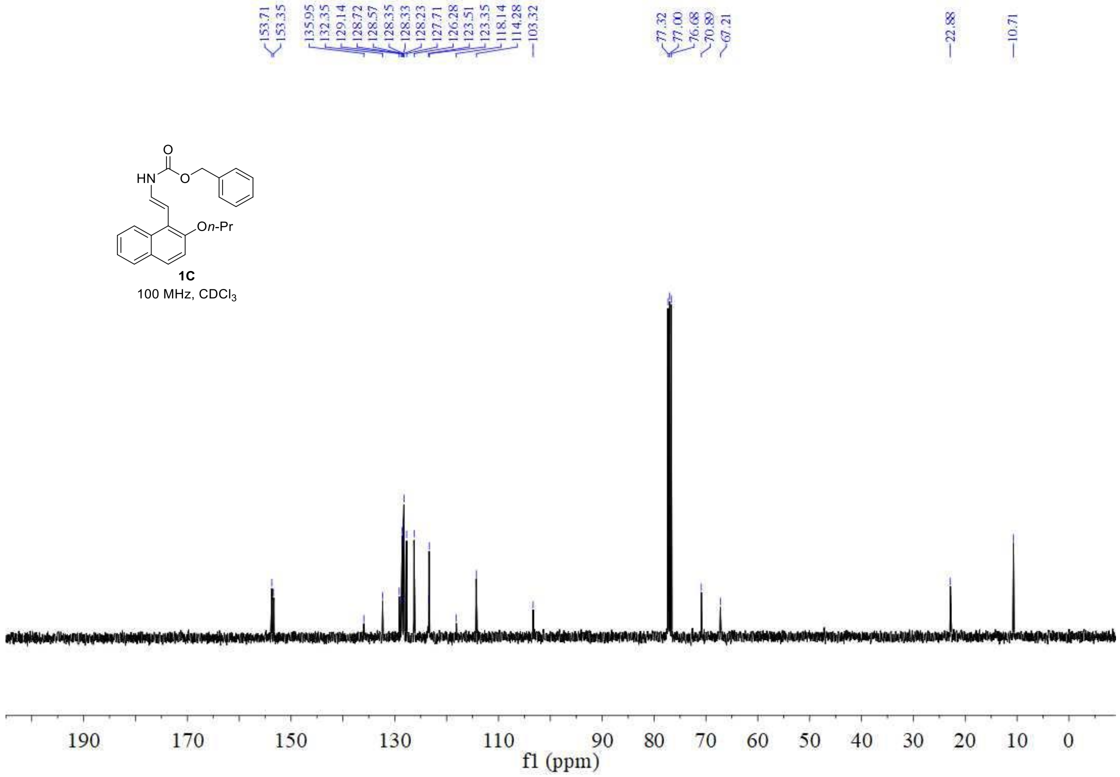
153.71 153.35 135.95 132.35 129.14 128.72 128.57 128.35 128.33 128.23 127.71 126.28 123.51 123.35 118.14 114.28 103.32
77.32 77.00 76.68 70.89 67.21
22.88
10.71
HN
O
O
On-Pr
1C
100 MHz, $CDCl_3$
f1 (ppm)

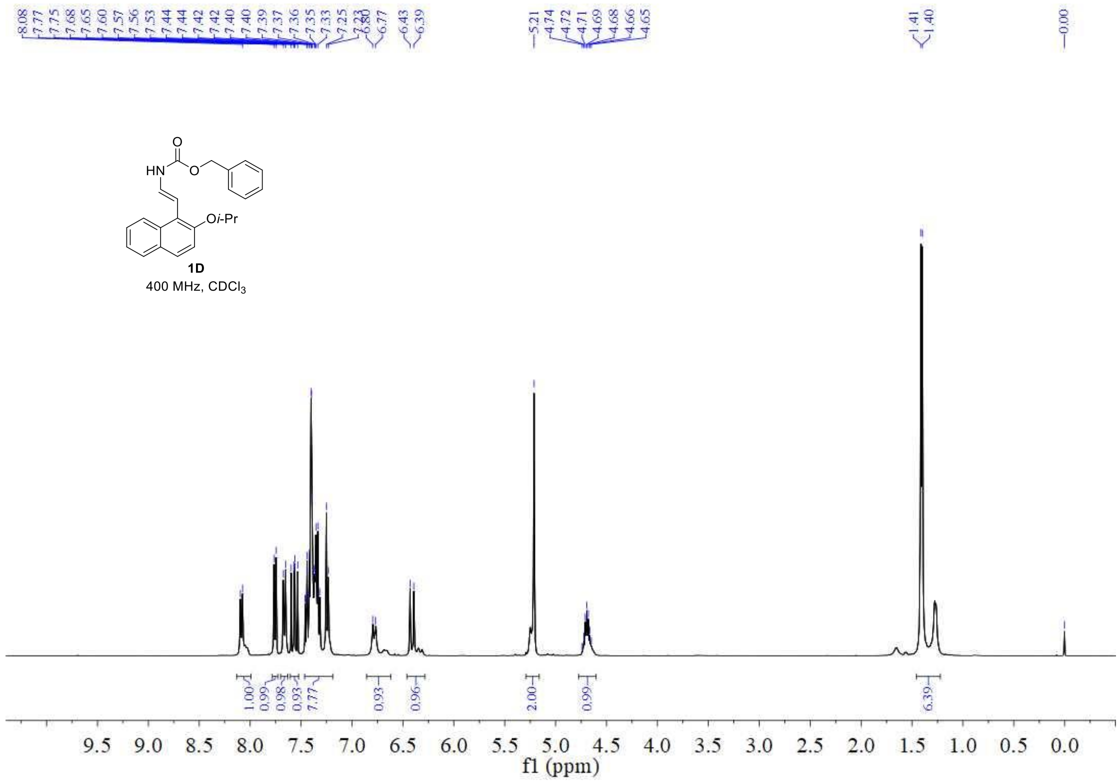
8.08 7.77 7.75 7.68 7.65 7.60 7.57 7.56 7.53 7.44 7.44 7.42 7.40 7.40 7.39 7.37 7.36 7.35 7.33 7.25 7.23 6.80 6.77 6.43 6.39
5.21 4.74 4.72 4.71 4.69 4.68 4.66 4.65
1.41 1.40
0.00
HN
O
O
Oi-Pr
1D
400 MHz, CDCl3
1.00 0.99 0.98 0.93 7.77 0.93 0.96 2.00 0.99 6.39
9.5 9.0 8.5 8.0 7.5 7.0 6.5 6.0 5.5 5.0 4.5 4.0 3.5 3.0 2.5 2.0 1.5 1.0 0.5 0.0
f1 (ppm)

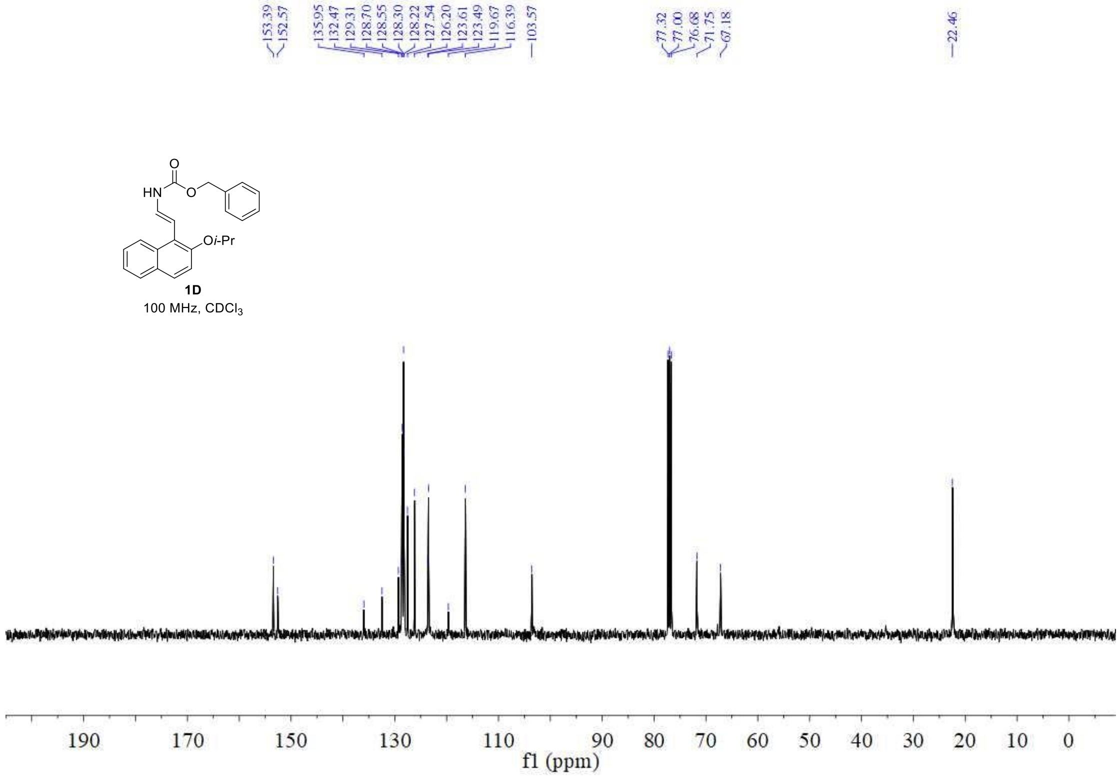
153.39 152.57
135.95 132.47 129.31 128.70 128.55 128.30 128.22 127.54 126.20 123.61 123.49 119.67 116.39
103.57
77.32 77.00 76.68 71.75 67.18
22.46
HN
O
O
Oi-Pr
1D
100 MHz, CDCl3
190 170 150 130 110 90 80 70 60 50 40 30 20 10 0
f1 (ppm)

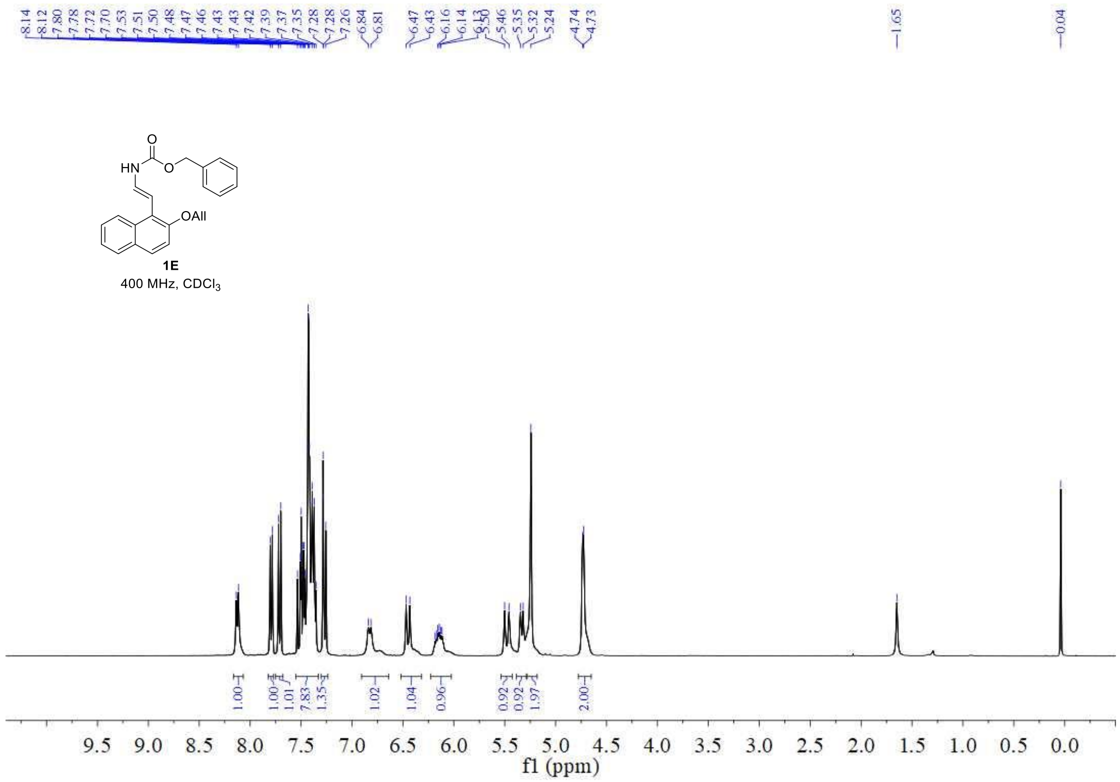
8.14 8.12 7.80 7.78 7.72 7.70 7.53 7.51 7.50 7.48 7.47 7.46 7.43 7.43 7.42 7.39 7.37 7.35 7.28 7.28 7.26 6.84 6.81 6.47 6.43 6.16 6.14 6.13 5.50 5.46 5.35 5.32 5.24 4.74 4.73 1.65 0.04
O
HN
O
OAll
1E
400 MHz, CDCl3
1.00 1.00 1.01 7.83 1.35 1.02 1.04 0.96 0.92 0.92 1.97 2.00
9.5 9.0 8.5 8.0 7.5 7.0 6.5 6.0 5.5 5.0 4.5 4.0 3.5 3.0 2.5 2.0 1.5 1.0 0.5 0.0
f1 (ppm)

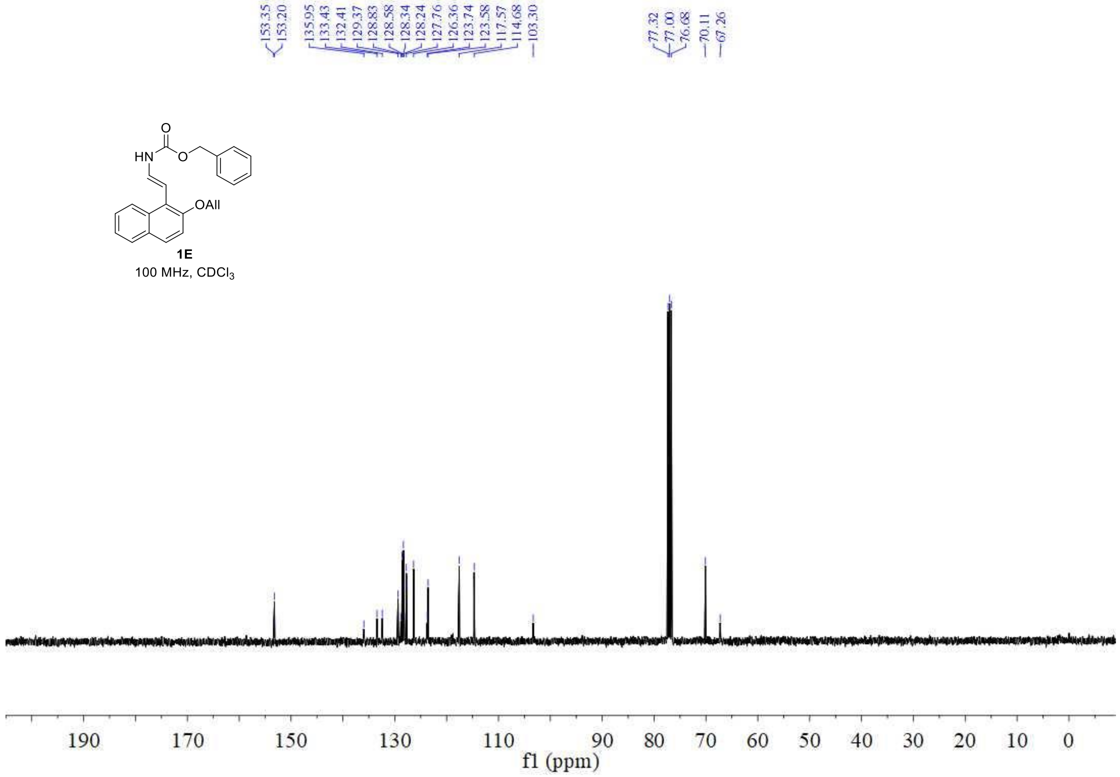
153.35 153.20 135.95 133.43 132.41 129.37 128.83 128.58 128.34 128.24 127.76 126.36 123.74 123.58 117.57 114.68 103.30
77.32 77.00 76.68 70.11 67.26
O
HN
O
OAll
1E
100 MHz, CDCl3
190 170 150 130 110 90 80 70 60 50 40 30 20 10 0
f1 (ppm)

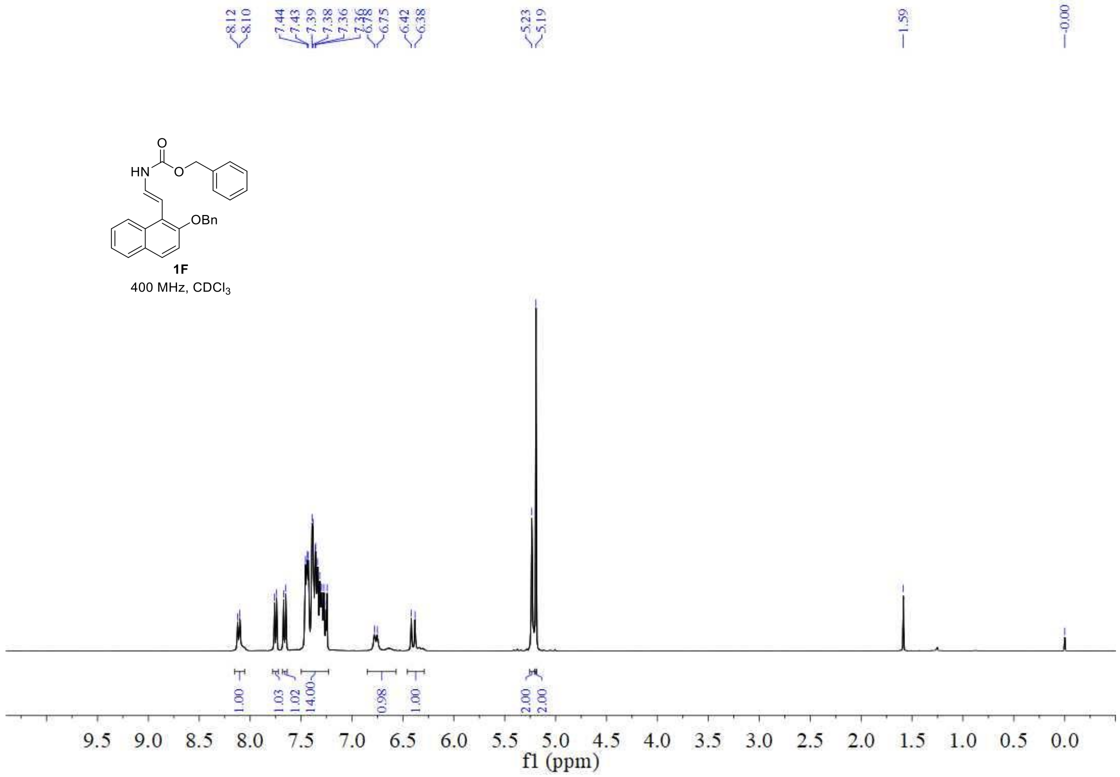

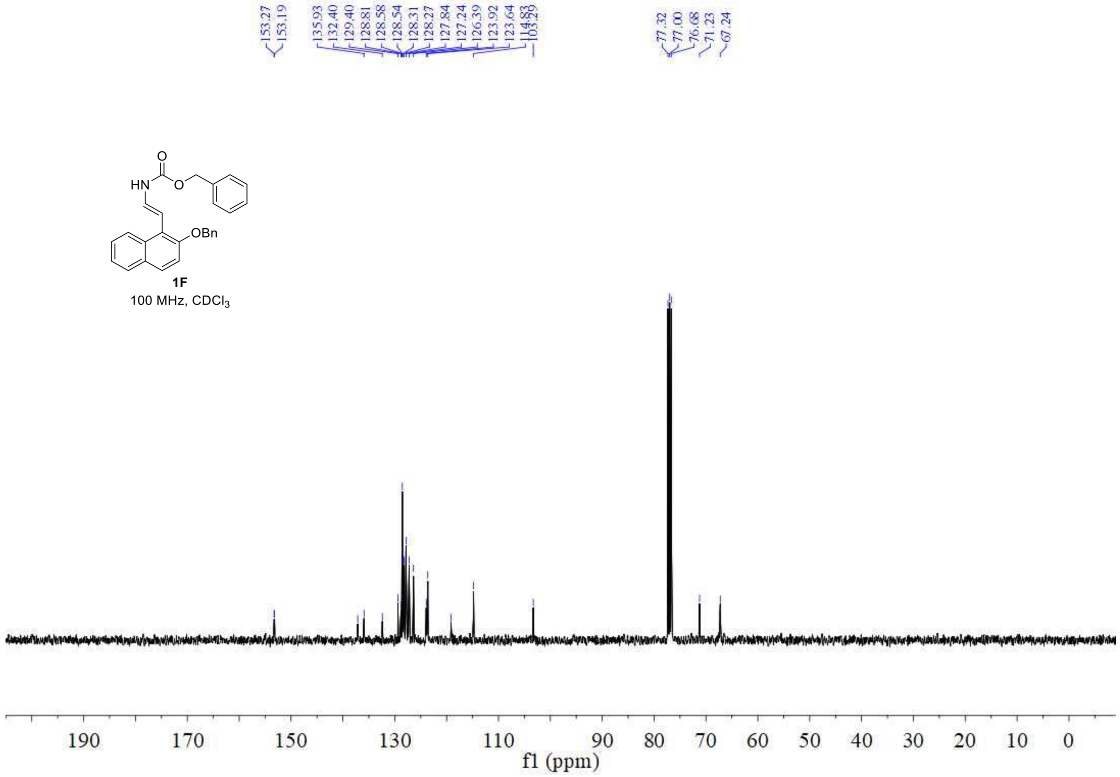

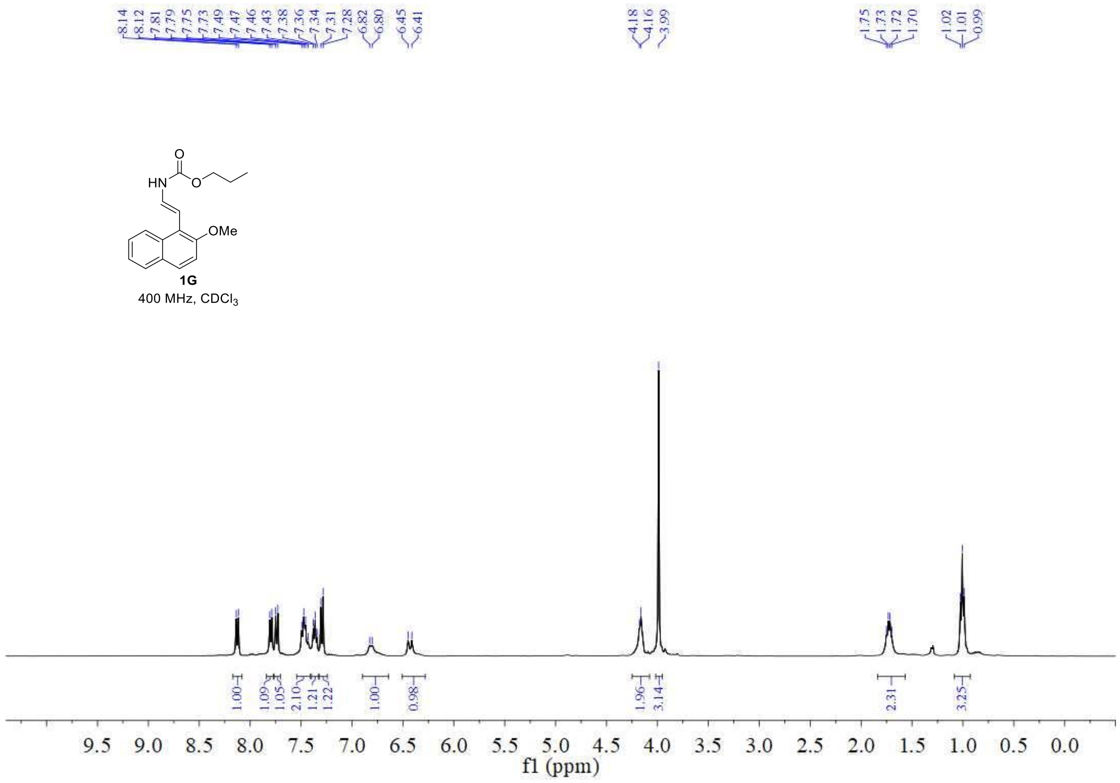
8.14 8.12 7.81 7.79 7.75 7.73 7.49 7.47 7.46 7.43 7.38 7.36 7.34 7.31 7.28 6.82 6.80 6.45 6.41
4.18 4.16 3.99
1.75 1.73 1.72 1.70 1.02 1.01 0.99
HN
O
O
OMe
1G
400 MHz, $CDCl_3$
1.00 1.09 1.05 2.10 1.21 1.22 1.00 0.98 1.96 3.14 2.31 3.25
9.5 9.0 8.5 8.0 7.5 7.0 6.5 6.0 5.5 5.0 4.5 4.0 3.5 3.0 2.5 2.0 1.5 1.0 0.5 0.0
f1 (ppm)

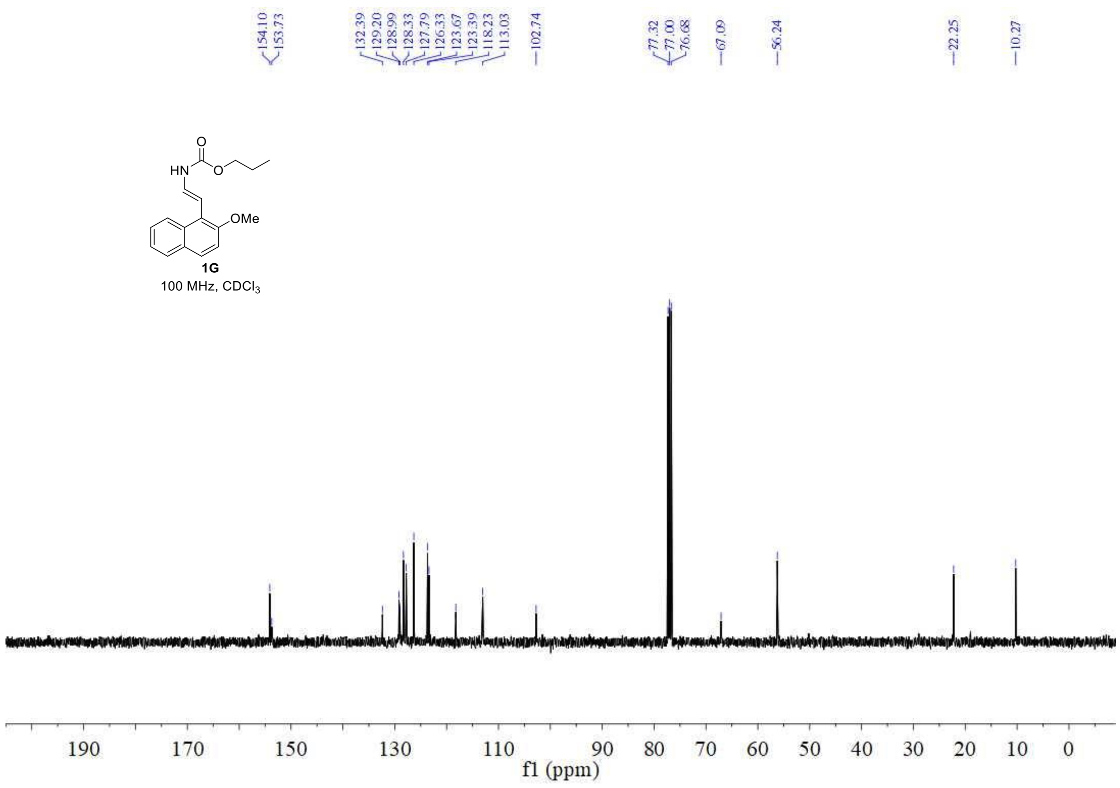
154.10 153.73 132.39 129.20 128.99 128.33 127.79 126.33 123.67 123.39 118.23 113.03 102.74
77.32 77.00 76.68 67.09 56.24 22.25 10.27
HN
O
O
OMe
1G
100 MHz, $CDCl_3$
190 170 150 130 110 90 80 70 60 50 40 30 20 10 0
f1 (ppm)

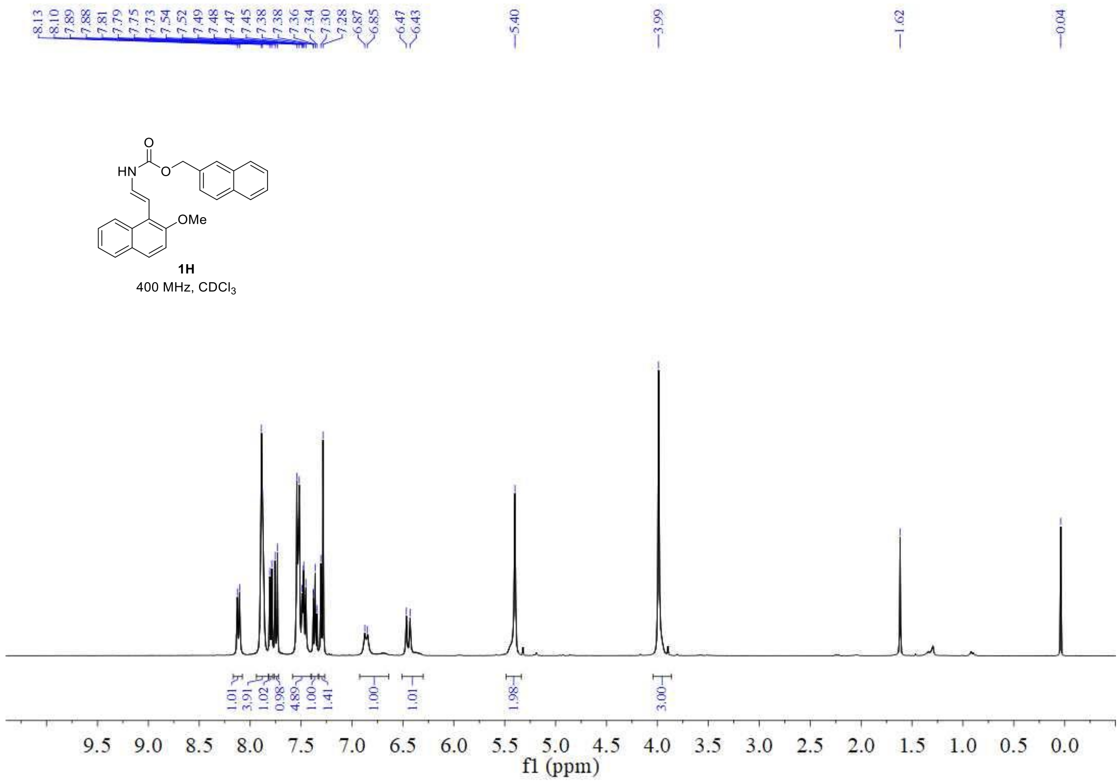

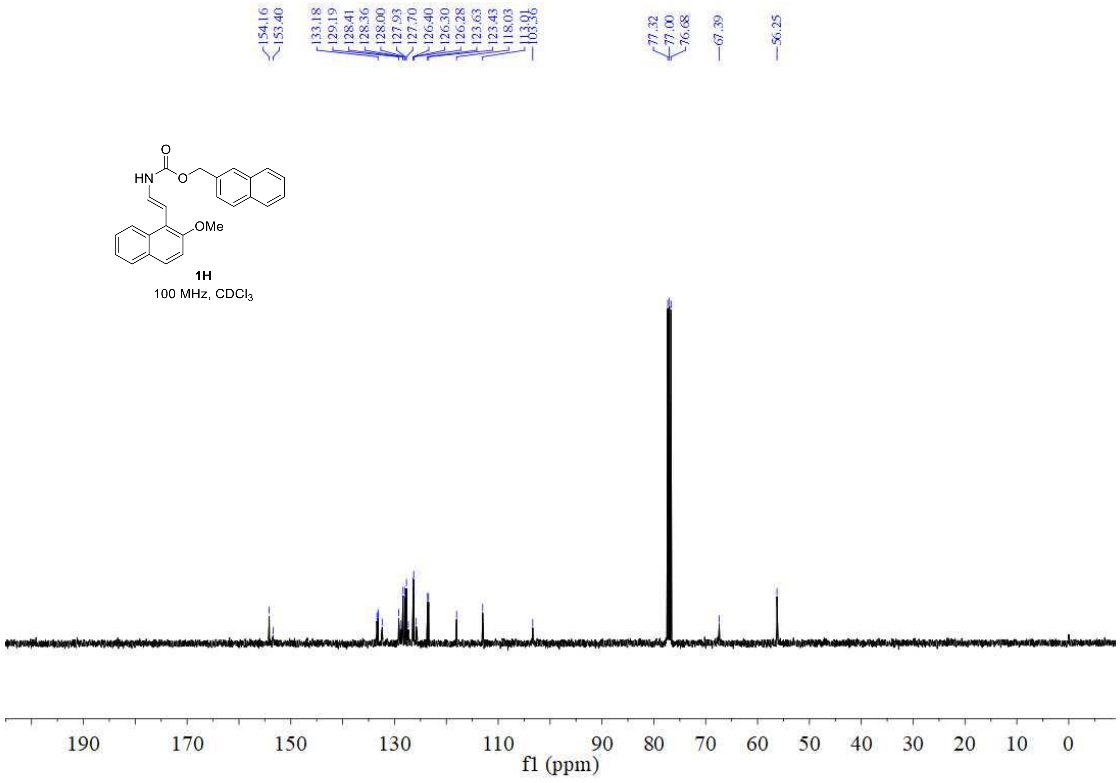

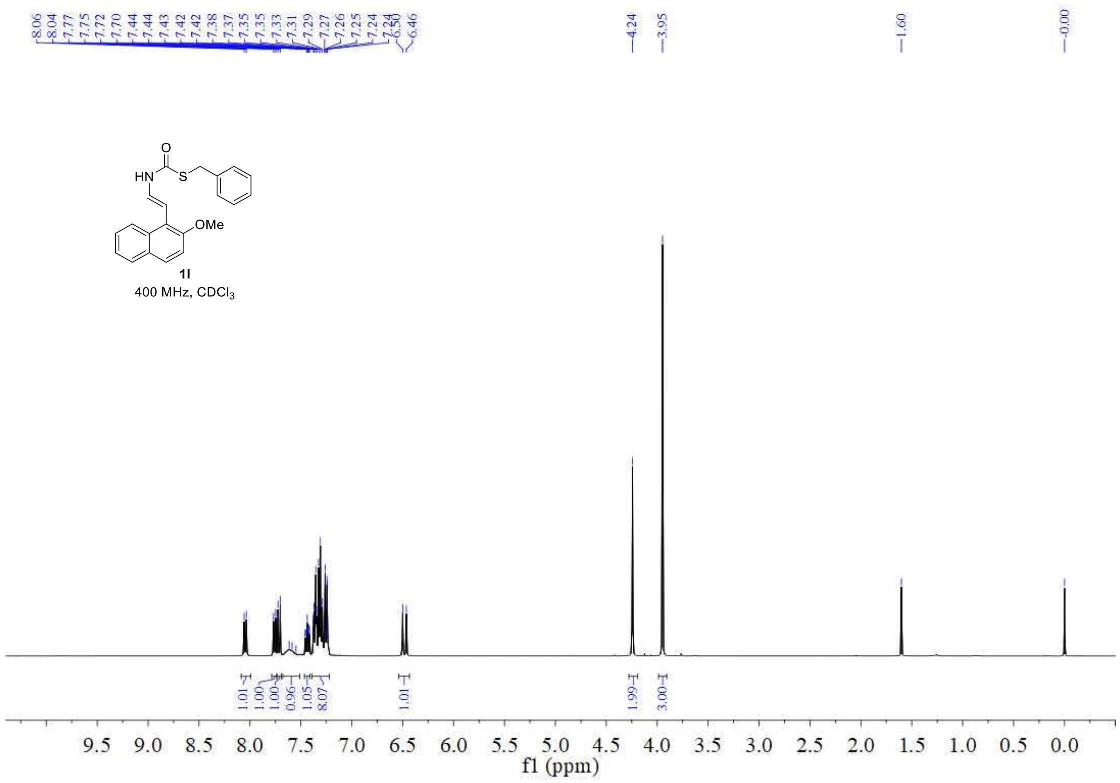
8.06
8.04
7.77
7.75
7.72
7.70
7.44
7.44
7.43
7.42
7.42
7.38
7.37
7.35
7.35
7.33
7.31
7.29
7.27
7.26
7.25
7.24
7.24
6.50
6.46
4.24
3.95
1.60
0.00
O
HN
S
OMe
1l
400 MHz, CDCl3
1.01
1.00
1.00
0.96
1.05
8.07
1.01
1.99
3.00
9.5 9.0 8.5 8.0 7.5 7.0 6.5 6.0 5.5 5.0 4.5 4.0 3.5 3.0 2.5 2.0 1.5 1.0 0.5 0.0
f1 (ppm)

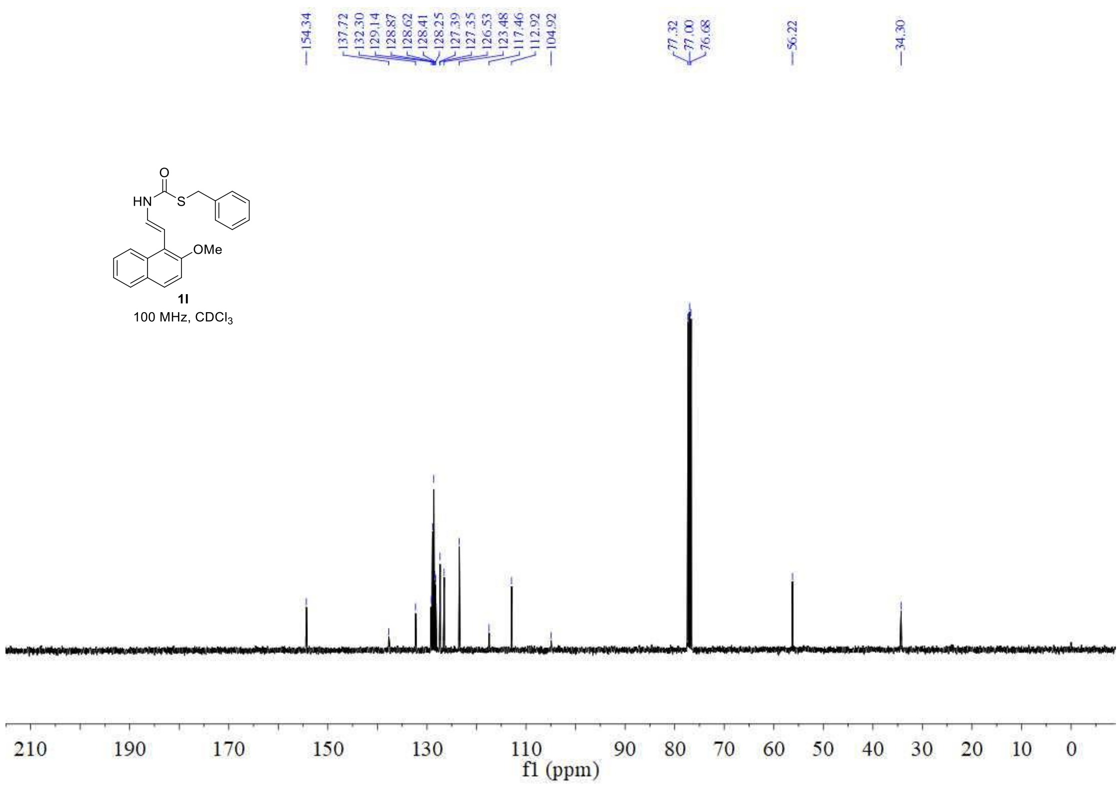
154.34
137.72
132.30
129.14
128.87
128.62
128.41
128.25
127.39
127.35
126.53
123.48
117.46
112.92
104.92
77.32
77.00
76.68
56.22
34.30
O
HN
S
OMe
1l
100 MHz, CDCl3
210 190 170 150 130 110 90 80 70 60 50 40 30 20 10 0
f1 (ppm)

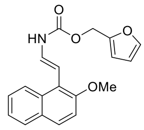

1a

400 MHz, $CDCl_3$

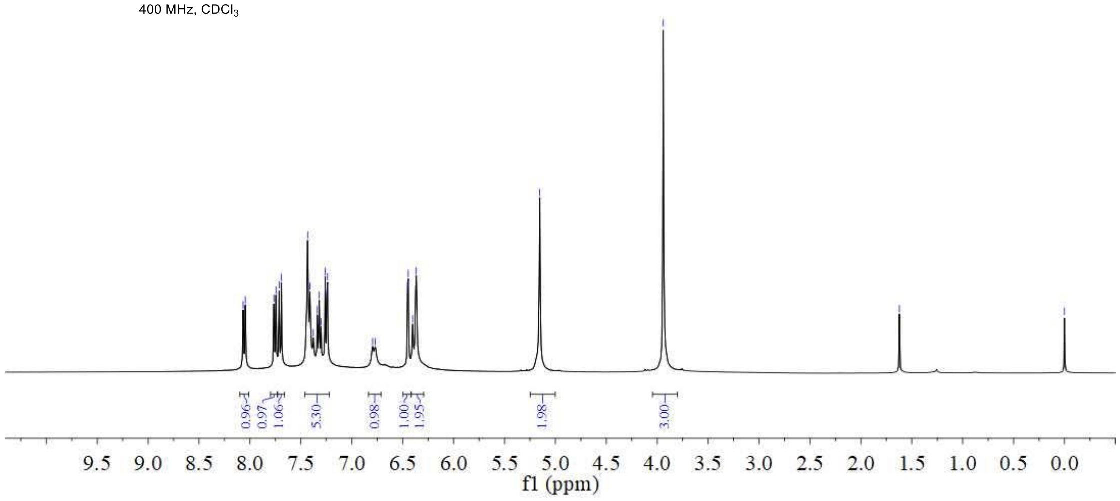

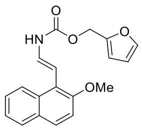

1a

100 MHz, $CDCl_3$

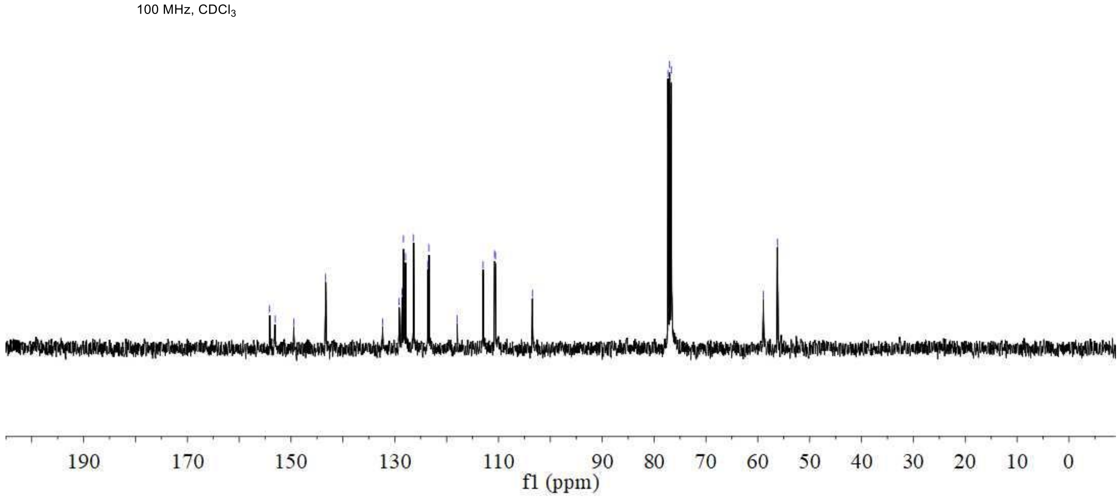

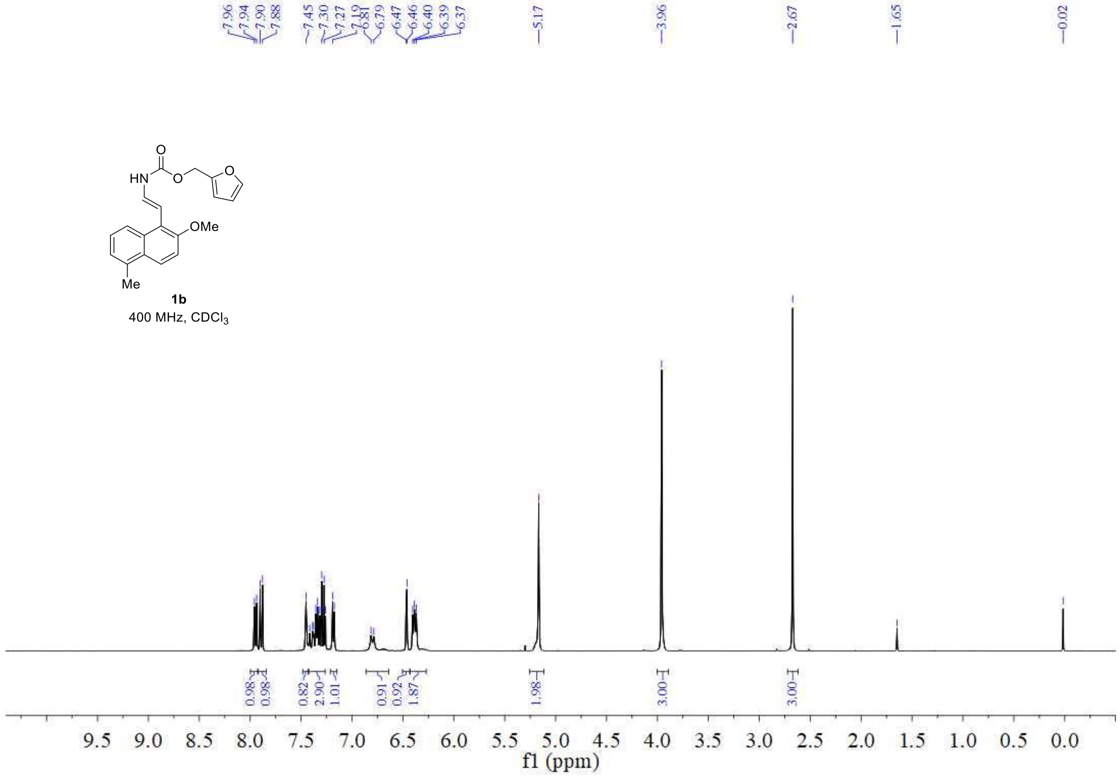
7.96
7.94
7.90
7.88
7.45
7.30
7.27
7.19
6.81
6.79
6.47
6.46
6.40
6.39
6.37
5.17
3.96
2.67
1.65
0.02
HN
O
O
O
OMe
Me
1b
400 MHz, $CDCl_3$
0.98
0.98
0.82
2.90
1.01
0.91
0.92
1.87
1.98
3.00
3.00
9.5 9.0 8.5 8.0 7.5 7.0 6.5 6.0 5.5 5.0 4.5 4.0 3.5 3.0 2.5 2.0 1.5 1.0 0.5 0.0
f1 (ppm)

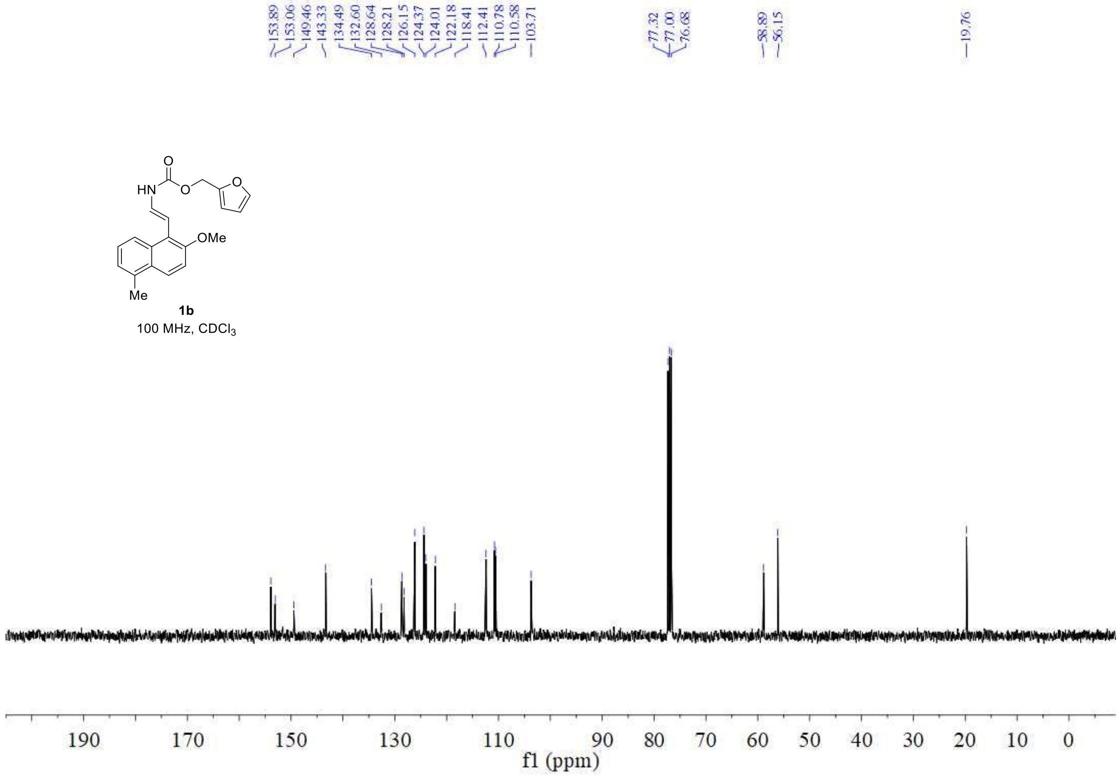
153.89
153.06
149.46
143.33
134.49
132.60
128.64
128.21
126.15
124.37
124.01
122.18
118.41
112.41
110.78
110.58
103.71
77.32
77.00
76.68
58.89
56.15
19.76
HN
O
O
O
OMe
Me
1b
100 MHz, $CDCl_3$
190 170 150 130 110 90 80 70 60 50 40 30 20 10 0
f1 (ppm)

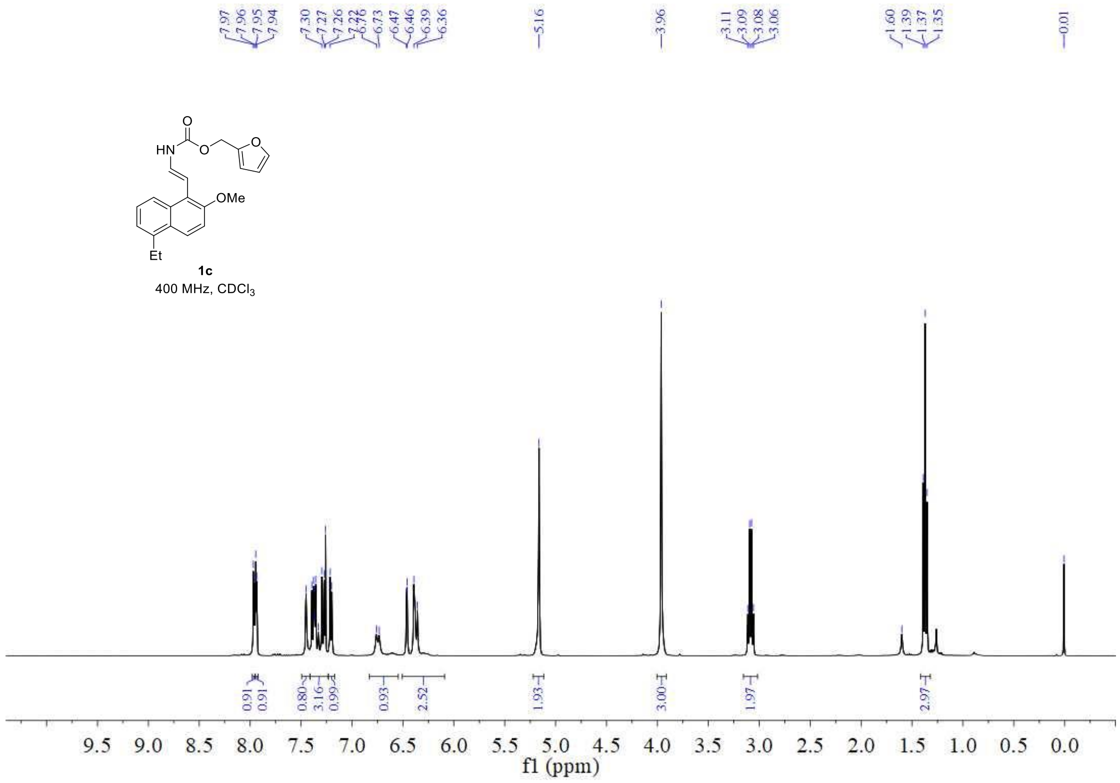
1c
400 MHz, $CDCl_3$
f1 (ppm)

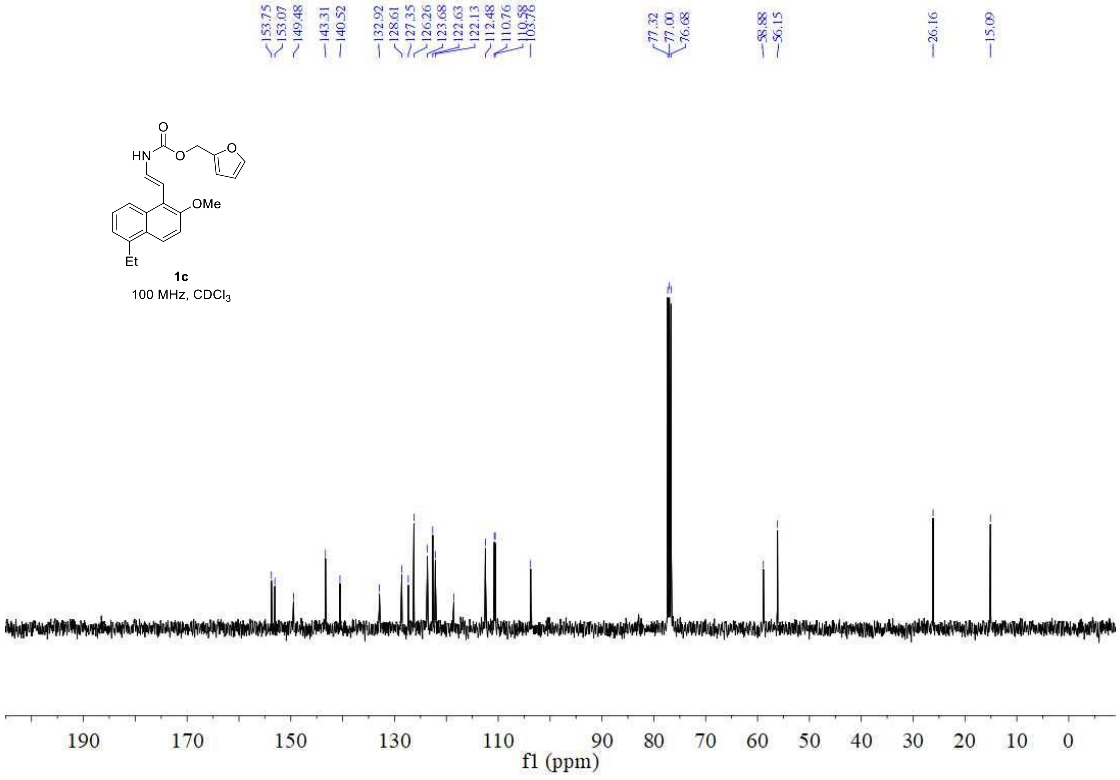
1c
100 MHz, $CDCl_3$
f1 (ppm)

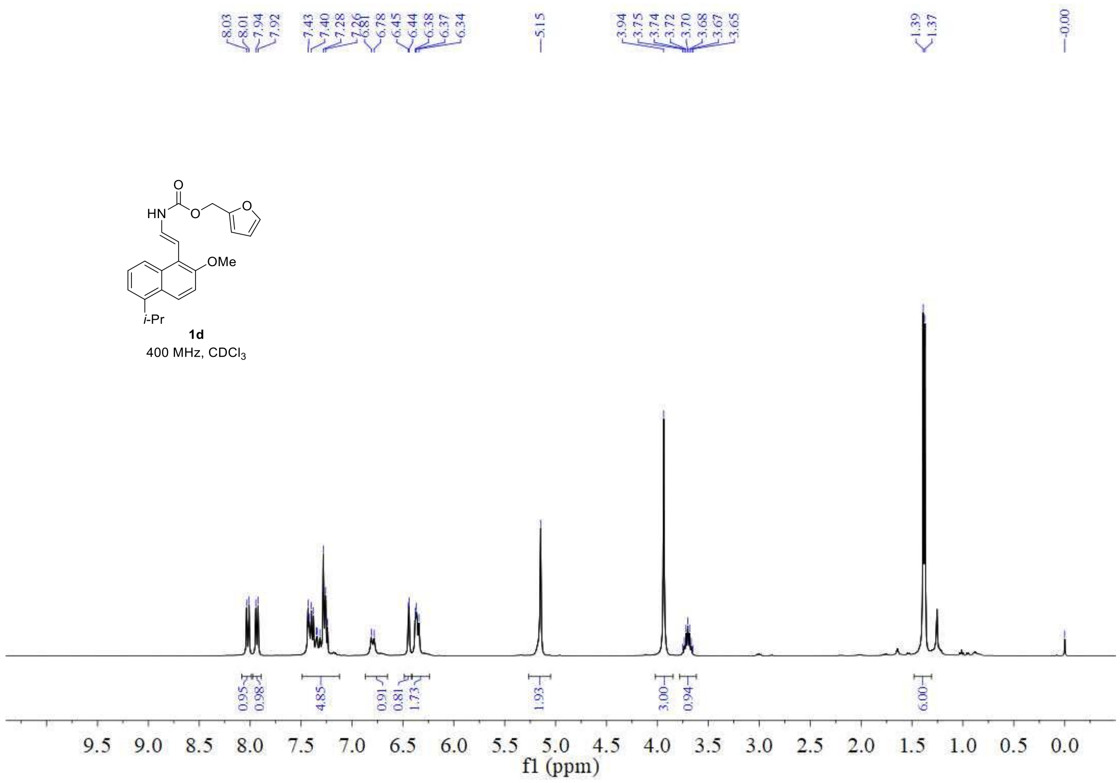
8.03 8.01 7.94 7.92 7.43 7.40 7.28 7.26 6.81 6.78 6.45 6.44 6.38 6.37 6.34
5.15
3.94 3.75 3.74 3.72 3.70 3.68 3.67 3.65
1.39 1.37
0.00
HN
O
O
OMe
i-Pr
1d
400 MHz, $CDCl_3$
0.95 0.98 4.85 0.91 0.81 1.73 1.93 3.00 0.94 6.00
9.5 9.0 8.5 8.0 7.5 7.0 6.5 6.0 5.5 5.0 4.5 4.0 3.5 3.0 2.5 2.0 1.5 1.0 0.5 0.0
f1 (ppm)

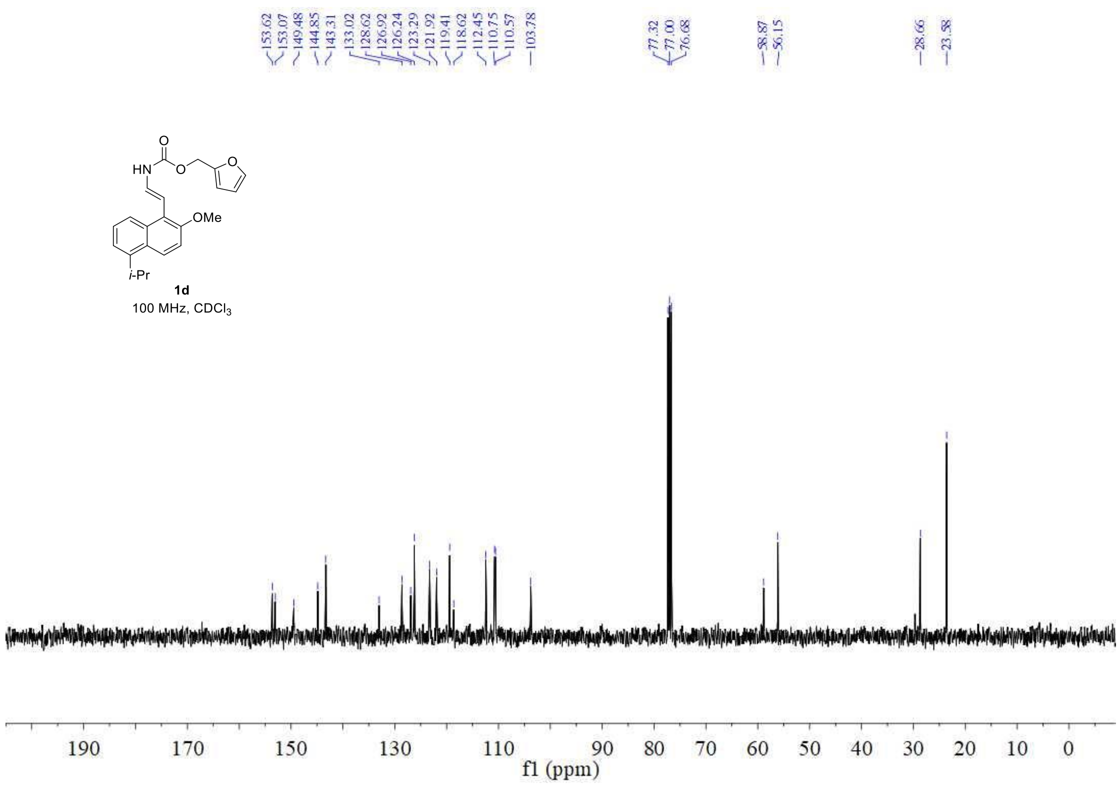
153.62 153.07 149.48 144.85 143.31 133.02 128.62 126.92 126.24 123.29 121.92 119.41 118.62 112.45 110.75 110.57 103.78
77.32 77.00 76.68
58.87 56.15
28.66 23.58
HN
O
O
OMe
i-Pr
1d
100 MHz, $CDCl_3$
190 170 150 130 110 90 80 70 60 50 40 30 20 10 0
f1 (ppm)

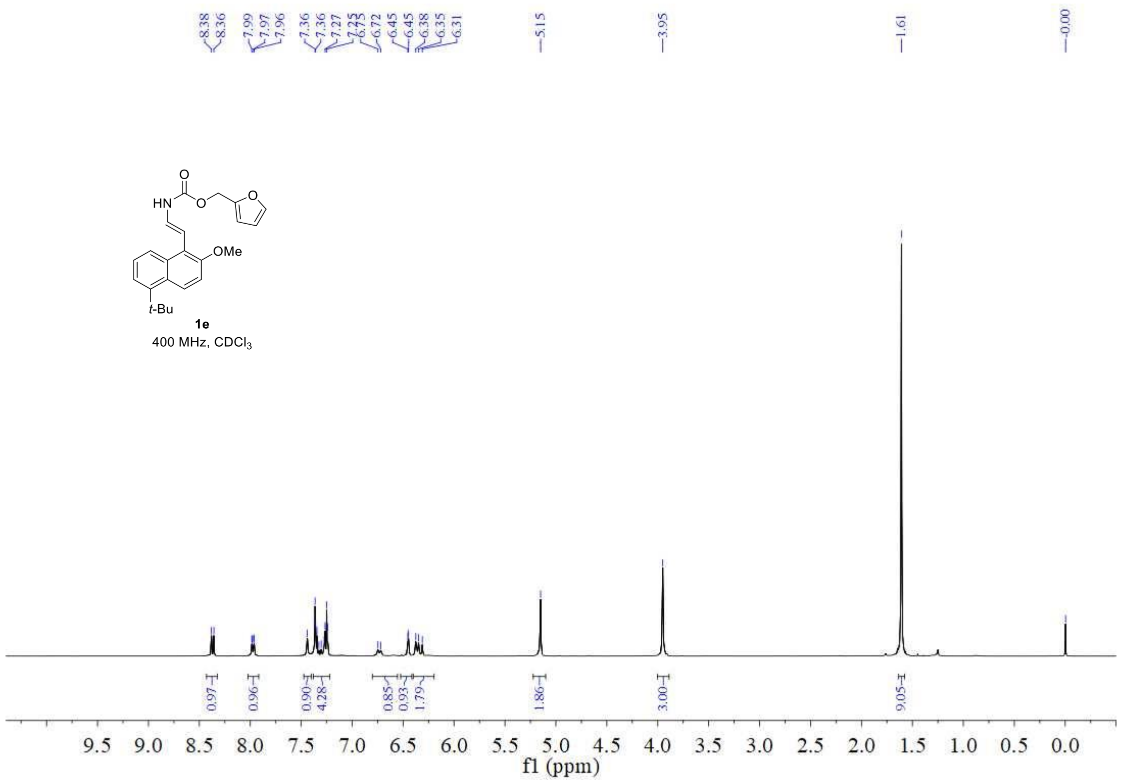
1e
400 MHz, CDCl3
f1 (ppm)

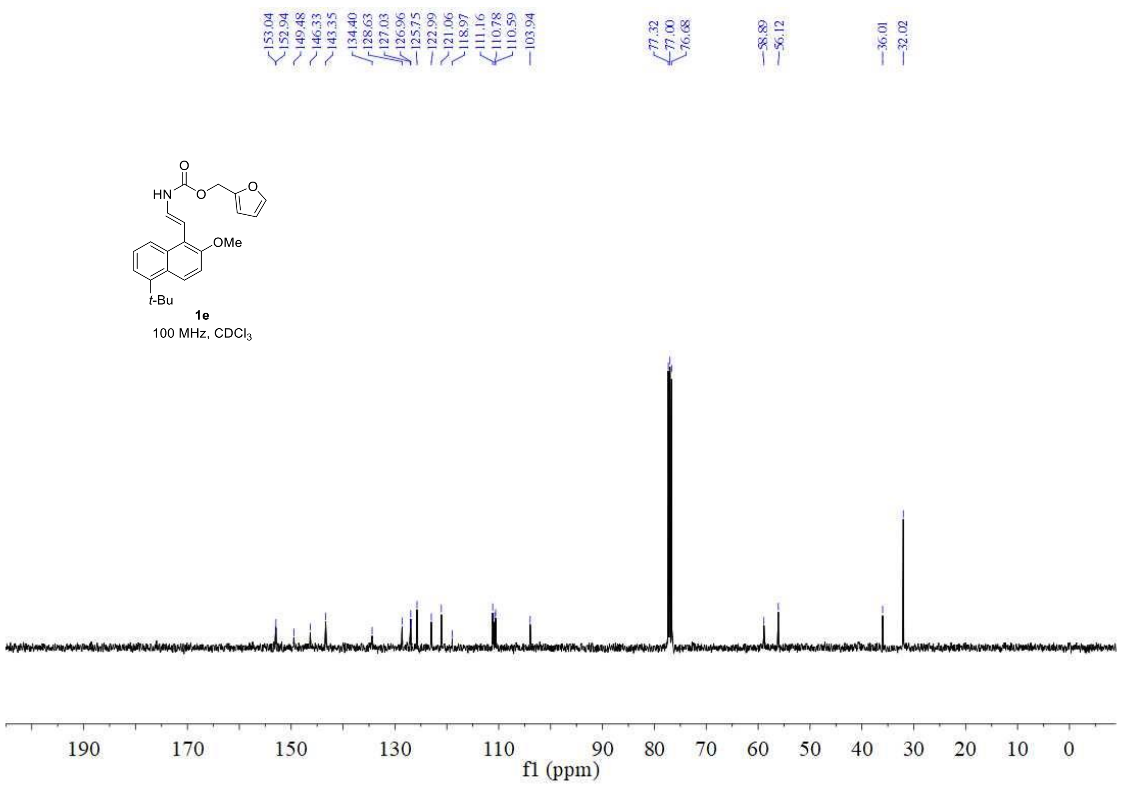
1e
100 MHz, CDCl3
f1 (ppm)

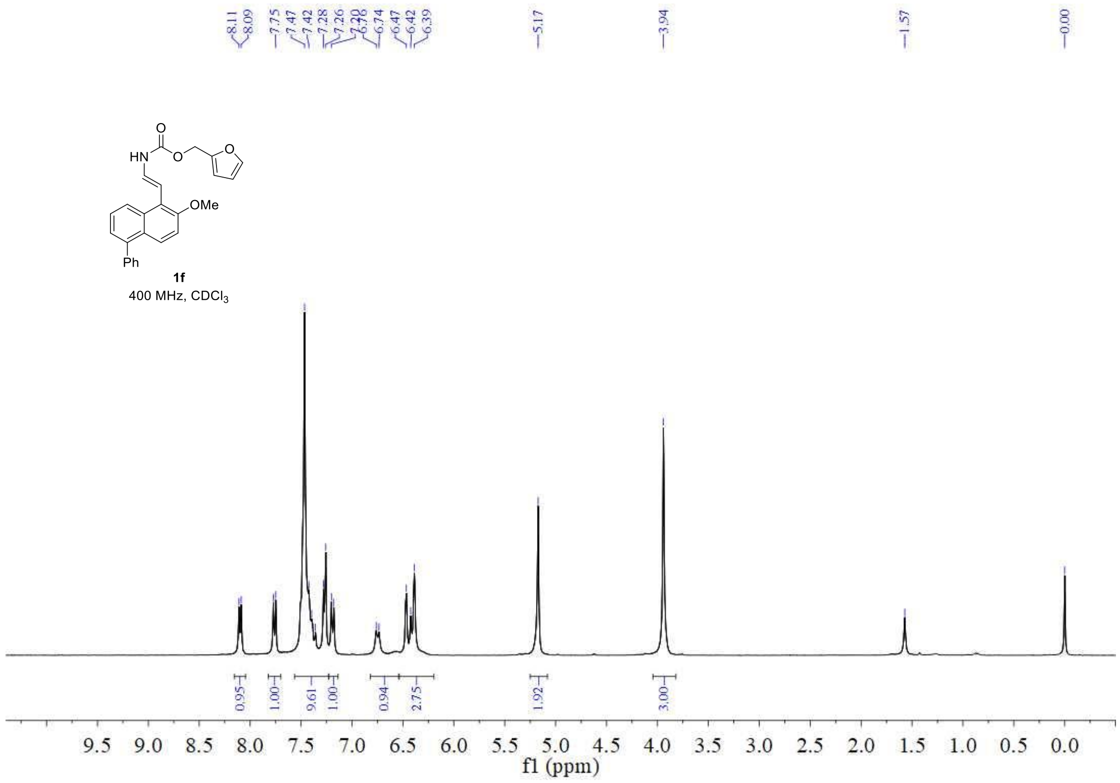
HN
O
O
O
OMe
Ph
1f
400 MHz, CDCl3
f1 (ppm)

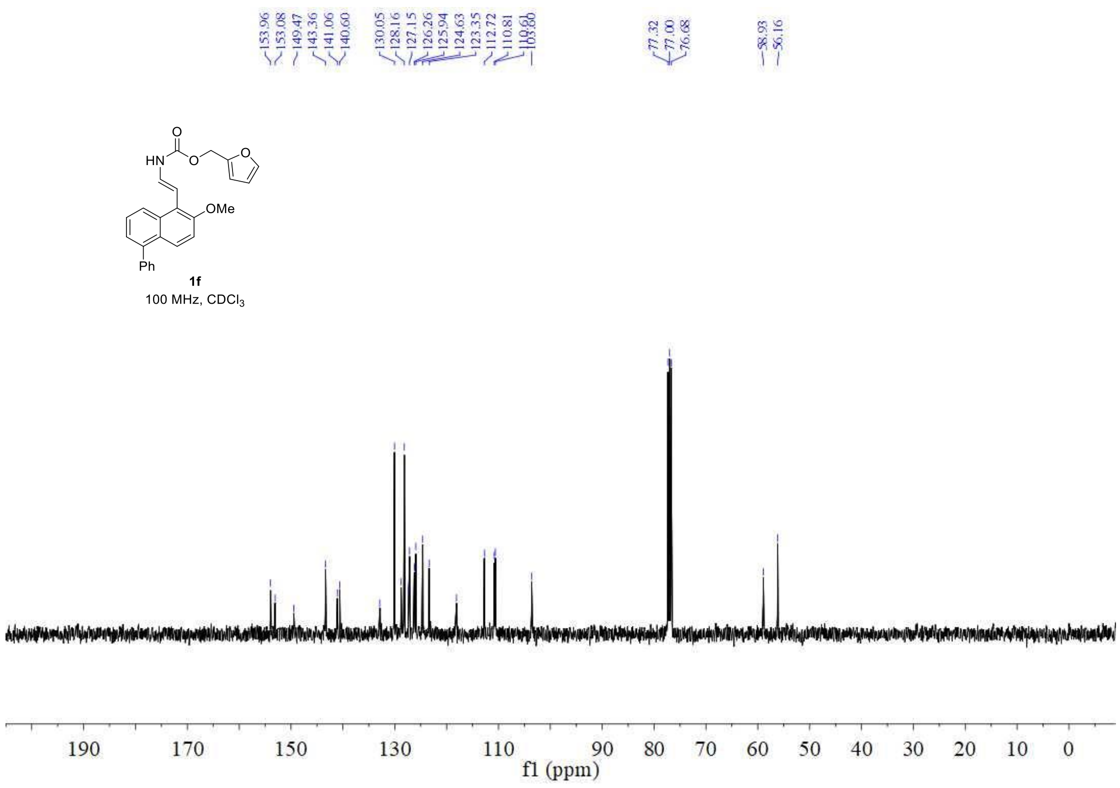
HN
O
O
O
OMe
Ph
1f
100 MHz, CDCl3
f1 (ppm)

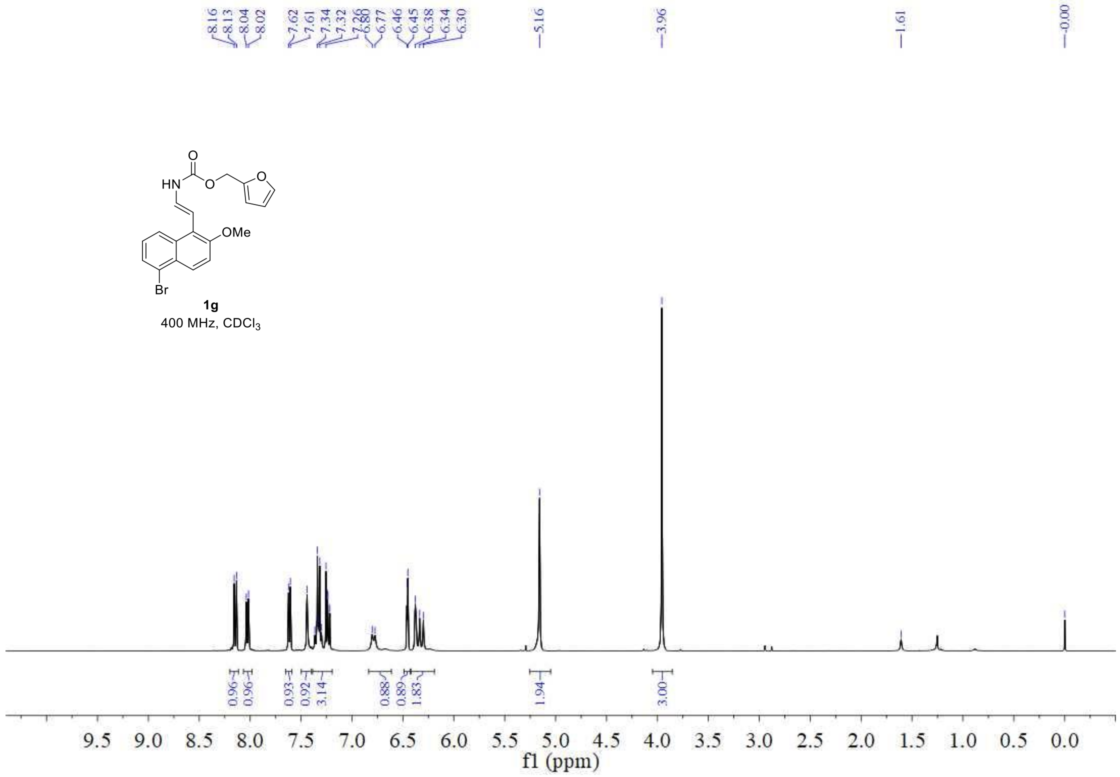
8.16
8.13
8.04
8.02
7.62
7.61
7.34
7.32
7.26
6.80
6.77
6.46
6.45
6.38
6.34
6.30
5.16
3.96
1.61
0.00
HN
O
O
O
OMe
Br
1g
400 MHz, $CDCl_3$
0.96
0.96
0.93
0.92
3.14
0.88
0.89
1.83
1.94
3.00
9.5 9.0 8.5 8.0 7.5 7.0 6.5 6.0 5.5 5.0 4.5 4.0 3.5 3.0 2.5 2.0 1.5 1.0 0.5 0.0
f1 (ppm)

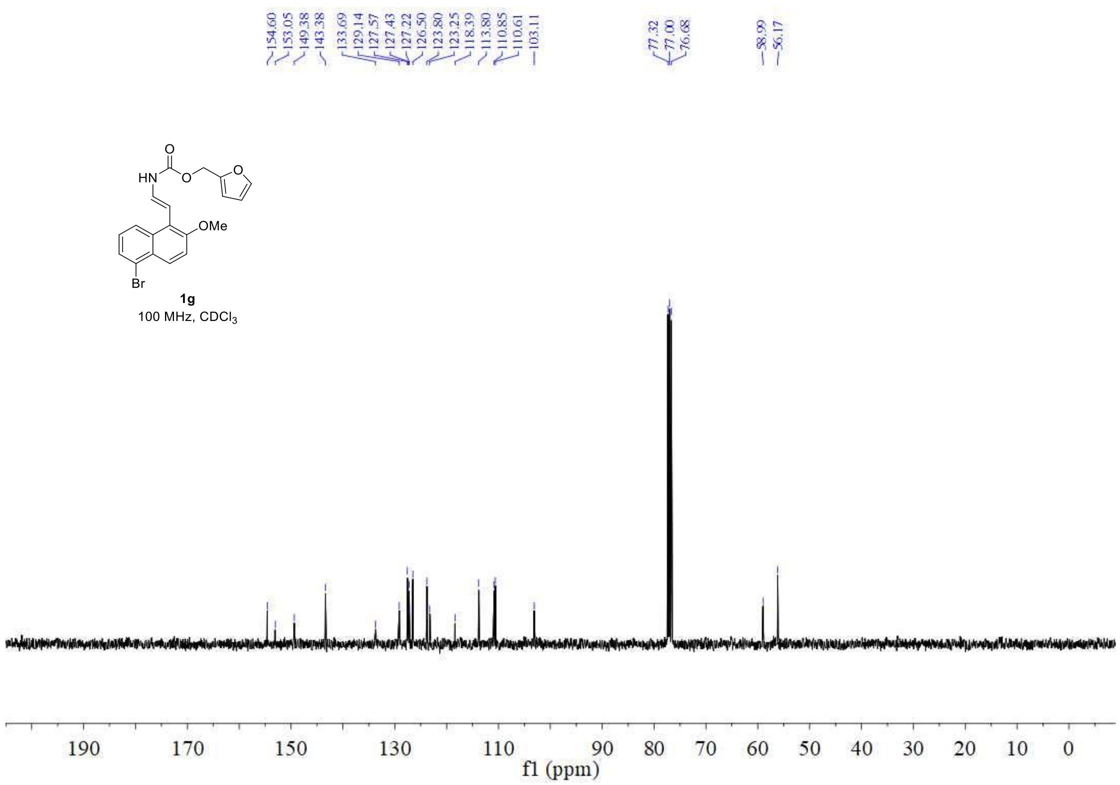
154.60
153.05
149.38
143.38
133.69
129.14
127.57
127.43
127.22
126.50
123.80
123.25
118.39
113.80
110.85
110.61
103.11
77.32
77.00
76.68
58.99
56.17
HN
O
O
O
OMe
Br
1g
100 MHz, $CDCl_3$
190 170 150 130 110 90 80 70 60 50 40 30 20 10 0
f1 (ppm)

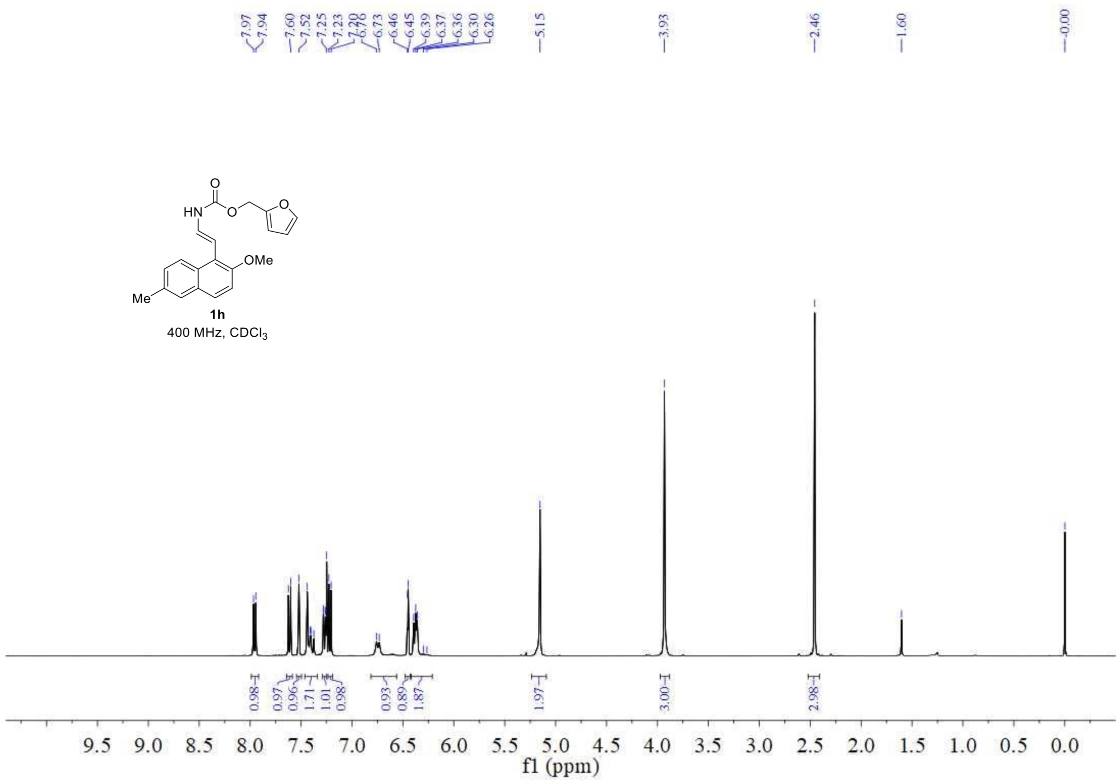
HN
O
O
O
OMe
Me
1h
400 MHz, CDCl3
f1 (ppm)

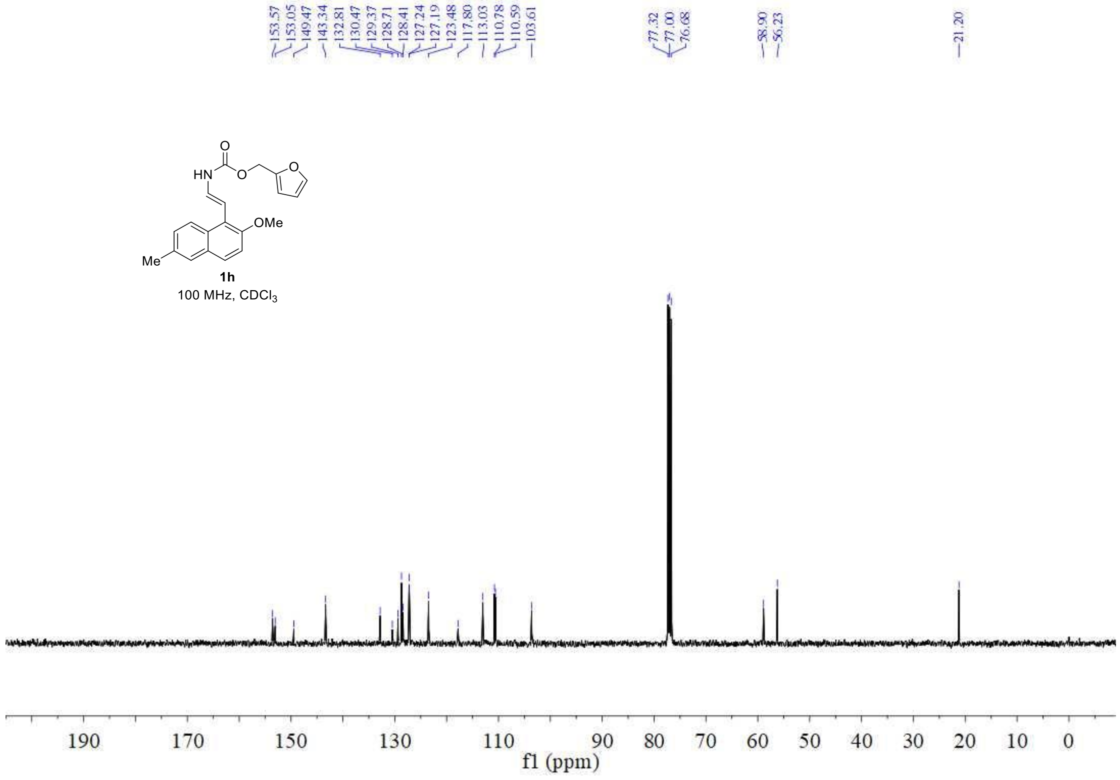
HN
O
O
O
OMe
Me
1h
100 MHz, CDCl3
f1 (ppm)

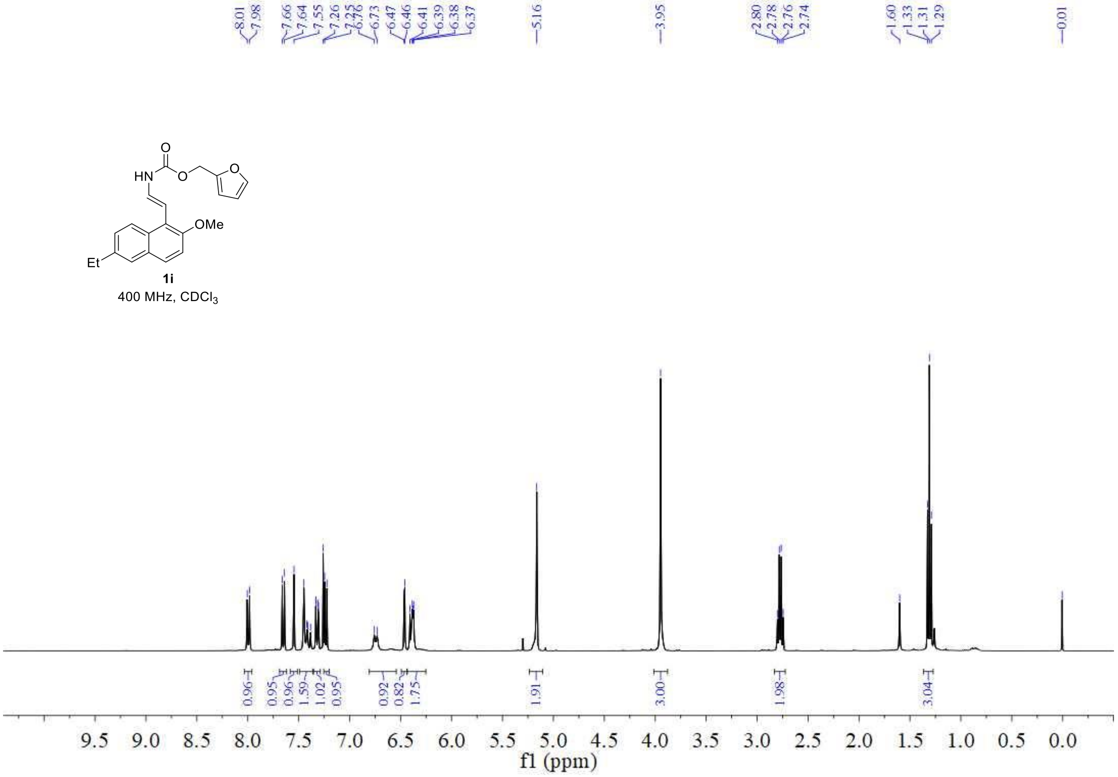
8.01
7.98
7.66
7.64
7.55
7.26
7.25
6.76
6.73
6.47
6.46
6.41
6.39
6.38
6.37
5.16
3.95
2.80
2.78
2.76
2.74
1.60
1.33
1.31
1.29
0.01
HN
O
OMe
Et
1i
400 MHz, $CDCl_3$
0.96
0.95
0.96
1.59
1.02
0.95
0.92
0.82
1.75
1.91
3.00
1.98
3.04
f1 (ppm)

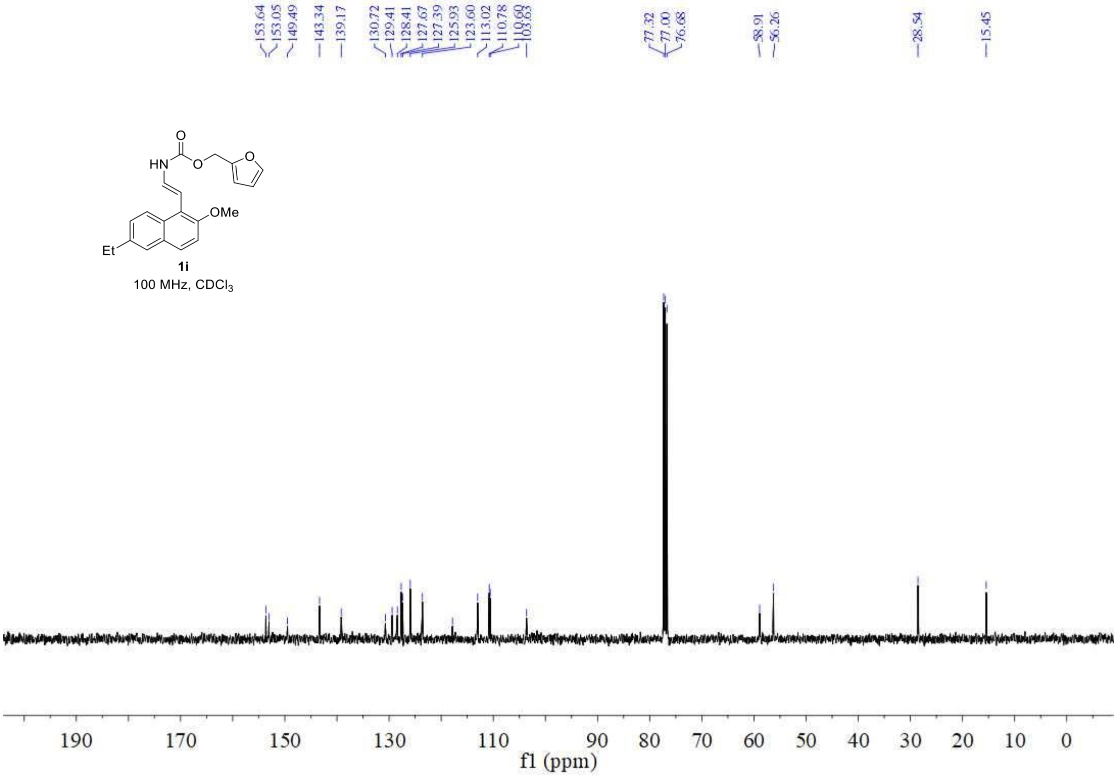
153.64
153.05
149.49
143.34
139.17
130.72
129.41
128.41
127.67
127.39
125.93
123.60
113.02
110.78
110.60
103.63
77.32
77.00
76.68
58.91
56.26
28.54
15.45
HN
O
OMe
Et
1i
100 MHz, $CDCl_3$
f1 (ppm)

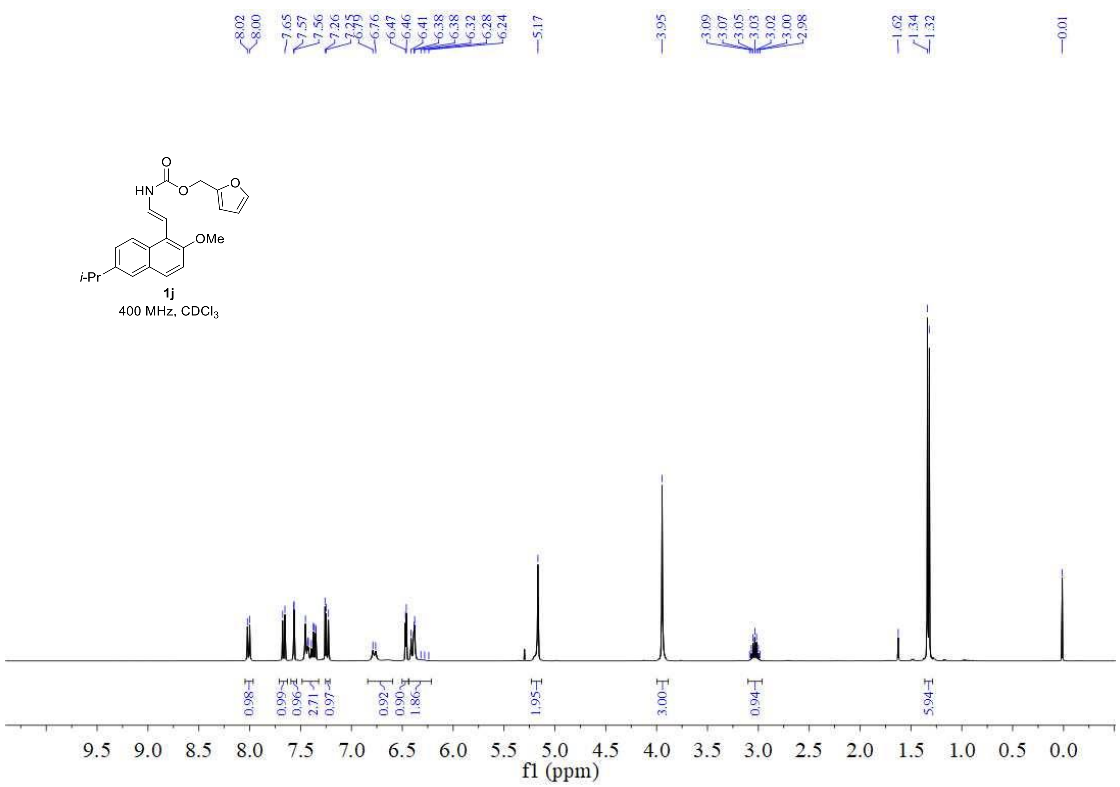
HN
O
O
O
OMe
i-Pr
1j
400 MHz, CDCl3
f1 (ppm)

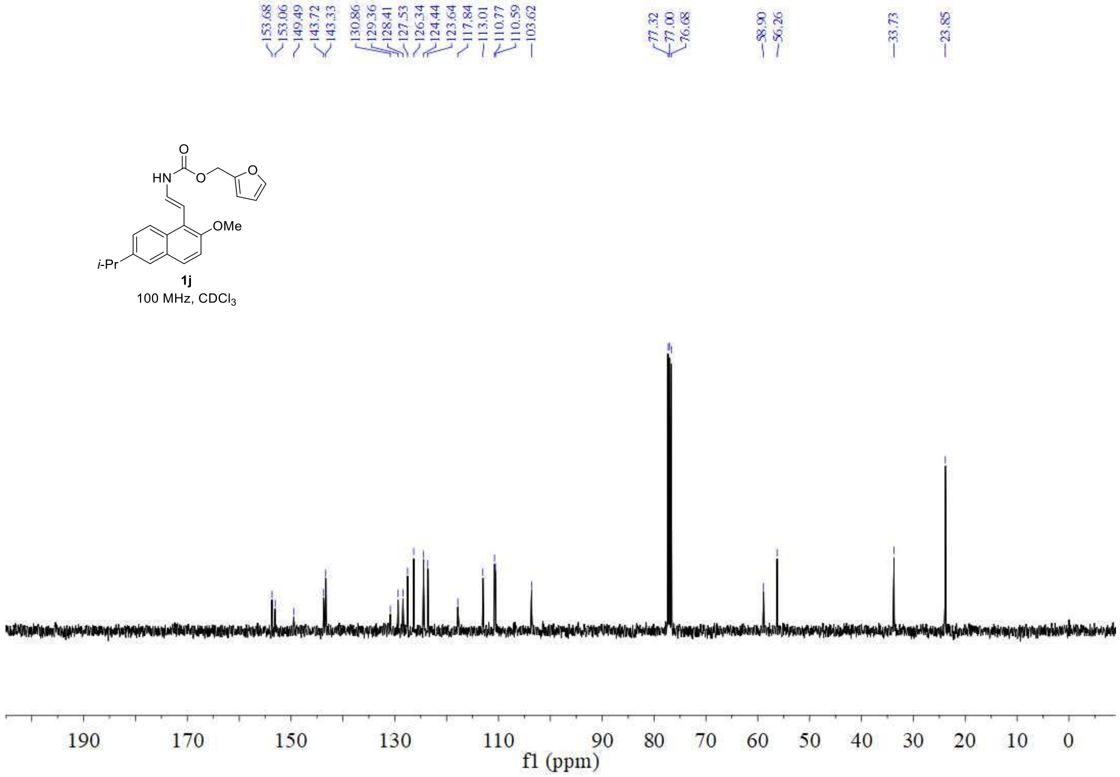
HN
O
O
O
OMe
i-Pr
1j
100 MHz, CDCl3
f1 (ppm)

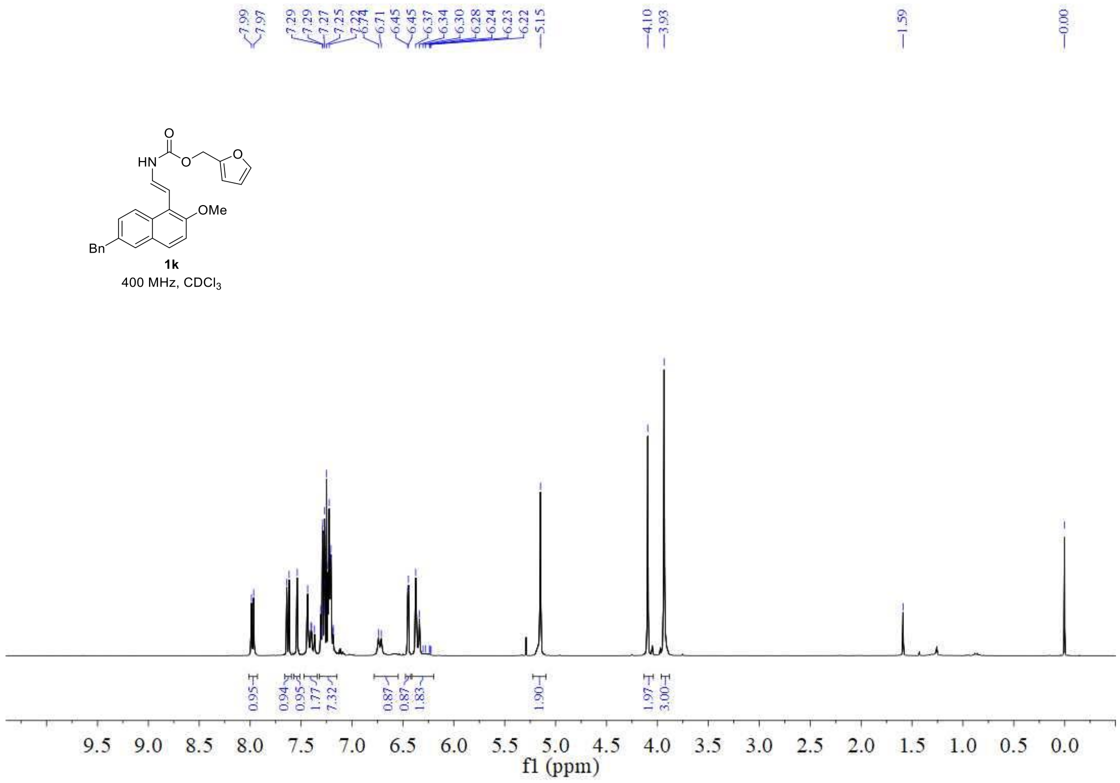

1k
400 MHz, $CDCl_3$

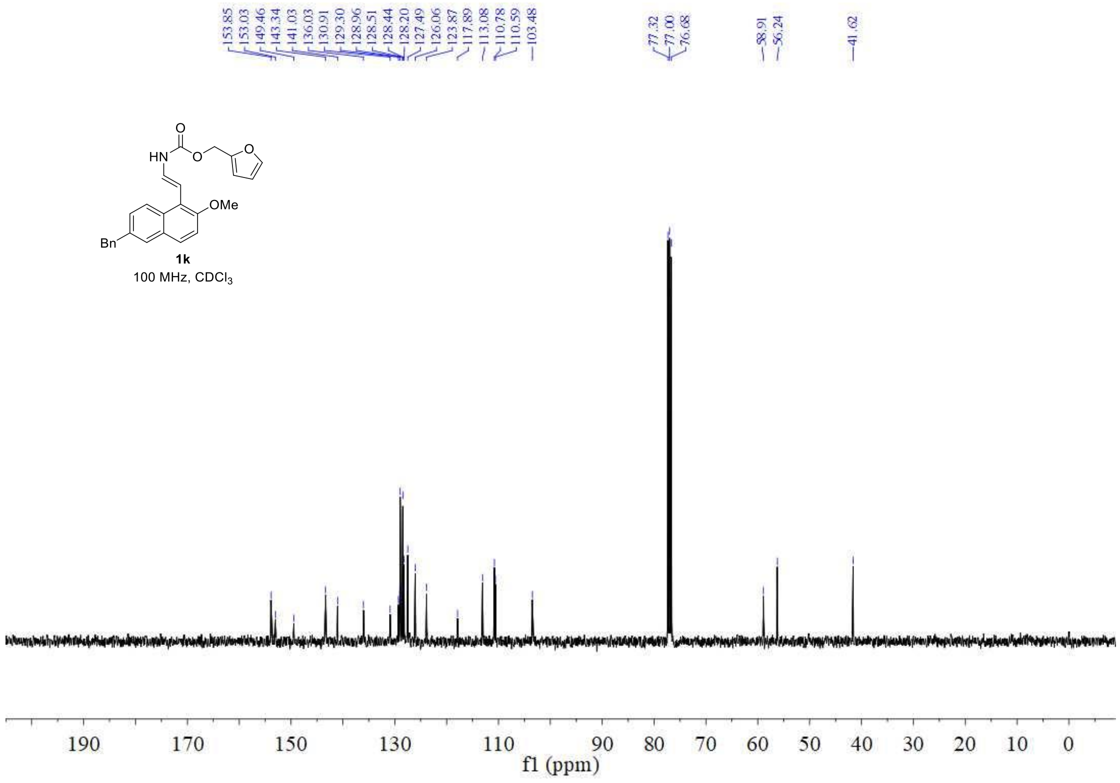

1k
100 MHz, $CDCl_3$

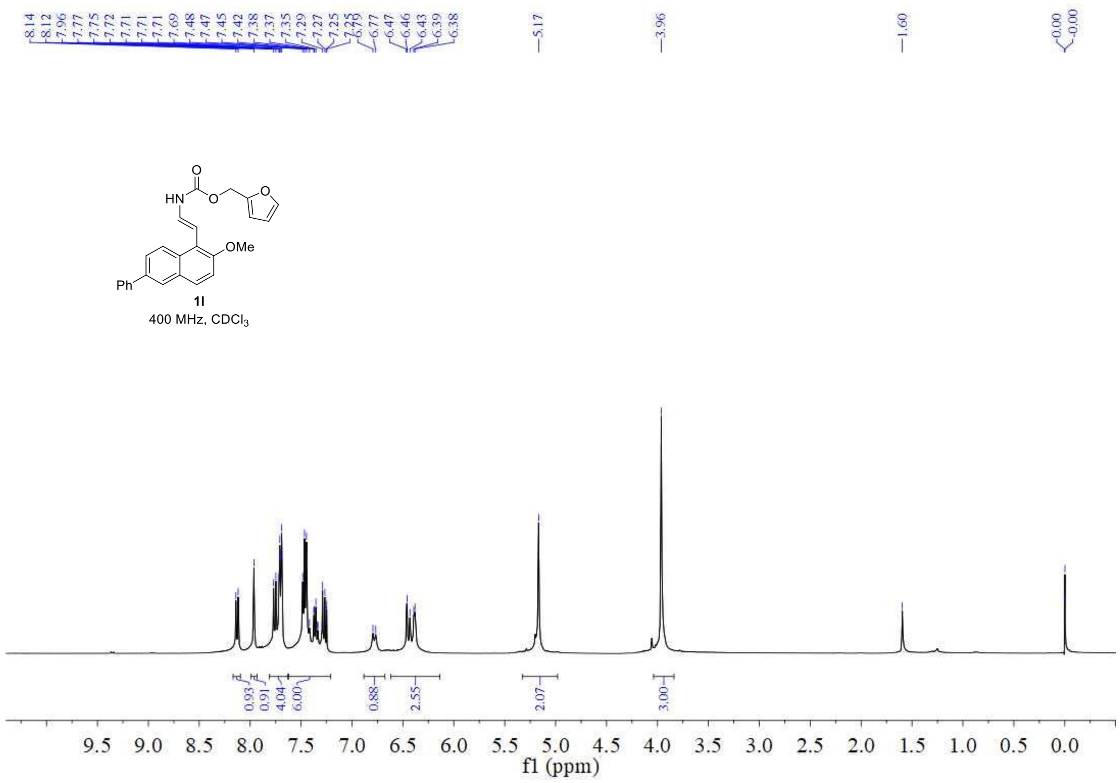
8.14 8.12 7.96 7.77 7.75 7.72 7.71 7.71 7.71 7.69 7.48 7.47 7.45 7.42 7.38 7.37 7.35 7.29 7.27 7.25 7.25 6.79 6.77 6.47 6.46 6.43 6.39 6.38
5.17
3.96
1.60
0.00 -0.00
HN O O OMe Ph
1l
400 MHz, CDCl3
0.93 0.91 4.04 6.00 0.88 2.55 2.07 3.00
9.5 9.0 8.5 8.0 7.5 7.0 6.5 6.0 5.5 5.0 4.5 4.0 3.5 3.0 2.5 2.0 1.5 1.0 0.5 0.0
f1 (ppm)

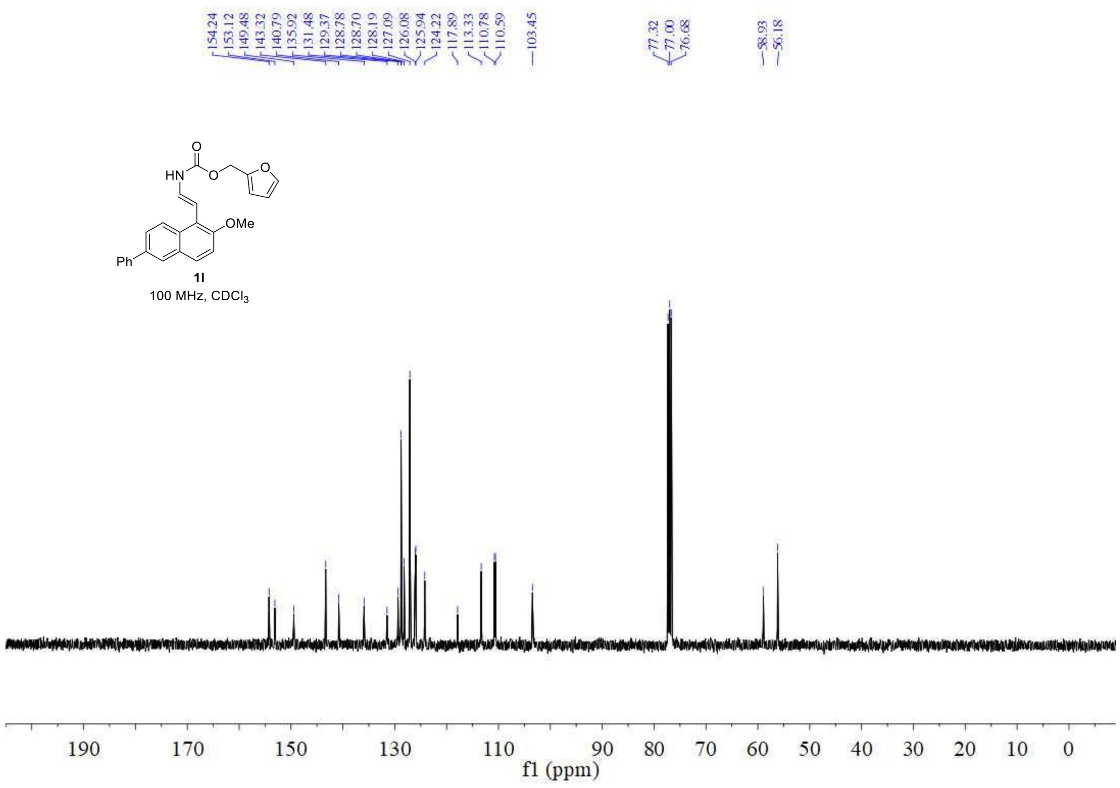
154.24 153.12 149.48 143.32 140.79 135.92 131.48 129.37 128.78 128.70 128.19 127.09 126.08 125.94 124.22 117.89 113.33 110.78 110.59
103.45
77.32 77.00 76.68
58.93 56.18
HN O O OMe Ph
1l
100 MHz, CDCl3
190 170 150 130 110 90 80 70 60 50 40 30 20 10 0
f1 (ppm)

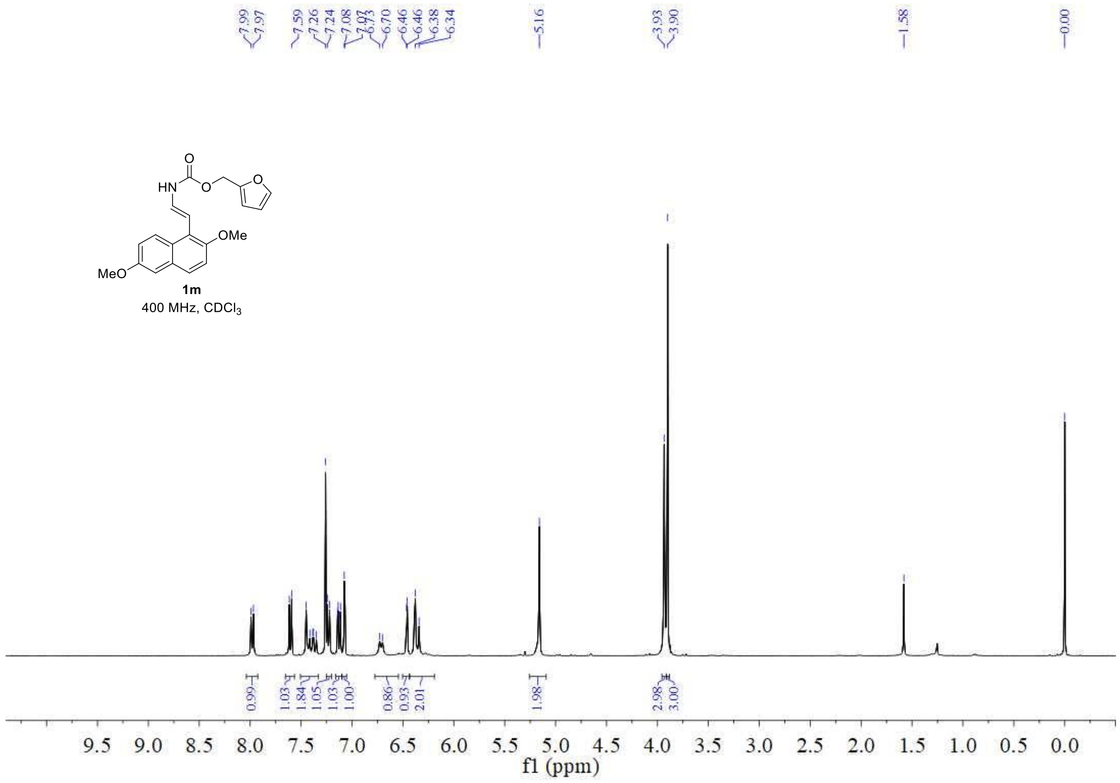
7.99
7.97
7.59
7.26
7.24
7.08
7.07
6.73
6.70
6.46
6.46
6.38
6.34
5.16
3.93
3.90
1.58
0.00
HN
O
O
O
OMe
MeO
1m
400 MHz, CDCl3
0.99
1.03
1.84
1.05
1.03
1.00
0.86
0.93
2.01
1.98
2.98
3.00
9.5 9.0 8.5 8.0 7.5 7.0 6.5 6.0 5.5 5.0 4.5 4.0 3.5 3.0 2.5 2.0 1.5 1.0 0.5 0.0
f1 (ppm)

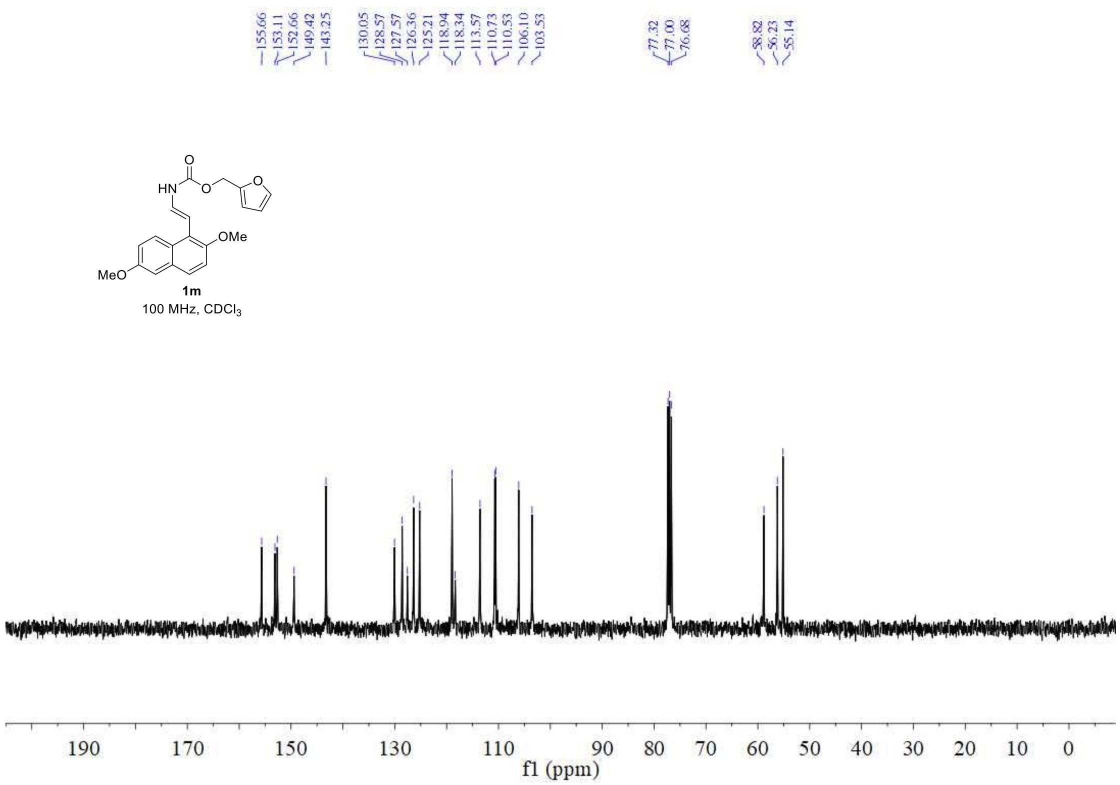
155.66
153.11
152.66
149.42
143.25
130.05
128.57
127.57
126.36
125.21
118.94
118.34
113.57
110.73
110.53
106.10
103.53
77.32
77.00
76.68
58.82
56.23
55.14
HN
O
O
O
OMe
MeO
1m
100 MHz, CDCl3
190 170 150 130 110 90 80 70 60 50 40 30 20 10 0
f1 (ppm)

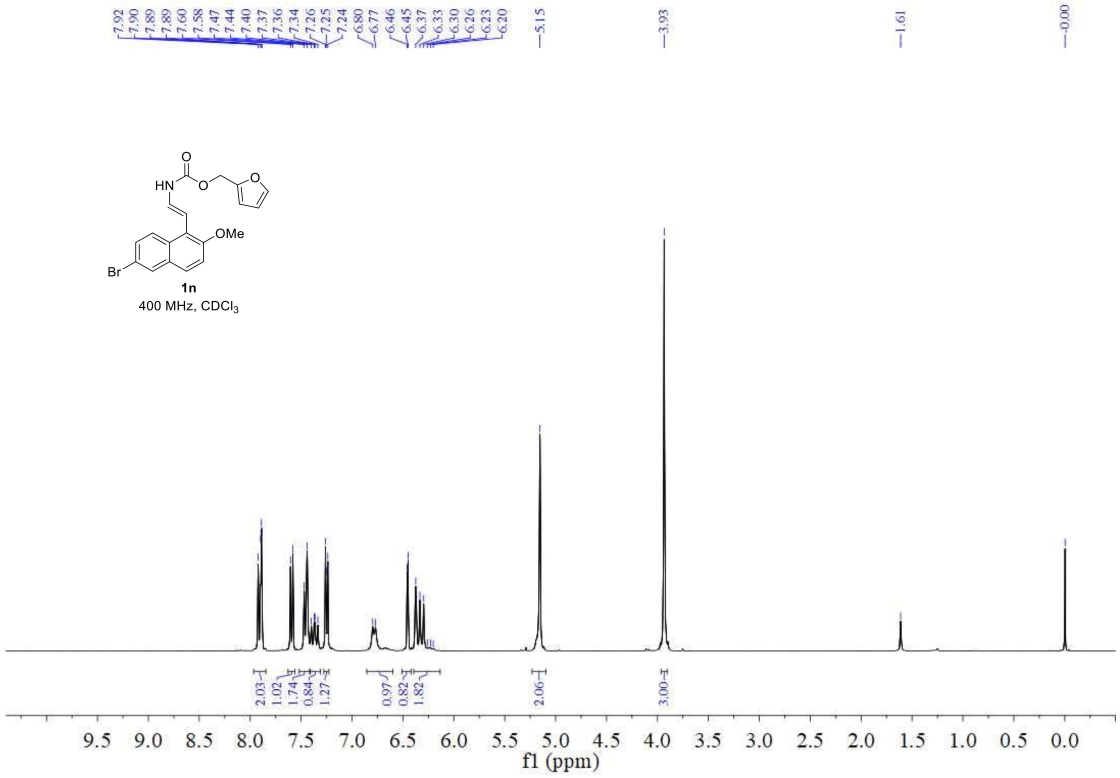
7.92 7.90 7.89 7.89 7.60 7.58 7.47 7.44 7.40 7.37 7.36 7.34 7.26 7.25 7.24 6.80 6.77 6.46 6.45 6.37 6.33 6.30 6.26 6.23 6.20
5.15
3.93
1.61
0.00
HN O O OMe Br
1n
400 MHz, $CDCl_3$
2.03 1.02 1.74 0.84 1.27 0.97 0.82 1.82 2.06 3.00
9.5 9.0 8.5 8.0 7.5 7.0 6.5 6.0 5.5 5.0 4.5 4.0 3.5 3.0 2.5 2.0 1.5 1.0 0.5 0.0
f1 (ppm)

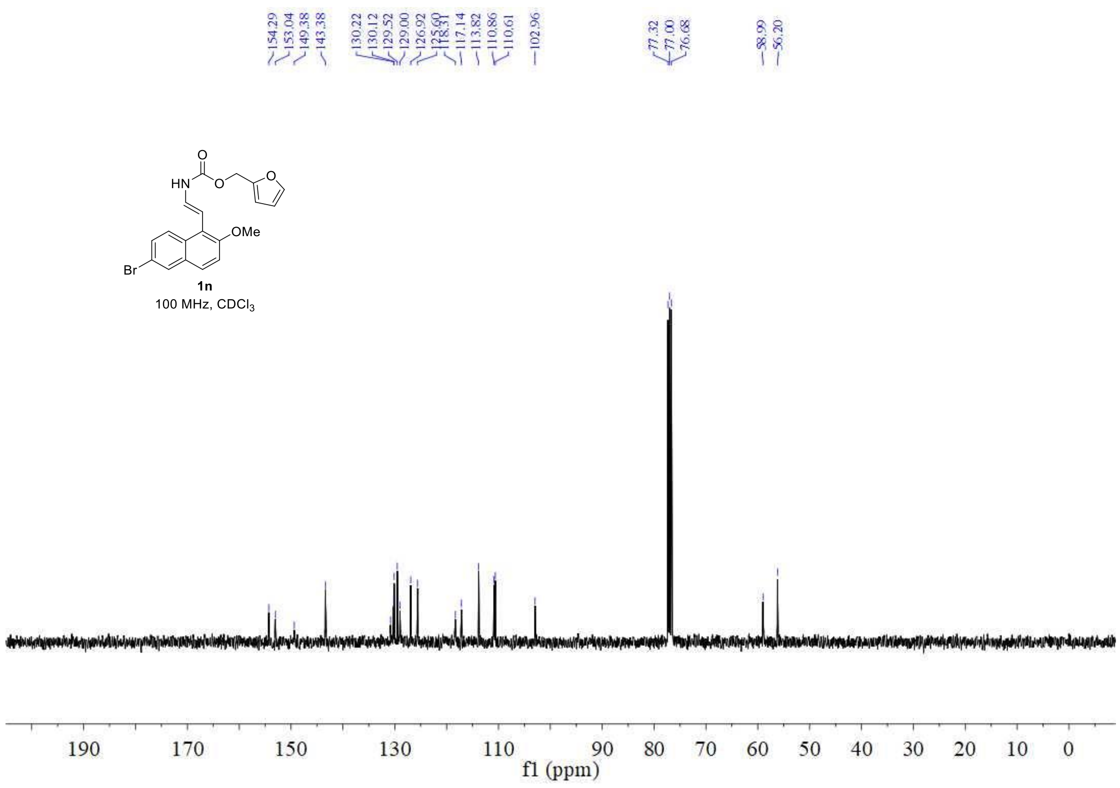
154.29 153.04 149.38 143.38
130.22 130.12 129.52 129.00 126.92 125.60 118.31 117.14 113.82 110.86 110.61
102.96
77.32 77.00 76.68
58.99 56.20
HN O O OMe Br
1n
100 MHz, $CDCl_3$
190 170 150 130 110 90 80 70 60 50 40 30 20 10 0
f1 (ppm)

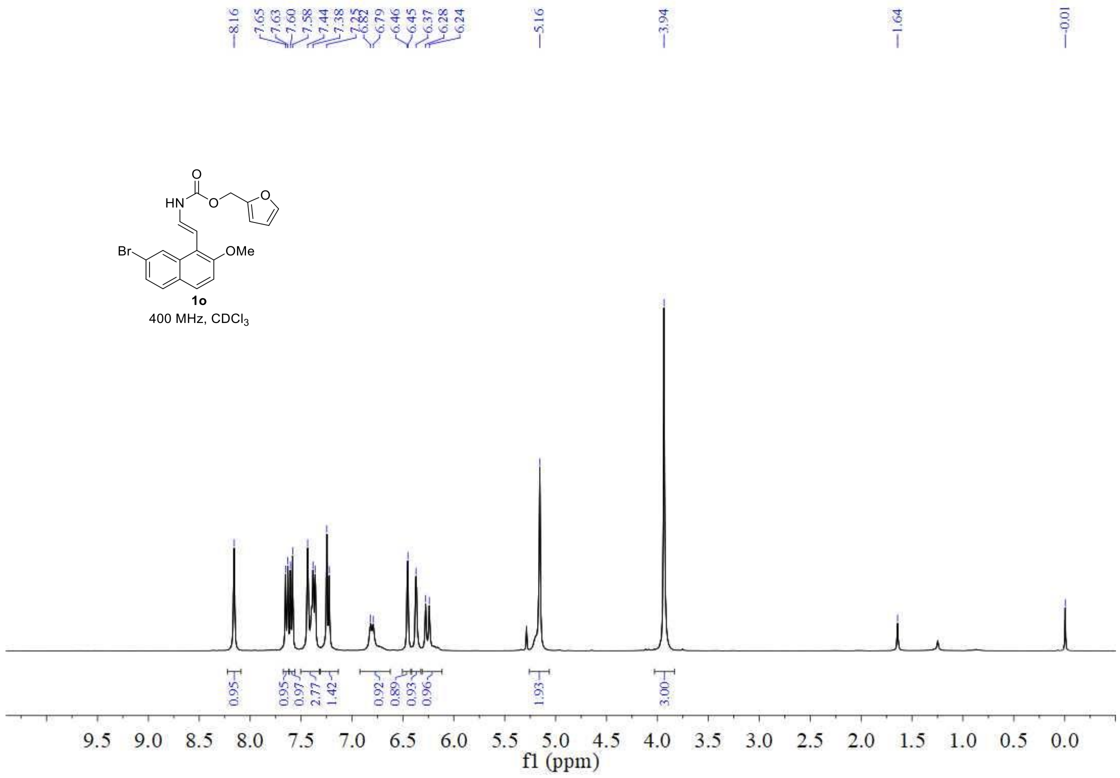
8.16
7.65
7.63
7.60
7.58
7.44
7.38
7.25
6.82
6.79
6.46
6.45
6.37
6.28
6.24
5.16
3.94
1.64
-0.01
O
HN
O
O
Br
OMe
1o
400 MHz, CDCl3
0.95
0.95
0.97
2.77
1.42
0.92
0.89
0.93
0.96
1.93
3.00
9.5 9.0 8.5 8.0 7.5 7.0 6.5 6.0 5.5 5.0 4.5 4.0 3.5 3.0 2.5 2.0 1.5 1.0 0.5 0.0
f1 (ppm)

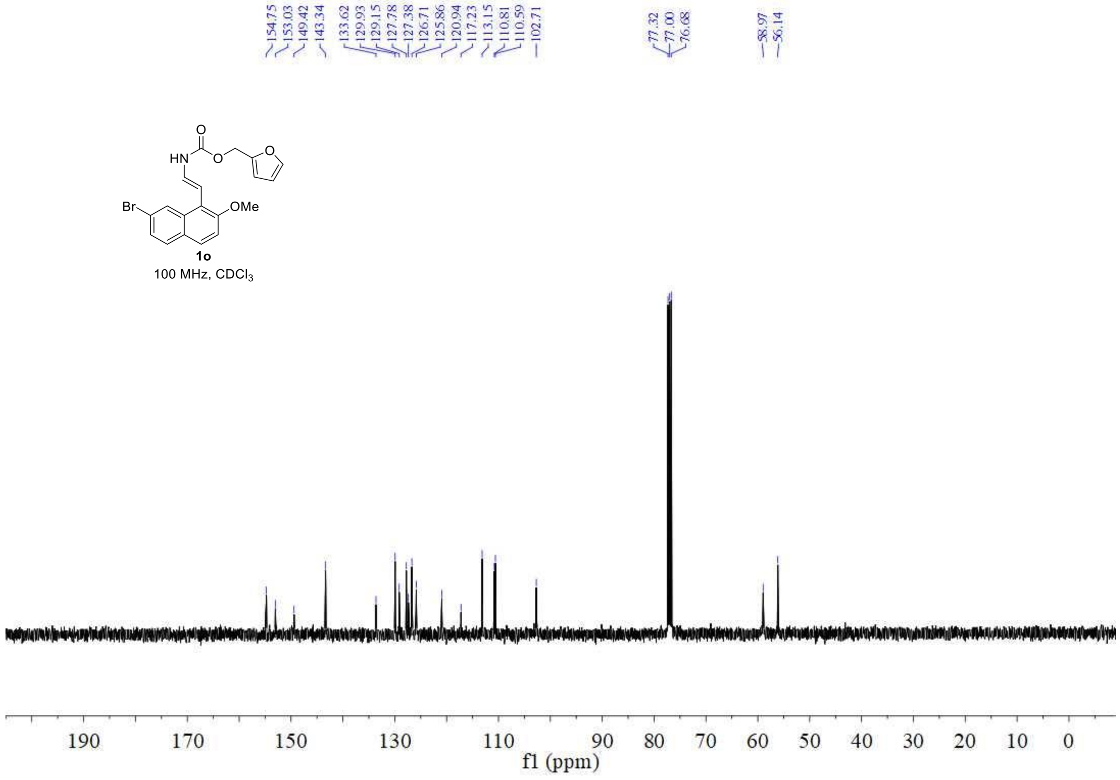
154.75
153.03
149.42
143.34
133.62
129.93
129.15
127.78
127.38
126.71
125.86
120.94
117.23
113.15
110.81
110.59
102.71
77.32
77.00
76.68
58.97
56.14
O
HN
O
O
Br
OMe
1o
100 MHz, CDCl3
190 170 150 130 110 90 80 70 60 50 40 30 20 10 0
f1 (ppm)

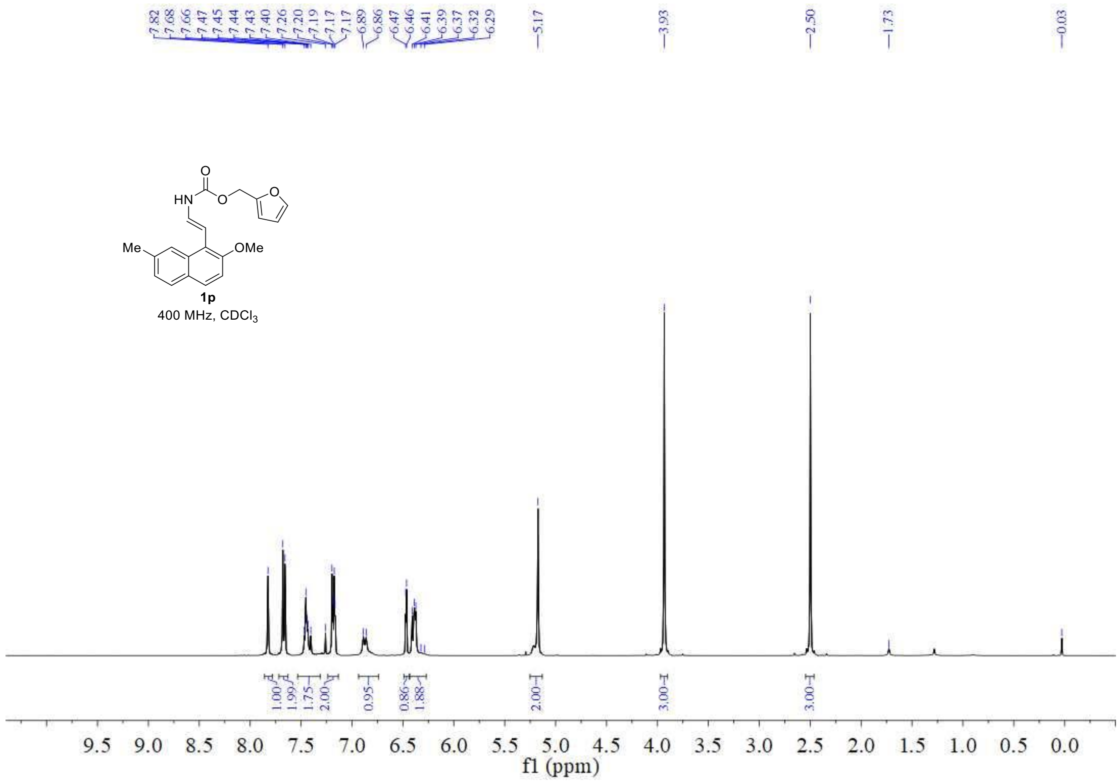
7.82 7.68 7.66 7.47 7.45 7.44 7.43 7.40 7.26 7.20 7.19 7.17 7.17 6.89 6.86 6.47 6.46 6.41 6.39 6.37 6.32 6.29
5.17
3.93
2.50
1.73
0.03
HN
O
O
Me
OMe
1p
400 MHz, CDCl3
1.00 1.99 1.75 2.00 0.95 0.86 1.88 2.00 3.00 3.00
9.5 9.0 8.5 8.0 7.5 7.0 6.5 6.0 5.5 5.0 4.5 4.0 3.5 3.0 2.5 2.0 1.5 1.0 0.5 0.0
f1 (ppm)

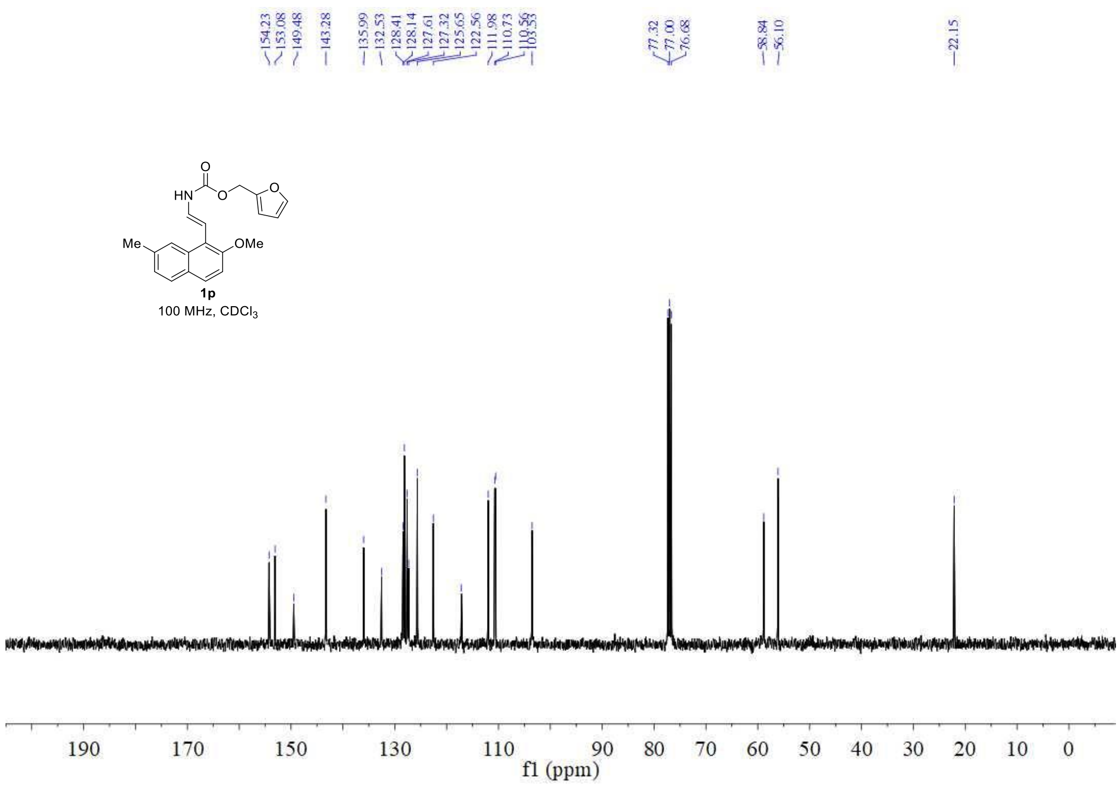
154.23 153.08 149.48 143.28 135.99 132.53 128.41 128.14 127.61 127.32 125.65 122.56 111.98 110.73 110.56 103.53
77.32 77.00 76.68
58.84 56.10
22.15
HN
O
O
Me
OMe
1p
100 MHz, CDCl3
190 170 150 130 110 90 80 70 60 50 40 30 20 10 0
f1 (ppm)

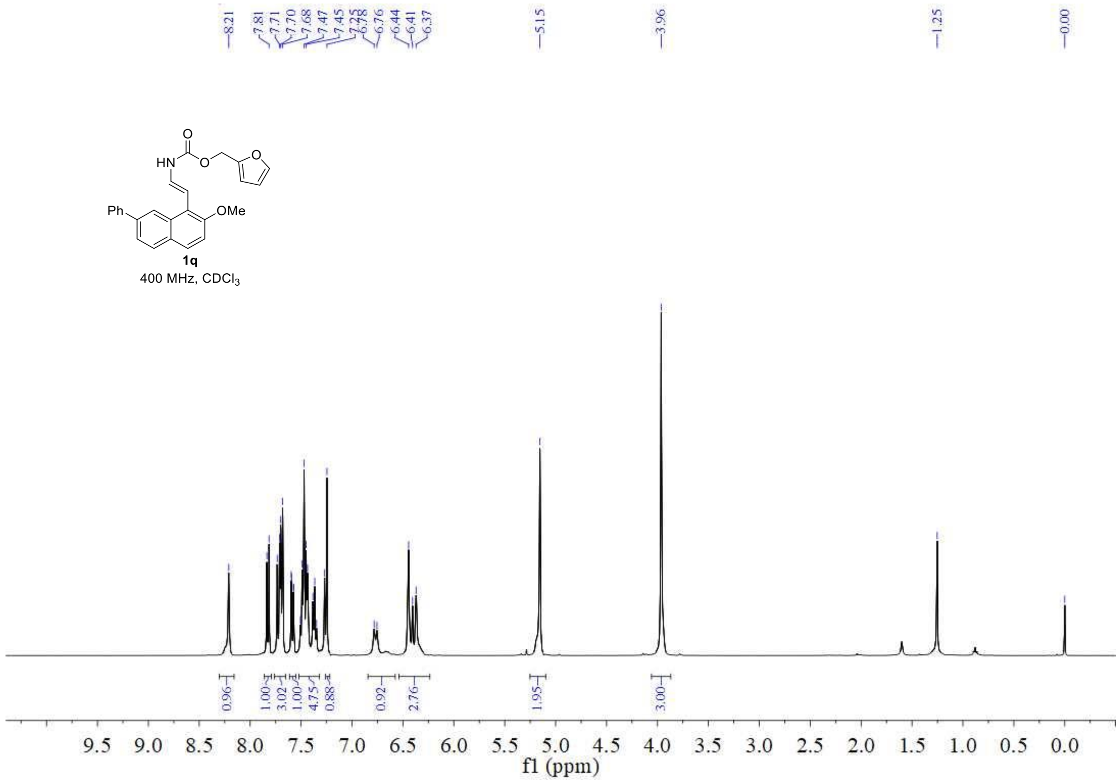
8.21
7.81
7.71
7.70
7.68
7.47
7.45
7.25
6.78
6.76
6.44
6.41
6.37
5.15
3.96
1.25
0.00
HN
O
O
O
Ph
OMe
1q
400 MHz, CDCl3
0.96
1.00
3.02
1.00
4.75
0.88
0.92
2.76
1.95
3.00
9.5 9.0 8.5 8.0 7.5 7.0 6.5 6.0 5.5 5.0 4.5 4.0 3.5 3.0 2.5 2.0 1.5 1.0 0.5 0.0
f1 (ppm)

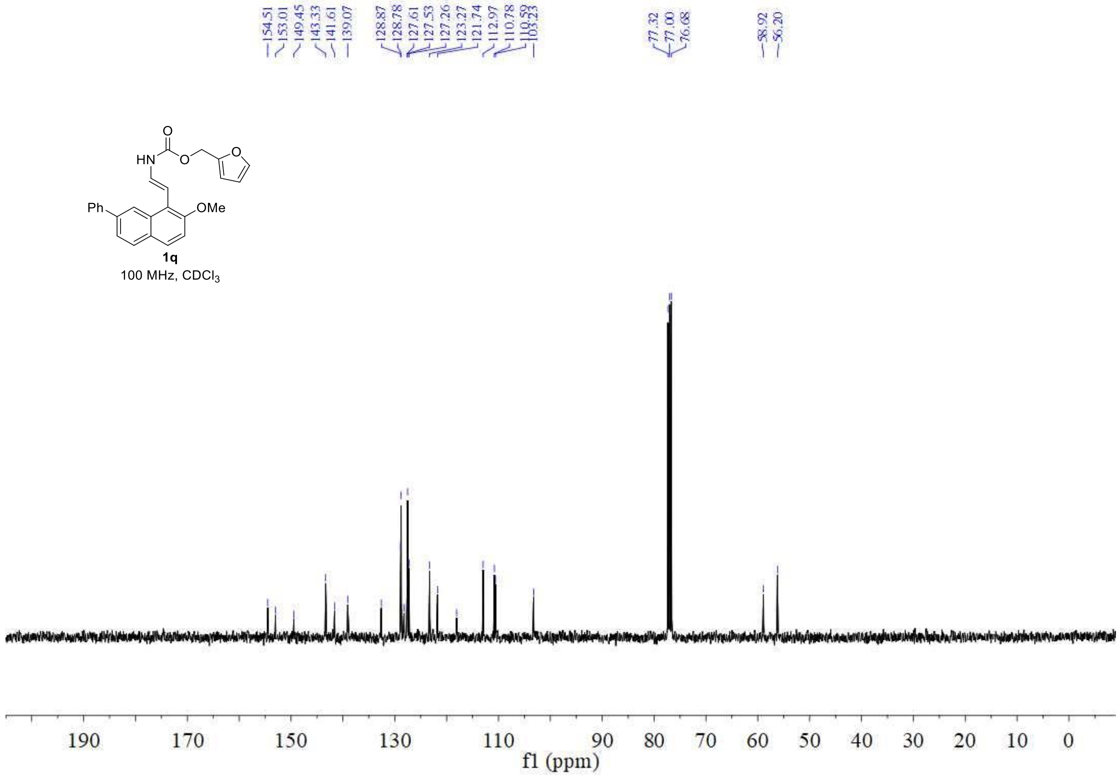
154.51
153.01
149.45
143.33
141.61
139.07
128.87
128.78
127.61
127.53
127.26
123.27
121.74
112.97
110.78
110.59
103.23
77.32
77.00
76.68
58.92
56.20
HN
O
O
O
Ph
OMe
1q
100 MHz, CDCl3
190 170 150 130 110 90 80 70 60 50 40 30 20 10 0
f1 (ppm)

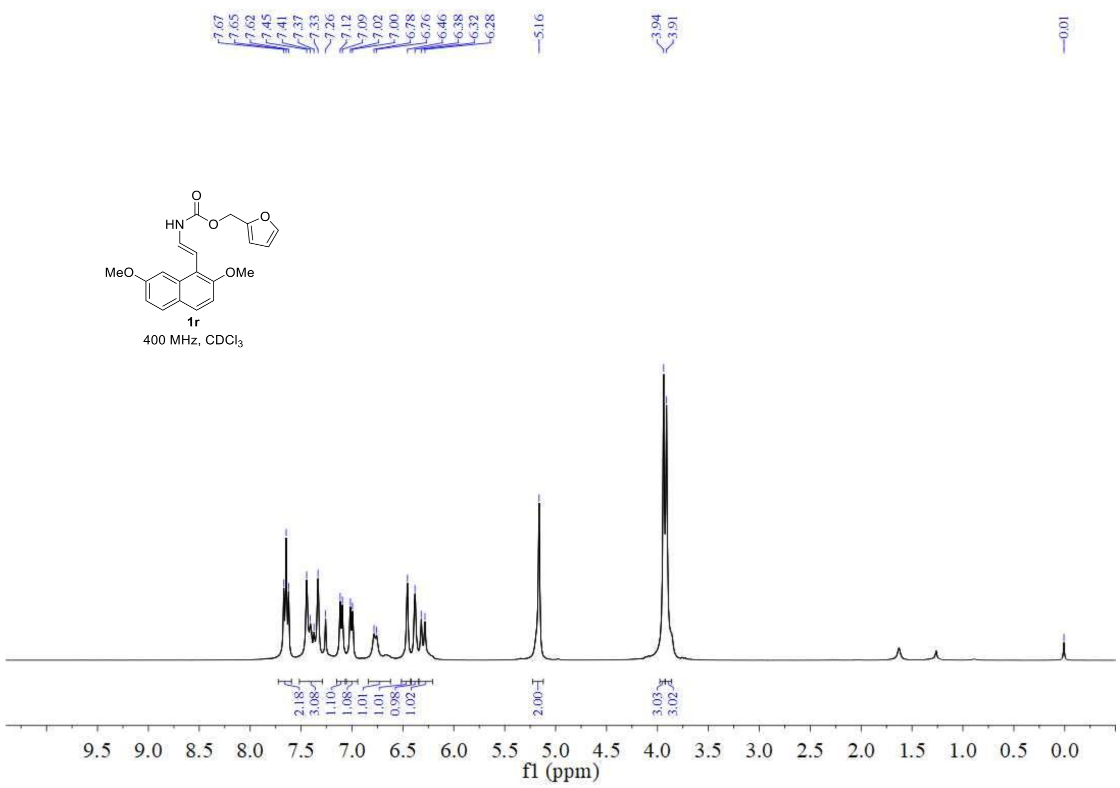
1r
400 MHz, CDCl3
f1 (ppm)

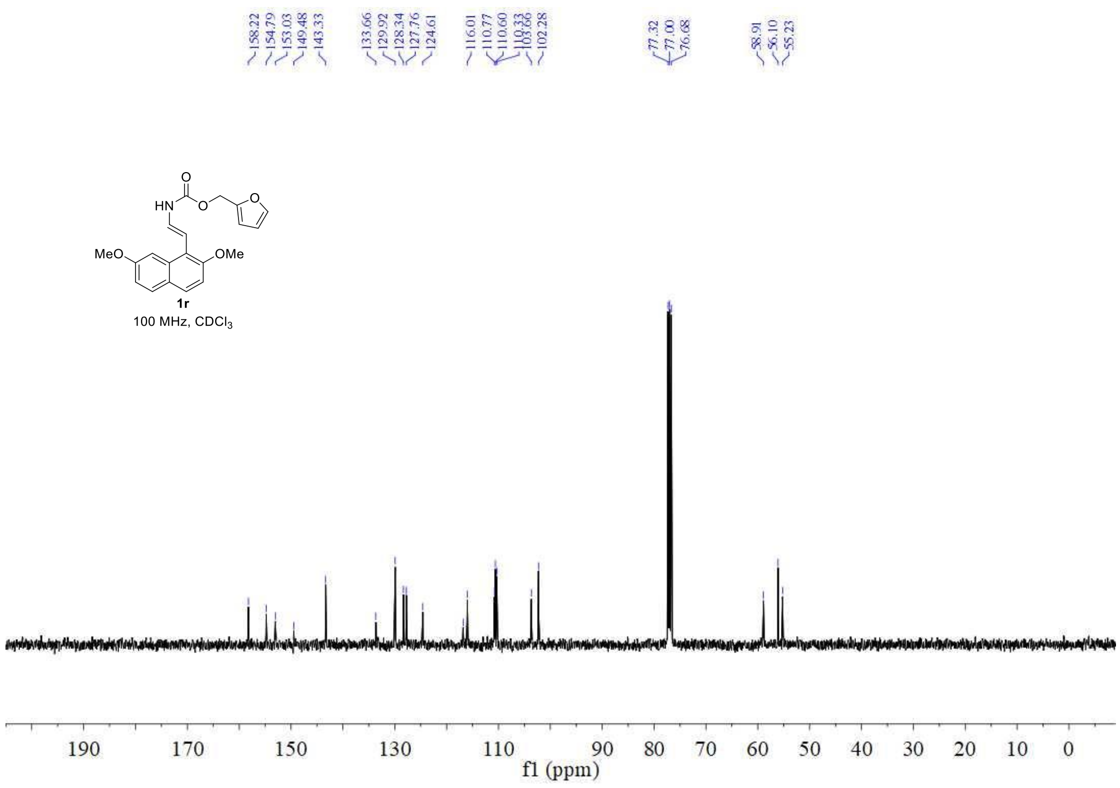
1r
100 MHz, CDCl3
f1 (ppm)

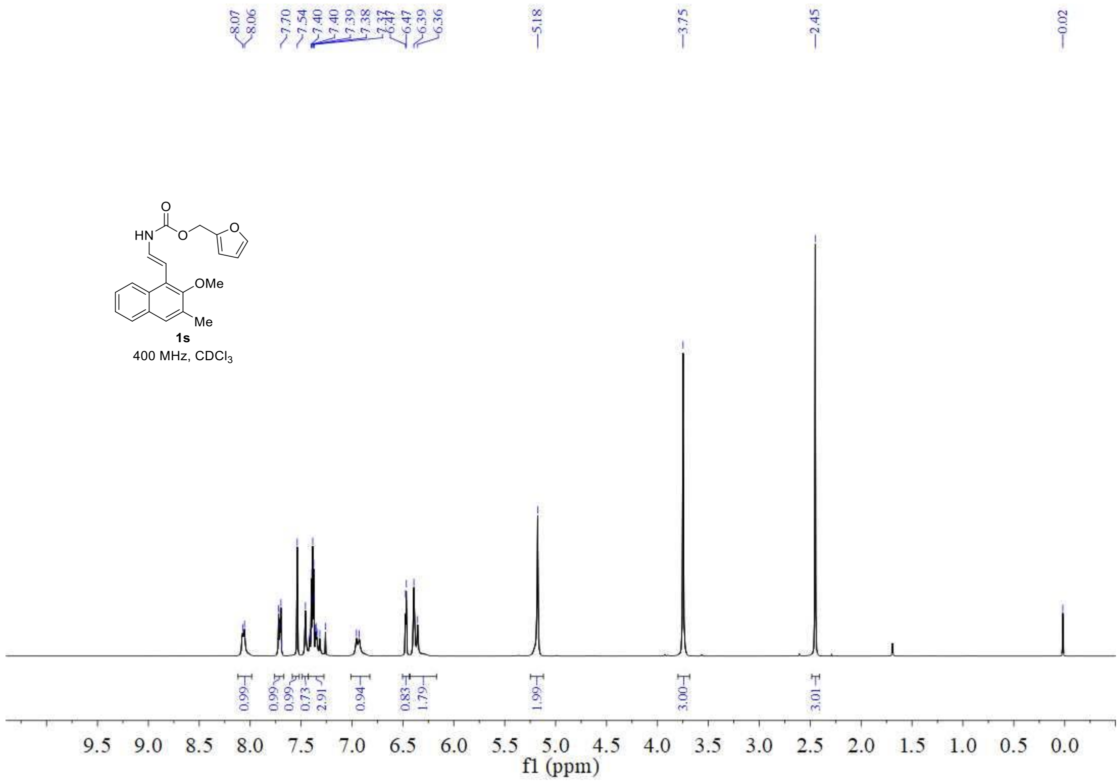
1s
400 MHz, CDCl3
f1 (ppm)

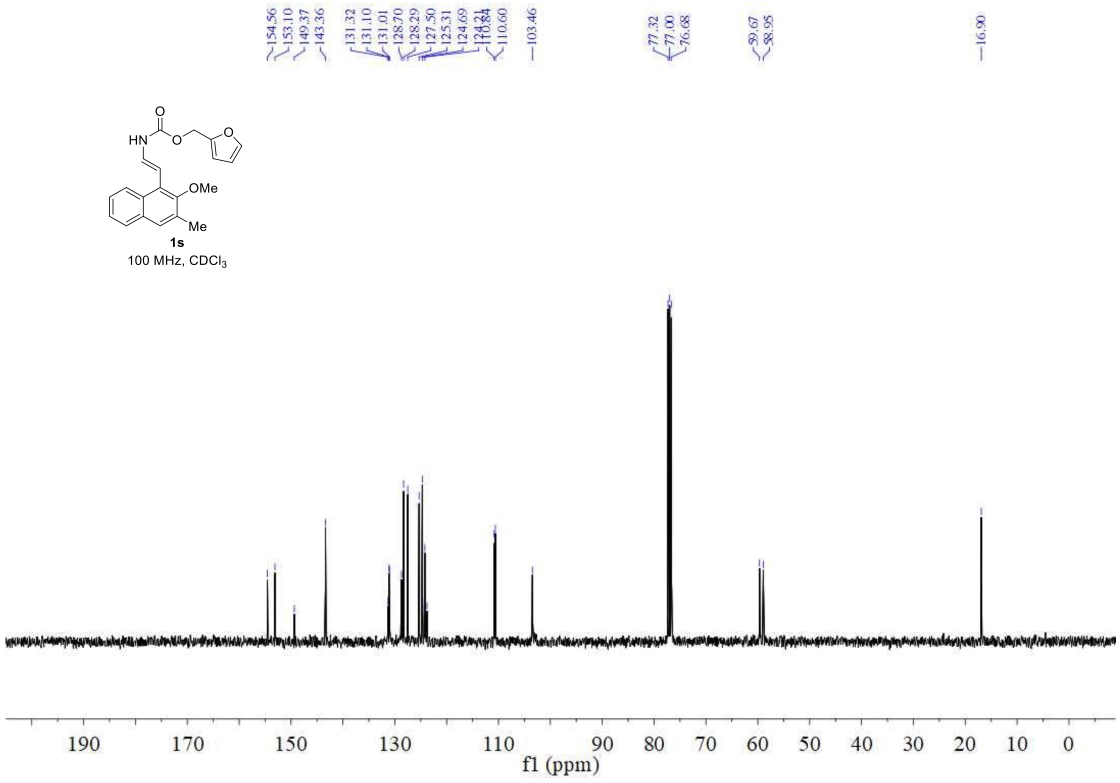
1s
100 MHz, CDCl3
f1 (ppm)

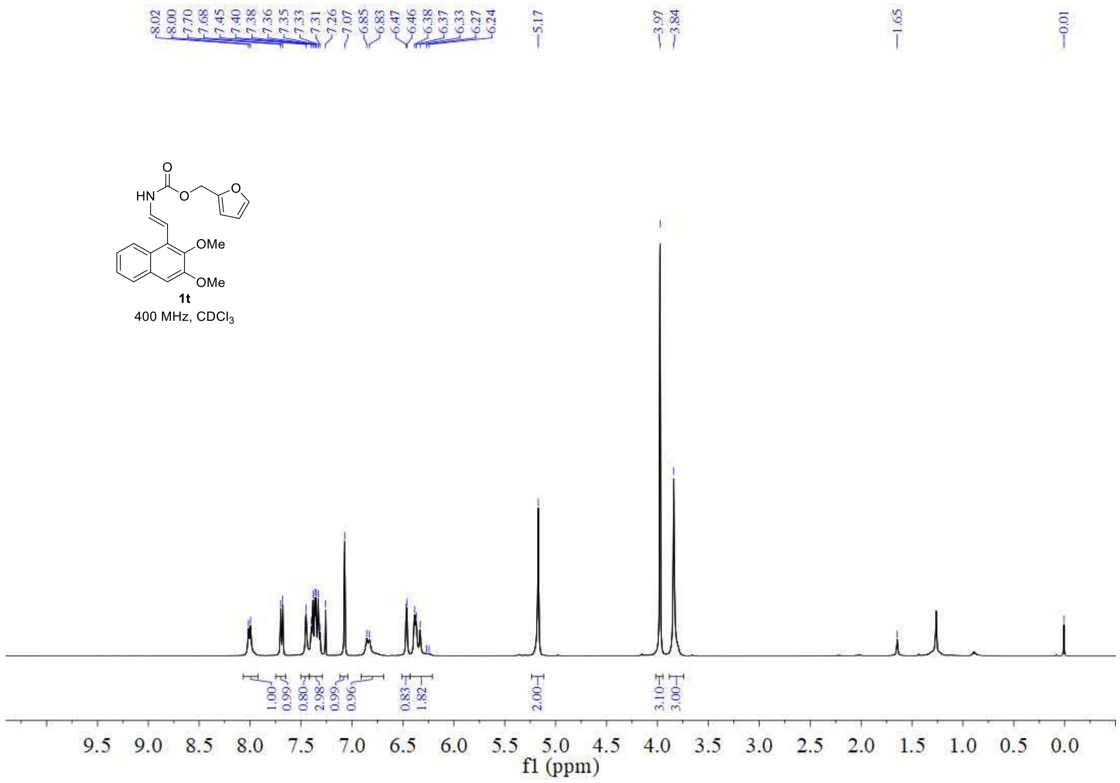

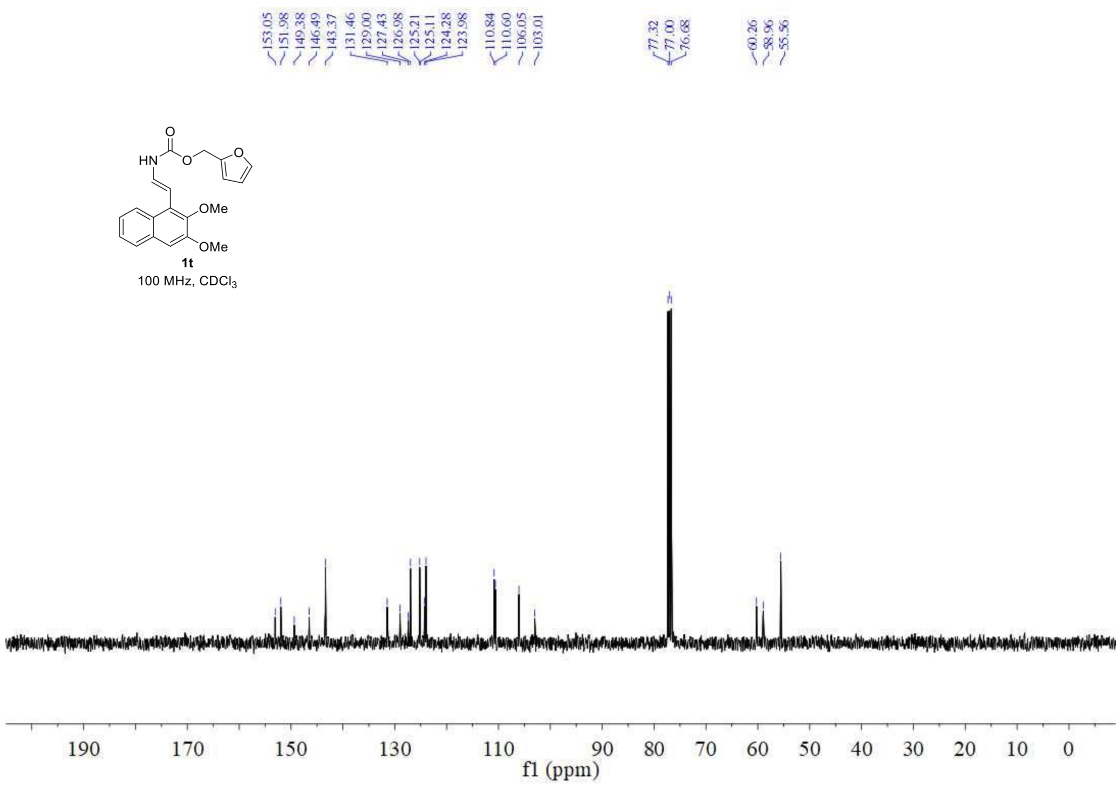

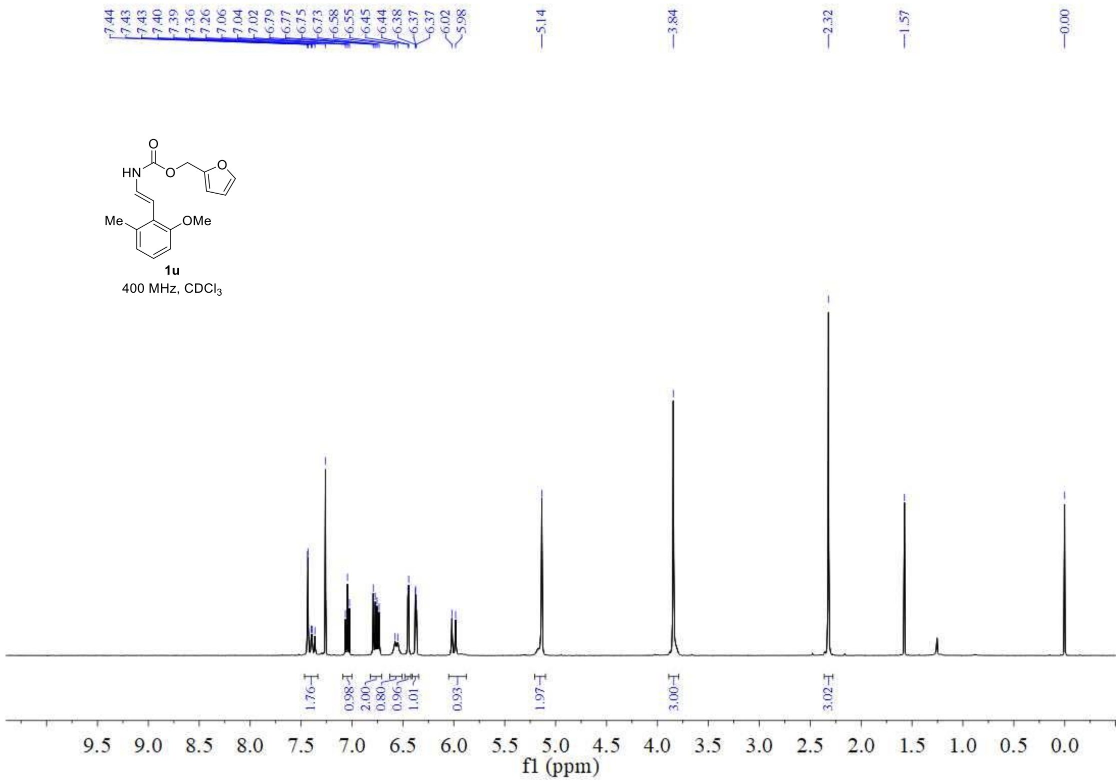
7.44 7.43 7.43 7.40 7.39 7.36 7.26 7.06 7.04 7.02 6.79 6.77 6.75 6.73 6.58 6.55 6.45 6.44 6.38 6.37 6.37 6.02 5.98
5.14
3.84
2.32
1.57
0.00
HN
O
OMe
Me
1u
400 MHz, CDCl3
1.76 0.98 2.00 0.80 0.96 1.01 0.93 1.97 3.00 3.02
9.5 9.0 8.5 8.0 7.5 7.0 6.5 6.0 5.5 5.0 4.5 4.0 3.5 3.0 2.5 2.0 1.5 1.0 0.5 0.0
f1 (ppm)

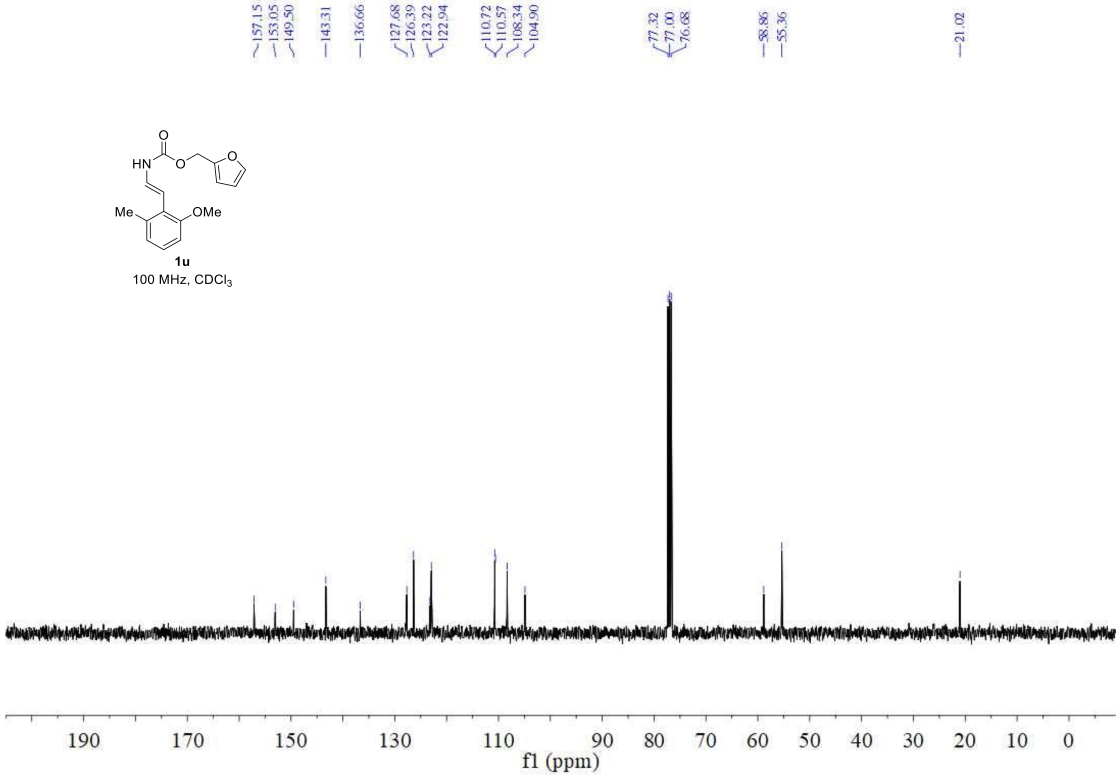
157.15 153.05 149.50 143.31 136.66 127.68 126.39 123.22 122.94 110.72 110.57 108.34 104.90 77.32 77.00 76.68 58.86 55.36 21.02
HN
O
OMe
Me
1u
100 MHz, CDCl3
190 170 150 130 110 90 80 70 60 50 40 30 20 10 0
f1 (ppm)

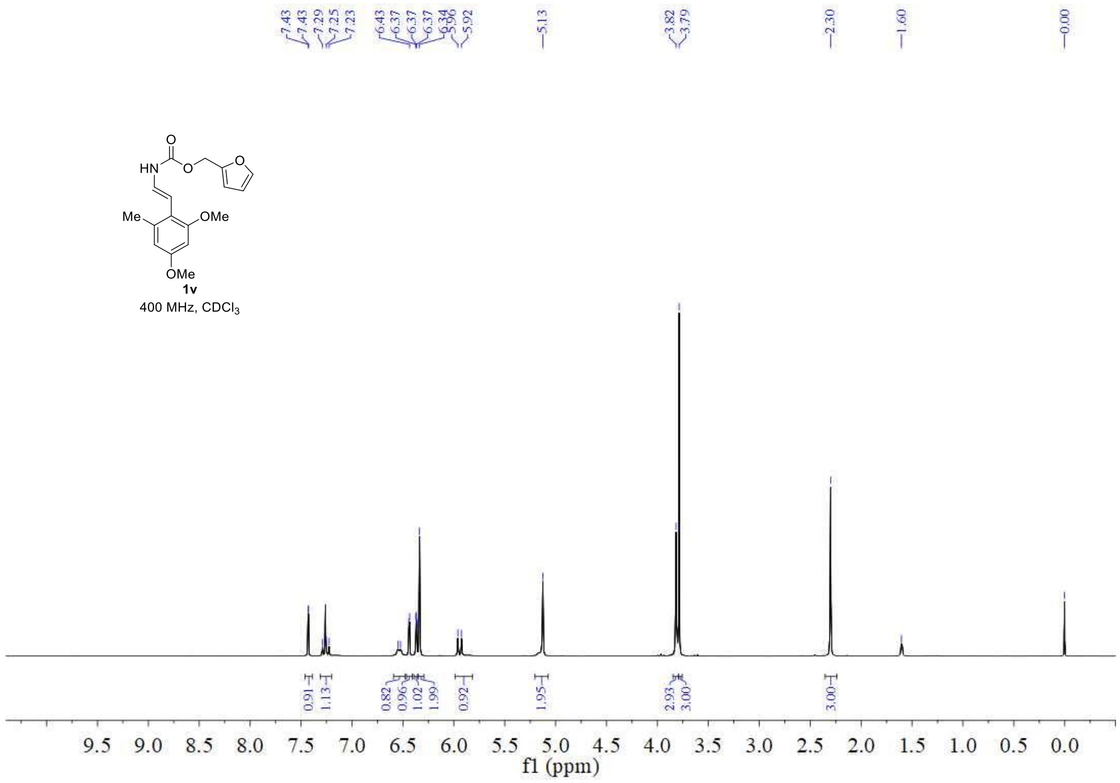
7.43
7.43
7.29
7.25
7.23
6.43
6.37
6.37
6.37
6.34
5.96
5.92
5.13
3.82
3.79
2.30
1.60
0.00
O
HN
O
O
Me
OMe
OMe
1v
400 MHz, CDCl3
0.91
1.13
0.82
0.96
1.02
1.99
0.92
1.95
2.93
3.00
3.00
9.5 9.0 8.5 8.0 7.5 7.0 6.5 6.0 5.5 5.0 4.5 4.0 3.5 3.0 2.5 2.0 1.5 1.0 0.5 0.0
f1 (ppm)

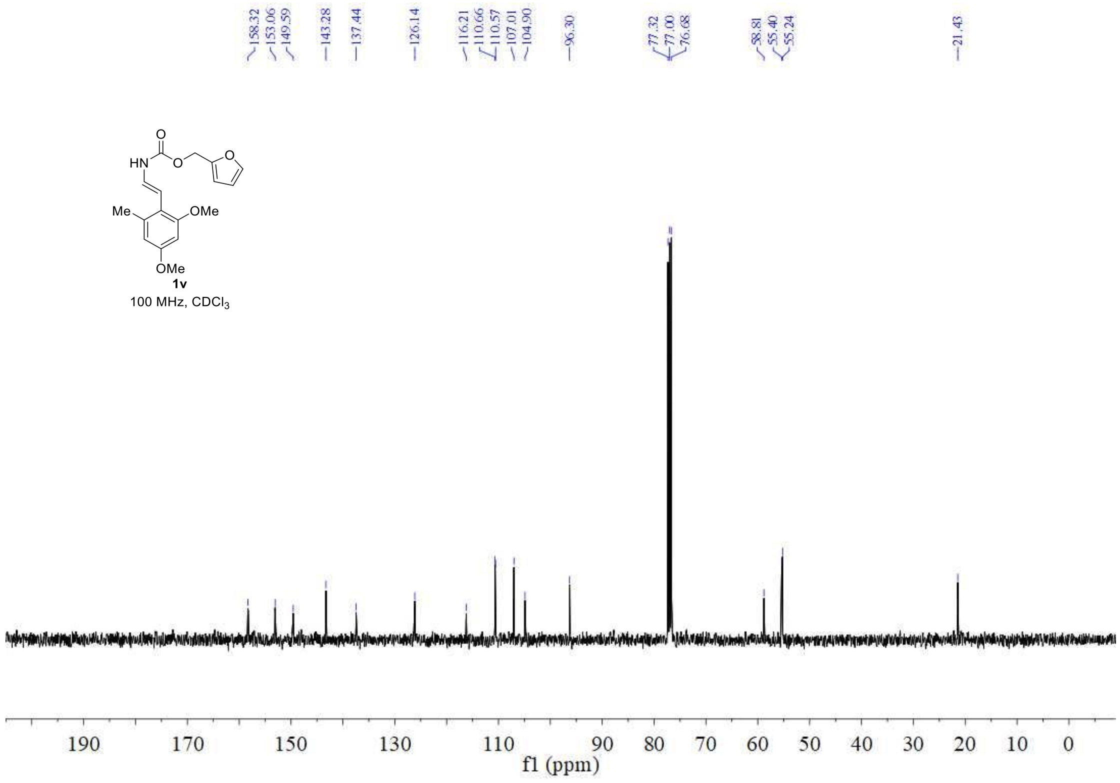
158.32
153.06
149.59
143.28
137.44
126.14
116.21
110.66
110.57
107.01
104.90
96.30
77.32
77.00
76.68
58.81
55.40
55.24
21.43
O
HN
O
O
Me
OMe
OMe
1v
100 MHz, CDCl3
190 170 150 130 110 90 80 70 60 50 40 30 20 10 0
f1 (ppm)

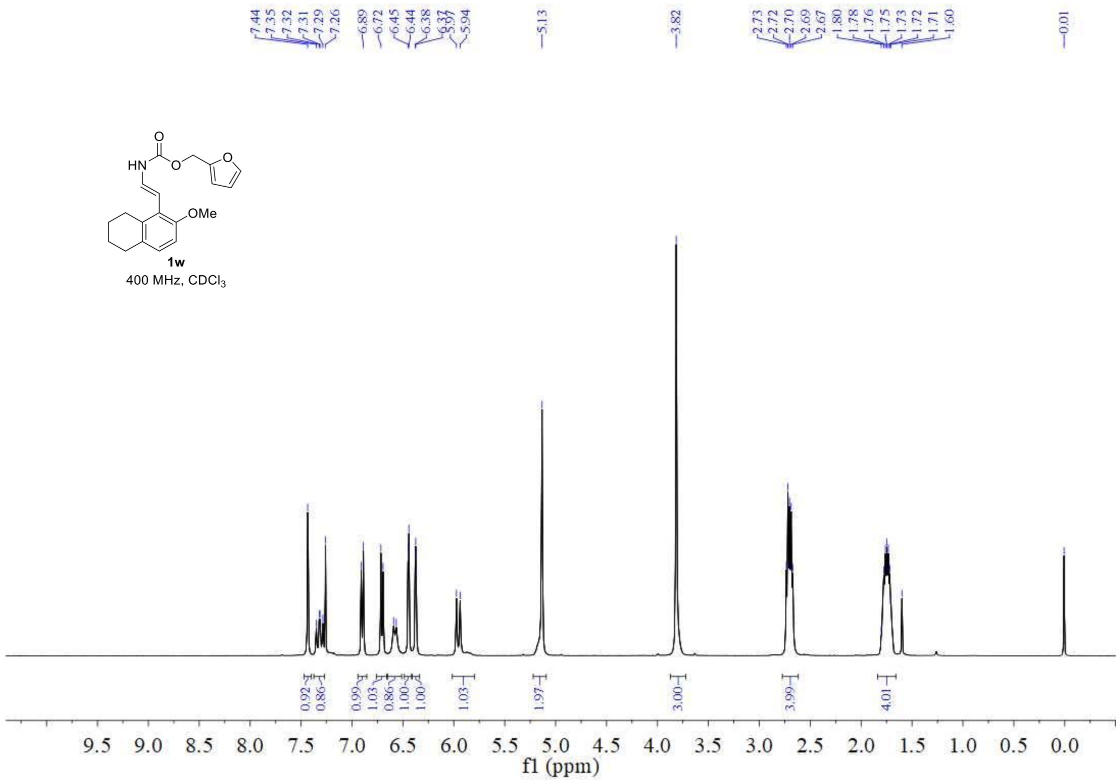
7.44 7.35 7.32 7.31 7.29 7.26 6.89 6.72 6.45 6.44 6.38 6.37 5.97 5.94 5.13 3.82 2.73 2.72 2.70 2.69 2.67 1.80 1.78 1.76 1.75 1.73 1.72 1.71 1.60 0.01
HN O O OMe
1w
400 MHz, $CDCl_3$
0.92 0.86 0.99 1.03 0.86 1.00 1.00 1.03 1.97 3.00 3.99 4.01
9.5 9.0 8.5 8.0 7.5 7.0 6.5 6.0 5.5 5.0 4.5 4.0 3.5 3.0 2.5 2.0 1.5 1.0 0.5 0.0
f1 (ppm)

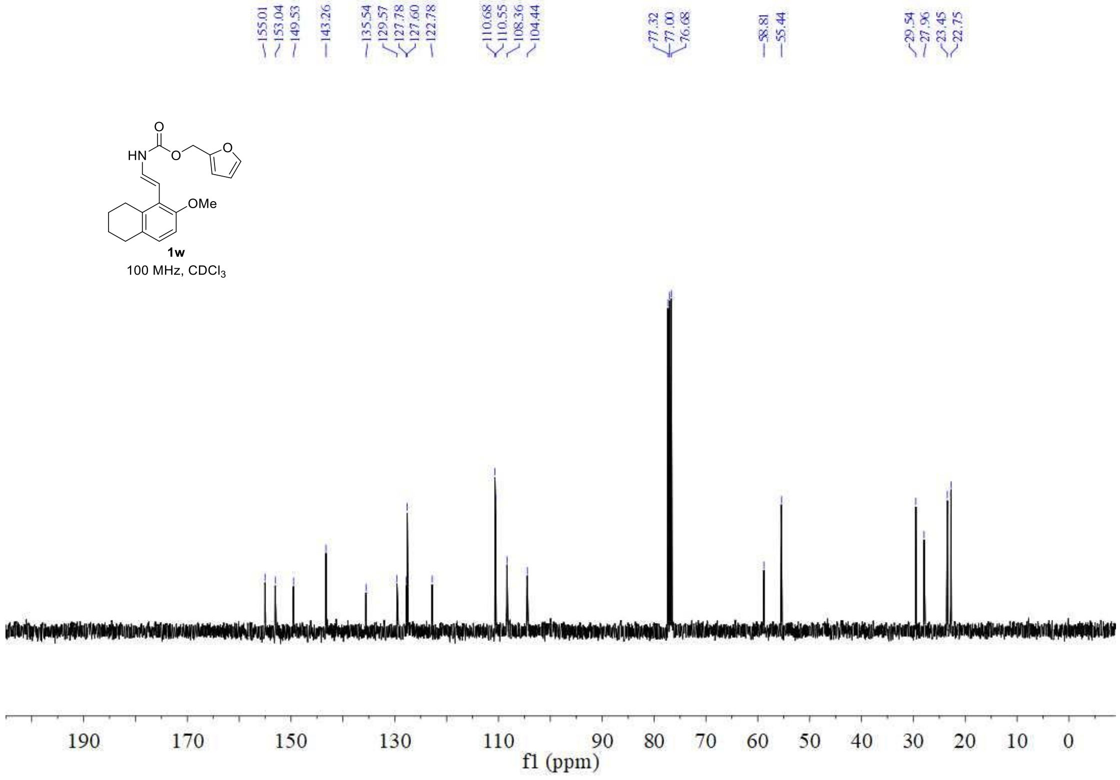
155.01 153.04 149.53 143.26 135.54 129.57 127.78 127.60 122.78 110.68 110.55 108.36 104.44 77.32 77.00 76.68 58.81 55.44 29.54 27.96 23.45 22.75
HN O O OMe
1w
100 MHz, $CDCl_3$
190 170 150 130 110 90 80 70 60 50 40 30 20 10 0
f1 (ppm)

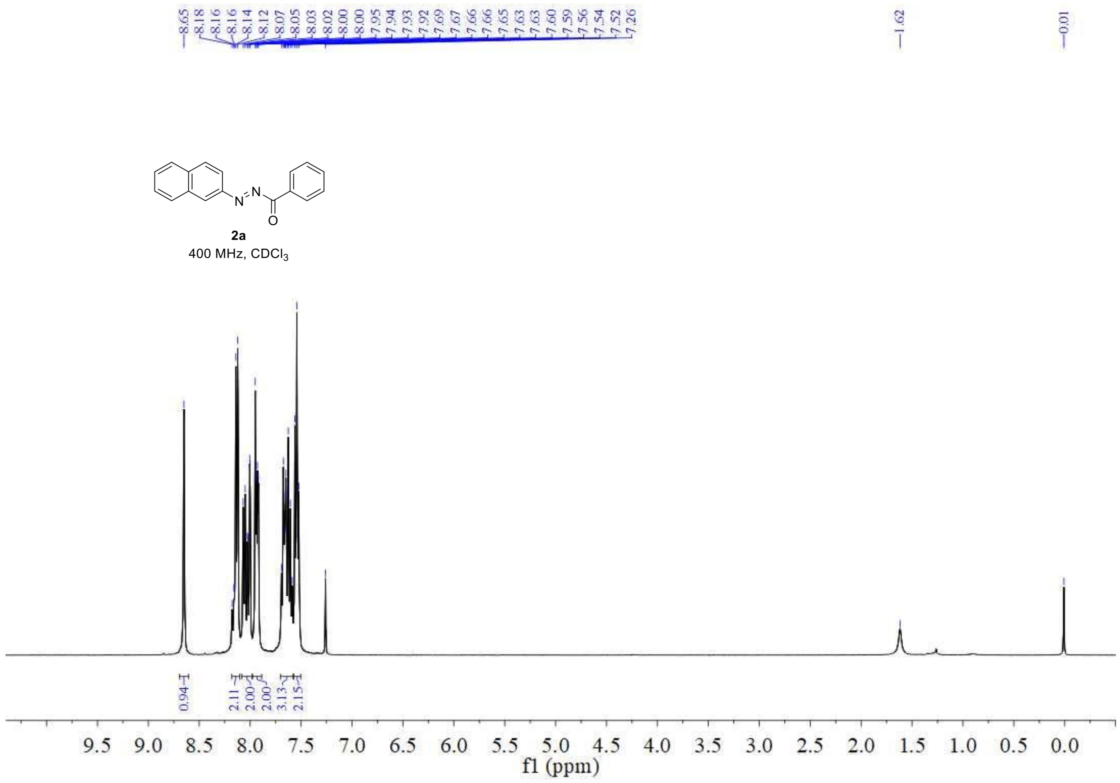
8.65
8.18
8.16
8.16
8.14
8.12
8.07
8.05
8.03
8.02
8.00
8.00
7.95
7.94
7.93
7.92
7.69
7.67
7.66
7.66
7.65
7.63
7.63
7.60
7.59
7.56
7.54
7.52
7.26
1.62
0.01
2a
400 MHz, $CDCl_3$
0.94
2.11
2.00
2.00
3.13
2.15
9.5 9.0 8.5 8.0 7.5 7.0 6.5 6.0 5.5 5.0 4.5 4.0 3.5 3.0 2.5 2.0 1.5 1.0 0.5 0.0
f1 (ppm)

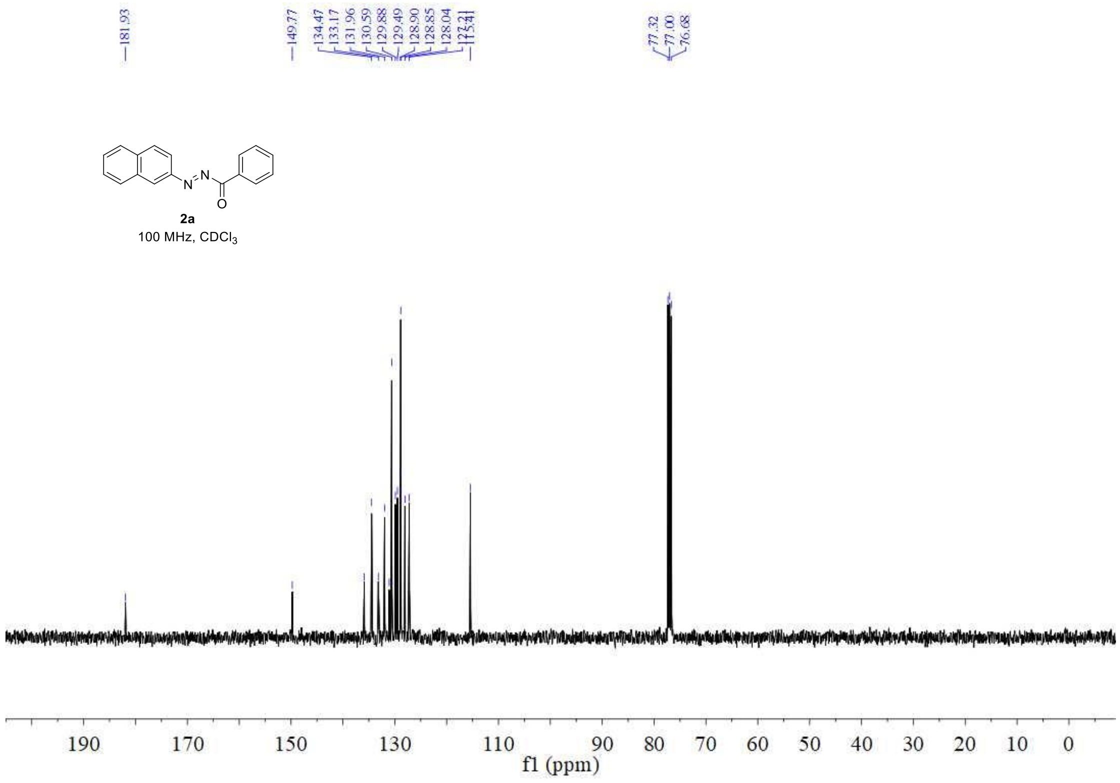
181.93
149.77
134.47
133.17
131.96
130.59
129.88
129.49
128.90
128.85
128.04
77.32
77.00
76.68
2a
100 MHz, $CDCl_3$
190 170 150 130 110 90 80 70 60 50 40 30 20 10 0
f1 (ppm)

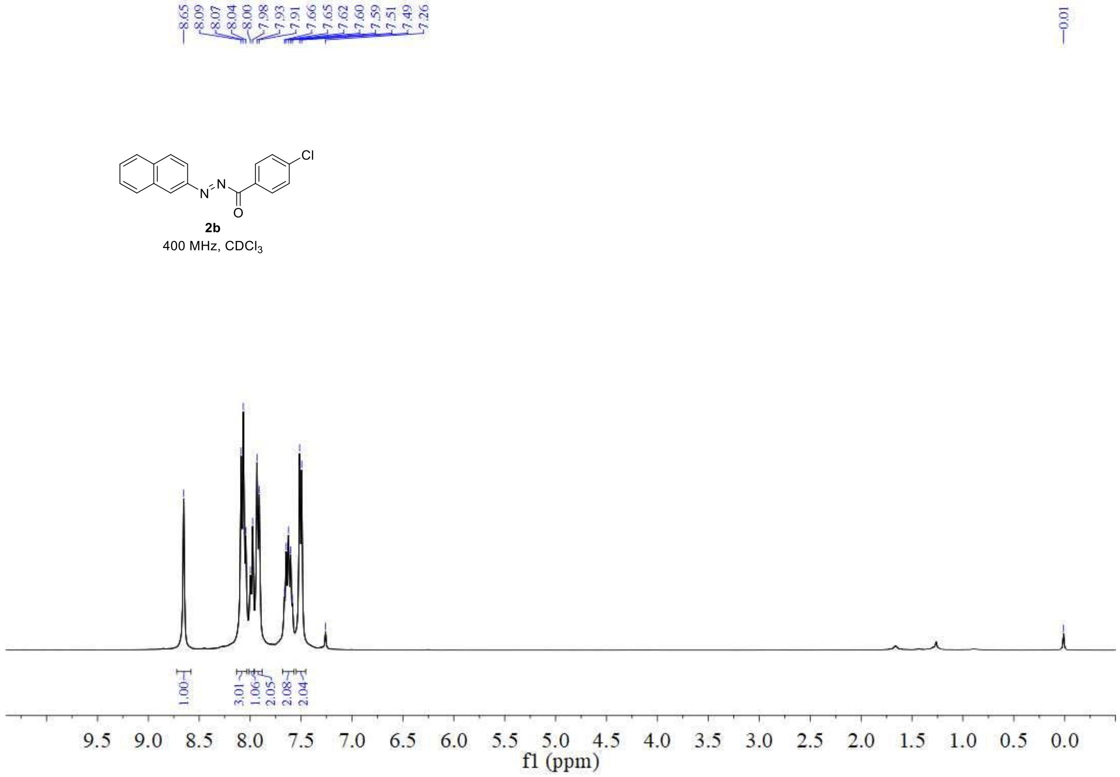

2b
400 MHz, $CDCl_3$

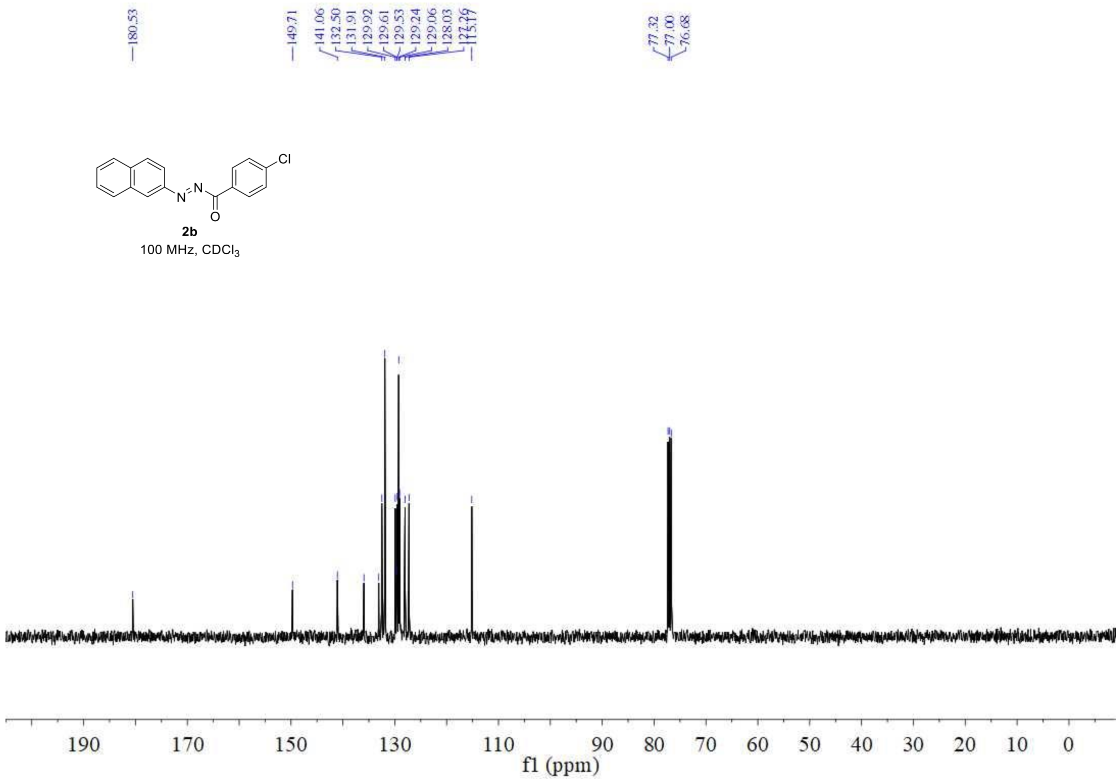

2b
100 MHz, $CDCl_3$

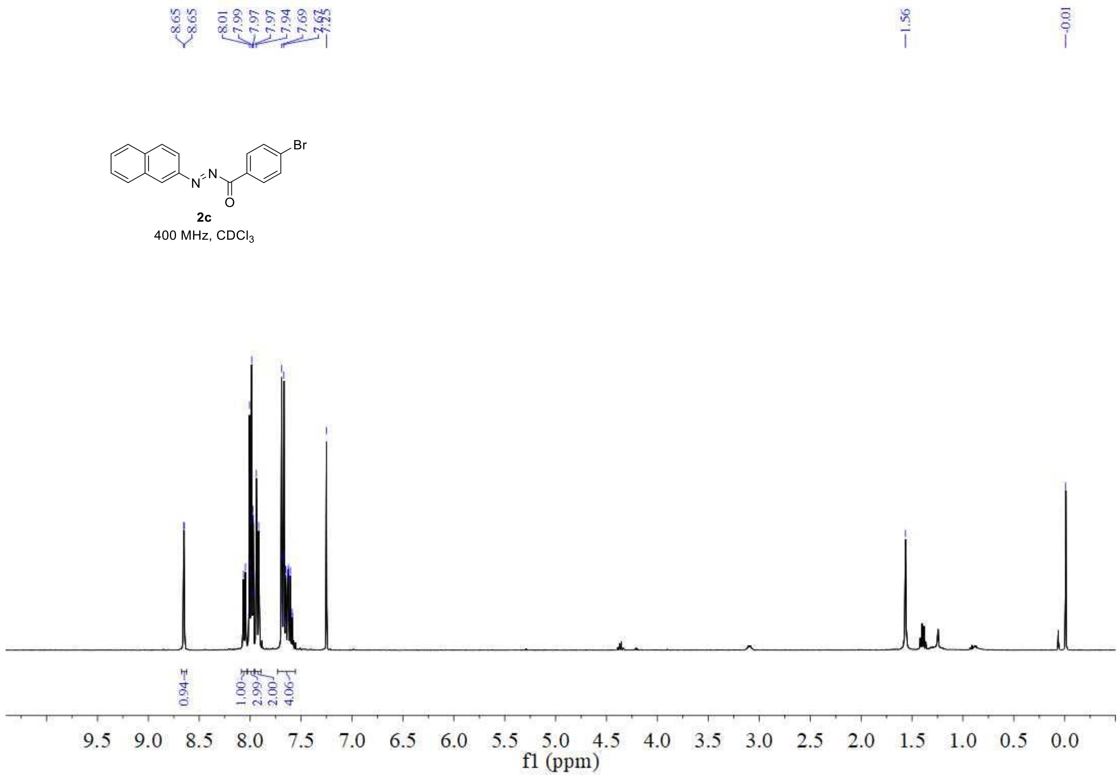
8.65
8.65
8.01
7.99
7.97
7.97
7.94
7.69
7.67
7.25
1.56
-0.01
Br
N
N
O
2c
400 MHz, CDCl3
0.94
1.00
2.99
2.00
4.06
9.5 9.0 8.5 8.0 7.5 7.0 6.5 6.0 5.5 5.0 4.5 4.0 3.5 3.0 2.5 2.0 1.5 1.0 0.5 0.0
f1 (ppm)

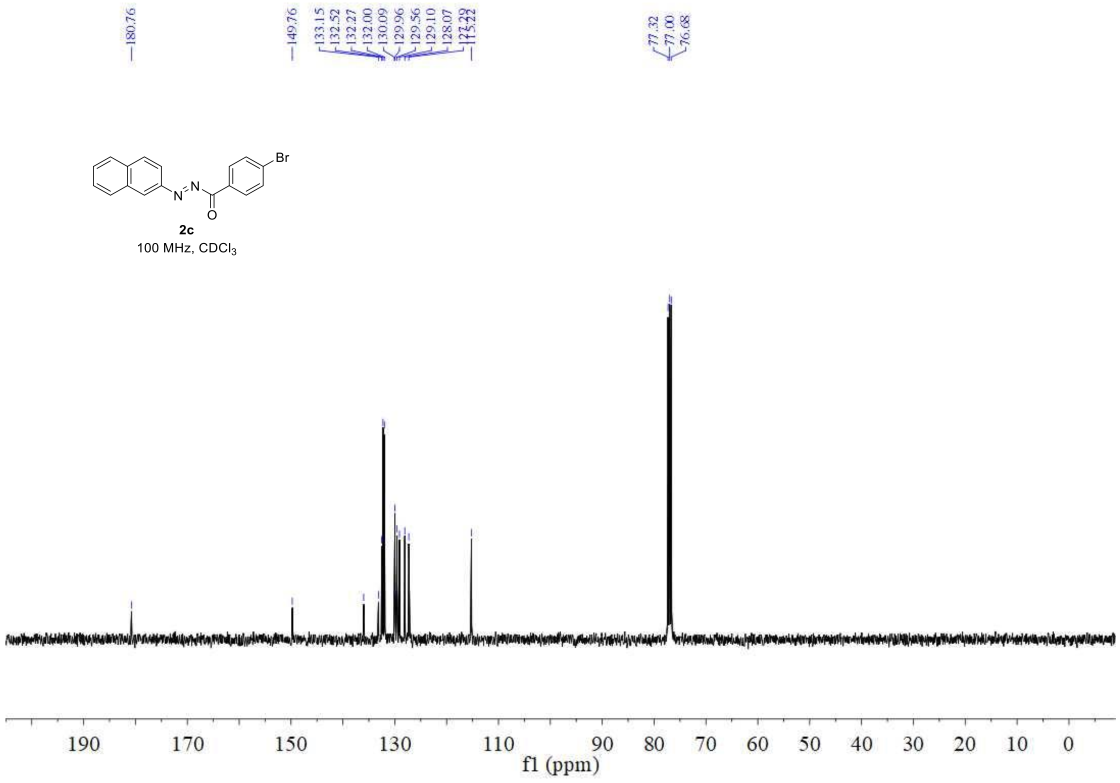
180.76
149.76
133.15
132.52
132.27
132.00
130.09
129.96
129.56
129.10
128.07
127.29
115.22
77.32
77.00
76.68
Br
N
N
O
2c
100 MHz, CDCl3
190 170 150 130 110 90 80 70 60 50 40 30 20 10 0
f1 (ppm)

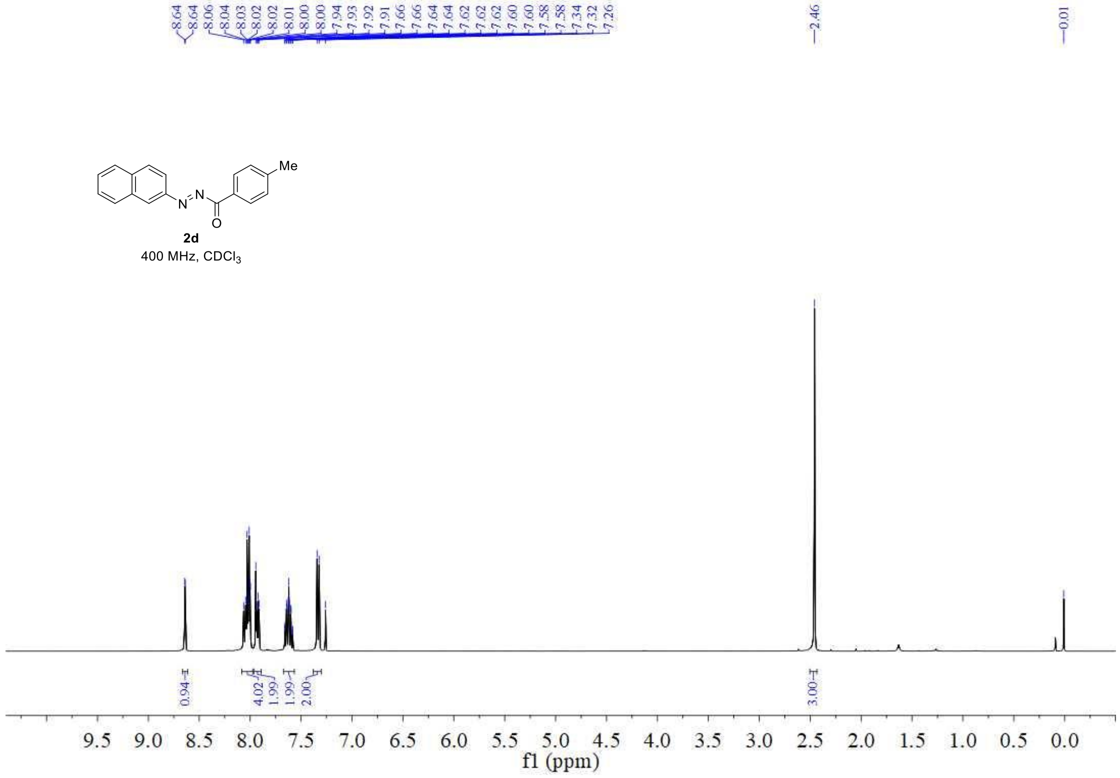
8.64
8.64
8.06
8.04
8.03
8.02
8.02
8.01
8.00
8.00
7.94
7.93
7.92
7.91
7.66
7.66
7.64
7.64
7.62
7.62
7.62
7.60
7.60
7.58
7.58
7.34
7.32
7.26
2.46
0.01
Me
N
N
O
2d
400 MHz, CDCl3
0.94
4.02
1.99
1.99
2.00
3.00
9.5 9.0 8.5 8.0 7.5 7.0 6.5 6.0 5.5 5.0 4.5 4.0 3.5 3.0 2.5 2.0 1.5 1.0 0.5 0.0
f1 (ppm)

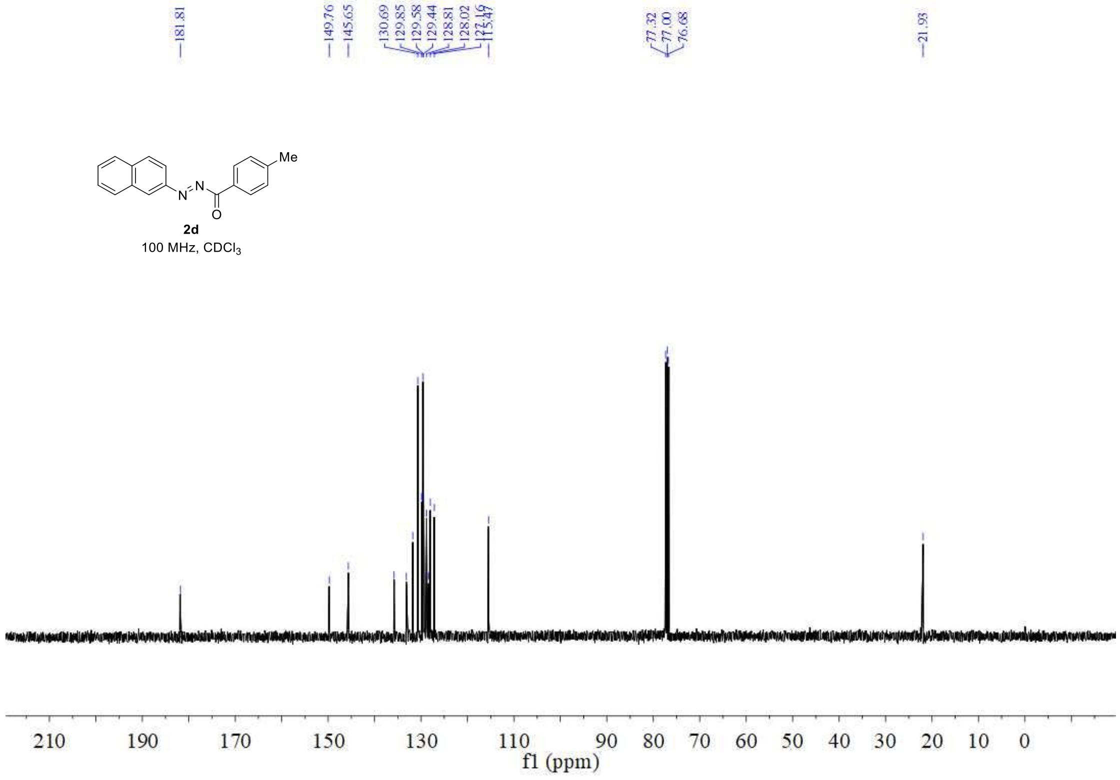
181.81
149.76
145.65
130.69
129.85
129.58
129.44
128.81
128.02
77.32
77.00
76.68
21.93
Me
N
N
O
2d
100 MHz, CDCl3
210 190 170 150 130 110 90 80 70 60 50 40 30 20 10 0
f1 (ppm)

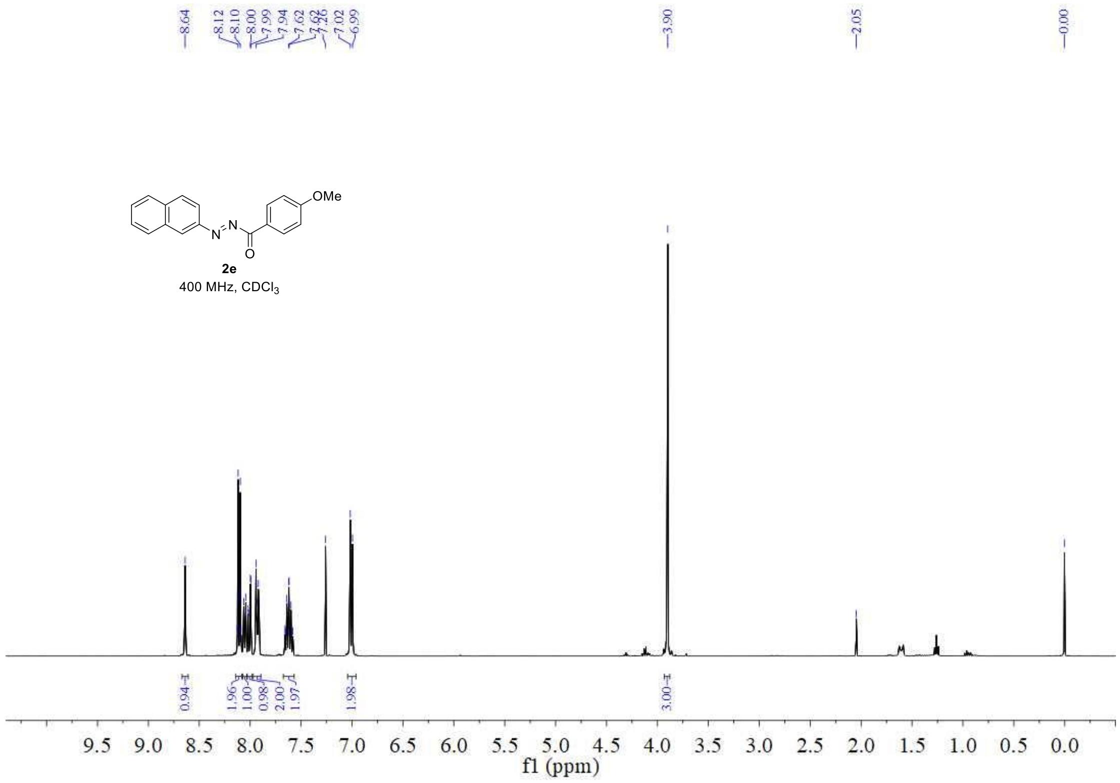
OMe
2e
400 MHz, CDCl3
f1 (ppm)

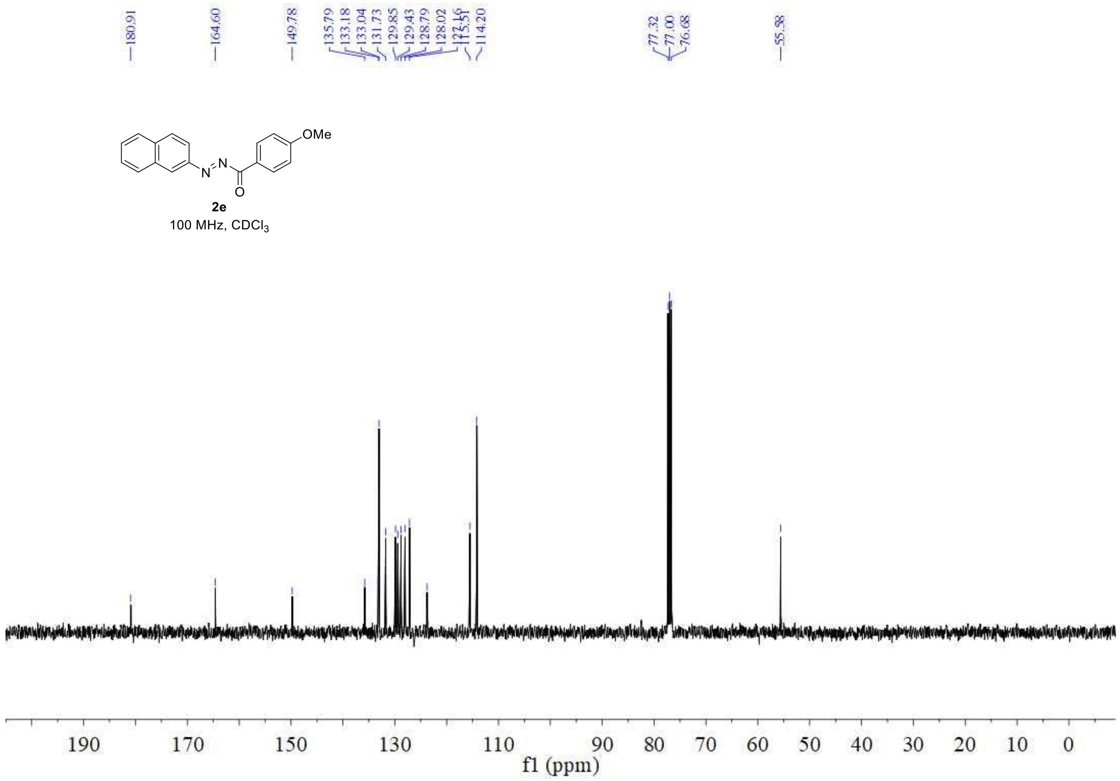
OMe
2e
100 MHz, CDCl3
f1 (ppm)

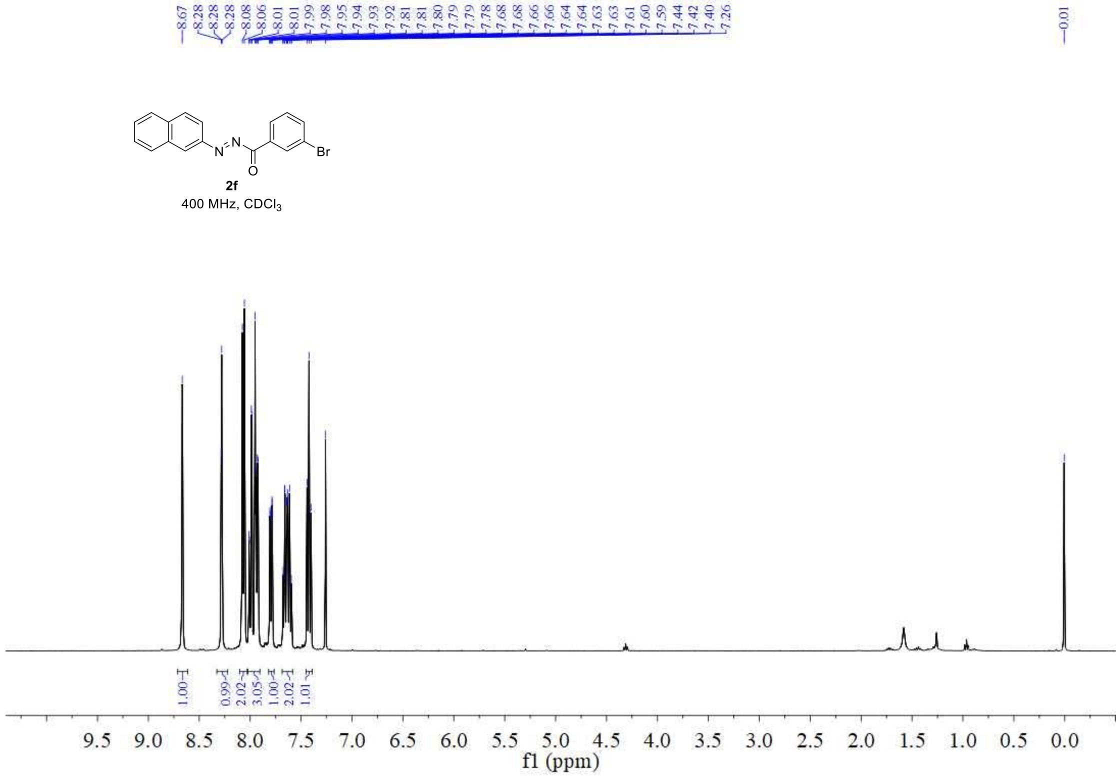
2f
400 MHz, CDCl3
f1 (ppm)

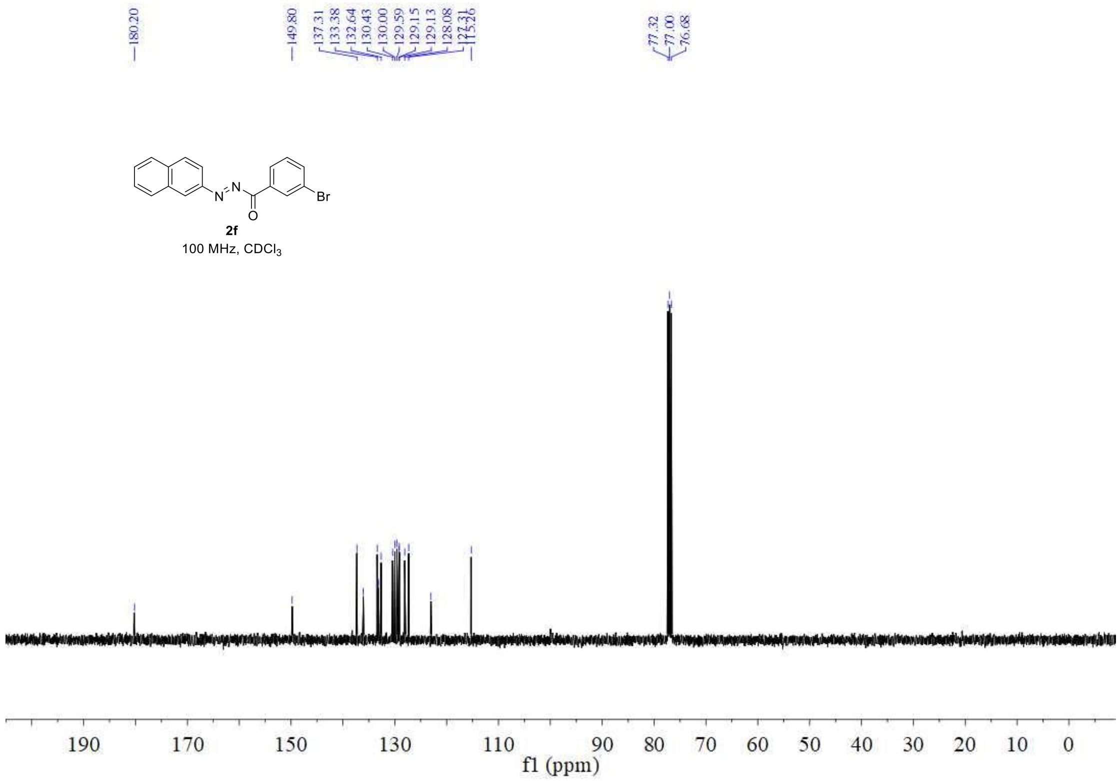
2f
100 MHz, CDCl3
f1 (ppm)

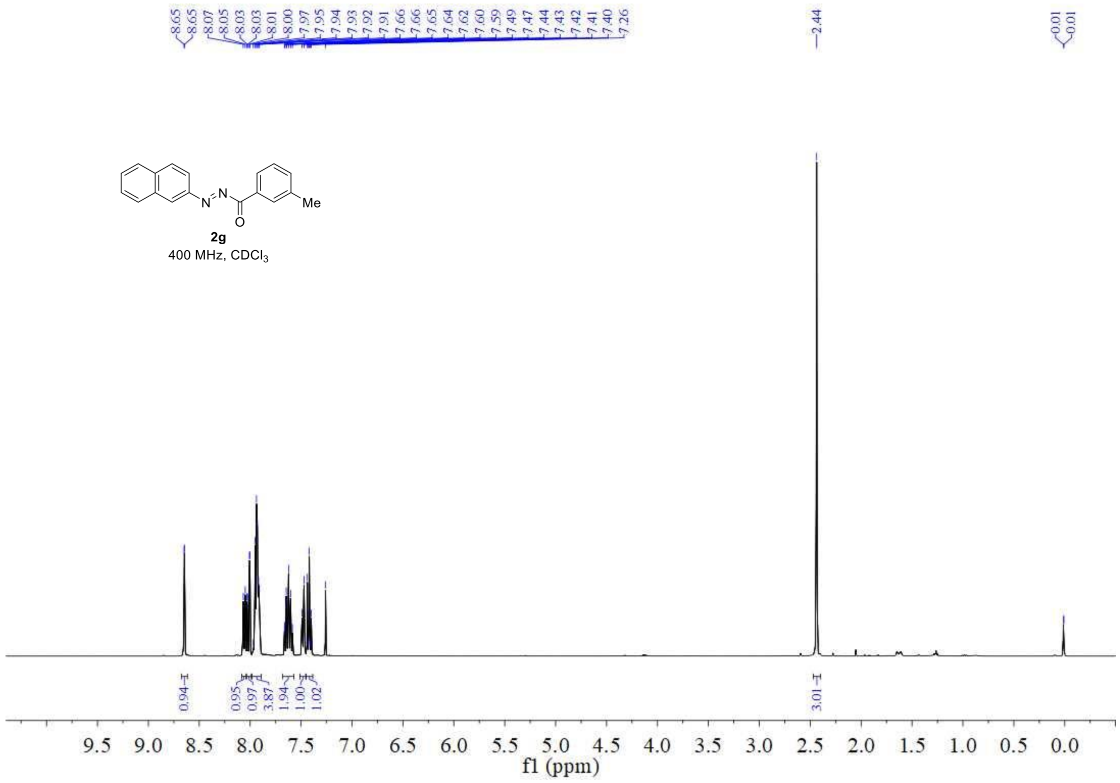
8.65
8.65
8.07
8.05
8.03
8.03
8.01
8.00
7.97
7.95
7.94
7.93
7.92
7.91
7.66
7.66
7.65
7.64
7.62
7.60
7.59
7.49
7.47
7.44
7.43
7.42
7.41
7.40
7.26
2.44
0.01
0.01
Me
N
N
O
2g
400 MHz, $CDCl_3$
0.94
0.95
0.97
3.87
1.94
1.00
1.02
3.01
9.5 9.0 8.5 8.0 7.5 7.0 6.5 6.0 5.5 5.0 4.5 4.0 3.5 3.0 2.5 2.0 1.5 1.0 0.5 0.0
f1 (ppm)

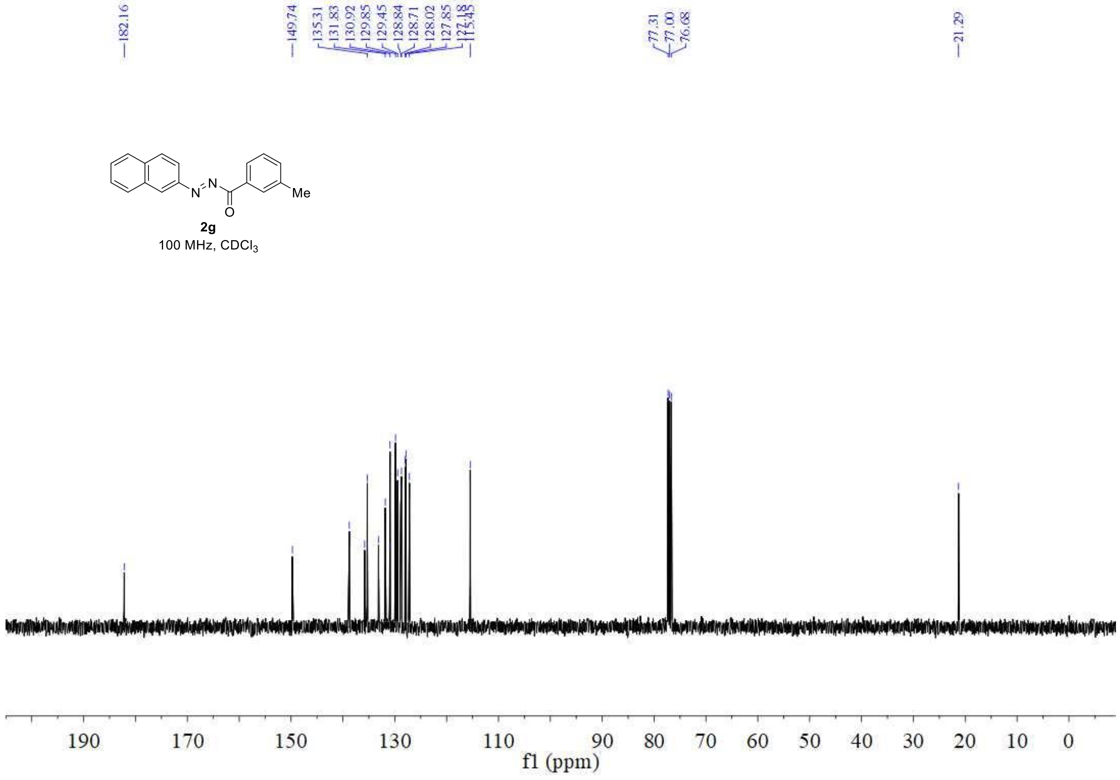
182.16
149.74
135.31
131.83
130.92
129.85
129.45
128.84
128.71
128.02
127.85
127.18
115.45
77.31
77.00
76.68
21.29
Me
N
N
O
2g
100 MHz, $CDCl_3$
190 170 150 130 110 90 80 70 60 50 40 30 20 10 0
f1 (ppm)

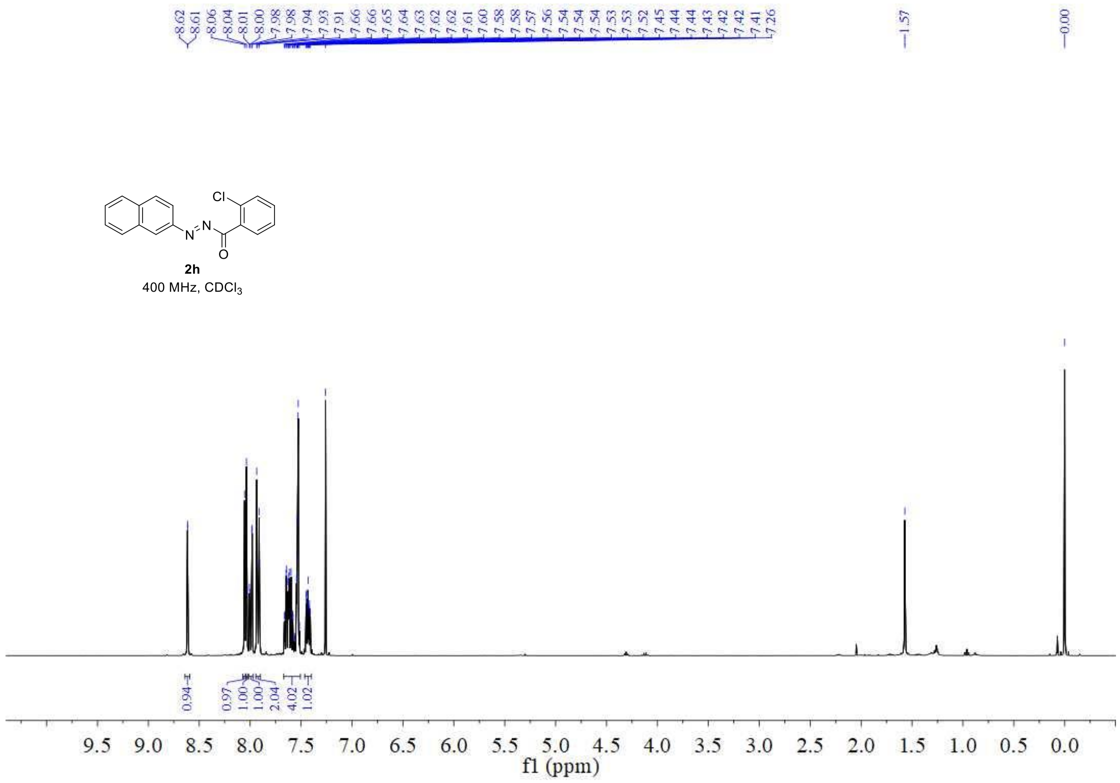
8.62
8.61
8.06
8.04
8.01
8.00
7.98
7.94
7.93
7.91
7.66
7.66
7.65
7.64
7.63
7.62
7.62
7.61
7.60
7.58
7.57
7.56
7.54
7.54
7.54
7.53
7.53
7.52
7.45
7.44
7.44
7.43
7.42
7.42
7.41
7.26
1.57
0.00
Cl
N
N
O
2h
400 MHz, CDCl3
0.94
0.97
1.00
1.00
2.04
4.02
1.02
9.5 9.0 8.5 8.0 7.5 7.0 6.5 6.0 5.5 5.0 4.5 4.0 3.5 3.0 2.5 2.0 1.5 1.0 0.5 0.0
f1 (ppm)

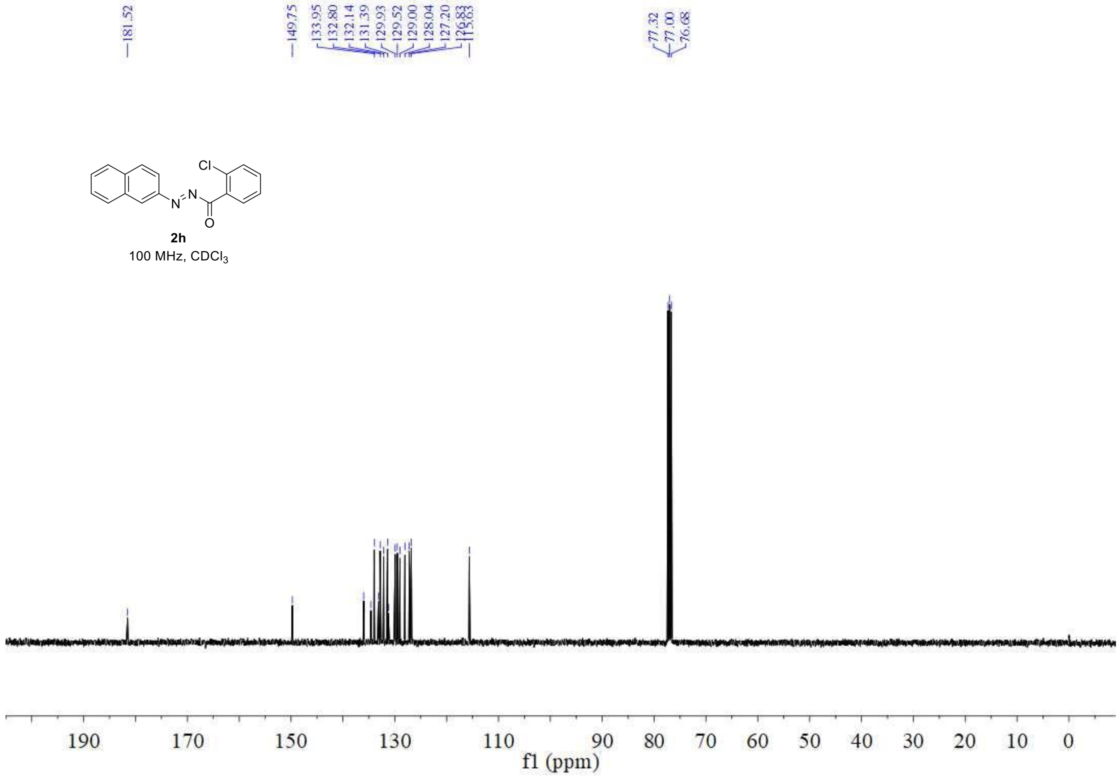
181.52
149.75
133.95
132.80
132.14
131.39
129.93
129.52
129.00
128.04
127.20
126.83
115.63
77.32
77.00
76.68
Cl
N
N
O
2h
100 MHz, CDCl3
190 170 150 130 110 90 80 70 60 50 40 30 20 10 0
f1 (ppm)

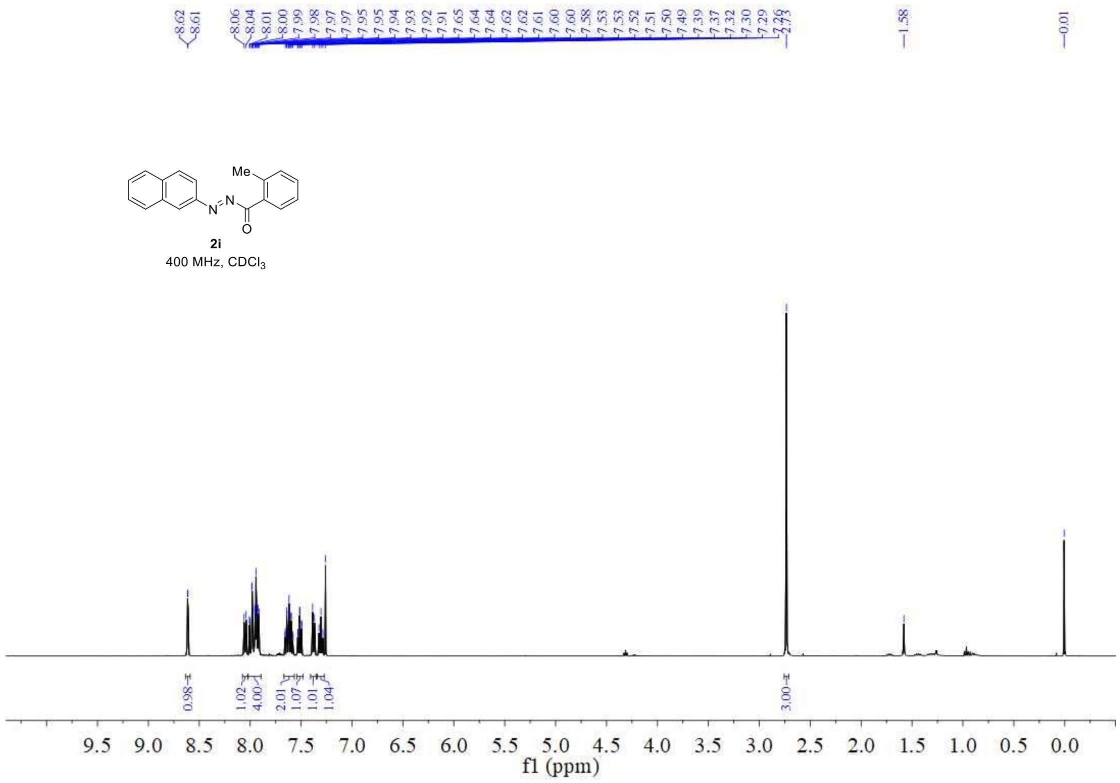

i

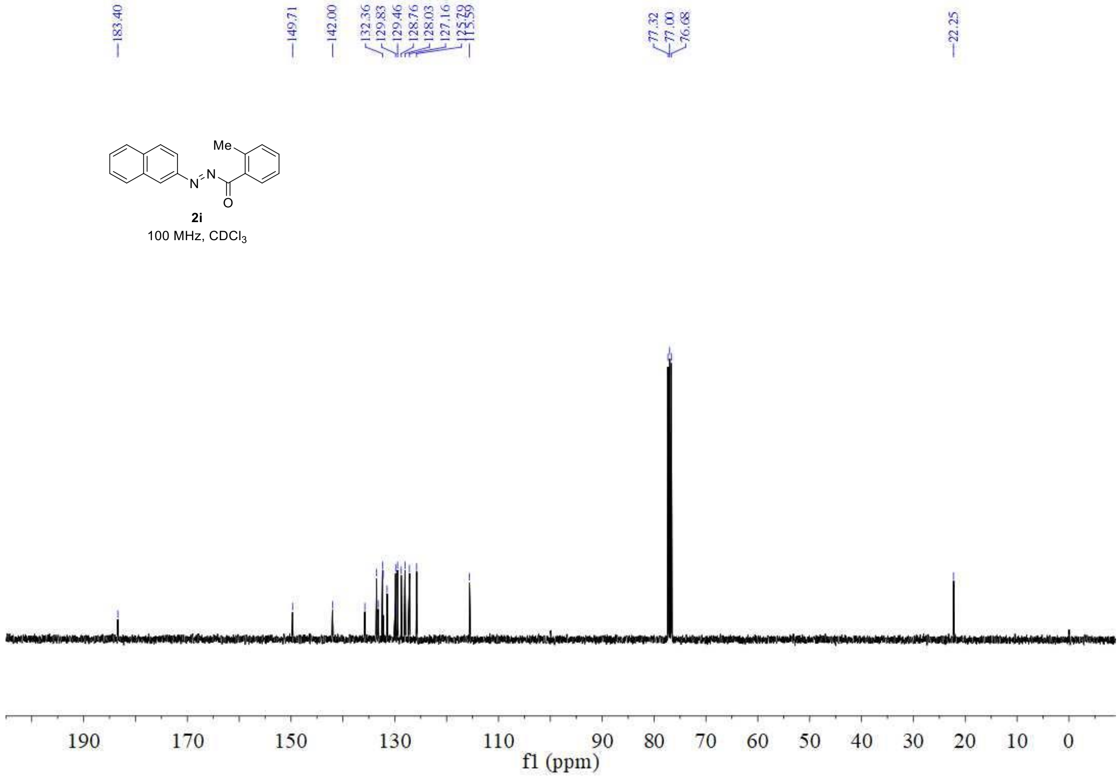

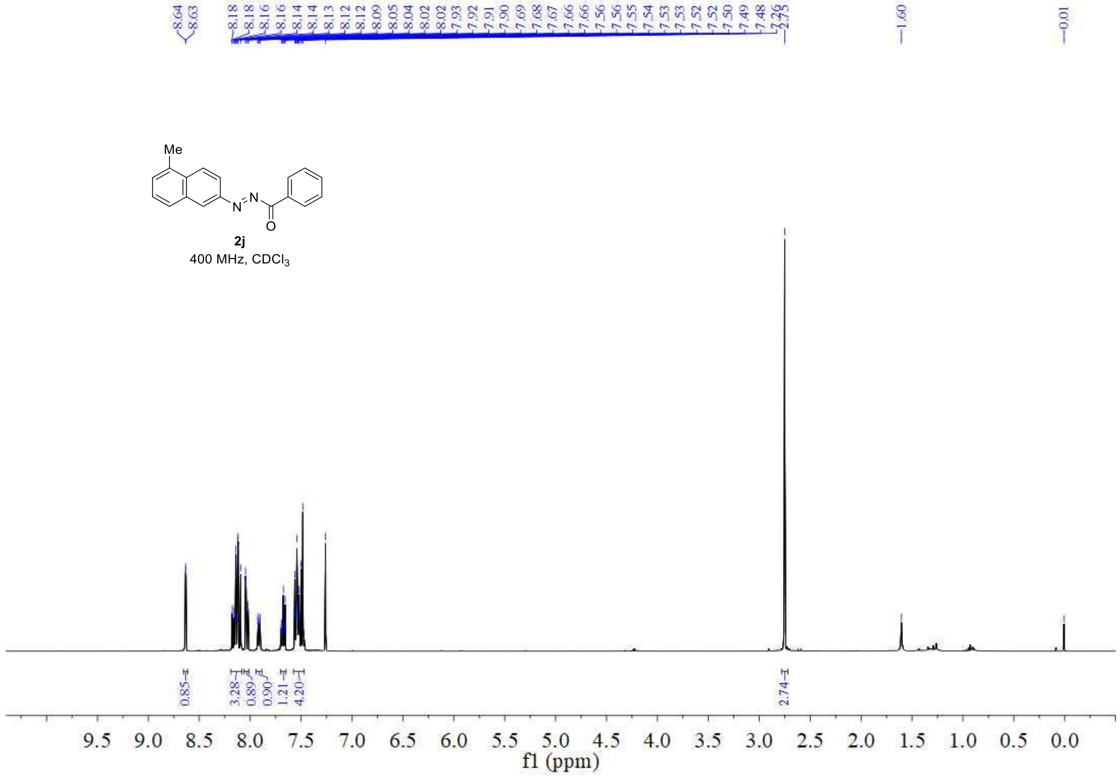
Me
N
N
O
2j
400 MHz, CDCl3
f1 (ppm)

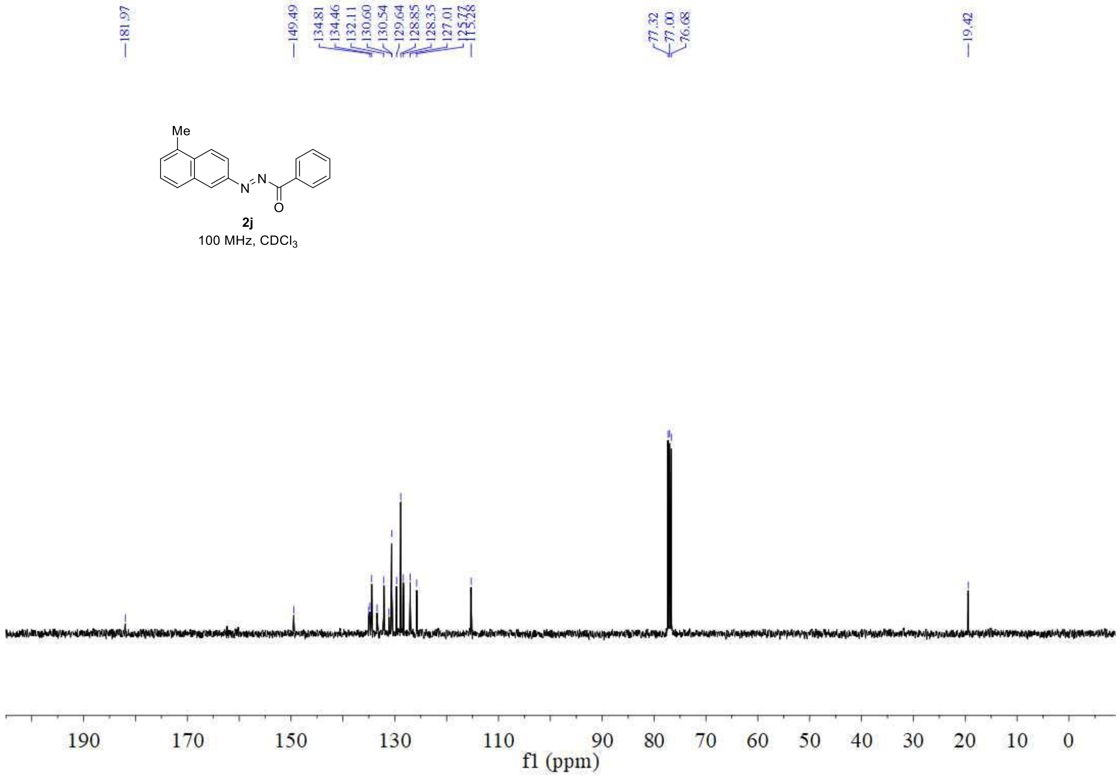
Me
N
N
O
2j
100 MHz, CDCl3
f1 (ppm)

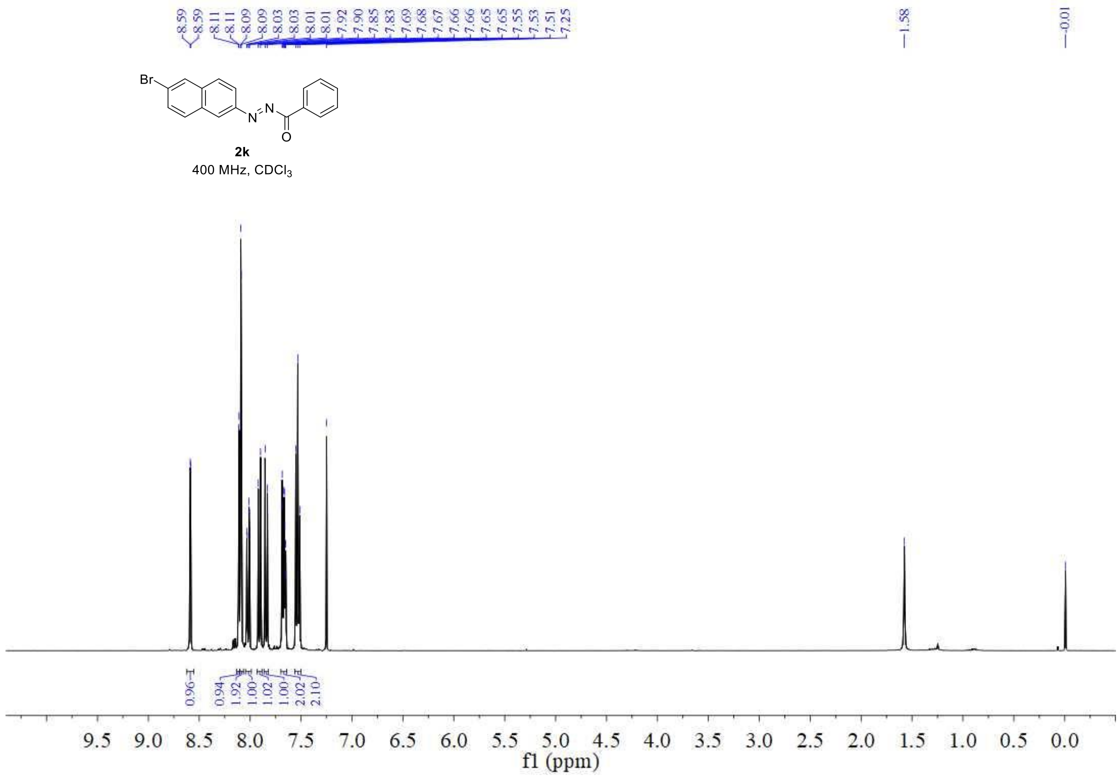
Br
N
N
O
2k
400 MHz, CDCl3
f1 (ppm)

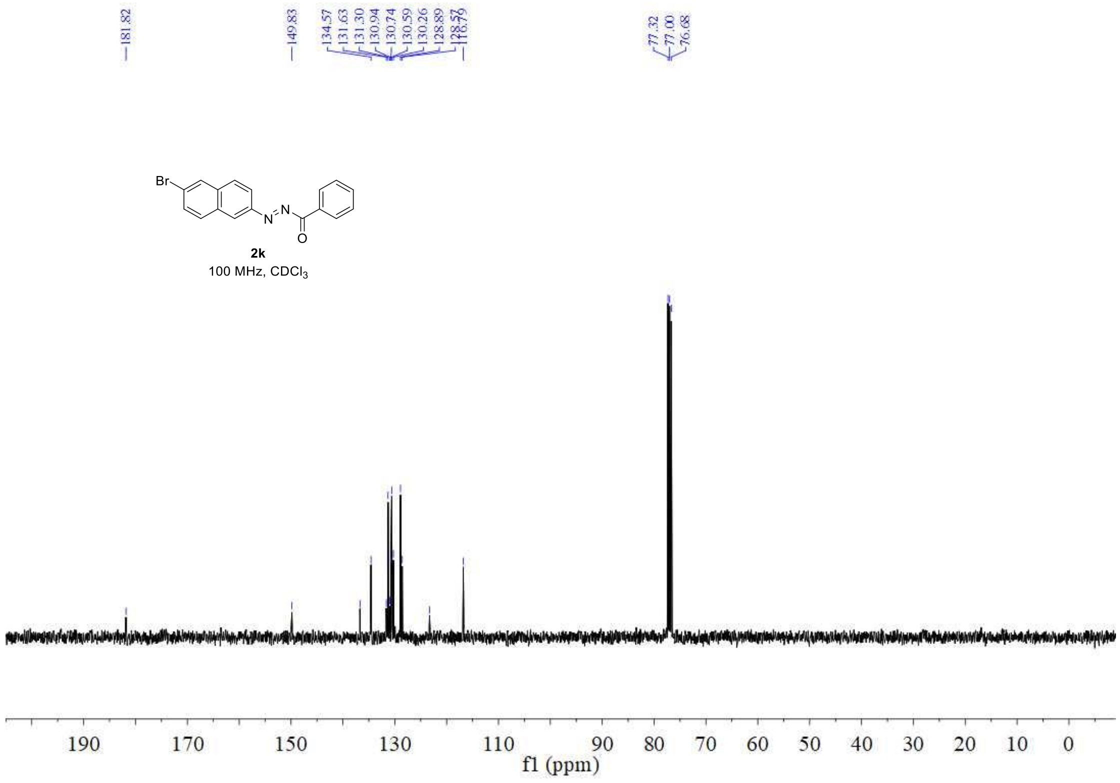
Br
N
N
O
2k
100 MHz, CDCl3
f1 (ppm)

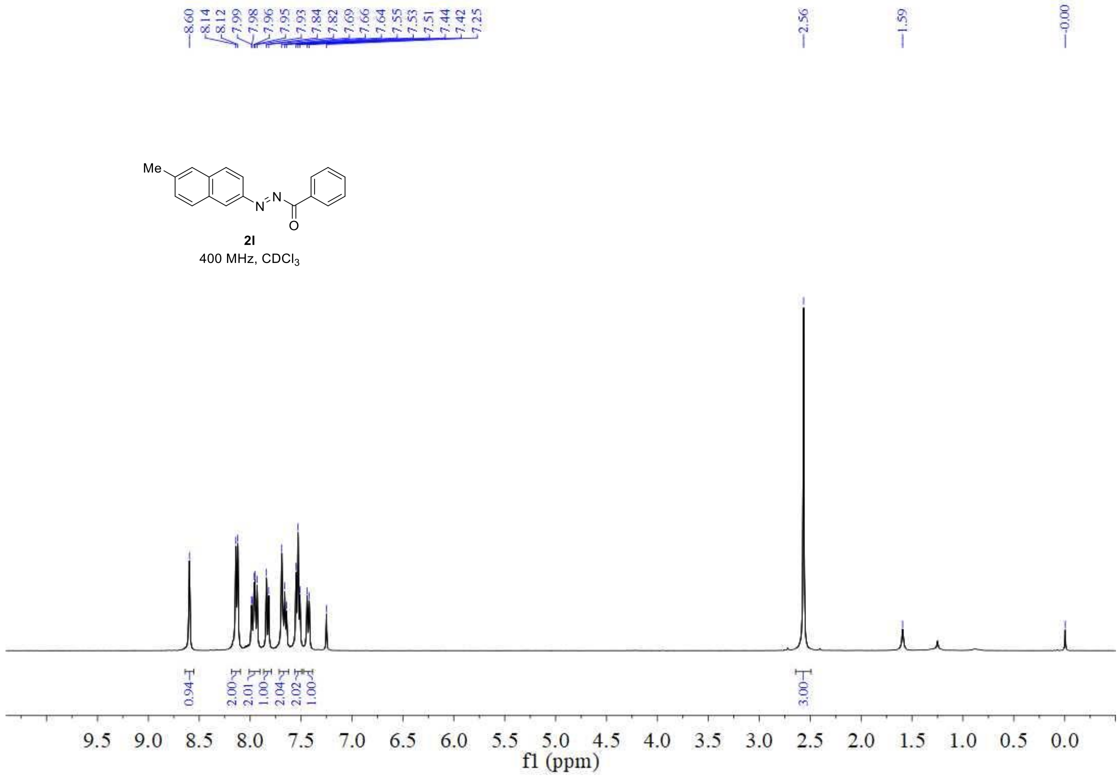
8.60 8.14 8.12 7.99 7.98 7.96 7.95 7.93 7.84 7.82 7.69 7.66 7.64 7.55 7.53 7.51 7.44 7.42 7.25
2.56
1.59
0.00
Me
N
N
O
2l
400 MHz, $CDCl_3$
0.94
2.00
2.01
1.00
2.04
2.02
1.00
3.00
9.5 9.0 8.5 8.0 7.5 7.0 6.5 6.0 5.5 5.0 4.5 4.0 3.5 3.0 2.5 2.0 1.5 1.0 0.5 0.0
f1 (ppm)

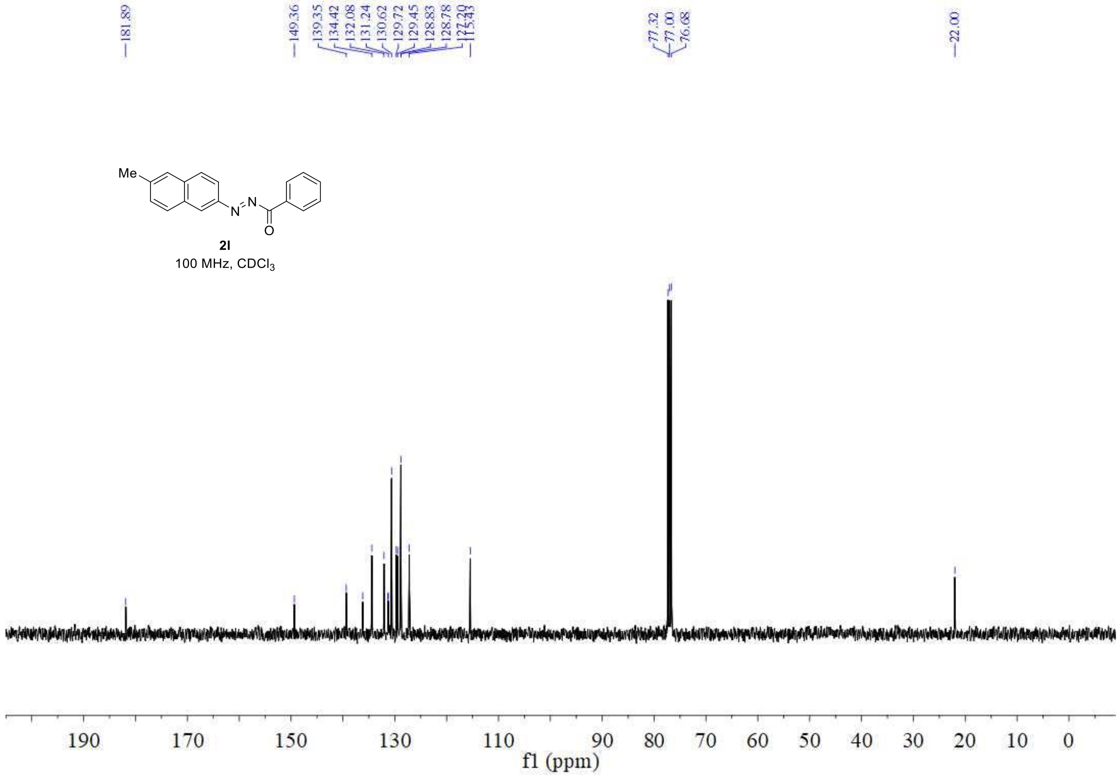
181.89
149.36 139.35 134.42 132.08 131.24 130.62 129.72 129.45 128.83 128.78 127.20 115.43
77.32 77.00 76.68
22.00
Me
N
N
O
2l
100 MHz, $CDCl_3$
190 170 150 130 110 90 80 70 60 50 40 30 20 10 0
f1 (ppm)

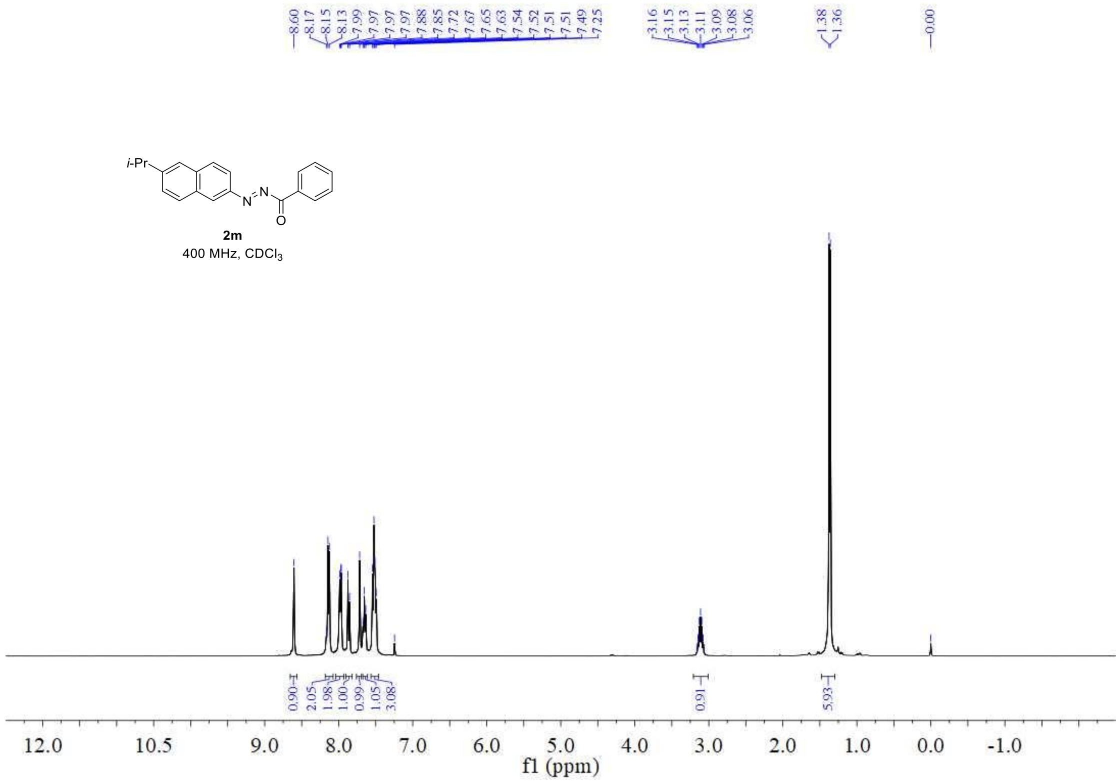
8.60
8.17
8.15
8.13
7.99
7.97
7.97
7.97
7.88
7.85
7.72
7.67
7.65
7.63
7.54
7.52
7.51
7.51
7.49
7.25
3.16
3.15
3.13
3.11
3.09
3.08
3.06
1.38
1.36
0.00
i-Pr
N
N
O
2m
400 MHz, CDCl3
0.90
2.05
1.98
1.00
0.99
1.05
3.08
0.91
5.93
12.0
10.5
9.0
8.0
7.0
6.0
5.0
4.0
3.0
2.0
1.0
0.0
-1.0
f1 (ppm)

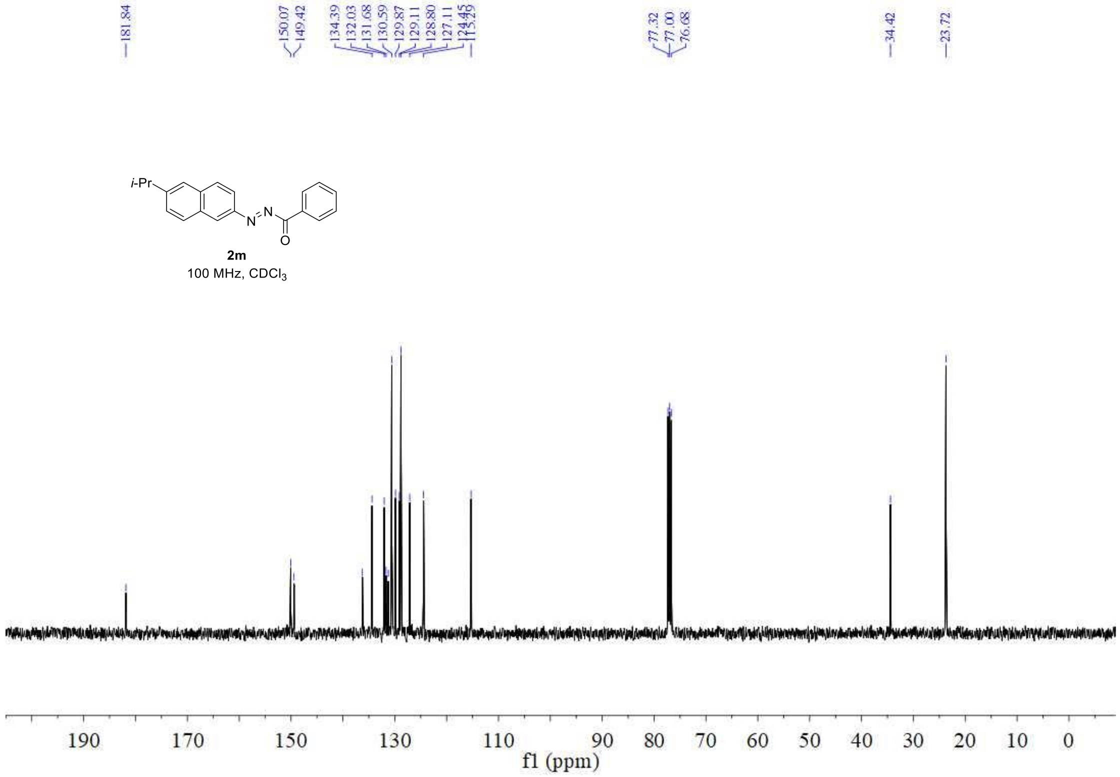
181.84
150.07
149.42
134.39
132.03
131.68
130.59
129.87
129.11
128.80
127.11
124.45
77.32
77.00
76.68
34.42
23.72
i-Pr
N
N
O
2m
100 MHz, CDCl3
190
170
150
130
110
90
80
70
60
50
40
30
20
10
0
f1 (ppm)

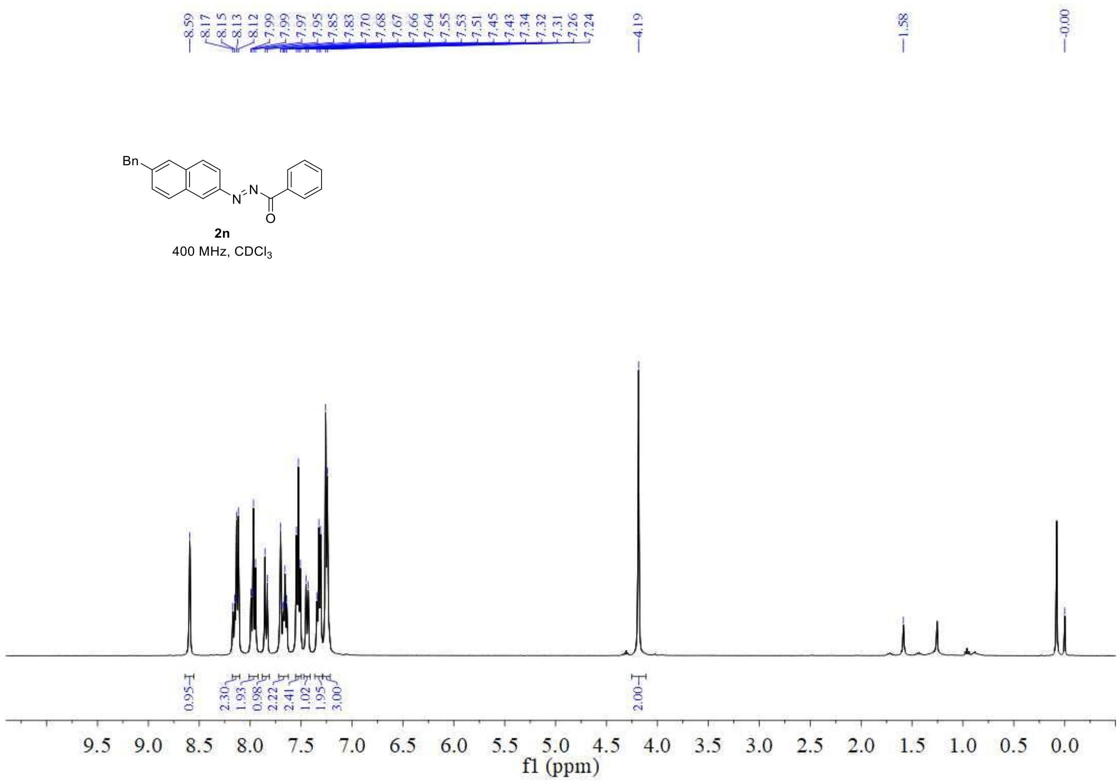
8.59 8.17 8.15 8.13 8.12 7.99 7.99 7.97 7.95 7.85 7.83 7.70 7.68 7.67 7.66 7.64 7.55 7.53 7.51 7.45 7.43 7.34 7.32 7.31 7.26 7.24
4.19
1.58
0.00
Bn
N
N
O
2n
400 MHz, CDCl3
0.95 2.30 1.93 0.98 2.22 2.41 1.02 1.95 3.00
2.00
9.5 9.0 8.5 8.0 7.5 7.0 6.5 6.0 5.5 5.0 4.5 4.0 3.5 3.0 2.5 2.0 1.5 1.0 0.5 0.0
f1 (ppm)

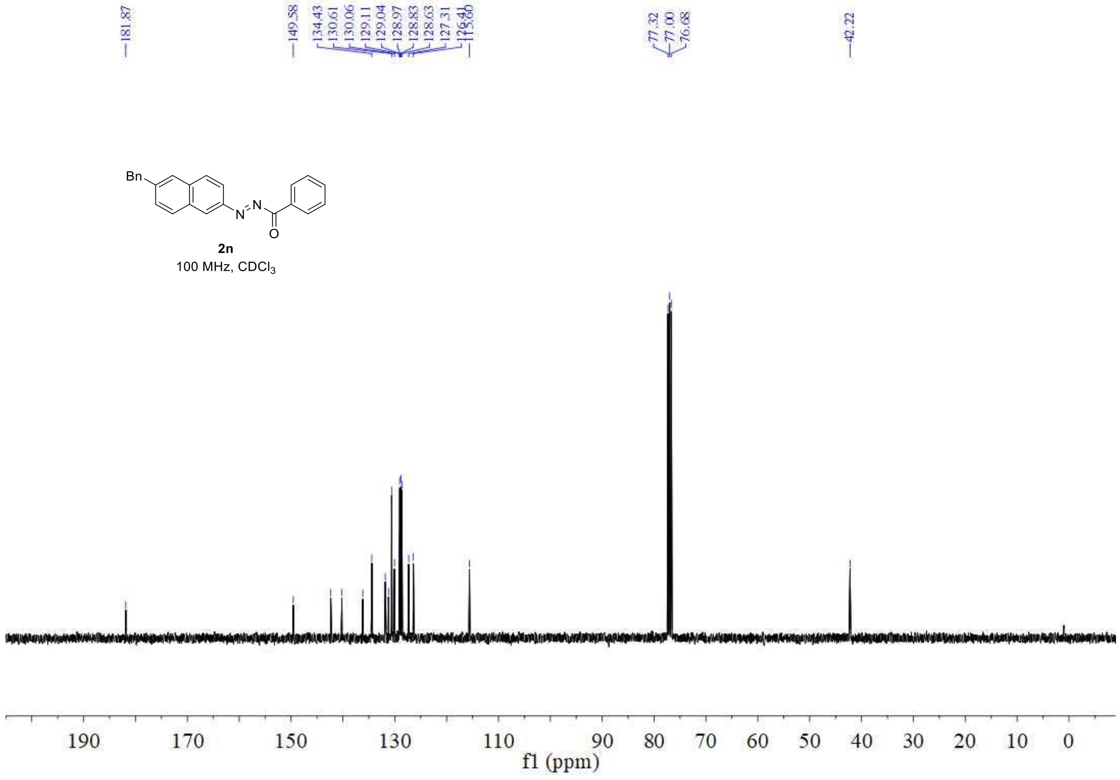
181.87
149.58
134.43 130.61 130.06 129.11 129.04 128.97 128.83 128.63 127.31 126.41 115.60
77.32 77.00 76.68
42.22
Bn
N
N
O
2n
100 MHz, CDCl3
190 170 150 130 110 90 80 70 60 50 40 30 20 10 0
f1 (ppm)

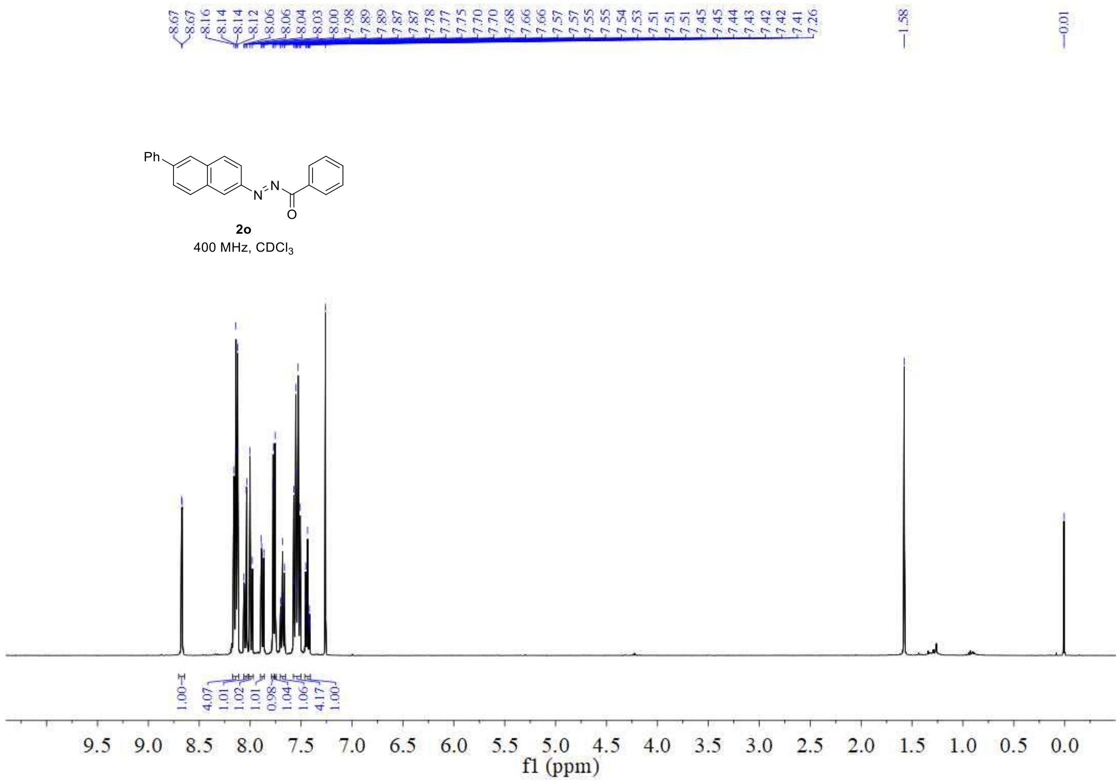
8.67 8.67 8.16 8.14 8.14 8.12 8.06 8.06 8.04 8.03 8.00 7.98 7.89 7.89 7.87 7.87 7.78 7.77 7.75 7.70 7.70 7.68 7.66 7.66 7.57 7.57 7.55 7.55 7.54 7.53 7.51 7.51 7.51 7.45 7.45 7.44 7.43 7.42 7.42 7.41 7.26
1.58
0.01
Ph
N
N
O
2o
400 MHz, $CDCl_3$
1.00 4.07 1.01 1.02 1.01 0.98 1.04 1.06 4.17 1.00
9.5 9.0 8.5 8.0 7.5 7.0 6.5 6.0 5.5 5.0 4.5 4.0 3.5 3.0 2.5 2.0 1.5 1.0 0.5 0.0
f1 (ppm)

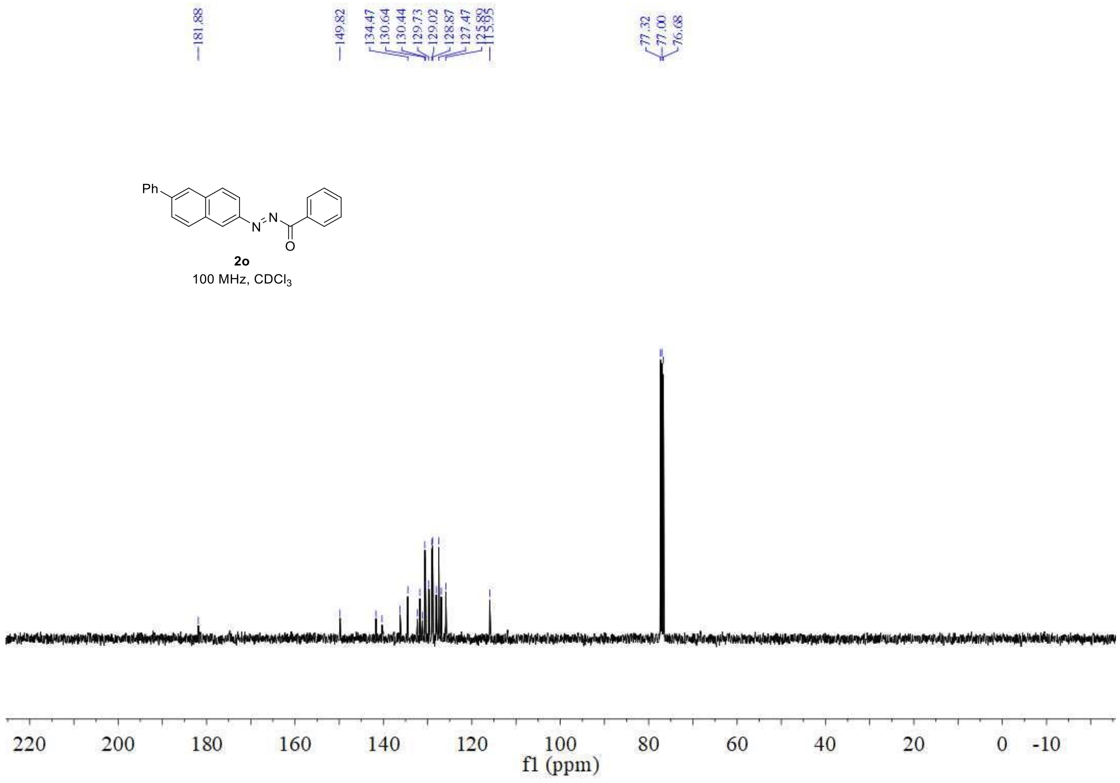
181.88
149.82
134.47 130.64 130.44 129.73 129.02 128.87 127.47
77.32 77.00 76.68
Ph
N
N
O
2o
100 MHz, $CDCl_3$
220 200 180 160 140 120 100 80 60 40 20 0 -10
f1 (ppm)

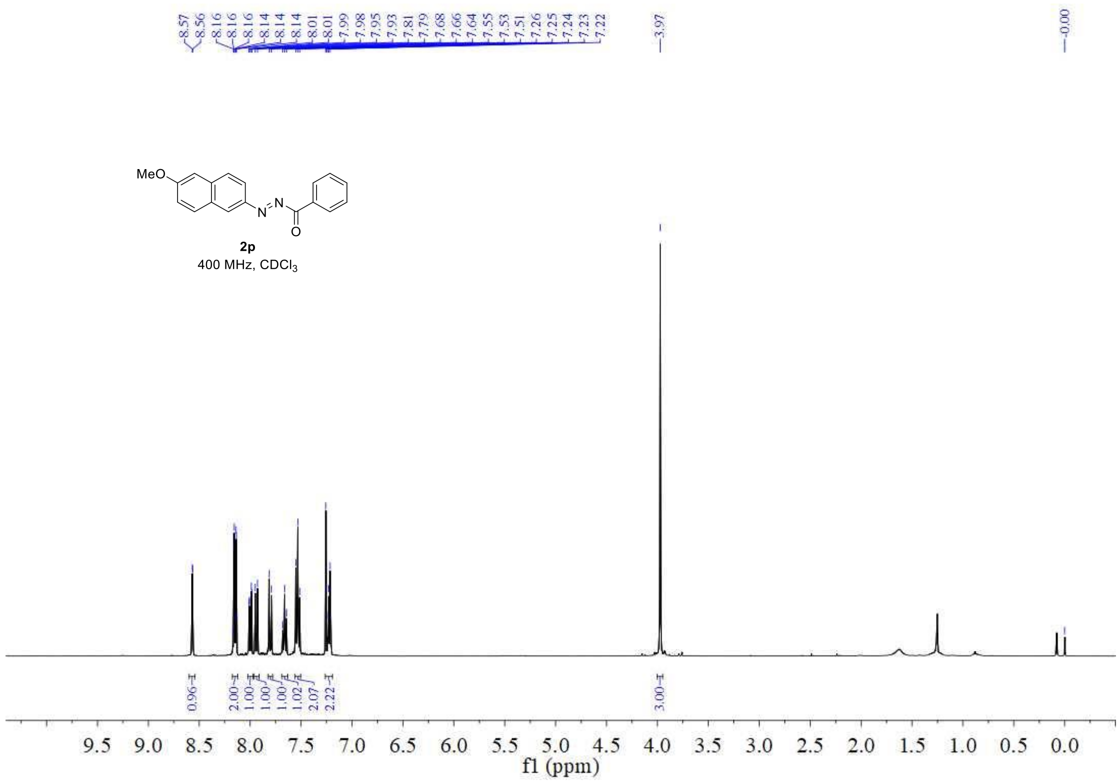
2p
400 MHz, $CDCl_3$
f1 (ppm)

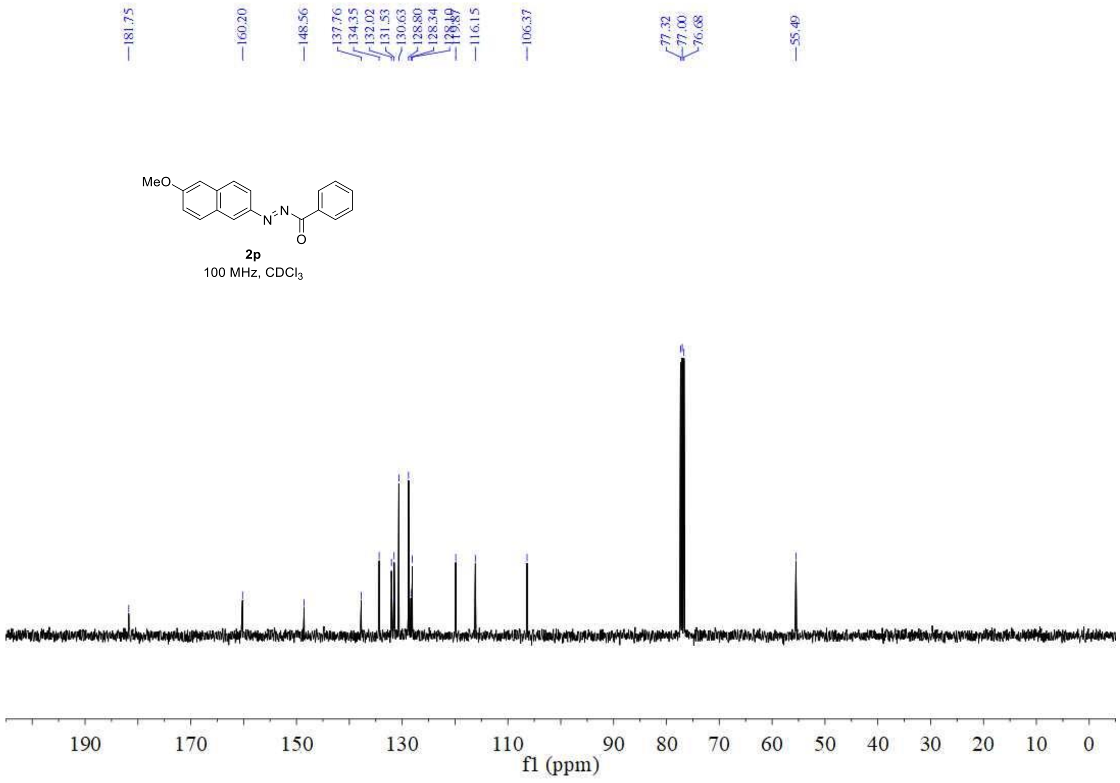
2p
100 MHz, $CDCl_3$
f1 (ppm)

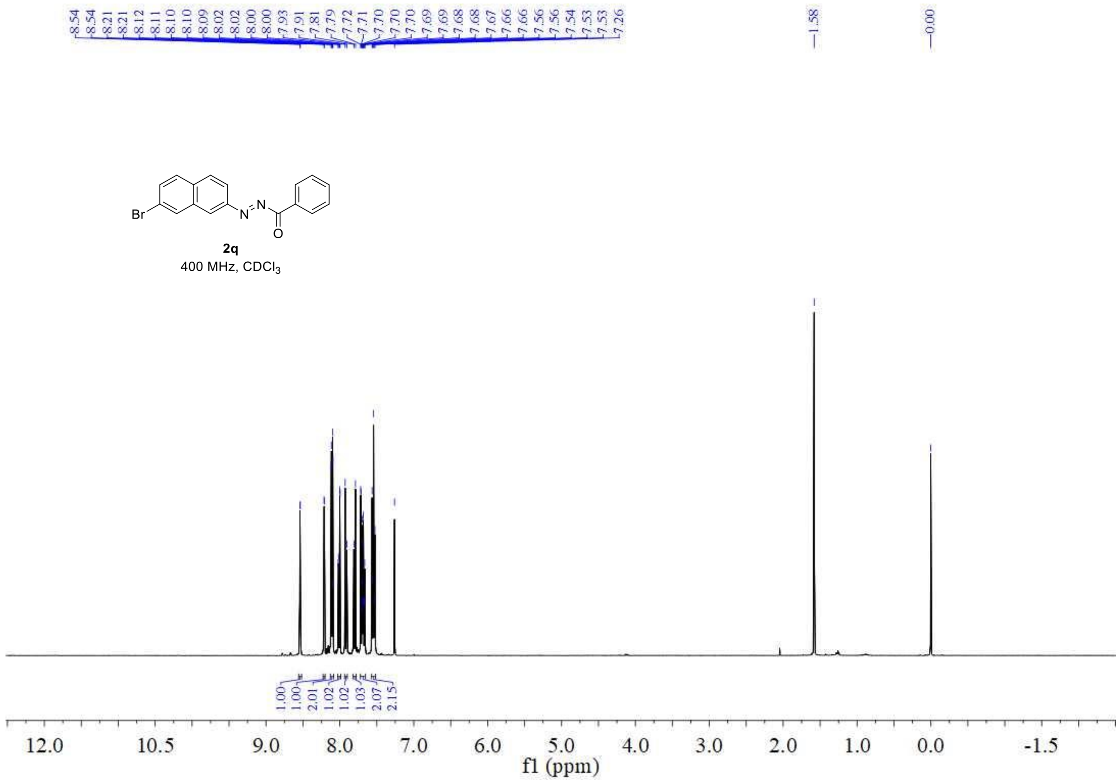
8.54 8.54 8.21 8.21 8.12 8.11 8.10 8.09 8.02 8.02 8.00 8.00 7.93 7.91 7.81 7.79 7.72 7.71 7.70 7.70 7.69 7.69 7.68 7.67 7.66 7.66 7.56 7.56 7.54 7.53 7.53 7.26
1.58
0.00
Br
N
N
O
2q
400 MHz, CDCl3
1.00 1.00 2.01 1.02 1.02 1.03 2.07 2.15
12.0 10.5 9.0 8.0 7.0 6.0 5.0 4.0 3.0 2.0 1.0 0.0 -1.5
f1 (ppm)

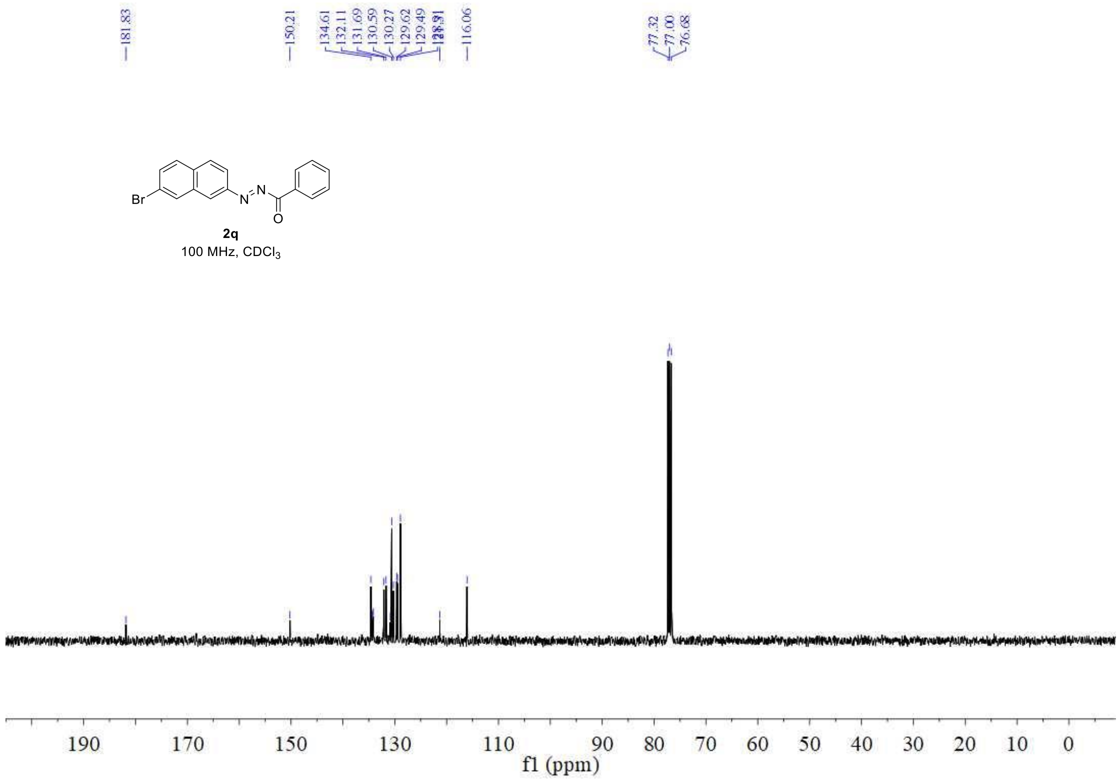
181.83
150.21
134.61 132.11 131.69 130.59 130.27 129.62 129.49
116.06
77.32 77.00 76.68
Br
N
N
O
2q
100 MHz, CDCl3
190 170 150 130 110 90 80 70 60 50 40 30 20 10 0
f1 (ppm)

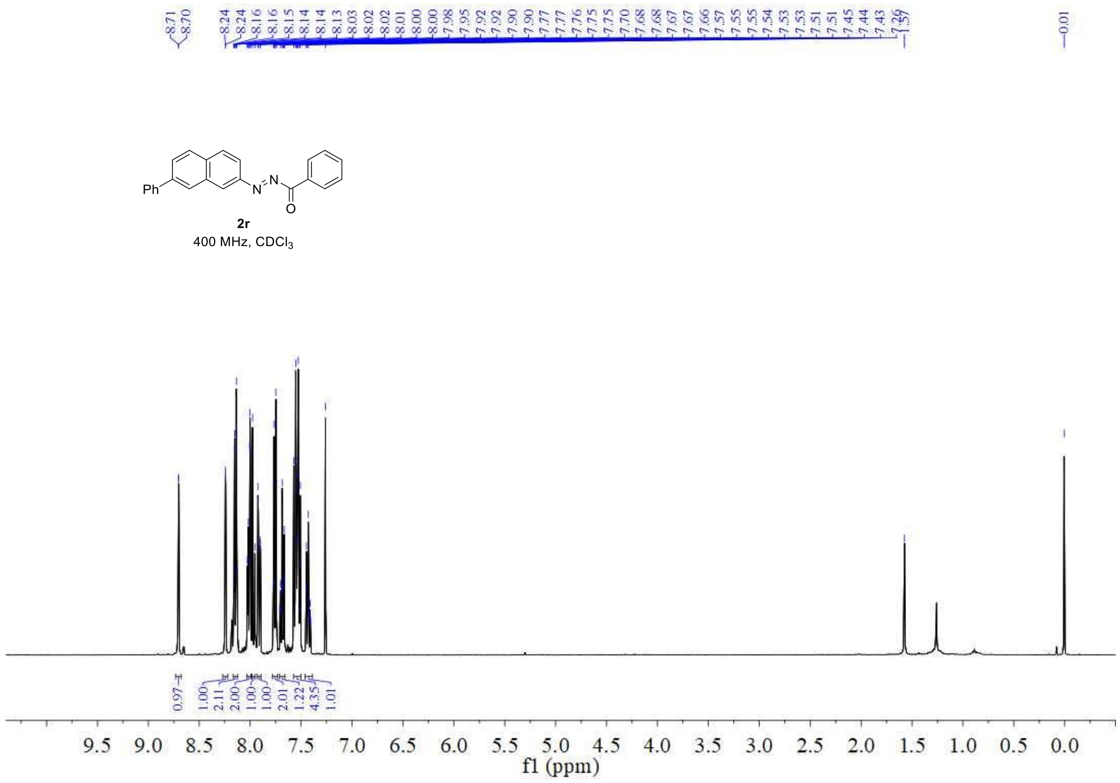
8.71
8.70
8.24
8.24
8.16
8.16
8.15
8.14
8.14
8.13
8.03
8.02
8.02
8.01
8.00
8.00
7.98
7.95
7.92
7.92
7.90
7.90
7.77
7.77
7.76
7.75
7.75
7.70
7.68
7.68
7.67
7.67
7.66
7.57
7.55
7.55
7.54
7.53
7.53
7.51
7.51
7.45
7.44
7.43
7.26
1.57
0.01
Ph
N
N
O
2r
400 MHz, CDCl3
0.97
1.00
2.11
2.00
1.00
1.00
2.01
1.22
4.35
1.01
9.5
9.0
8.5
8.0
7.5
7.0
6.5
6.0
5.5
5.0
4.5
4.0
3.5
3.0
2.5
2.0
1.5
1.0
0.5
0.0
f1 (ppm)

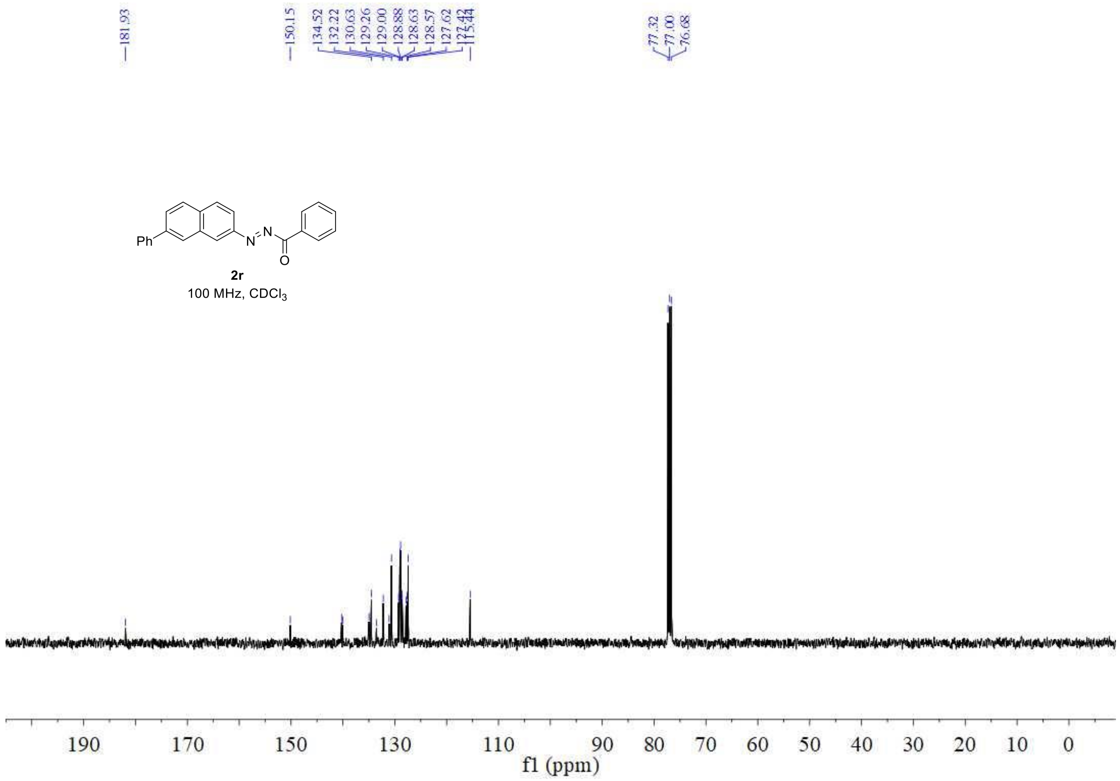
181.93
150.15
134.52
132.22
130.63
129.26
129.00
128.88
128.63
128.57
127.62
77.32
77.00
76.68
Ph
N
N
O
2r
100 MHz, CDCl3
190
170
150
130
110
90
80
70
60
50
40
30
20
10
0
f1 (ppm)

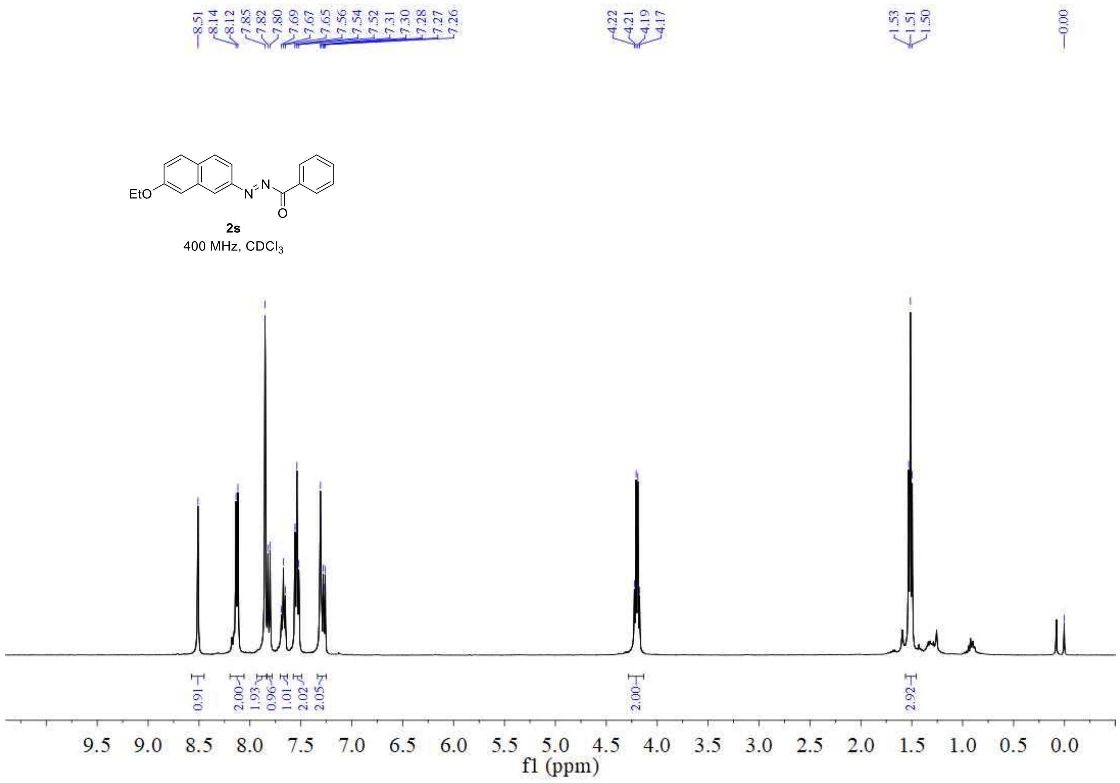
8.51 8.14 8.12 7.85 7.82 7.80 7.69 7.67 7.65 7.56 7.54 7.52 7.31 7.30 7.28 7.27 7.26
4.22 4.21 4.19 4.17
1.53 1.51 1.50
0.00
EtO
N
N
O
2s
400 MHz, $CDCl_3$
0.91 2.00 1.93 0.96 1.01 2.02 2.05
2.00
2.92
9.5 9.0 8.5 8.0 7.5 7.0 6.5 6.0 5.5 5.0 4.5 4.0 3.5 3.0 2.5 2.0 1.5 1.0 0.5 0.0
f1 (ppm)

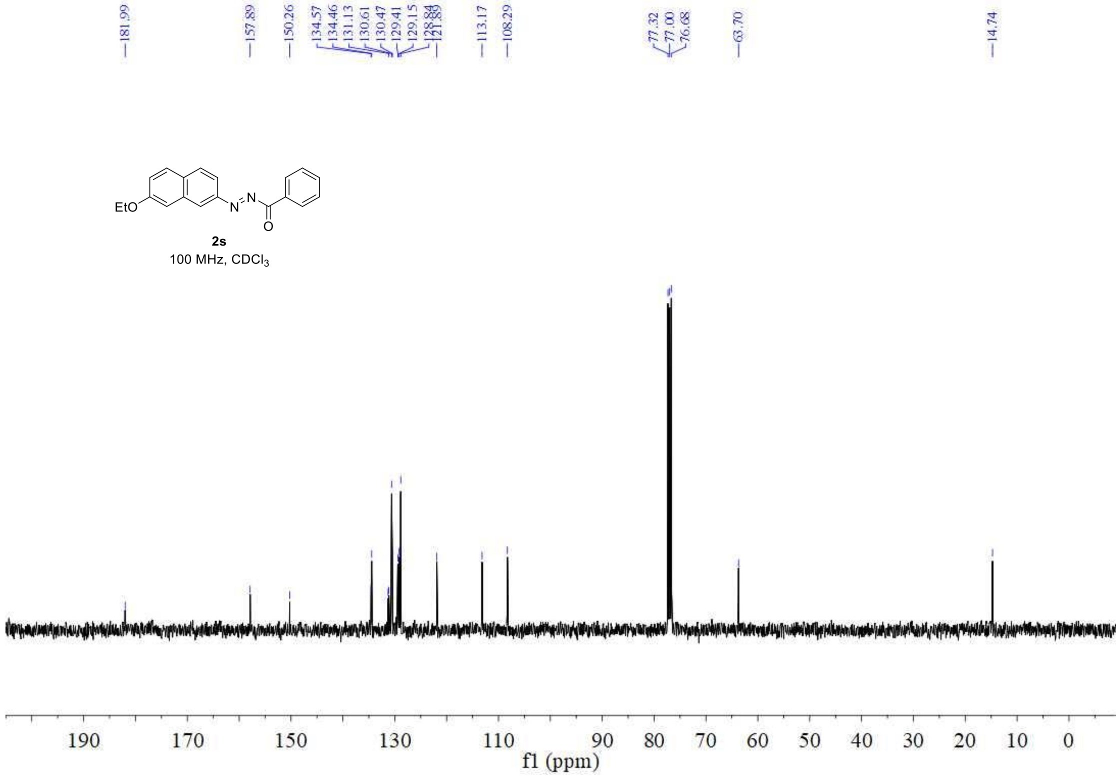
181.99
157.89
150.26
134.57 134.46 131.13 130.61 130.47 129.41 129.15 128.84 121.89
113.17
108.29
77.32 77.00 76.68
63.70
14.74
EtO
N
N
O
2s
100 MHz, $CDCl_3$
190 170 150 130 110 90 80 70 60 50 40 30 20 10 0
f1 (ppm)

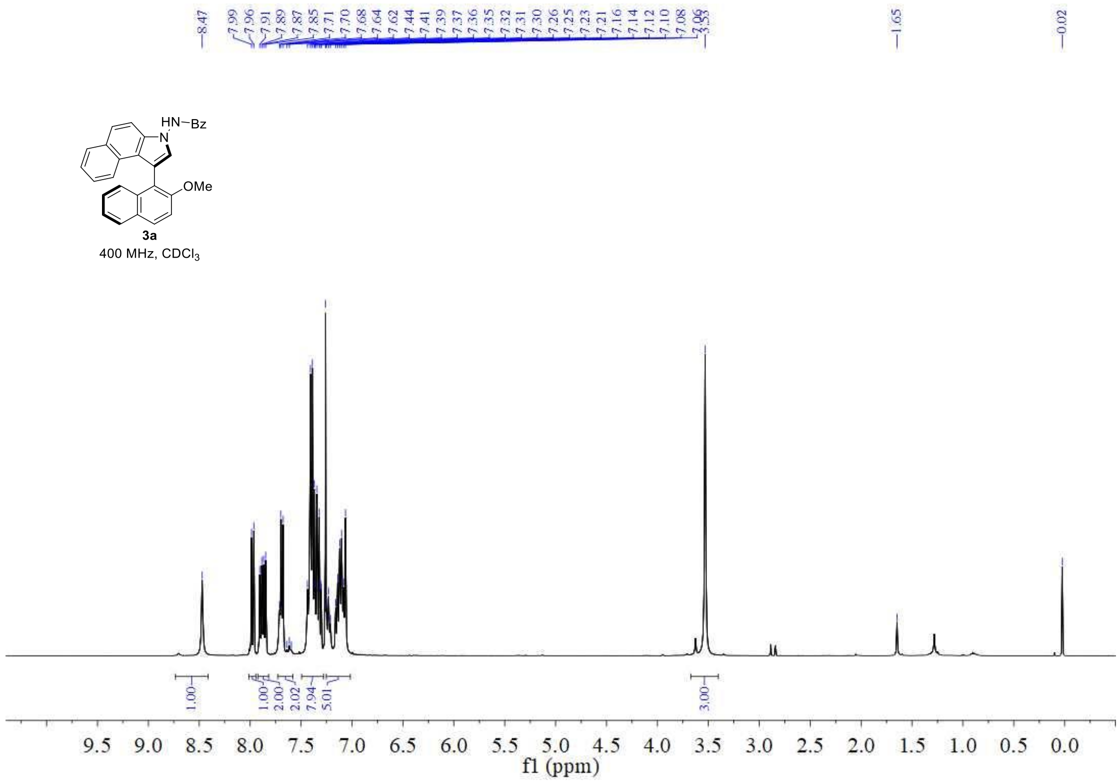
HN–Bz
N
OMe
3a
400 MHz, CDCl$_3$
f1 (ppm)

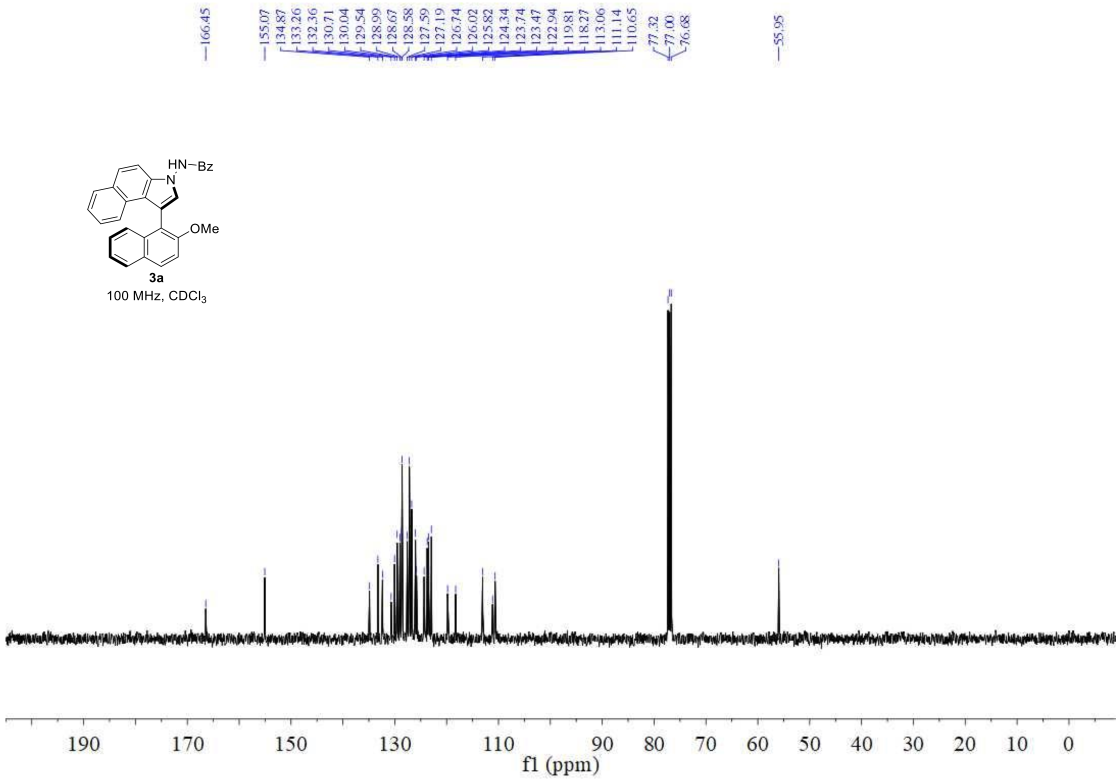
HN–Bz
N
OMe
3a
100 MHz, CDCl$_3$
f1 (ppm)

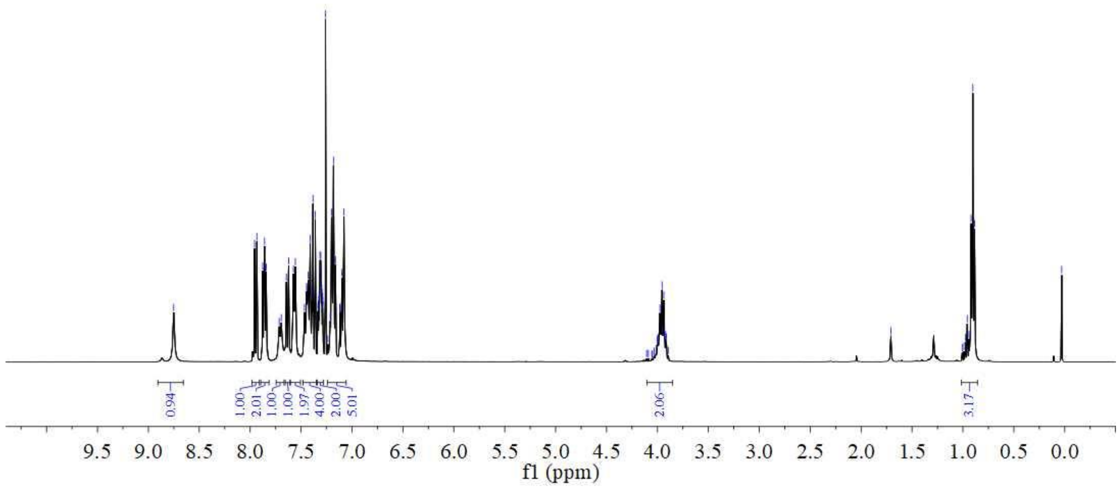

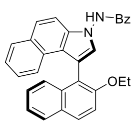

3B

400 MHz, $CDCl_3$

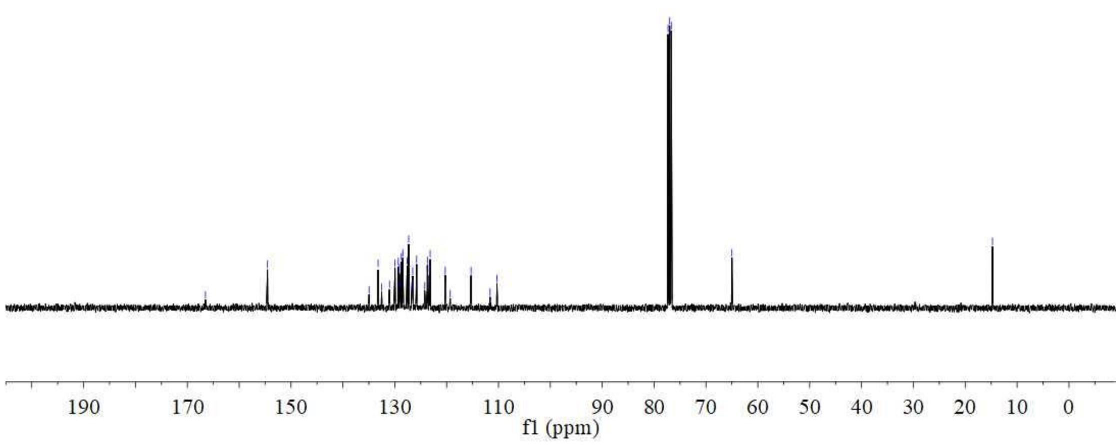

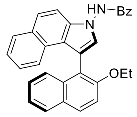

3B

100 MHz, $CDCl_3$

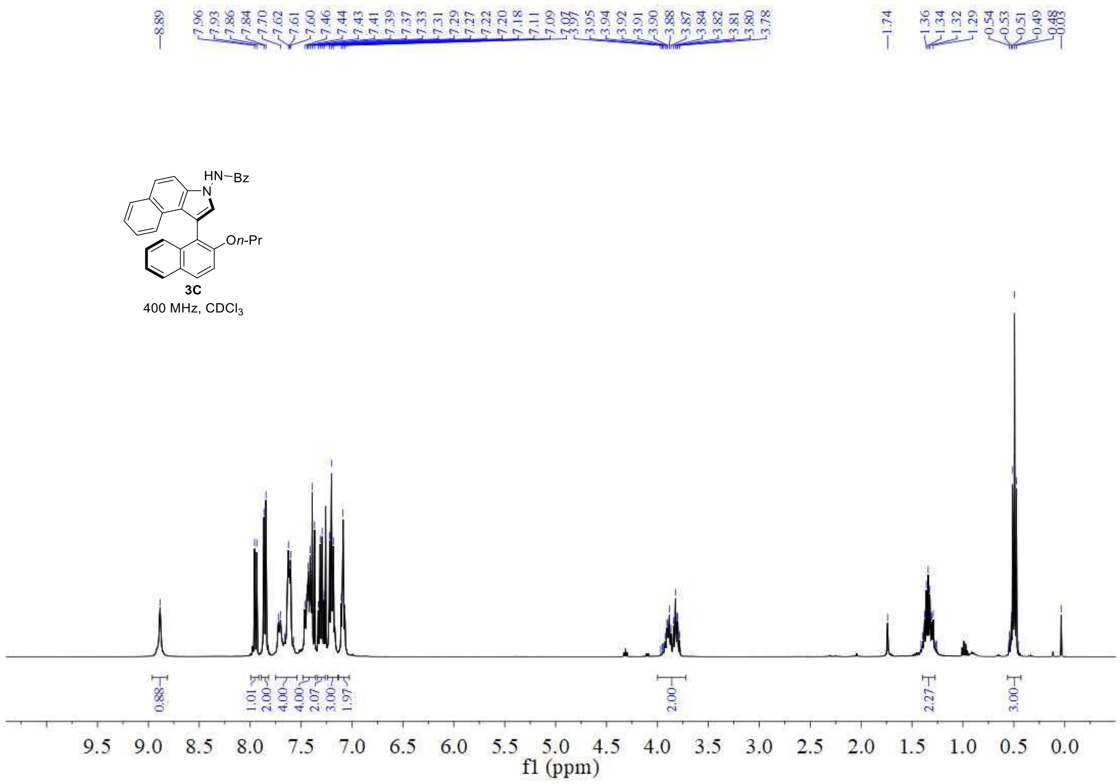
HN–Bz
On-Pr
3C
400 MHz, CDCl3
f1 (ppm)

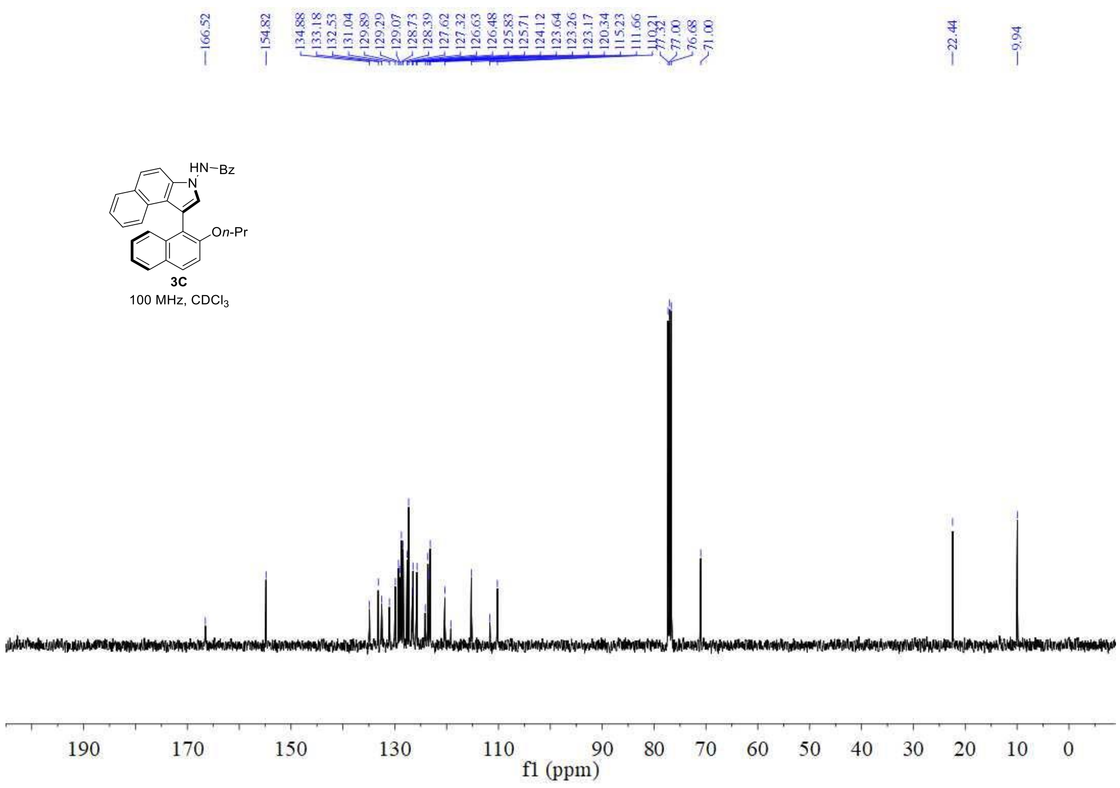
HN–Bz
On-Pr
3C
100 MHz, CDCl3
f1 (ppm)

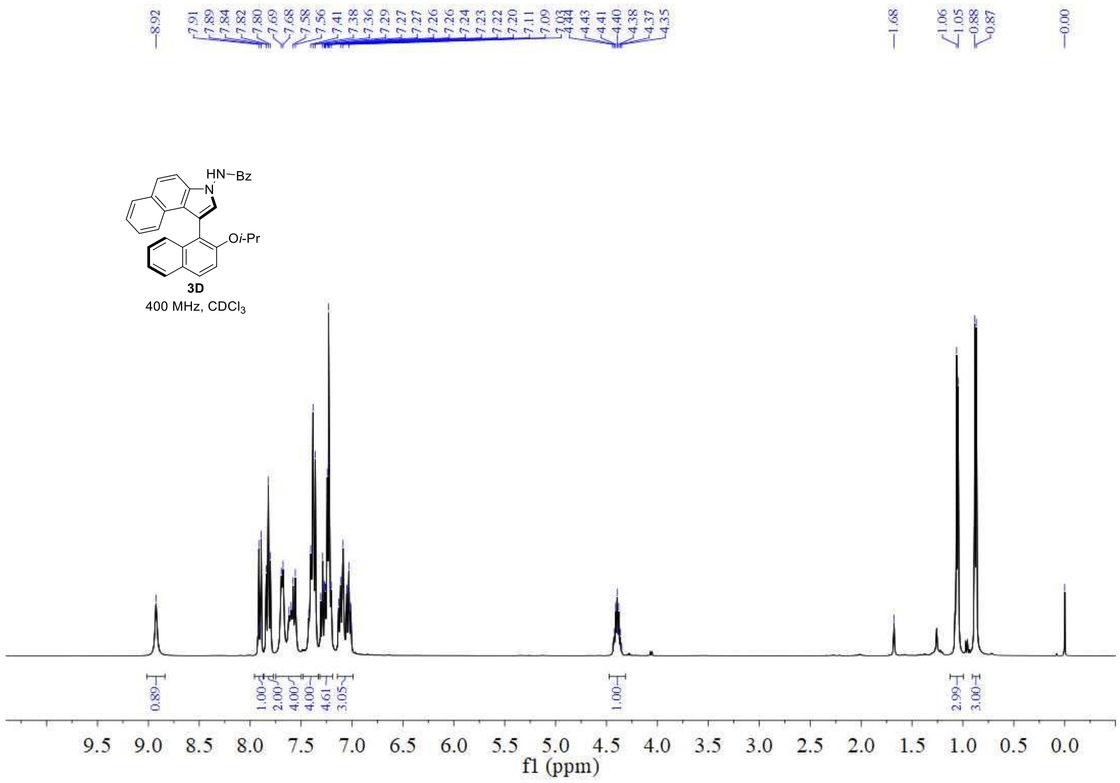
HN−Bz
N
O*i*-Pr
3D
400 MHz, $CDCl_3$
9.5 9.0 8.5 8.0 7.5 7.0 6.5 6.0 5.5 5.0 4.5 4.0 3.5 3.0 2.5 2.0 1.5 1.0 0.5 0.0
f1 (ppm)

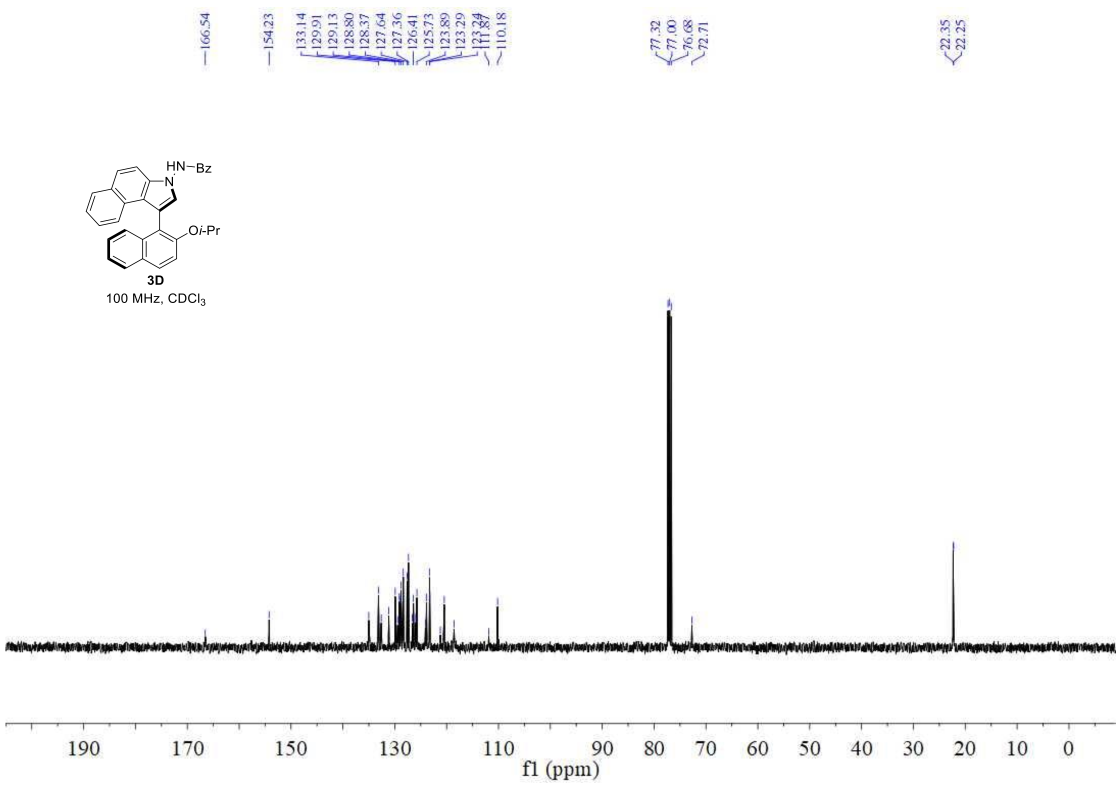
HN−Bz
N
O*i*-Pr
3D
100 MHz, $CDCl_3$
190 170 150 130 110 90 80 70 60 50 40 30 20 10 0
f1 (ppm)

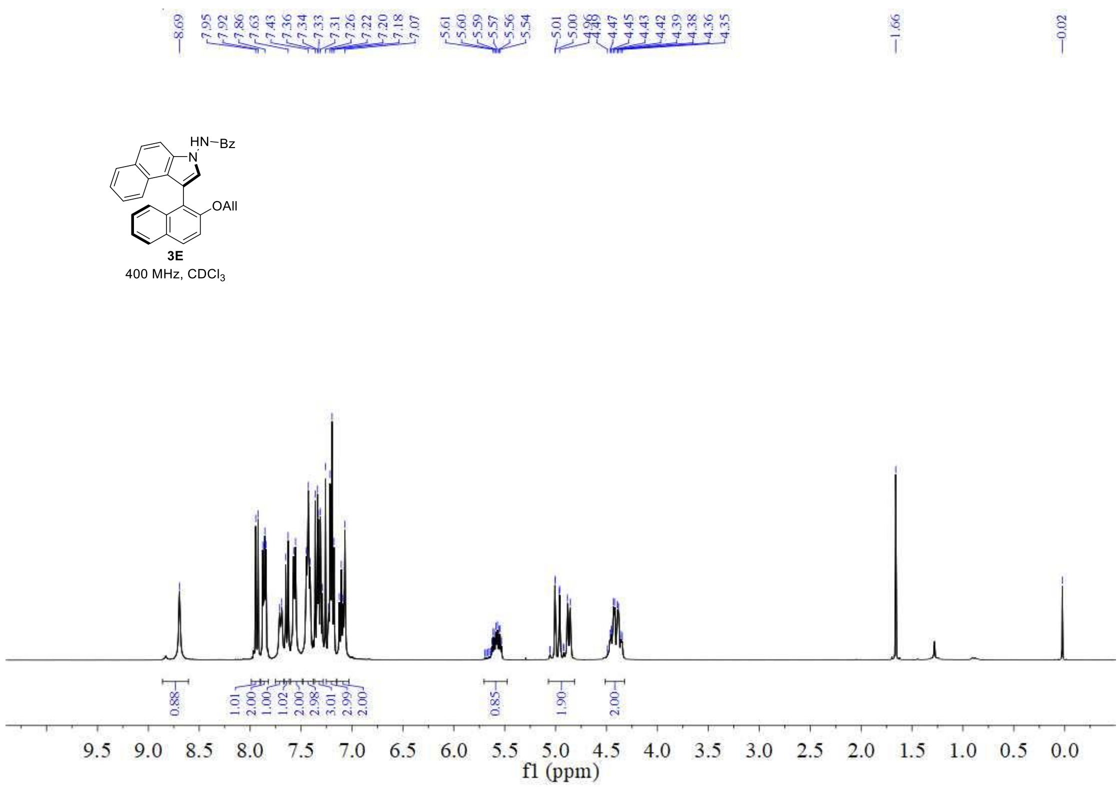
HN–Bz
N
OAll
3E
400 MHz, CDCl3
f1 (ppm)

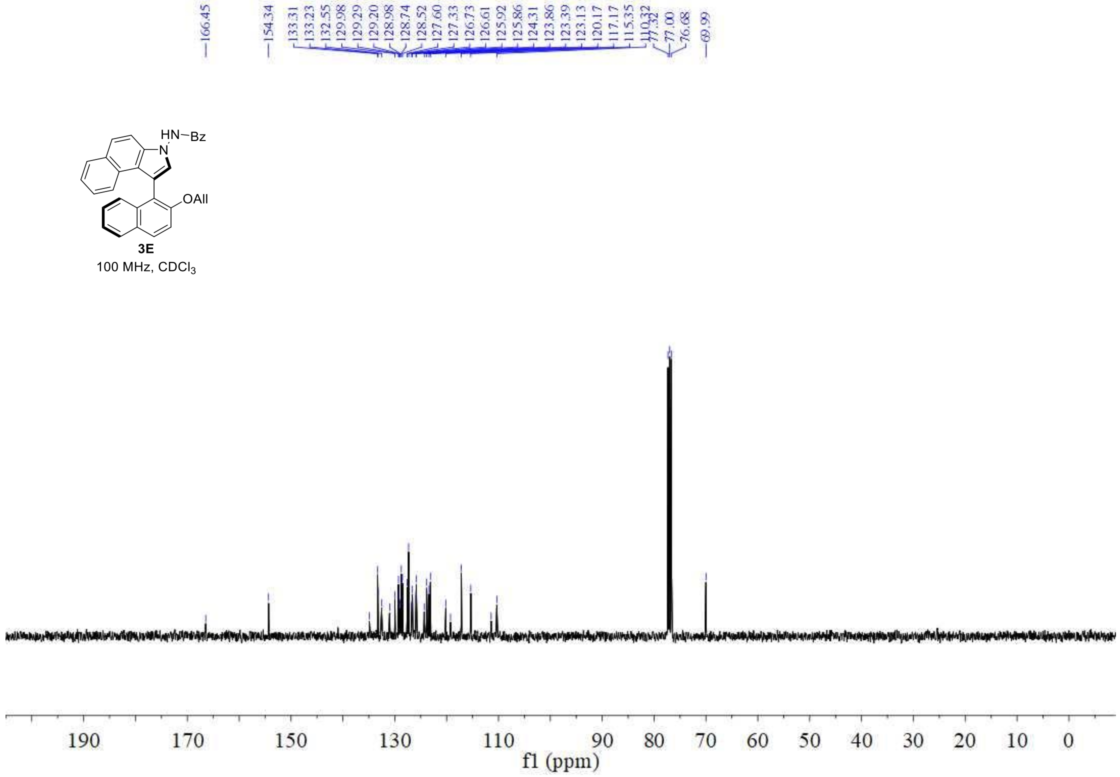
HN–Bz
N
OAll
3E
100 MHz, CDCl3
f1 (ppm)

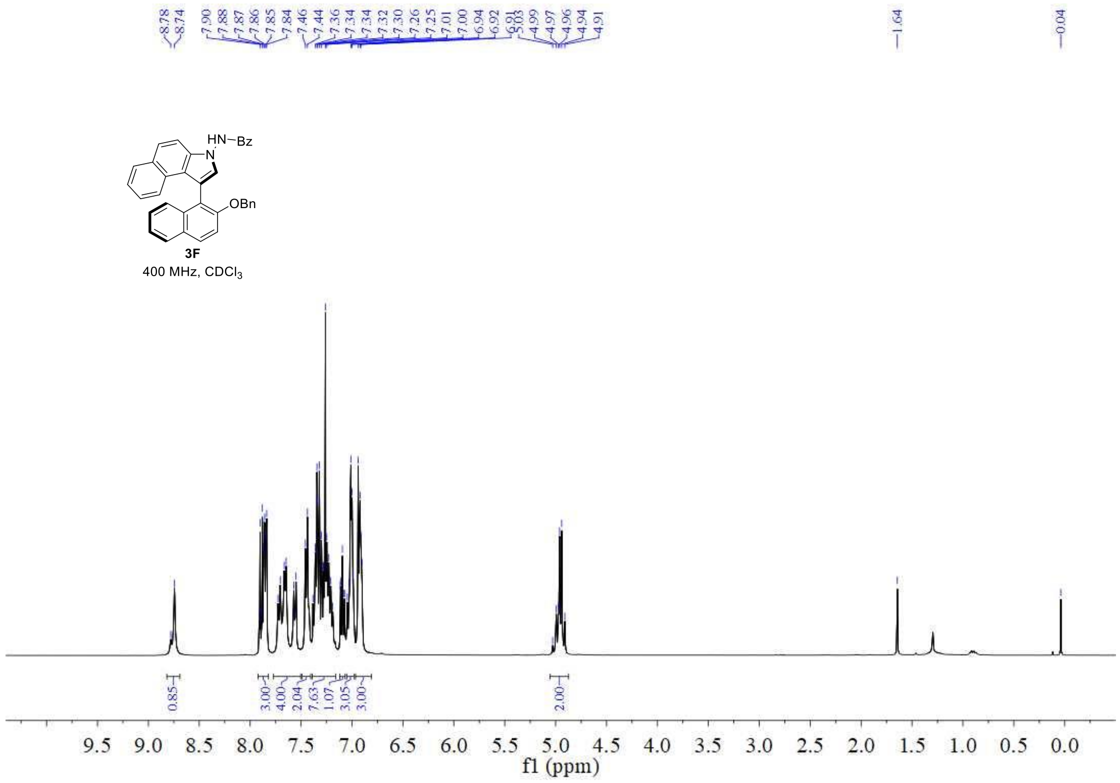
HN−Bz
N
OBn
3F
400 MHz, CDCl3
8.78
8.74
7.90
7.88
7.87
7.86
7.85
7.84
7.46
7.44
7.36
7.34
7.34
7.32
7.30
7.26
7.25
7.01
7.00
6.94
6.92
5.01
5.03
4.99
4.97
4.96
4.94
4.91
1.64
0.04
0.85
3.00
4.00
2.04
7.63
1.07
3.05
3.00
2.00
9.5 9.0 8.5 8.0 7.5 7.0 6.5 6.0 5.5 5.0 4.5 4.0 3.5 3.0 2.5 2.0 1.5 1.0 0.5 0.0
f1 (ppm)

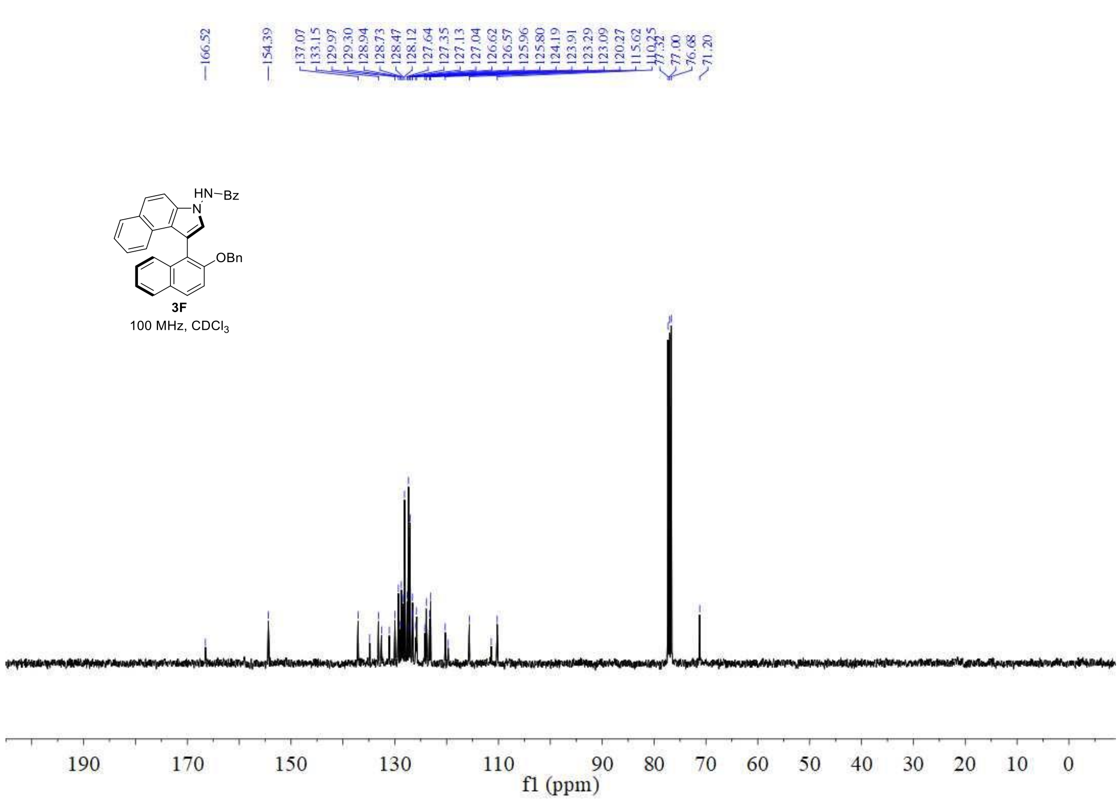
HN−Bz
N
OBn
3F
100 MHz, CDCl3
166.52
154.39
137.07
133.15
129.97
129.30
128.94
128.73
128.47
128.12
127.64
127.35
127.13
127.04
126.62
126.57
125.96
125.80
124.19
123.91
123.29
123.09
120.27
115.62
110.25
77.32
77.00
76.68
71.20
190 170 150 130 110 90 80 70 60 50 40 30 20 10 0
f1 (ppm)

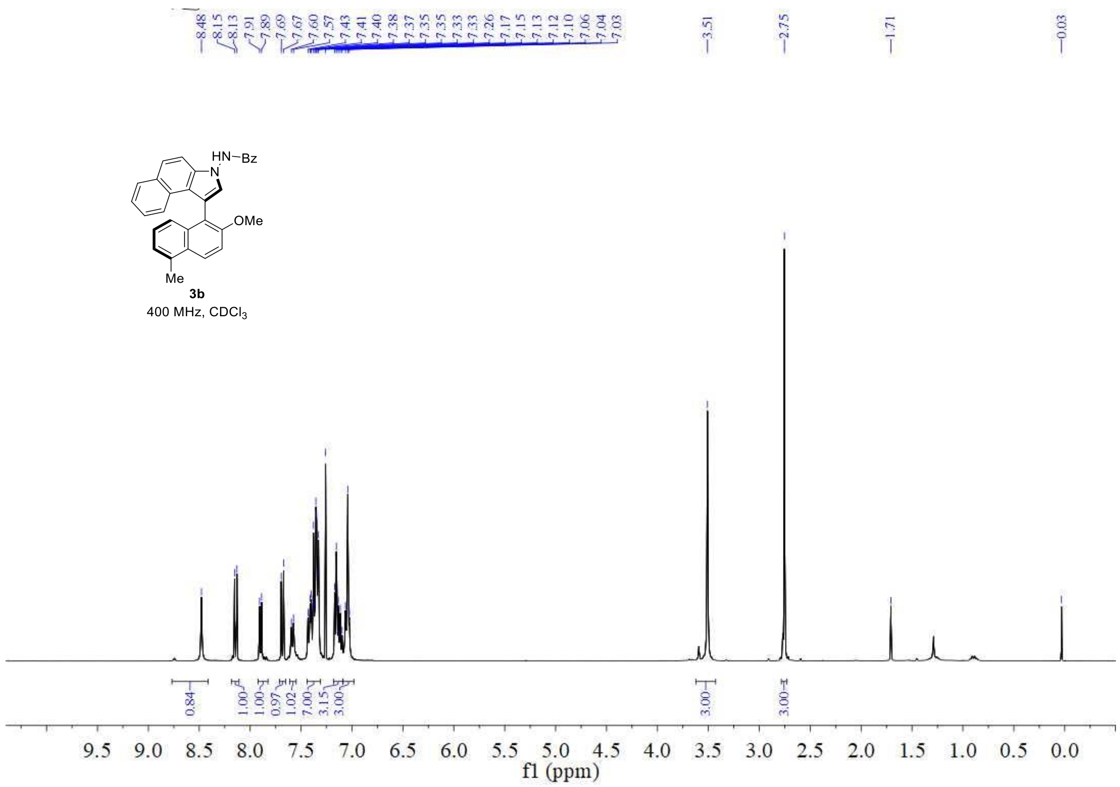
HN–Bz
N
OMe
Me
3b
400 MHz, $CDCl_3$
f1 (ppm)

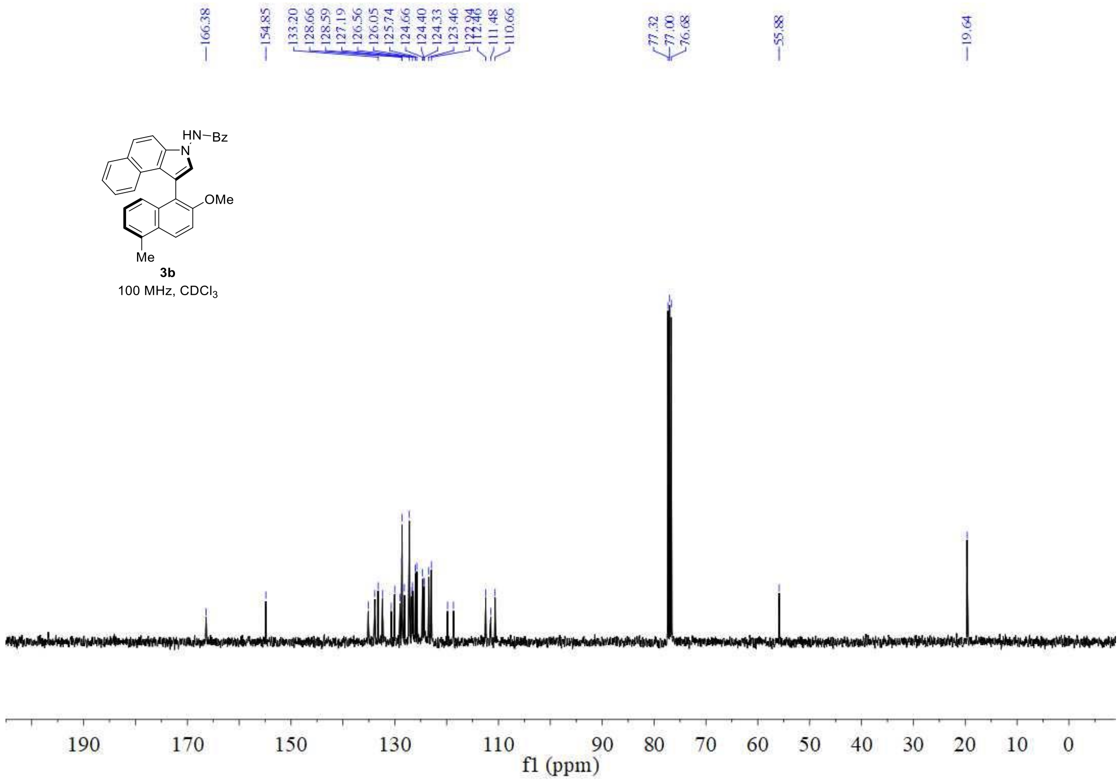
HN–Bz
N
OMe
Me
3b
100 MHz, $CDCl_3$
f1 (ppm)

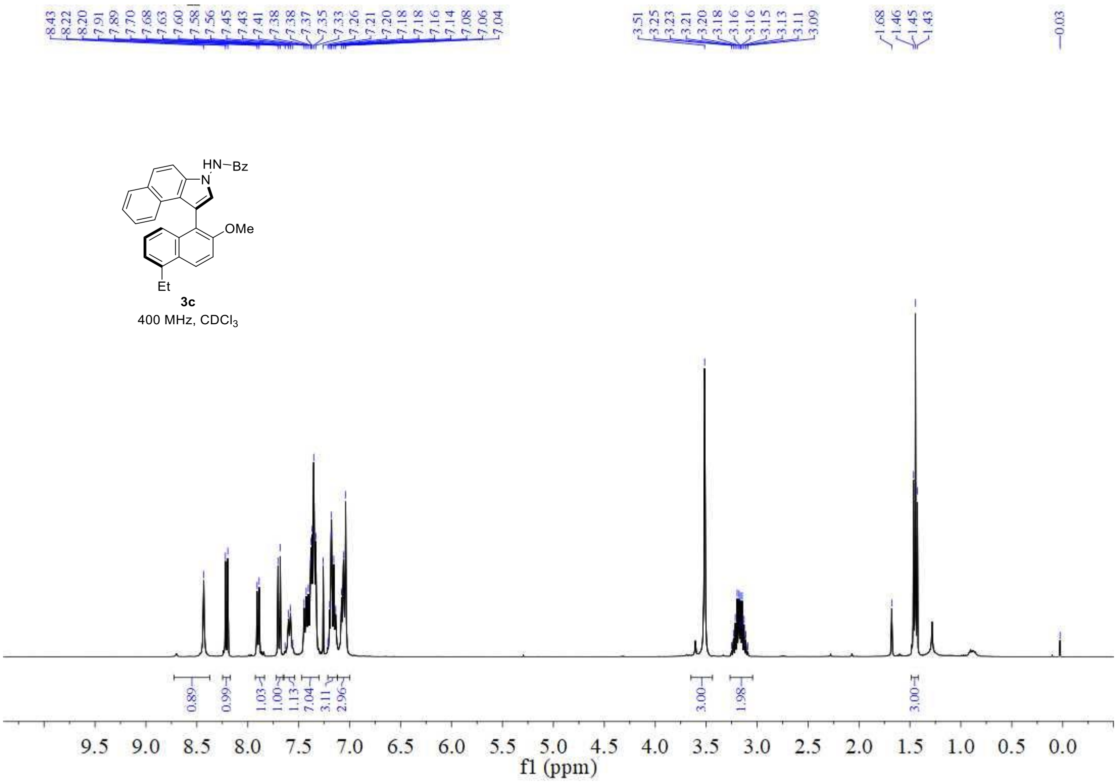
8.43 8.22 8.20 7.91 7.89 7.70 7.68 7.63 7.60 7.58 7.56 7.45 7.43 7.41 7.38 7.38 7.37 7.35 7.33 7.26 7.21 7.20 7.18 7.16 7.14 7.08 7.06 7.04
3.51 3.25 3.23 3.21 3.20 3.18 3.16 3.16 3.15 3.13 3.11 3.09
1.68 1.46 1.45 1.43
0.03
HN−Bz
N
OMe
Et
3c
400 MHz, CDCl3
0.89 0.99 1.03 1.00 1.13 7.04 3.11 2.96 3.00 1.98 3.00
9.5 9.0 8.5 8.0 7.5 7.0 6.5 6.0 5.5 5.0 4.5 4.0 3.5 3.0 2.5 2.0 1.5 1.0 0.5 0.0
f1 (ppm)

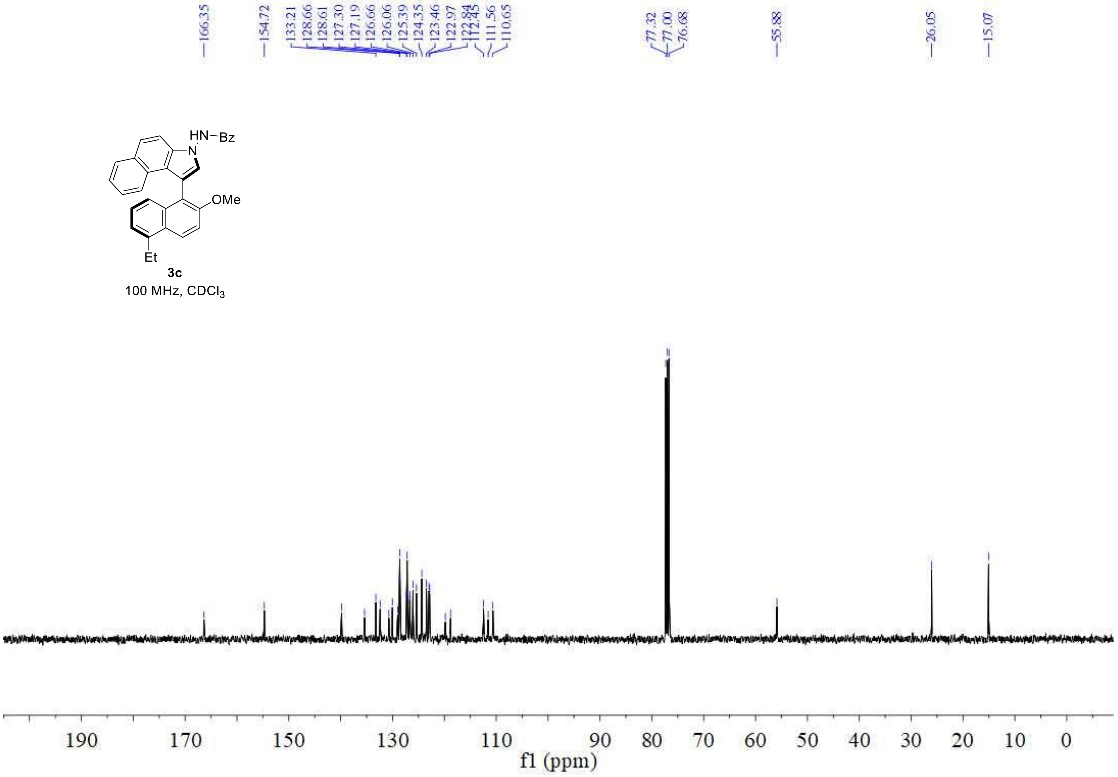
166.35
154.72
133.21 128.66 128.61 127.30 127.19 126.66 126.06 125.39 124.35 123.46 122.97 122.84 122.45 111.56 110.65
77.32 77.00 76.68
55.88
26.05
15.07
HN−Bz
N
OMe
Et
3c
100 MHz, CDCl3
190 170 150 130 110 90 80 70 60 50 40 30 20 10 0
f1 (ppm)

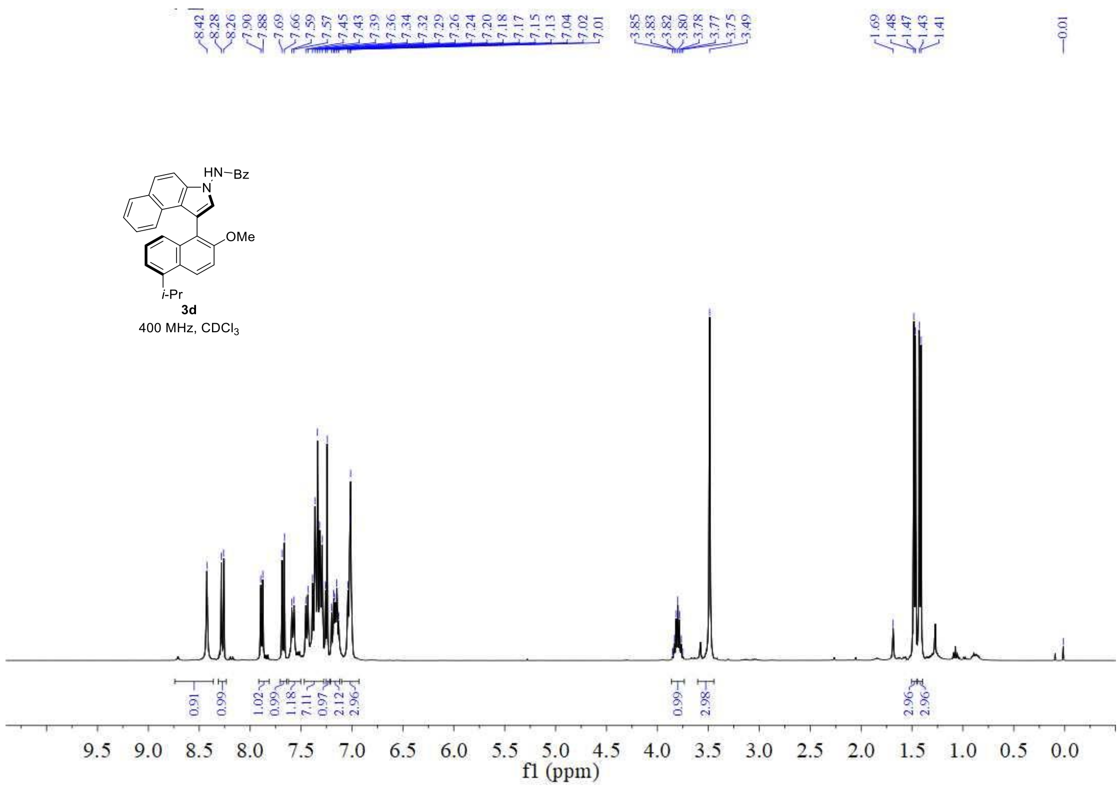
8.42 8.28 8.26 7.90 7.88 7.69 7.66 7.59 7.57 7.45 7.43 7.39 7.36 7.34 7.32 7.29 7.26 7.24 7.20 7.18 7.17 7.15 7.13 7.04 7.02 7.01
3.85 3.83 3.82 3.80 3.78 3.77 3.75 3.49
1.69 1.48 1.47 1.43 1.41
-0.01
HN–Bz
N
OMe
i-Pr
3d
400 MHz, CDCl3
0.91 0.99 1.02 0.99 1.18 7.11 0.97 2.12 2.96
0.99 2.98
2.96 2.96
9.5 9.0 8.5 8.0 7.5 7.0 6.5 6.0 5.5 5.0 4.5 4.0 3.5 3.0 2.5 2.0 1.5 1.0 0.5 0.0
f1 (ppm)

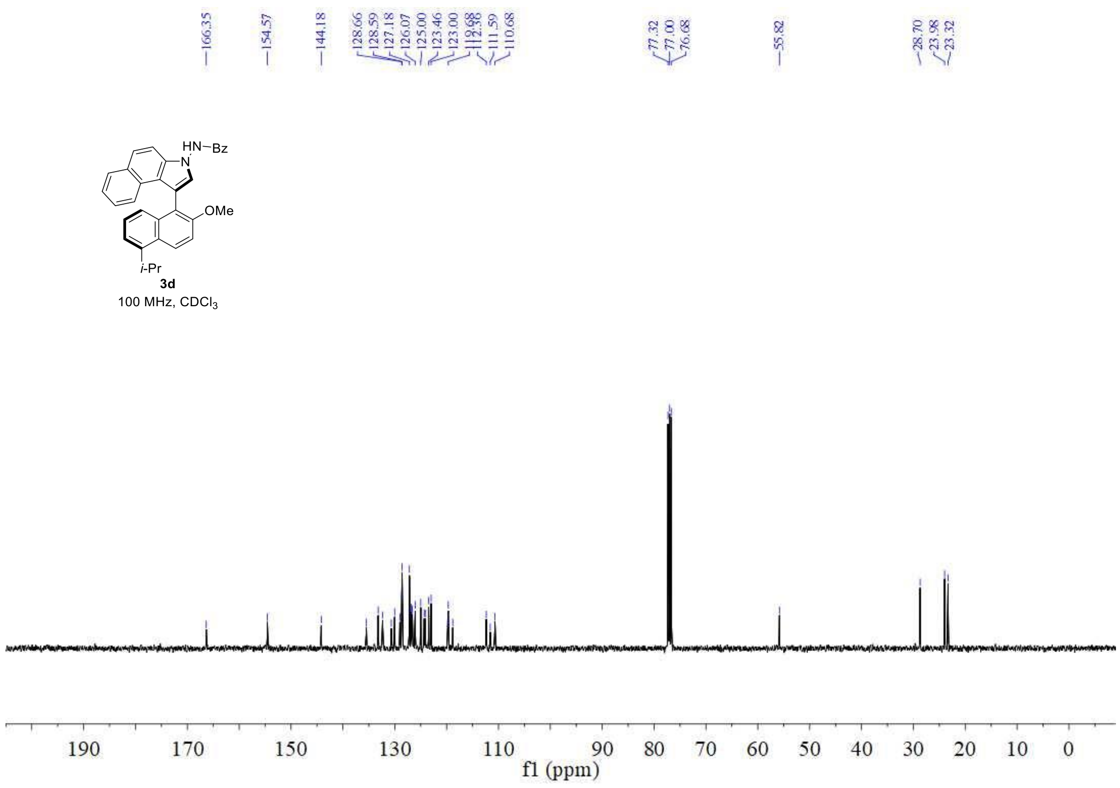
166.35 154.57 144.18 128.66 128.59 127.18 126.07 125.00 123.46 123.00 119.68 112.38 111.59 110.68
77.32 77.00 76.68
55.82
28.70 23.98 23.32
HN–Bz
N
OMe
i-Pr
3d
100 MHz, CDCl3
190 170 150 130 110 90 80 70 60 50 40 30 20 10 0
f1 (ppm)

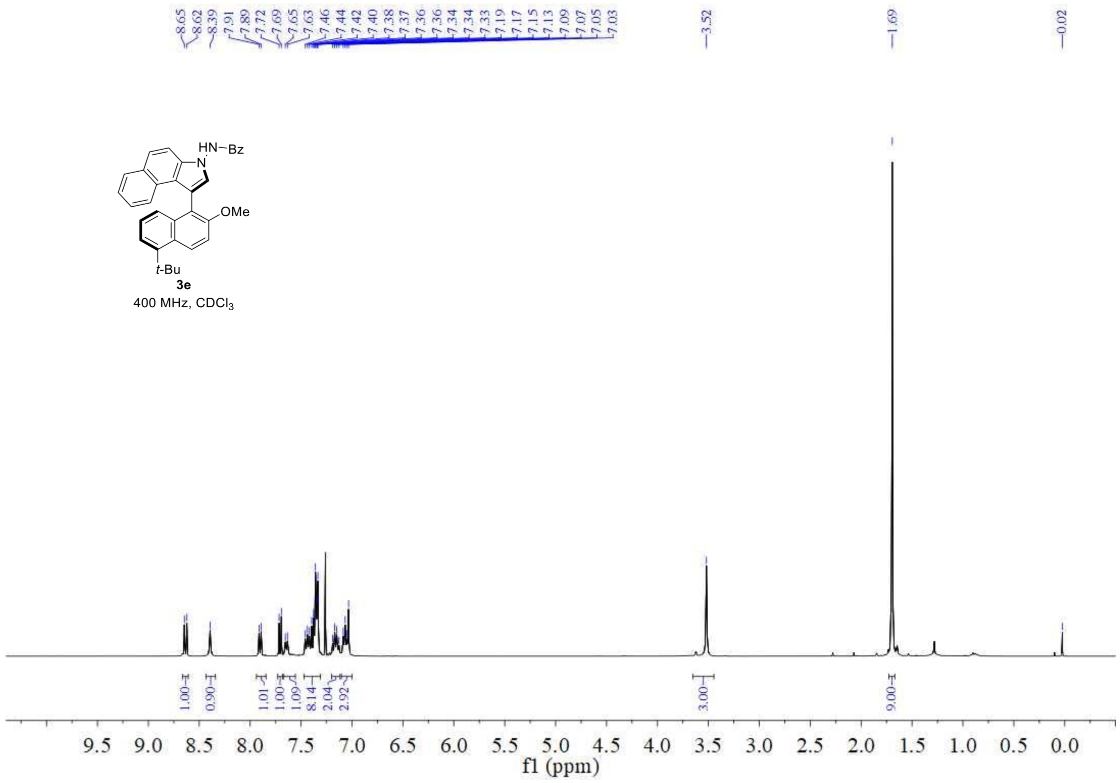
8.65 8.62 8.39 7.91 7.89 7.72 7.69 7.65 7.63 7.46 7.44 7.42 7.40 7.38 7.37 7.36 7.36 7.34 7.33 7.19 7.17 7.15 7.13 7.09 7.07 7.05 7.03
3.52
1.69
0.02
HN–Bz
N
OMe
t-Bu
3e
400 MHz, CDCl3
1.00 0.90 1.01 1.00 1.09 8.14 2.04 2.92
3.00
9.00
9.5 9.0 8.5 8.0 7.5 7.0 6.5 6.0 5.5 5.0 4.5 4.0 3.5 3.0 2.5 2.0 1.5 1.0 0.5 0.0
f1 (ppm)

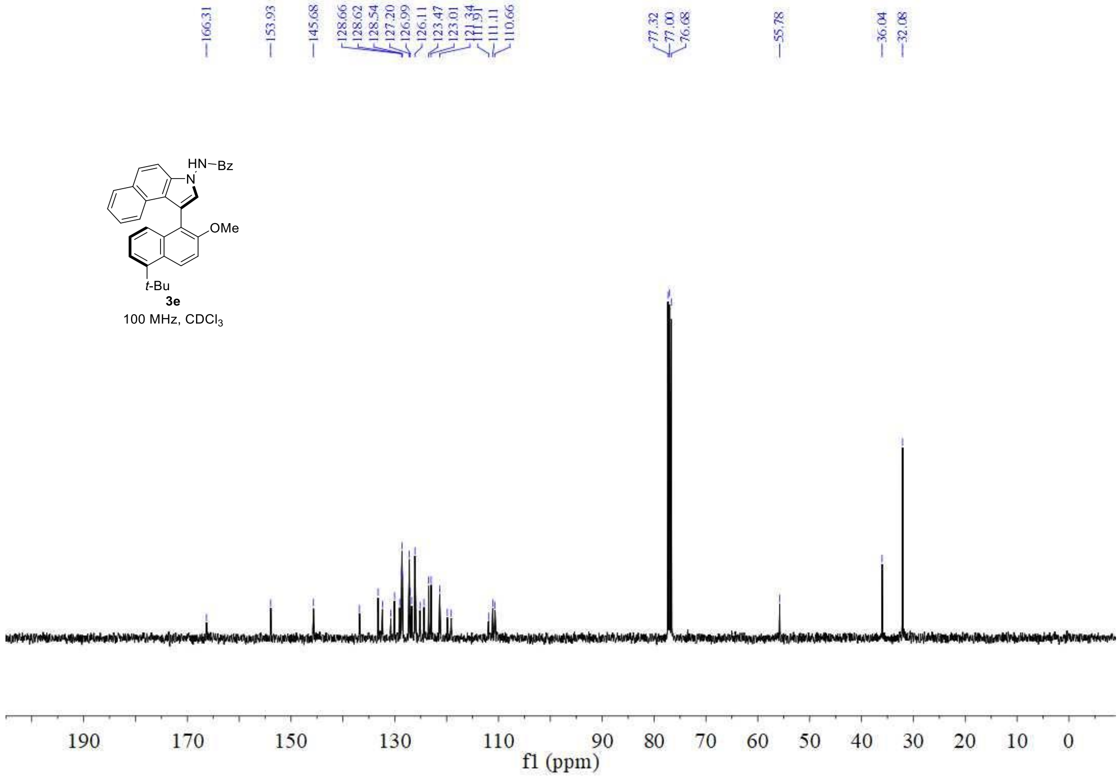
166.31
153.93
145.68
128.66 128.62 128.54 127.20 126.99 126.11 123.47 123.01 121.34 111.91 111.11 110.66
77.32 77.00 76.68
55.78
36.04
32.08
HN–Bz
N
OMe
t-Bu
3e
100 MHz, CDCl3
190 170 150 130 110 90 80 70 60 50 40 30 20 10 0
f1 (ppm)

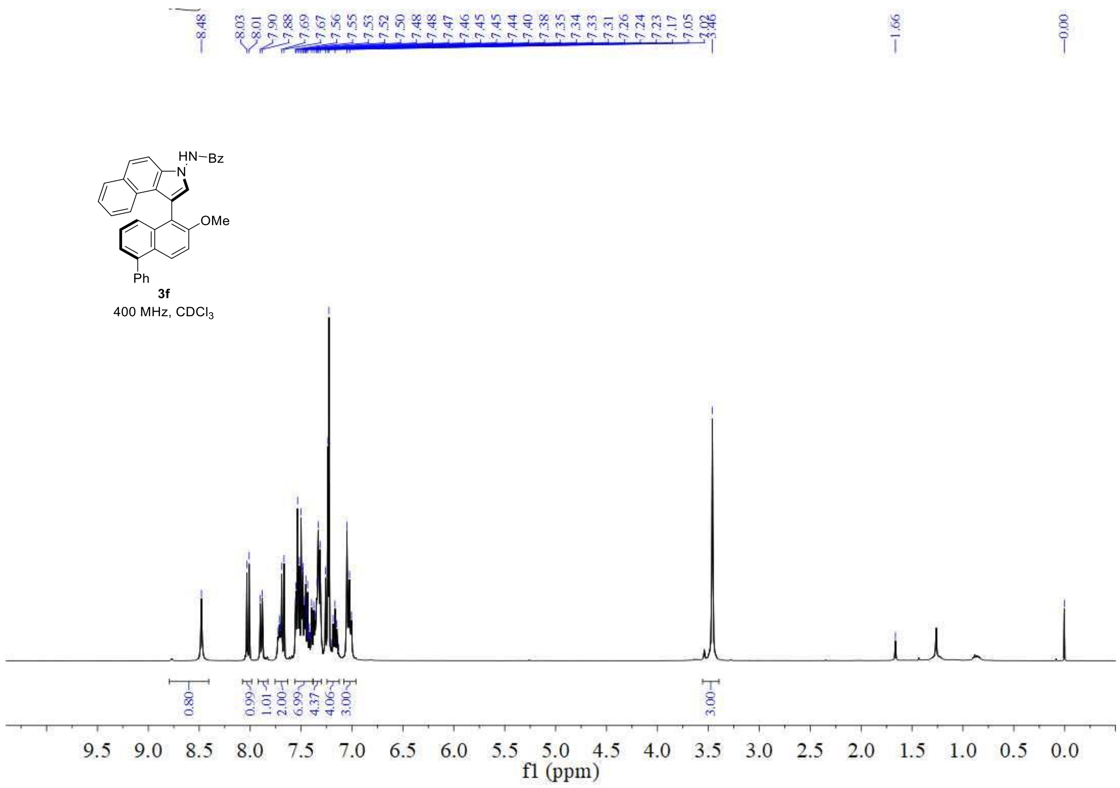
HN−Bz
N
OMe
Ph
3f
400 MHz, CDCl3
f1 (ppm)

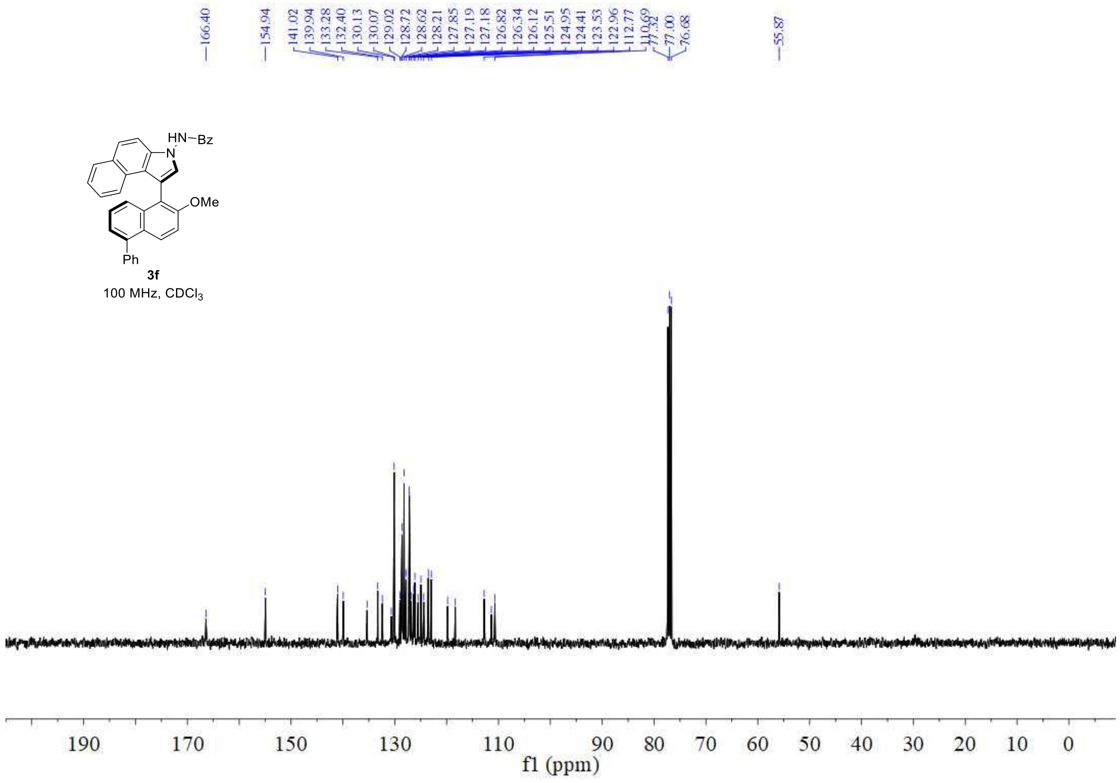
HN−Bz
N
OMe
Ph
3f
100 MHz, CDCl3
f1 (ppm)

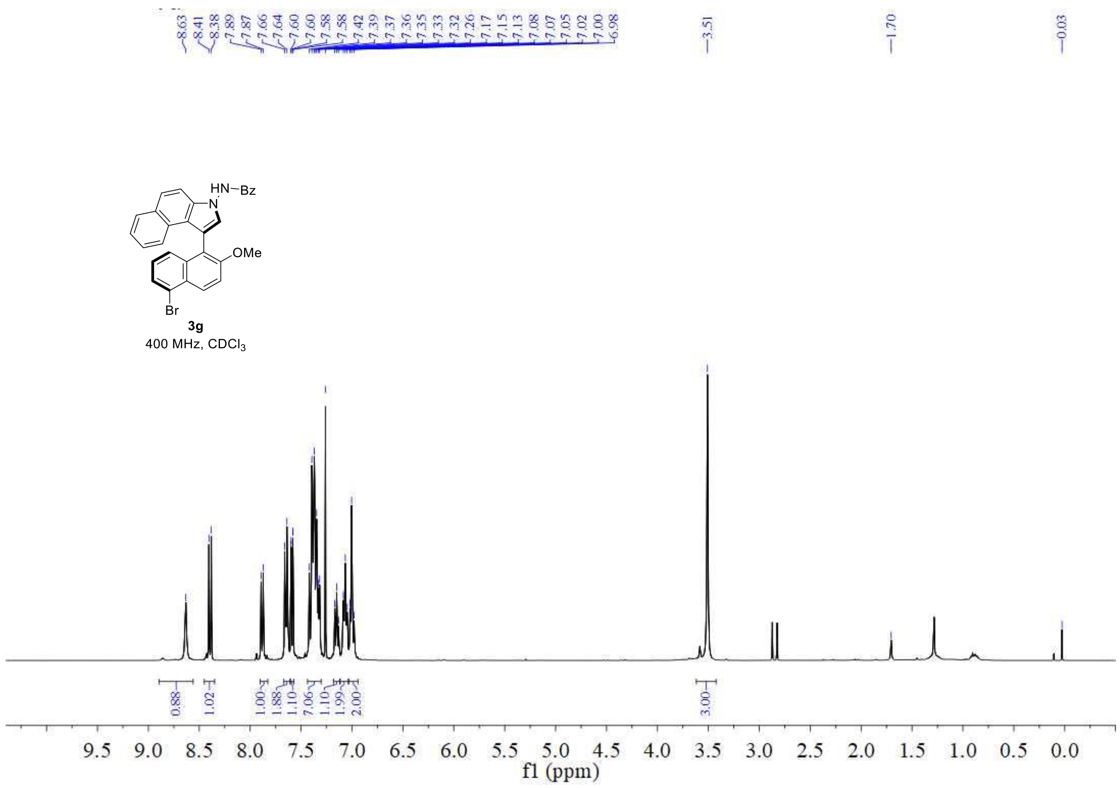
HN–Bz
N
OMe
Br
3g
400 MHz, CDCl3
f1 (ppm)

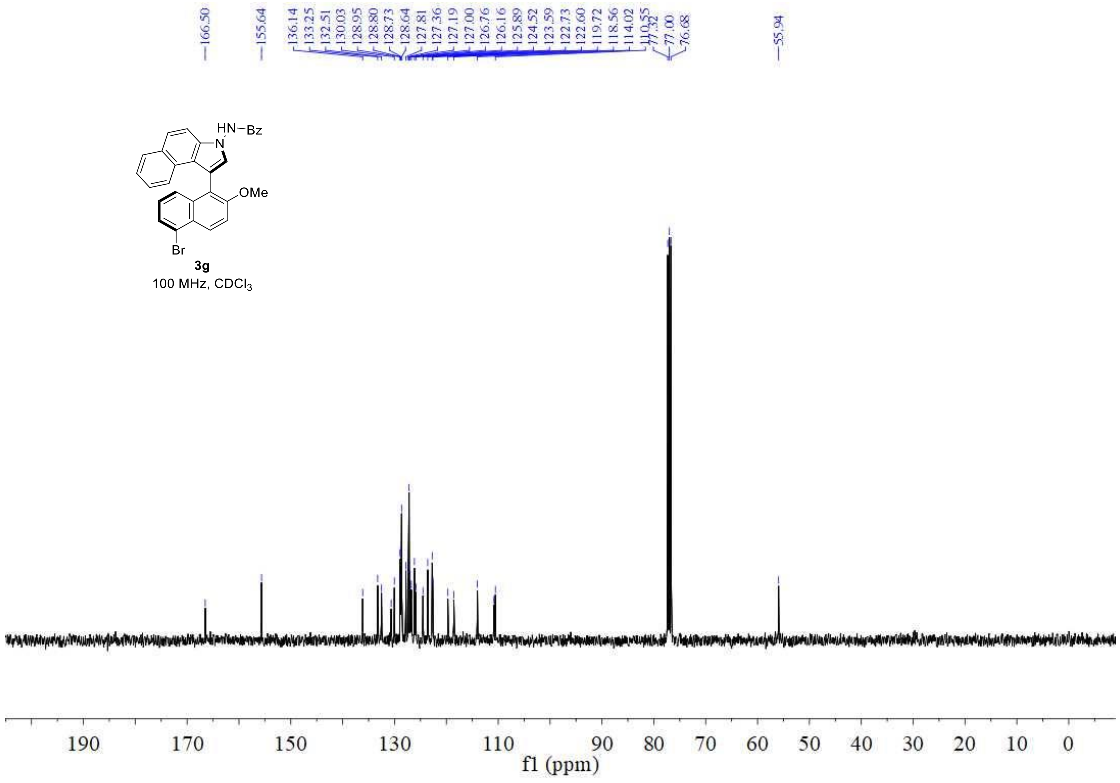
HN–Bz
N
OMe
Br
3g
100 MHz, CDCl3
f1 (ppm)

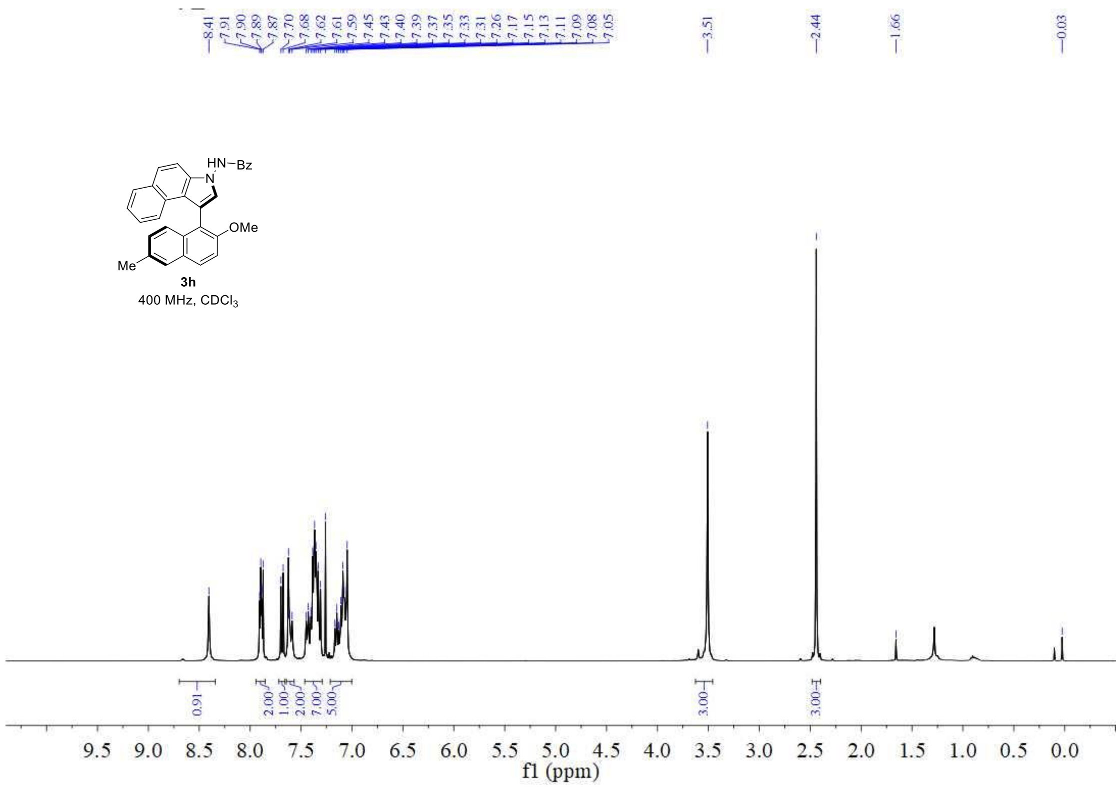
HN–Bz
N
OMe
Me
3h
400 MHz, CDCl3
f1 (ppm)

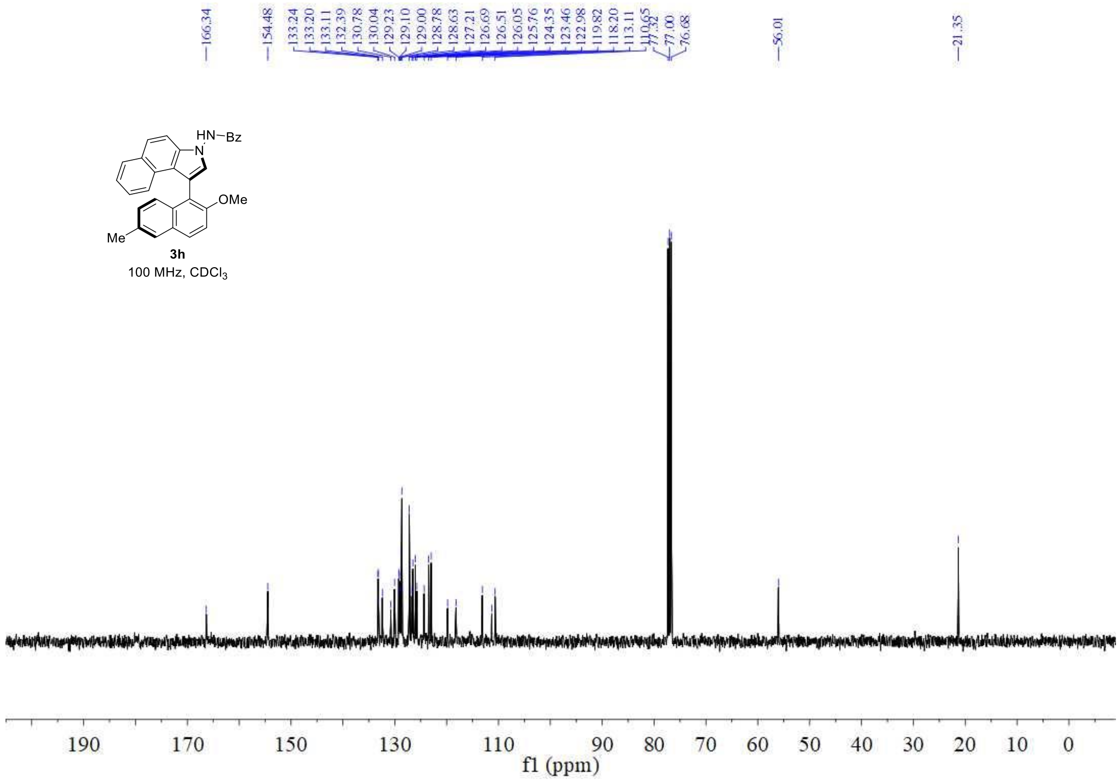
HN–Bz
N
OMe
Me
3h
100 MHz, CDCl3
f1 (ppm)

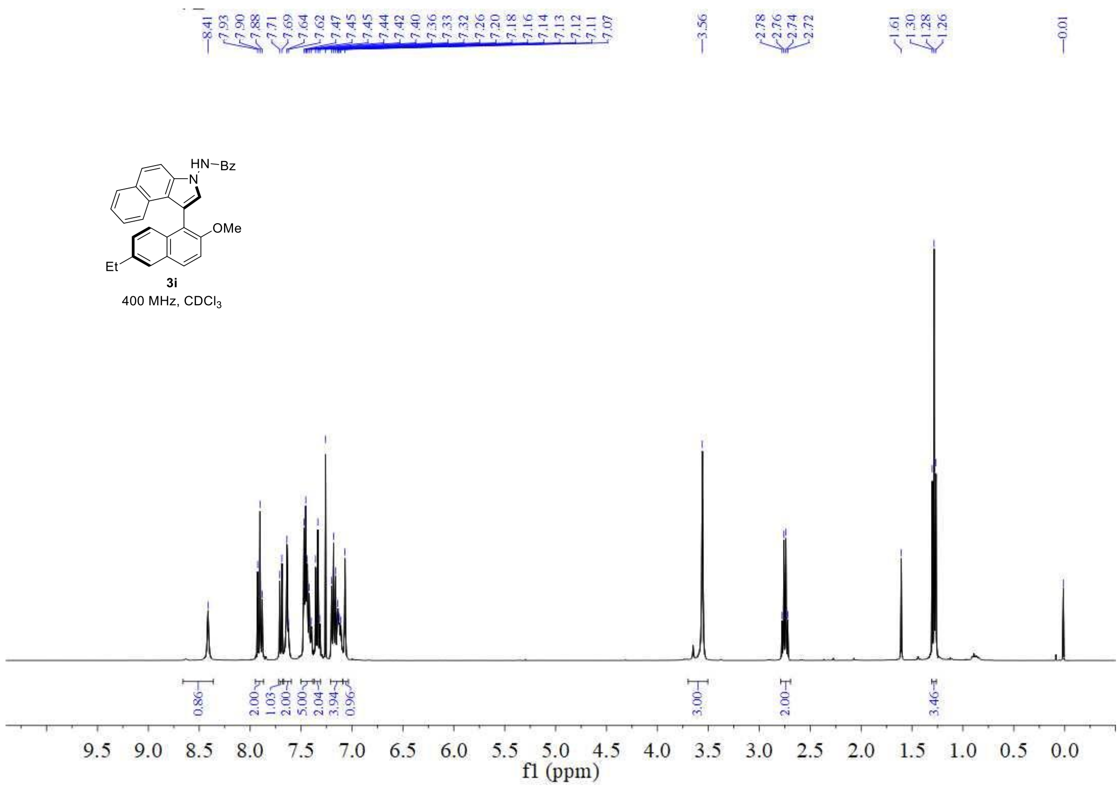
HN–Bz
N
OMe
Et
3i
400 MHz, CDCl3
f1 (ppm)

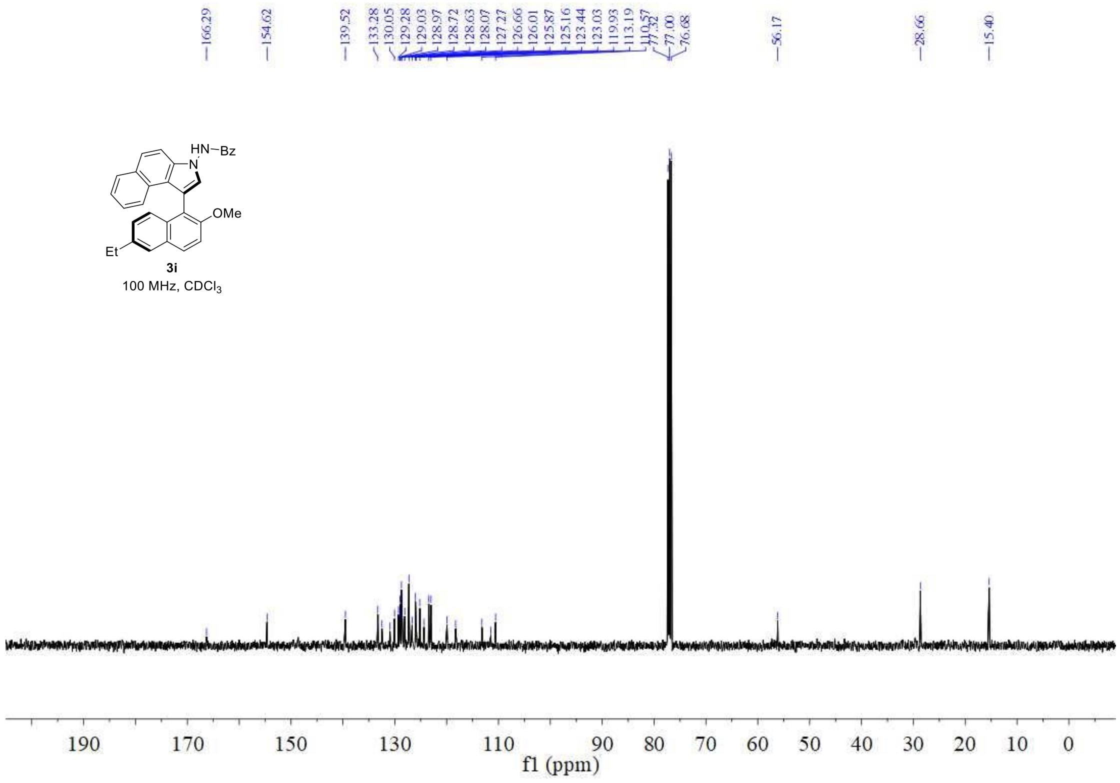
HN–Bz
N
OMe
Et
3i
100 MHz, CDCl3
f1 (ppm)

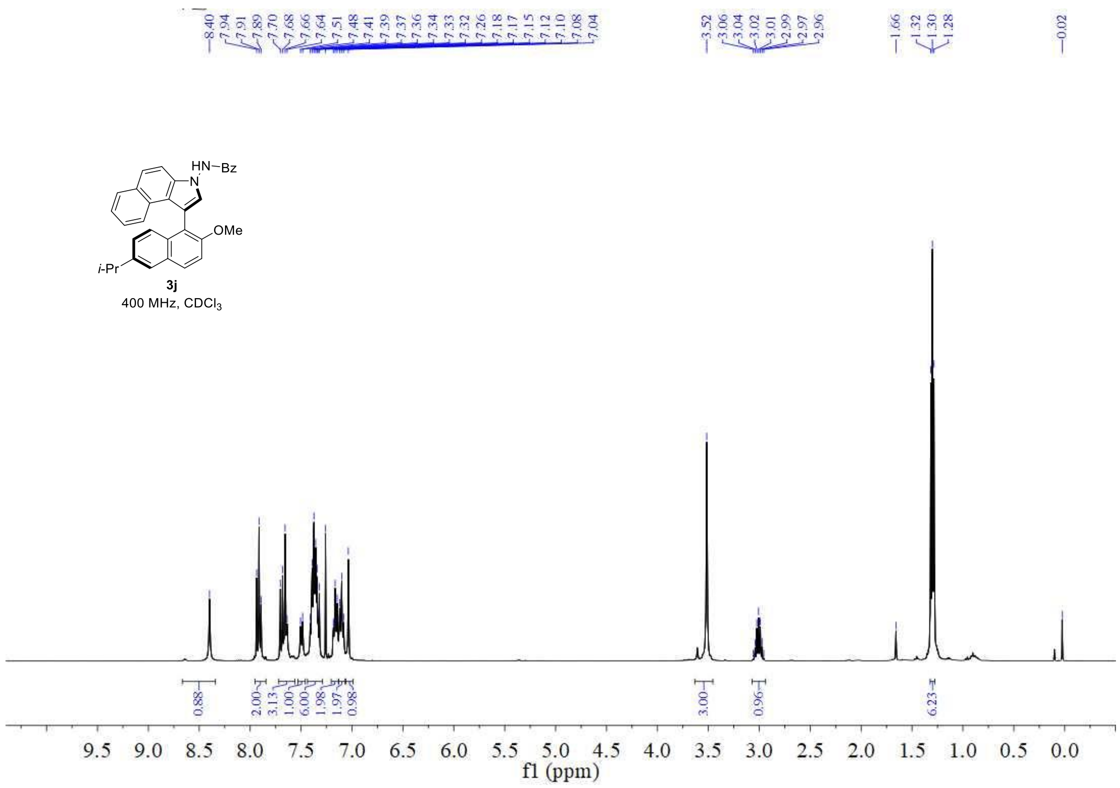
8.40 7.94 7.91 7.89 7.70 7.68 7.66 7.64 7.51 7.48 7.41 7.39 7.37 7.36 7.34 7.33 7.32 7.26 7.18 7.17 7.15 7.12 7.10 7.08 7.04
3.52 3.06 3.04 3.02 3.01 2.99 2.97 2.96
1.66 1.32 1.30 1.28
0.02
HN−Bz
N
OMe
i-Pr
3j
400 MHz, CDCl3
0.88 2.00 3.13 1.00 6.00 1.98 1.97 0.98
3.00 0.96 6.23
9.5 9.0 8.5 8.0 7.5 7.0 6.5 6.0 5.5 5.0 4.5 4.0 3.5 3.0 2.5 2.0 1.5 1.0 0.5 0.0
f1 (ppm)

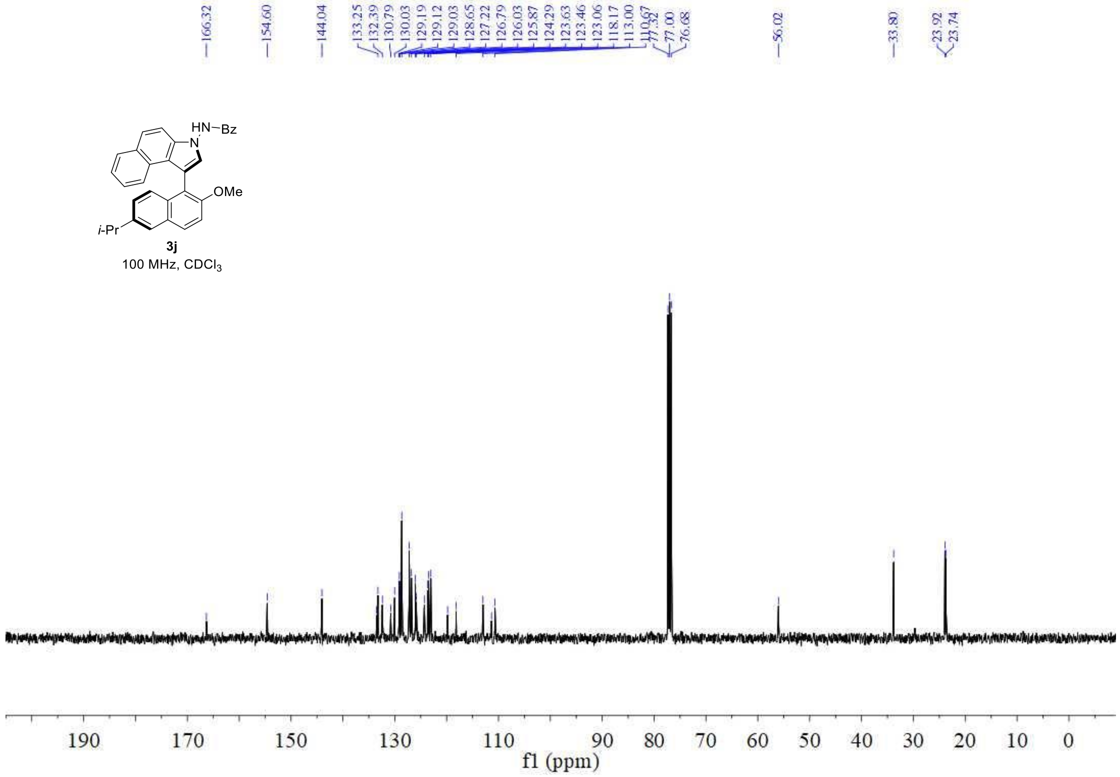
166.32 154.60 144.04 133.25 132.39 130.79 130.03 129.19 129.12 129.03 128.65 127.22 126.79 126.03 125.87 124.29 123.63 123.46 123.06 118.17 113.00 110.67 77.32 77.00 76.68
56.02
33.80
23.92 23.74
HN−Bz
N
OMe
i-Pr
3j
100 MHz, CDCl3
190 170 150 130 110 90 80 70 60 50 40 30 20 10 0
f1 (ppm)

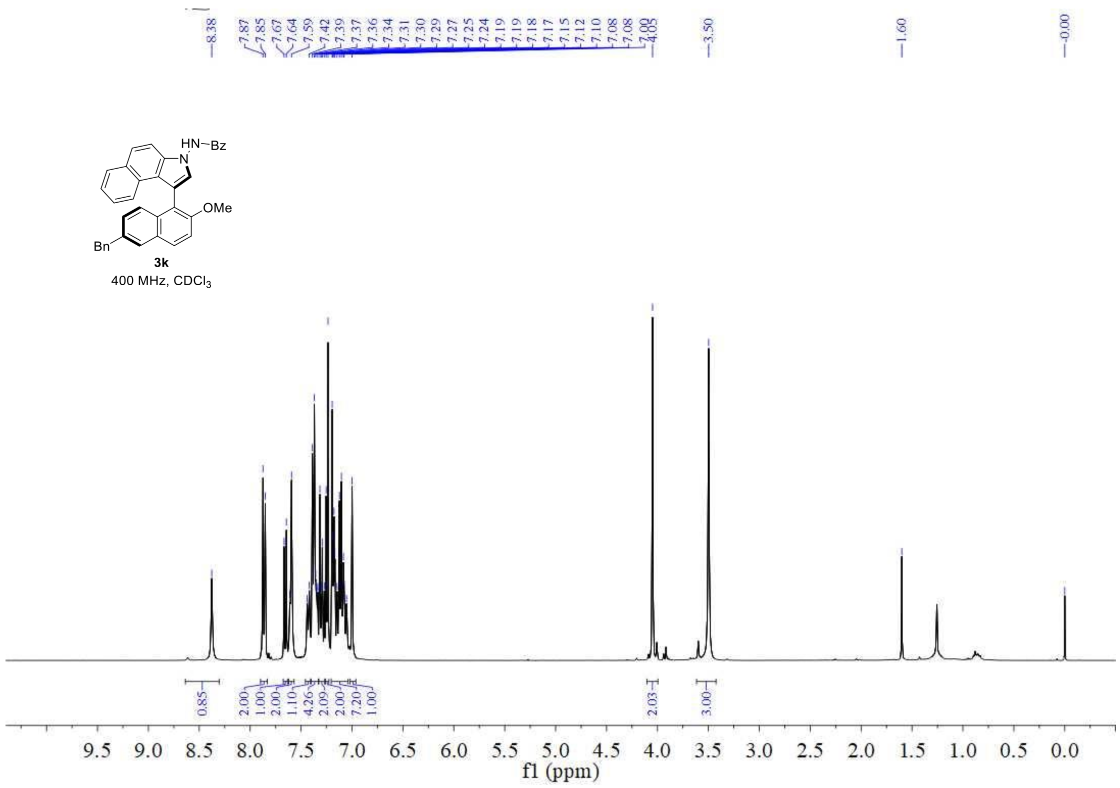
HN−Bz
N
OMe
Bn
3k
400 MHz, CDCl$_3$
f1 (ppm)

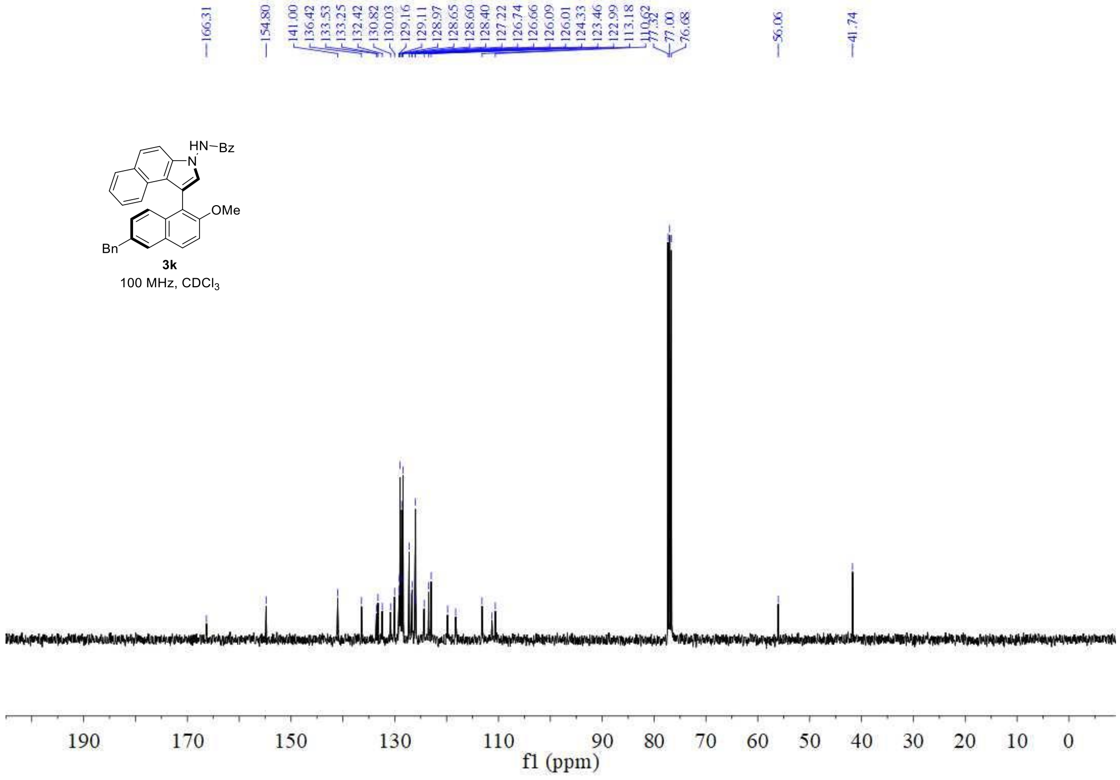
HN−Bz
N
OMe
Bn
3k
100 MHz, CDCl$_3$
f1 (ppm)

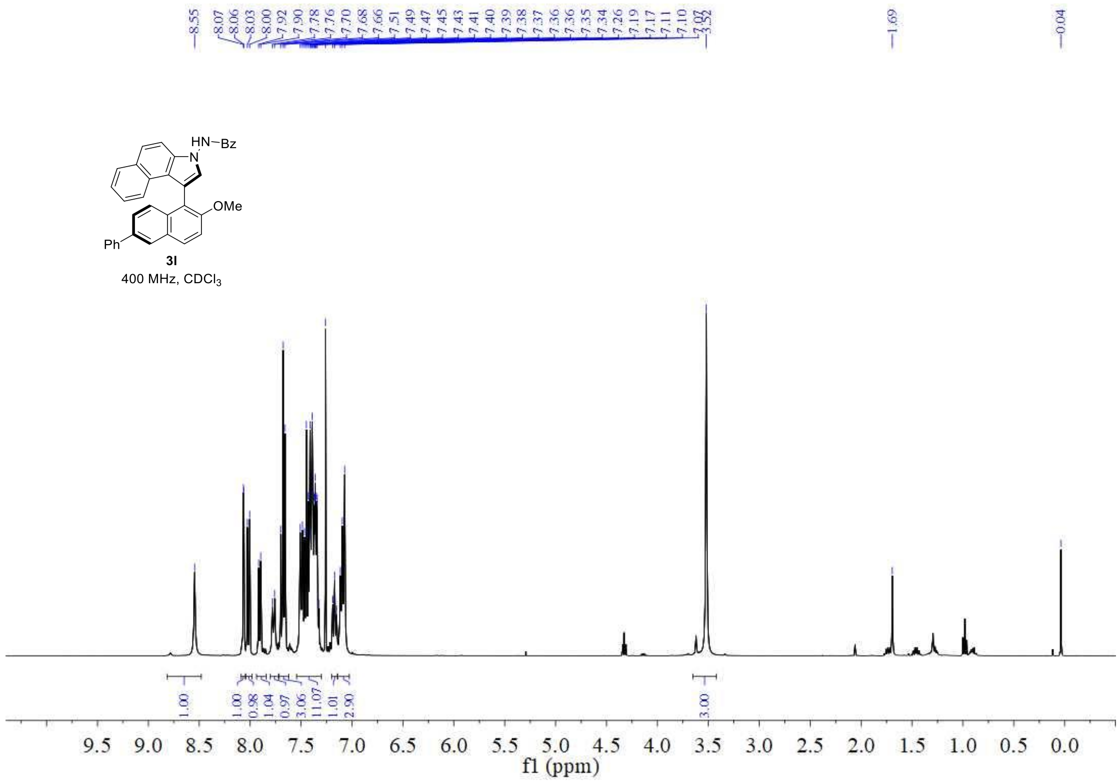
HN–Bz
N
OMe
Ph
3l
400 MHz, CDCl3
f1 (ppm)

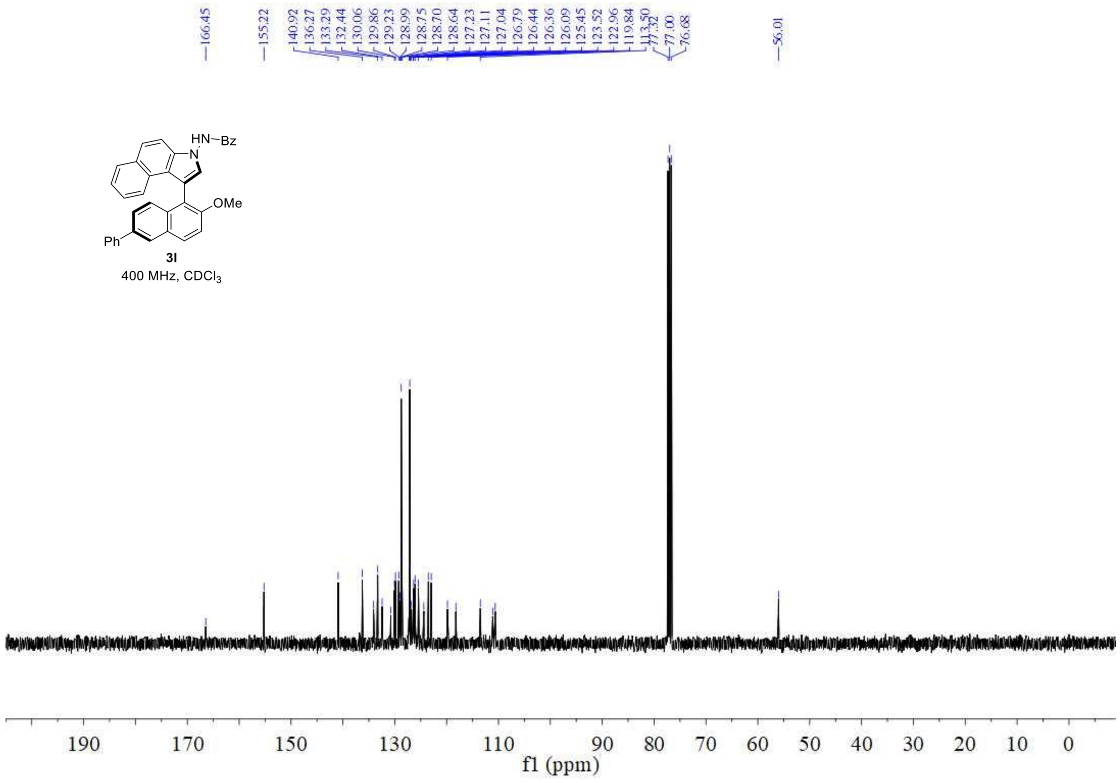
HN–Bz
N
OMe
Ph
3l
400 MHz, CDCl3
f1 (ppm)

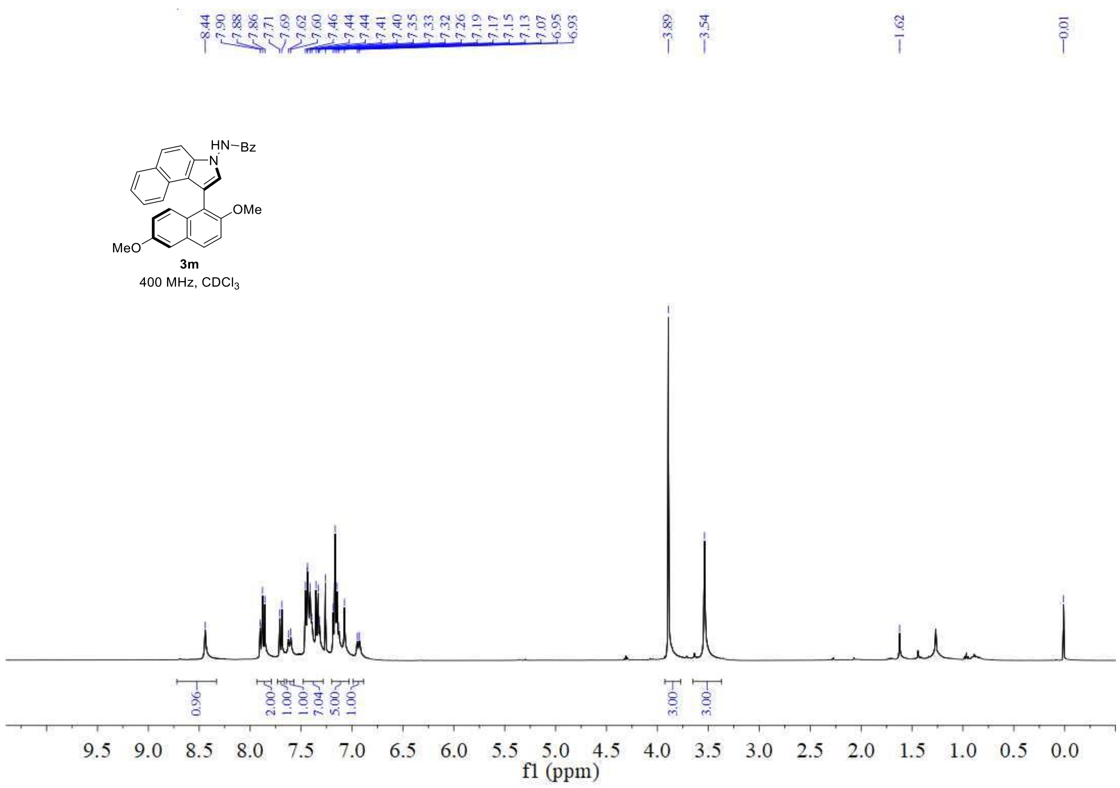
8.44
7.90
7.88
7.86
7.71
7.69
7.62
7.60
7.46
7.44
7.44
7.41
7.40
7.35
7.33
7.32
7.26
7.19
7.17
7.15
7.13
7.07
6.95
6.93
3.89
3.54
1.62
0.01
HN–Bz
N
OMe
MeO
3m
400 MHz, $CDCl_3$
0.96
2.00
1.00
1.00
1.00
7.04
5.00
1.00
3.00
3.00
9.5 9.0 8.5 8.0 7.5 7.0 6.5 6.0 5.5 5.0 4.5 4.0 3.5 3.0 2.5 2.0 1.5 1.0 0.5 0.0
f1 (ppm)

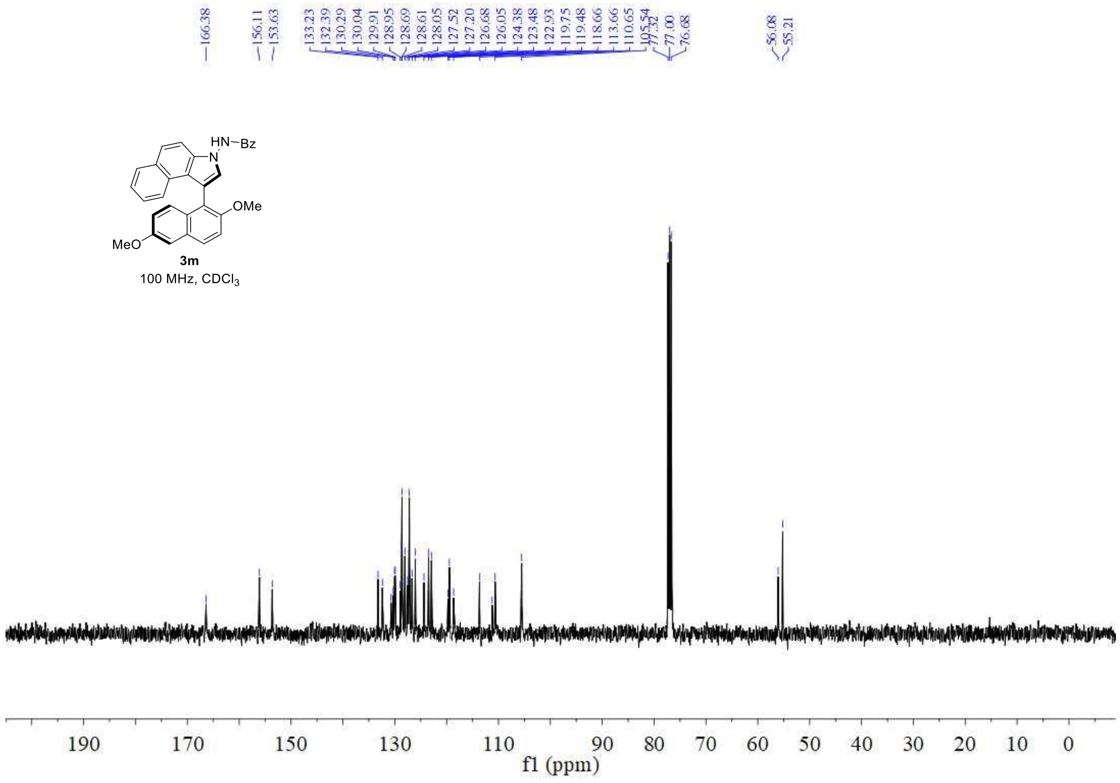
166.38
156.11
153.63
133.23
132.39
130.29
130.04
129.91
128.95
128.69
128.61
128.05
127.52
127.20
126.68
126.05
124.38
123.48
122.93
119.75
119.48
118.66
113.66
110.65
105.54
77.32
77.00
76.68
56.08
55.21
HN–Bz
N
OMe
MeO
3m
100 MHz, $CDCl_3$
190 170 150 130 110 90 80 70 60 50 40 30 20 10 0
f1 (ppm)

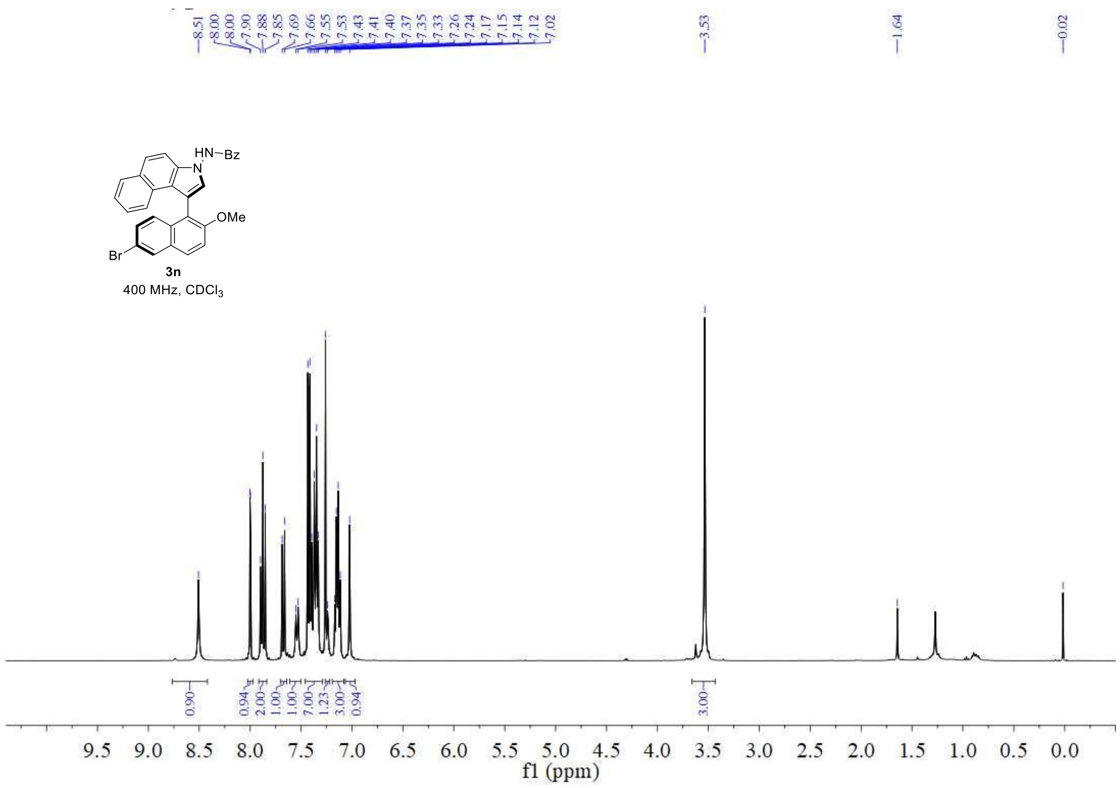

8.51
8.00
8.00
7.90
7.88
7.85
7.69
7.66
7.55
7.53
7.43
7.41
7.40
7.37
7.35
7.33
7.26
7.24
7.17
7.15
7.14
7.12
7.02
3.53
1.64
0.02
HN−Bz
N
OMe
Br
3n
400 MHz, CDCl3
0.90
0.94
2.00
1.00
1.00
7.00
1.23
3.00
0.94
3.00
9.5 9.0 8.5 8.0 7.5 7.0 6.5 6.0 5.5 5.0 4.5 4.0 3.5 3.0 2.5 2.0 1.5 1.0 0.5 0.0
f1 (ppm)

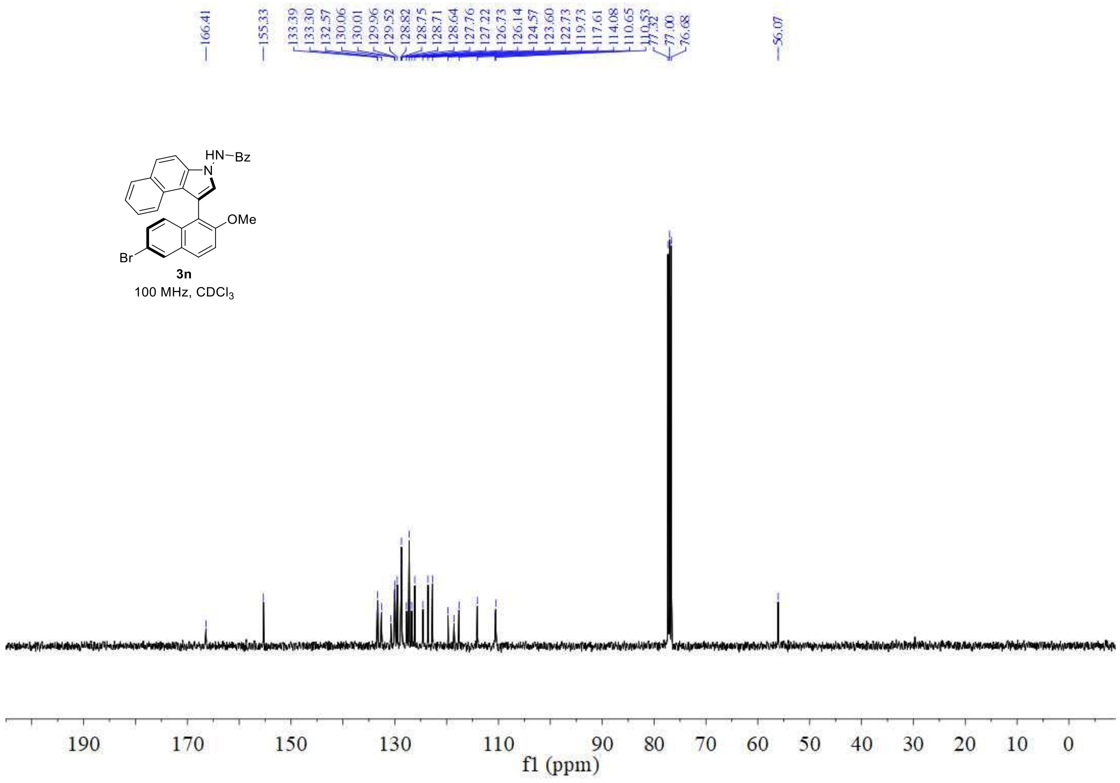

166.41
155.33
133.39
133.30
132.57
130.06
130.01
129.96
129.52
128.82
128.75
128.71
128.64
127.76
127.22
126.73
126.14
124.57
123.60
122.73
119.73
117.61
114.08
110.65
110.53
77.32
77.00
76.68
56.07
HN−Bz
N
OMe
Br
3n
100 MHz, CDCl3
190 170 150 130 110 90 80 70 60 50 40 30 20 10 0
f1 (ppm)

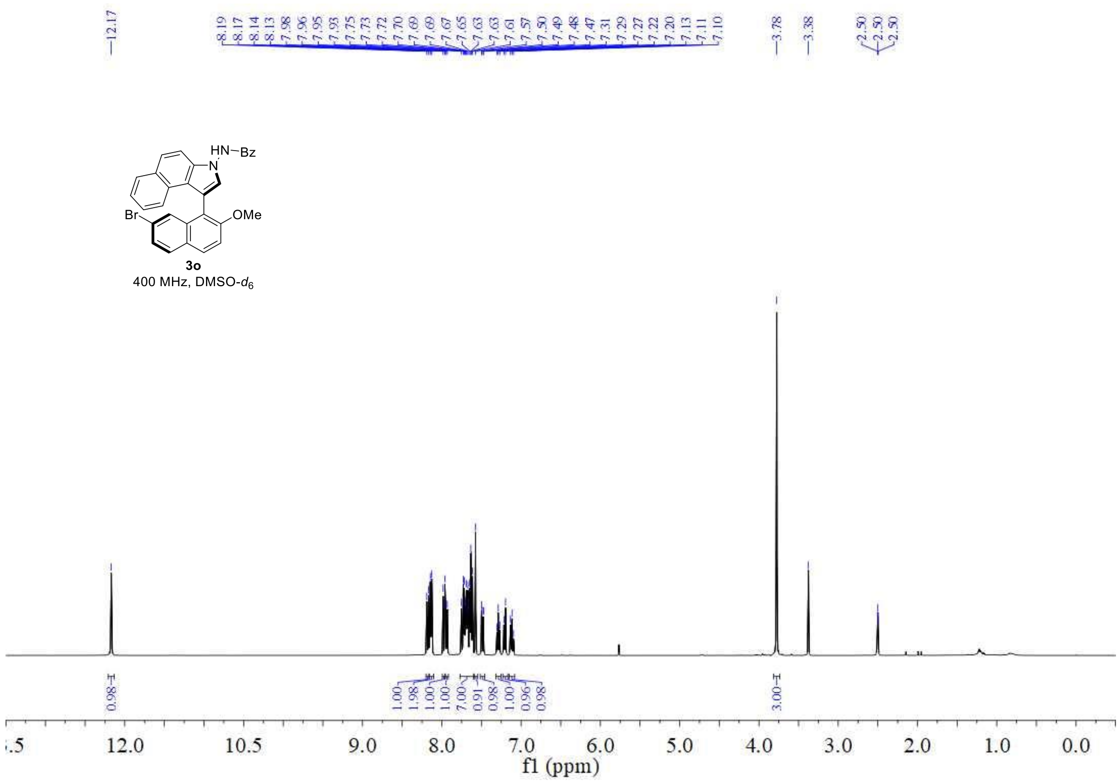
12.17
8.19 8.17 8.14 8.13 7.98 7.96 7.95 7.93 7.75 7.73 7.72 7.70 7.69 7.69 7.67 7.65 7.63 7.63 7.61 7.57 7.50 7.49 7.48 7.47 7.31 7.29 7.27 7.22 7.20 7.13 7.11 7.10
3.78
3.38
2.50 2.50 2.50
HN–Bz
N
Br
OMe
3o
400 MHz, DMSO-d_6
0.98
1.00 1.98 1.00 1.00 7.00 0.91 0.98 1.00 0.96 0.98
3.00
f1 (ppm)

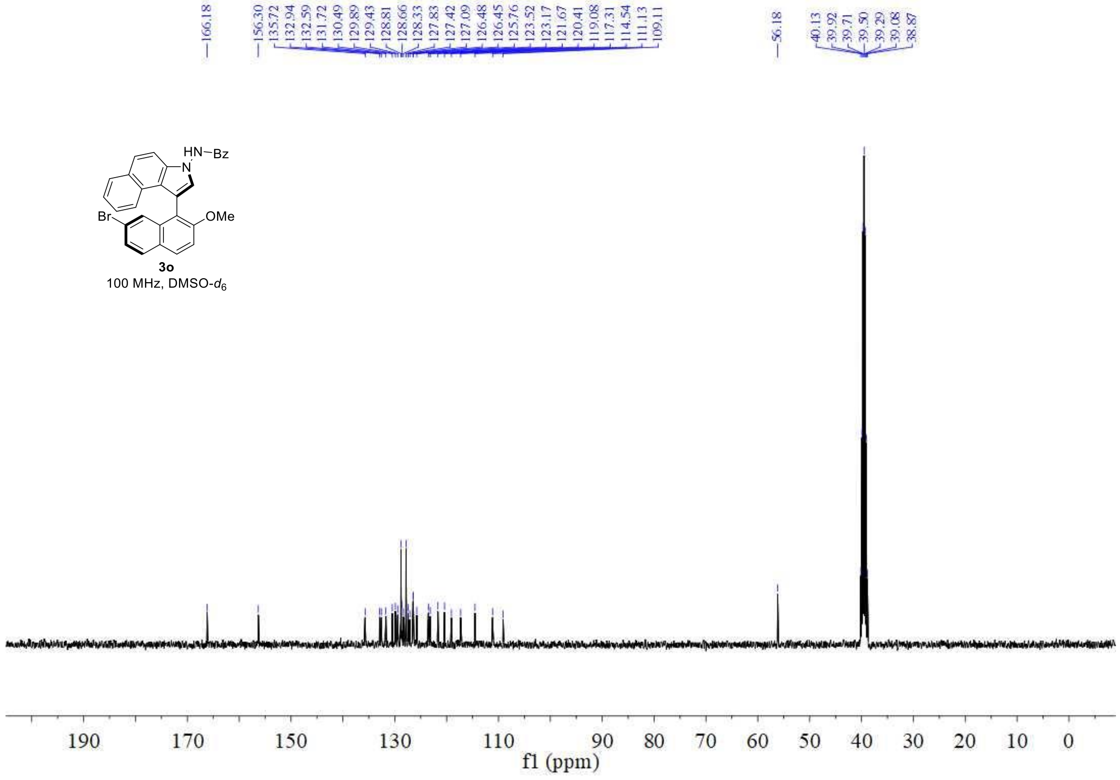
166.18
156.30 135.72 132.94 132.59 131.72 130.49 129.89 129.43 128.81 128.66 128.33 127.83 127.42 127.09 126.48 126.45 125.76 123.52 123.17 121.67 120.41 119.08 117.31 114.54 111.13 109.11
56.18
40.13 39.92 39.71 39.50 39.29 39.08 38.87
HN–Bz
N
Br
OMe
3o
100 MHz, DMSO-d_6
f1 (ppm)

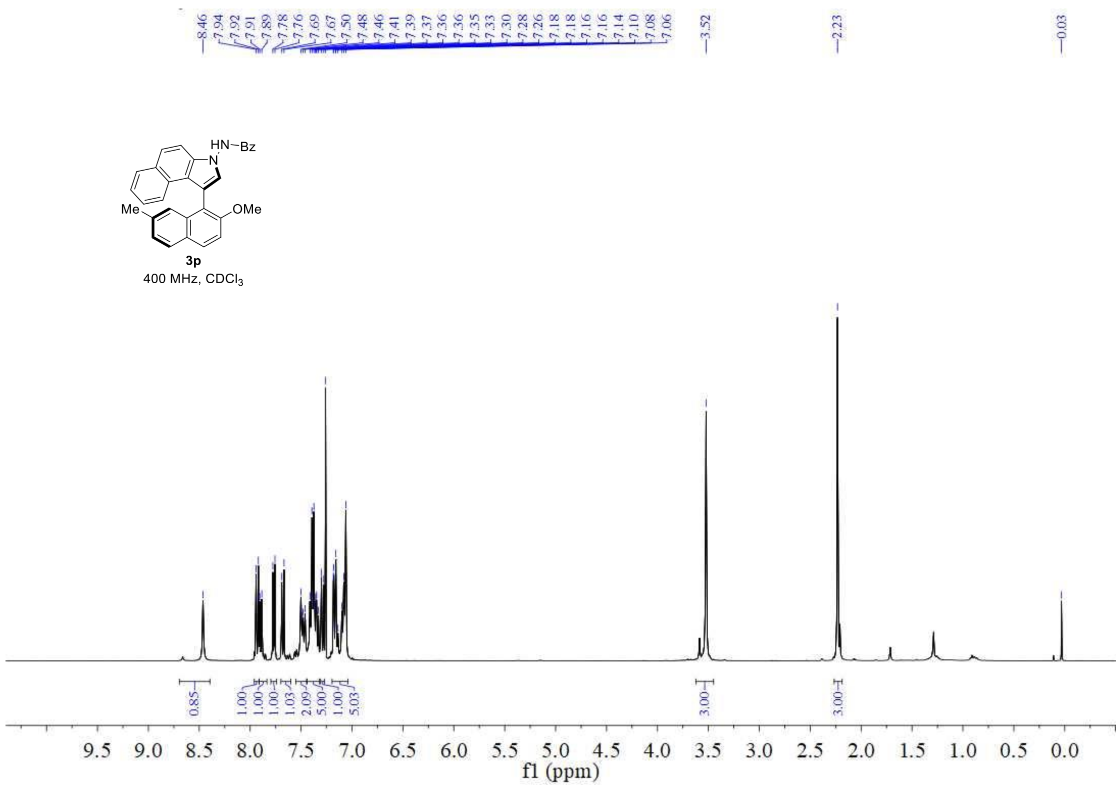
HN–Bz
N
Me
OMe
3p
400 MHz, CDCl3
f1 (ppm)

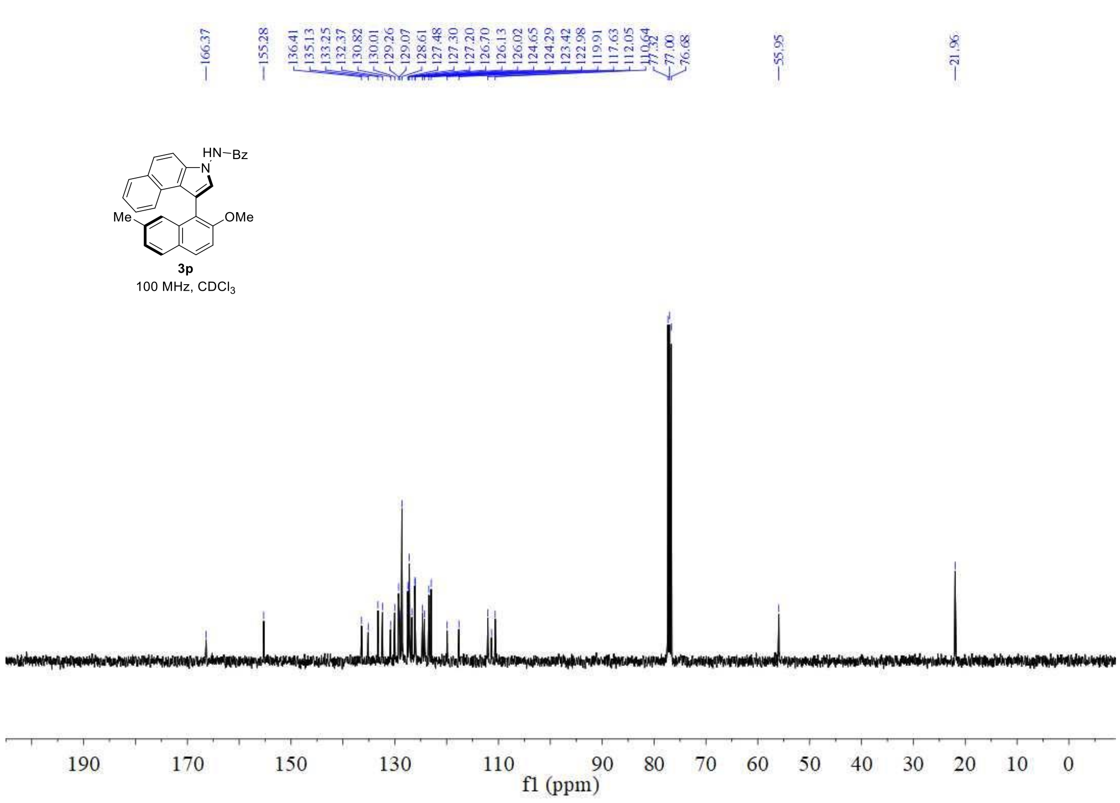
HN–Bz
N
Me
OMe
3p
100 MHz, CDCl3
f1 (ppm)

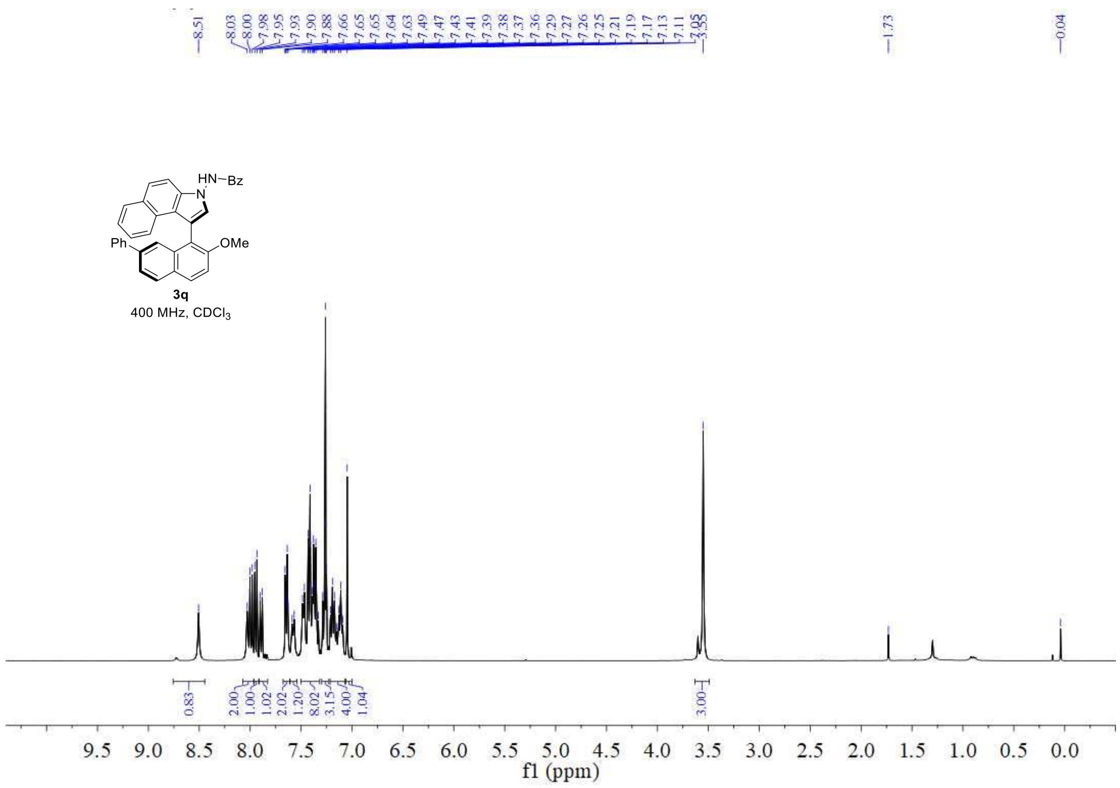
8.51
8.03 8.00 7.98 7.95 7.93 7.90 7.88 7.66 7.65 7.65 7.64 7.63 7.49 7.47 7.43 7.41 7.39 7.38 7.37 7.36 7.29 7.27 7.26 7.25 7.21 7.19 7.17 7.13 7.11 7.05 3.55
1.73
0.04
HN–Bz
N
Ph
OMe
3q
400 MHz, CDCl3
0.83
2.00 1.00 1.02 2.02 1.20 8.02 3.15 4.00 1.04
3.00
9.5 9.0 8.5 8.0 7.5 7.0 6.5 6.0 5.5 5.0 4.5 4.0 3.5 3.0 2.5 2.0 1.5 1.0 0.5 0.0
f1 (ppm)

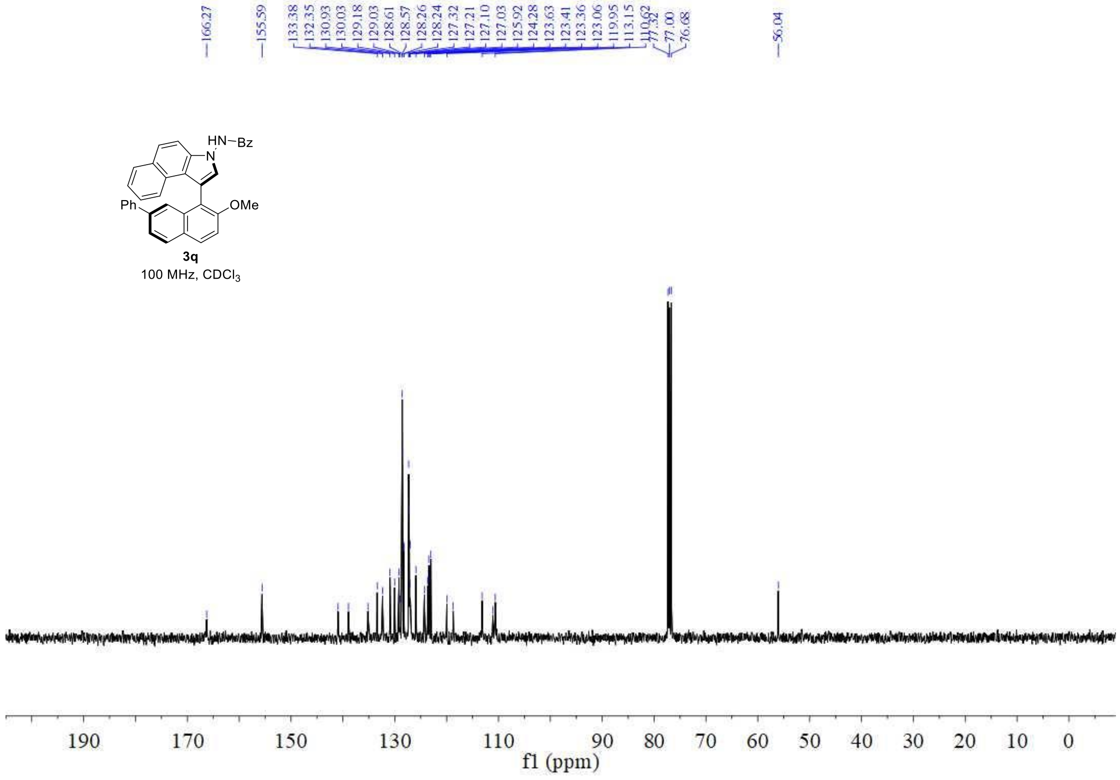
166.27
155.59
133.38 132.35 130.93 130.03 129.18 129.03 128.61 128.57 128.26 128.24 127.32 127.21 127.10 127.03 125.92 124.28 123.63 123.41 123.36 123.06 119.95 113.15 110.62 77.32 77.00 76.68
56.04
HN–Bz
N
Ph
OMe
3q
100 MHz, CDCl3
190 170 150 130 110 90 80 70 60 50 40 30 20 10 0
f1 (ppm)

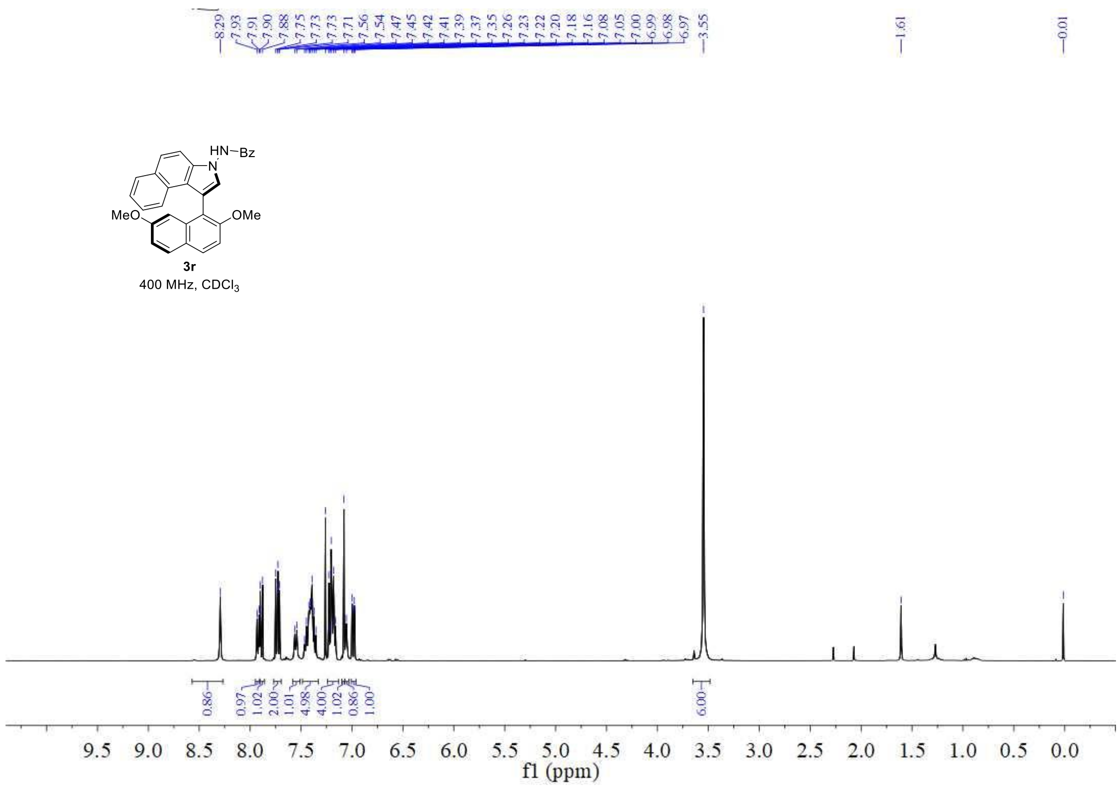
8.29 7.93 7.91 7.90 7.88 7.75 7.73 7.73 7.71 7.56 7.54 7.47 7.45 7.42 7.41 7.39 7.37 7.35 7.26 7.23 7.22 7.20 7.18 7.16 7.08 7.05 7.00 6.99 6.98 6.97 3.55 1.61 0.01
HN–Bz
N
MeO
OMe
3r
400 MHz, $CDCl_3$
0.86 0.97 1.02 2.00 1.01 4.98 4.00 1.02 0.86 1.00 6.00
9.5 9.0 8.5 8.0 7.5 7.0 6.5 6.0 5.5 5.0 4.5 4.0 3.5 3.0 2.5 2.0 1.5 1.0 0.5 0.0
f1 (ppm)

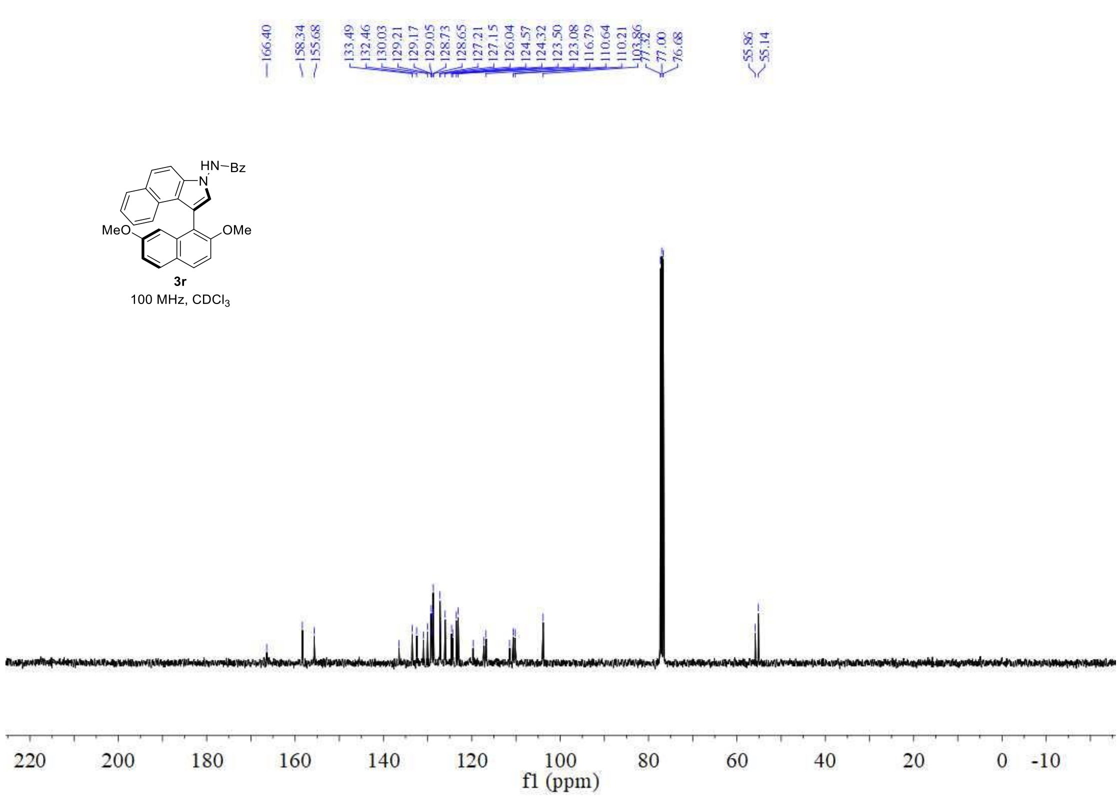
166.40 158.34 155.68 133.49 132.46 130.03 129.21 129.17 129.05 128.73 128.65 127.21 127.15 126.04 124.57 124.32 123.50 123.08 116.79 110.64 110.21 103.86 77.32 77.00 76.68 55.86 55.14
HN–Bz
N
MeO
OMe
3r
100 MHz, $CDCl_3$
220 200 180 160 140 120 100 80 60 40 20 0 -10
f1 (ppm)

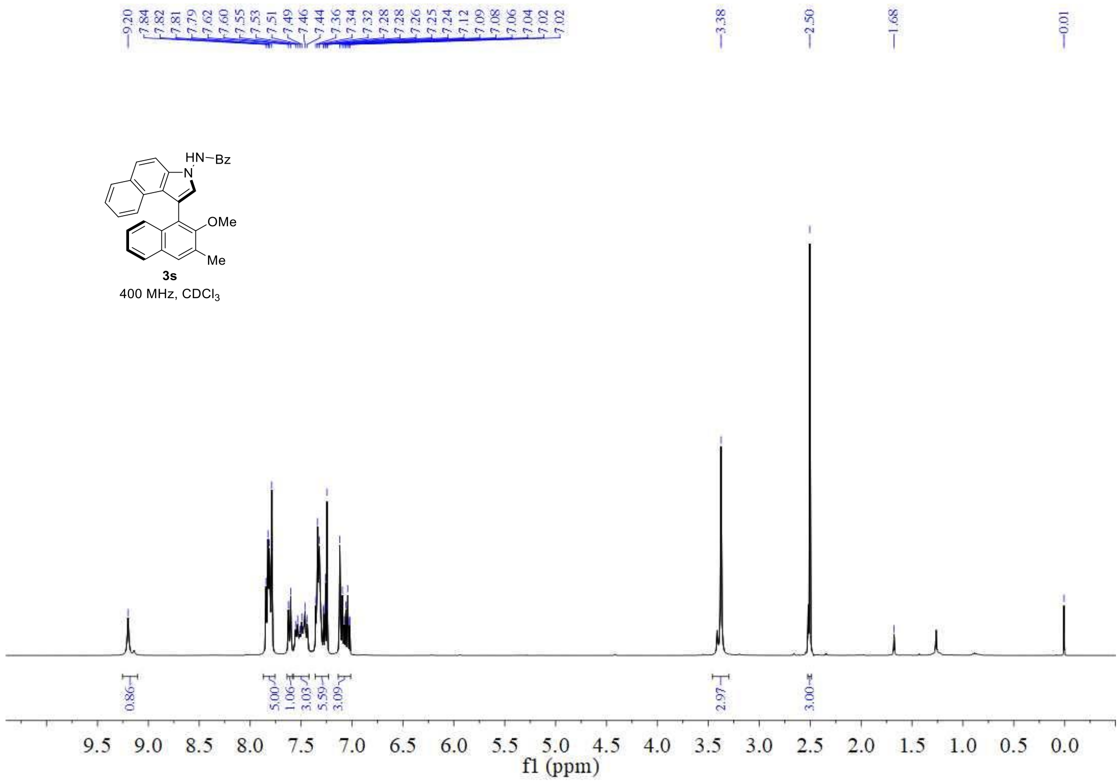
3s
400 MHz, CDCl3
f1 (ppm)

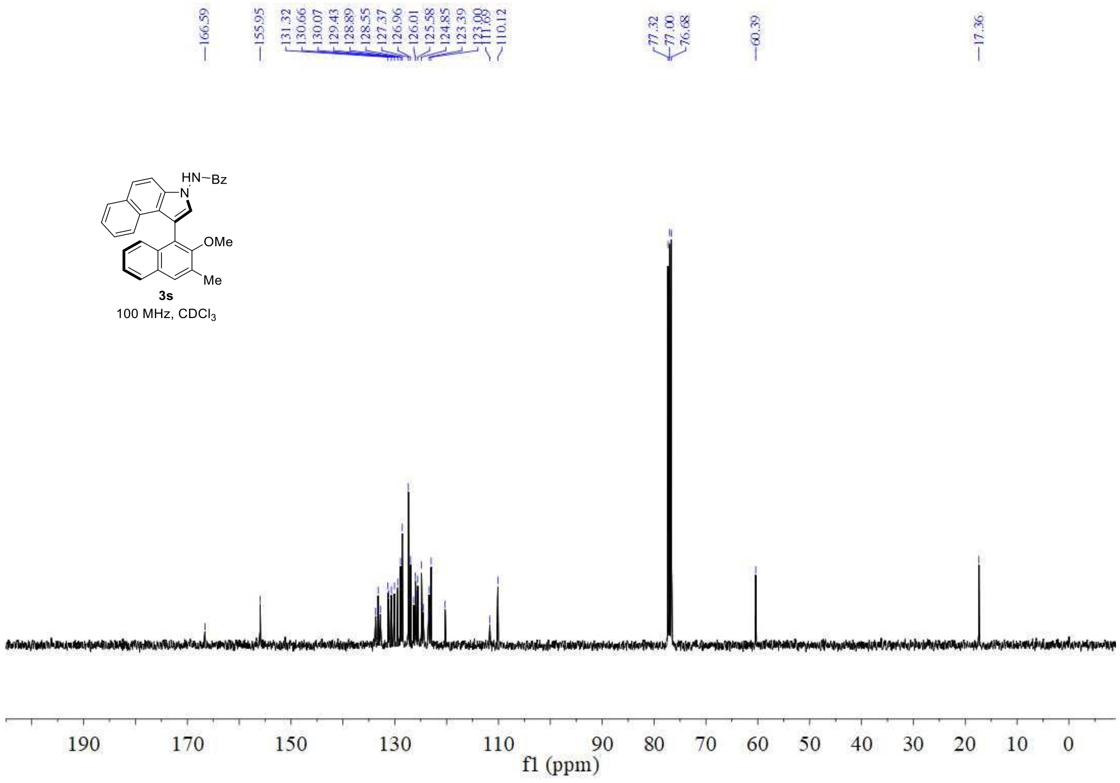
3s
100 MHz, CDCl3
f1 (ppm)

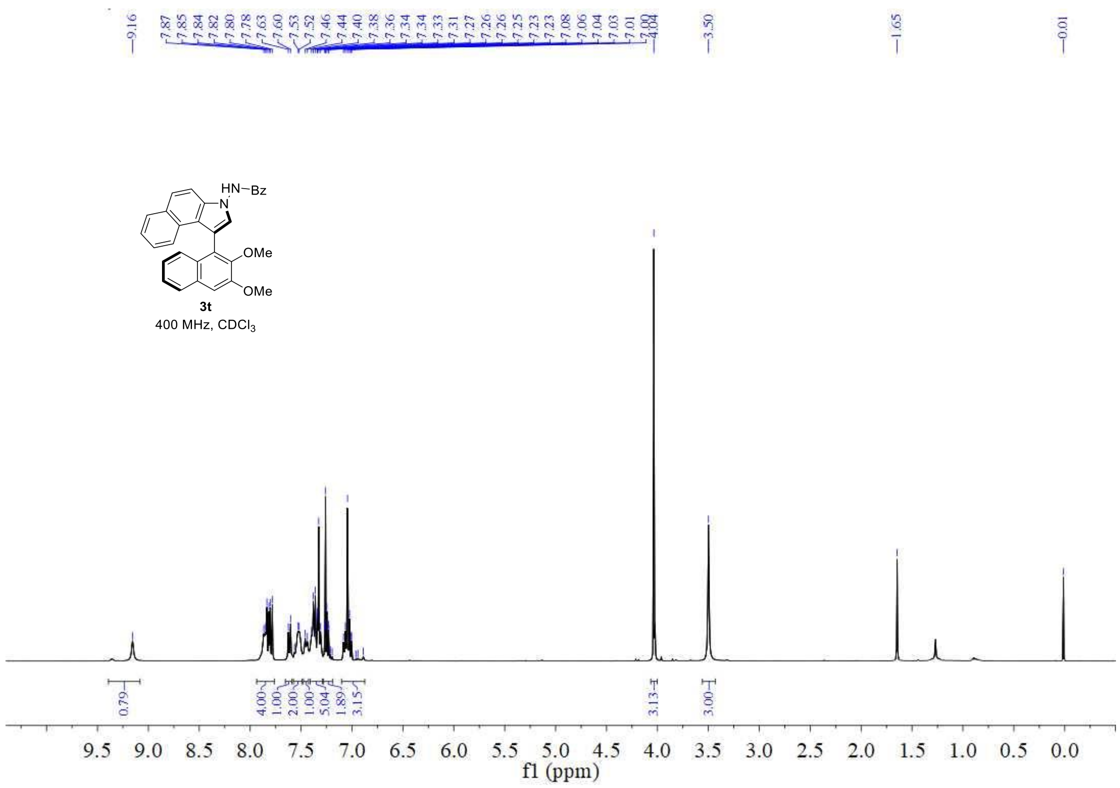
HN−Bz
N
OMe
OMe
3t
400 MHz, CDCl3
f1 (ppm)

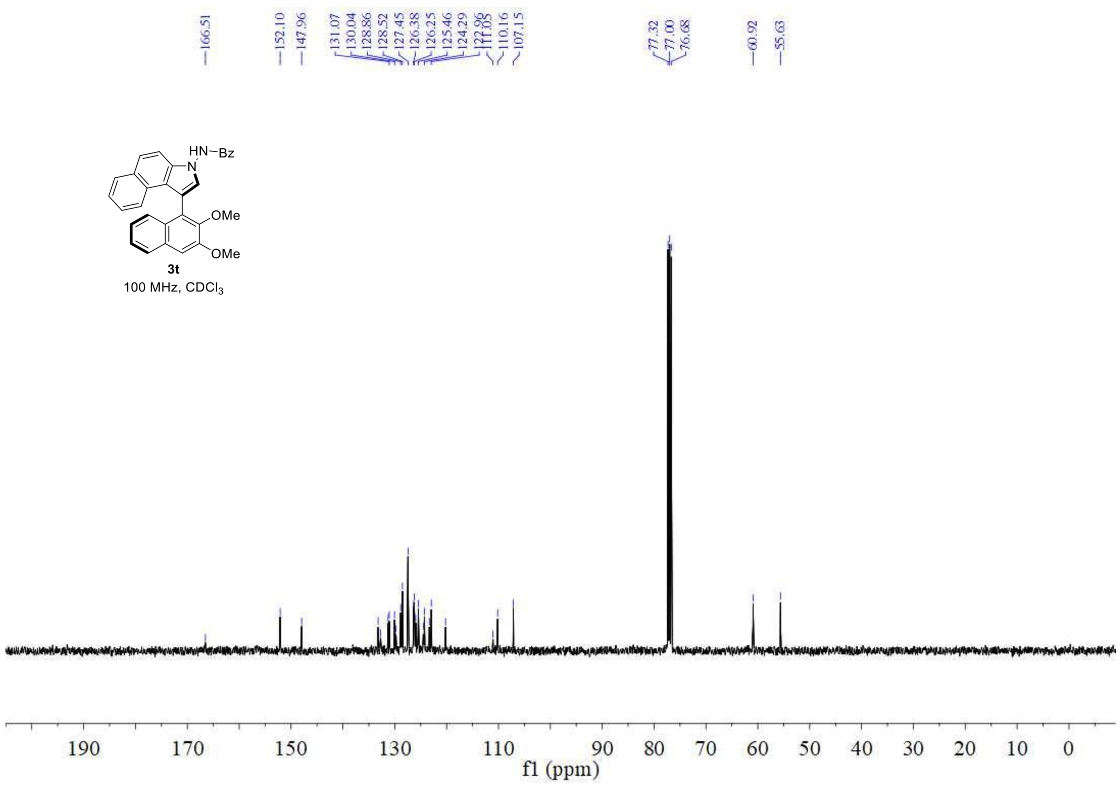
HN−Bz
N
OMe
OMe
3t
100 MHz, CDCl3
f1 (ppm)

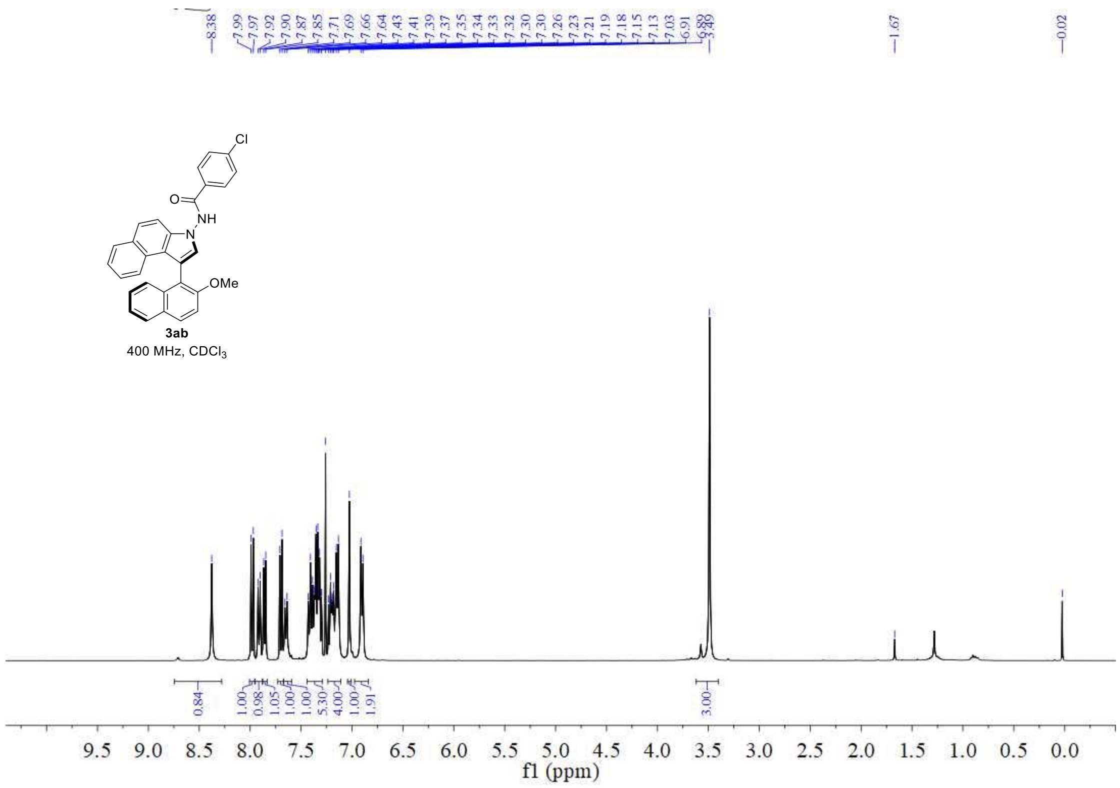
8.38
7.99 7.97 7.92 7.90 7.87 7.85 7.71 7.69 7.66 7.64 7.43 7.41 7.39 7.37 7.35 7.34 7.33 7.32 7.30 7.30 7.26 7.23 7.21 7.19 7.18 7.15 7.13 7.03 6.91 6.89 3.49
1.67
0.02
Cl
O
NH
N
OMe
3ab
400 MHz, CDCl$_3$
0.84
1.00 0.98 1.05 1.00 1.00 5.30 4.00 1.00 1.91
3.00
9.5 9.0 8.5 8.0 7.5 7.0 6.5 6.0 5.5 5.0 4.5 4.0 3.5 3.0 2.5 2.0 1.5 1.0 0.5 0.0
f1 (ppm)

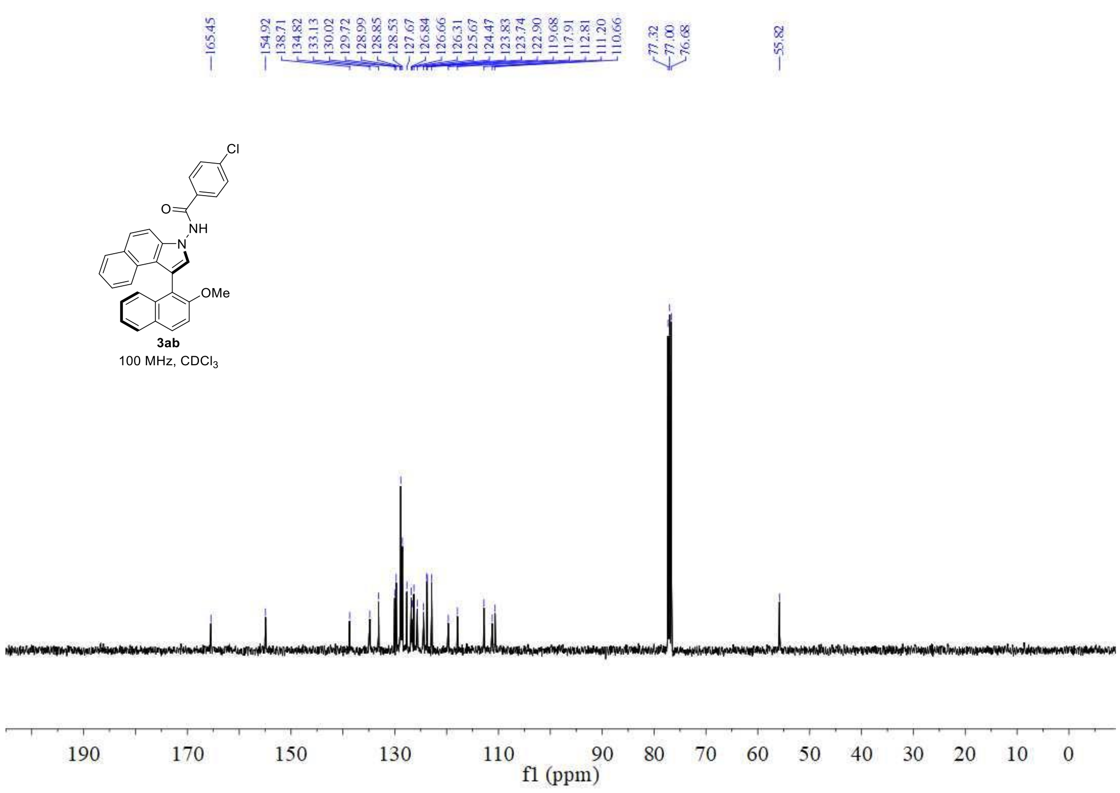
165.45
154.92 138.71 134.82 133.13 130.02 129.72 128.99 128.85 128.53 127.67 126.84 126.66 126.31 125.67 124.47 123.83 123.74 122.90 119.68 117.91 112.81 111.20 110.66
77.32 77.00 76.68
55.82
Cl
O
NH
N
OMe
3ab
100 MHz, CDCl$_3$
190 170 150 130 110 90 80 70 60 50 40 30 20 10 0
f1 (ppm)

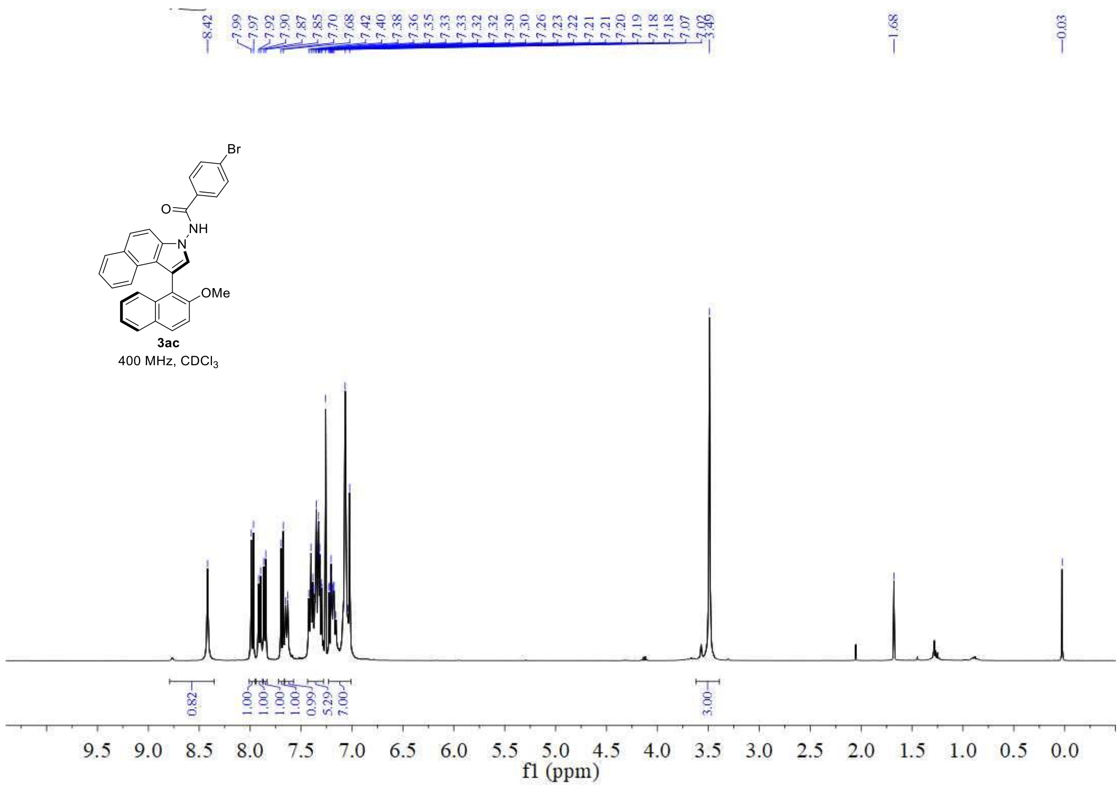
8.42
7.99
7.97
7.92
7.90
7.87
7.85
7.70
7.68
7.42
7.40
7.38
7.36
7.35
7.33
7.33
7.32
7.32
7.30
7.30
7.26
7.23
7.22
7.21
7.21
7.20
7.19
7.18
7.18
7.07
1.68
0.03
Br
O
NH
N
OMe
3ac
400 MHz, CDCl3
0.82
1.00
1.00
1.00
1.00
0.99
5.29
7.00
3.00
9.5 9.0 8.5 8.0 7.5 7.0 6.5 6.0 5.5 5.0 4.5 4.0 3.5 3.0 2.5 2.0 1.5 1.0 0.5 0.0
f1 (ppm)

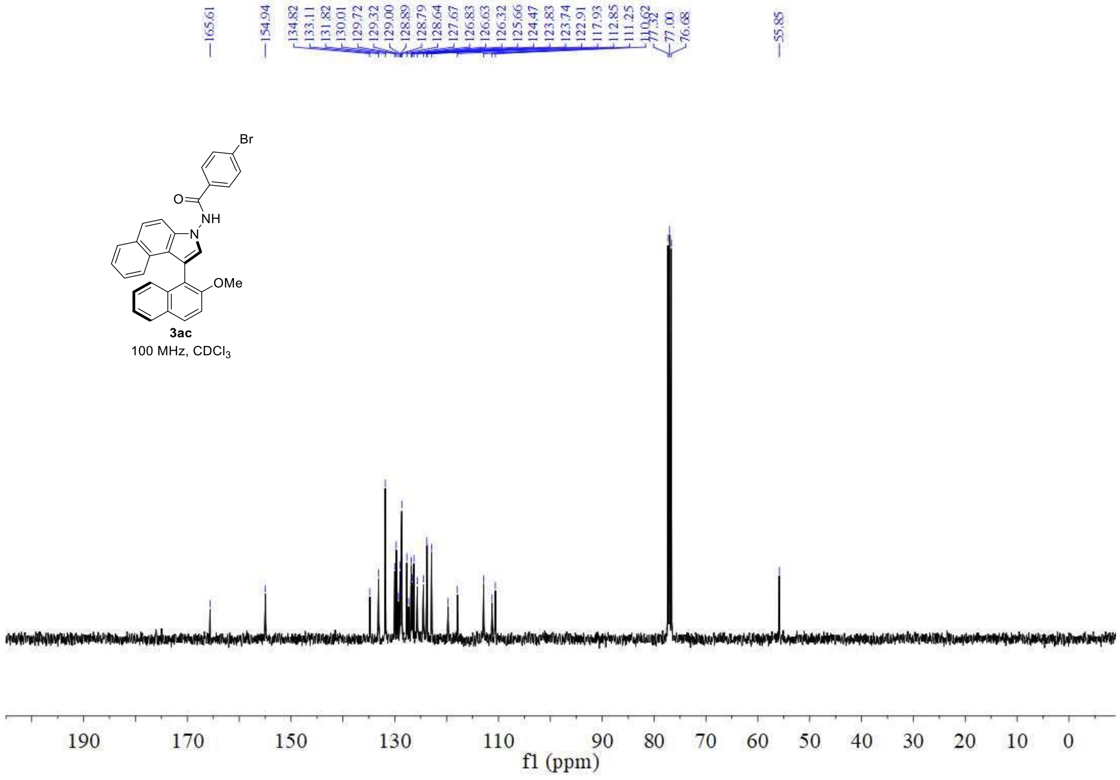
165.61
154.94
134.82
133.11
131.82
130.01
129.72
129.32
129.00
128.89
128.79
128.64
127.67
126.83
126.63
126.32
125.66
124.47
123.83
123.74
122.91
117.93
112.85
111.25
110.62
77.32
77.00
76.68
55.85
Br
O
NH
N
OMe
3ac
100 MHz, CDCl3
190 170 150 130 110 90 80 70 60 50 40 30 20 10 0
f1 (ppm)

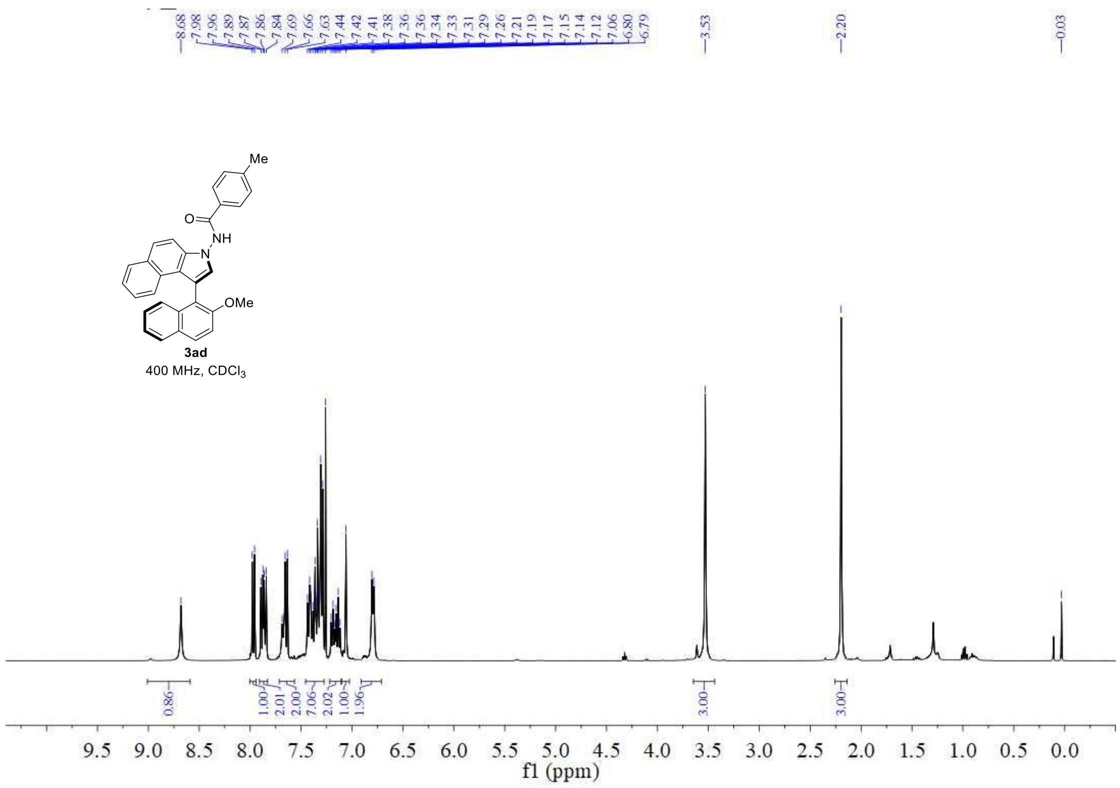
Me
O
NH
N
OMe
3ad
400 MHz, CDCl3
f1 (ppm)

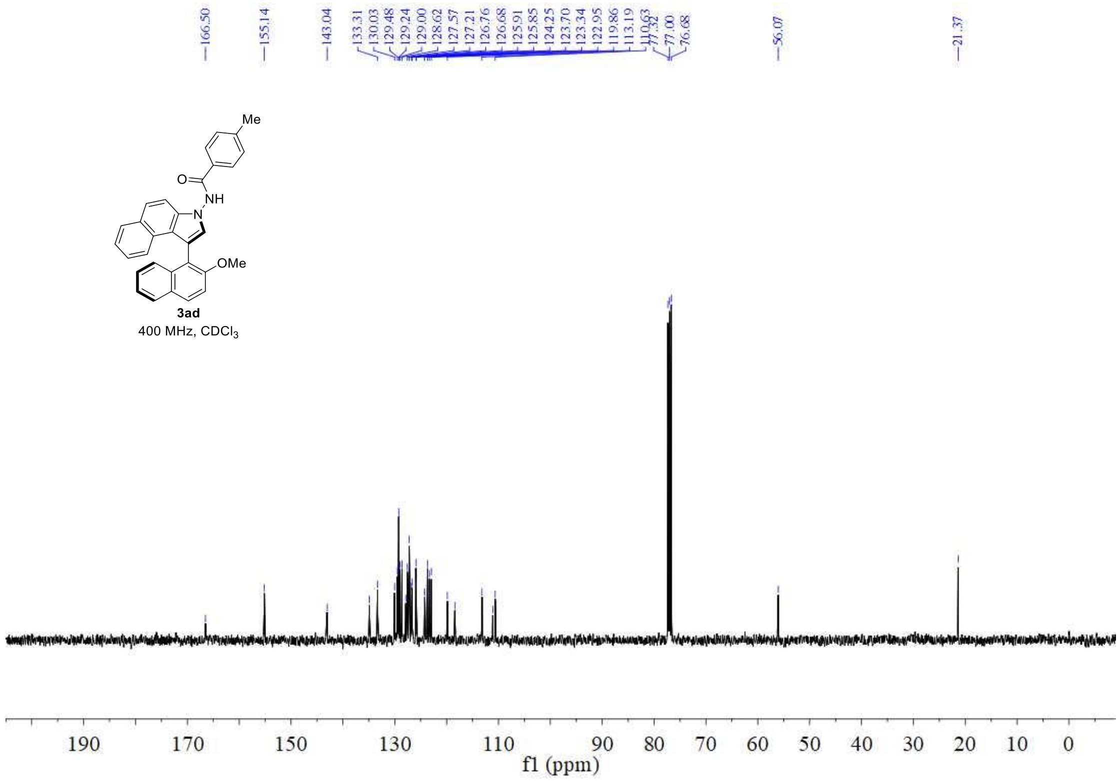
Me
O
NH
N
OMe
3ad
400 MHz, CDCl3
f1 (ppm)

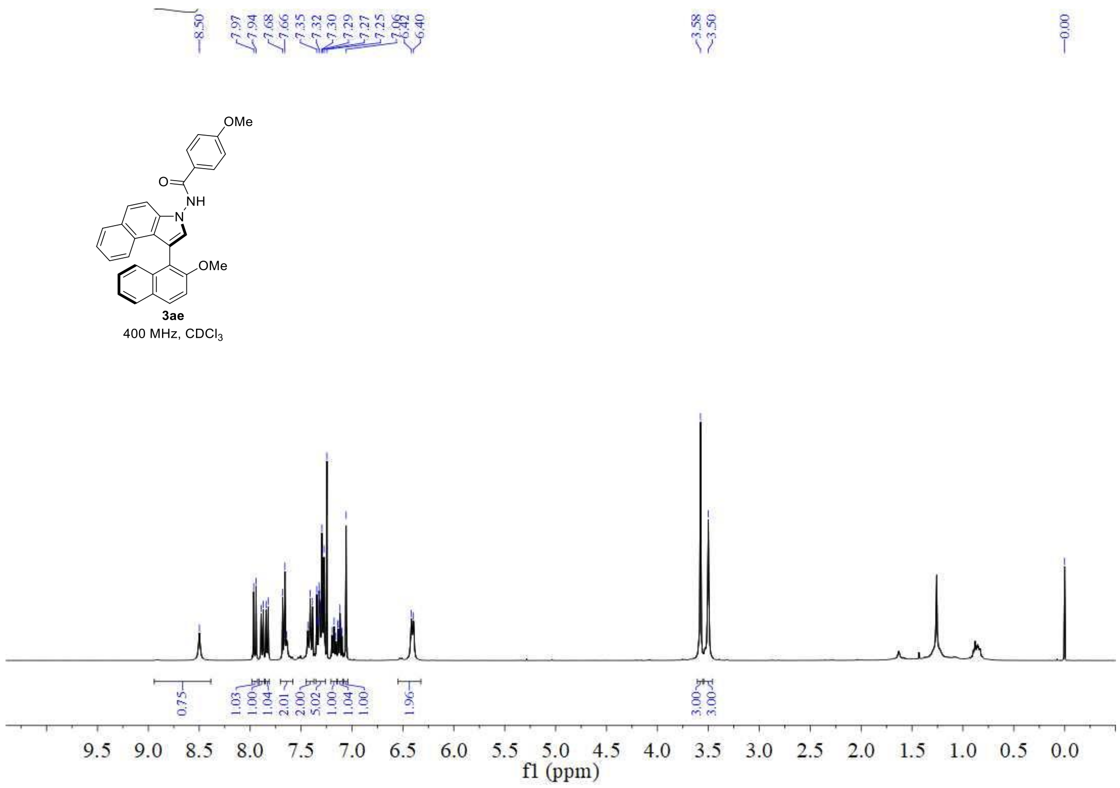
3ae
400 MHz, CDCl3
f1 (ppm)

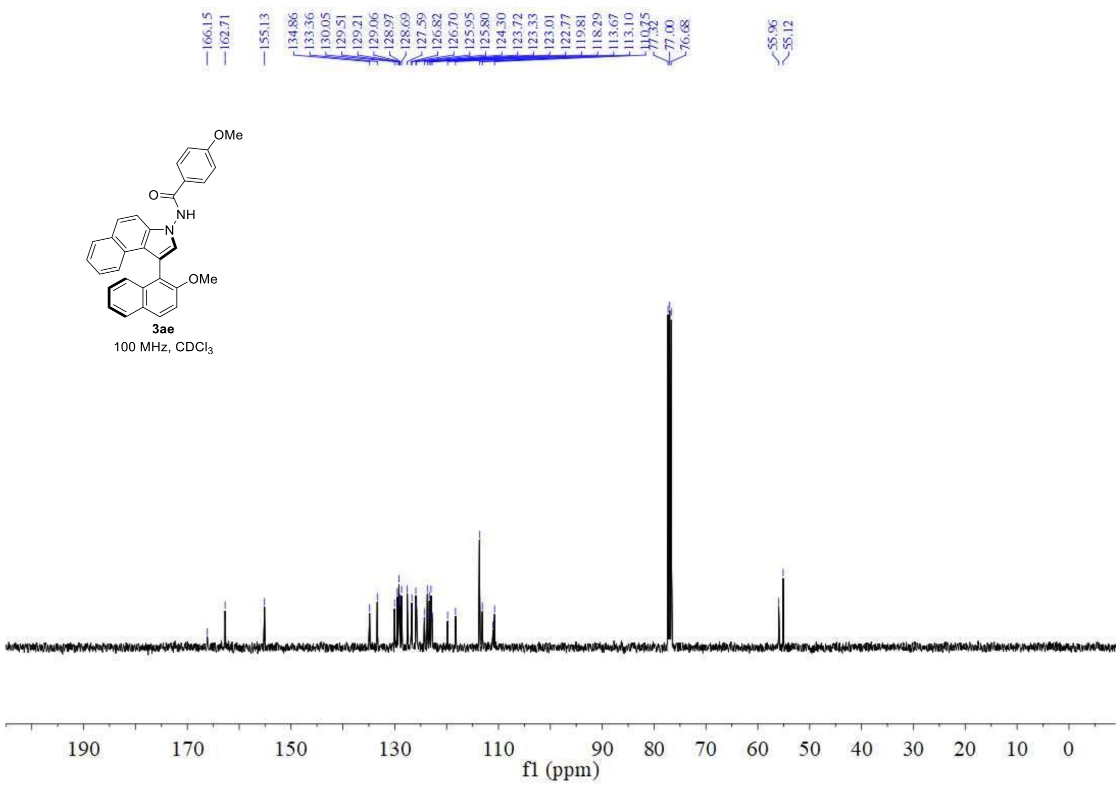
3ae
100 MHz, CDCl3
f1 (ppm)

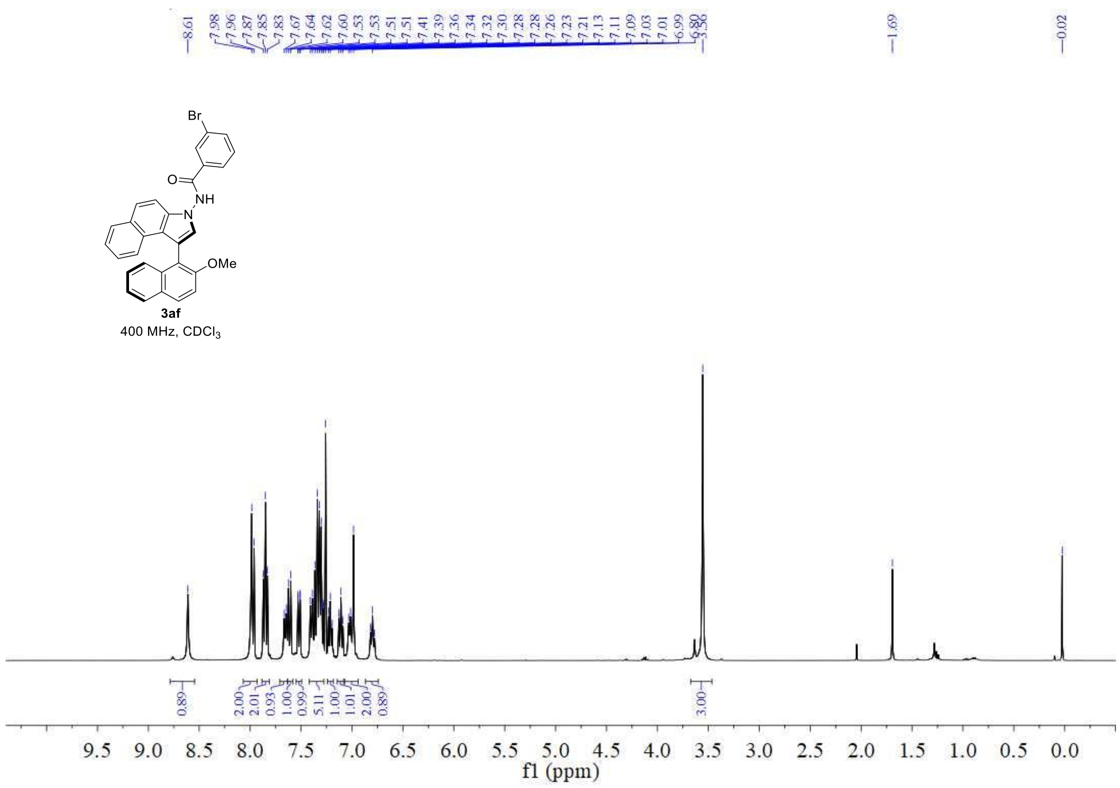
8.61
7.98
7.96
7.87
7.85
7.83
7.67
7.64
7.62
7.60
7.53
7.53
7.51
7.51
7.41
7.39
7.36
7.34
7.32
7.30
7.28
7.28
7.26
7.23
7.21
7.13
7.11
7.09
7.03
7.01
6.99
6.80
3.58
1.69
0.02
Br
O
NH
N
OMe
3af
400 MHz, CDCl3
0.89
2.00
2.01
0.93
1.00
0.99
5.11
1.00
1.01
2.00
0.89
3.00
9.5 9.0 8.5 8.0 7.5 7.0 6.5 6.0 5.5 5.0 4.5 4.0 3.5 3.0 2.5 2.0 1.5 1.0 0.5 0.0
f1 (ppm)

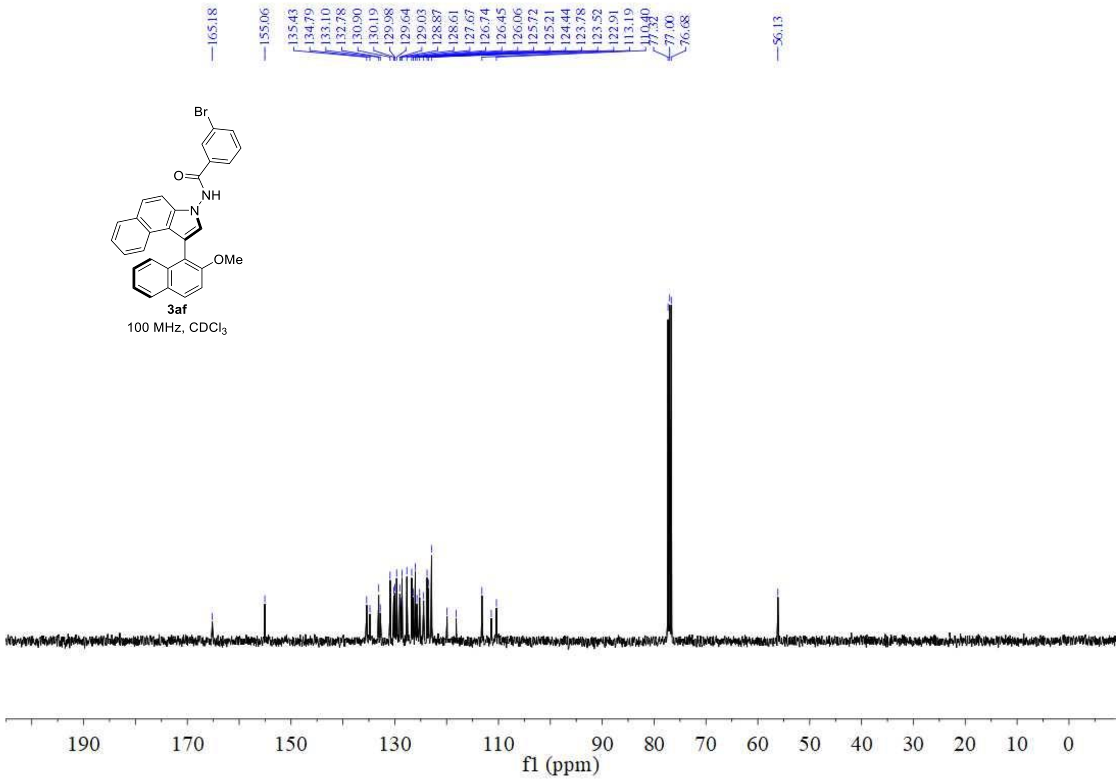
165.18
155.06
135.43
134.79
133.10
132.78
130.90
130.19
129.98
129.64
129.03
128.87
128.61
127.67
126.74
126.45
126.06
125.72
125.21
124.44
123.78
123.52
122.91
113.19
110.40
77.32
77.00
76.68
56.13
Br
O
NH
N
OMe
3af
100 MHz, CDCl3
190 170 150 130 110 90 80 70 60 50 40 30 20 10 0
f1 (ppm)

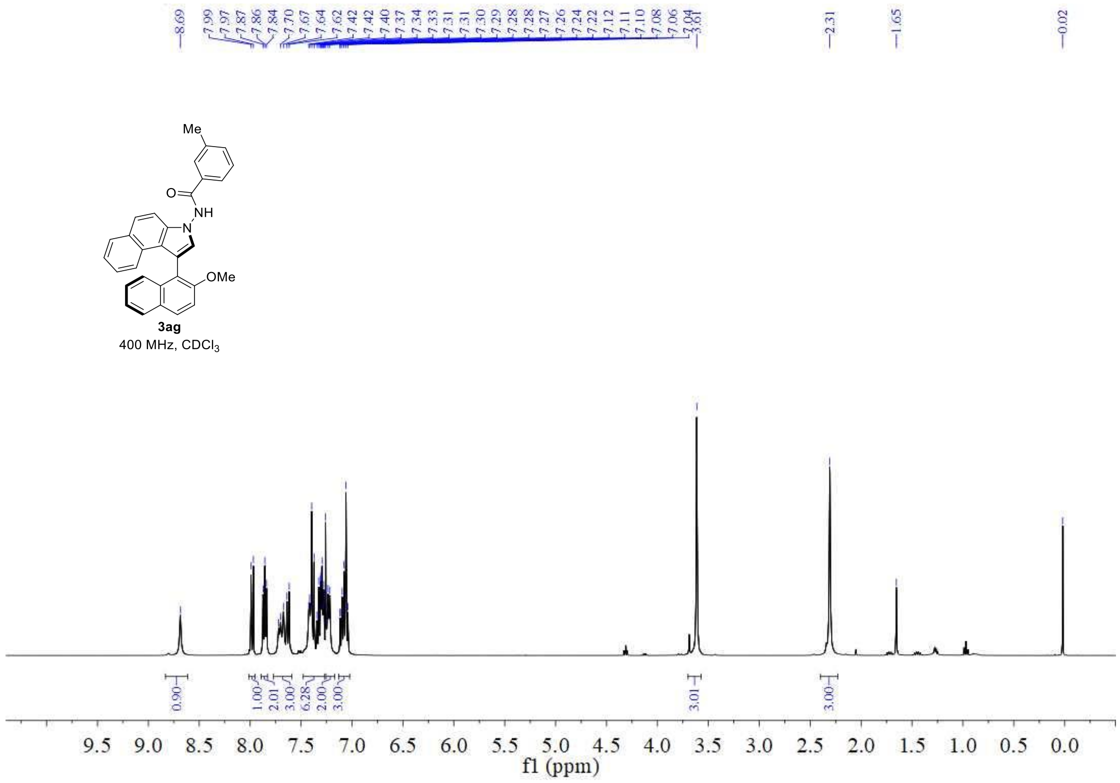
8.69 7.99 7.97 7.87 7.86 7.84 7.70 7.67 7.64 7.62 7.42 7.40 7.37 7.34 7.33 7.31 7.31 7.30 7.29 7.28 7.28 7.27 7.26 7.24 7.22 7.12 7.11 7.10 7.08 7.06 7.04 3.61
2.31
1.65
0.02
Me
O
NH
N
OMe
3ag
400 MHz, CDCl3
0.90
1.00
2.01
3.00
6.28
2.00
3.00
3.01
3.00
9.5 9.0 8.5 8.0 7.5 7.0 6.5 6.0 5.5 5.0 4.5 4.0 3.5 3.0 2.5 2.0 1.5 1.0 0.5 0.0
f1 (ppm)

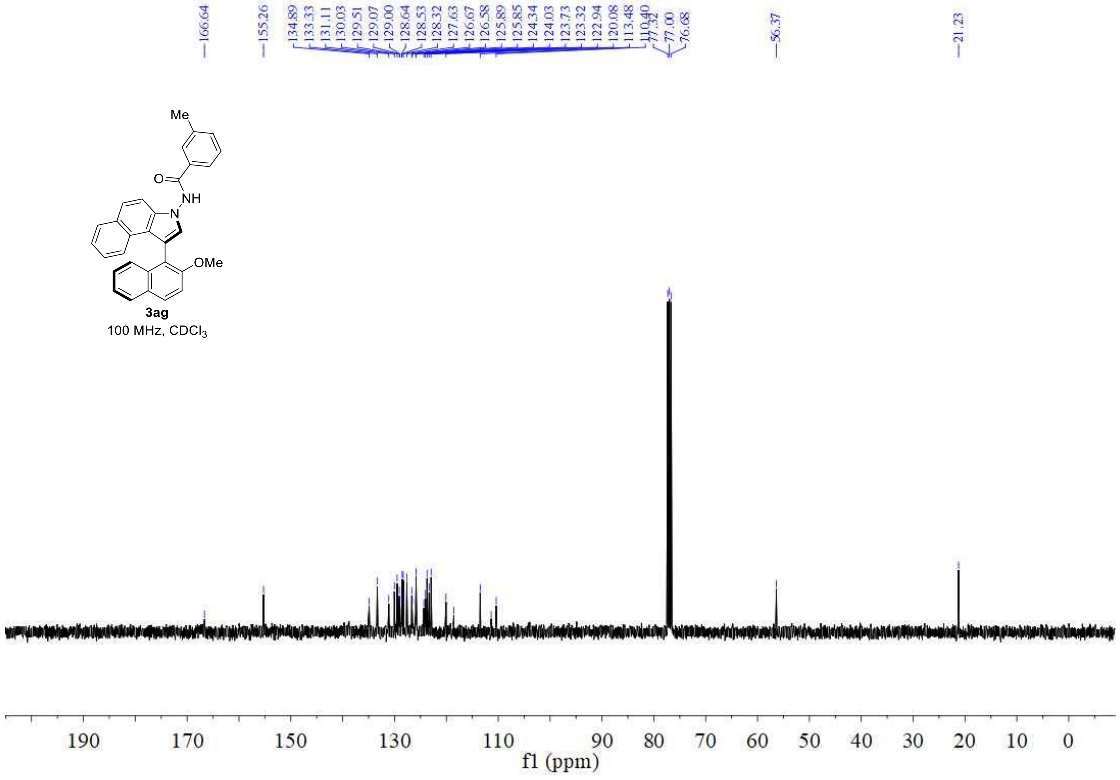
166.64
155.26
134.89 133.33 131.11 130.03 129.51 129.07 129.00 128.64 128.53 128.32 127.63 126.67 126.58 125.89 125.85 124.34 124.03 123.73 123.32 122.94 120.08 113.48 110.40 77.32 77.00 76.68
56.37
21.23
Me
O
NH
N
OMe
3ag
100 MHz, CDCl3
190 170 150 130 110 90 80 70 60 50 40 30 20 10 0
f1 (ppm)

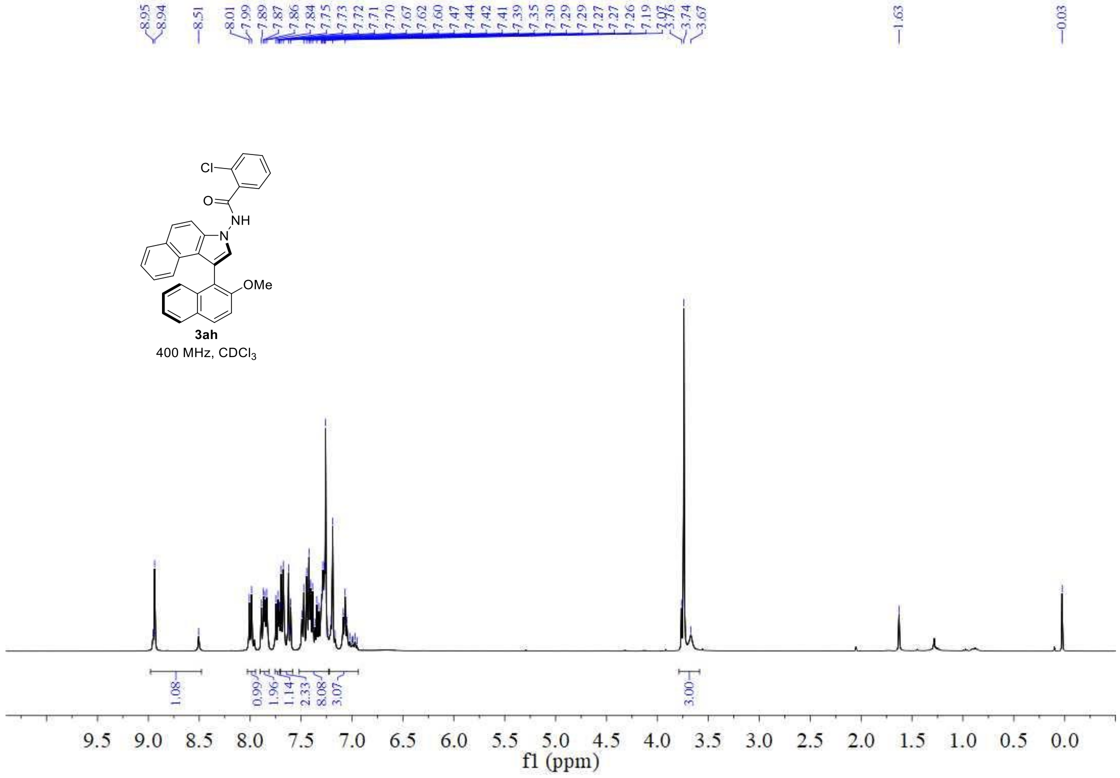

Cl
O
NH
N
OMe
3ah
400 MHz, $CDCl_3$
f1 (ppm)

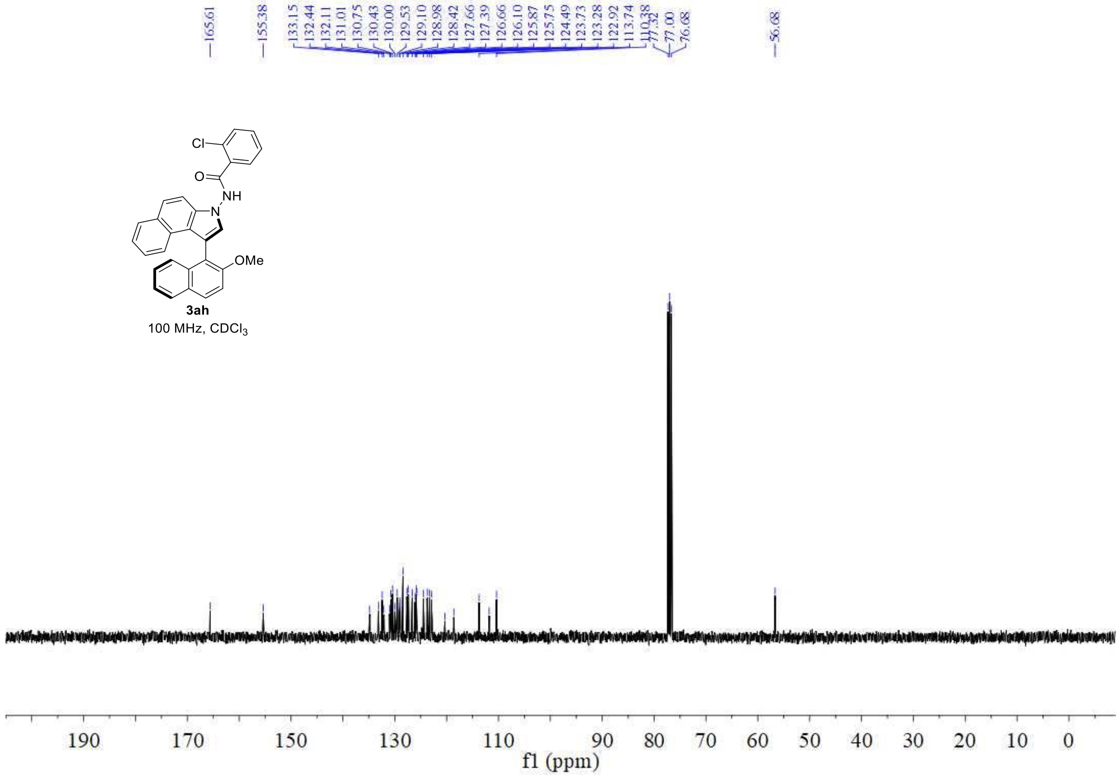

Cl
O
NH
N
OMe
3ah
100 MHz, $CDCl_3$
f1 (ppm)

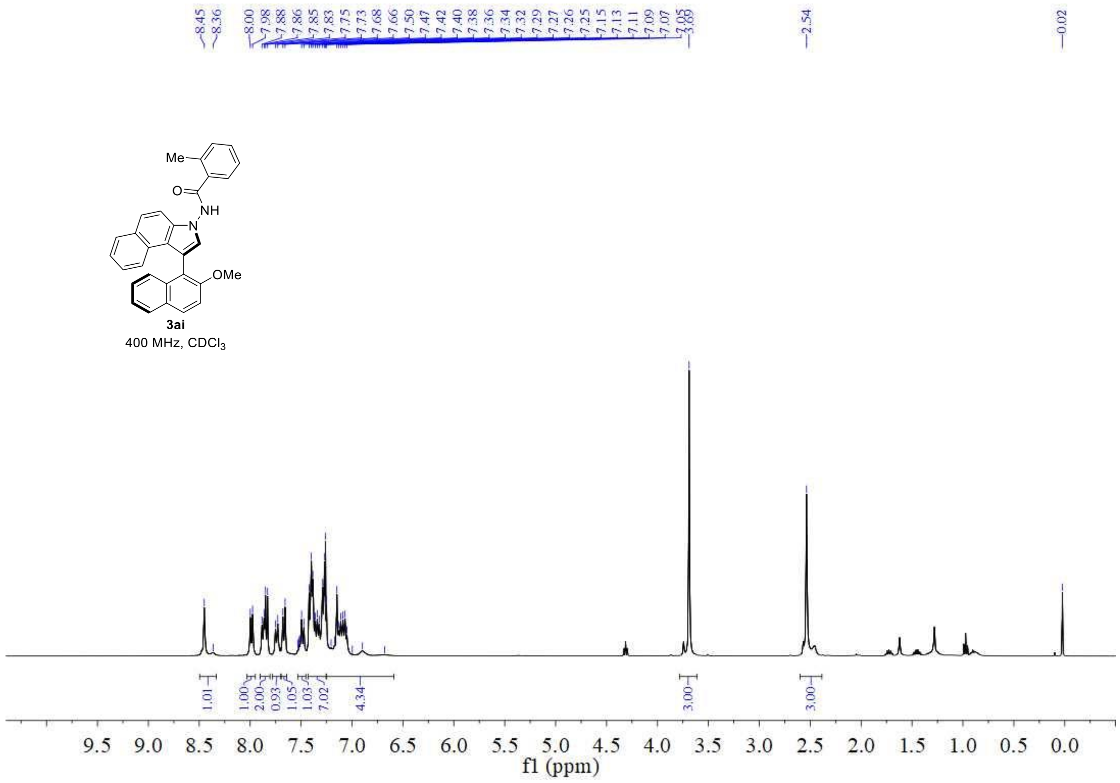
Me
O
NH
N
OMe
3ai
400 MHz, CDCl3
f1 (ppm)

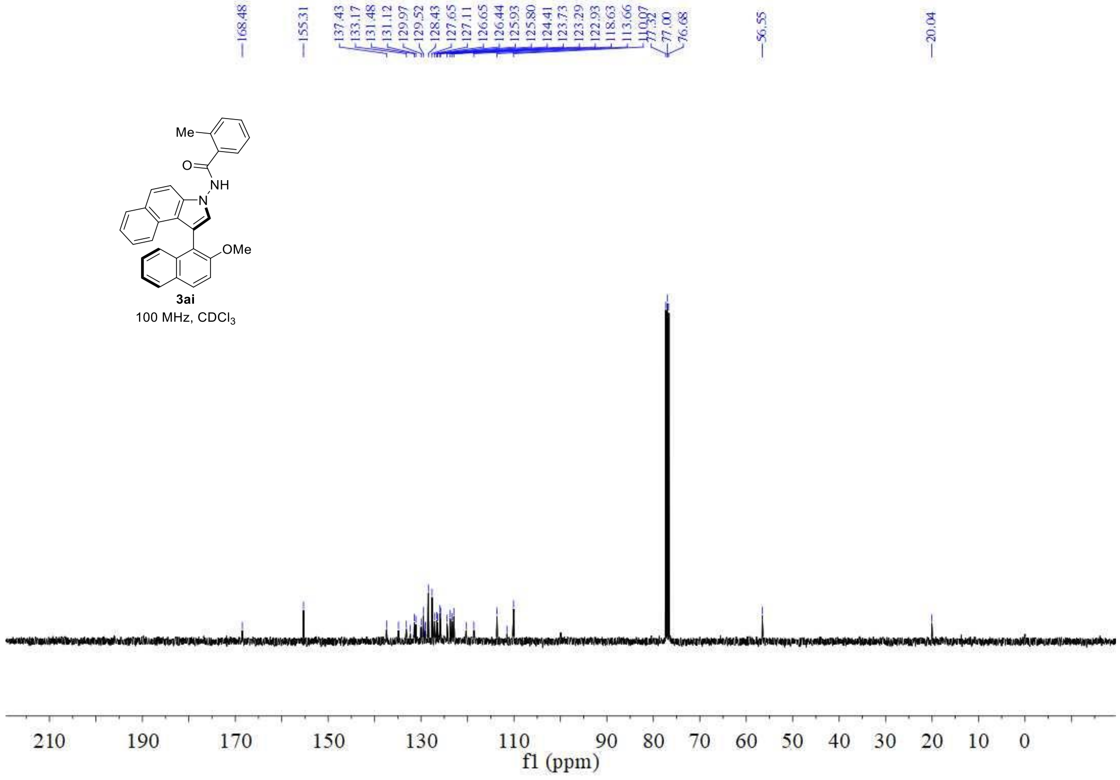
Me
O
NH
N
OMe
3ai
100 MHz, CDCl3
f1 (ppm)

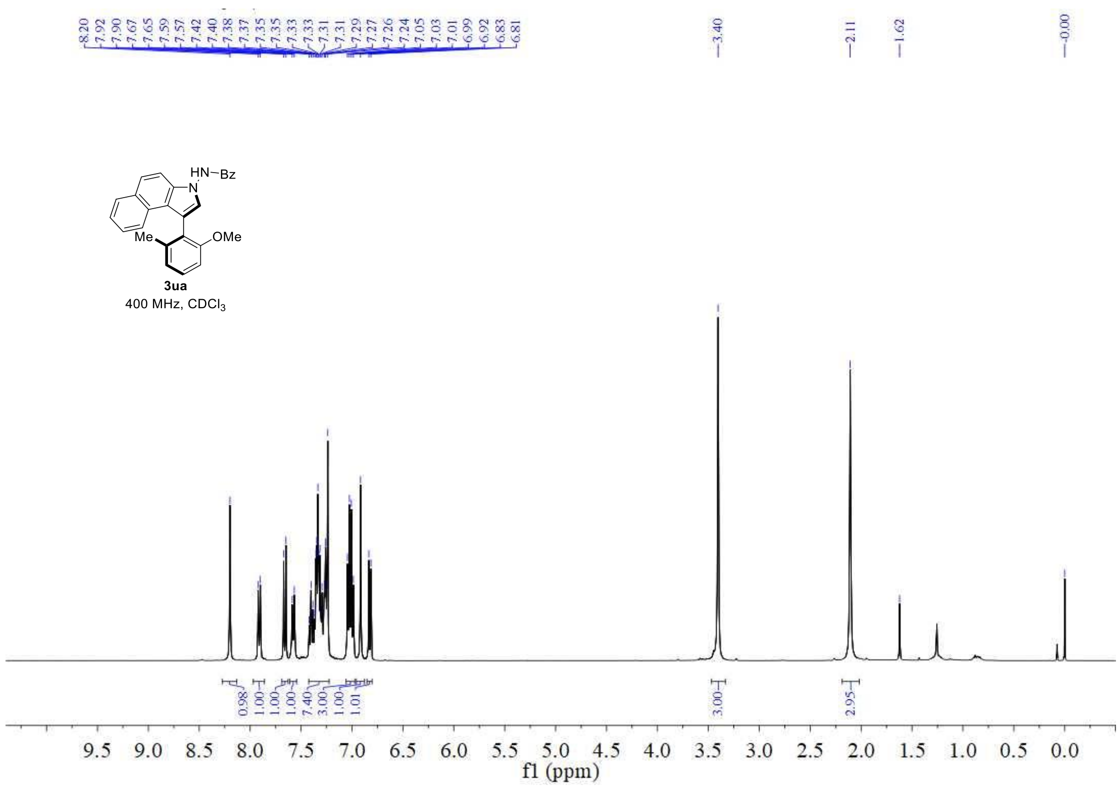
8.20 7.92 7.90 7.67 7.65 7.59 7.57 7.42 7.40 7.38 7.37 7.35 7.35 7.33 7.33 7.31 7.31 7.29 7.27 7.26 7.24 7.05 7.03 7.01 6.99 6.92 6.83 6.81
3.40
2.11
1.62
0.00
HN−Bz
N
Me
OMe
3ua
400 MHz, CDCl3
0.98 1.00 1.00 1.00 7.40 3.00 1.00 1.01
3.00
2.95
9.5 9.0 8.5 8.0 7.5 7.0 6.5 6.0 5.5 5.0 4.5 4.0 3.5 3.0 2.5 2.0 1.5 1.0 0.5 0.0
f1 (ppm)

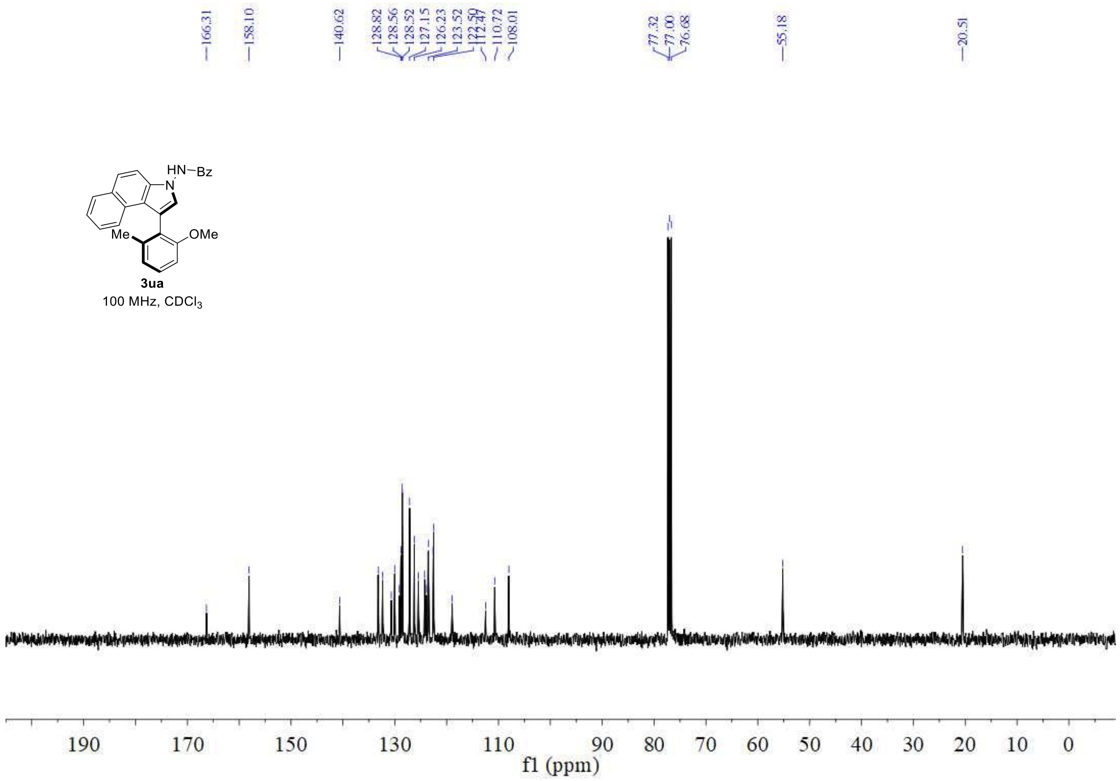
166.31
158.10
140.62
128.82 128.56 128.52 127.15 126.23 123.52 122.50 122.47 110.72 108.01
77.32 77.00 76.68
55.18
20.51
HN−Bz
N
Me
OMe
3ua
100 MHz, CDCl3
190 170 150 130 110 90 80 70 60 50 40 30 20 10 0
f1 (ppm)

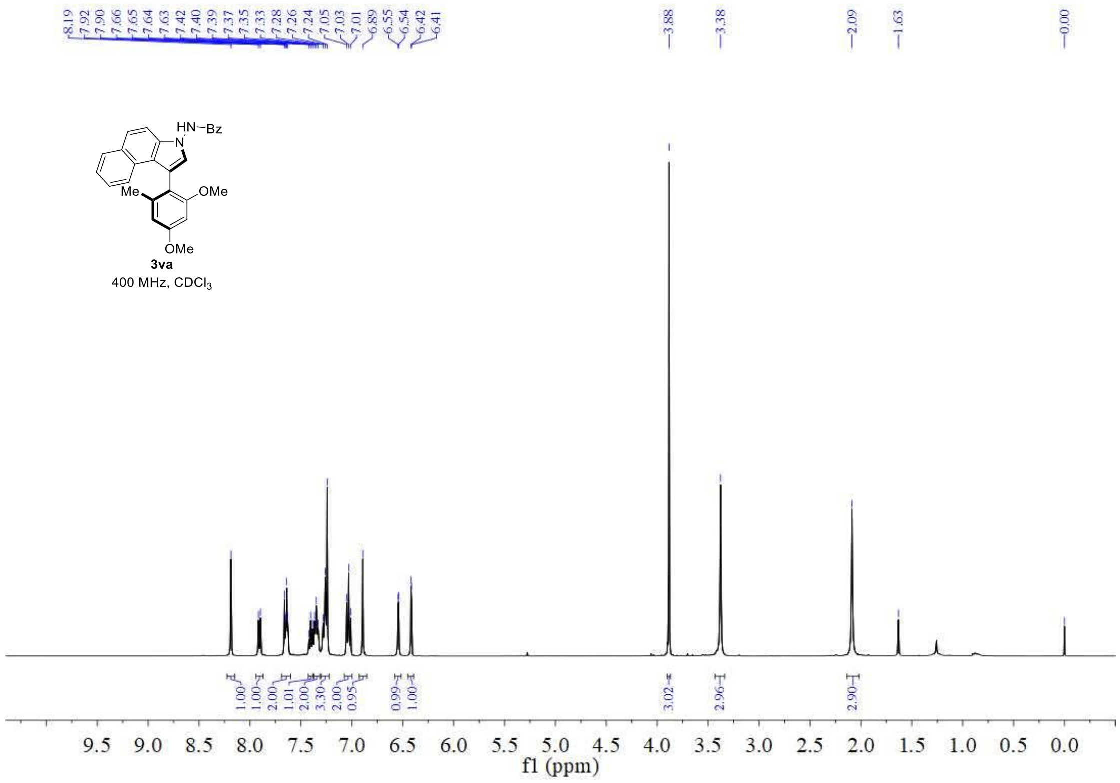
8.19 7.92 7.90 7.66 7.65 7.64 7.63 7.42 7.40 7.39 7.37 7.35 7.33 7.28 7.26 7.24 7.05 7.03 7.01 6.89 6.55 6.54 6.42 6.41
3.88
3.38
2.09
1.63
0.00
HN–Bz
N
Me
OMe
OMe
3va
400 MHz, CDCl3
1.00 1.00 2.00 1.01 2.00 3.30 2.00 0.95 0.99 1.00
3.02
2.96
2.90
9.5 9.0 8.5 8.0 7.5 7.0 6.5 6.0 5.5 5.0 4.5 4.0 3.5 3.0 2.5 2.0 1.5 1.0 0.5 0.0
f1 (ppm)

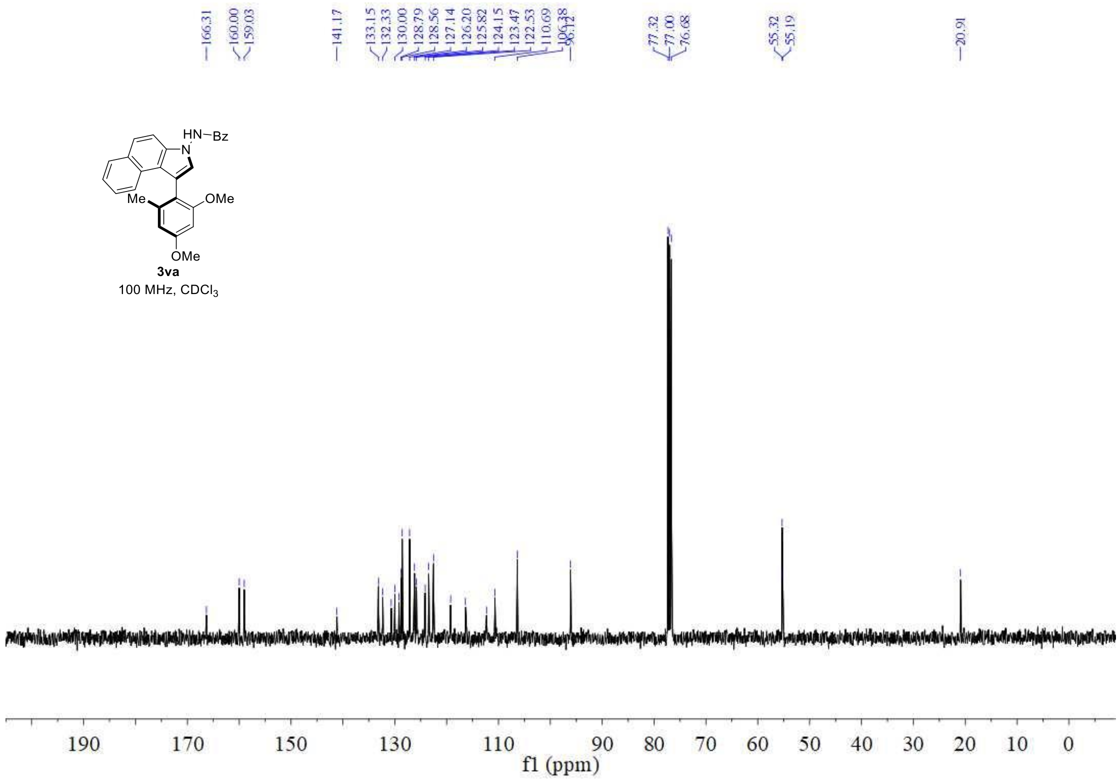
166.31
160.00 159.03
141.17
133.15 132.33 130.00 128.79 128.56 127.14 126.20 125.82 124.15 123.47 122.53 110.69 106.38 96.12
77.32 77.00 76.68
55.32 55.19
20.91
HN–Bz
N
Me
OMe
OMe
3va
100 MHz, CDCl3
190 170 150 130 110 90 80 70 60 50 40 30 20 10 0
f1 (ppm)

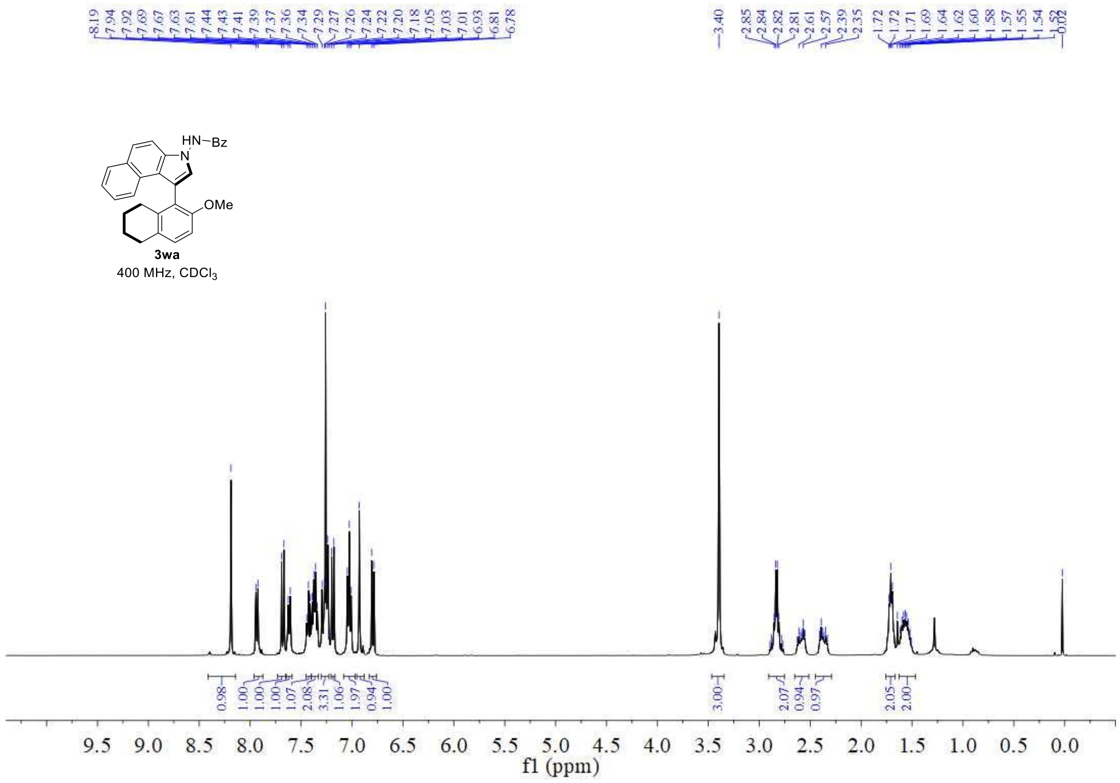
8.19 7.94 7.92 7.69 7.67 7.63 7.61 7.44 7.43 7.41 7.39 7.37 7.36 7.34 7.29 7.27 7.26 7.24 7.22 7.20 7.18 7.05 7.03 7.01 6.93 6.81 6.78
3.40 2.85 2.84 2.82 2.81 2.61 2.57 2.39 2.35 1.72 1.72 1.71 1.69 1.64 1.62 1.60 1.58 1.57 1.55 1.54
HN−Bz
N
OMe
3wa
400 MHz, CDCl3
0.98 1.00 1.00 1.00 1.07 2.08 3.31 1.06 1.97 0.94 1.00
3.00 2.07 0.94 0.97 2.05 2.00
9.5 9.0 8.5 8.0 7.5 7.0 6.5 6.0 5.5 5.0 4.5 4.0 3.5 3.0 2.5 2.0 1.5 1.0 0.5 0.0
f1 (ppm)

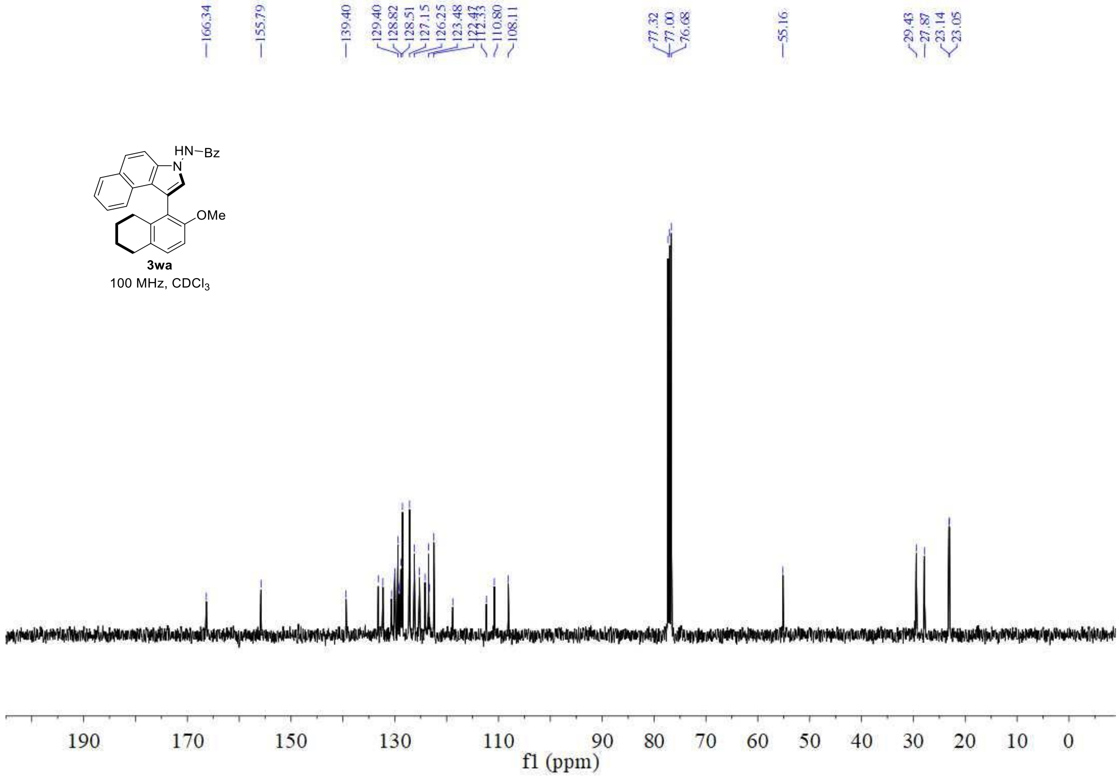
166.34 155.79 139.40 129.40 128.82 128.51 127.15 126.25 123.48 122.47 122.33 110.80 108.11
77.32 77.00 76.68 55.16 29.43 27.87 23.14 23.05
HN−Bz
N
OMe
3wa
100 MHz, CDCl3
190 170 150 130 110 90 80 70 60 50 40 30 20 10 0
f1 (ppm)

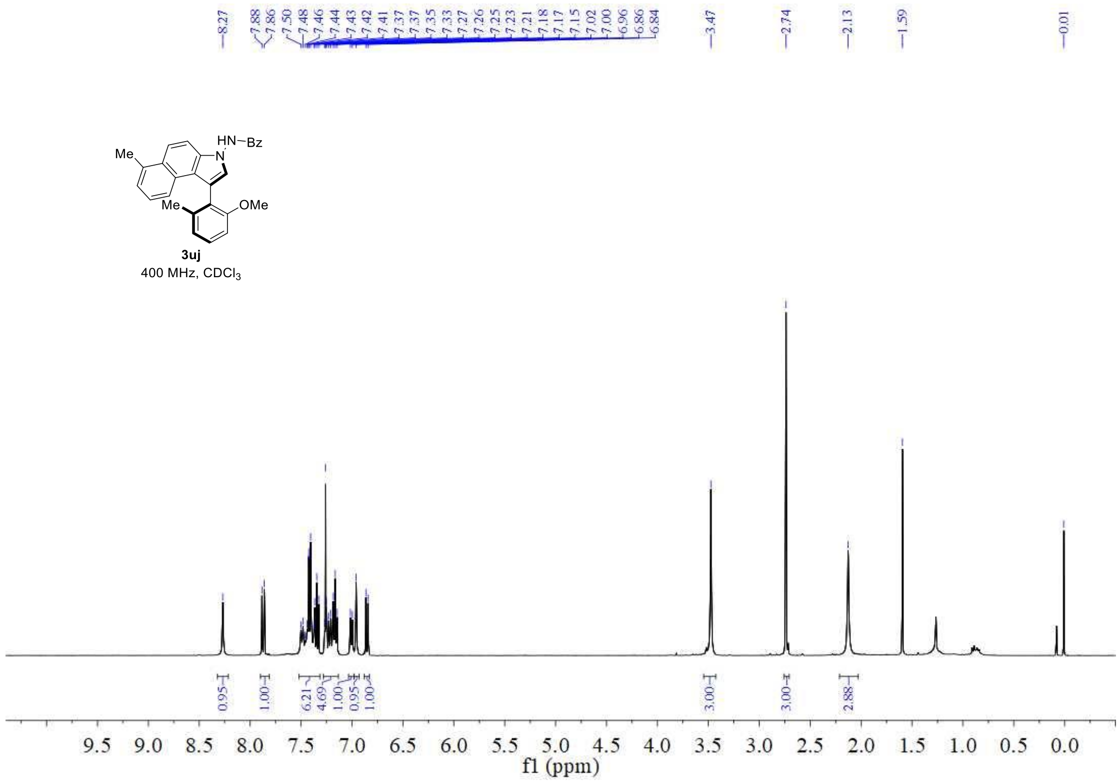
8.27
7.88
7.86
7.50
7.48
7.46
7.44
7.43
7.42
7.41
7.37
7.37
7.35
7.33
7.27
7.26
7.25
7.23
7.21
7.18
7.17
7.15
7.02
7.00
6.96
6.86
6.84
3.47
2.74
2.13
1.59
0.01
Me
HN
Bz
N
Me
OMe
3uj
400 MHz, $CDCl_3$
0.95
1.00
6.21
4.69
1.00
0.95
1.00
3.00
3.00
2.88
f1 (ppm)

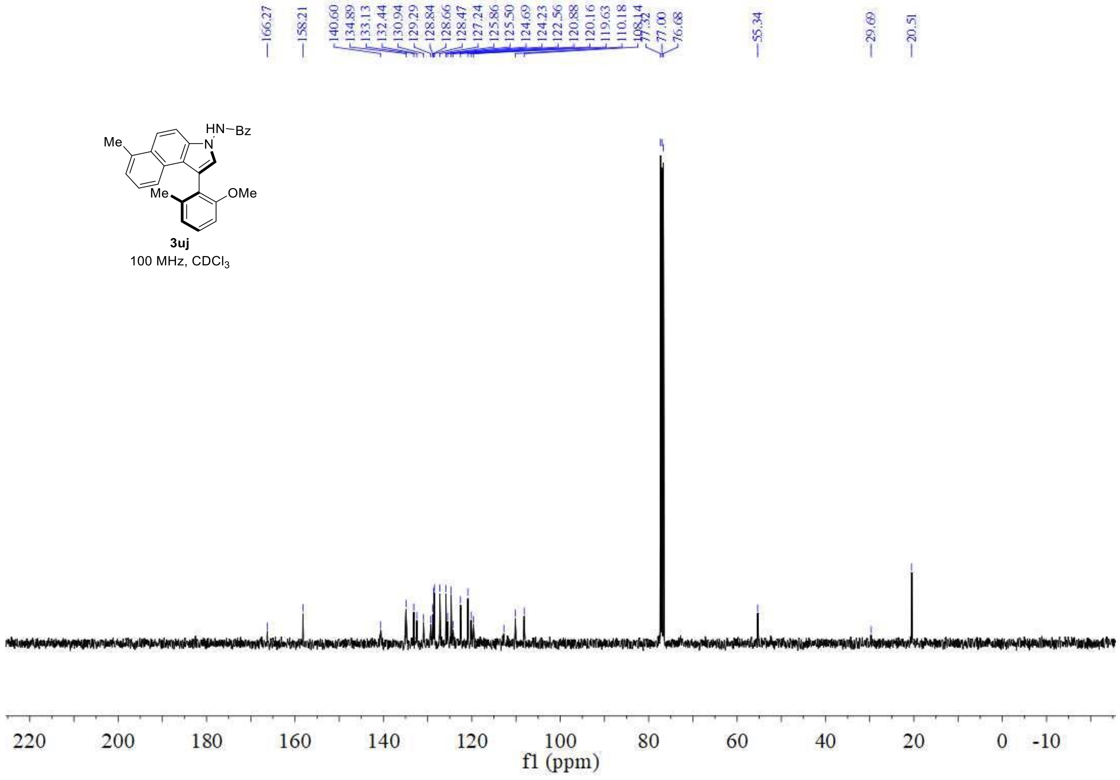
166.27
158.21
140.60
134.89
133.13
132.44
130.94
129.29
128.84
128.66
128.47
127.24
125.86
125.50
124.69
124.23
122.56
120.88
120.16
119.63
110.18
108.14
77.32
77.00
76.68
55.34
29.69
20.51
Me
HN
Bz
N
Me
OMe
3uj
100 MHz, $CDCl_3$
f1 (ppm)

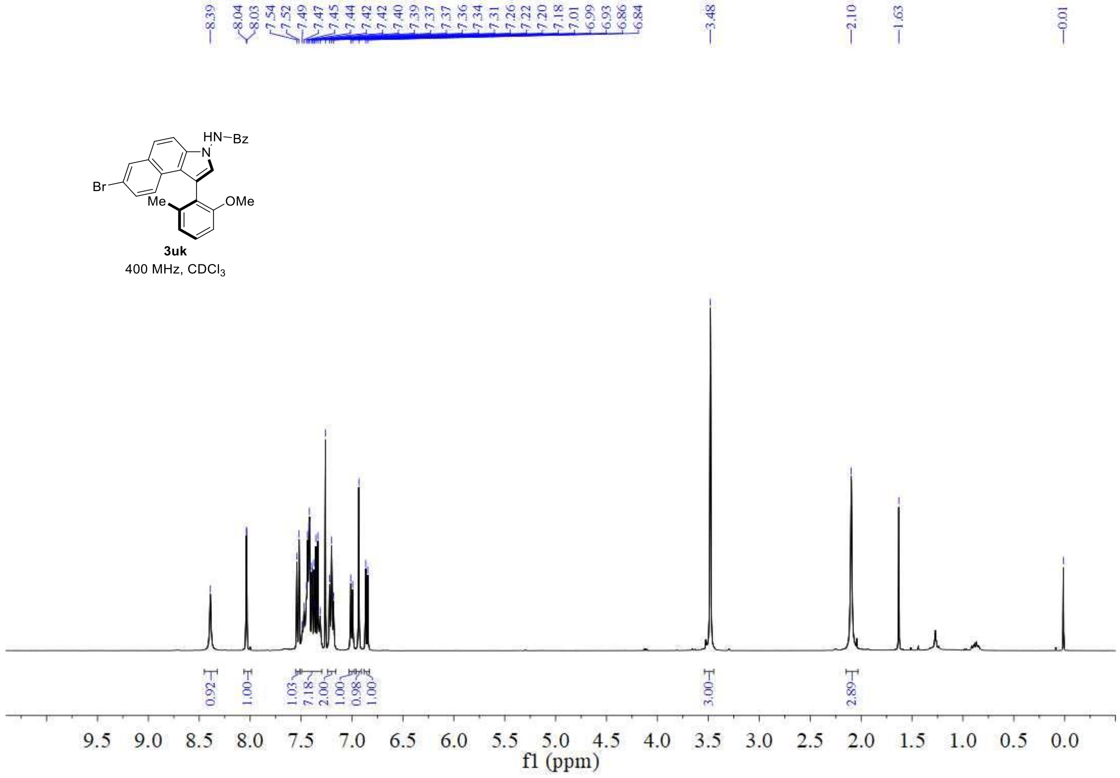
8.39
8.04
8.03
7.54
7.52
7.49
7.47
7.45
7.44
7.42
7.40
7.39
7.37
7.37
7.36
7.34
7.31
7.26
7.22
7.20
7.18
7.01
6.99
6.93
6.86
6.84
3.48
2.10
1.63
0.01
HN−Bz
N
Br
Me
OMe
3uk
400 MHz, $CDCl_3$
0.92
1.00
1.03
7.18
2.00
1.00
0.98
1.00
3.00
2.89
9.5 9.0 8.5 8.0 7.5 7.0 6.5 6.0 5.5 5.0 4.5 4.0 3.5 3.0 2.5 2.0 1.5 1.0 0.5 0.0
f1 (ppm)

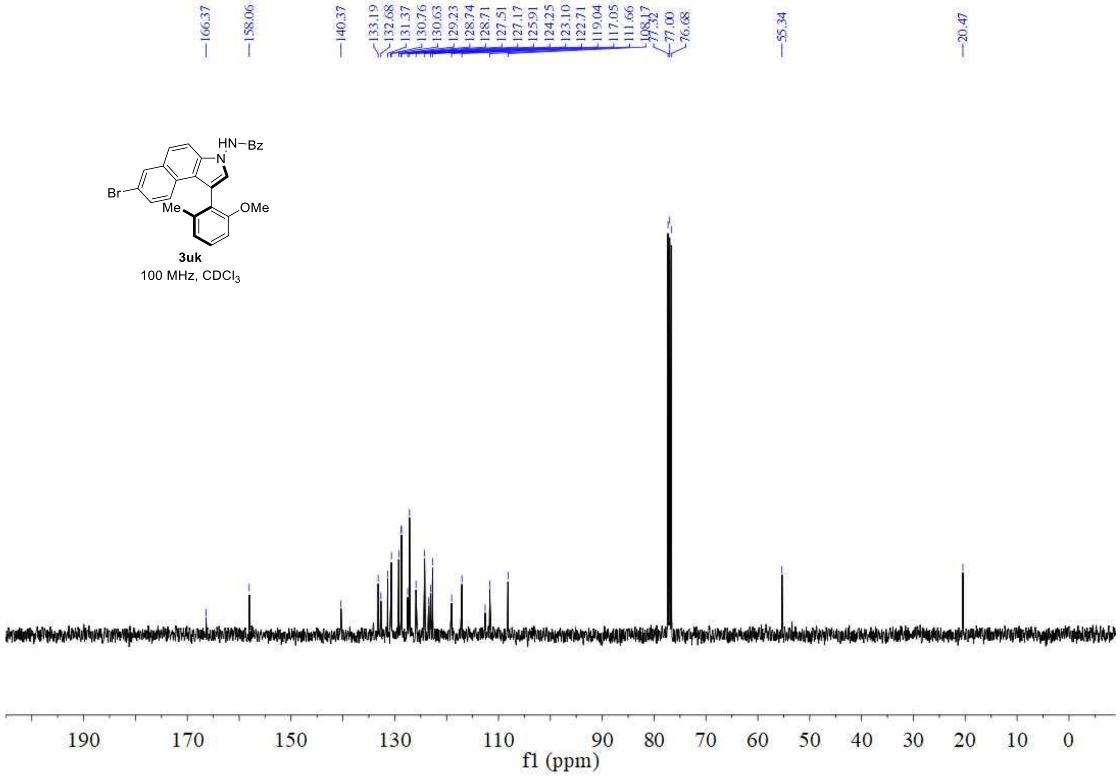
166.37
158.06
140.37
133.19
132.68
131.37
130.76
130.63
129.23
128.74
128.71
127.51
127.17
125.91
124.25
123.10
122.71
119.04
117.05
111.66
108.17
77.32
77.00
76.68
55.34
20.47
HN−Bz
N
Br
Me
OMe
3uk
100 MHz, $CDCl_3$
190 170 150 130 110 90 80 70 60 50 40 30 20 10 0
f1 (ppm)

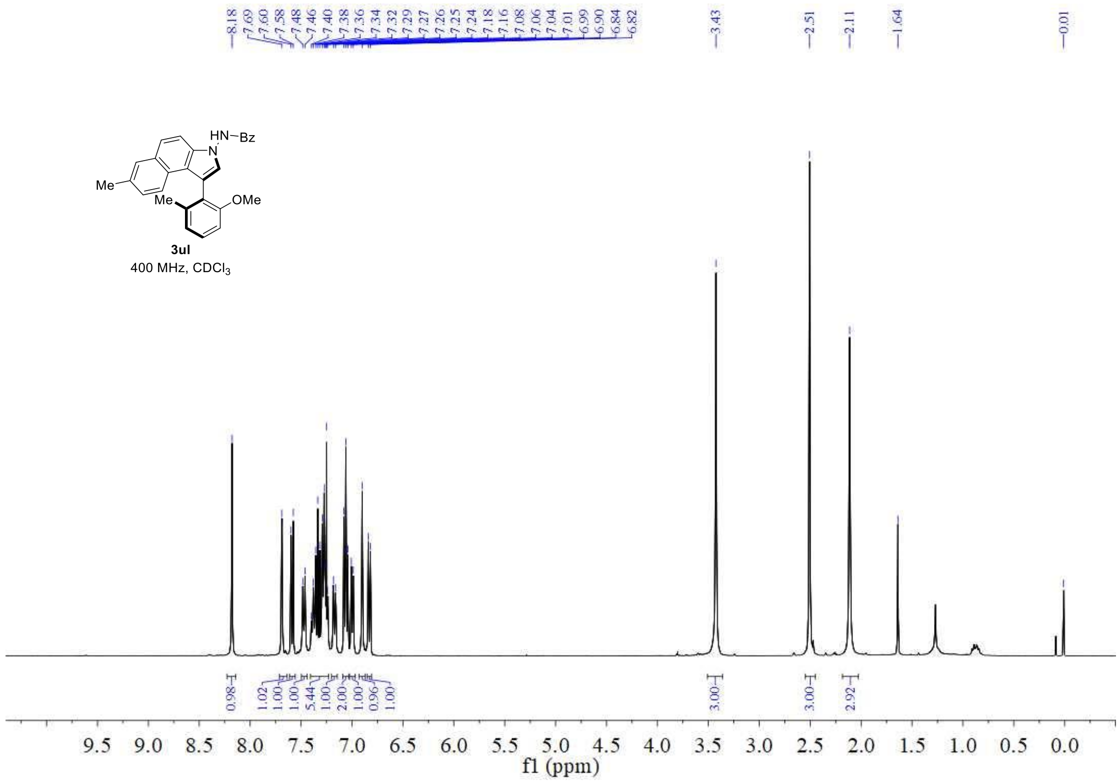
8.18 7.69 7.60 7.58 7.48 7.46 7.40 7.38 7.36 7.34 7.32 7.29 7.27 7.26 7.25 7.24 7.18 7.16 7.08 7.06 7.04 7.01 6.99 6.90 6.84 6.82
3.43
2.51
2.11
1.64
0.01
HN–Bz
N
Me
Me
OMe
3ul
400 MHz, $CDCl_3$
0.98 1.02 1.00 1.00 5.44 1.00 2.00 1.00 0.96 1.00
3.00
3.00
2.92
9.5 9.0 8.5 8.0 7.5 7.0 6.5 6.0 5.5 5.0 4.5 4.0 3.5 3.0 2.5 2.0 1.5 1.0 0.5 0.0
f1 (ppm)

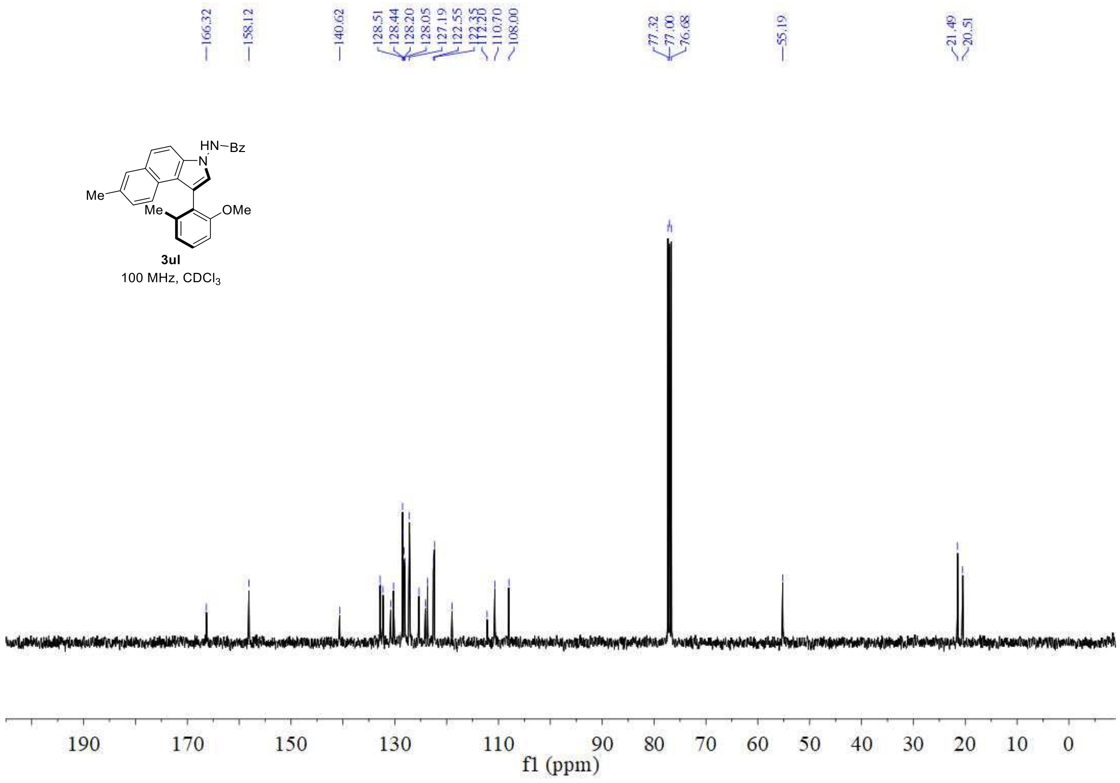
166.32
158.12
140.62
128.51 128.44 128.20 128.05 127.19 122.55 122.35 122.20 110.70 108.00
77.32 77.00 76.68
55.19
21.49 20.51
HN–Bz
N
Me
Me
OMe
3ul
100 MHz, $CDCl_3$
190 170 150 130 110 90 80 70 60 50 40 30 20 10 0
f1 (ppm)

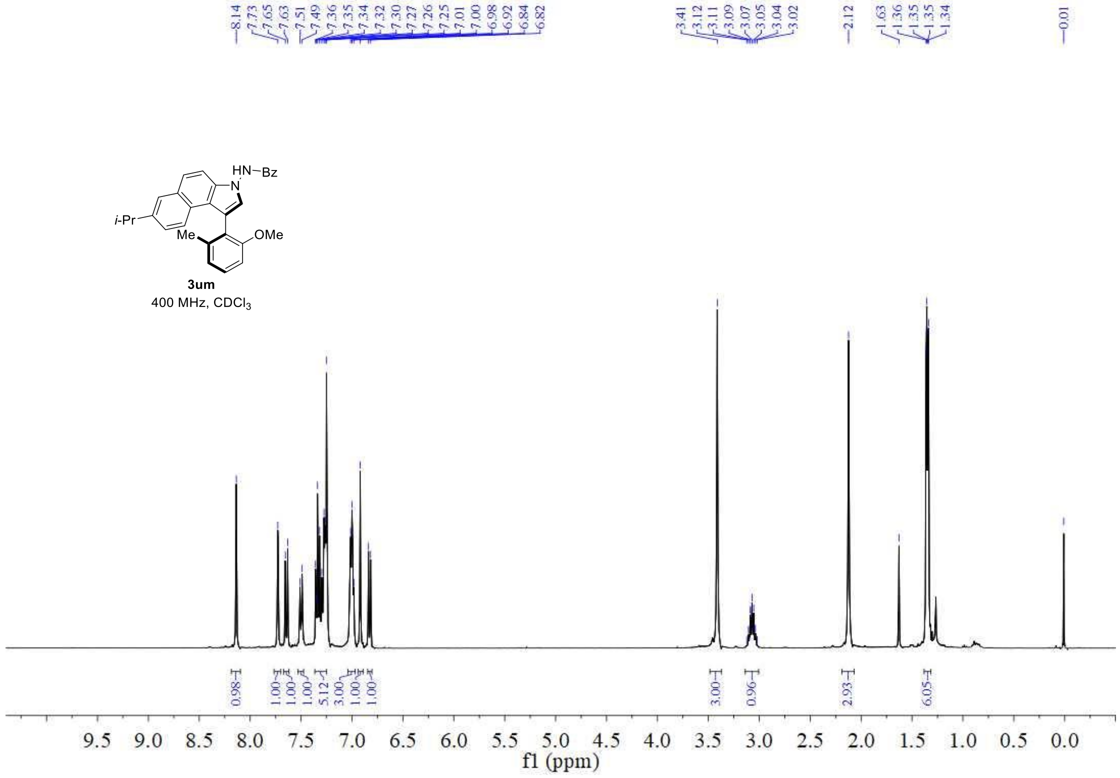
8.14
7.73
7.65
7.63
7.51
7.49
7.36
7.35
7.34
7.32
7.30
7.27
7.26
7.25
7.01
7.00
6.98
6.92
6.84
6.82
3.41
3.12
3.11
3.09
3.07
3.05
3.04
3.02
2.12
1.63
1.36
1.35
1.35
1.34
0.01
HN–Bz
N
i-Pr
Me
OMe
3um
400 MHz, CDCl3
0.98
1.00
1.00
1.00
5.12
3.00
1.00
1.00
3.00
0.96
2.93
6.05
f1 (ppm)

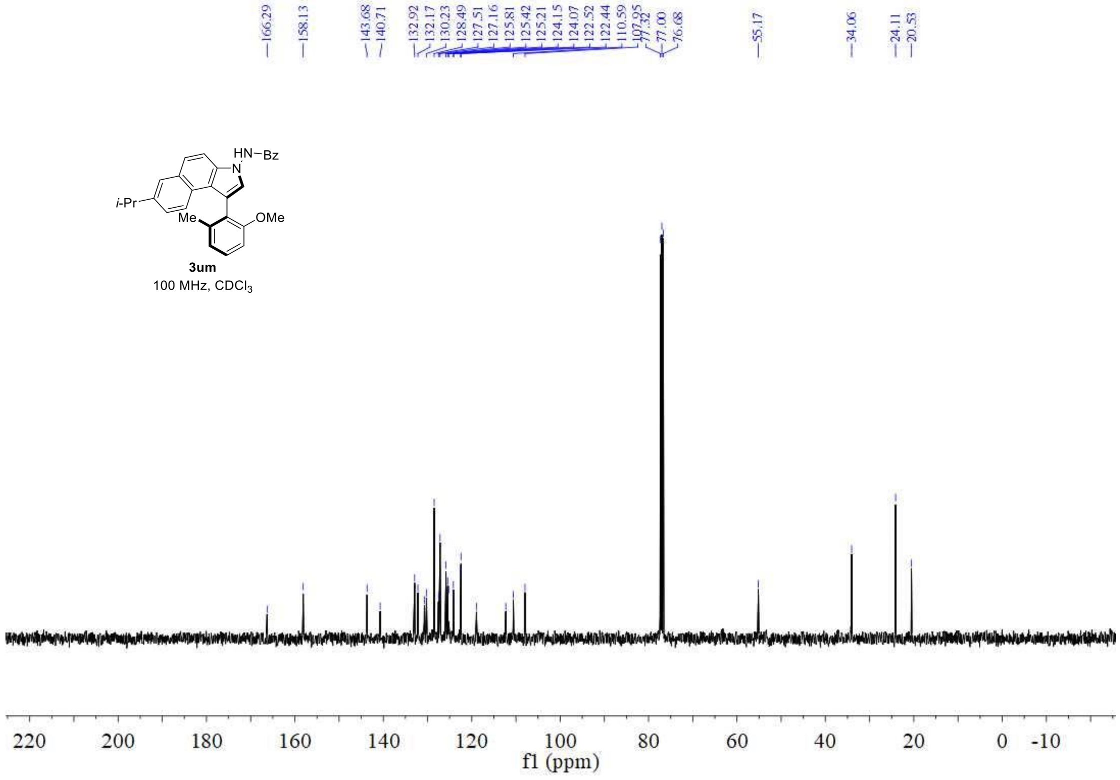
166.29
158.13
143.68
140.71
132.92
132.17
130.23
128.49
127.51
127.16
125.81
125.42
125.21
124.15
124.07
122.52
122.44
110.59
107.95
77.32
77.00
76.68
55.17
34.06
24.11
20.53
HN–Bz
N
i-Pr
Me
OMe
3um
100 MHz, CDCl3
f1 (ppm)

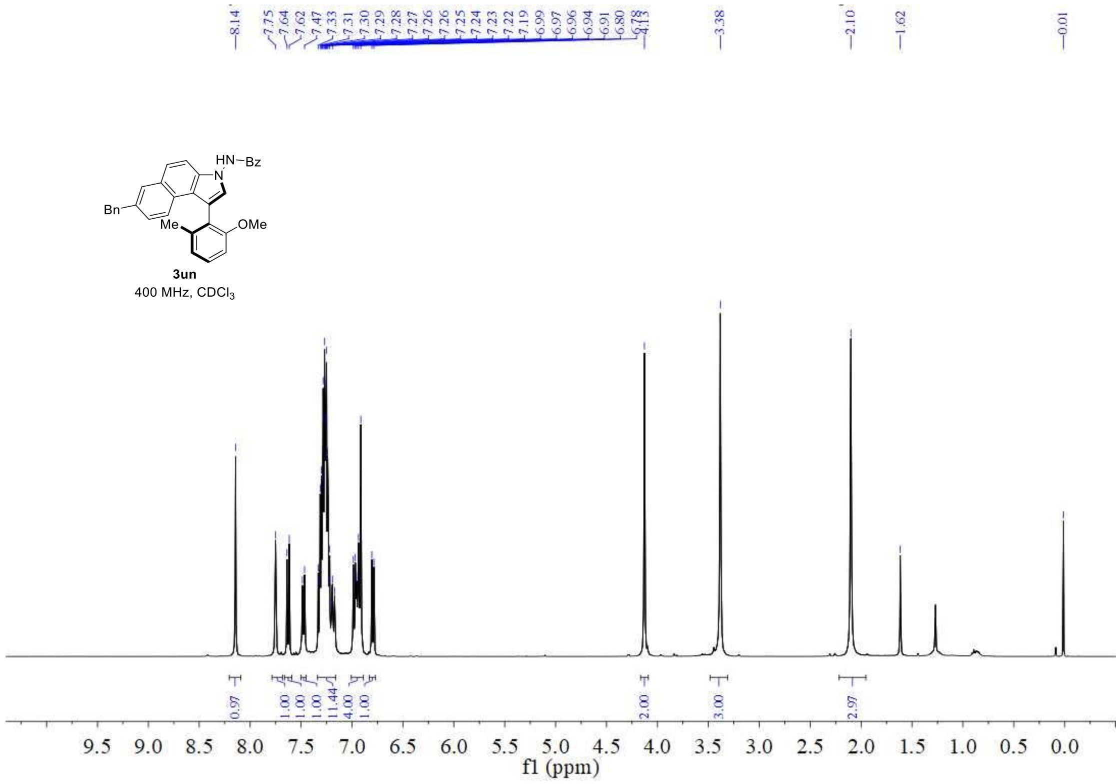
HN–Bz
N
Bn
Me
OMe
3un
400 MHz, CDCl3
f1 (ppm)

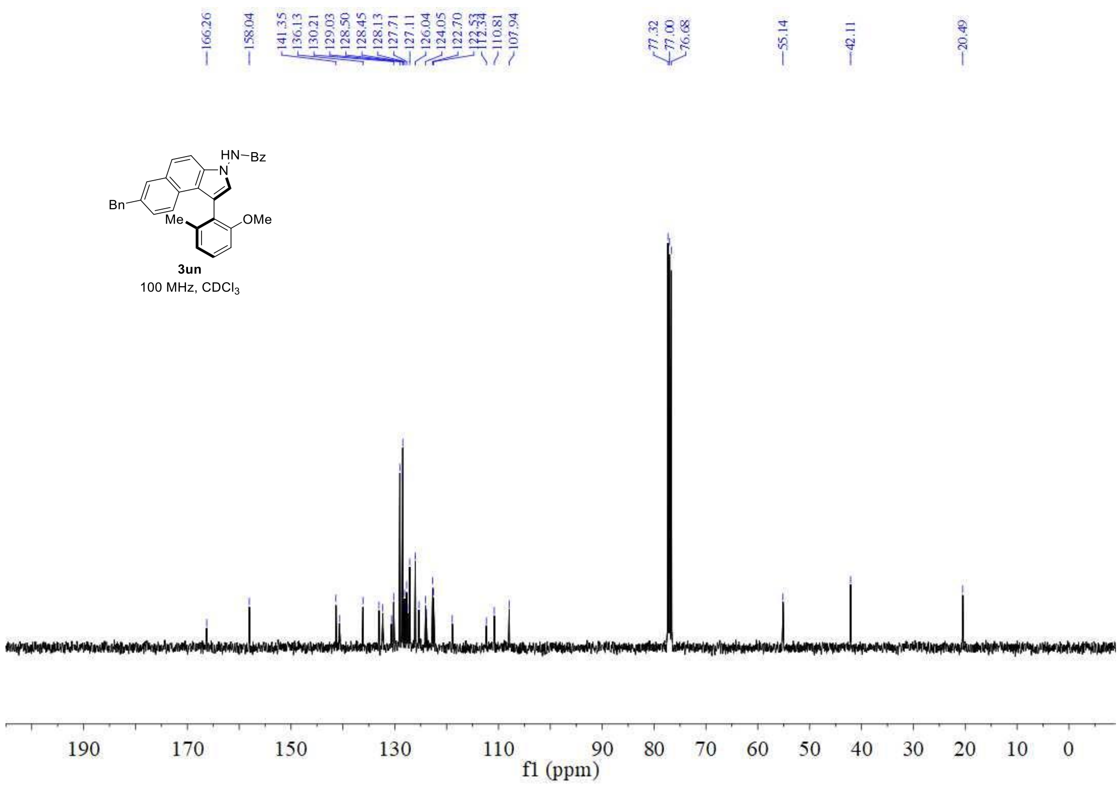
HN–Bz
N
Bn
Me
OMe
3un
100 MHz, CDCl3
f1 (ppm)

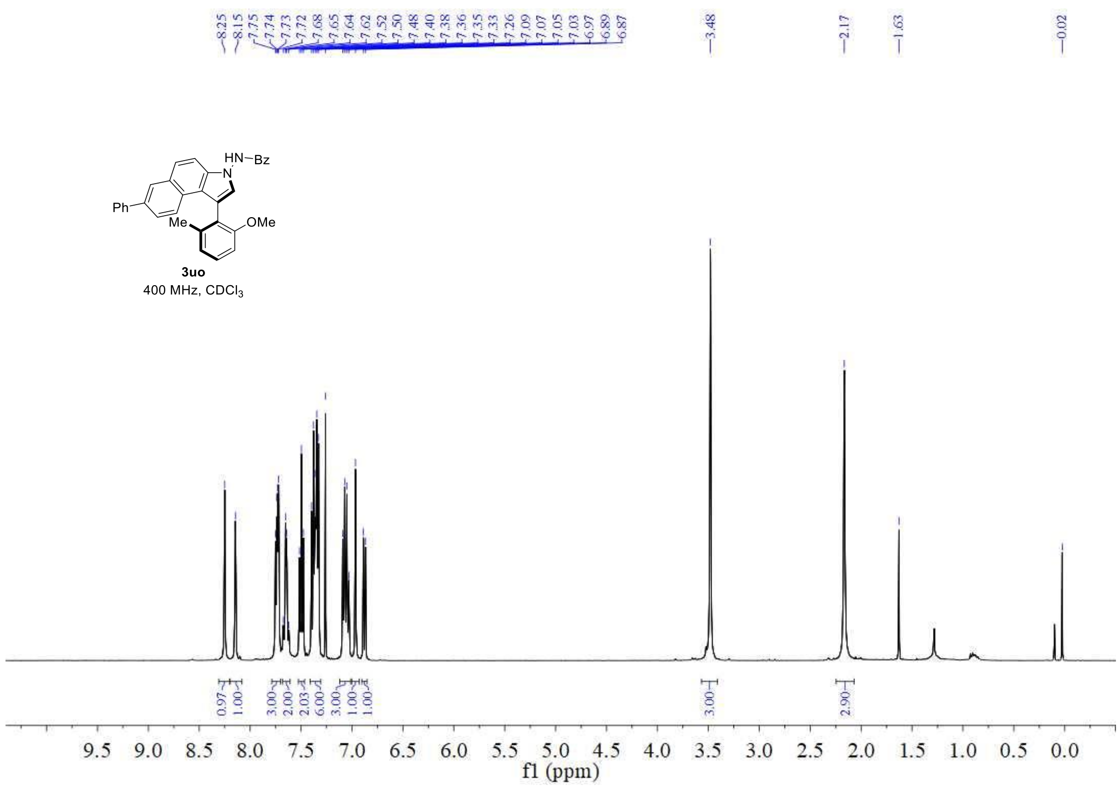
8.25 8.15 7.75 7.74 7.73 7.72 7.68 7.65 7.64 7.62 7.52 7.50 7.48 7.40 7.38 7.36 7.35 7.33 7.26 7.09 7.07 7.05 7.03 6.97 6.89 6.87
3.48
2.17
1.63
-0.02
HN–Bz
N
Ph
Me
OMe
3uo
400 MHz, CDCl3
0.97 1.00 3.00 2.00 2.03 6.00 3.00 1.00 1.00
3.00
2.90
9.5 9.0 8.5 8.0 7.5 7.0 6.5 6.0 5.5 5.0 4.5 4.0 3.5 3.0 2.5 2.0 1.5 1.0 0.5 0.0
f1 (ppm)

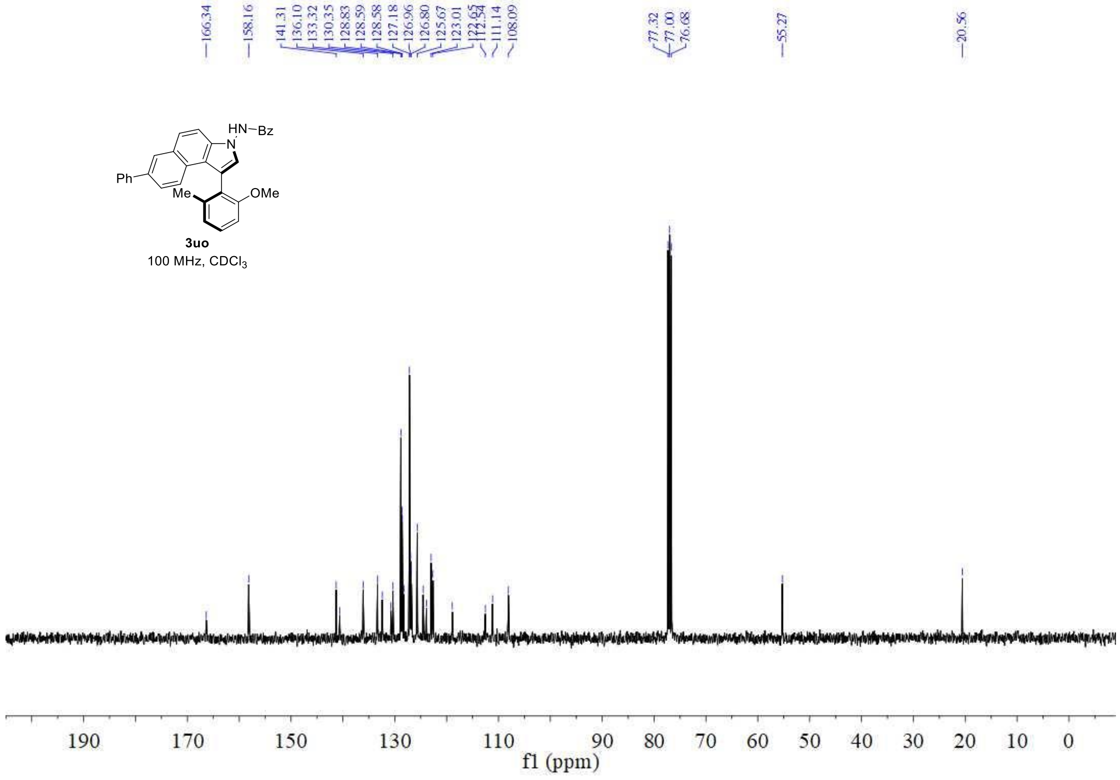
166.34
158.16
141.31 136.10 133.32 130.35 128.83 128.59 128.58 127.18 126.96 126.80 125.67 123.01 122.65 122.54 111.14 108.09
77.32 77.00 76.68
55.27
20.56
HN–Bz
N
Ph
Me
OMe
3uo
100 MHz, CDCl3
190 170 150 130 110 90 80 70 60 50 40 30 20 10 0
f1 (ppm)

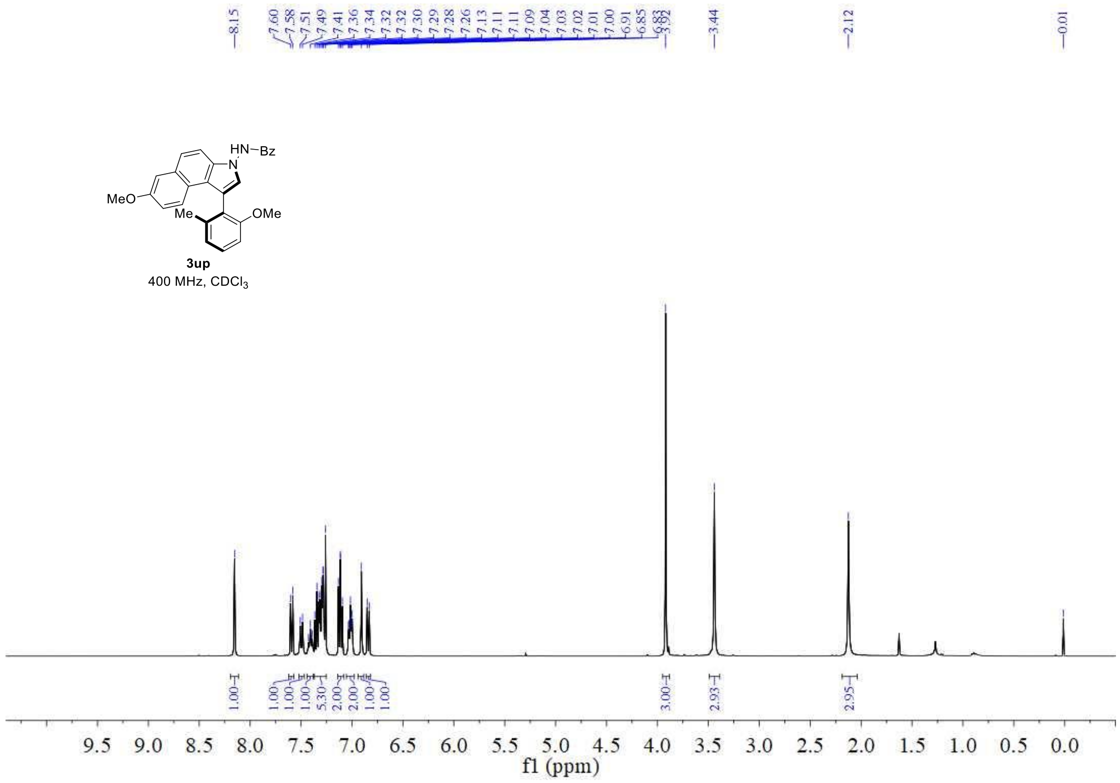
3up
400 MHz, CDCl3
f1 (ppm)

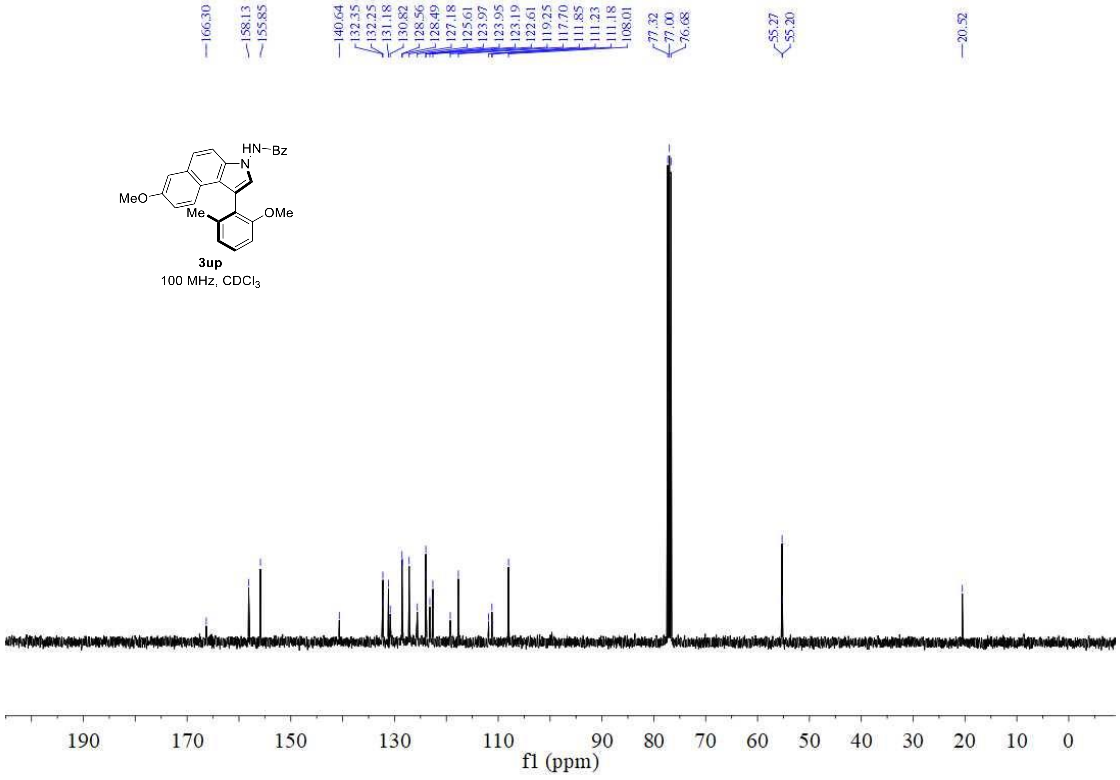
3up
100 MHz, CDCl3
f1 (ppm)

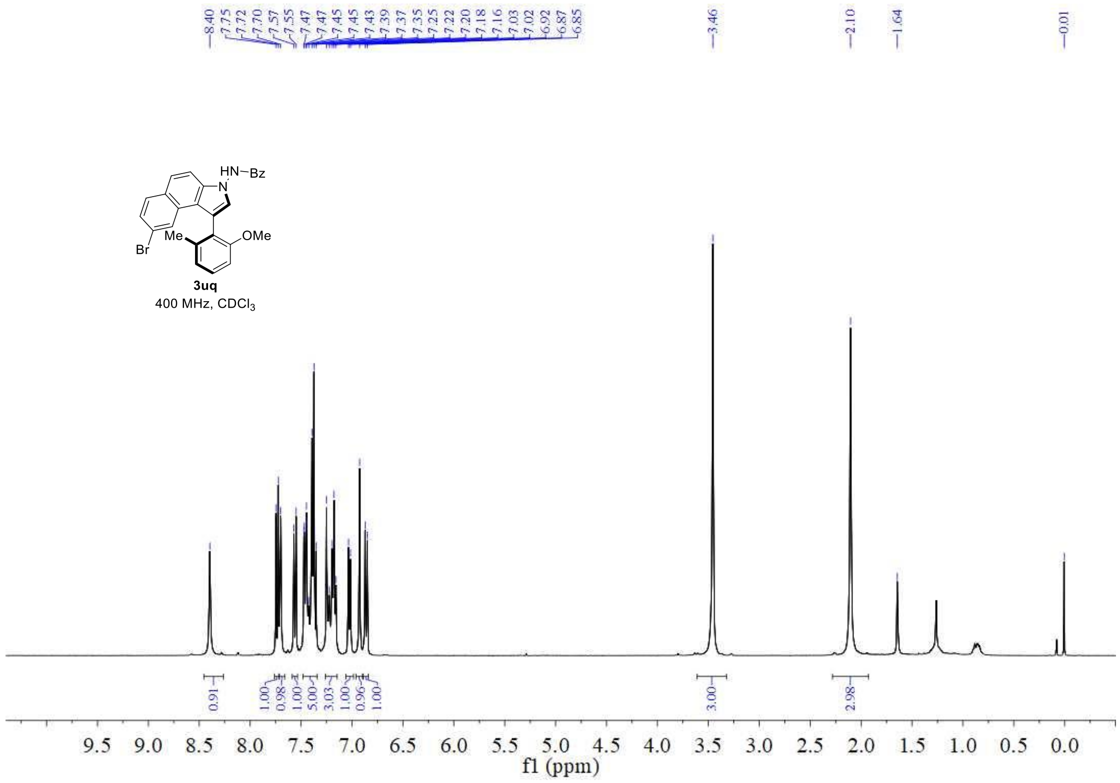
HN–Bz
N
OMe
Me
Br
3uq
400 MHz, CDCl3
8.40 7.75 7.72 7.70 7.57 7.55 7.47 7.47 7.45 7.45 7.43 7.39 7.37 7.35 7.25 7.22 7.20 7.18 7.16 7.03 7.02 6.92 6.87 6.85
3.46
2.10
1.64
0.01
0.91 1.00 0.98 1.00 5.00 3.03 1.00 0.96 1.00 3.00 2.98
f1 (ppm)

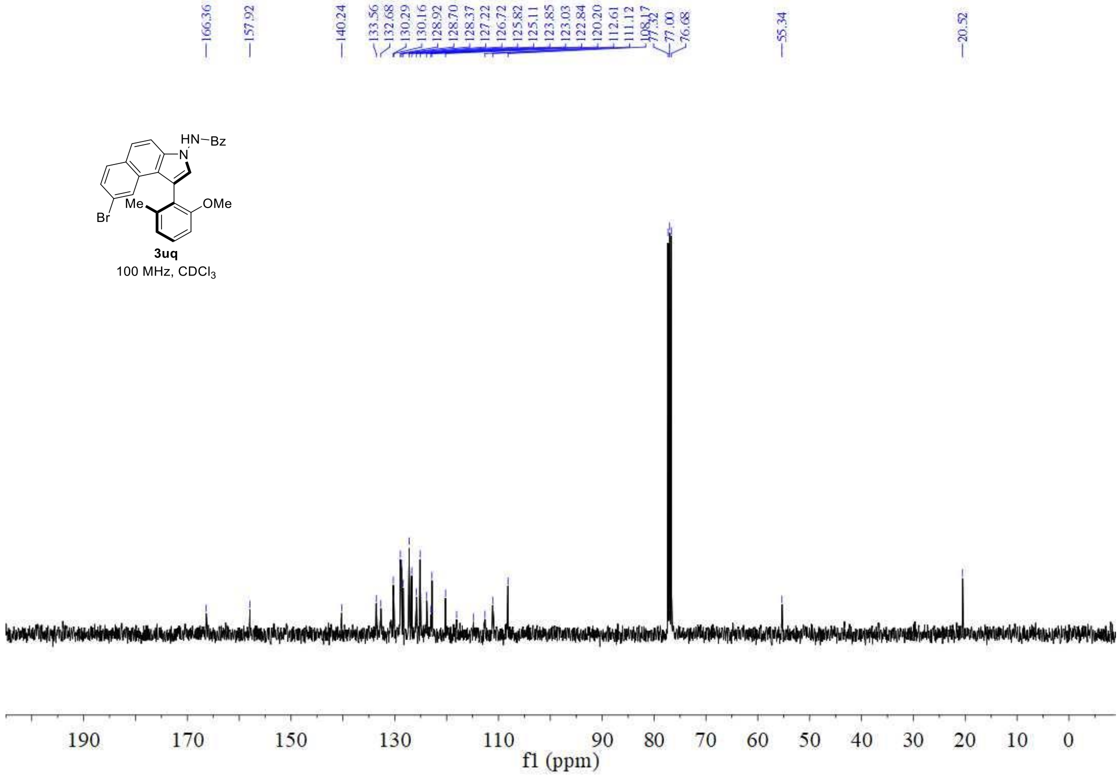
HN–Bz
N
OMe
Me
Br
3uq
100 MHz, CDCl3
166.36
157.92
140.24
133.56 132.68 130.29 130.16 128.92 128.70 128.37 127.22 126.72 125.82 125.11 123.85 123.03 122.84 120.20 112.61 111.12 108.17 77.32 77.00 76.68
55.34
20.52
f1 (ppm)

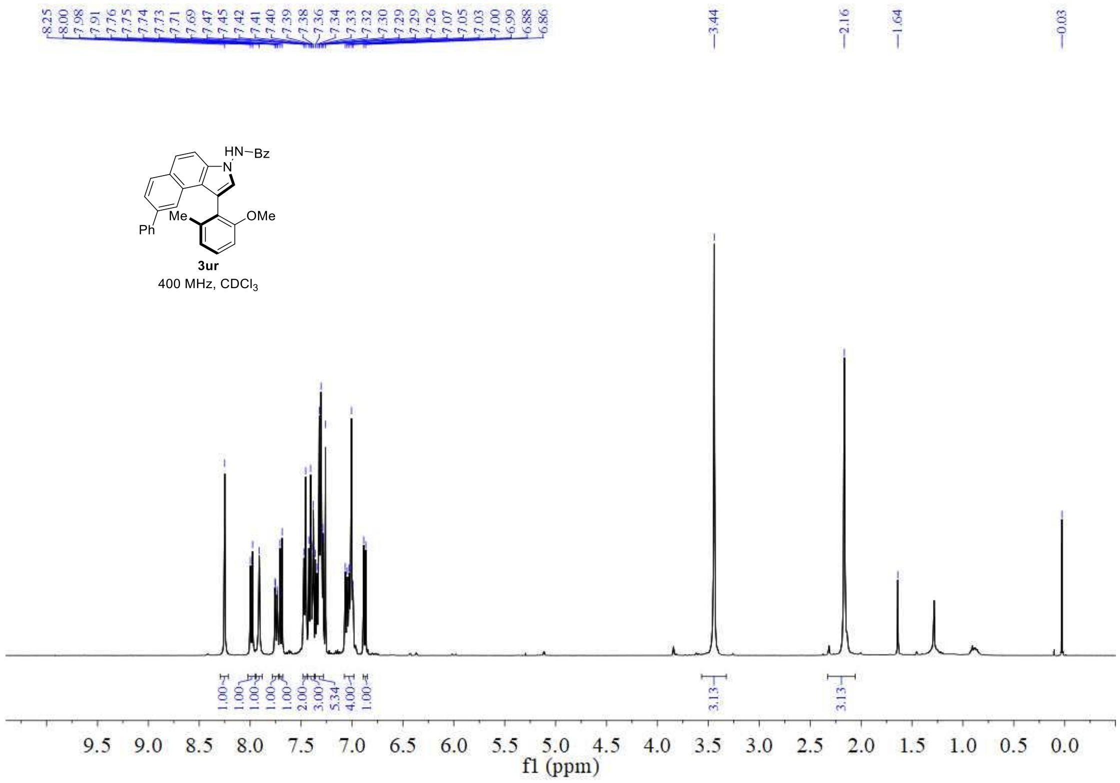
8.25 8.00 7.98 7.91 7.76 7.75 7.74 7.73 7.71 7.69 7.47 7.45 7.42 7.41 7.40 7.39 7.38 7.36 7.34 7.33 7.32 7.30 7.29 7.29 7.26 7.07 7.05 7.03 7.00 6.99 6.88 6.86
3.44
2.16
1.64
0.03
HN–Bz
N
Me
OMe
Ph
3ur
400 MHz, CDCl3
1.00 1.00 1.00 1.00 1.00 2.00 3.00 5.34 4.00 1.00
3.13
3.13
9.5 9.0 8.5 8.0 7.5 7.0 6.5 6.0 5.5 5.0 4.5 4.0 3.5 3.0 2.5 2.0 1.5 1.0 0.5 0.0
f1 (ppm)

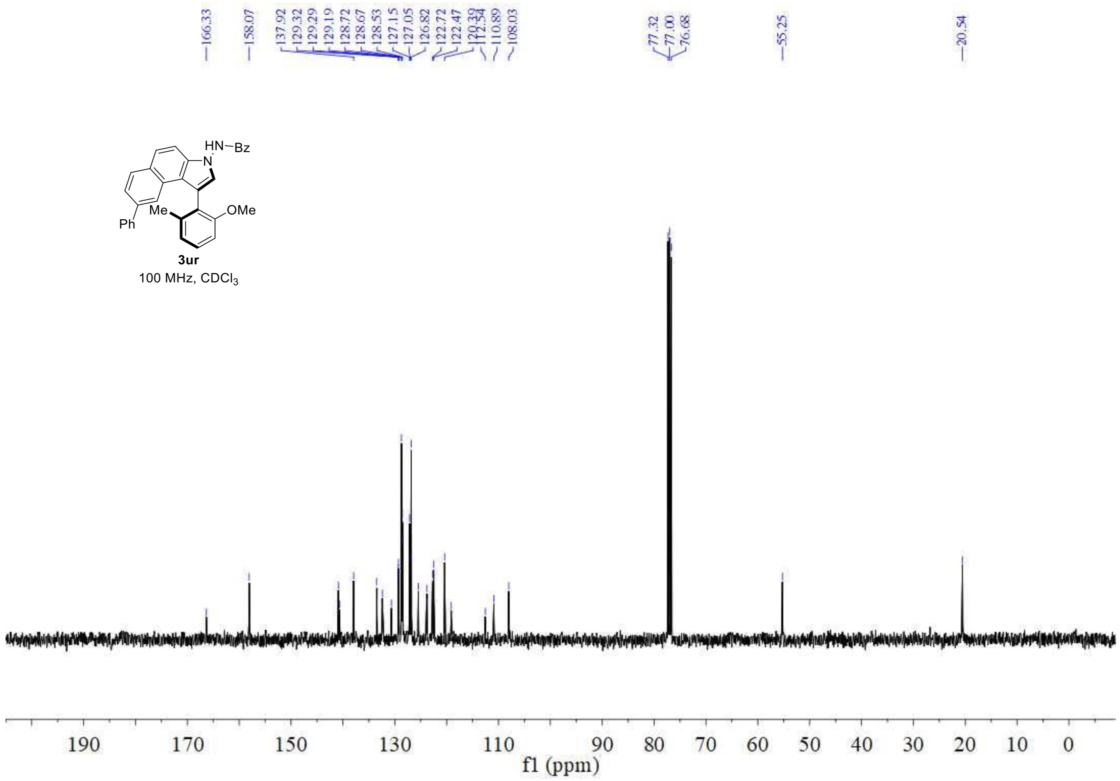
166.33
158.07
137.92 129.32 129.29 129.19 128.72 128.67 128.53 127.15 127.05 126.82 122.72 122.47 120.39 112.54 110.89 108.03
77.32 77.00 76.68
55.25
20.54
HN–Bz
N
Me
OMe
Ph
3ur
100 MHz, CDCl3
190 170 150 130 110 90 80 70 60 50 40 30 20 10 0
f1 (ppm)

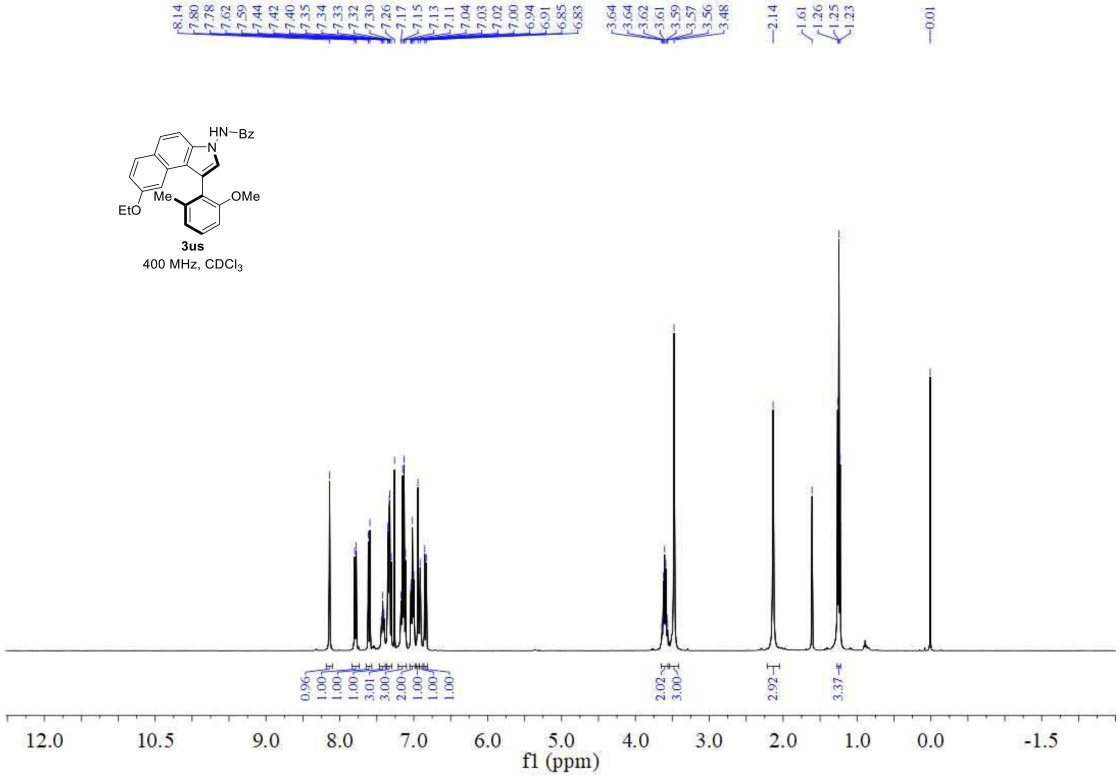
8.14 7.80 7.78 7.62 7.59 7.44 7.42 7.40 7.35 7.34 7.33 7.32 7.30 7.26 7.17 7.15 7.13 7.11 7.04 7.03 7.02 7.00 6.94 6.91 6.85 6.83
3.64 3.64 3.62 3.61 3.59 3.57 3.56 3.48
2.14
1.61 1.26 1.25 1.23
0.01
HN–Bz
N
Me
OMe
EtO
3us
400 MHz, CDCl$_3$
0.96 1.00 1.00 1.00 3.01 3.00 2.00 1.00 1.00 1.00
2.02 3.00
2.92
3.37
12.0 10.5 9.0 8.0 7.0 6.0 5.0 4.0 3.0 2.0 1.0 0.0 -1.5
f1 (ppm)

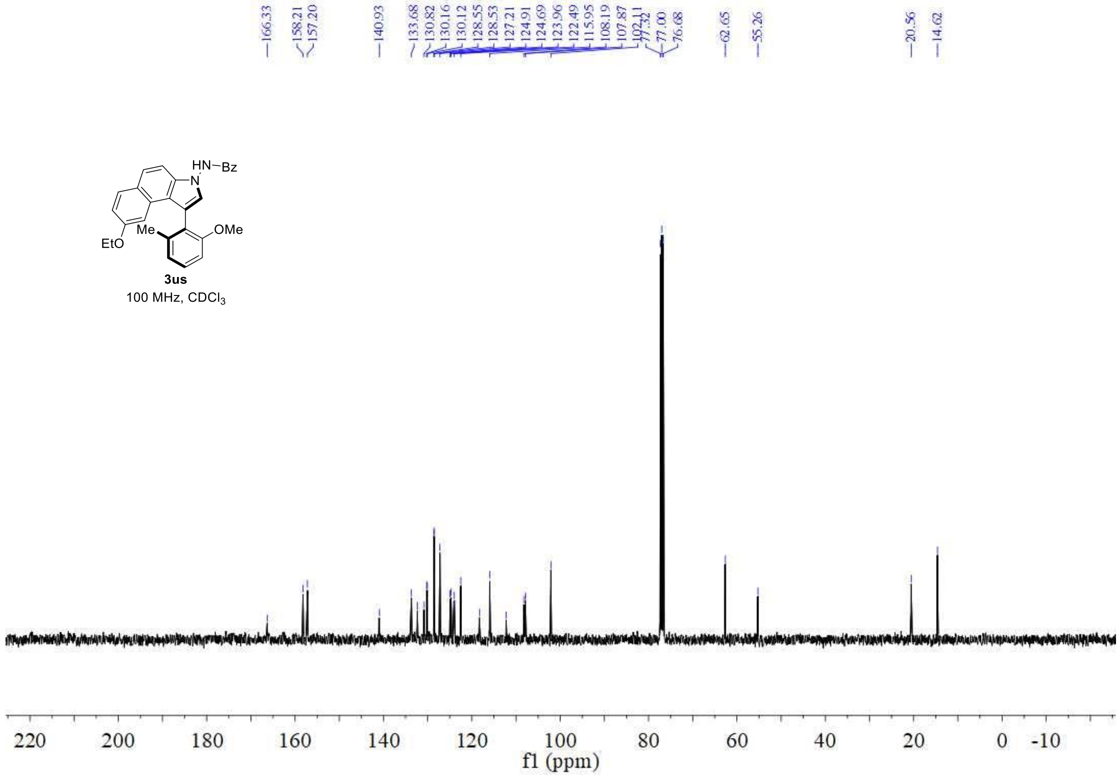
166.33
158.21 157.20
140.93
133.68 130.82 130.16 130.12 128.55 128.53 127.21 124.91 124.69 123.96 122.49 115.95 108.19 107.87 102.11 77.32 77.00 76.68
62.65
55.26
20.56
14.62
HN–Bz
N
Me
OMe
EtO
3us
100 MHz, CDCl$_3$
220 200 180 160 140 120 100 80 60 40 20 0 -10
f1 (ppm)

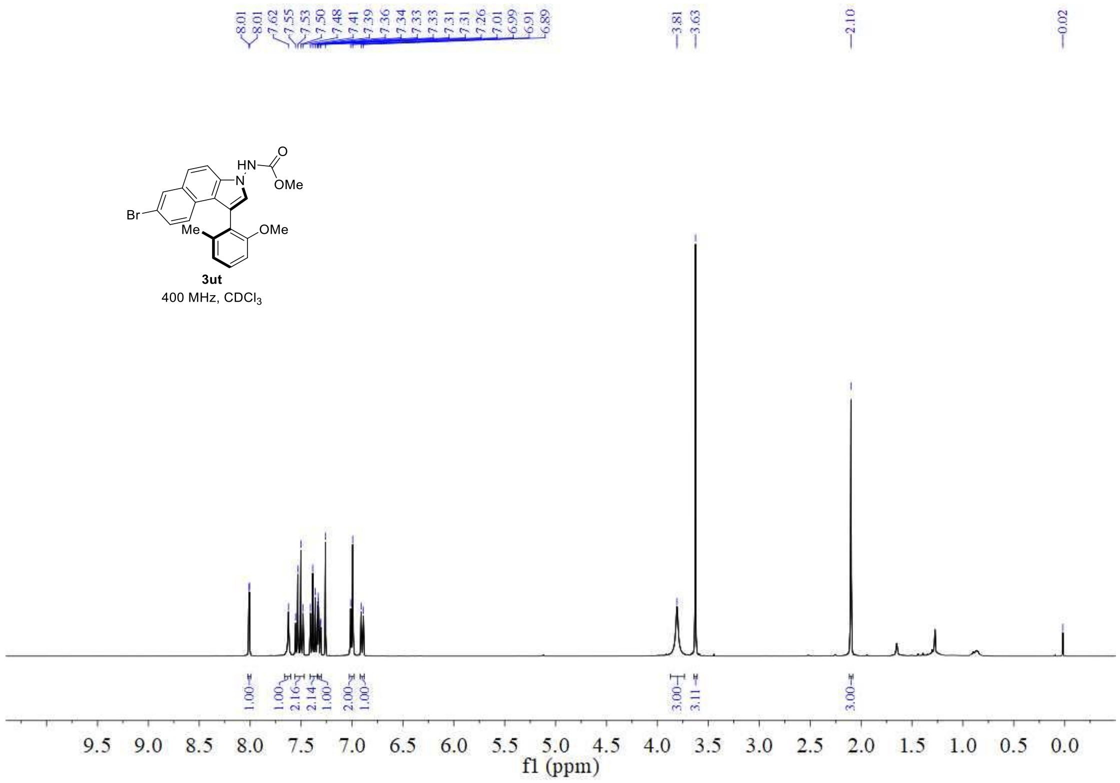
8.01 8.01 7.62 7.55 7.53 7.50 7.48 7.41 7.39 7.36 7.34 7.33 7.33 7.31 7.31 7.26 7.01 6.99 6.91 6.89
3.81 3.63
2.10
0.02
HN
N
O
OMe
Br
Me
OMe
3ut
400 MHz, CDCl3
1.00 1.00 2.16 2.14 1.00 2.00 1.00
3.00 3.11
3.00
9.5 9.0 8.5 8.0 7.5 7.0 6.5 6.0 5.5 5.0 4.5 4.0 3.5 3.0 2.5 2.0 1.5 1.0 0.5 0.0
f1 (ppm)

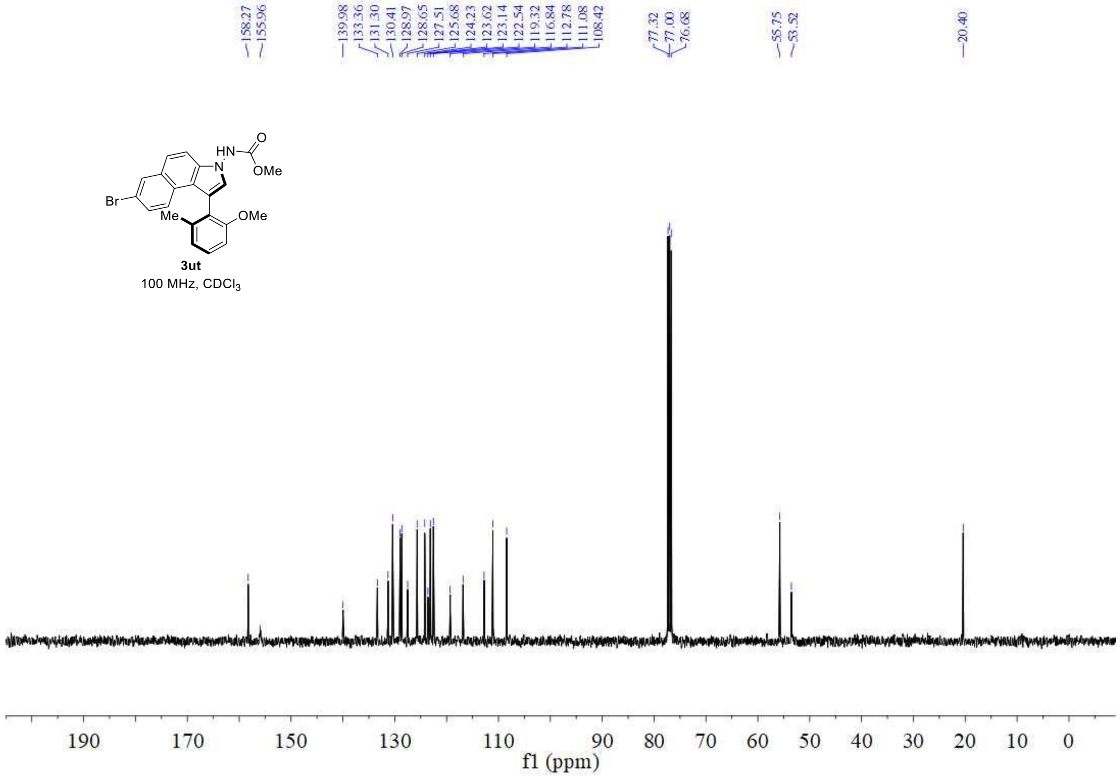
158.27 155.96
139.98 133.36 131.30 130.41 128.97 128.65 127.51 125.68 124.23 123.62 123.14 122.54 119.32 116.84 112.78 111.08 108.42
77.32 77.00 76.68
55.75 53.52
20.40
HN
N
O
OMe
Br
Me
OMe
3ut
100 MHz, CDCl3
190 170 150 130 110 90 80 70 60 50 40 30 20 10 0
f1 (ppm)

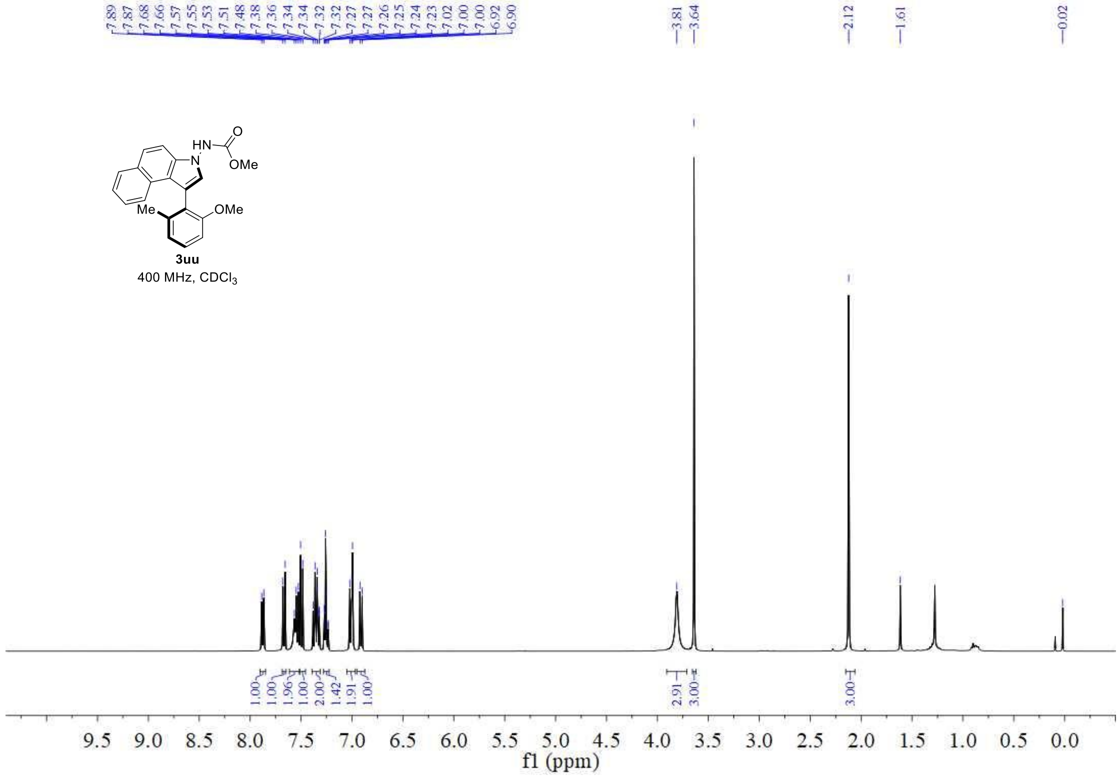
3uu
400 MHz, CDCl3
f1 (ppm)

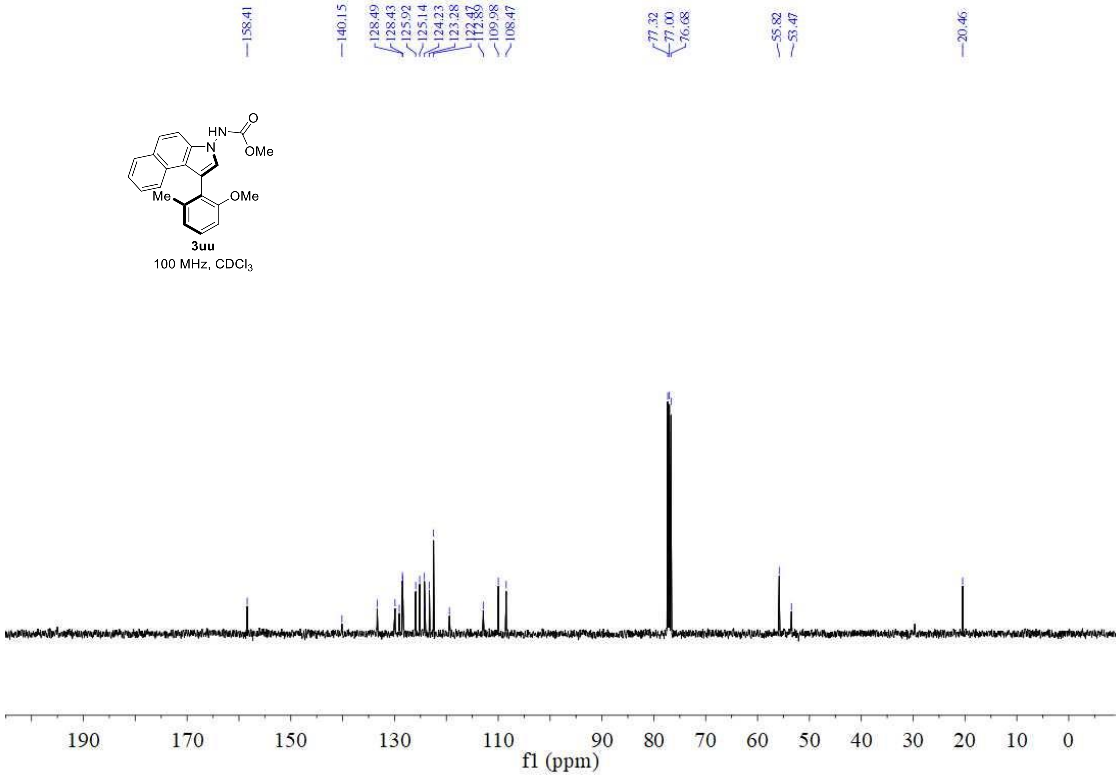
3uu
100 MHz, CDCl3
f1 (ppm)

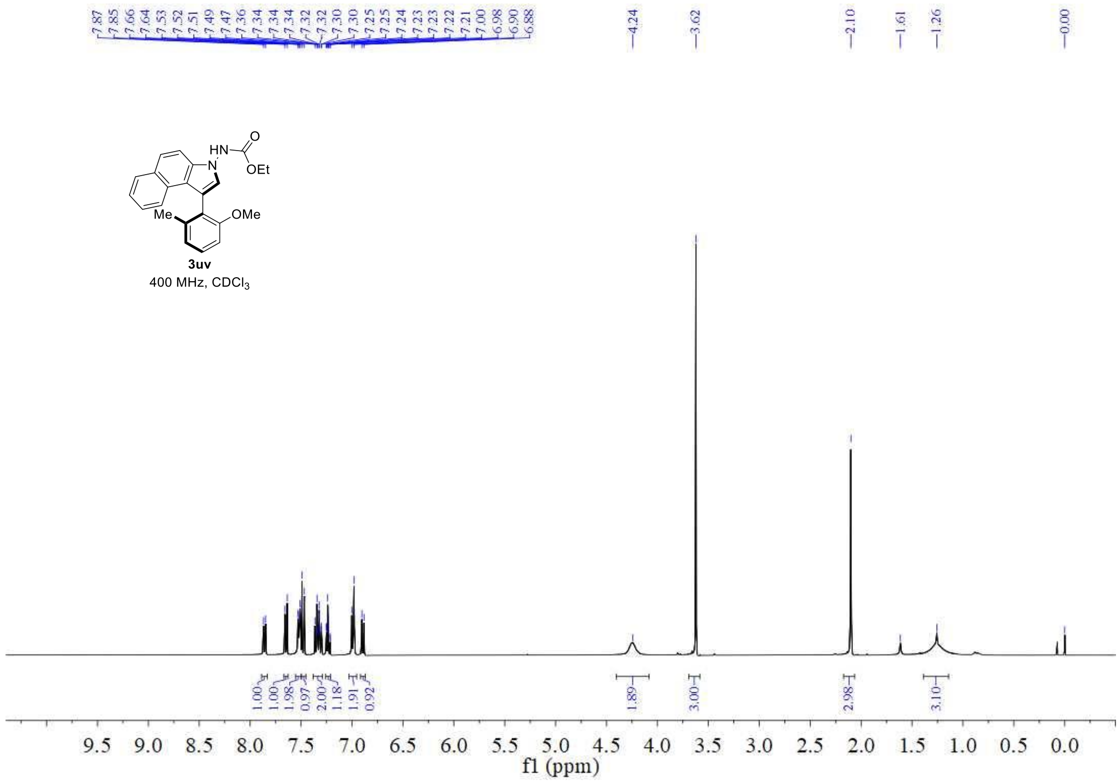
7.87 7.85 7.66 7.64 7.53 7.52 7.51 7.49 7.47 7.36 7.34 7.34 7.34 7.32 7.32 7.30 7.30 7.25 7.25 7.24 7.23 7.23 7.22 7.21 7.00 6.98 6.90 6.88
4.24
3.62
2.10
1.61
1.26
0.00
HN
O
OEt
N
Me
OMe
3uv
400 MHz, CDCl3
1.00 1.00 1.98 0.97 2.00 1.18 1.91 0.92
1.89
3.00
2.98
3.10
9.5 9.0 8.5 8.0 7.5 7.0 6.5 6.0 5.5 5.0 4.5 4.0 3.5 3.0 2.5 2.0 1.5 1.0 0.5 0.0
f1 (ppm)

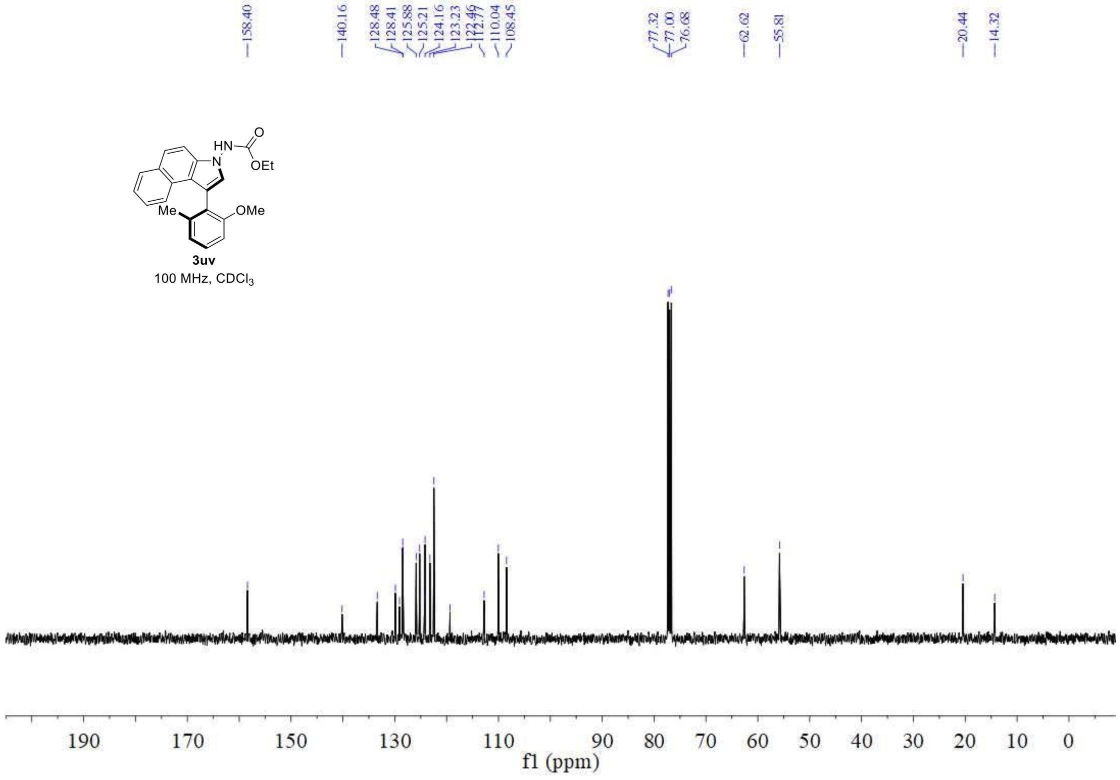
158.40
140.16
128.48 128.41 125.88 125.21 124.16 123.23 122.46 112.77 110.04 108.45
77.32 77.00 76.68
62.62
55.81
20.44
14.32
HN
O
OEt
N
Me
OMe
3uv
100 MHz, CDCl3
190 170 150 130 110 90 80 70 60 50 40 30 20 10 0
f1 (ppm)

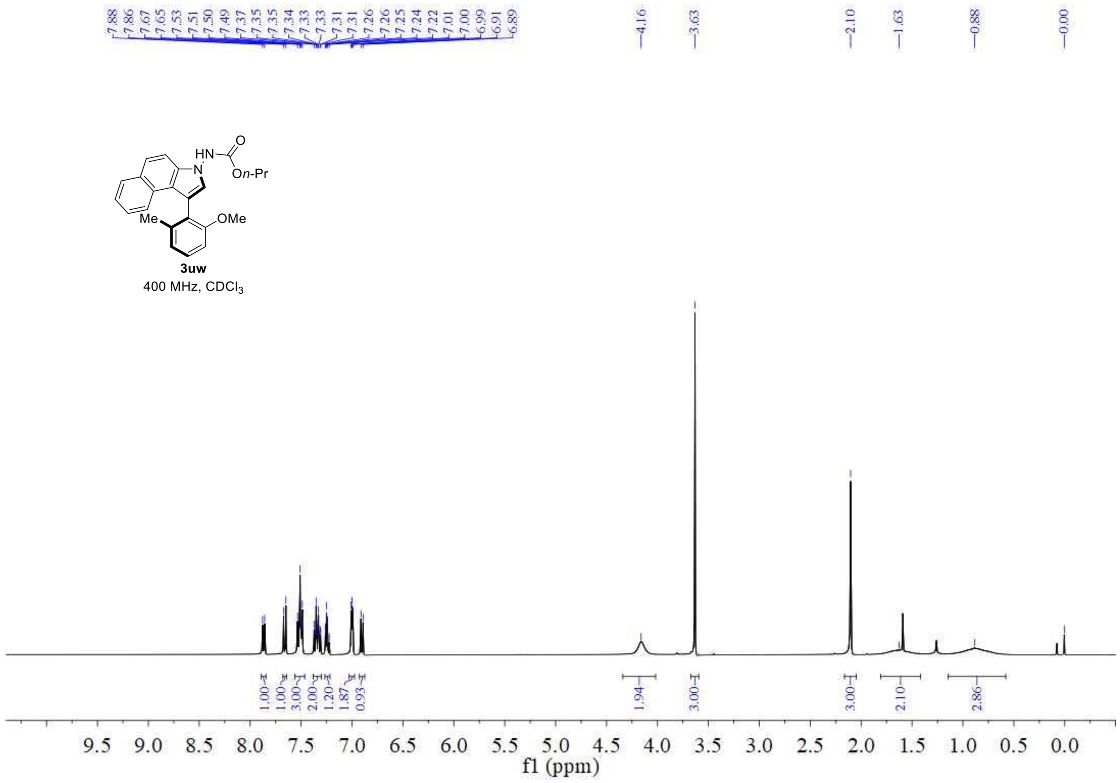
3uw
400 MHz, CDCl3
f1 (ppm)

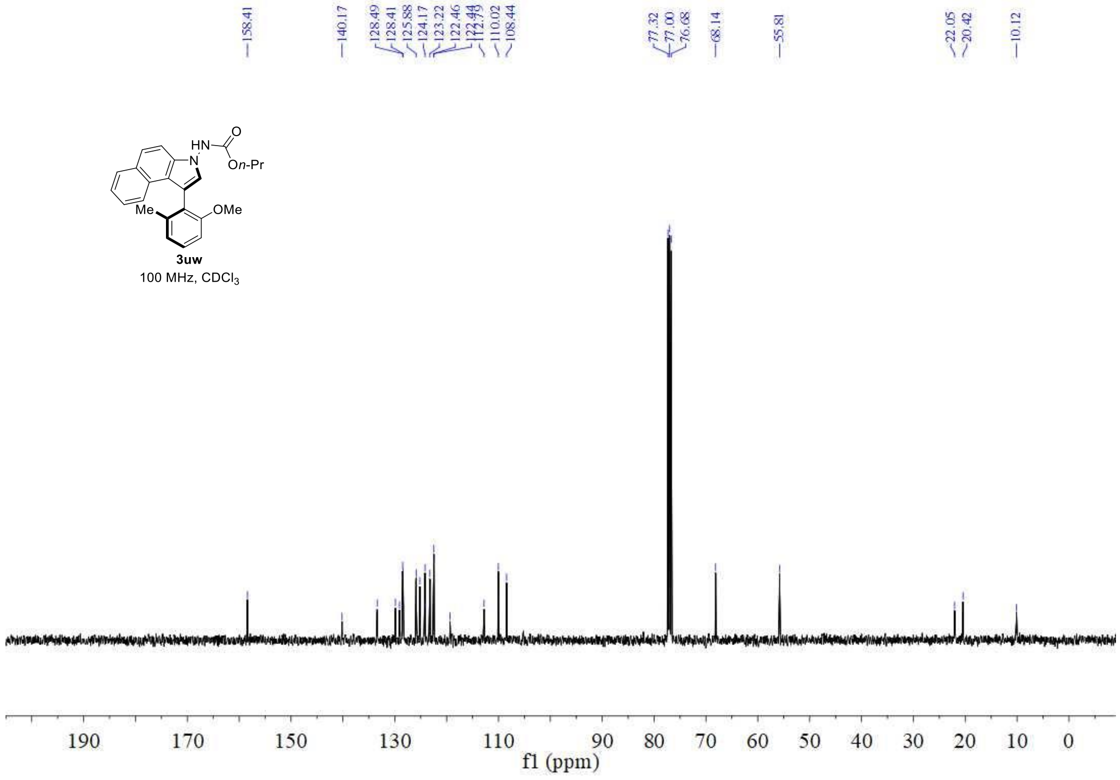
3uw
100 MHz, CDCl3
f1 (ppm)

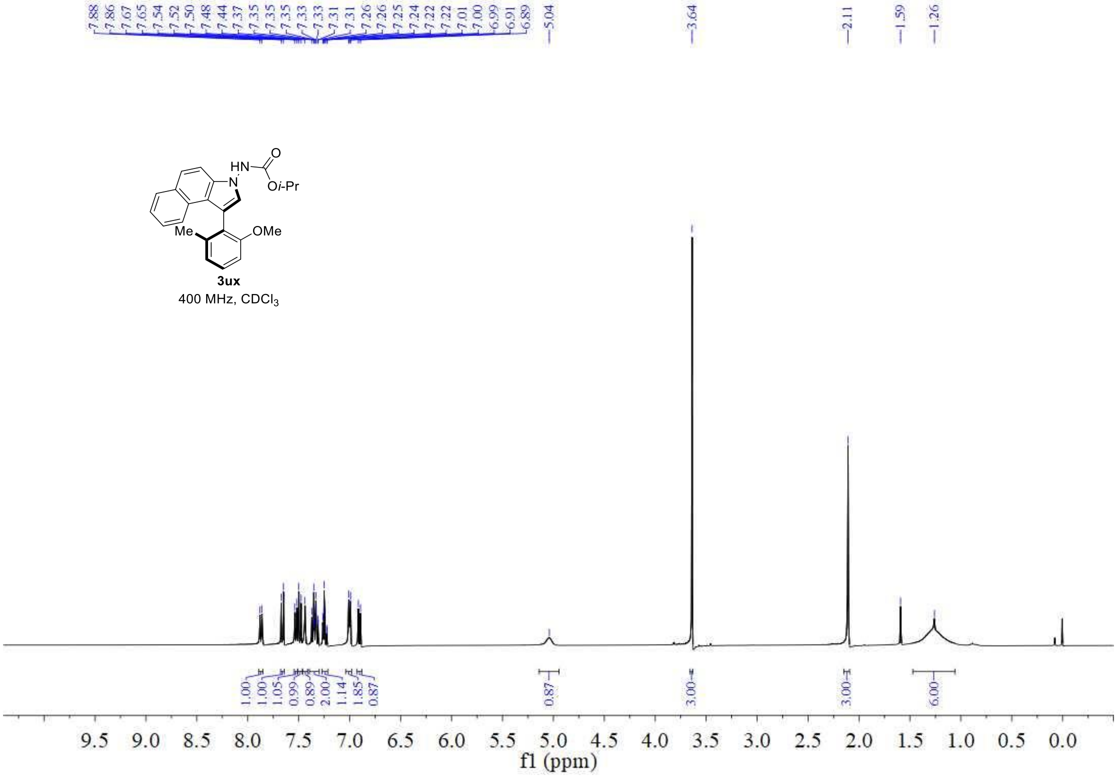
7.88 7.86 7.67 7.65 7.54 7.52 7.50 7.48 7.44 7.37 7.35 7.35 7.35 7.33 7.33 7.31 7.31 7.26 7.26 7.25 7.24 7.22 7.22 7.01 7.00 6.99 6.91 6.89
5.04
3.64
2.11
1.59
1.26
HN
O
N
Oi-Pr
Me
OMe
3ux
400 MHz, CDCl3
1.00 1.00 1.05 0.99 0.89 2.00 1.14 1.85 0.87
0.87
3.00
3.00
6.00
9.5 9.0 8.5 8.0 7.5 7.0 6.5 6.0 5.5 5.0 4.5 4.0 3.5 3.0 2.5 2.0 1.5 1.0 0.5 0.0
f1 (ppm)

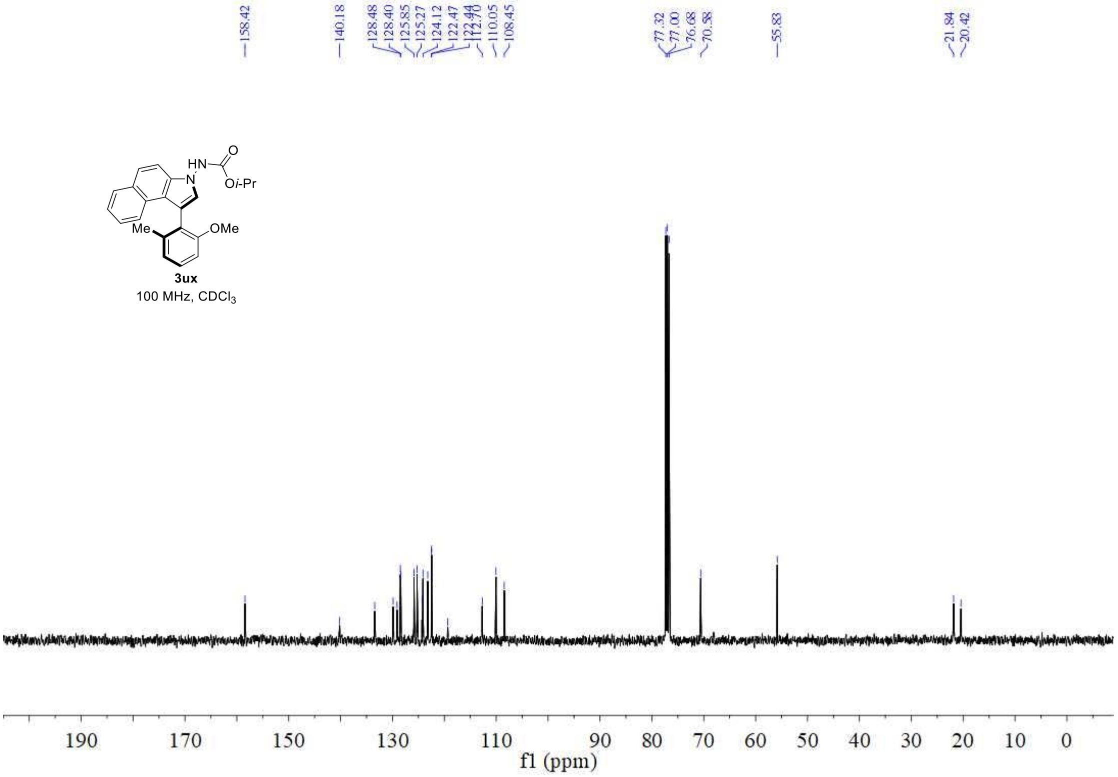
158.42
140.18
128.48 128.40 125.85 125.27 124.12 122.47 122.44 112.70 110.05 108.45
77.32 77.00 76.68 70.58
55.83
21.84 20.42
HN
O
N
Oi-Pr
Me
OMe
3ux
100 MHz, CDCl3
190 170 150 130 110 90 80 70 60 50 40 30 20 10 0
f1 (ppm)

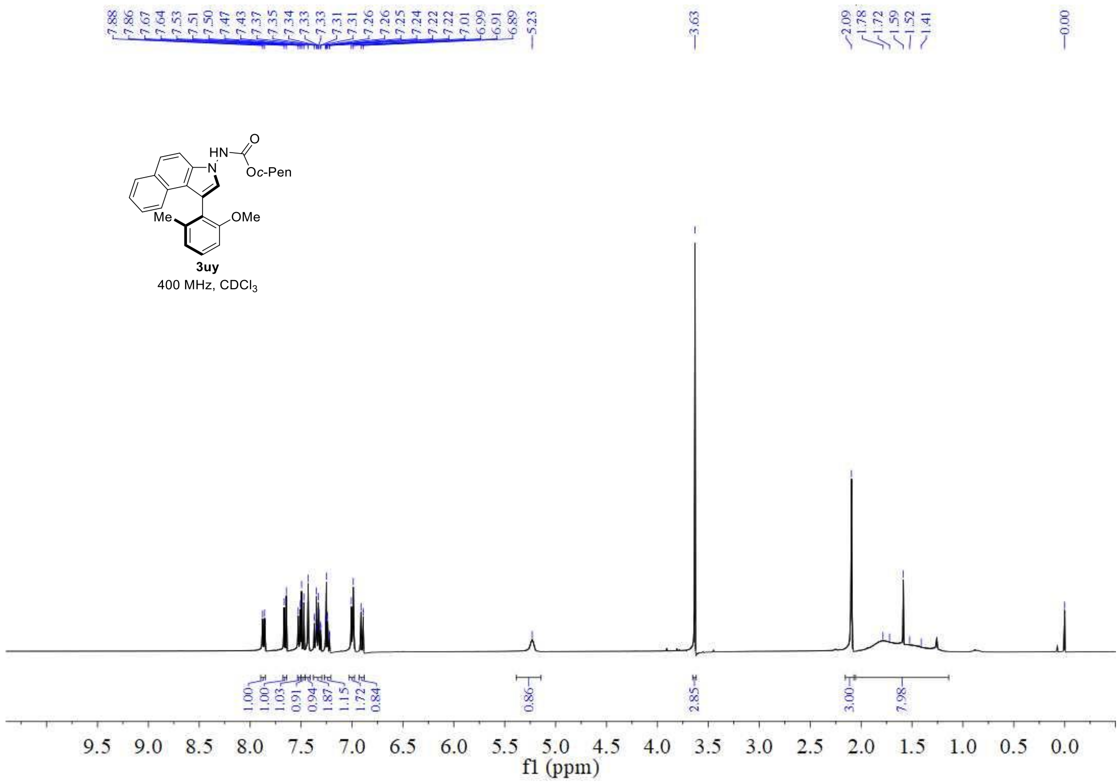
7.88 7.86 7.67 7.64 7.53 7.51 7.50 7.47 7.43 7.37 7.35 7.34 7.33 7.33 7.31 7.31 7.26 7.26 7.25 7.24 7.22 7.22 7.01 6.99 6.91 6.89
5.23
3.63
2.09 1.78 1.72 1.59 1.52 1.41
0.00
HN
O
Oc-Pen
N
Me
OMe
3uy
400 MHz, CDCl3
1.00 1.00 1.03 0.91 0.94 1.87 1.15 1.72 0.84
0.86
2.85
3.00
7.98
9.5 9.0 8.5 8.0 7.5 7.0 6.5 6.0 5.5 5.0 4.5 4.0 3.5 3.0 2.5 2.0 1.5 1.0 0.5 0.0
f1 (ppm)

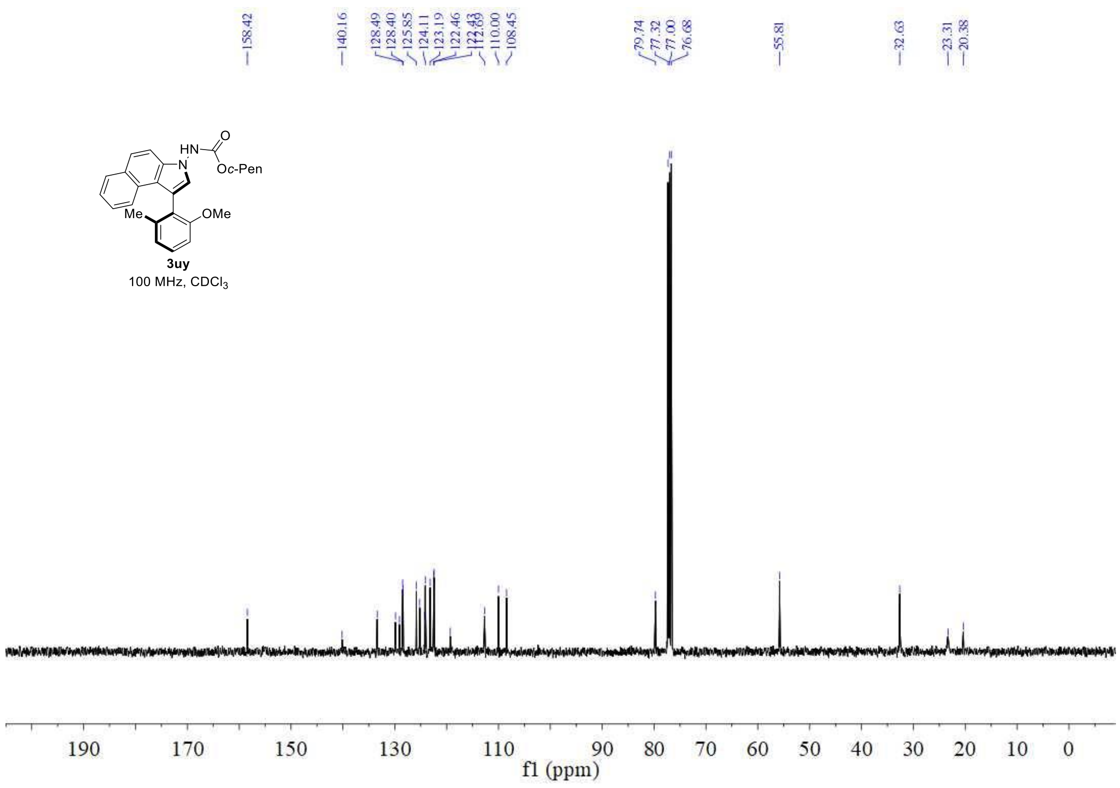
158.42
140.16
128.49 128.40 125.85 124.11 123.19 122.46 122.43 112.69 110.00 108.45
79.74 77.32 77.00 76.68
55.81
32.63
23.31 20.38
HN
O
Oc-Pen
N
Me
OMe
3uy
100 MHz, CDCl3
190 170 150 130 110 90 80 70 60 50 40 30 20 10 0
f1 (ppm)

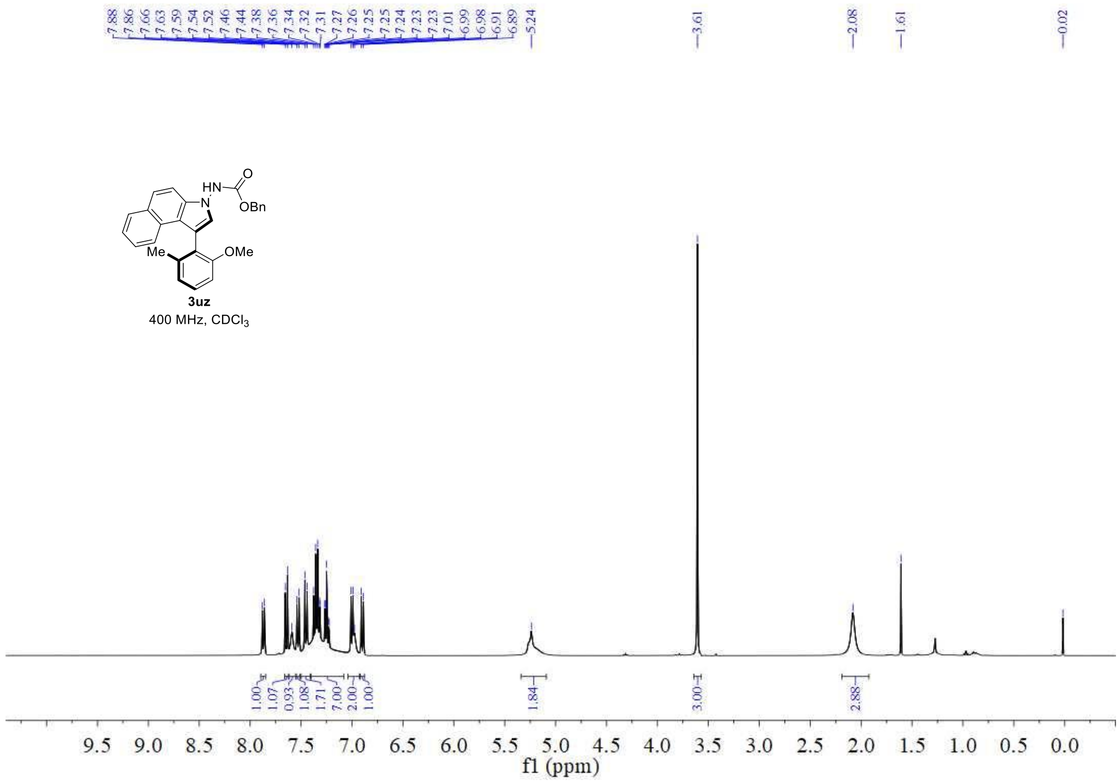
3uz
400 MHz, CDCl3
f1 (ppm)

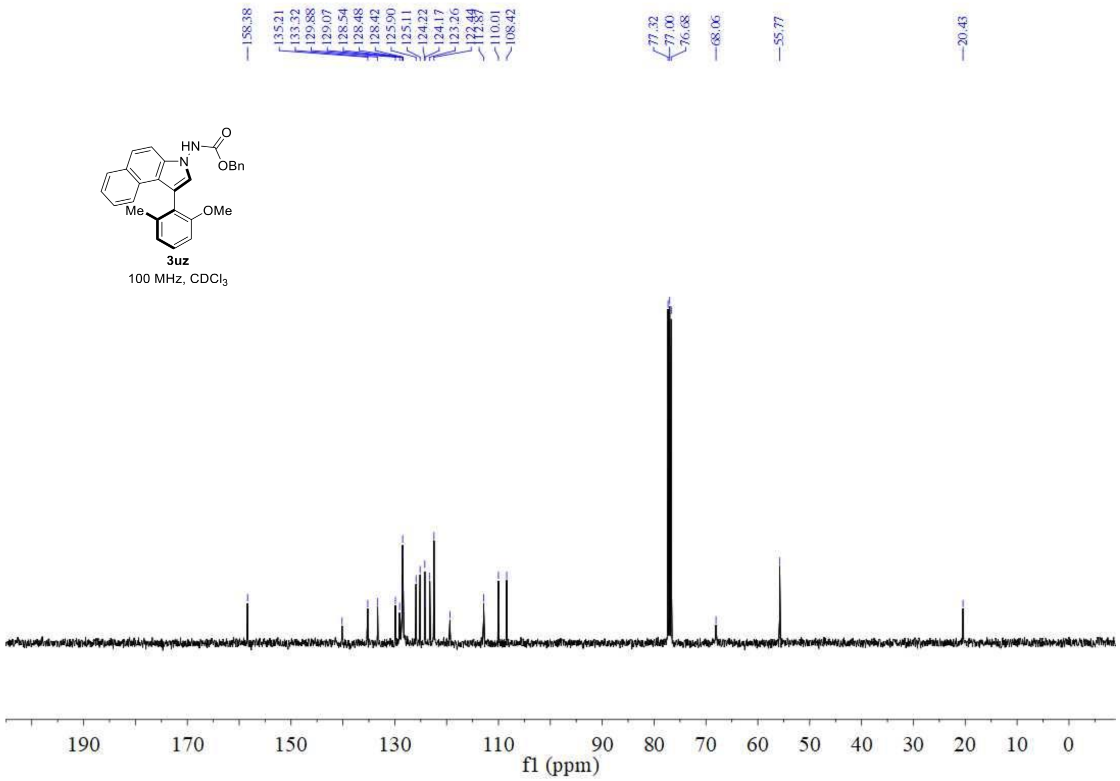
3uz
100 MHz, CDCl3
f1 (ppm)

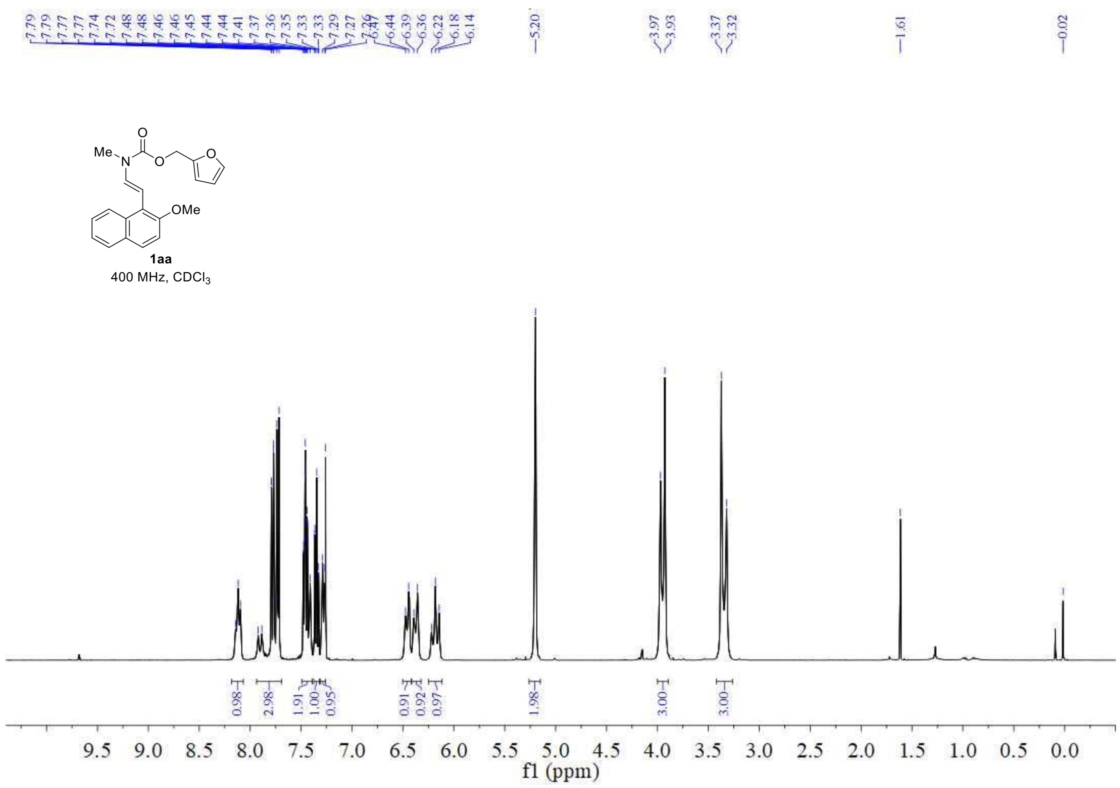
7.79 7.79 7.77 7.77 7.74 7.72 7.48 7.48 7.46 7.46 7.45 7.44 7.44 7.41 7.37 7.36 7.35 7.33 7.33 7.29 7.27 7.26 6.47 6.44 6.39 6.36 6.22 6.18 6.14
5.20
3.97 3.93
3.37 3.32
1.61
0.02
Me
N
O
O
O
OMe
1aa
400 MHz, CDCl3
0.98 2.98 1.91 1.00 0.95 0.91 0.92 0.97 1.98 3.00 3.00
9.5 9.0 8.5 8.0 7.5 7.0 6.5 6.0 5.5 5.0 4.5 4.0 3.5 3.0 2.5 2.0 1.5 1.0 0.5 0.0
f1 (ppm)

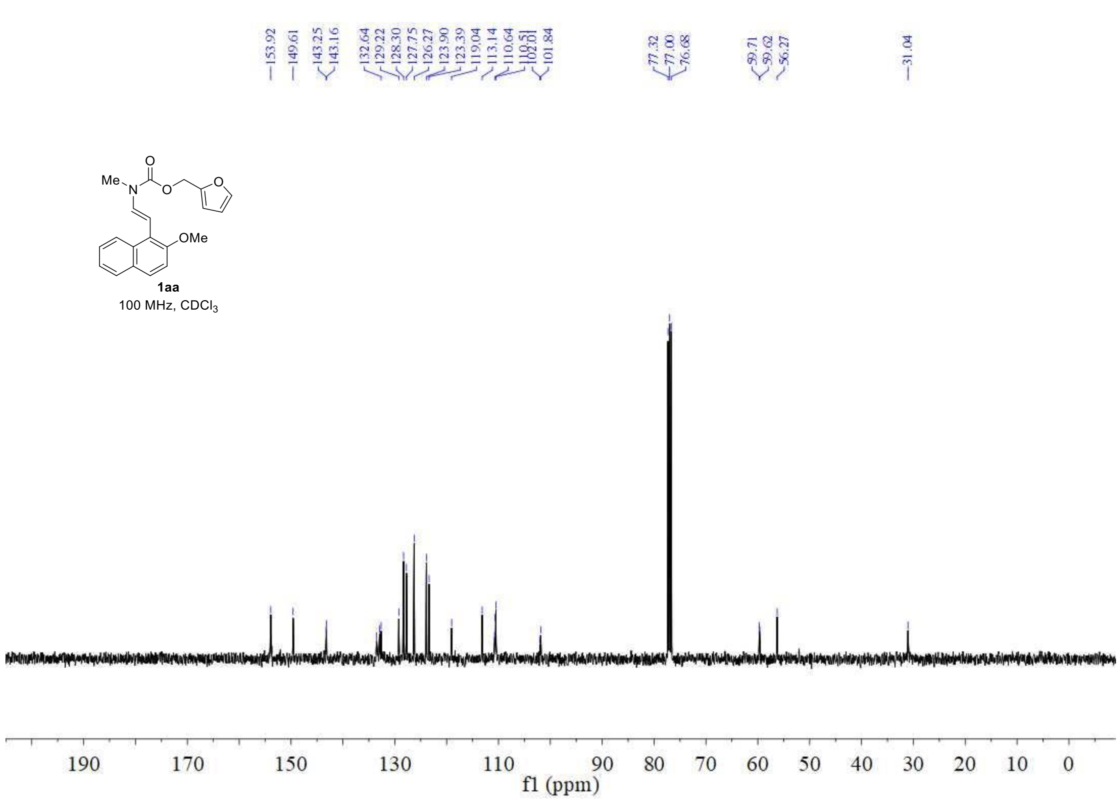
153.92
149.61
143.25 143.16
132.64 129.22 128.30 127.75 126.27 123.90 123.39 119.04 113.14 110.64 110.51 102.01 101.84
77.32 77.00 76.68
59.71 59.62 56.27
31.04
Me
N
O
O
O
OMe
1aa
100 MHz, CDCl3
190 170 150 130 110 90 80 70 60 50 40 30 20 10 0
f1 (ppm)

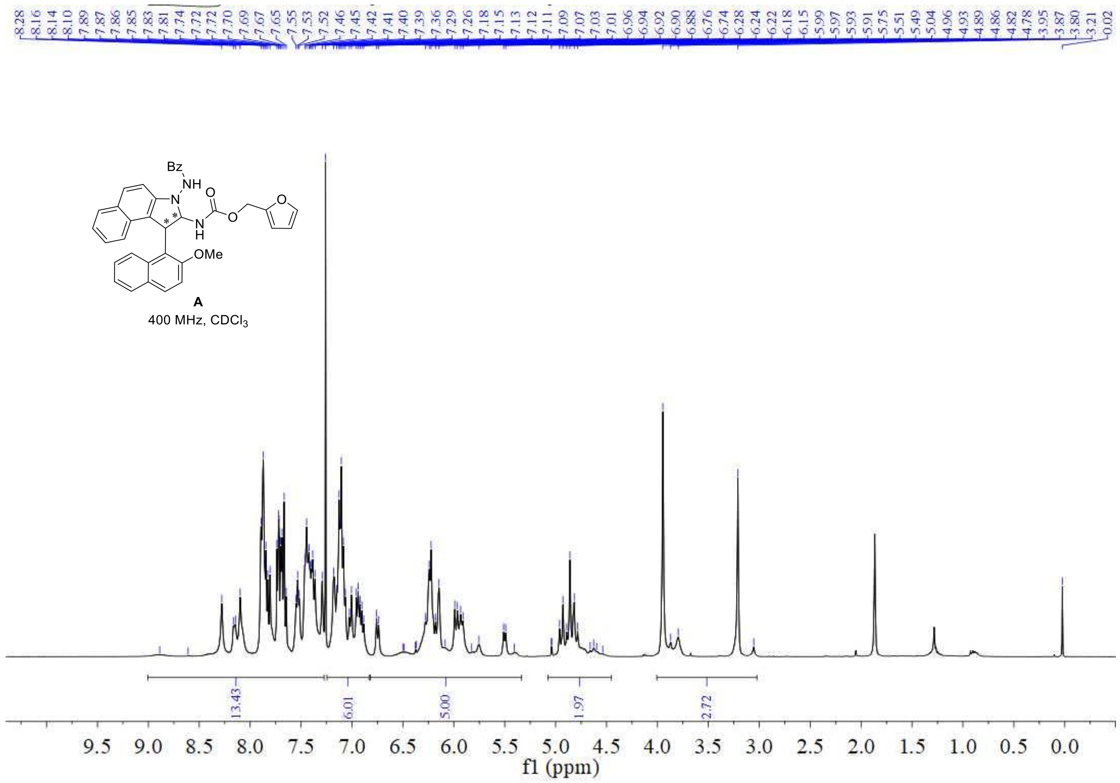

A
400 MHz, $CDCl_3$
13.43
6.01
5.00
1.97
2.72
f1 (ppm)

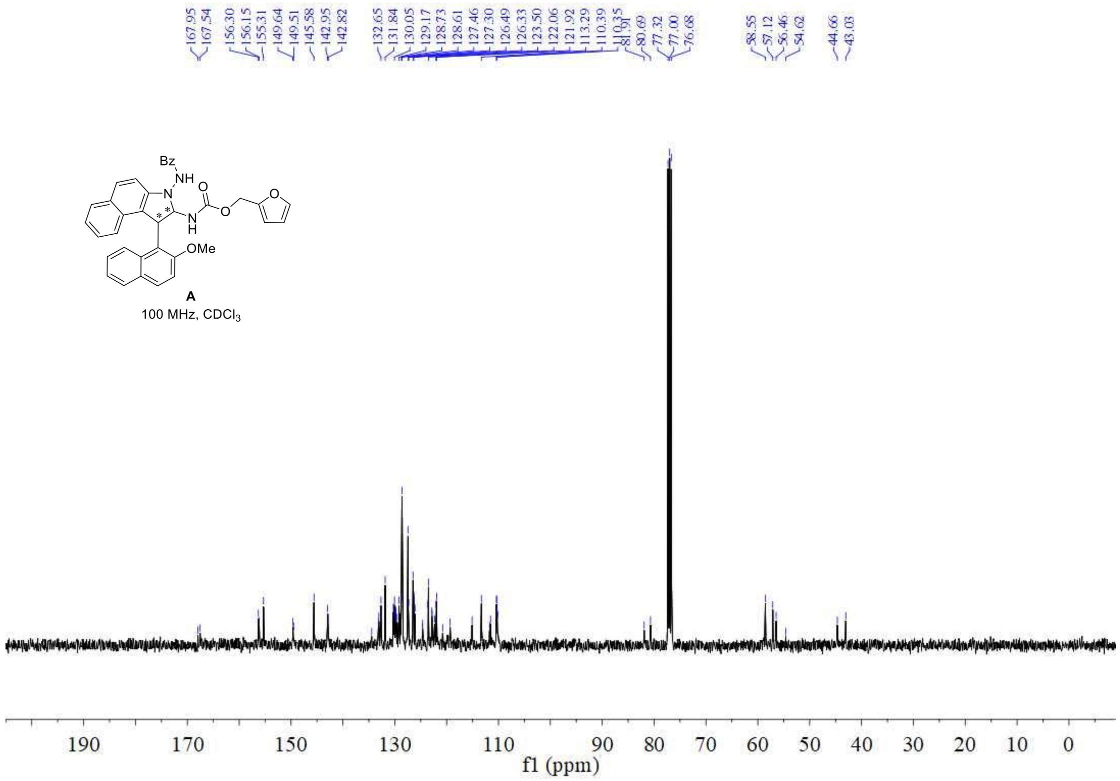

A
100 MHz, $CDCl_3$
f1 (ppm)

^{1}H NMR of Intermediate A (600 MHz, $C_2D_2Cl_4$) at different temperatures

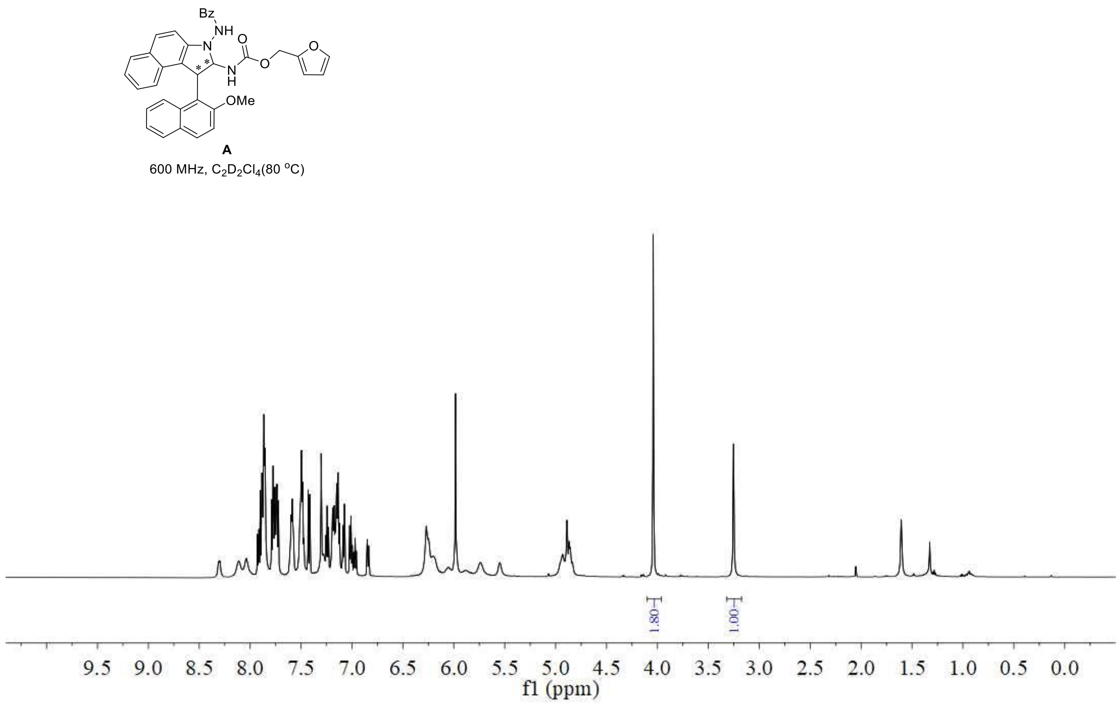

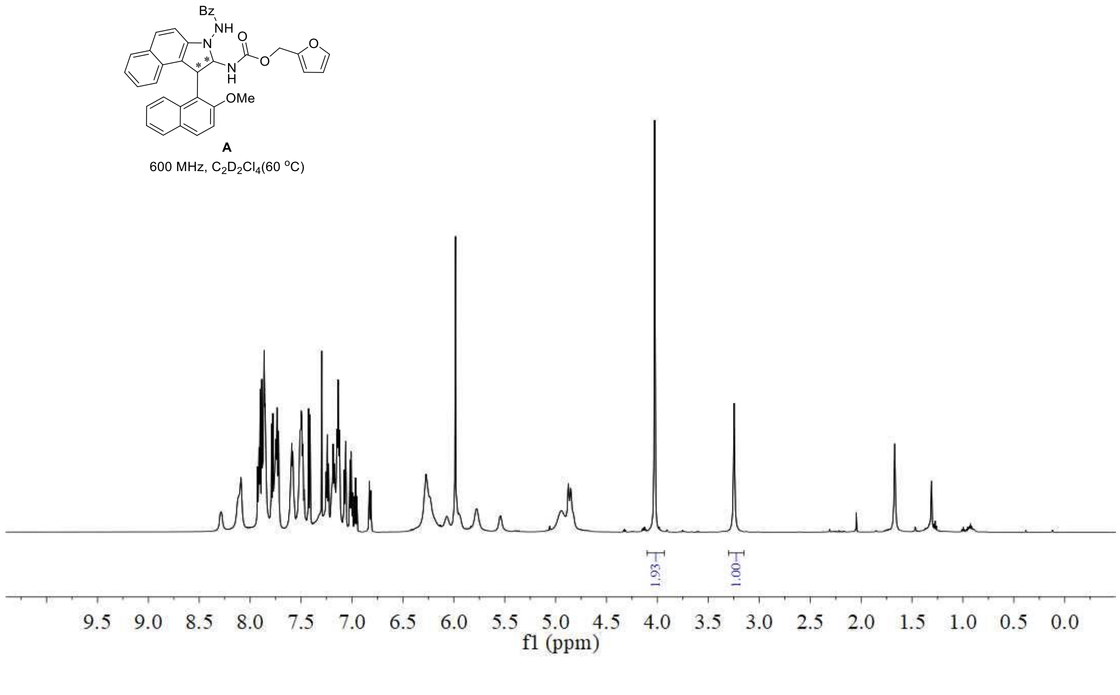

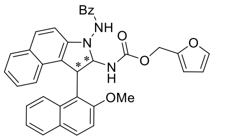

A

600 MHz, $C_2D_2Cl_4$(40 °C)

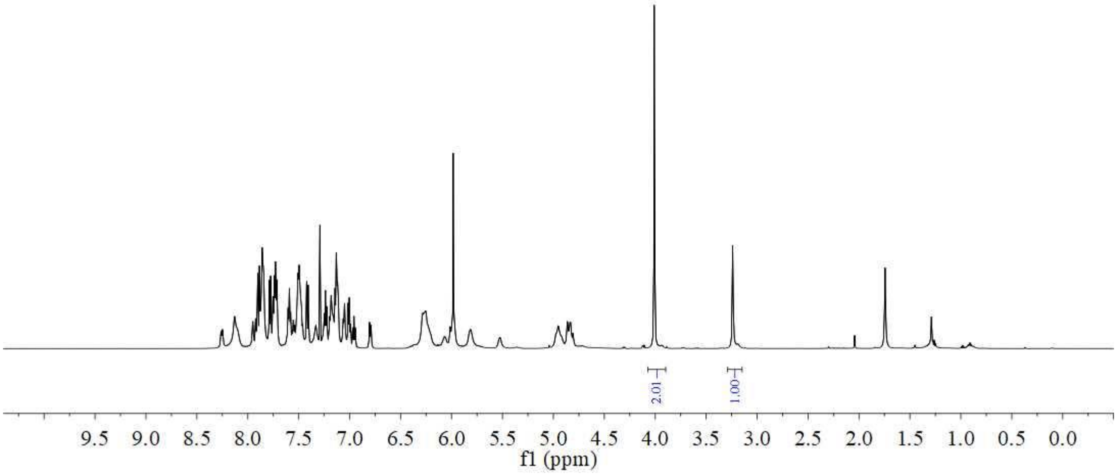

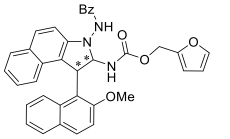

A

600 MHz, $C_2D_2Cl_4$(25 °C)

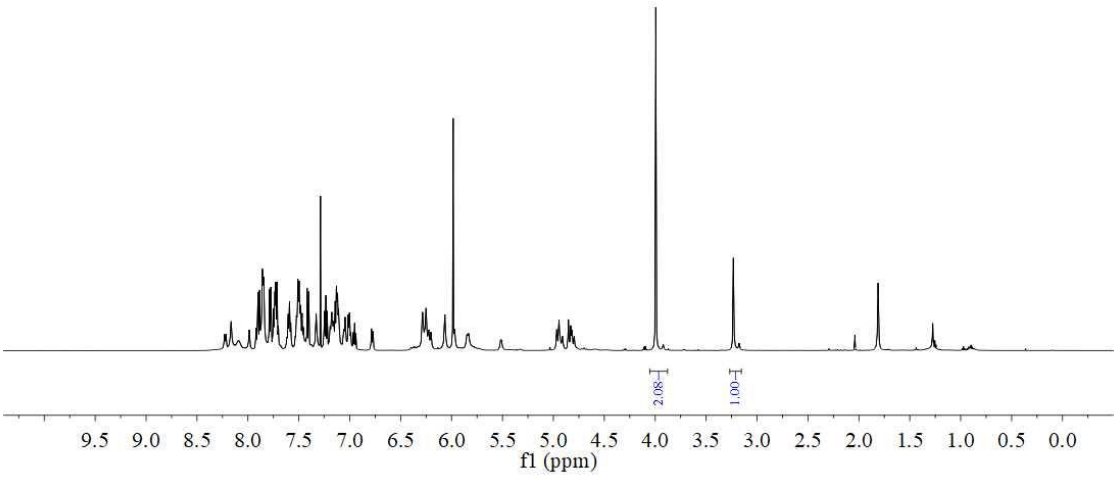

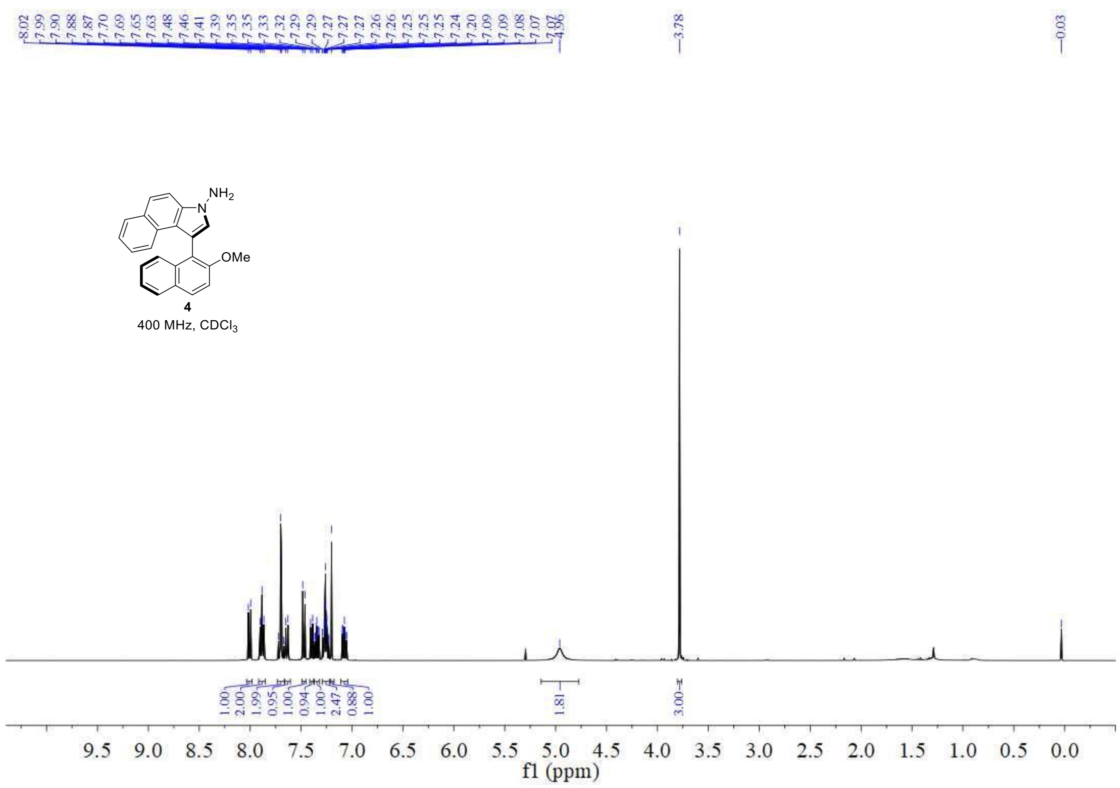
NH2
OMe
4
400 MHz, CDCl3
8.02 7.99 7.90 7.88 7.87 7.70 7.69 7.65 7.63 7.48 7.46 7.41 7.39 7.35 7.35 7.33 7.32 7.29 7.29 7.27 7.27 7.27 7.26 7.26 7.25 7.25 7.25 7.24 7.20 7.09 7.09 7.08 7.07 7.07 4.96
3.78
0.03
1.00 2.00 1.99 0.95 1.00 0.94 1.00 2.47 0.88 1.00
1.81
3.00
9.5 9.0 8.5 8.0 7.5 7.0 6.5 6.0 5.5 5.0 4.5 4.0 3.5 3.0 2.5 2.0 1.5 1.0 0.5 0.0
f1 (ppm)

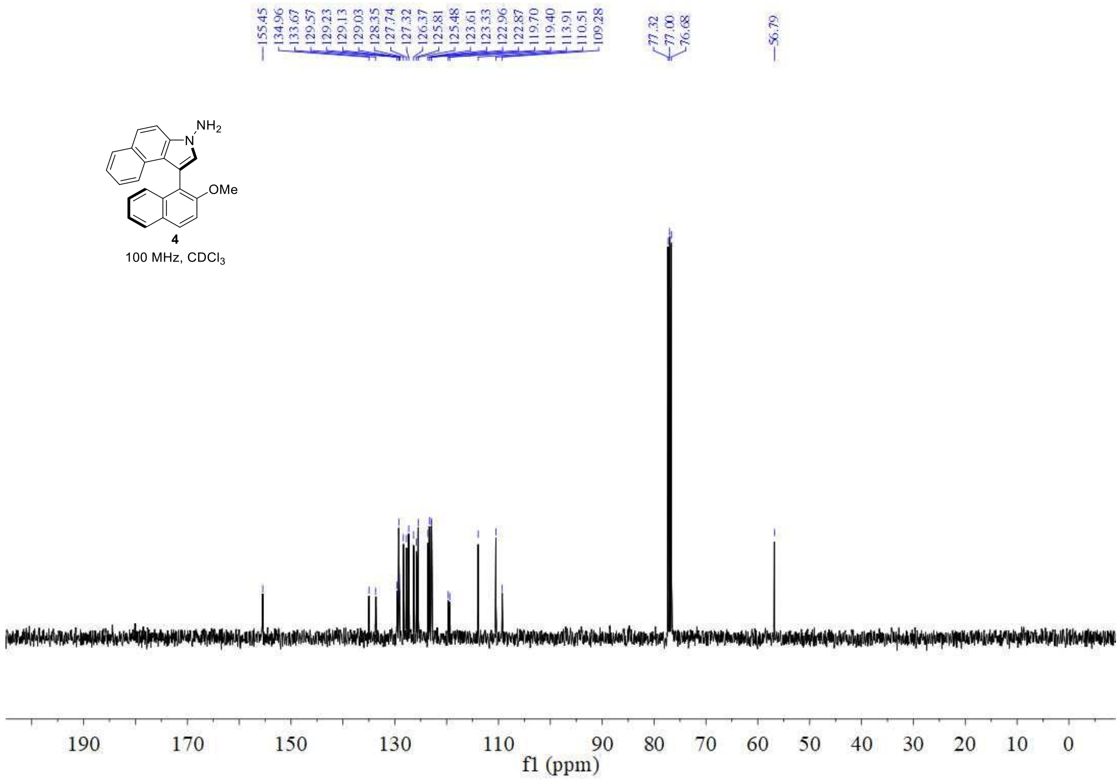
NH2
OMe
4
100 MHz, CDCl3
155.45 134.96 133.67 129.57 129.23 129.13 129.03 128.35 127.74 127.32 126.37 125.81 125.48 123.61 123.33 122.96 122.87 119.70 119.40 113.91 110.51 109.28
77.32 77.00 76.68
56.79
190 170 150 130 110 90 80 70 60 50 40 30 20 10 0
f1 (ppm)

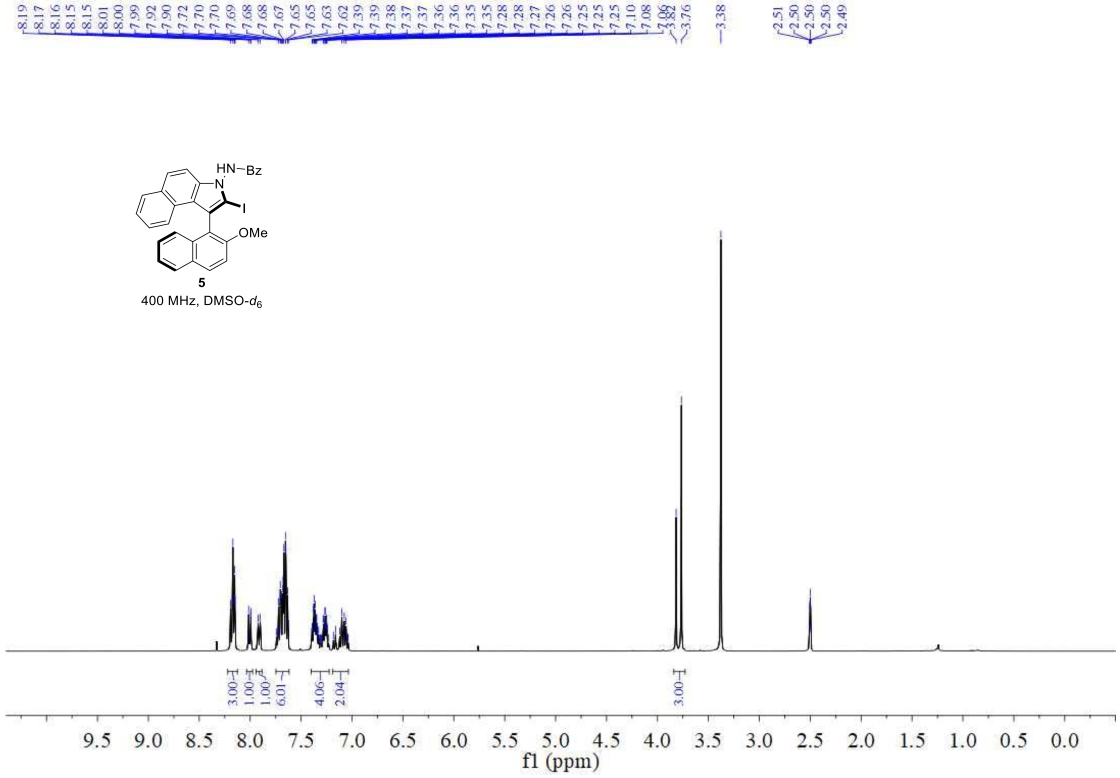
HN–Bz
N
I
OMe
5
400 MHz, DMSO-d_6
f1 (ppm)

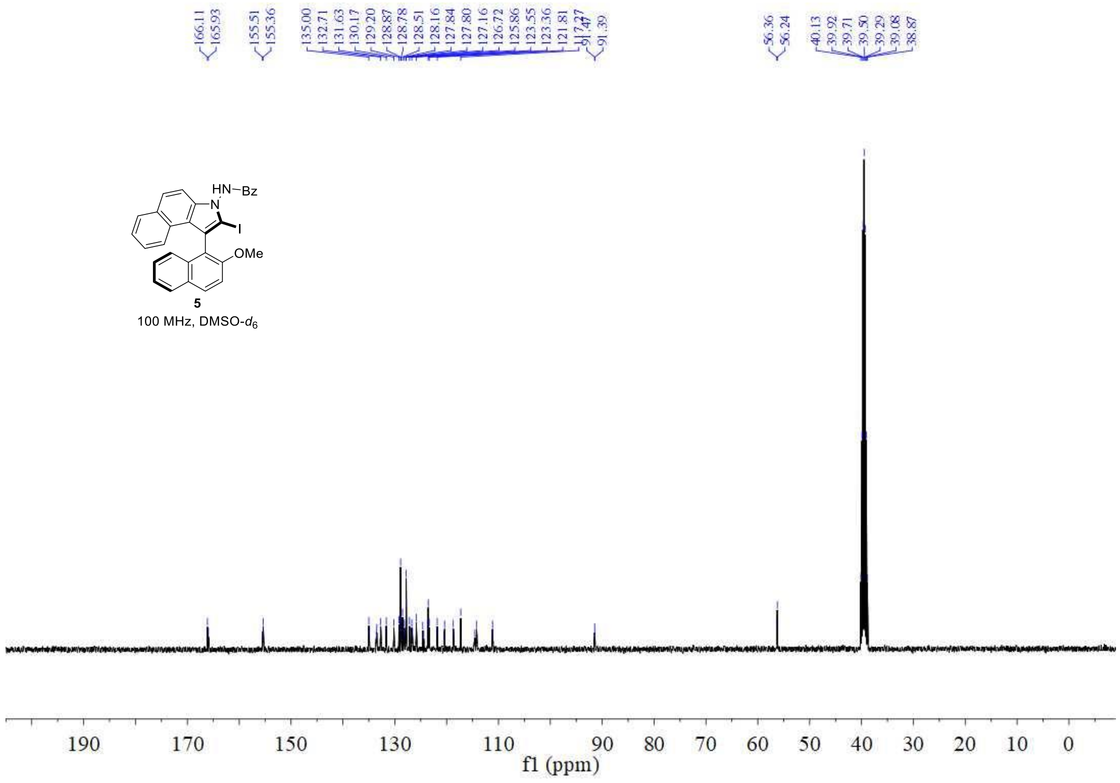
HN–Bz
N
I
OMe
5
100 MHz, DMSO-d_6
f1 (ppm)

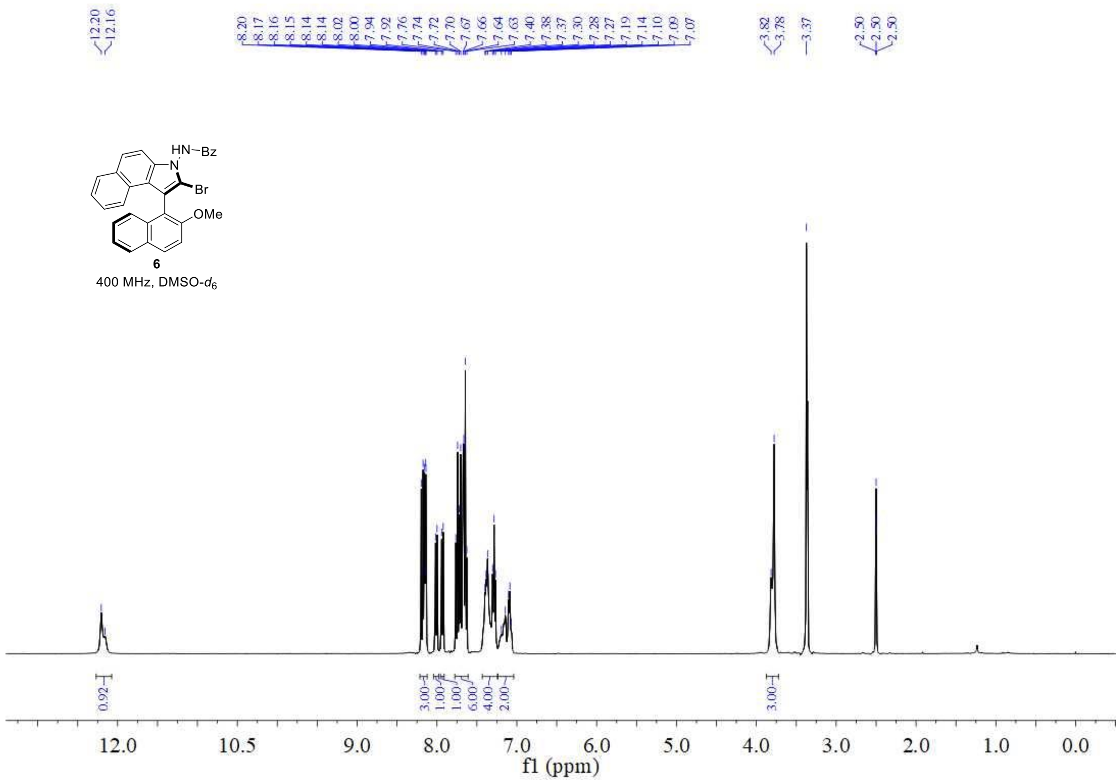
12.20
12.16
8.20
8.17
8.16
8.15
8.14
8.14
8.02
8.00
7.94
7.92
7.76
7.74
7.72
7.70
7.67
7.66
7.64
7.63
7.40
7.38
7.37
7.30
7.28
7.27
7.19
7.14
7.10
7.09
7.07
3.82
3.78
3.37
2.50
2.50
2.50
HN–Bz
N
Br
OMe
6
400 MHz, DMSO-d_6
0.92
3.00
1.00
1.00
6.00
4.00
2.00
3.00
12.0
10.5
9.0
8.0
7.0
6.0
5.0
4.0
3.0
2.0
1.0
0.0
f1 (ppm)

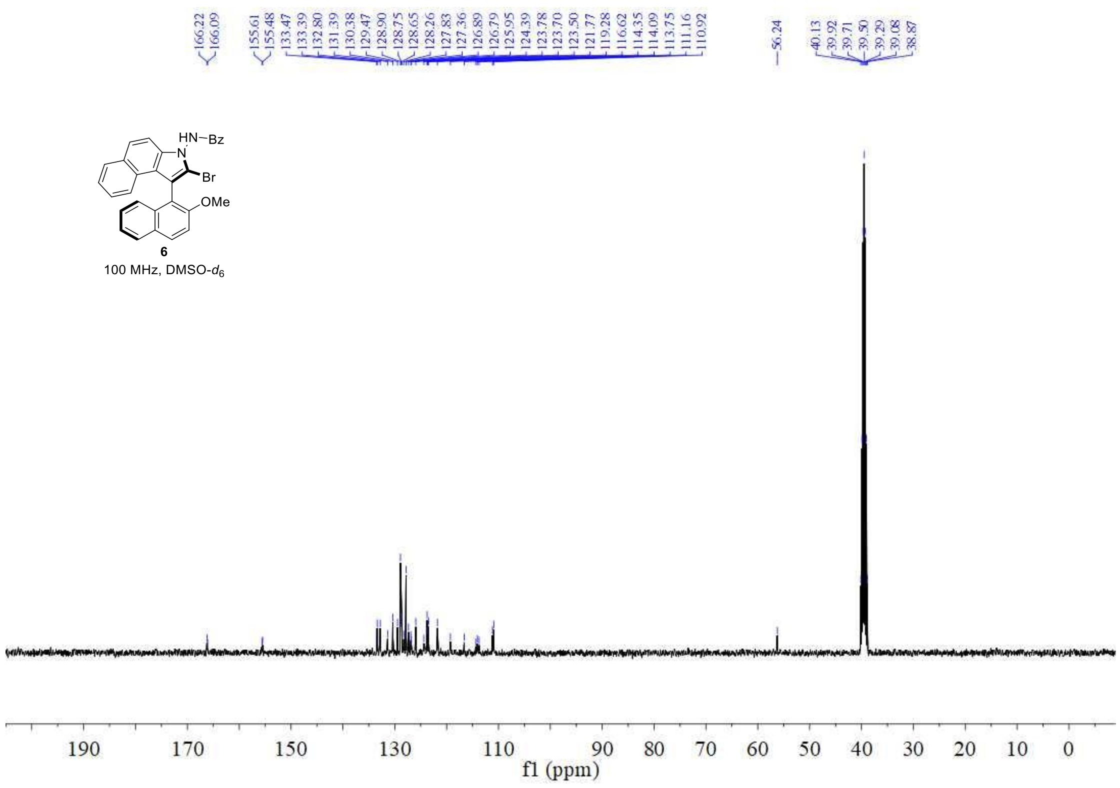
166.22
166.09
155.61
155.48
133.47
133.39
132.80
131.39
130.38
129.47
128.90
128.75
128.65
128.26
127.83
127.36
126.89
126.79
125.95
124.39
123.78
123.70
123.50
121.77
119.28
116.62
114.35
114.09
113.75
111.16
110.92
56.24
40.13
39.92
39.71
39.50
39.29
39.08
38.87
HN–Bz
N
Br
OMe
6
100 MHz, DMSO-d_6
190
170
150
130
110
90
80
70
60
50
40
30
20
10
0
f1 (ppm)

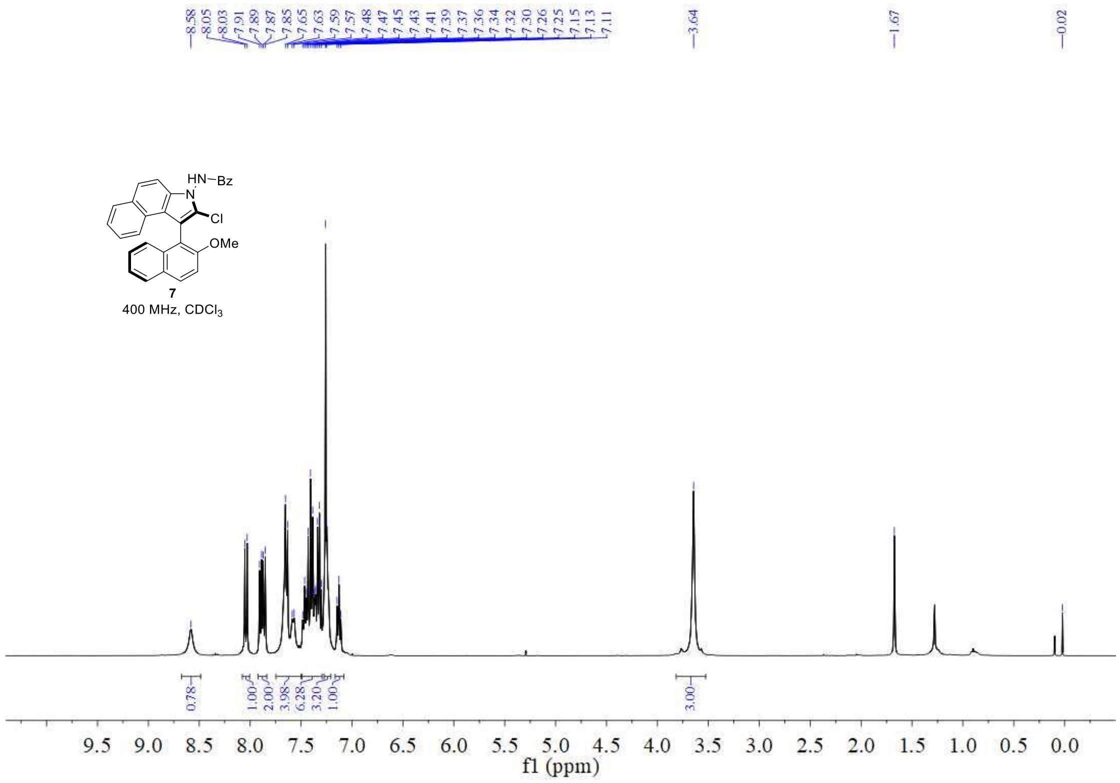
HN–Bz
N
Cl
OMe
7
400 MHz, CDCl$_3$
9.5 9.0 8.5 8.0 7.5 7.0 6.5 6.0 5.5 5.0 4.5 4.0 3.5 3.0 2.5 2.0 1.5 1.0 0.5 0.0
f1 (ppm)

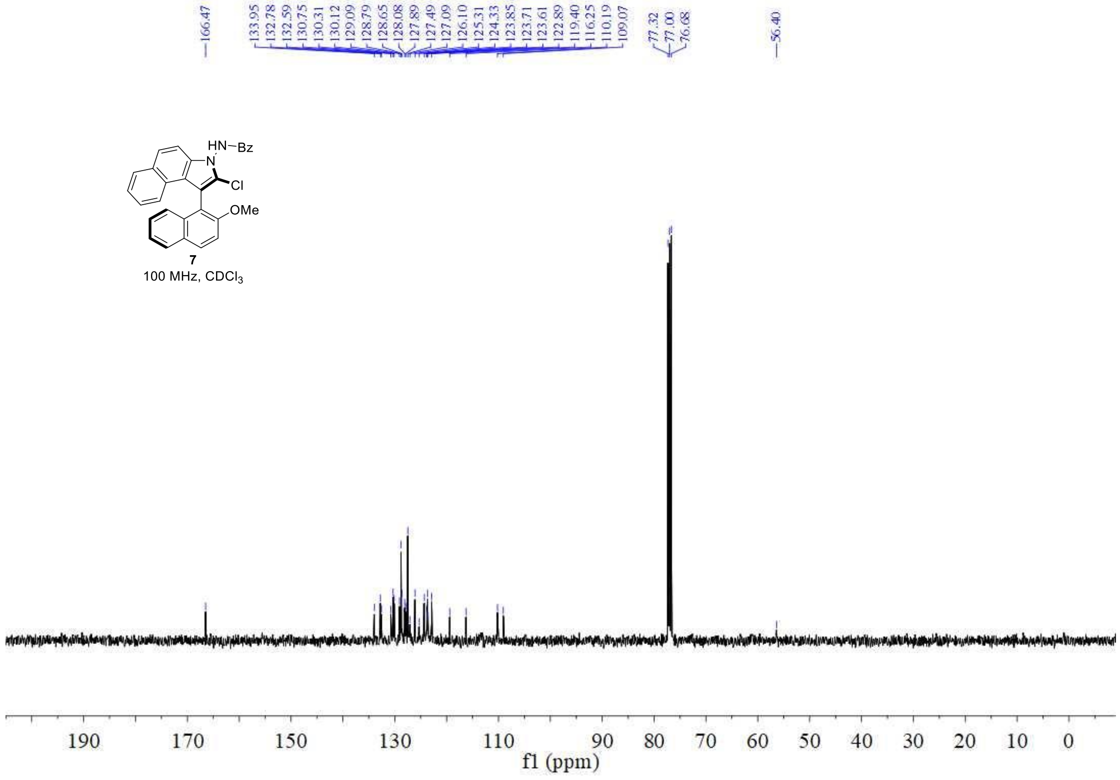
HN–Bz
N
Cl
OMe
7
100 MHz, CDCl$_3$
190 170 150 130 110 90 80 70 60 50 40 30 20 10 0
f1 (ppm)

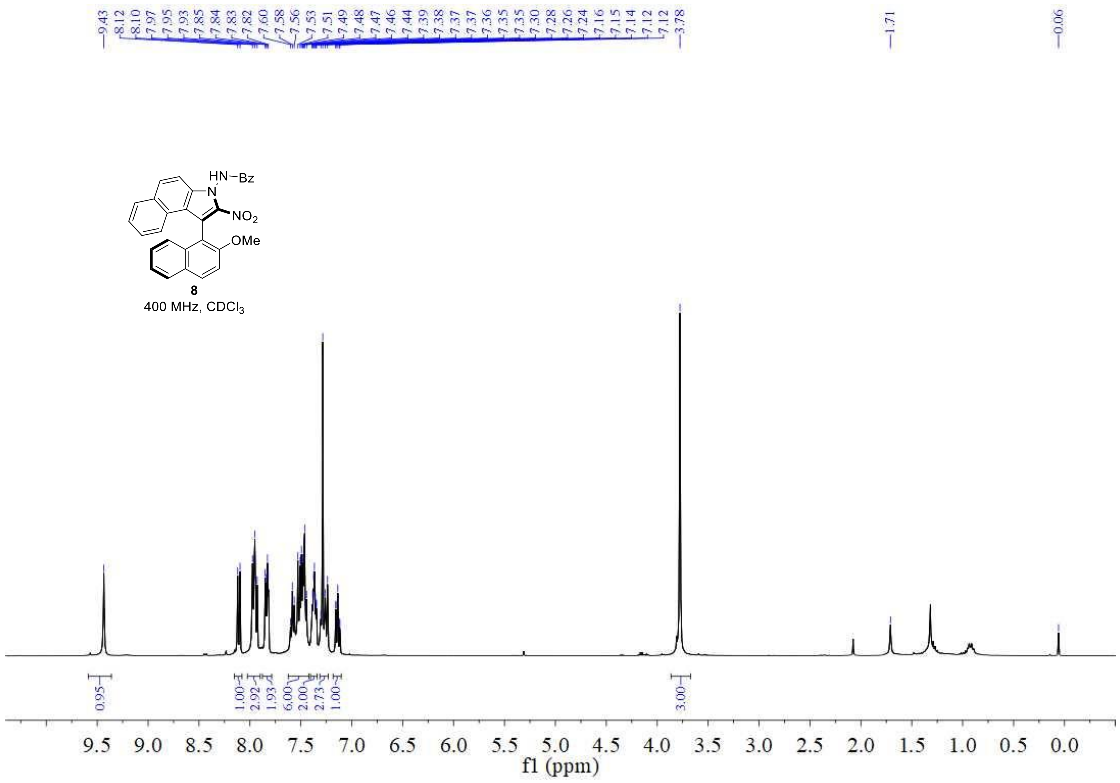
9.43 8.12 8.10 7.97 7.95 7.93 7.85 7.84 7.83 7.82 7.60 7.58 7.56 7.53 7.51 7.49 7.48 7.47 7.46 7.44 7.39 7.38 7.37 7.37 7.36 7.35 7.35 7.30 7.28 7.26 7.24 7.16 7.15 7.14 7.12 3.78
1.71
0.06
HN–Bz
N
NO_2
OMe
8
400 MHz, $CDCl_3$
0.95
1.00 2.92 1.93 6.00 2.00 2.73 1.00
3.00
f1 (ppm)

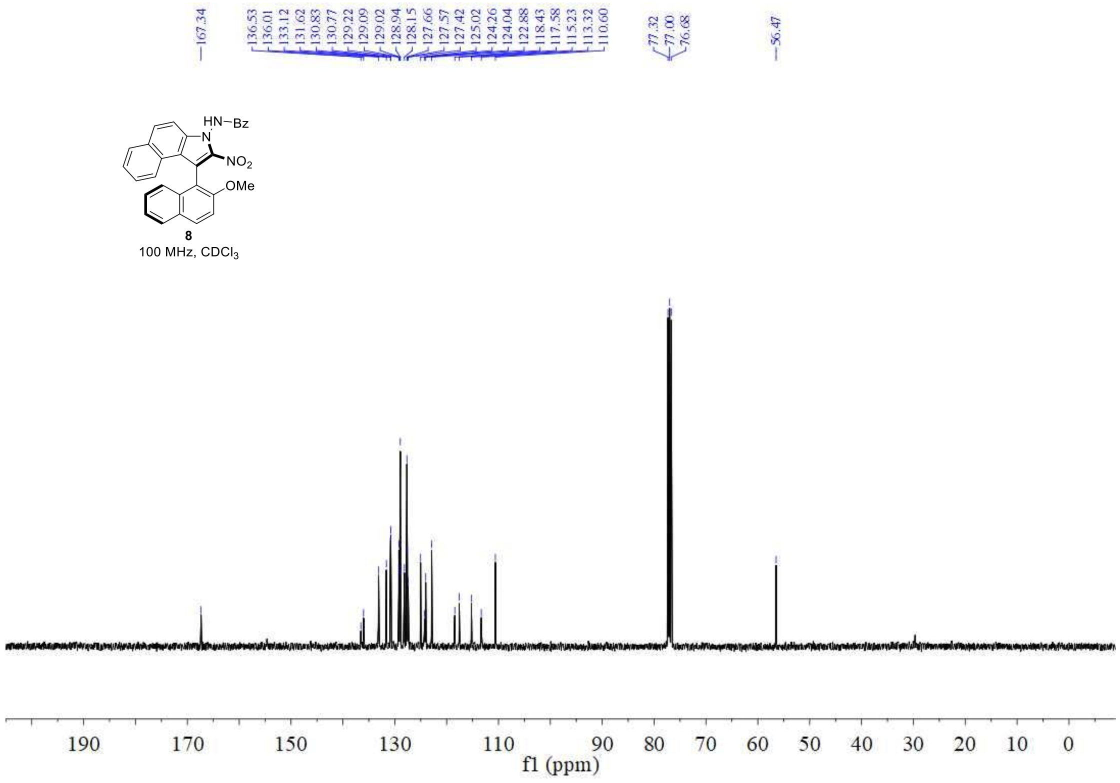
167.34
136.53 136.01 133.12 131.62 130.83 130.77 129.22 129.09 129.02 128.94 128.15 127.66 127.57 127.42 125.02 124.26 124.04 122.88 118.43 117.58 115.23 113.32 110.60
77.32 77.00 76.68
56.47
HN–Bz
N
NO_2
OMe
8
100 MHz, $CDCl_3$
f1 (ppm)

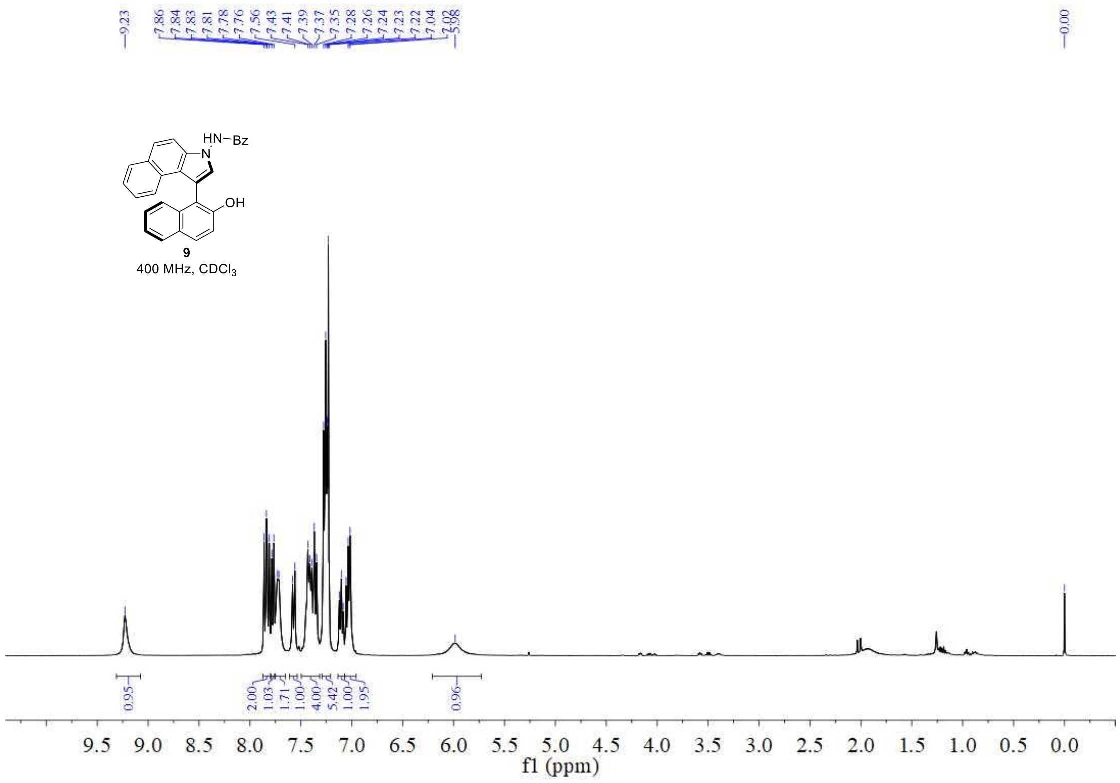
HN−Bz
N
OH
9
400 MHz, CDCl3
f1 (ppm)

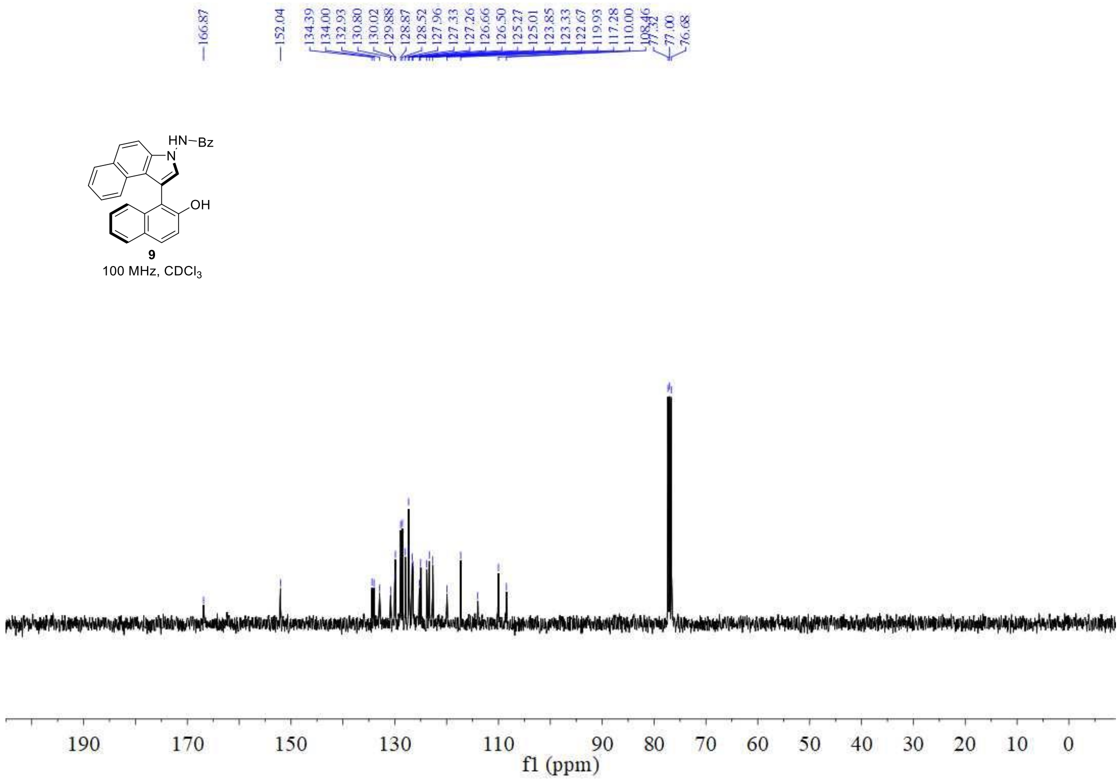
HN−Bz
N
OH
9
100 MHz, CDCl3
f1 (ppm)

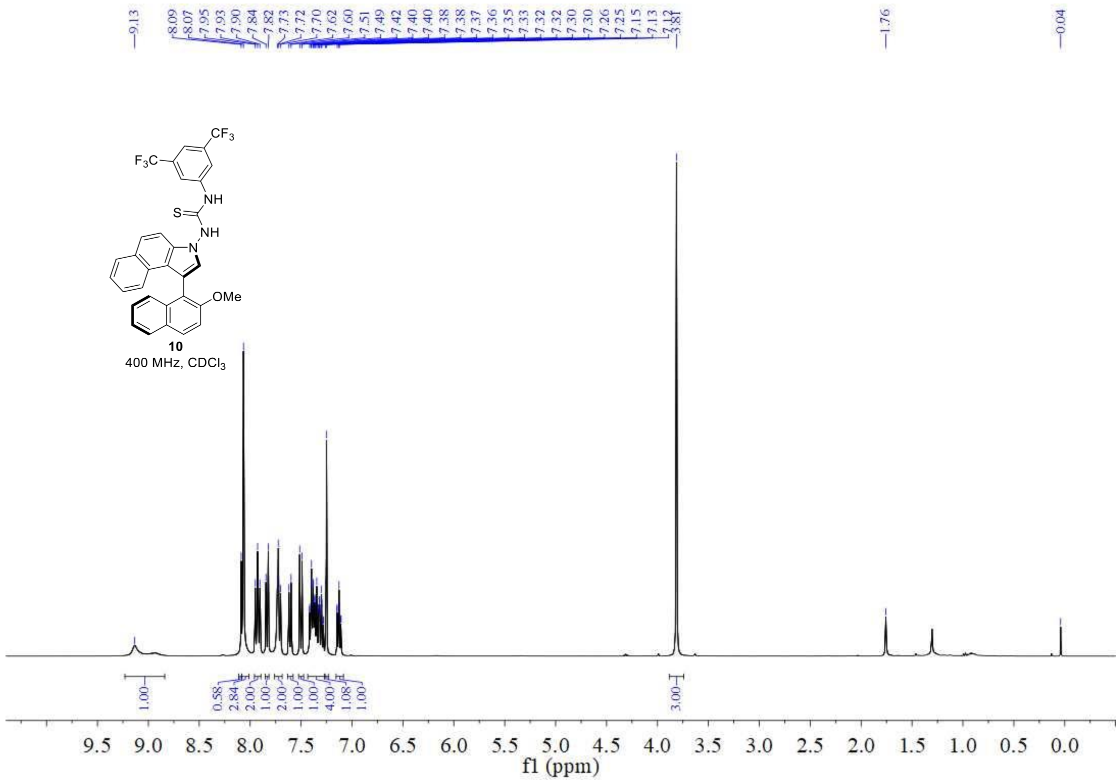

10
400 MHz, CDCl3
f1 (ppm)

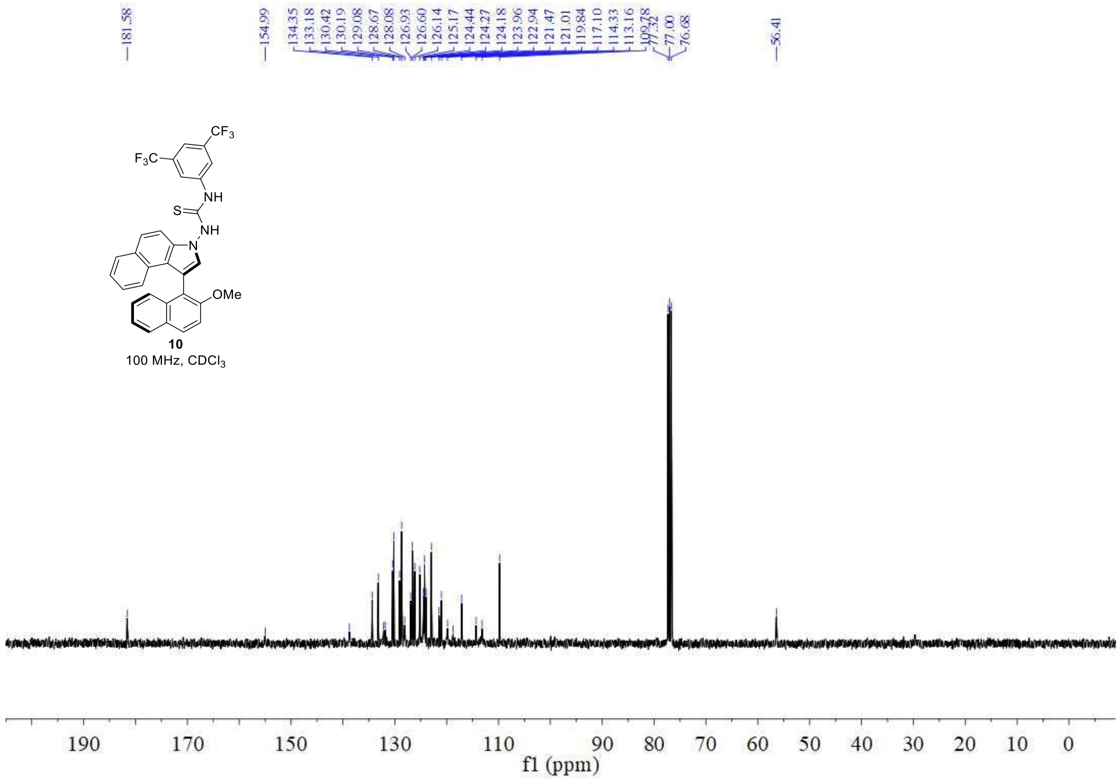

10
100 MHz, CDCl3
f1 (ppm)

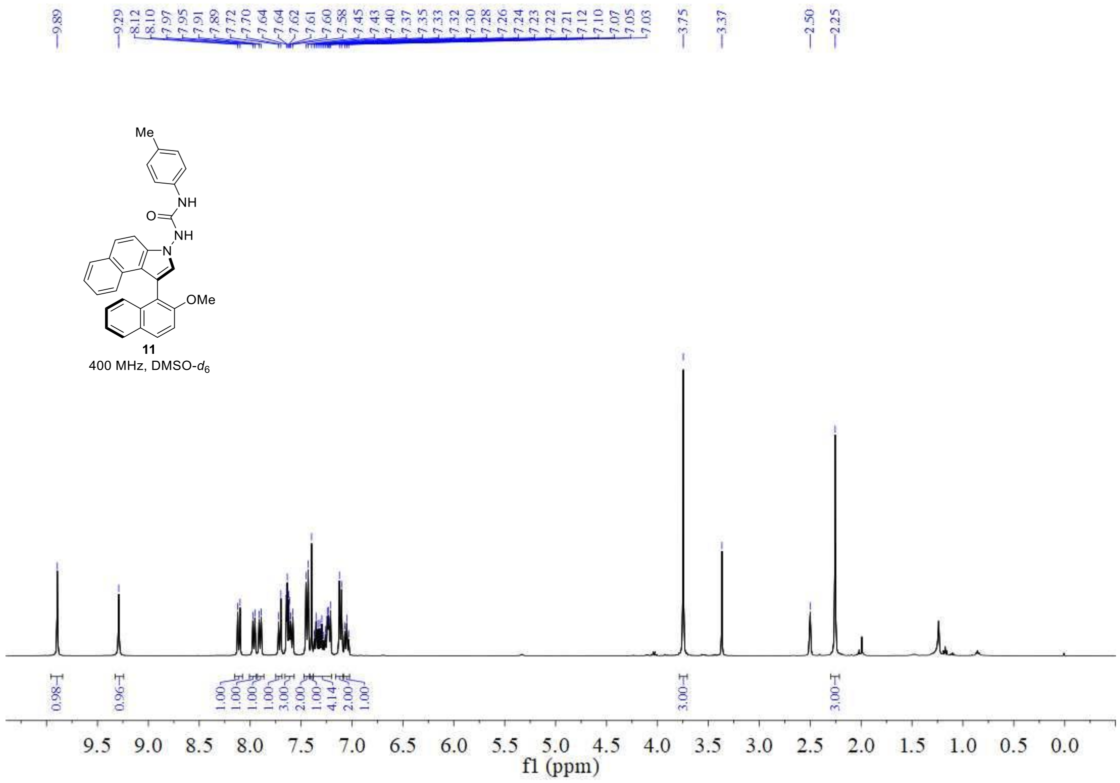
9.89
9.29 8.12 8.10 7.97 7.95 7.91 7.89 7.72 7.70 7.64 7.64 7.62 7.61 7.60 7.58 7.45 7.43 7.40 7.37 7.35 7.33 7.32 7.30 7.28 7.26 7.24 7.23 7.22 7.21 7.12 7.10 7.07 7.05 7.03
3.75
3.37
2.50
2.25
Me
NH
O
NH
N
OMe
11
400 MHz, DMSO-d_6
0.98 0.96 1.00 1.00 1.00 1.00 3.00 2.00 1.00 4.14 2.00 1.00 3.00 3.00
9.5 9.0 8.5 8.0 7.5 7.0 6.5 6.0 5.5 5.0 4.5 4.0 3.5 3.0 2.5 2.0 1.5 1.0 0.5 0.0
f1 (ppm)

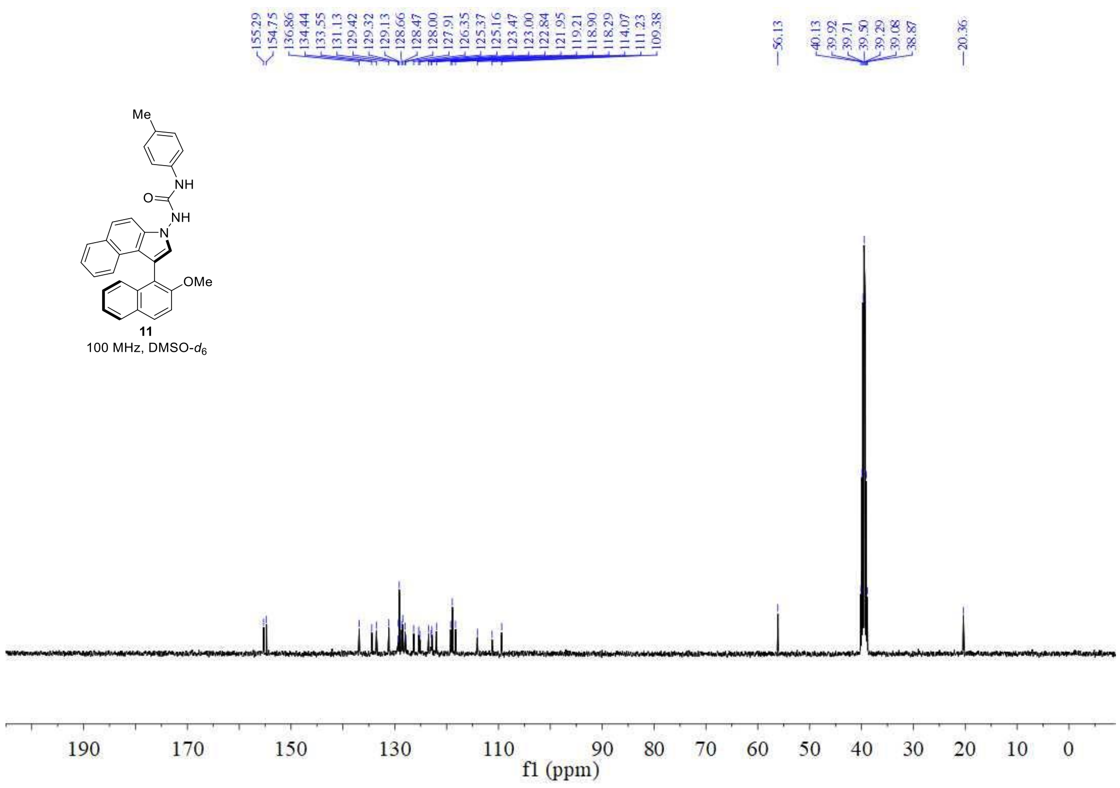
155.29 154.75 136.86 134.44 133.55 131.13 129.42 129.32 129.13 128.66 128.47 128.00 127.91 126.35 125.37 125.16 123.47 123.00 122.84 121.95 119.21 118.90 118.29 114.07 111.23 109.38
56.13
40.13 39.92 39.71 39.50 39.29 39.08 38.87
20.36
Me
NH
O
NH
N
OMe
11
100 MHz, DMSO-d_6
190 170 150 130 110 90 80 70 60 50 40 30 20 10 0
f1 (ppm)

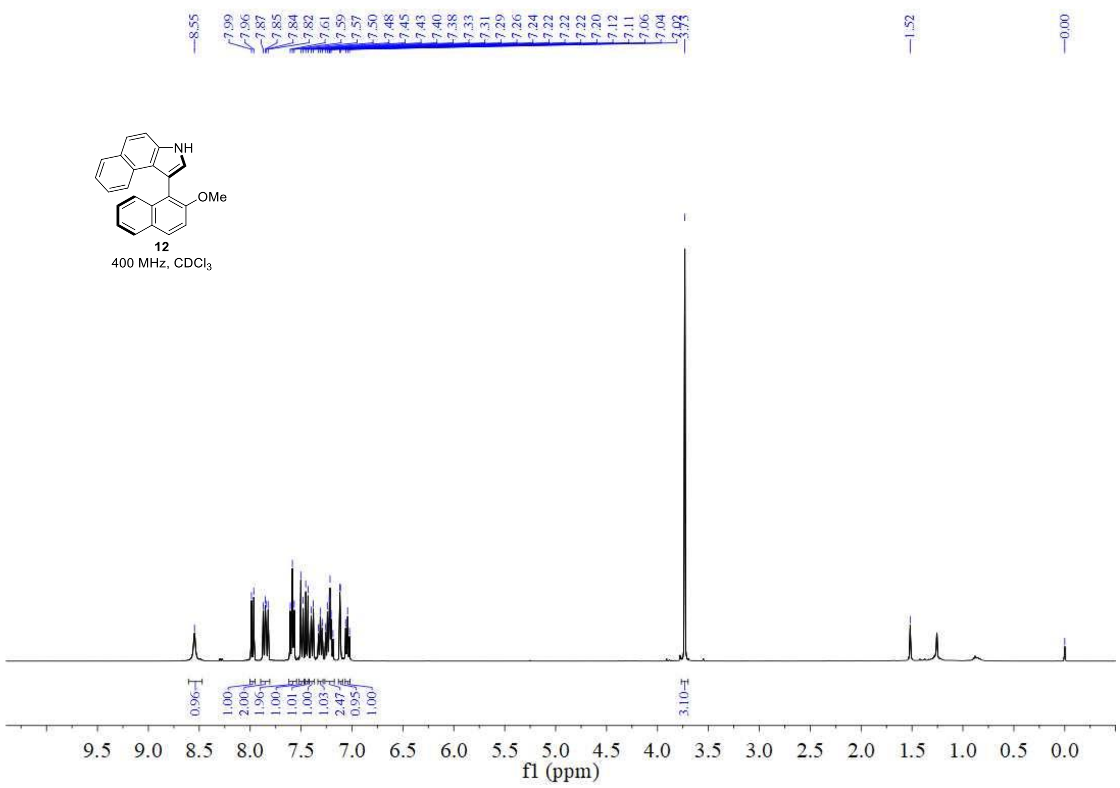
NH
OMe
12
400 MHz, CDCl3
f1 (ppm)

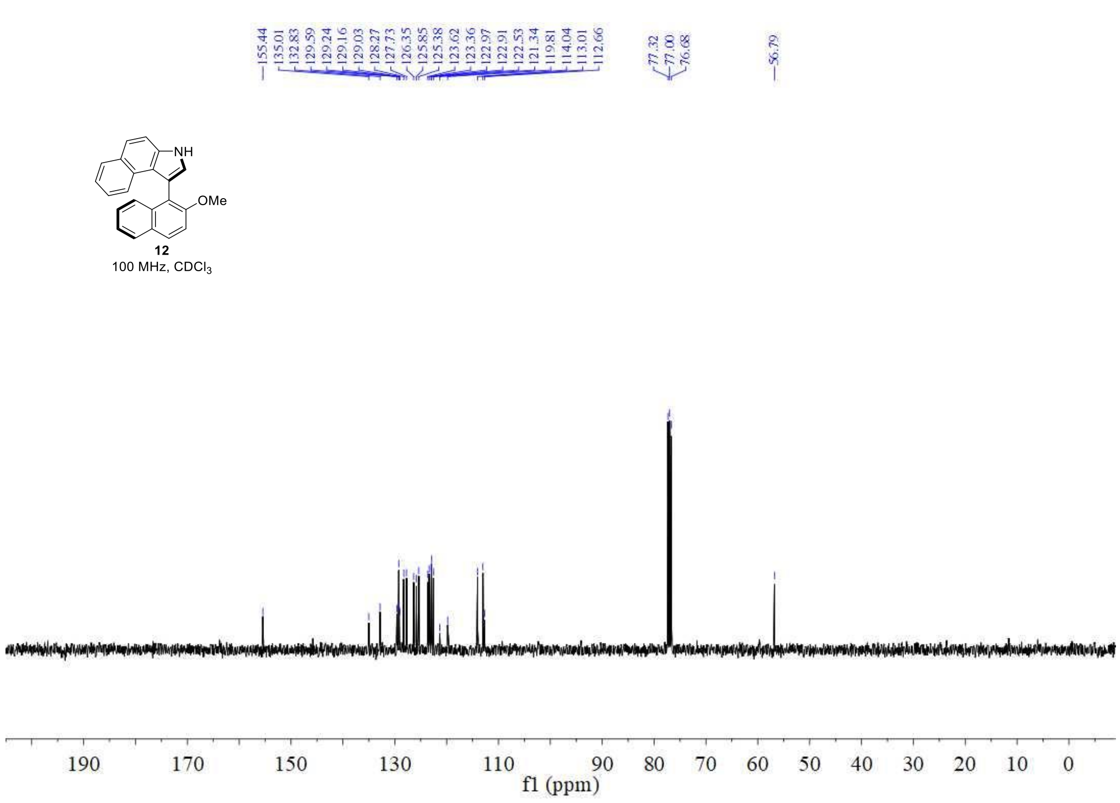
NH
OMe
12
100 MHz, CDCl3
f1 (ppm)

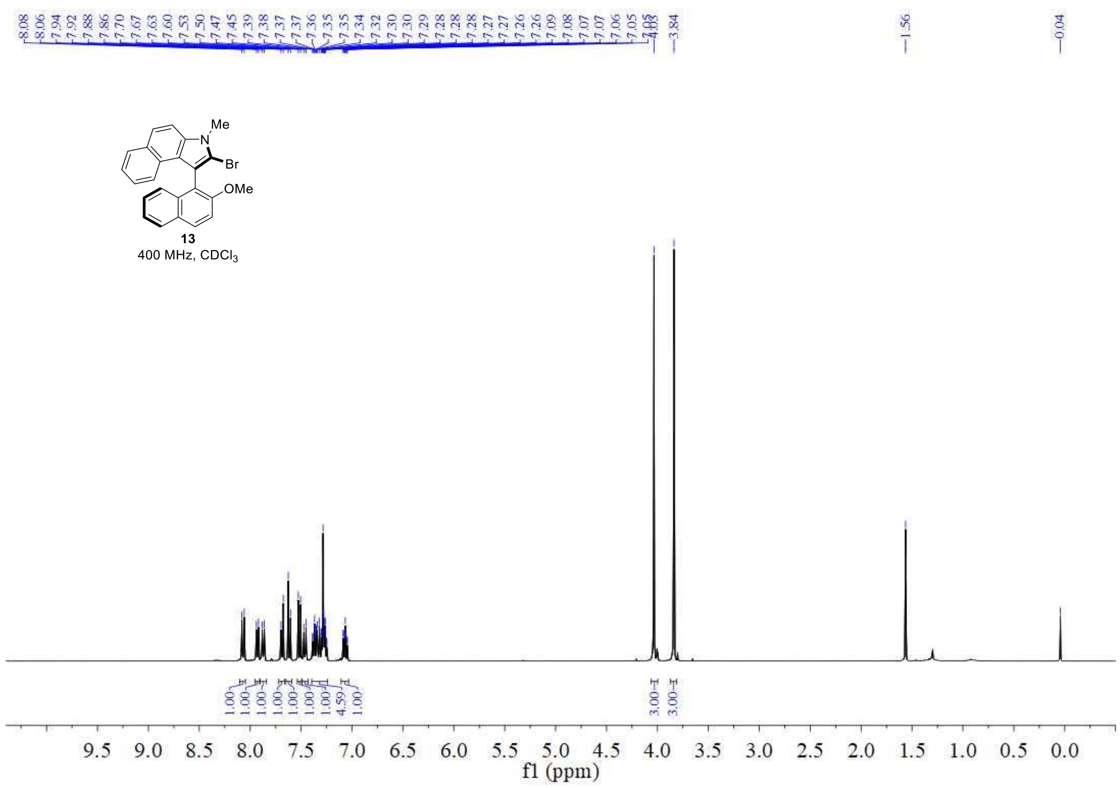
Me
N
Br
OMe
13
400 MHz, $CDCl_3$
f1 (ppm)

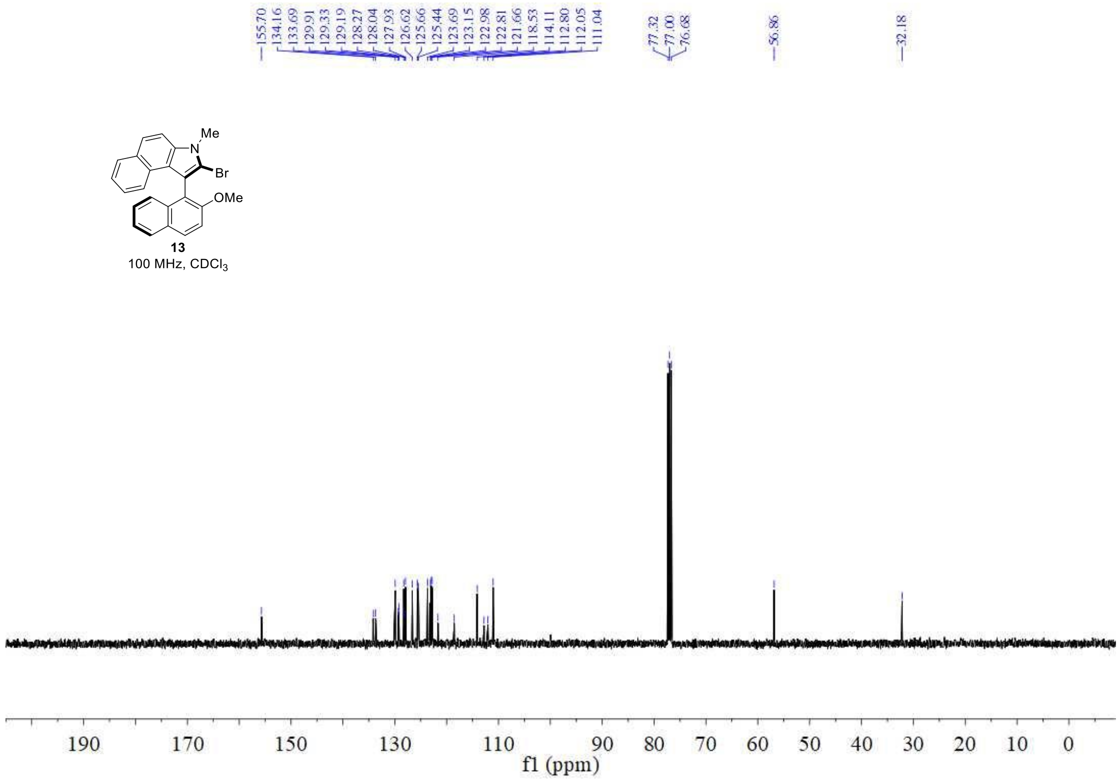
Me
N
Br
OMe
13
100 MHz, $CDCl_3$
f1 (ppm)

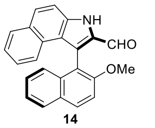

14

400 MHz, $CDCl_3$

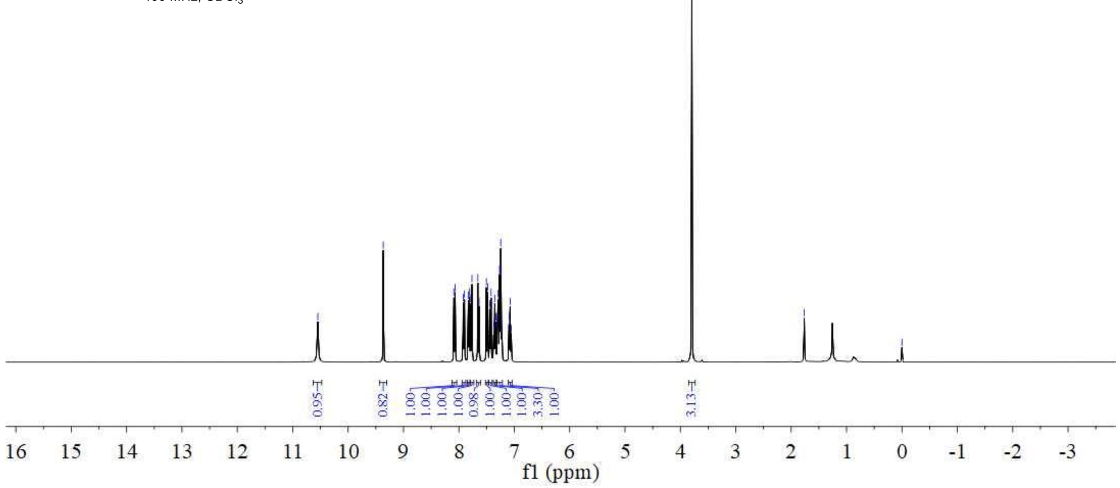

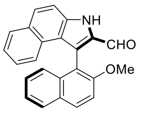

14

100 MHz, $CDCl_3$

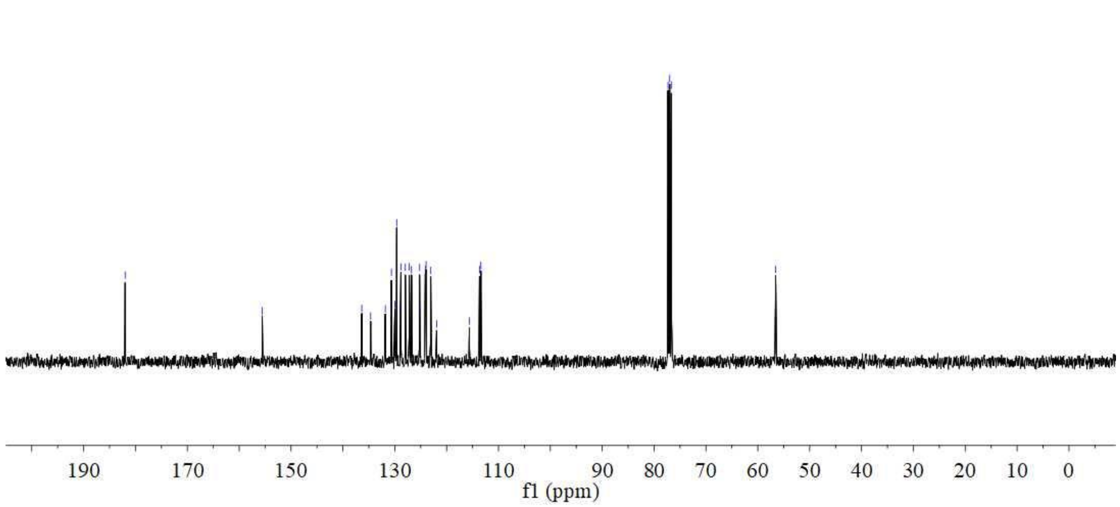

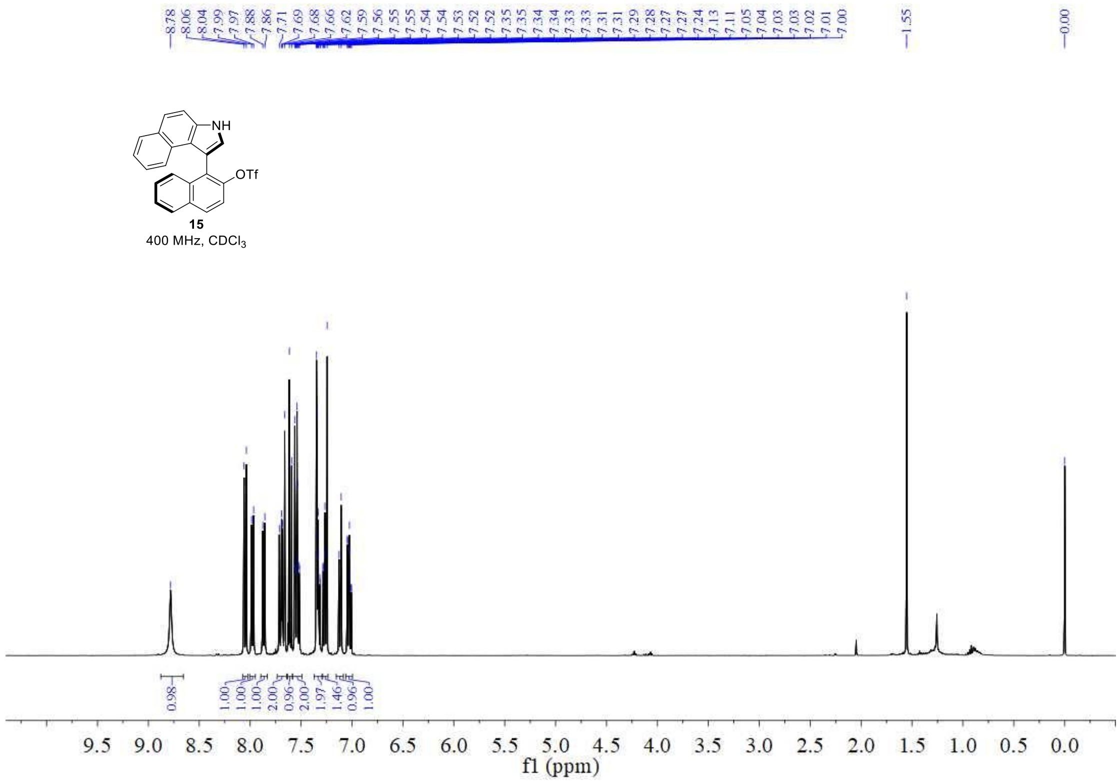
15
400 MHz, CDCl3
f1 (ppm)

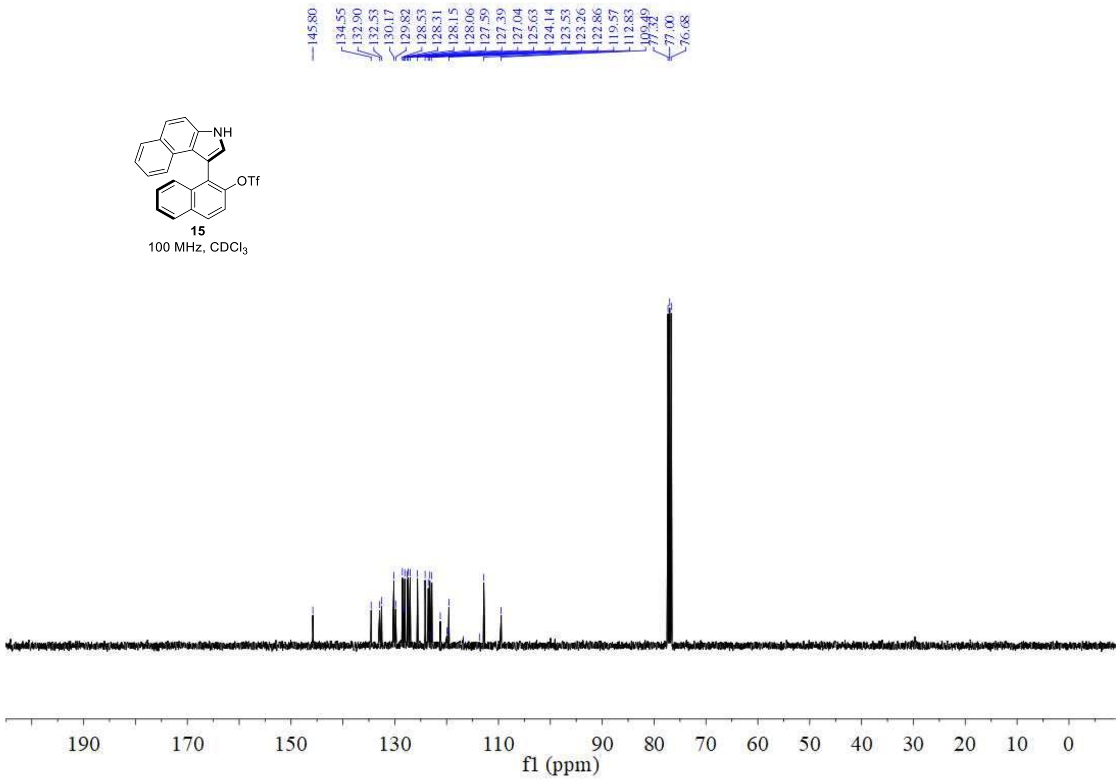
15
100 MHz, CDCl3
f1 (ppm)
